# Recycling fluoropolymers to acyl fluorides through shuttle catalysis

**DOI:** 10.1039/d6sc02698b

**Published:** 2026-06-29

**Authors:** Shannon E. S. Farley, Amanda A. Fogh, Mark R. Crimmin

**Affiliations:** a Department of Chemistry, Molecular Sciences Research Hub, Imperial College London 82 Wood Lane, White City London W12 0BZ UK m.crimmin@imperial.ac.uk

## Abstract

The chemical recycling of a small array of fluoropolymers including poly(vinylidene difluoride) is reported. Using a catalytic combination of BF_3_·OEt_2_ and BF_3_·PCy_3_, equivalents of HF can be harvested from these fluoropolymers and delivered to acid anhydrides to generate the corresponding acyl fluorides. Catalytic conditions were established and optimised using fluoroethane (HFC-161) as a model substrate and can be applied to a range of acid anhydrides, including acetic anhydride – paving the way for a scalable process. Mechanistic studies suggest that two competitive processes involving both hydrofluorination and fluoroalkylation of the acid anhydride are in operation. Spectroscopic studies in combination with DFT calculations shed light on the roles that BF_3_·OEt_2_ and BF_3_·PCy_3_ play in these processes. The methodology was applied to a series of pristine fluoropolymers (PVDF, PVF, ETFE, and HFP-PVDF) along with post-consumer materials including PVDF recovered from a Li-ion battery. Spectroscopic and thermal analyses (powder XRD, XPS, IR spectroscopy, DSC, and TGA) of the recovered fluoropolymer showed that the reaction occurs with decrease in the crystallinity of PVDF, with loss of the *α*-phase of the pristine material. Defluorination introduces new unsaturated C=C and oxygen-containing functional groups into the fluoropolymer and drastically lowers the onset temperature for its thermal decomposition. This approach has the potential not only to recycle the fluorine content of selected fluoropolymers but also create new fluorinated materials with different properties to pristine polymers.

## Abstract 2

The chemical recycling of a small array of fluoropolymers including poly(vinylidene difluoride) is reported.

## Graphical abstract

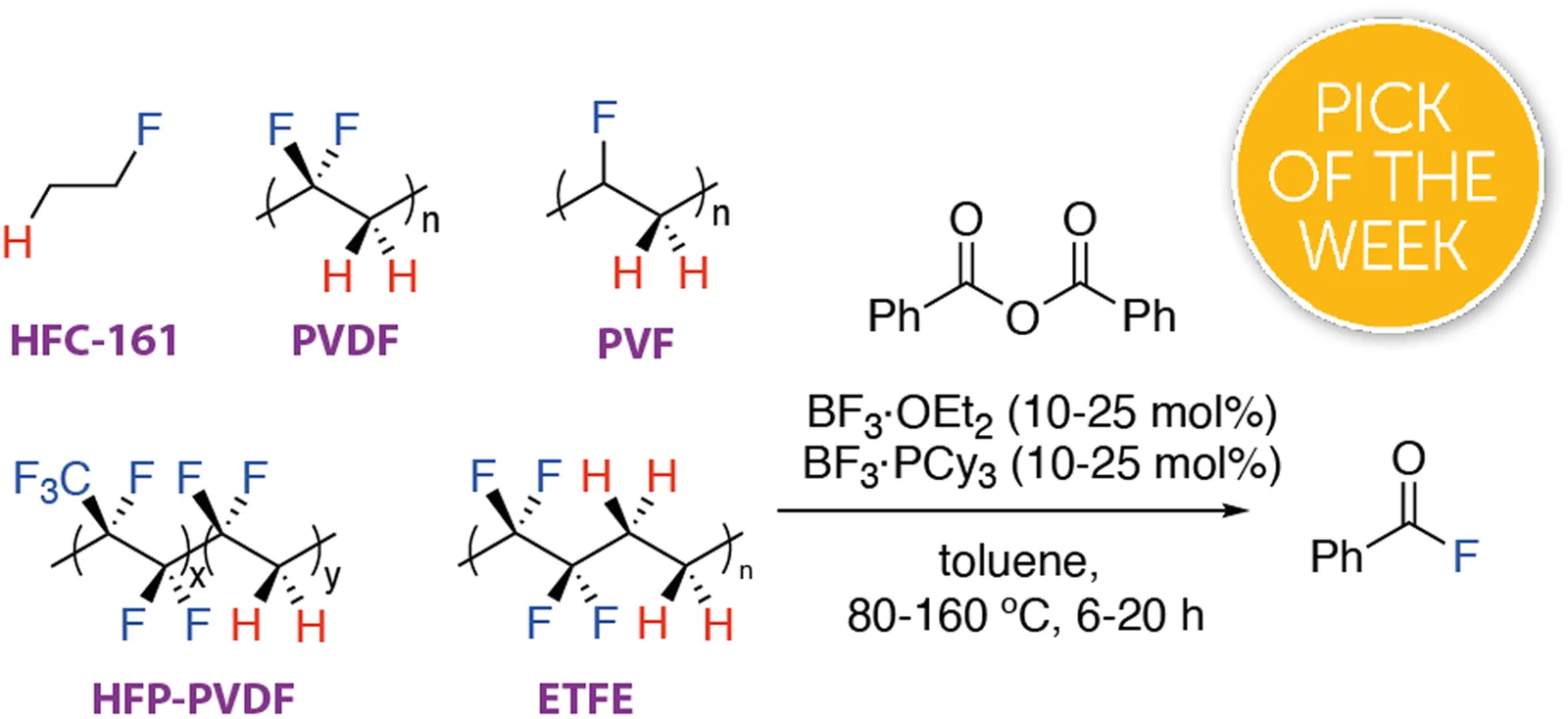

## Introduction

Fluorinated polymers (fluoropolymers) are widely used materials across the energy, healthcare, and construction sectors.^1–3^ In 2018, the global consumption of fluoropolymers was estimated as 320 kt.^4^ Poly(vinylidene difluoride) (PVDF) is the second largest contributor to this market. PVDF is a melt-processible thermoplastic that is used in moulded materials, coatings, and films. The demand for PVDF is expected to grow over the coming decades, in part due to its use a binding material for electrodes in Li-ion batteries.^5^ Despite their widespread use, recycling strategies for fluoropolymers are currently limited.^4,6,7^ It has been estimated that only 3.4% of fluoropolymers are recycled.^4^ PVDF remains challenging in this regard, as while it can be recovered and reprocessed a handful of times, it cannot easily be depolymerised back to its monomeric components above its ceiling temperature. Proposed changes to the REACH legislation have raised the possibility that certain fluoropolymers, including PVDF, will be classified as PFAS.^8,9^ Underpinning this proposition is increasing concern over the environmental fate and health impacts of fluorinated chemicals, including the potential degradation products of fluoropolymers.^10^

**Fig. 1:**
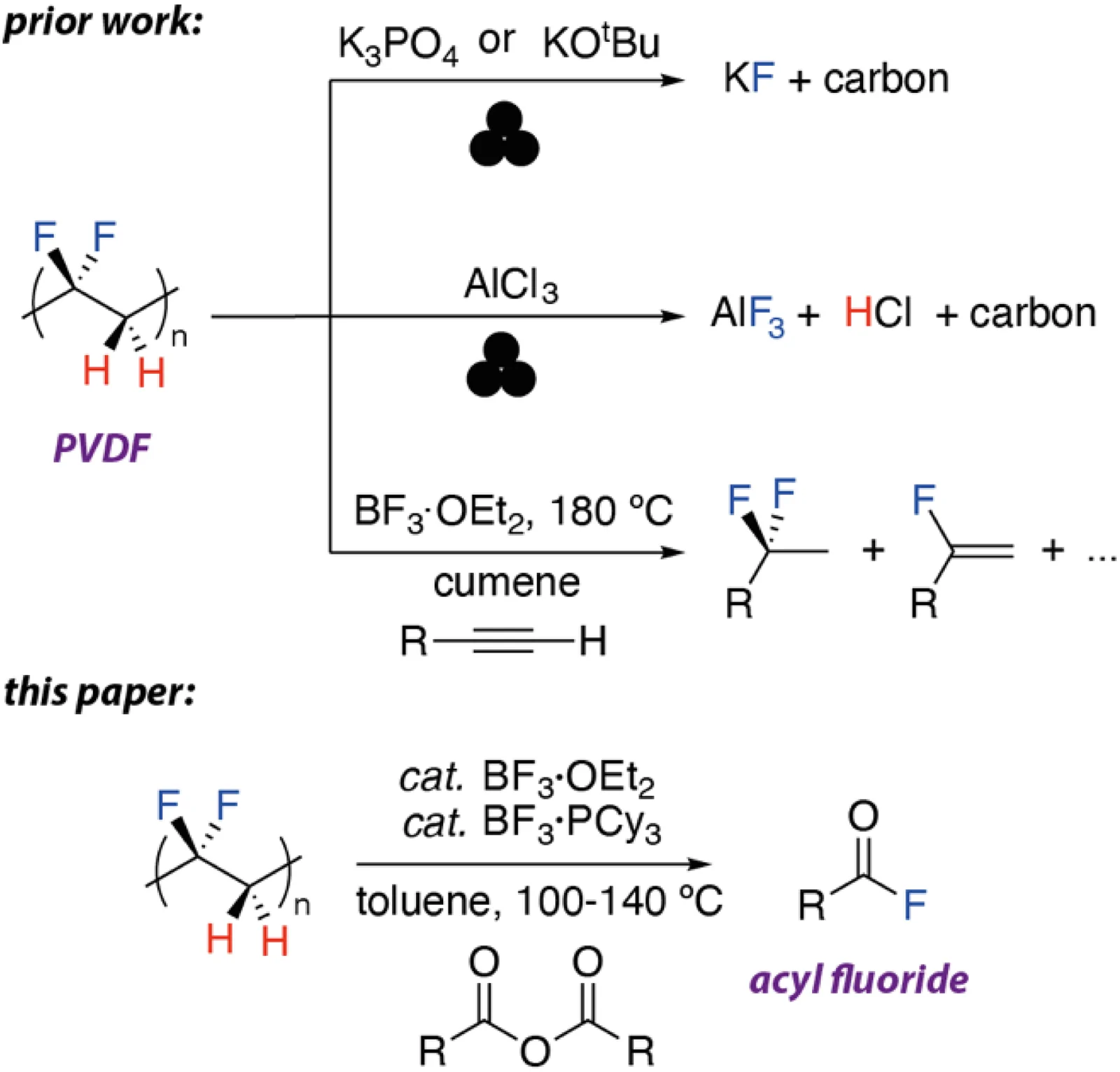
Selected examples of PVDF recycling by transfer fluorination and HF shuttling methods.

In recent years, several strategies have emerged for the chemical destruction of fluoropolymers into inorganic fluorides. This mineralisation approach requires the use of stoichiometric reagents (*e.g.* metals, bases, phosphate salts, and aluminium halides) and has been achieved with thermal, electrochemical, photochemical and mechanochemical conditions.^11–17^ Several research teams have shown that it is possible to mineralise fluoropolymers into useful forms of inorganic fluoride (*e.g.* LiF, NaF, KF, CaF_2_, or AlF_3_).^18–22^ In certain cases, the inorganic fluoride products have been shown to be competent reagents to fluorinate organic electrophiles allowing the fluorine content of fluoropolymers to be recycled through a two-step transfer fluorination process (Fig. 1).^23–30^ Although an encouraging step toward a circular economy for fluorine, these approaches are not without their drawbacks. The use of a two-step process and stoichiometric reagents to promote defluorination of these polymers potentially limits their scalability. Furthermore, complete mineralisation of fluoropolymers necessarily occurs with generation of carbon-containing side-products including methane, along with formate, carbonate, oxalate salts and amorphous carbon which potentially need to be separated from the fluoride containing products.^18–21,24,25,27^

An alternative approach to recycling fluoropolymers, which complements this mineralisation strategy, involves their partial defluorination. Partial defluorination involves removal of some of the fluorine content while keeping the carbon backbone of the polymer intact. It has the potential to create modified fluoropolymer materials that might not be classified as PFAS, while also harvesting and recovering the fluorine content in a useable form. If achieved through the use of catalysis, partial defluorination strategies for fluoropolymer recycling could complement mineralisation approaches.

Recently we, and others, reported a method for catalytic shuttling of HF between fluoroalkanes and alkynes.^31,32^ As part of these studies, we demonstrated that treatment of PVDF with BF_3_·OEt_2_ (1 equiv.) in the presence of an alkyne at 180 °C allowed the shuttling of HF equivalents from the polymer to the alkyne, creating a mixture of fluoroalkane and fluoroalkene products (Fig. 1). While demonstrating proof-of-concept, this system was impractical for further development due to the high temperatures required, the high loading of BF_3_·OEt_2_, and the generation of fluorine-containing products that have limited synthetic use. Herein, we report a next generation protocol for recycling of PVDF (and related fluoropolymers) through partial defluorination. We identified acid anhydrides as suitable reaction partners to accept HF from PVDF, allowing creation of acyl fluorides as easily accessible and versatile fluoride-carriers for use in synthesis (Fig. 1). We demonstrate improvements in catalyst performance, characterise the new materials that result, and apply the methodology to post-consumer products.

## Results and discussion

### Reaction discovery

During investigation of the reaction between PVDF powder (*M*_w_ = 534 000) and 1-dodecyne catalysed by BF_3_·OEt_2_, we discovered that phosphine bases were beneficial additives for catalysis and that acid anhydrides could be used as suitable reaction partners in place of the alkyne (see the SI).^31^ The discovery of the beneficial effect of phosphines was important as it allowed the reaction time and temperature to be decreased from our previously reported conditions. The use of acid anhydrides vastly simplified product distributions generating acyl fluorides as the only fluorine containing product in solution. Acyl fluorides are easy to handle and versatile fluoride carriers that find broad applications in the synthesis of fluorochemicals.^33^ To further optimise and better understand this reaction, we chose to focus on fluoroethane (HFC-161) as a fluorine donor in place of PVDF. Fluoroethane is a niche use refrigerant that has been proposed as a replacement for difluorochloromethane (HCFC-22) in air-conditioning units.^34^ While its recycling is an important challenge in its own right,^35^ in the current case it was investigated as it simplified not only analysis of the reaction products by ^1^H and ^19^F NMR spectroscopy, but also DFT modelling of potential mechanisms (*vide infra*).

**Fig. 2:**
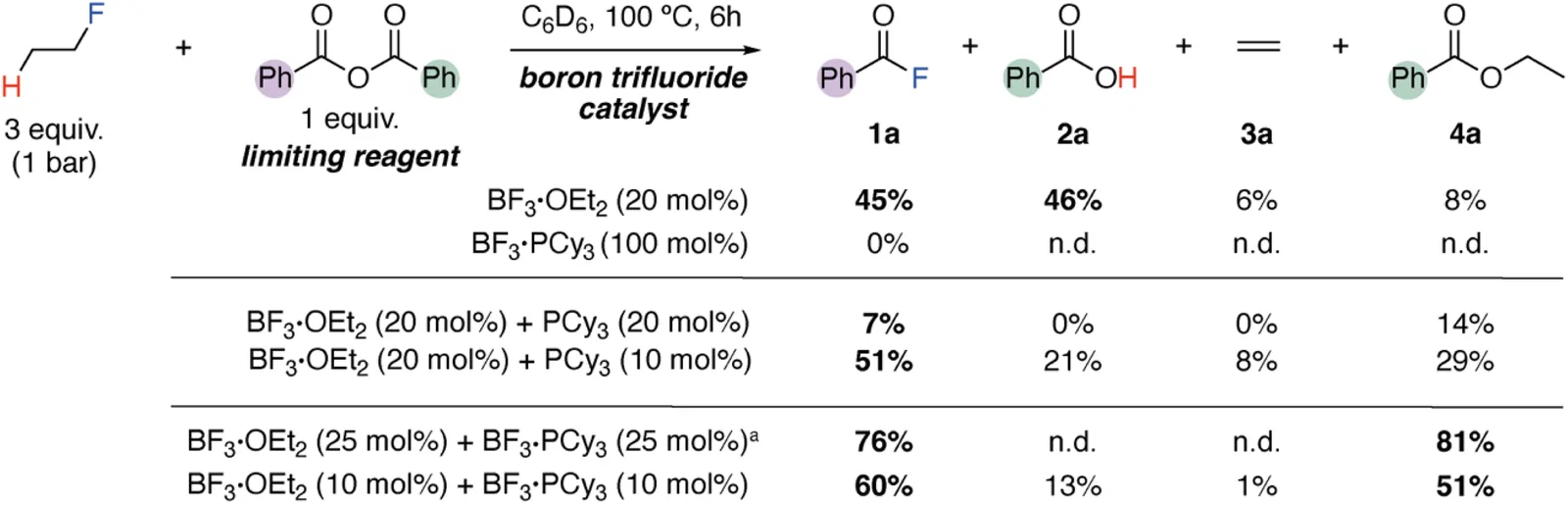
Catalytic HF shuttling reaction of fluoroethane (HFC-161) with benzoic anhydride. Yields determined by ^19^F NMR and ^1^H NMR against fluorobenzene and ferrocene as an internal standard, respectively. ^a^10 h reaction time.

The reaction of 3 equiv. of fluoroethane (1 bar) with benzoic anhydride catalysed by BF_3_·OEt_2_ and PCy_3_ was investigated (Fig. 2). In the reaction with 20 mol% BF_3_·OEt_2_, benzoyl fluoride (1a), benzoic acid (2a), ethene (3a) and ethyl benzoate (4a) are formed in 45%, 46%, 6% and 8% yield, respectively. Benzoyl fluoride is the sole fluorine-containing product of this reaction. Given its volatility, the amount of ethene produced is likely underestimated by quantification using NMR spectroscopy. Yields are calculated based on benzoic anhydride as the limiting reagent assuming that 1 equiv. of anhydride can only generate 1 equiv. of acyl fluoride. A control reaction in the absence of fluoroethane showed no formation of benzoyl fluoride (1a).

Tricyclohexylphosphine (PCy_3_) was investigated as an additive. A ratio of 1 : 1 of BF_3_·OEt_2_ to PCy_3_ led to limited product formation. However, using a 2 : 1 mixture of BF_3_·OEt_2_ and PCy_3_ gave the 1a, 2a, 3a and 4a in a 51%, 21%, 8% and 29% distribution. Hence, the major side-product of the reaction switches from benzoic acid to ethyl benzoate in the presence of PCy_3_. This result is significant, as it suggests that a switch in mechanism may be occurring when PCy_3_ is present in the catalytic mixture. BF_3_·PCy_3_ was prepared through independent synthesis and tested as a catalyst in the HF shuttling procedure; this was also inactive by itself. Using a mixture of BF_3_·OEt_2_ and BF_3_·PCy_3_, however reestablished catalytic activities at levels commensurate with those obtained with PCy_3_ as an additive. Ultimately, through variation of the temperature, solvent, and catalyst loading, 25 mol% of each BF_3_·OEt_2_ and BF_3_·PCy_3_ at 100 °C in toluene were identified as high-yielding conditions for HF shuttling. Decent yields were still obtained using 10 mol% of each catalyst and were deemed optimum for further reactions.

**Fig. 3:**
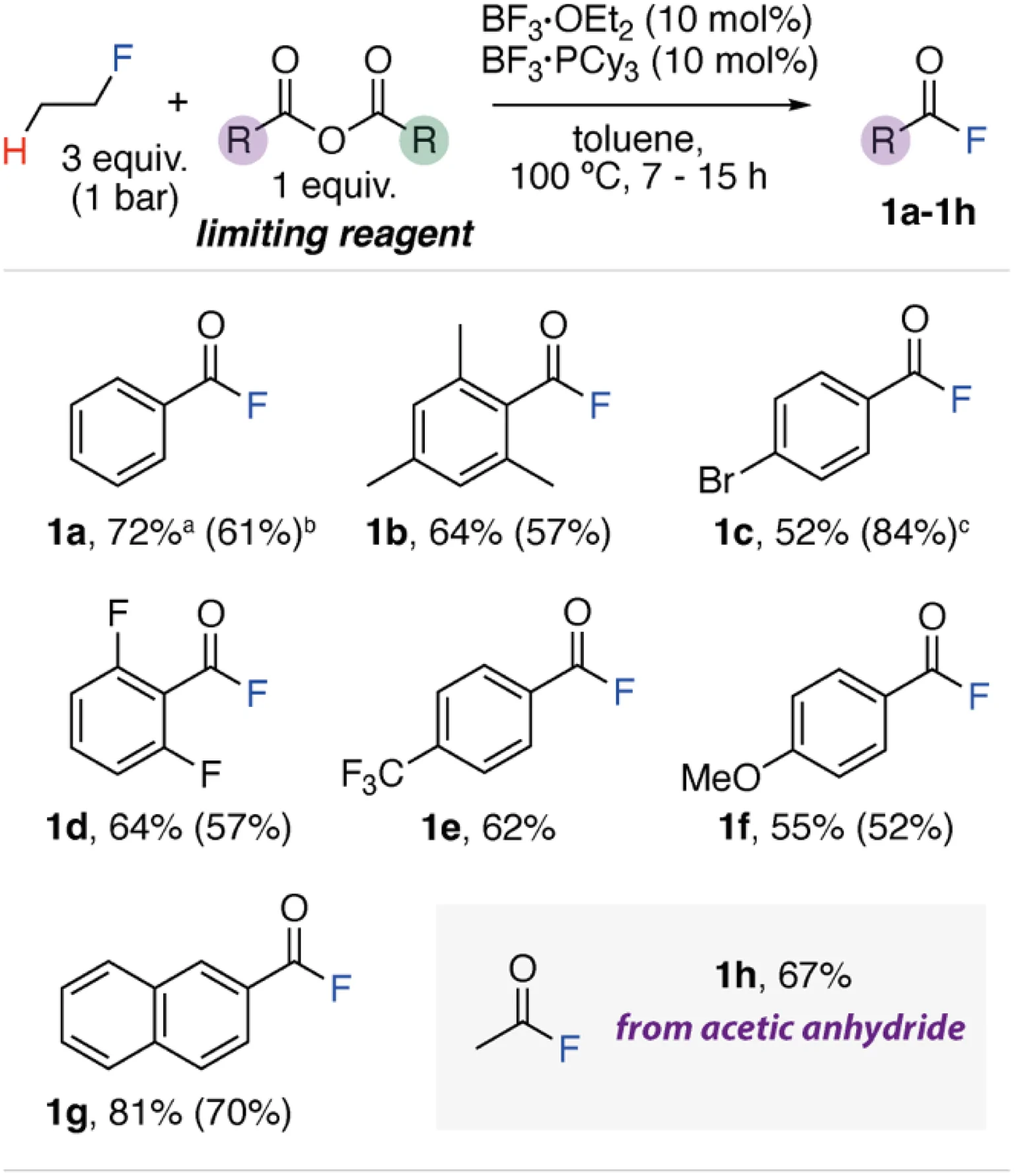
Array of benzoyl fluorides synthesised from HF shuttling from fluoroethane (HFC-161). ^a^Yields determined by ^19^F NMR spectroscopy using fluorobenzene as an internal standard. ^b^Isolated yields are given in parentheses. ^c^The improved yield is likely due to improved mixing on scale-up as the anhydride is only partially soluble under the reaction conditions.

An array of acid anhydrides was investigated using the optimised HF shuttling conditions with fluoroethane to form the corresponding acyl fluorides 1a–h in 52–81% *in situ* and 52–70% isolated yield (Fig. 3). In the case of non-volatile compounds, the desired acyl fluorides were readily isolable by column chromatography. It is particularly notable that acetic anhydride can be used in this reaction as it is an inexpensive and readily available bulk chemical whose production is an order of magnitude larger than fluoropolymer production. Hence, this protocol represents a viable first step to scalable fluoride recovery from fluorocarbons available on industrial scales.^36^

### Mechanism of HF shuttling

Recent studies have demonstrated that combinations of Lewis acids and Lewis bases are effective reagents for the defluorination of fluorocarbons, including the selective functionalisation of both fluoroalkane and geminal difluoroalkane groups, similar to those found in fluoroethane and PVDF.^37–43^ Curious as to the role of PCy_3_ in promoting reactivity and changing the product distribution, a series of experiments and DFT calculations were undertaken.

**Fig. 4:**
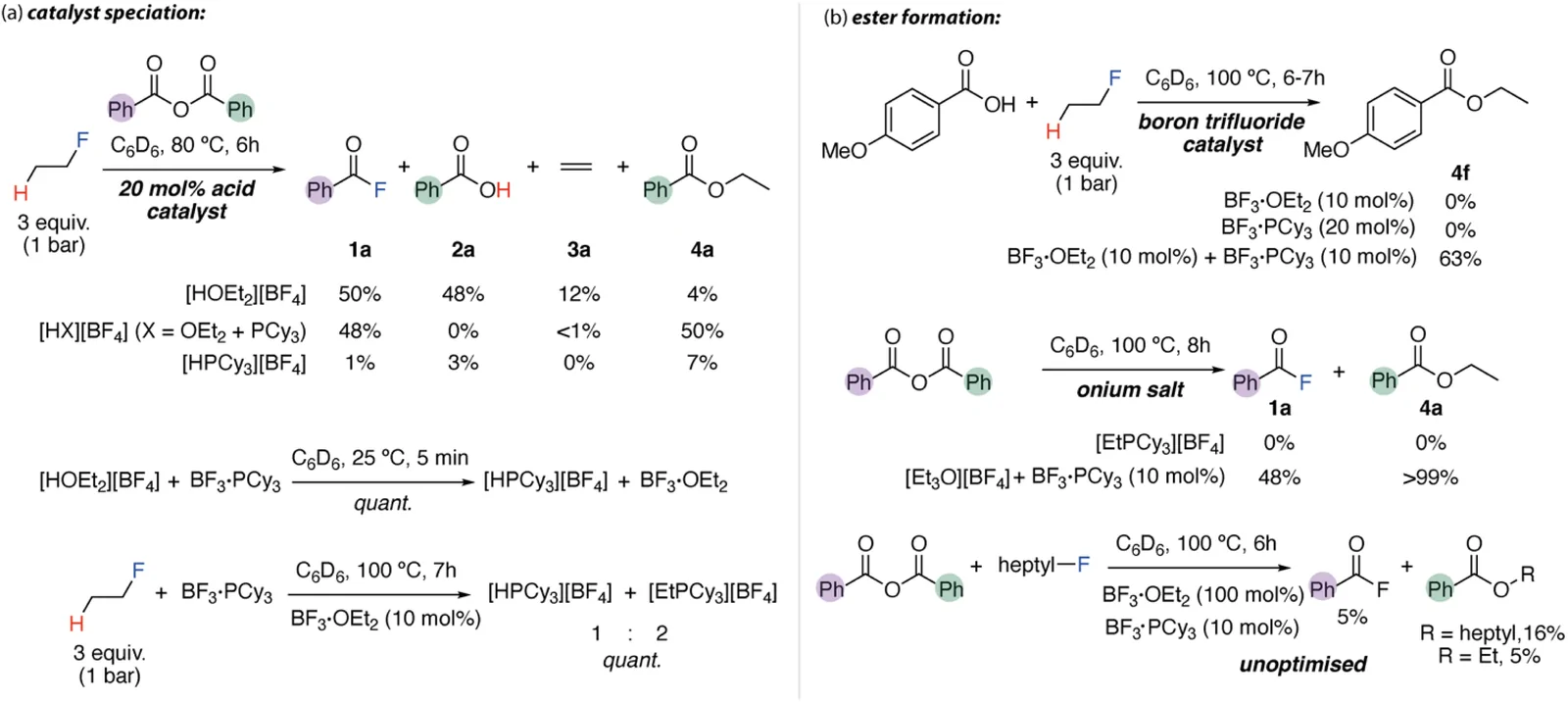
Experiments to probe (a) catalyst speciation and (b) the mechanism of ester formation. *In situ* yields measured by ^19^F and ^1^H NMR against fluorobenzene and ferrocene as an internal standard.

Catalyst speciation was initially considered. Monitoring catalytic reaction mixtures by ^31^P{^1^H} NMR spectroscopy revealed that both [HPCy_3_][BF_4_] and BF_3_·PCy_3_ were present as evidenced by resonances at *δ* = 28.0 ppm (s) and *δ* = −3.5 ppm (qq, ^2^*J*_P–F_ = 203.5, ^1^*J*_B–P_ = 156.5 Hz) respectively. [HOEt_2_][BF_4_] was shown to be catalytically competent at 20 mol% loading for the HF shuttling from fluoroethane to benzoic anhydride to form 1a, 2a, 3a and 4a. Mixtures of 10 mol% [HOEt_2_][BF_4_] and 10 mol% [HPCy_3_][BF_4_] were also catalytically active, but biased side-product formation toward ethyl benzoate. Attempts to catalyse the process by 20 mol% [HPCy_3_][BF_4_] alone led to minimal product formation, consistent with the low activity of BF_3_·PCy_3_ (Fig. 4a).

A stoichiometric reaction between [HOEt_2_][BF_4_] and BF_3_·PCy_3_ instantaneously produced [HPCy_3_][BF_4_] and BF_3_·OEt_2_. The reaction between BF_3_·PCy_3_ and fluoroethane in the presence of 10 mol% BF_3_·OEt_2_ also provided evidence for the formation of [HPCy_3_][BF_4_] observed at *δ*_31P_ = +28.7 ppm by ^31^P NMR spectroscopy, along with a second phosphonium salt determined to be the product of fluoroalkylation of the phosphine [EtPCy_3_][BF_4_]; these species formed in a 1 : 2 ratio in quantitative yield.^43^ [EtPCy_3_][BF_4_] was prepared by independent synthesis from addition of PCy_3_ to [Et_3_O][BF_4_] and is characterised by diagnostic resonances at *δ*_19F_ = −149.9 ppm, *δ*_31P_ = 32.6 ppm, and *δ*_11B_ = −0.3 ppm apparent in multinuclear NMR spectra. The phosphonium ion [EtPCy_3_]^+^ was readily observed by positive-mode mass spectrometry with *m*/*z* = 309.26.

The plausible routes to ester formation were considered. Reaction of 4-methoxybenzoic acid with fluoroethane in the presence of 1 equiv. of BF_3_·OEt_2_ did not yield the corresponding ethyl benzoate 4f. In contrast, when catalysed by 10 mol% BF_3_·PCy_3_ and 10 mol% BF_3_·OEt_2_4f was observed in 63% yield. To confirm the origin of the ethyl group in this experiment a further reaction between 1-fluoroheptane and benzoic acid catalysed by 10 mol% BF_3_·PCy_3_ and 100 mol% BF_3_·OEt_2_ was conducted, which gave a mixture of *n*-heptyl benzoate (16%) and ethyl benzoate (5%), suggesting that it is possible to incorporate the ethyl group from the catalyst into the product.

**Fig. 5:**
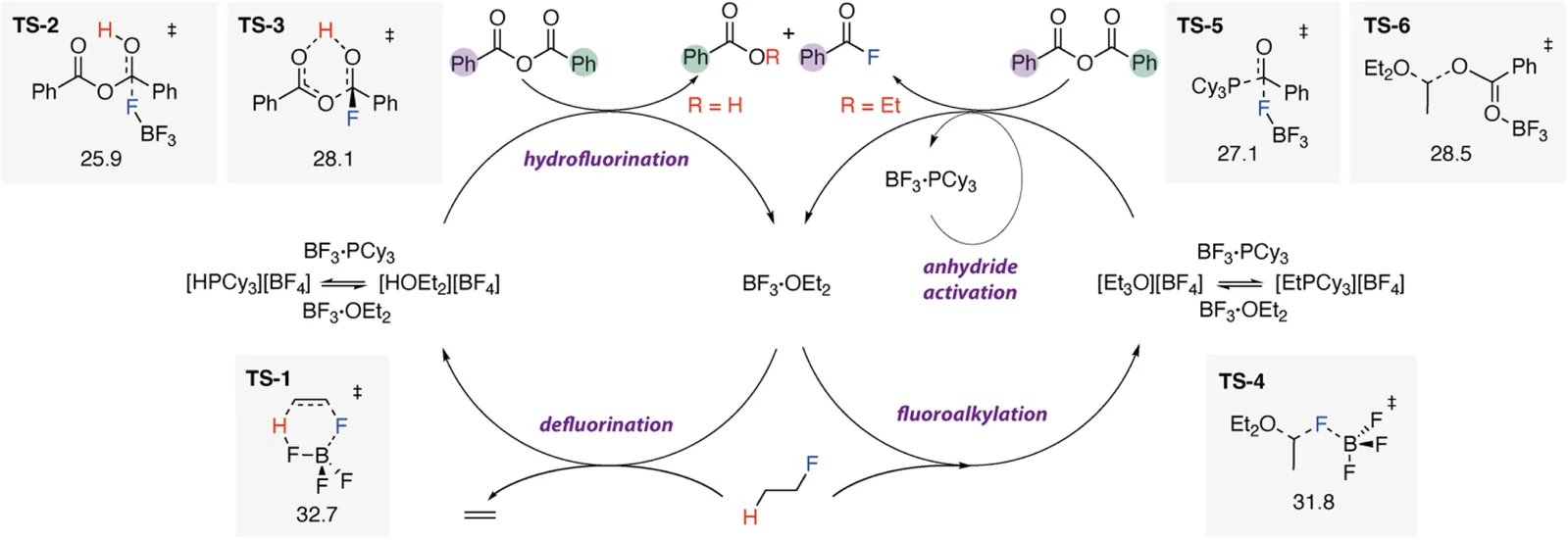
Proposed mechanism of HF shuttling of acid anhydride catalysed by BF_3_·PCy_3_ and BF_3_·OEt_2_ mixtures. Calculated transition state energies for key steps in the proposed cycle. B3LYP-GD3BJ/def2-QVPPD/SMD(toluene)//B3LYP-GD3BJ/6311++G**/PCM(toluene). Energies in kcal mol^−1^ corrected to 373 K and 0.1 M in anhydride, given relative to zero-point for each cycle.

In combination these experiments shed light on the precise mechanism of catalysis. We suggest that the complete product distribution can be accounted for by two connected catalytic cycles involving (i) a dehydrofluorination–hydrofluorination sequence and (ii) fluoroalkylation of the acid anhydride, in which BF_3_·PCy_3_ and BF_3_·OEt_2_ play myriad roles (Fig. 5). Here we define dehydrofluorination as the removal of HF from a molecule.

In the absence of BF_3_·PCy_3_ (or PCy_3_) it appears that the dehydrofluorination–hydrofluorination sequence is the major pathway. This mechanism is initiated by dissociation of Et_2_O from BF_3_ followed by association of BF_3_ with fluoroethane to form an intermediate which can undergo concerted HF elimination from fluoroethane through TS-1 to form ethene and [HOEt_2_][BF_4_]. The Gibbs activation energy of this step was calculated as Δ*G*^‡^_373K_ = 32.7 kcal mol^−1^ in an overall slightly endergonic process, Δ*G*^°^_373K_ = 2.5 kcal mol^−1^ with generation of [HOEt_2_][BF_4_] being slightly uphill. Calculations also support a viable pathway for hydrofluorination of benzoic anhydride from [HOEt_2_][BF_4_] involving an addition–elimination process. Initial proton transfer from [HOEt_2_][BF_4_] to benzoic anhydride occurs with subsequent nucleophilic attack of a fluoride from the BF_4_ anion *via*TS-2 (Δ*G*^‡^_373K_ = 25.9 kcal mol^−1^) forming the tetrahedral intermediate which can collapse to benzoyl fluoride (1a) and benzoic acid (2a) through TS-3 (Δ*G*^‡^_373K_ = 28.1 kcal mol^−1^). NBO analysis of these pathways is entirely consistent with charge distributions, bond breaking, and bond making expected from a textbook addition–elimination mechanism. Although BF_3_·PCy_3_ is not an on-cycle intermediate that is involved in the dehydrofluorination–hydrofluorination sequence, it is expected to impact catalyst speciation and stability through the off-cycle equilibrium that forms [HPCy_3_][BF_4_].

The overall transformation of fluoroethane and benzoic anhydride to a mixture of benzoyl fluoride and benzoic acid is exergonic with Δ*G*^°^_373K_ = −7.6 kcal mol^−1^. Comparison of the thermodynamics of this process to that reported for HF shuttling from fluoroethane to propyne to form 2-fluoroprop-1-ene and ethene (Δ*G*^°^_373K_ = −13.9 kcal mol^−1^) suggests that the reaction could be reversible.^31^ Combining benzoyl fluoride (1a), benzoic acid (2a), an alkyne, BF_3_·PCy_3_ (10 mol%) and BF_3_·OEt_2_ (10 mol%) and heating for 18 h at 80 °C revealed formation of a mixture fluoroalkene and difluoroalkane products in 20% yield, suggesting that the reaction is at least partially reversible (see the SI). Hence it is appropriate to describe it as a shuttling process.^44^

In the presence of BF_3_·PCy_3_ (or PCy_3_) a second catalytic process involving fluoroalkylation of benzoic anhydride can become competitive with the dehydrofluorination–hydrofluorination cycle. This pathway is initiated from a Lewis-acid assisted nucleophilic substitution of fluoroethane by BF_3_·OEt_2_ to form the oxonium salt [Et_3_O][BF_4_] through TS-4 (Δ*G*^‡^_373K_ = 31.8 kcal mol^−1^). For comparison, nucleophilic displacement with PCy_3_ directly from BF_3_·PCy_3_ was found to be a higher energy process, likely due to the energetic cost of phosphine dissociation from BF_3_. Nevertheless, [Et_3_O][BF_4_] can react with BF_3_·PCy_3_ to form the experimentally observed phosphonium salt [EtPCy_3_][BF_4_] which is proposed to be an off-cycle resting state. Alkylation of benzoic anhydride does not likely occur through a direct reaction with [Et_3_O][BF_4_]. Rather DFT calculations suggest that BF_3_·PCy_3_ first activates the anhydride to form the ion-pair [PhC(O)PCy_3_]^+^ [PhCO_2_BF_3_]^−^ which can then react with the oxonium salt through two separate pathways; forming benzoyl fluoride *via*TS-5 (Δ*G*^‡^_373K_ = 27.1 kcal mol^−1^) and ethyl benzoate *via*TS-6 (Δ*G*^‡^_373K_ = 28.5 kcal mol^−1^).^45^

### Application to fluoropolymers

**Fig. 6:**
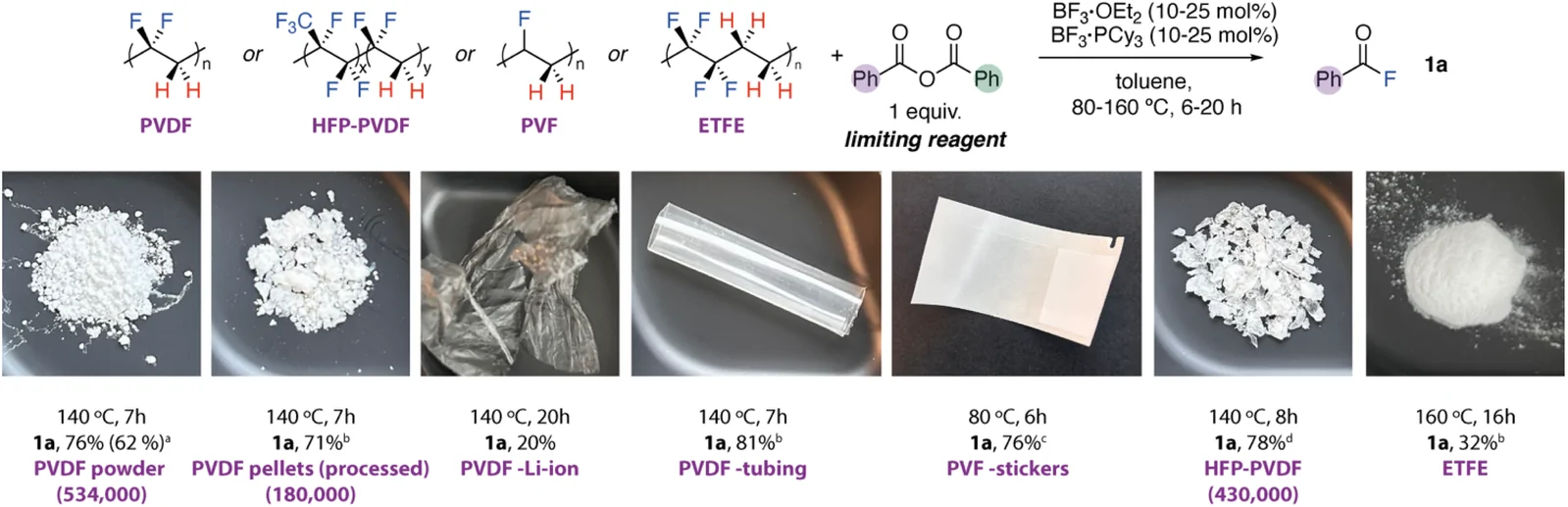
Application of catalytic HF shuttling to PVDF and related fluoropolymers. ^a^2 equiv. of repeat unit of polymer, 25 mol% BF_3_·PCy_3_, and 25 mol% BF_3_·OEt_2_; yield monitored by ^19^F NMR spectroscopy against fluorobenzene as an internal standard. Isolated yield in parentheses. ^b^Iso-propylbenzene used as a solvent. ^c^10 mol% BF_3_·PCy_3_ and 10 mol% BF_3_·OEt_2_. ^d^1.9 equiv. of repeat unit of polymer.

The new protocol for HF shuttling was applied to PVDF and a series of related fluoropolymers (Fig. 6). Reactions were conducted with PVDF powder (*M*_w_ = 534 000), PVDF pellets (*M*_w_ = 180 000), poly(vinylidene difluoride)-*co*-(hexafluoropropylene) copolymer (*M*_w_ = 430 000), and ethylene tetrafluoroethylene copolymer (ETFE). A series of post-consumer materials including PVDF tubing (Pro Powder SHF175-6.4MM-1.2M), PVDF recovered from a Li-ion battery, and PVF stickers (Brady Tedlar M71-21-634/BMP71) were also investigated. In all cases, benzoyl fluoride (1a) was generated in modest to excellent yields of 20–81%. The current protocol appears to work well for PVDF and PVF but is less efficient for ETFE. This may be due to the microstructure of the fluoropolymer, with the repeat unit of PVDF promoting a chain-reaction in which one dehydrofluorination event promotes reaction of the adjacent site.

**Fig. 7:**
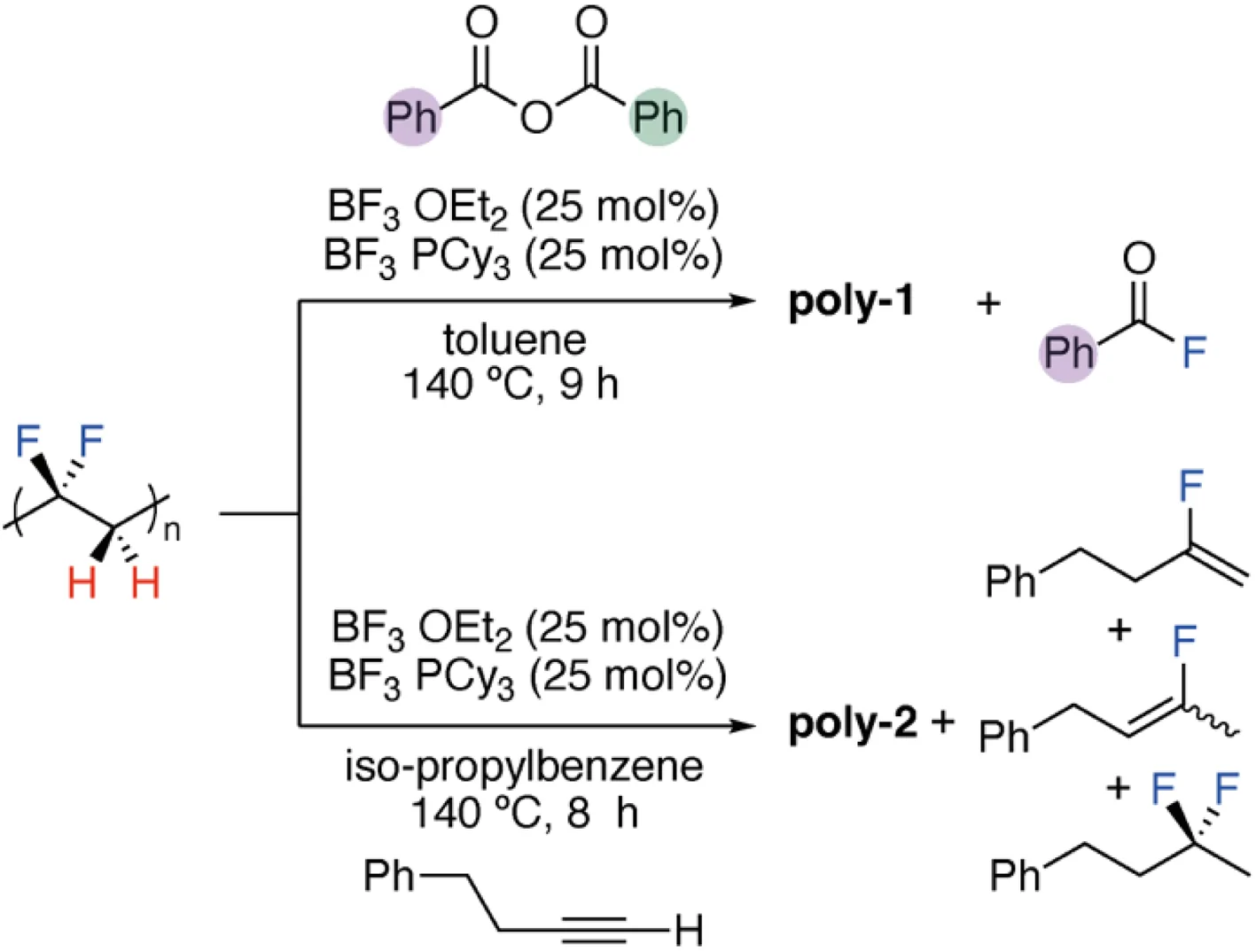
Isolation of polymeric side products from HF shuttle catalysis with benzoic anhydride (poly-1) and 4-phenyl-1-butyne (poly-2).

As these reactions proceed, PVDF (*M*_w_ = 534 000), which is never entirely dissolved in solution, changes appearance from colourless to a grey/black solid. Following the reaction, the recovered polymeric side-product poly-1 is more brittle and less soluble than PVDF itself (Fig. 7). Attempts to extract further equivalents of fluorine from the polymer through re-exposing of poly-1 to the catalytic conditions met with limited success. Repeated reaction cycles of PVDF (*M*_w_ = 534 000) gave 70%, 9% and 4% yields of 1a, suggesting that nearly all the accessible fluoride content of the polymer is obtained in the first catalytic run. Further fluorine content could be extracted from the sample, however, using a recently developed transfer fluorination method developed in our labs.^29,30^ Treatment of poly-1 with KO^t^Bu (0.5 equiv. as limiting reagent) in THF for 24 h followed by extraction into H_2_O and quantification by ^19^F NMR spectroscopy showed formation of KF in 90% yield.

To gain further insight, the materials recovered from HF shuttling catalysis were characterised by standard solid-state methods. As a point of comparison, the polymeric side-product poly-2 was also isolated from the HF shuttling reaction using 1-dodecyne as an HF acceptor and characterised alongside poly-1 (Fig. 7). Notably the data that follow suggest that poly-1 and poly-2 differ in structure. Consistent with the model studies with fluoroethane (*vide supra*), reactions of PVDF with benzoic anhydride catalysed by mixtures of BF_3_·PCy_3_ (10 mol%) and BF_3_·OEt_2_ (10 mol%) led to a polymeric side-product in which oxygen-containing functional groups are incorporated into the polymer chain. The implication is that the mechanistic model developed for fluoroethane is directly applicable to PVDF, and that benzoate is incorporated into the modified polymer backbone due to a combination of competitive dehydrofluorination–hydrofluorination and fluoroalkylation pathways.

**Fig. 8:**
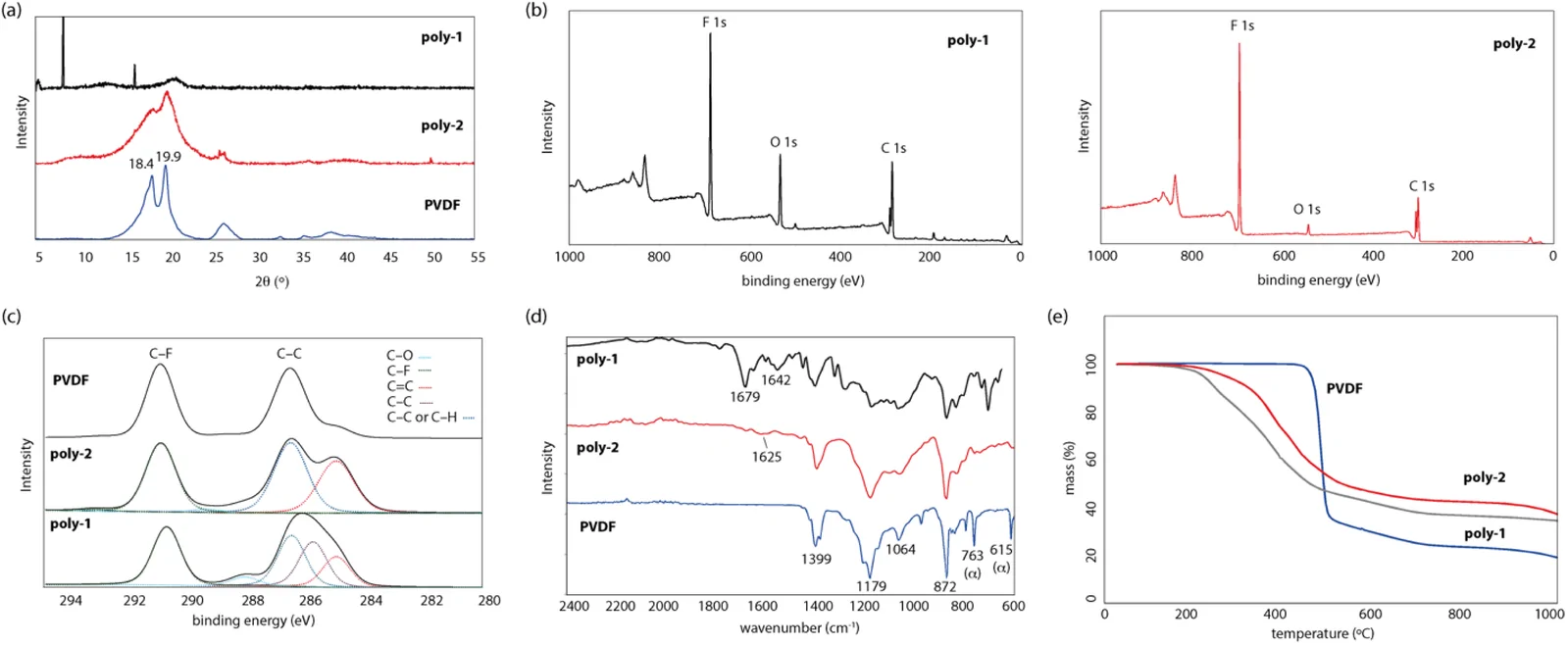
(a) Powder XRD data. (b) Comparison of XPS survey data for poly-1 and poly-2. (c) C 1s XPD data for PVDF, poly-1, and poly-2 along with fits for the latter two polymers. (d) Comparison of selected region of the infrared spectra of PVDF, poly-1 and poly-2. (e) TGA data comparing thermal decomposition profiles of PVDF, poly-1 and poly-2.

Powder X-ray diffraction (XRD) data show a loss of crystallinity of the recovered polymers following HF shuttling (Fig. 8a). Pristine PVDF (*M*_w_ = 534 000) shows diagnostic reflections at 18.4 and 19.9° and is believed to exist primarily as the *α*-phase. Prior studies have shown that dehydrofluorination of PVDF occurs with a phase transition to the *β*-phase of the fluoropolymer.^46,47^ The *β*-phase of PVDF is of particular interest due to its conductive and piezoelectric properties.^47^ The changes in relative intensity of the reflections in poly-2 compared to PVDF can be attributed to an increase in the *β*-phase in the recovered polymer. Signal-to-noise ratios of powder XRD of poly-1 and poly-2 are poorer than that of PVDF. While both lack crystallinity, poly-1 from reactions involving benzoic anhydride is almost completely amorphous. These bulk changes were also reflected in differential scanning calorimetry (DSC) data. PVDF displays a well-defined melting transition *T*_m_ as an endothermic peak between 153 and 165 °C. There is no clear *T*_g_ – as PVDF is a semi-crystalline polymer, the glass transition represents a relatively small endothermic energy change, which is outside of the limits of detection of the instrument used. However, literature reports place the *T*_g_ of PVDF at approximately −40 °C.^48^Poly-2 does not show a melting transition consistent with the lack of crystallinity. A small glass transition occurs at 19 °C. Poly-1 shows a complex peak at 77 °C.

The chemical changes that underpin the materials properties were probed further. X-ray photoelectron spectroscopy (XPS) revealed that samples of poly-1 contained a significantly higher quantity of oxygen than poly-2 and PVDF (*M*_w_ = 534 000) as evidenced by the intensity of the O 1s transitions (Fig. 8b). Comparison of the C 1s peaks revealed that pristine PVDF shows well-defined peaks at 286.6 eV and 291.1 eV assigned to the C–C and CF_2_ environments, respectively. Integration shows that these are in a near 1 : 1 ratio consistent with the expected [CH_2_CF_2_] repeat unit of the fluoropolymer. On dehydrofluorination to form poly-2 the spectrum shows a clear increase in peaks associated with non-fluorinated carbon environments with the appearance of a transition at 285.0 eV assigned as a C=C environment. Similar observations have been made before during the treatment of PVDF with basic reagents expected to promote dehydrofluorination.^25,46,49–51^ Peak fitting suggests that the ratio of CF_2_ to C–C and C=C environments is 1 : 2, consistent with, on average, removal of one fluorine atom per repeat unit of the polymer on dehydrofluorination. While XPS is a surface sensitive technique that is not necessarily representative of the bulk sample, CHN analysis on poly-2 returns C, 61.4% and H, 5.2% and is consistent with significant loss of fluorine per repeat unit (C_2_H_2_F_2_ = C, 37.5%, H, 2.7%; C_2_HF = C, 54.6%, H, 2.3%; C_2_ = C, 100%, H, 0%). The XPS data for poly-1 are more complex but can be fitted to a combination of C–F, C–O, C–C and C=C environments, particularly clear is the transition at 288.1 eV assigned as a C–O environment (Fig. 8c).

Infrared spectroscopy of pristine PVDF (*M*_w_ = 534 000) is consistent with it existing primarily as the *α*-phase (Fig. 8d). Diagnostic absorptions associated with this phase at 763 and 615 cm^−1^ are no longer apparent in poly-1 and poly-2 due to the loss of this phase following catalytic HF shuttling.^52^Poly-2 shows weak absorptions between 1620 and 1595 cm^−1^ assigned to conjugated C=C vibrations.^51,53–55^ These stretches disappear on hydroboration of the samples with BH_3_·PCy_3_. Similar absorptions are apparent in poly-1, accompanied by strong stretches at 1679 cm^−1^ and 1642 cm^−1^, assigned to C=O and C=C stretching modes, respectively. Prior work has suggested that C≡C bonds might form on dehydrofluorination of PVDF based on absorptions at 2100–2170 cm^−1^.^53,56^ No such stretches are obvious in poly-1 or poly-2. In combination with the XPS data, these findings suggest that poly-1 contains both unsaturated C=C bonds and oxygen-containing functional groups, due to a combination of dehydrofluorination–hydrofluorination and fluoroalkylation pathways occurring during catalysis.

The recovered fluoropolymers showed very different thermal decomposition profiles when compared to PVDF (*M*_w_ = 534 000) as evidence by thermogravimetric analysis (TGA) coupled with mass spectrometry (MS), carried out under a N_2_ atmosphere (Fig. 8e). Onset of mass loss from pristine PVDF occurs at around 450 °C with loss of 67.5% of mass accounting for elimination of 2 equiv. of HF from the polymer to form a material that then undergoes a slow mass loss at higher temperatures.^20,54^ MS analysis of the volatile materials confirmed a species with *m*/*z* = 20 likely to be HF. In contrast, poly-1 and poly-2 begin to show mass loss on heating to 200 °C with similar profiles. For both, more than 50% mass loss occurs before the onset temperature of decomposition of pristine PVDF. These data suggest that following recovery from the HF shuttling reaction, the polymer products may be easier to destroy than their pristine counterpart, PVDF.

## Conclusions

In summary, a catalytic approach to extract and recycle the fluorine content of certain fluoropolymers (PVDF, PVF, PVDF-HFP, and ETFE) to generate acyl fluorides from acid anhydrides through HF shuttling is reported. Acyl fluorides themselves are versatile fluorine carriers that are finding increased use in the preparation of fluorochemicals. A combination of BF_3_·OEt_2_ and BF_3_·PCy_3_ was found to be the most effective catalytic combination for HF shuttling. Through model studies using fluoroethane (HFC-161) as a substrate, a detailed understanding of catalyst speciation and reactivity was obtained. These studies suggest that at least two mechanisms are at play, the first involving the expected dehydrofluorination–hydrofluorination sequence, and the second involving fluoroalkylation of the acid anhydride to form an ester group. The new recycling approach was applied to both pristine fluoropolymers and post-consumer materials, including PVDF recovered from a Li-ion battery. Analysis of the polymeric materials recovered from catalytic reactions with PVDF suggests that dehydrofluorination occurs with the introduction of both C=C bonds and oxygen-containing functional groups (derived from the fluoroalkylation pathway with the acid anhydride) into the polymer chain. During thermogravimetric analysis, these materials show decomposition onset temperatures over 200 °C lower than pristine PVDF, suggesting that they might be easier to destroy following harvesting of their fluorine content by catalytic approaches.

## Author contributions

The manuscript was written through contributions of all authors. All authors have given approval to the final version of the manuscript.

## Conflicts of interest

The authors declare no competing interests.

## Supplementary Material

SC-017-D6SC02698B-s001

SC-017-D6SC02698B-s002

## Data Availability

Supplementary information (SI): synthetic procedures, NMR spectra of all compounds, all computation methods (PDF), and Cartesian coordinates of all the DFT-optimised structures (XYZ). See DOI: https://doi.org/10.1039/d6sc02698b.
