# Supplementary material for: Recycling fluoropolymers to acyl fluorides through shuttle catalysis
Source: Chem Sci. 2026 Jun 29;17(35):16781–8. doi: 10.1039/d6sc02698b (PMC13389741; doi:10.1039/d6sc02698b)
Supplement: Supplementary file 1 [file SC-017-D6SC02698B-s002.pdf]

Supplementary Information (SI) for Chemical Science.
This journal is © The Royal Society of Chemistry 2026

Recycling Fluoropolymers to Acyl Fluorides through Shuttle Catalysis

Supporting Information

Shannon E. S. Farley, Amanda A. Fogh and Mark R. Crimmin*

Department of Chemistry, Molecular Sciences Research Hub, Imperial College London, 82 Wood Lane, Shepherds Bush, London W12 0BZ, UK

**Corresponding author e-mail: m.crimmin@imperial.ac.uk*

1 Contents

1 Contents 2
2 Materials and Methods 4
3 Experimental procedures and analytical data 6
3.1 Preparation of acid anhydrides 6
3.2 Synthesis of $BF_3 \cdot PCy_3$ 10
3.3 Reaction discovery and polymer donor optimisation 11
3.3.1 Stoichiometry of PVDF 11
3.3.2 Reaction of PVDF with terminal alkynes. 11
3.3.3 Additive screen 11
3.3.4 Catalyst and cocatalyst loading optimisation 17
3.3.5 Polymer donor to alkyne acceptor stoichiometry optimisation 18
3.3.6 Acceptor scope 19
3.3.7 Solvent screen for reaction of PVDF with benzoic anhydride 21
3.3.8 Concentration optimisation for the reaction of PVDF with benzoic anhydride 21
3.4 Shuttle catalysis of fluoroethane 22
3.4.1 Temperature optimisation 22
3.4.2 Variation of the ratio of fluoroethane and acid anhydride reagents 22
3.4.3 Solvent screen 23
3.4.4 Catalyst and cocatalyst loading optimisation 24
3.5 Shuttle catalysis reactions between fluoroethane and acid anhydrides 26
3.6 Mechanistic investigation 31
3.6.1 Control Reactions 31
3.6.2 Role of $BF_3 \cdot PCy_3$ 33
3.6.3 Hydrofluorination of acid anhydrides 36
3.6.3 Mechanism of ester formation 37
3.7 Shuttle catalysis reactions between fluoropolymers and benzoic anhydride 42
3.7.1 Scope in fluoropolymer donors 42
3.7.2 Successive defluorination trials 48
3.7.3 Further fluorine recycling of *poly-1* and *poly-2* by fluorine mineralisation 49
3.7.4 Further fluorine recycling of *poly-1* and *poly-2* by fluorine transfer 51
3.7.5 Shuttle catalysis reactions between fluoropolymers and alkynes 53
4 NMR spectra 55
5 Solid state characterisation of polymers 82
5.1 Infrared spectroscopy 82

5.2 X-ray photoelectron spectroscopy 88
5.3 Powder X-ray diffraction spectroscopy 94
5.4 Differential scanning calorimetry 95
5.5 Thermogravimetric-mass spectrum analysis 97
5.6 Elemental analysis 101
6 Computational details 102
6.1 Methods 102
6.2 Reaction pathway for the boron trifluoride diethyl etherate and boron trifluoride tricyclohexylphosphine catalysed reaction of fluoroethane and benzoic anhydride 107
6.2.1 Description of possible pathways 107
6.2.2 Key geometrical parameters of intermediates 115
6.2.3 Key geometrical parameters of transition states 130
6.2.4 Intrinsic reaction coordinate plots 143
6.2.5 General pathway 153
6.2.6 NBO analysis: Wiberg Bond Indices (WBI) and NPA charges 162
7 XYZ coordinates 173
8 References 214

2 Materials and Methods

Reactions were carried out using standard Schlenk-line and glovebox techniques. All reagents were manipulated in a glovebox unless otherwise stated. Reactions were carried out in J. Young's tap NMR tubes, ampoules or Schlenk flasks. Glassware was dried at 120 °C for 12 hours prior to use. All solvents and liquid reagents (with the exception of $BF_3{\cdot}OEt_2$) were dried over 4 Å molecular sieves and freeze-pump-thaw degassed prior to use. Reactions were heated using sand baths or silicone oil baths. ^1H and ^{19}F NMR spectra were obtained on Bruker 400 MHz or 500 MHz machines and were referenced against $SiMe_4$ (^1H or ^{13}C) or fluorobenzene (^{19}F). NMR data were processed using the MestReNova software package. Multiplicity assignments in NMR spectra are labelled as follows: "s" = singlet, "d"= doublet, "t"= triplet, "q"= quartet, "m" = multiplet.

Infrared spectra were obtained on a Cary630 spectrometer. Powder X-ray diffraction was obtained on a Brucker D2 Phaser XE-T diffractometer. TGA analysis was performed on a Perkin Elmer TGA8000. Analyses were performed under nitrogen and samples were loaded onto aluminium pans for analysis. A heating rate of 10 °C min^{-1} was applied for all samples. Elemental analysis was performed by London Metropolitan University (https://www.londonmet.ac.uk/). Differential scanning calorimetry was performed by University of St Andrews (https://www.st-andrews.ac.uk/).

X-ray photoelectron (XPS) data was acquired at the EPSRC National Facility for XPS. X-ray photoelectron spectroscopy (XPS) was performed on a Thermo Fisher Scientific K-alpha+ spectrometer. Samples were analysed using a micro-focused monochromatic An X-ray source (72 W) using the "200-micron spot" mode, which provides an analysis defining elliptical X-ray spot of *ca*. 200 x 300 microns. Data was recorded at pass energies of 150 eV for survey scans and 50 eV for high resolution scans with step sizes of 1 eV and 0.1 eV respectively, the dwell time was 50 ms and 10 ms in each case. Samples were pressed on to doubled sided adhesive tape (3M Scotch 665) using a UV-ozone cleaned glass microscope slide and mounted in the spectrometer. Charge compensation was achieved using a combination of low energy electrons and argon ions. A minimum of 3 points were analysed per sample.

As samples are known to degrade under x-ray analysis, samples were acquired with a minimal number of sweeps (ca 4 per element).[1] The total analysis time was *ca.* 3 minutes per area. Data analysis was performed in CasaXPS v2.3.26 after calibrating the data to the lowest C(1s) component taken to have a value of 285 eV.[2] Quantification was made using a 2-Parameter Tougaard type background and Scofield cross sections, with an electron energy dependence based on the TPP-2M relationship.[3]

PVF was obtained as Brady Tedlar (M71-21-634/BMP71) labels, and PVDF tubing was obtained from Pro Powder (SHF175-6.4MM-1.2M). PVDF from Li-ion batteries was donated by the ReLiB project. Poly(vinylidene difluoride) was purchased from Sigma Aldrich or Fluorochem (average M_w= 534,000 or 180,000 by GPC as powder and pellets respectively). Poly(vinylidene difluoride)-co-(hexafluoropropylene) was purchased from Sigma-Aldrich (M_w= 400,000 by GPC) as pellets. PVDF and PVDF-co-HFP pellets were processed by dissolution in acetonitrile at 60 °C and concentration *in vacuo* – the resulting solids were ground to smaller pellets with a pestle and mortar before use. PVF stickers were soaked in acetone for 24 hours then dried *in vacuo* prior to use to remove the adhesive glue. Ethylene tetrafluoroethylene (ETFE) (Fluon ETFE Z-8820X) was donated by AGC Chemicals. All other reagents were acquired from Sigma-Aldrich, Fluorochem or Alfa Aesar and used without any further purification unless specified. Purifications were carried out by column chromatography on silica gel (tech grades, 60 Å, 230-400 mesh, 40-63 particle size).

3 Experimental procedures and analytical data

3.1 Preparation of acid anhydrides

General procedure for the synthesis of acid anhydrides.

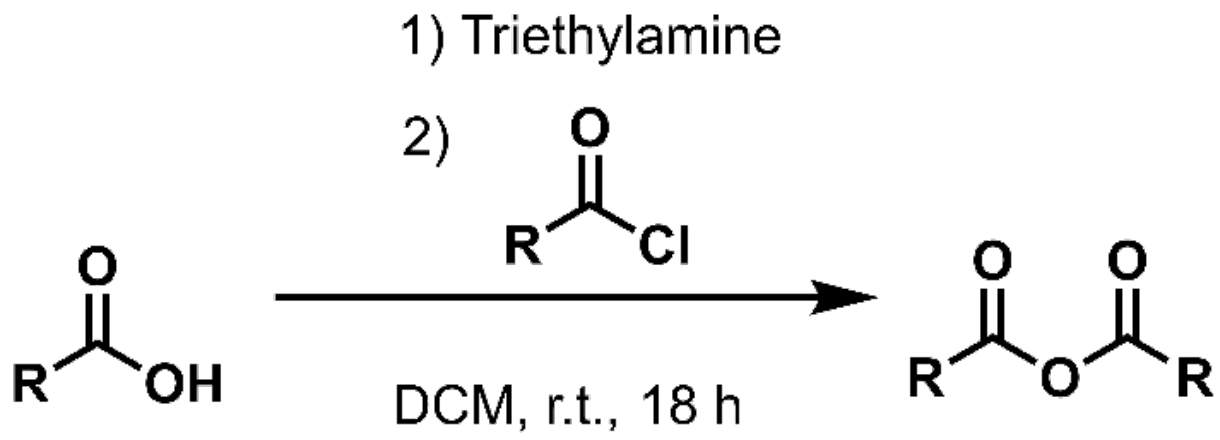

According to a modified literature procedure,[4] under an inert atmosphere to a solution of benzoic acid (6.00 mmol) and triethylamine (12.00 mmol) in DCM (80 mL) was added a solution of acyl chloride (6 mmol) in DCM (20 mL) drop wise at room temperature. The resulting mixture was stirred at room temperature for 18 hours before being washed with $NaHCO_3$ (aq)$_{SAT}$ (3 x 20 mL) and brine (3 x 20 mL). The combined organic layers were dried over $MgSO_4$, filtered and concentrated *in vacuo*. Unless otherwise stated, the resulting crude products were purified by column chromatography using silica gel in n-hexane and ethyl acetate (4:1). Yields and characterisation data given below.

2,4,6-trimethylbenzoic anhydride

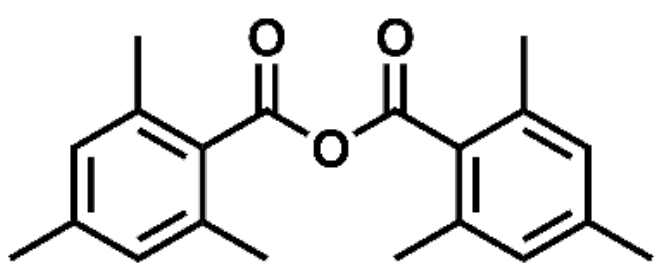

Isolated as a white powder (1.17 g, 3.78 mmol, **63 %**). The spectroscopic data matched those reported in the literature.[4]

^{1}H NMR (400 MHz, $CDCl_3$) δ 6.88 (s, 4H, $C_{Ph}H$), 2.40 (s, 12H, CH_3), 2.29 (s, 6H, CH_3). **^{13}C{^{1}H} NMR** (101 MHz, $CDCl_3$) δ 165.4 (s, COO), 140.8 (C_{Ph}), 136.3 (C_{Ph}), 134.9 (C_{Ph}), 128.9 ($C_{Ph}H$), 21.2 (s, CH_3), 20.1 (s, CH_3).

4-bromobenzoic anhydride

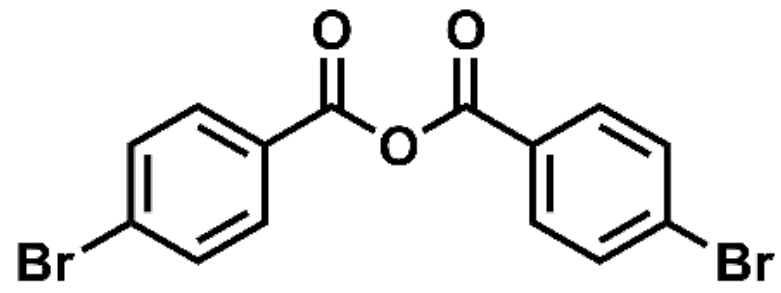

Isolated as a white powder (2.21 g, 5.52 mmol, **92 %**). The spectroscopic data matched those reported in the literature.[5]

^{1}H NMR (400 MHz, $CDCl_3$): δ 8.02 (d, $^3J_{H\text{-}H}$ = 8.5 Hz, 4H, $C_{Ph}H$), 7.71 (d, $^3J_{H\text{-}H}$ = 8.5 Hz, 4H, $C_{Ph}H$). **^{13}C{^{1}H} NMR** (101 MHz, $CDCl_3$): δ 161.6 (s, COO), 132.5 (s, $C_{Ph}H$), 132.1 (s, $C_{Ph}H$), 130.4 (s, $C_{Ph}Br$), 127.7 (s, C_{Ph}).

2,6-difluorobenzoic anhydride

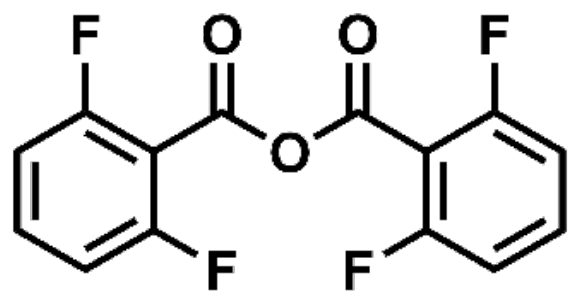

Isolated as a crystalline white solid (1.41 g, 4.86 mmol, **61 %**).

^{1}H NMR (400 MHz, $CDCl_3$) δ 7.63 – 7.51 (m, 4H, $C_{Ph}H$), 7.05 (t, $^3J_{H–F}$ = 9.1, 2H, $C_{Ph}H$). **^{13}C NMR** (101 MHz, $CDCl_3$) δ 161.20 (d, $^1J_{C–F}$ = 260.2 Hz, $C_{Ph}F$), 155.8 (s, COO), 135.2 (dd, $^2J_{C–F}$ = 21.8 Hz, $^4J_{C–F}$ = 9.3 Hz, $C_{Ph}H$), 112.7 (t, $^3J_{C–F}$ = 3.8 Hz, $C_{Ph}H$), 108.95 (t, $^2J_{C–F}$ = 14.9 Hz, C_{Ph}). **^{19}F NMR** (377 MHz, $CDCl_3$) δ -107.6 (m, 4F, $C_{Ph}F$). MS (EI): m/z calculated for $(C_{14}H_6F_4O_3)^+$: 298.0248; found: 298.0241.

4-trifluoromethylbenzoic anhydride

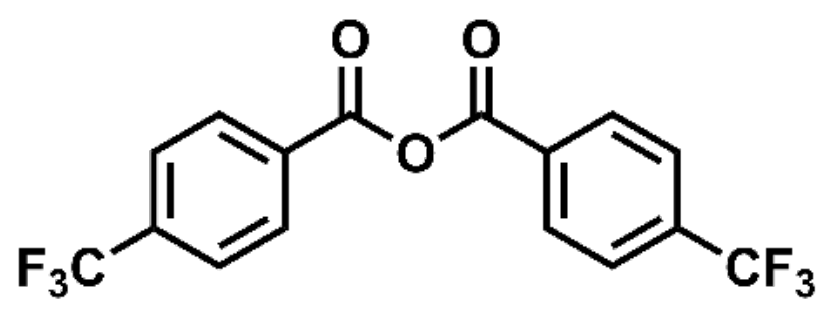

Isolated as a white solid (1.91 g, 5.28 mmol, **88 %**). The spectroscopic data matched those reported in the literature.[4]

^{1}H NMR (400 MHz, $CDCl_3$) δ 8.31 (d, $^3J_{H-H}$ = 8.1 Hz, 4H, $C_{Ph}H$), 7.85 (d, $^3J_{H-H}$ = 8.2 Hz, 4H, $C_{Ph}H$). **^{13}C{^{1}H} NMR** (101 MHz, $CDCl_3$) δ 160.8 (s, COO), 136.0 (q, $^2J_{C-F}$ = 33.0 Hz, **C**$_{Ph}$(CF_3)), 131.7 (s, C_{Ph}), 131.0 (s, C_{Ph}), 126.1 (q, $^3J_{C-F}$ = 3.7 Hz, $C_{Ph}H$), 123.3 (q, $^1J_{C-F}$ = 272.9 Hz, CF_3). **^{19}F NMR** (377 MHz, $CDCl_3$) δ -63.3 (s, 6F, CF_3).

4-methoxybenzoic anhydride

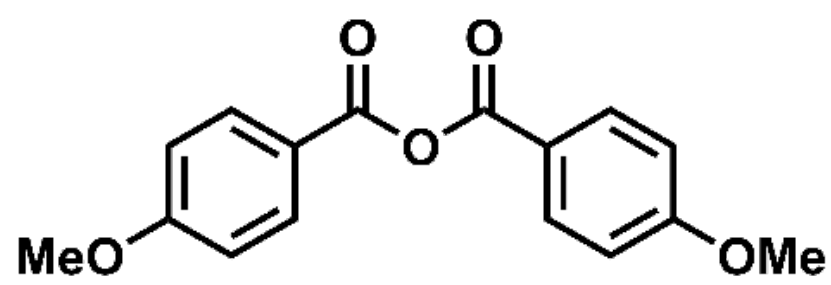

Isolated as a white solid (1.17 g, 4.08 mmol, **68 %**). The spectroscopic data matched those reported in the literature.[4]

^{1}H NMR (400 MHz, $CDCl_3$) δ 8.40 – 7.92 (d, $^3J_{H-H}$ = 8.8 Hz, 4H, $C_{Ph}H$), 7.01 (d, $^3J_{H-H}$ = 8.8 Hz, 4H, $C_{Ph}H$), 3.92 (s, 6H, OCH_3). **^{13}C{^{1}H} NMR** (101 MHz, $CDCl_3$) δ 164.7 (s, COO), 162.4 (s, C_{Ph}), 133.0 (s, C_{Ph}), 121.4 (s, C_{Ph}), 114.3 (s, C_{Ph}), 55.7 (s, OCH_3).

2-naphthoic anhydride

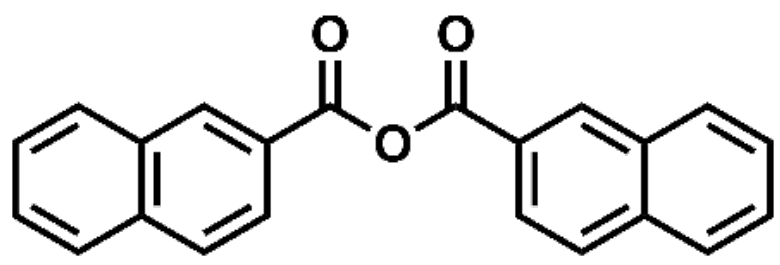

Isolated as a crystalline white solid (1.88 g, 5.76 mmol, **96 %**). The spectroscopic data matched those reported in the literature.[5]

^{1}H NMR (400 MHz, $CDCl_3$) δ 8.79 (s, 2H, $C_{Ph}H$), 8.20 (d, $^3J_{H-H}$ = 8.7 Hz, 2H, $C_{Ph}H$), 8.08 – 7.87 (m, 6H, $C_{Ph}H$), 7.67 (m, 2H, $C_{Ph}H$), 7.62 (m , 2H, $C_{Ph}H$). **^{13}C{^{1}H} NMR** (101 MHz, $CDCl_3$) δ 162.9 (s, **C**OO), 136.4 (s, C_{Ph}), 133.0 (s, C_{Ph}), 132.6 (s, $C_{Ph}H$), 129.8 (s, $C_{Ph}H$), 129.4 (s, $C_{Ph}H$), 129.0 (s, $C_{Ph}H$), 128.1 (s, $C_{Ph}H$), 127.3 (s, $C_{Ph}H$), 126.3 (s, $C_{Ph}H$), 125.6 (s, $C_{Ph}H$).

3.2 Synthesis of $BF_3{\cdot}PCy_3$

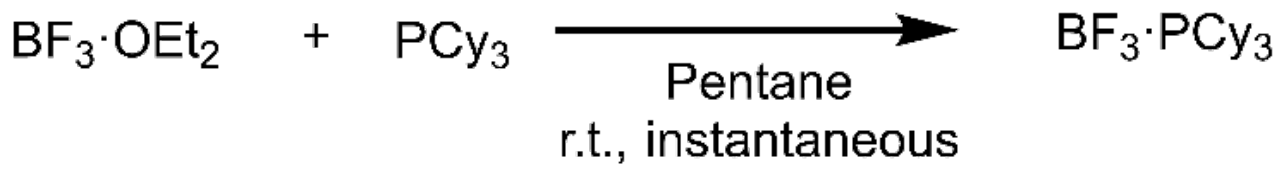

Under an inert atmosphere, $BF_3{\cdot}OEt_2$ (247 µL, 2.00 mmol, 2 equiv.) was added to a solution of PCy_3 (0.56 g, 2.00 mmol, 1 equiv.) in n-pentane (100 mL), causing a white solid to crash out of solution. The resulting solid was isolated by cannular filtration and dried *in vacuo* to yield $BF_3{\cdot}PCy_3$ as a white powder (0.68 g, 1.95 mmol, 98%).

^{1}H NMR (400 MHz, C_6D_6) δ 1.95 – 1.87 (m, 9H, CH), 1.59 – 1.57 (m, 6H, CH), 1.48 – 1.42 (m, 9H, CH), 1.06 – 0.98 (m, 9H, CH). **^{13}C{^{1}H} NMR** (101 MHz, C_6D_6) δ 29.2 (d, $^{1}J_{C\text{–}P}$ = 24.9 Hz, P**C**(CH_2)), 28.1 (s, CH), 27.3 (d, $^{2}J_{C\text{–}P}$ = 10.8 Hz, CH), 26.1 (s, CH). **^{19}F NMR** (377 MHz, C_6D_6) δ -122.71 (dq, $^{2}J_{P\text{–}F}$ = 203.5, $^{1}J_{B\text{–}F}$ = 52.5 Hz) **^{31}P{^{1}H} NMR** (162 MHz, C_6D_6) δ -3.54 (qq, $^{2}J_{P\text{–}F}$ = 203.5, $^{1}J_{B\text{–}P}$ = 156.5 Hz)**.** **^{11}B NMR** (128 MHz, C_6D_6) δ 2.04 (dq, $^{1}J_{B\text{–}P}$ = 156.5, $^{1}J_{B\text{–}F}$ = 52.5 Hz).

3.3 Reaction discovery and polymer donor optimisation

3.3.1 Stoichiometry of PVDF

The average molecular weight of the PVDF employed in reaction optimisation was 534,000 g mol $^{-1}$. Each PVDF repeat unit ($C_2F_2H_2$, molecular weight = 64.01 g mol $^{-1}$). Stoichiometry calculations were performed based on the mass of the repeat unit of PVDF – we can therefore consider an effective molar ratio of 2 mmol of PVDF to 1 mmol of acceptor. Similar estimations have been used on work in poly(vinyl chloride) and poly(tetrafluoroethylene).[6,7]

3.3.2 Reaction of PVDF with terminal alkynes.

We previously reported the reaction of PVDF with 4-phenyl-1-butyne using a stoichiometric volume of $BF_3{\cdot}OEt_2$ at 180 °C in iso-propyl benzene, giving rise to a mixture of fluorocarbon products.[8] In an effort to achieve product selectivity, we initially attempted using milder reaction conditions (**Scheme S.3.1**), with a sub-stoichiometric loading of $BF_3{\cdot}OEt_2$ and lower reaction times – however this resulted in significantly elongated reaction times and a reduction in overall yield, without a corresponding improvement in selectivity.

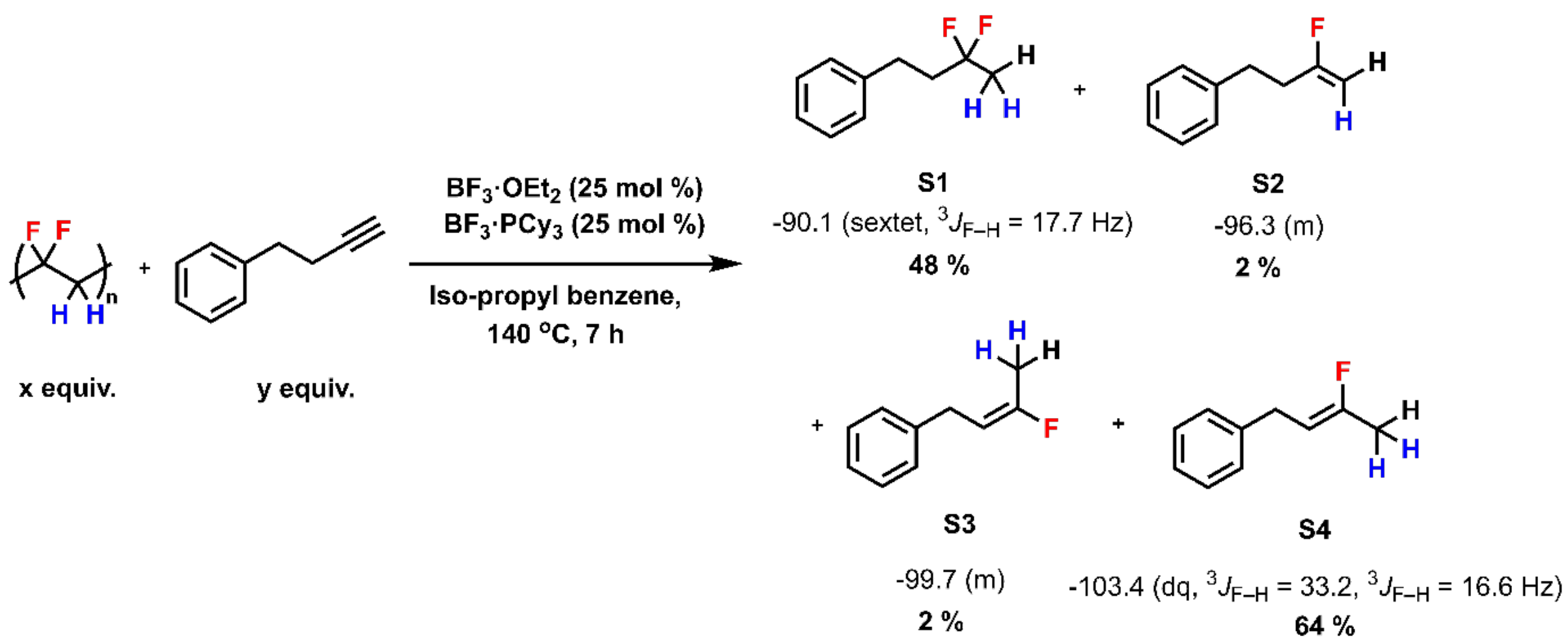

Scheme S.3.1 Reaction of PVDF (0.384 mmol, 2 equiv.) with 4-phenyl-1-butyne (0.192 mmol, 1 equiv.) catalysed by $BF_3{\cdot}OEt_2$ (0.096 mmol, 50 mol%) in iso-propyl benzene (0.2 mL). Yields monitored by quantitative ^{19}F NMR spectroscopy against a fluorobenzene internal standard. **3.3.3 Additive screen**

A scope of Lewis base additives was screened, to see if improved reactivity could be observed by promoting the defluorination of the PVDF starting material compared to previously reported results catalysed by $BF_3{\cdot}OEt_2$ (**Table S.3.1**), **Scheme S.3.1**.[8]

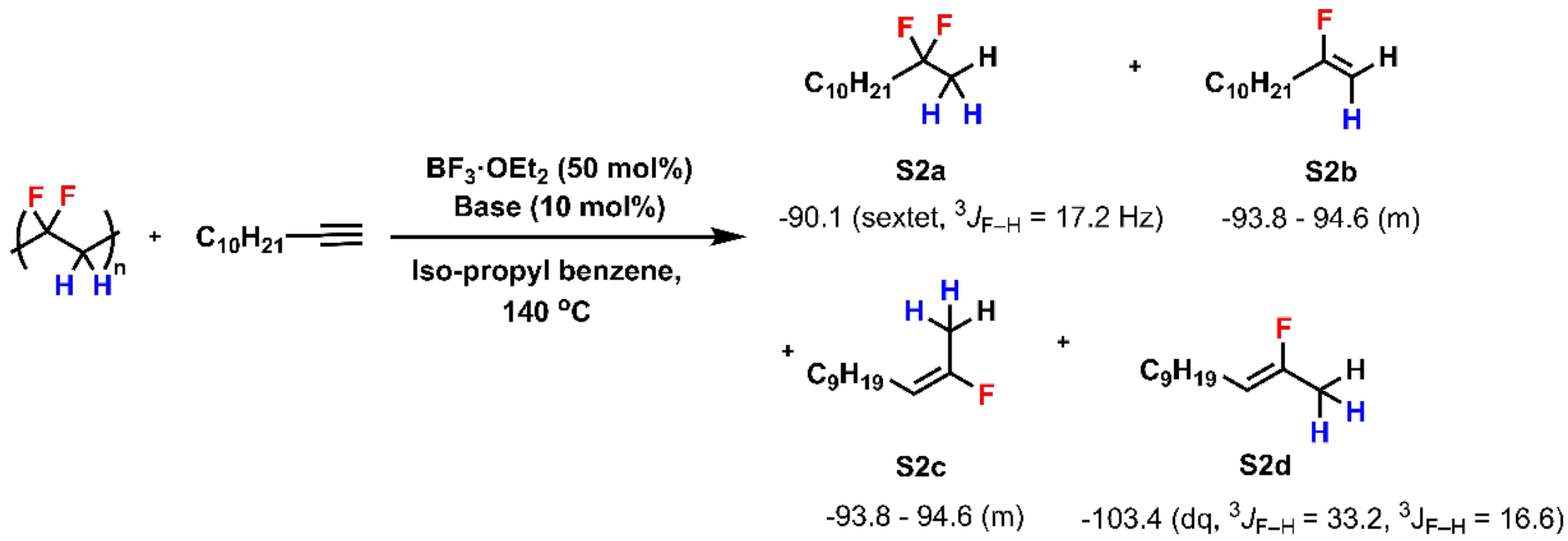

Entry n°.	Additive	Time (hours)	Yield **S2a** (%)	Yield **S2b** (%)	Yield **S2c** (%)	Yield **S2d** (%)	Total yield (**S2a** + **S2b** + **S2c** + **S2d**)
1	None	21	7	10	14	32	**63**
2	Et_3N	21	0	0	0	0	**0**
3	DBU	21	0	0	0	0	**0**
4	2,6-dichloropyridine	21	10	4	12	34	**60**
5	XantPhos	20	12	10	8	28	**58**
6	JohnPhos	19	30	4	8	24	**66**
7	PCy_3	18	9	6	14	38	**67**
8	PMe_3	21	6	2	4	10	**18**

Table S.3.1 Lewis base additive scope for the HF transfer reaction of poly(vinylidene difluoride) (0.38 mmol, 2 equiv.) with 1-dodecyne (0.19 mmol, 1 equiv.), yielding a mixture of fluorinated alkenes and alkanes in the presence of $BF_3{\cdot}OEt_2$ (0.096 mmol, 0.5 equiv.) and different bases (0.019 mmol). Yields monitored by quantitative ^{19}F NMR spectroscopy using a fluorobenzene internal standard. (DBU = 1,8-diazabicyclo[5.4.0]undec-7-ene) (Xantphos = 4,5-bis(diphenylphosphino)-9,9-dimethylxanthene) (JohnPhos = (2-biphenyl)tert-butylphosphine) (PCy_3 = tricyclohexylphosphine)

With the addition of 10 mol% tricyclohexylphosphine (PCy_3) improved yields were observed with a reduced reaction time, although comparable improvements in reaction time were observed with both 4,5-bis(diphenylphosphino)-9,9-dimethylxanthene (Xantphos) and (2-biphenyl)tert-butylphosphine (JohnPhos). No production of fluorinated product or change to PVDF was observed with the addition of triethylamine (Et_3N) or 1,8-diazabicyclo[5.4.0]undec-7-ene (DBU); instead, these formed adducts with $BF_3{\cdot}OEt_2$, hampering reactivity.

To build on this improved reactivity, several tests were performed with increasing loading of PCy_3 (**Table S.3.2**). Similar or improved yields were improved with PCy_3 loading up to 30 mol%, although beyond this, yields began to decrease suggesting a plateau or inhibition at higher loadings.

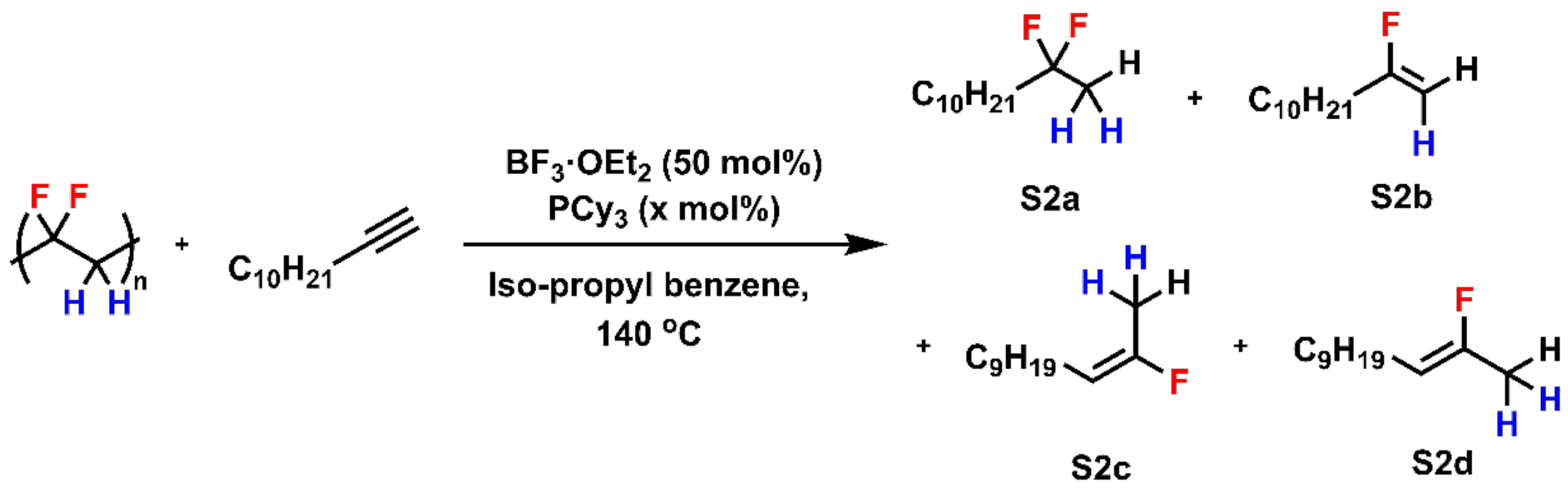

Entry n°.	PCy_3 (mol%)	$[BF_3]$: $[PCy_3]$	Time (hours)	Yield **S2a** (%)	Yield **S2b** (%)	Yield **S2c** (%)	Yield **S2d** (%)	Total yield (**S2a** + **S2b** + **S2c** + **S2d**)
1	None	1:0	21	7	10	14	32	**63**
2	10	1:0.2	18	9	6	14	38	**67**
3	20	1:0.4	14	22	6	14	36	**78**
4	30	1:0.6	9	30	2	10	32	**72**
5	40	1:0.8	9	1	8	4	8	**21**
6	50	1:1	9	2	8	2	8	**20**

Table S.3.2 PCy_3 loading optimisation for the shuttle catalysis reaction of PVDF (0.38 mmol, 2 equiv.) and 1-dodecyne (0.19 mmol, 1 equiv.) with $BF_3 \cdot OEt_2$ (0.096 mmol, 2 equiv.) generating a mixture of fluorinated alkene and alkane products. Yields monitored by quantitative ^{19}F NMR spectroscopy against a fluorobenzene internal standard.

The role of the PCy_3 additive was therefore probed – on addition to the reaction mixture, instantaneous reaction with $BF_3 \cdot OEt_2$ to generate boron trifluoride tricyclohexylphosphine ($BF_3 \cdot PCy_3$) was observed (**Figure S.3.1**). Small amounts of $[BF_4][HPCy_3]$ were also observed from reaction with $[BF_4][HOEt_2]$which exists in small quantities in equilibrium with $BF_3 \cdot OEt_2$ solutions from reaction with adventitious water.

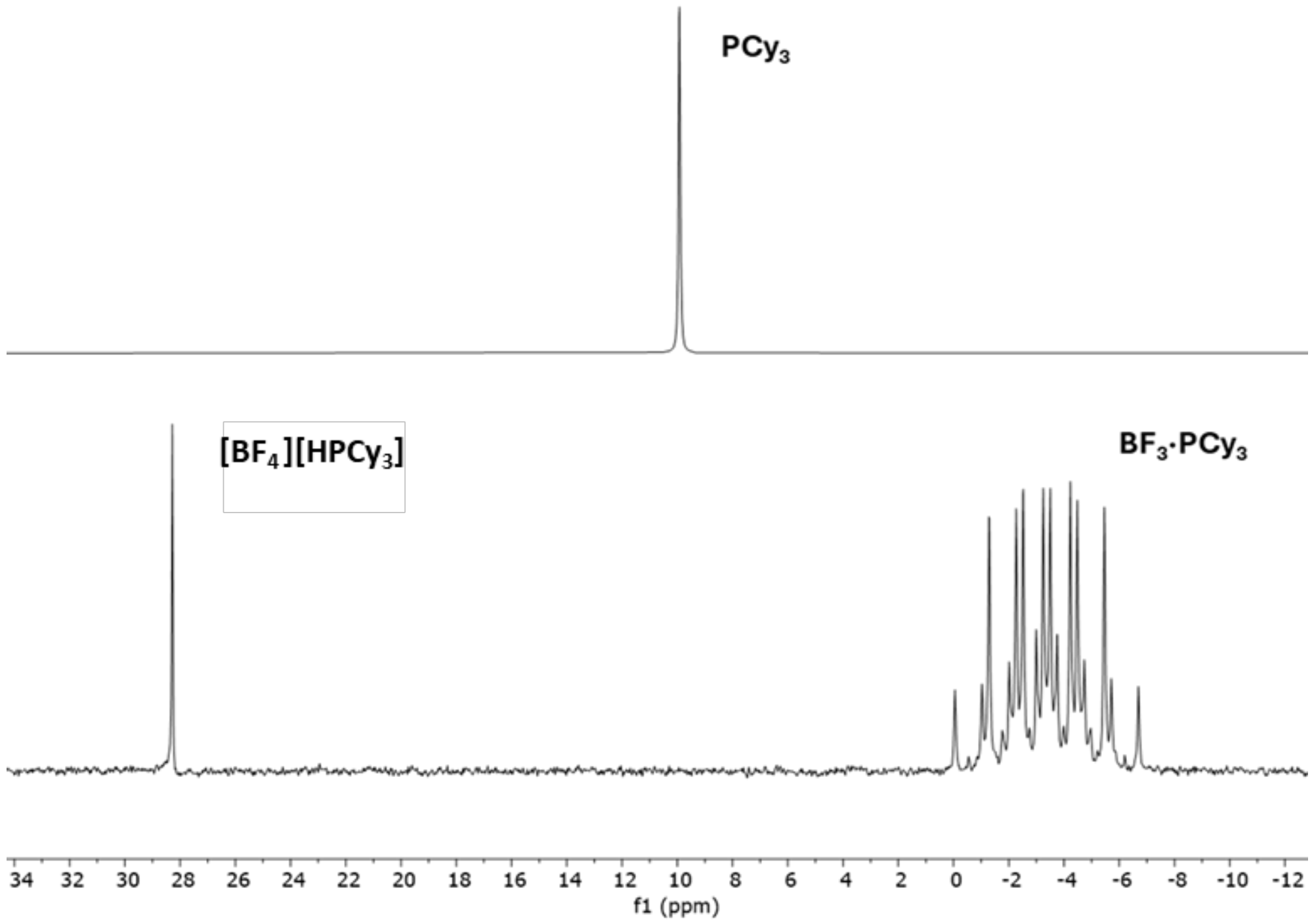

Figure S.3.1 Stack plot of $^{31}P\{^{1}H\}$ NMR spectra of PCy_3 starting material (top) and the reaction mixture at 0 hours prior to heating (bottom) showing the complete conversion of PCy_3 to $BF_3{\cdot}PCy_3$.

Following independent synthesis of $BF_3{\cdot}PCy_3$, its capacity to improve reactivity of the shuttle catalysis reaction as an additive was explored (**Table S.3.3**). Addition of $BF_3{\cdot}PCy_3$ resulted in greater improvements in yield than the addition of PCy_3, without displaying an inhibiting effect on addition of equivalent amounts to the $BF_3{\cdot}OEt_2$ catalyst. In the absence of $BF_3{\cdot}OEt_2$, no catalytic reactivity was observed with $BF_3{\cdot}PCy_3$. It is therefore expected that the $BF_3{\cdot}OEt_2$ remains the active catalyst in the process and the inhibiting effect on addition of greater than 30 mol% of PCy_3 was a result of complete consumption of $BF_3{\cdot}OEt_2$ to generate $BF_3{\cdot}PCy_3$, which is not catalytically active.

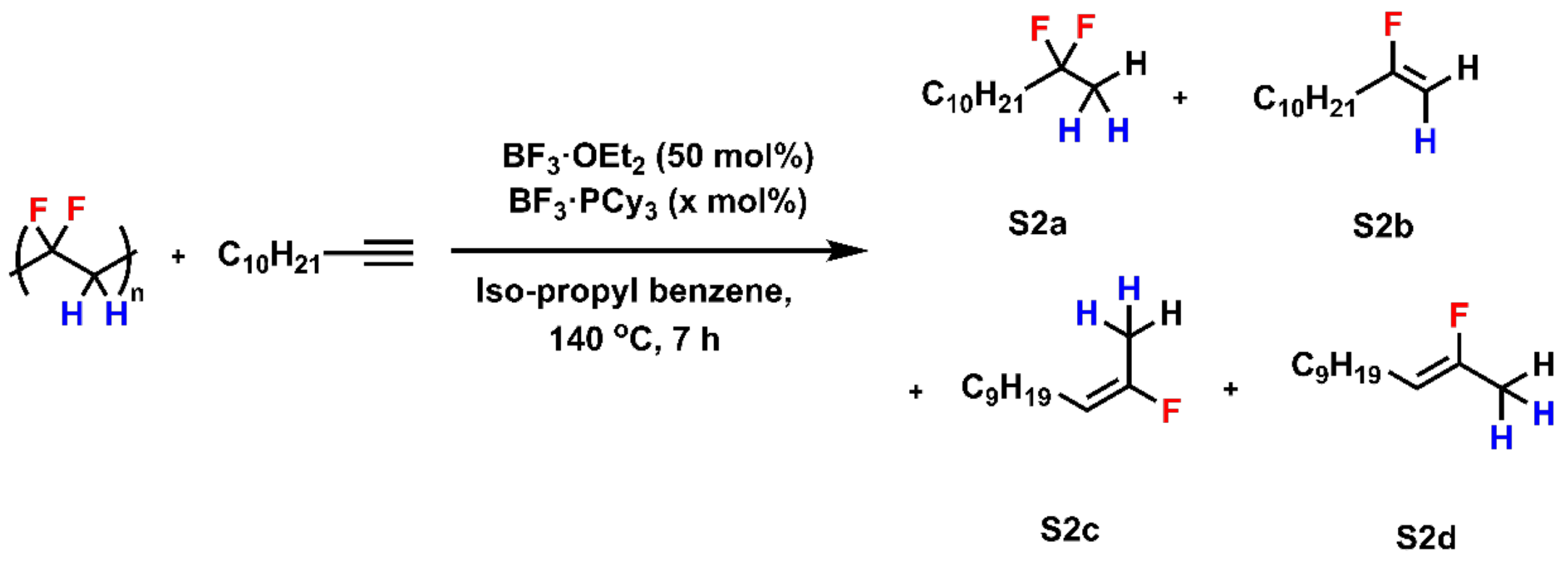

Entry n°.	$BF_3 \cdot PCy_3$ equiv. (x mol%)	$[BF_3] : [PCy_3]$	Time (hours)	Yield **S2a** (%)	Yield **S2b** (%)	Yield **S2c** (%)	Yield **S2d** (%)	Total yield (**S2a** + **S2b** + **S2c** + **S2d**) (%)
1	0	1 : 0	9	1	2	2	6	**11**
2	0.1	6 : 1	8	11	8	14	42	**73**
3	0.2	7 : 2	7	24	4	14	36	**78**
4	0.3	8 : 3	6	27	4	14	36	**81**
5	0.5	2 : 1	4	32	6	10	48	**96**

Table S.3.3 $BF_3 \cdot PCy_3$ cocatalyst loading optimisation for the reaction of PVDF (0.38 mmol, 2 equiv.) with 1-dodecyne (0.19 mmol, 1 equiv.) with in $BF_3 \cdot OEt_2$ (0.048 mmol, 0.5 equiv.) iso-propyl benzene (0.2 mL), generating a mixture of fluorinated alkene and alkane products. Yields monitored by quantitative ^{19}F NMR spectroscopy against a fluorobenzene internal standard.

3.3.4 Catalyst and cocatalyst loading optimisation

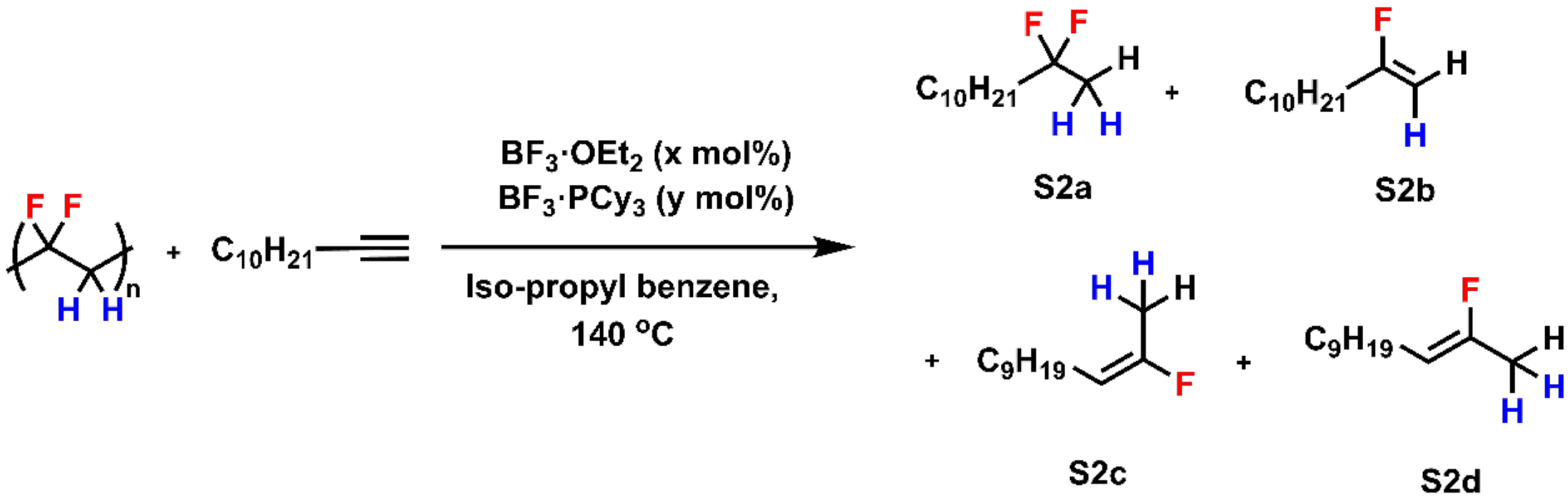

Entry n°.	$BF_3{\cdot}OEt_2$ equiv. (x mol%)	$BF_3{\cdot}PCy_3$ equiv. (y mol%)	[BF3]:[PCy3]	Time (hours)	Yield **S2a** (%)	Yield **S2b** (%)	Yield **S2c** (%)	Yield **S2d** (%)	Total yield (**S2a** + **S2b** + **S2c** + **S2d**) (%)
1	0.50	0.00	1 : 0.	9	1	2	2	6	**11**
2	0.50	0.10	6 : 1	8	35	2	6	24	**87**
3	0.50	0.20	7 : 2	7	24	4	14	36	**78**
4	0.50	0.30	8 : 3	6	27	4	14	36	**81**
5	0.25	0.25	2 : 1	7	35	4	12	36	**77**

Table S.3.4 Catalyst, $BF_3{\cdot}OEt_2$, and cocatalyst, $BF_3{\cdot}PCy_3$ loading optimisation for the reaction of PVDF (0.38 mmol, 2 equiv.) with 1-dodecyne (0.19 mmol, 1 equiv.) in iso-propyl benzene (0.2 mL), generating a mixture of fluorinated alkene and alkane products. Yields monitored by quantitative ^{19}F NMR spectroscopy against a fluorobenzene internal standard.

3.3.5 Polymer donor to alkyne acceptor stoichiometry optimisation

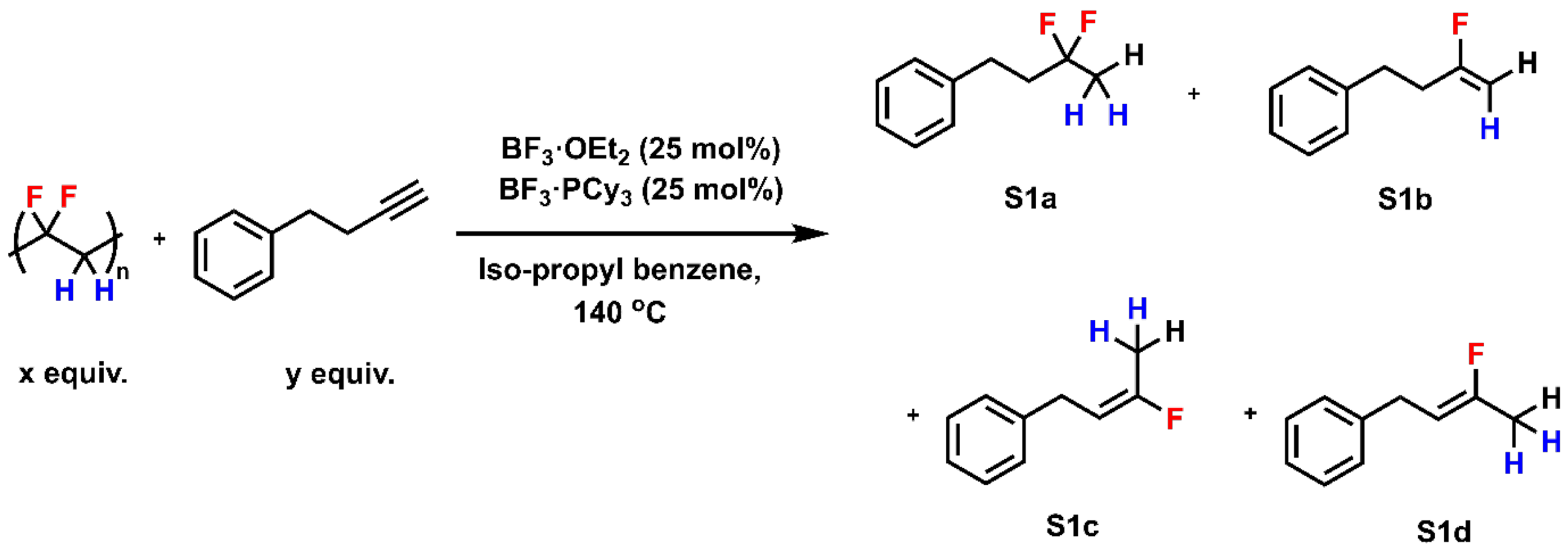

Entry n°.	X equiv.	Y equiv.	$[BF_3]:[PCy_3]$	Time (hours)	Yield **S1a** (%)	Yield **S1b** (%)	Yield **S1c** (%)	Yield **S1d** (%)	Total yield (**S1a** + **S1b** + **S1c** + **S1d**) (%)
1	1	2	2 : 1	30	1	7	11	34	**53**
2	1	1	2 : 1	12	9	18	6	61	**94**
3	2	1	2 : 1	7	23	14	4	44	**94**

Table S.3.5 Reaction stoichiometry optimisation for the reaction of PVDF (0.38 mmol, 2 equiv.) with 4-phenyl-1-butyne (0.19 mmol, 1 equiv.), catalysed by $BF_3{\cdot}OEt_2$, (0.048 mmol, 25 mol%) and cocatalyst, $BF_3{\cdot}PCy_3$ (0.048 mmol, 25 mol%) in iso-propylbenzene (0.2 mL), generating a mixture of fluorinated alkene and alkane products. Yields monitored by quantitative ^{19}F NMR spectroscopy against a fluorobenzene internal standard.

3.3.6 Acceptor scope

To investigate selectivity in product formation, a small scope of alkyne acceptors was examined, although poor selectivity for product formation was observed in all cases (**Table S.3.6**). This is expected to be a result of the reversibility of the reaction resulting in product isomerisation under the harsh reaction conditions.

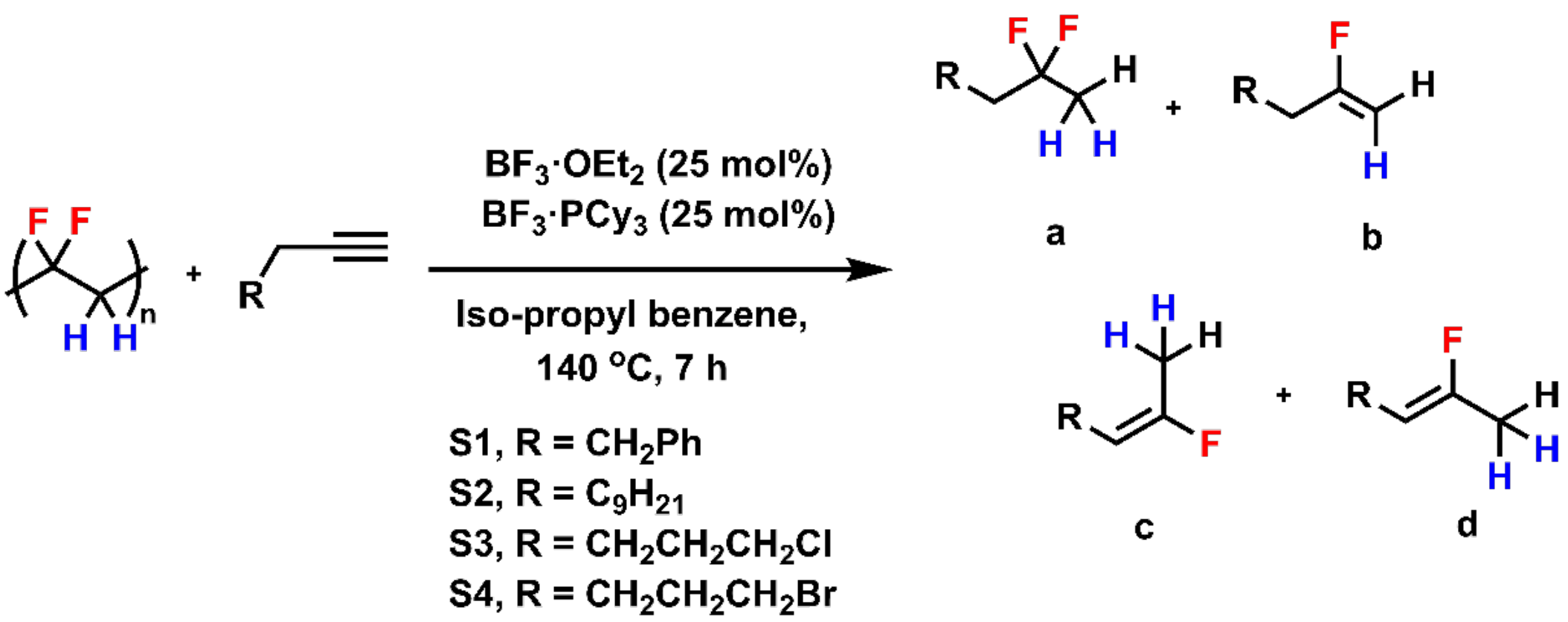

Entry n°.	Alkyne acceptor	Product number	Yield **a** (%)	Yield **b** (%)	Yield **c** (%)	Yield **d** (%)	Total yield (**a** + **b** + **c** + **d**) (%)
1	4-phenyl-1-butyne	**S1a-d**	23	14	4	44	**94**
2	1-dodecyne	**S2a-d**	35	4	12	8	**95**
3	6-chloro-1-hexyne	**S3a-d**	25	10	2	30	**67**
4	6-bromo-1-hexyne	**S4a-d**	9	12	6	40	**67**

Table S.3.6 Alkyne acceptor scope for the shuttle catalysis reaction of PVDF (0.38 mmol, 2 equiv.) with different alkyne acceptors (0.19 mmol, 1 equiv.) catalysed by $BF_3{\cdot}OEt_2$ (0.048 mmol, 25 mol%) with a $BF_3{\cdot}PCy_3$ cocatalyst (0.048 mmol, 25 mol%) in iso-propyl benzene (0.2 mL). Yields monitored by quantitative ^{19}F NMR spectroscopy against a fluorobenzene internal standard.

S3a ^{19}F NMR (377 MHz, $CDCl_3$) δ -90.53 (sextet, $^{1}J_{F\text{–}F}$ = 17.7 Hz, 2F, CF_2).

S3b ^{19}F NMR (377 MHz, $CDCl_3$) δ -94.10 (m, 1F, CF).

S3c ^{19}F NMR (377 MHz, $CDCl_3$) δ -94.75 – -95.29 (m, 1F, CF).

S3d ^{19}F NMR (377 MHz, $CDCl_3$) δ -101.63 (dq, $^3J_{F–H}$ = 33.4, $^3J_{F–H}$ = 16.5 Hz, 1F, CF)

S4a ^{19}F NMR (377 MHz, $CDCl_3$) δ -90.48 (sextet, $^1J_{C–F}$ = 17.4 Hz, 2F, CF_2)

S4b ^{19}F NMR (377 MHz, $CDCl_3$) δ -93.91 (m, 1F, CF)

S4c ^{19}F NMR (377 MHz, $CDCl_3$) δ -94.93 (dq, J = 50.1, 16.4 Hz, 1F, CF),

S4d ^{19}F NMR (377 MHz, $CDCl_3$) δ -101.38 (dq, $^3J_{F–H}$ = 33.9, $^3J_{F–H}$ 16.8 Hz, 1F, CF).

To prevent competitive isomerisation pathways through dehydrofluorination and hydrofluorination from yielding a mixture of fluorinated products, an acceptor lacking a β-hydrogen or with a functionality which would make isomerisation unfavourable was sought after. Acid anhydrides were employed to achieve this, generating acyl fluorides as a single fluorinated product (**Scheme S.3.2**).

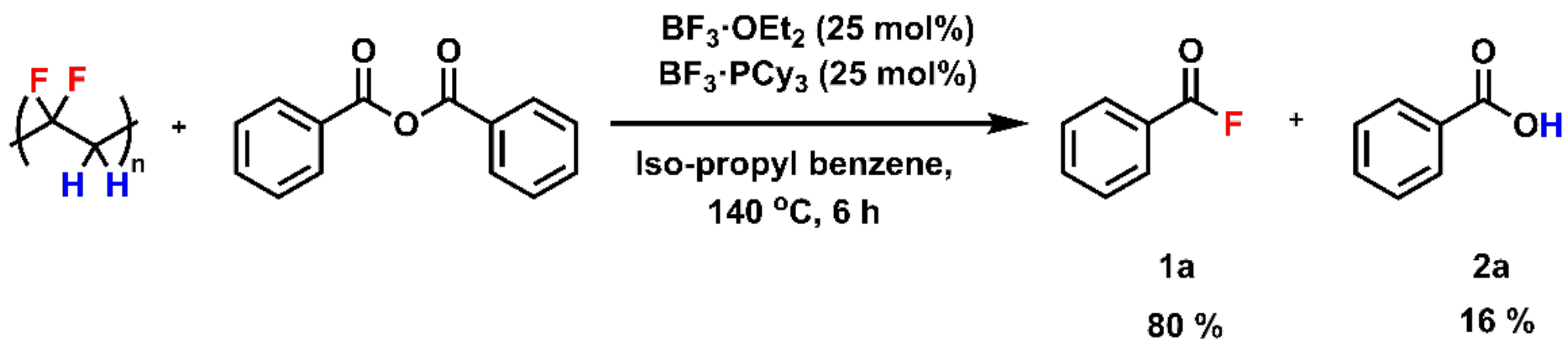

Scheme S.3.2 Reaction of PVDF (0.384 mmol, 2 equiv.) with benzoic anhydride (0.192 mmol, 2 equiv.) catalysed by $BF_3·OEt_2$ (0.048 mmol, 25 mol%) and $BF_3·PCy_3$ (0.048 mmol, 25 mol%) in iso-propyl benzene (0.6 mL). Yields monitored by quantitative ^{1}H and ^{19}F NMR spectroscopy against a fluorobenzene internal standard.

3.3.7 Solvent screen for reaction of PVDF with benzoic anhydride

A small solvent screen was performed to move away from the high boiling point iso-propyl benzene to assist in product isolation (**Table S.3.7**).

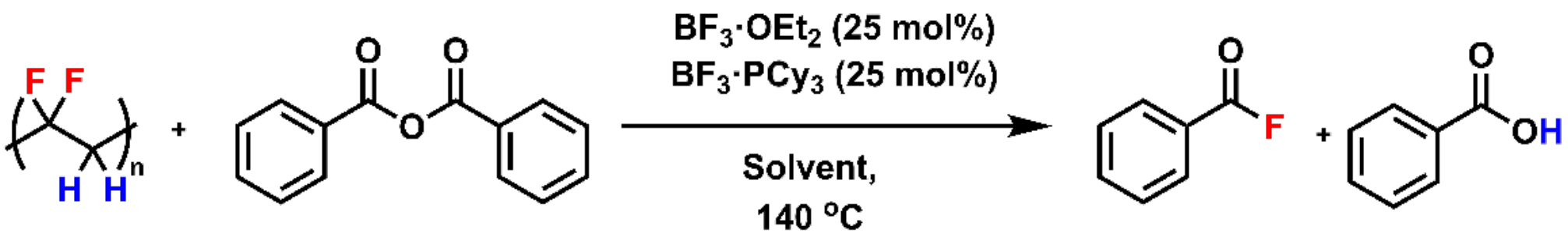

1a 2a

Entry n°.	Solvent	Time (hours)	Yield of **1a** (%)
1	Iso-propyl benzene	6	**80**
2	Toluene	6	**34**
3	Heptane	6	**0**

Table S.3.7 Solvent screen for the shuttle catalysis reaction between benzoic anhydride (0.19 mmol, 1 equiv.) and PVDF (0.38 mmol, 2 equiv.) catalysed by $BF_3 \cdot OEt_2$ (0.048 mmol, 25 mol%) with a $BF_3 \cdot PCy_3$ cocatalyst (0.048 mmol, 25 mol%) using 0.4 mL of various solvents. Yields monitored by quantitative ^{19}F NMR spectroscopy against a fluorobenzene internal standard.

3.3.8 Concentration optimisation for the reaction of PVDF with benzoic anhydride

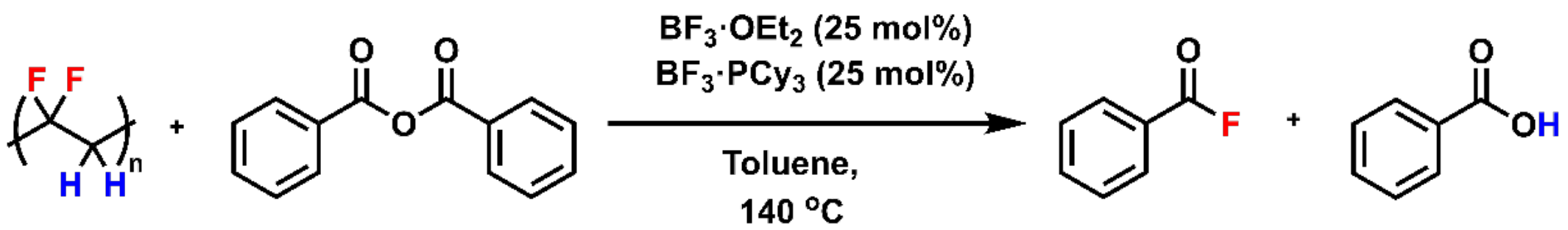

1a 2a

Entry n°.	Concentration of benzoic anhydride (mol dm^{-3})	Time (hours)	Yield of **1a** (%)	Yield of 2a (%)
2	0.48	10	**54**	8
3	0.32	14	**60**	8
4	0.24	9	**76**	18
5	0.19	8	**36**	13

Table S.3.8 Absolute reaction concentration optimisation for the shuttle catalysis reaction between benzoic anhydride (0.19 mmol, 1 equiv.) and PVDF (0.38 mmol, 2 equiv.) catalysed by $BF_3 \cdot OEt_2$ (0.048 mmol, 25 mol%) with a $BF_3 \cdot PCy_3$ cocatalyst (0.048 mmol, 25 mol%). Yields monitored by quantitative ^{1}H and ^{19}F NMR spectroscopy against a fluorobenzene internal standard.

3.4 Shuttle catalysis of fluoroethane

3.4.1 Temperature optimisation

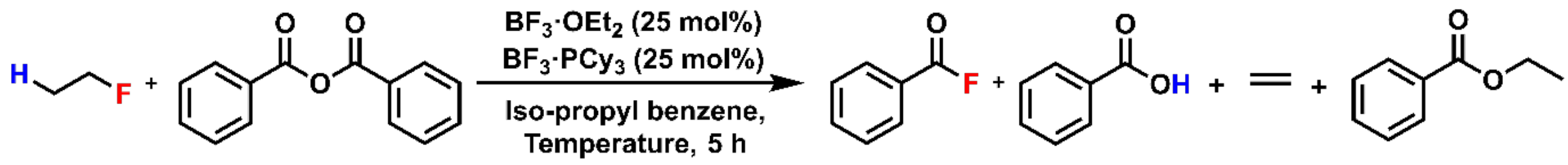

1a 2a 3a 4a

Entry	Temperature (°C)	Yield of **1a** (%)
1	140	**97**
2	100	**70**
3	60	**7**

Table S.3.9 Temperature optimisation for the $BF_3{\cdot}OEt_2$ (0.0044 mmol, 50 mol%) and $BF_3{\cdot}PCy_3$ (0.0044 mmol, 50 mol%) catalysed reaction of fluoroethane (0.13 mmol, 3 equiv.) and benzoic anhydride (0.044 mmol, 1 equiv.) in iso-propyl benzene (0.6 mL). Yields monitored by quantitative ^{19}F NMR spectroscopy using a fluorobenzene internal standard.

3.4.2 Variation of the ratio of fluoroethane and acid anhydride reagents

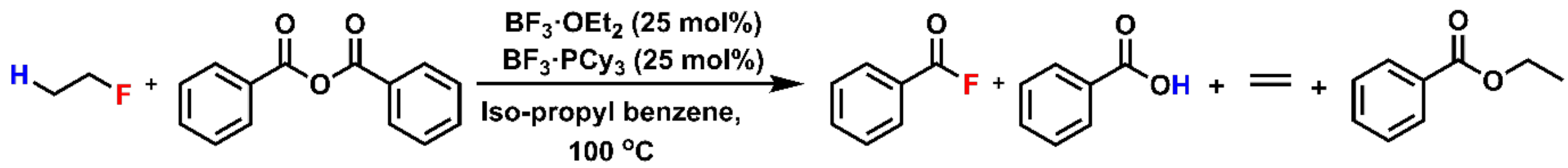

x equiv. y equiv. 1a 2a 3a 4a

Entry	x equiv.	y equiv.	Time (hours)	Yield of **1a** (%)
1	3	1	5	**70**
2	2	1	5	**38**
3	2	1	10	**62**
4	1	1	10	**16**

Table S.3.10 Variation of the ratio of fluoroethane and acid anhydride reagents in the $BF_3{\cdot}OEt_2$ (0.0044 mmol, 10 mol%) and $BF_3{\cdot}PCy_3$ (0.0044 mmol, 10 mol%) catalysed shuttle catalysis reactions of fluoroethane (0.13 mmol, 3 equiv.) and benzoic anhydride (0.044 mmol, 1 equiv.) in iso-propyl benzene (0.6 mL). Yields monitored by quantitative ^{1}H and ^{19}F NMR spectroscopy using a fluorobenzene internal standard.

3.4.3 Solvent screen

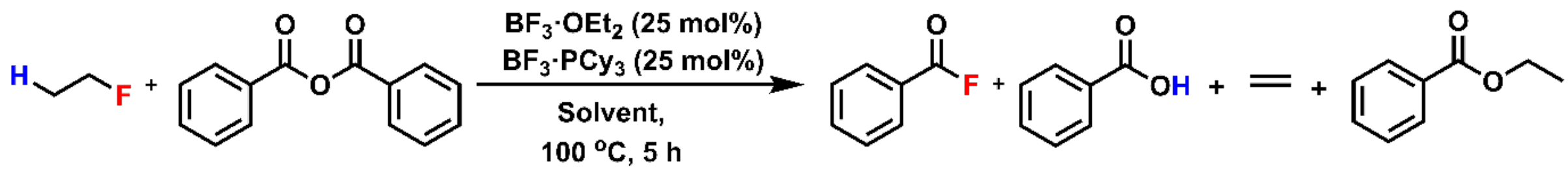

Entry	Solvent	Yield **1a** (%)
1	Iso-propyl benzene	**70**
2	$CDCl_3$	**35**
3	$CDCl_3$: iso-propyl benzene (10:1)	**40**
4	$CDCl_3$: iso-propyl benzene (1:1)	**51**
5	THF	**0**
6	C_6D_6	**59**
7	Toluene	**72**

Table S.3.11 Solvent screen for the BF_3·OEt_2 (0.022 mmol, 50 mol%) and BF_3·PCy_3 (0.022 mmol, 50 mol%) catalysed shuttle catalysis reaction of fluoroethane (0.13 mmol, 3 equiv.) and benzoic anhydride (0.044 mmol, 1 equiv.). Yields monitored by quantitative ^{19}F NMR spectroscopy using a fluorobenzene internal standard.

3.4.4 Catalyst and cocatalyst loading optimisation

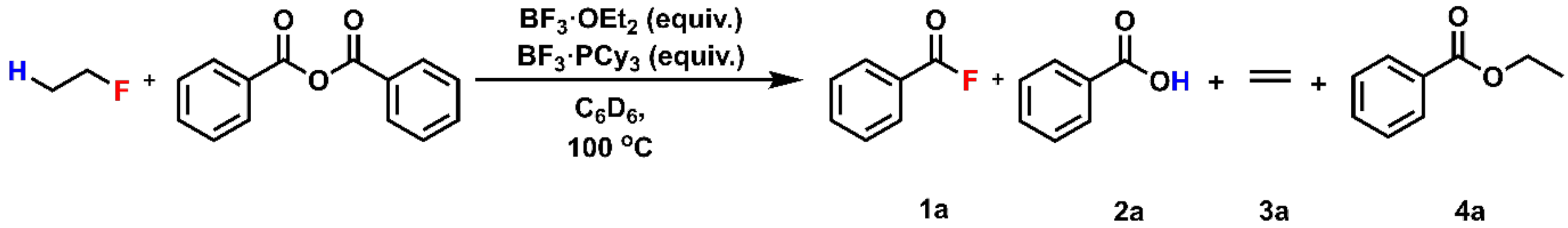

Entry	$BF_3{\cdot}OEt_2$ equiv.	$BF_3{\cdot}PCy_3$ equiv.	Time (hours)	Yield of **1a** (%)	Yield of **4a** (%)
1	1.00	1.00	5	**97**	92
2	0.50	0.50	5	**70**	62
3	0.25	0.25	10	**76**	81
4	0.10	0.10	10	**60**	42

Table S.3.12 Catalyst loading optimisation for the $BF_3{\cdot}OEt_2$ and $BF_3{\cdot}PCy_3$ catalysed shuttle catalysis reaction of fluoroethane (0.13 mmol, 3 equiv.) and benzoic anhydride (0.044 mmol, 1 equiv.) in C_6D_6 (0.6 mL). Yields monitored by quantitative ^{1}H and ^{19}F NMR spectroscopy using a fluorobenzene internal standard.

The role of catalyst speciation on reactivity was also considered (**Table S.3.13**).

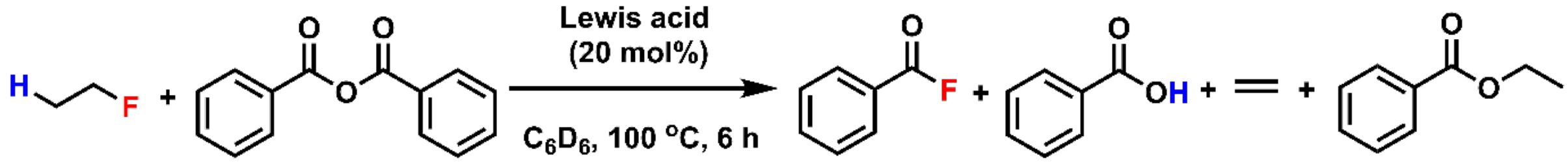

1a 2a 3a 4a

Entry n°.	Catalyst	Yield **1a** (%)	Yield **2a** (%)	Yield **3a** (%)	Yield **4a** (%)
1	BF_3·OEt_2	**45**	46	6	8
2	BF_3·X (X = OEt_2 10 mol% + PCy_3 10 mol%)	**60**	13	1	51
3	BF_3·OEt_2 (20 mol%) + PCy_3 (10 mol%)	**51**	21	8	29
4	BF_3·OEt_2 (20 mol%) + PCy_3 (20 mol%)	**7**	0	0	14
5	[BF_4][$HOEt_2$]	**50**	48	12	4
6	[BF_4][HX] (X = OEt_2 10 mol% + PCy_3 10 mol%)	**48**	0	1	50
7	[BF_4][$HPCy_3$]	**1**	3	0	7

Table S.3.13 Reaction of fluoroethane (0.13 mmol, 3 equiv.) with benzoic anhydride (0.044 mmol, 1 equiv.) catalysed by different Lewis acids (0.0088 mmol, 20 mol%) in C_6D_6 (0.6 mL). Yields monitored by quantitative ^{1}H and ^{19}F NMR spectroscopy against a fluorobenzene internal standard.

3.5 Shuttle catalysis reactions between fluoroethane and acid anhydrides

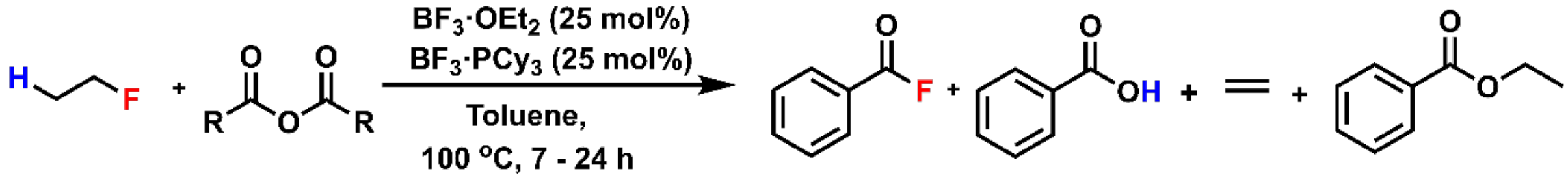

1a–h **2a–h** **3a** **4a–h**

Procedure C – NMR scale

In an N_2 filled glovebox, acid anhydride (0.044 mmol, 1 equiv.), $BF_3{\cdot}PCy_3$ (2 mg, 0.0044 mmol, 10 mol%) and fluorobenzene internal standard (4.16 µL) was added to a J. Young NMR tube. A 7.4 mmol L^{-1} solution of $BF_3{\cdot}OEt_2$ in toluene was prepared by addition of 25.20 µL of $BF_3{\cdot}OEt_2$ to 24 mL of toluene. 0.6 mL of the stock solution (0.0044 mmol $BF_3{\cdot}OEt_2$, 10 mol%) was added to the J. Young NMR tube before the tube was sealed and removed from the glovebox. The solution was degassed once via freeze-pump-thaw and fluoroethane (1.5 bar, 25 °C, 0.13 mmol, 3 equiv.) was added to the J. Young NMR tube. The J. Young NMR tube was inverted multiple times before being heated in a 100 °C silicone oil bath. After 7 – 24 hours, the solution was removed from the oil bath, allowed to cool to room temperature, and ^{19}F NMR spectra were obtained.

Procedure D – preparative scale

In an N_2 filled glovebox, acid anhydride (1.21 mmol), $BF_3{\cdot}OEt_2$ (15.00 µL, 0.12 mmol, 10 mol%) and $BF_3{\cdot}PCy_3$ (42 mg, 0.12 mmol, 10 mol%) in toluene (10 mL) was added to a 70 mL J. Young ampoule. The solution was degassed thrice via freeze-pump-thaw cycles and fluoroethane (1.5 bar, 25 °C, 60 mL headspace, 3.63 mmol, 3 equiv.) was added to the J. Young ampoule. The reaction mixture was heated to 100 °C in a silicone oil bath, after the reaction was complete the ampoule was removed from the oil bath and the mixture allowed to cool. The resulting crude mixture was directly loaded on to a silica column and immediately purified by column chromatography.

1a, benzoyl fluoride

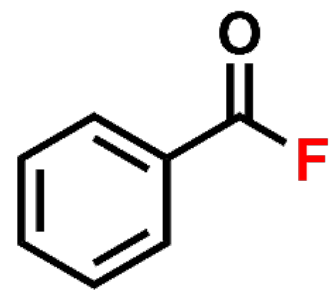

Yield monitored by quantitative ^{19}F NMR spectroscopy using a fluorobenzene internal standard using **Procedure C** (^{19}F NMR yield **= 72 ± 6 %**). Reaction performed for seven hours. Isolated as a clear oil using **Procedure D** (20:1 cyclohexane to acetone, R_f = 0.7) (91 mg, 0.73 mmol, **61 %**). The spectroscopic data matched those reported in the literature.[9]

^{1}H NMR (400 MHz, $CDCl_3$) δ 8.04 (m, 2H, C_{Ph}H), 7.70 (m, 1H, C_{Ph}H), 7.53 (m, 2H, C_{Ph}H). **^{13}C{^{1}H} NMR** (101 MHz, $CDCl_3$) δ 157.5 (d, $^{1}J_{C–F}$ = 344.4 Hz, **C**OF), 135.4 (s, C_{Ph}H), 131.5 (d, $^{3}J_{C–F}$ = 4.0 Hz, C_{Ph}H), 129.2 (s, C_{Ph}H), 125.0 (d, $^{2}J_{C–F}$ = 60.7 Hz, **C**$_{Ph}$(COF)). **^{19}F NMR** (377 MHz, $CDCl_3$) δ 18.05 (s, 1F, COF).

1b, 2,4,6-trimethylbenzoyl fluoride

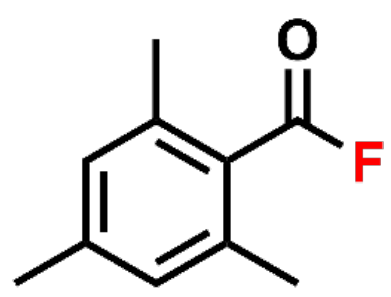

Yield monitored by quantitative ^{19}F NMR spectroscopy using a fluorobenzene internal standard using **Procedure C** (^{19}F NMR yield **= 64 ± 9 %**). Reaction performed for 15 hours. Isolated as a white solid using **Procedure D** (10:1 toluene to acetone, R_f = 0.8) (114 mg, 0.71 mmol, **57 %**). The spectroscopic data matched those reported in the literature.[10]

^{1}H NMR (400 MHz, $CDCl_3$) δ 6.91 (s, 2H, C_{Ph}H), 2.45 (s, 6H, CH_3), 2.32 (s, 3H, CH_3). **^{13}C{^{1}H} NMR** (101 MHz, $CDCl_3$) δ 157.2 (d, $^{1}J_{C–F}$ = 353.1 Hz), 142.9 (s, C_{Ph}), 139.7 (s, C_{Ph}), 129.8 (s, C_{Ph}), 123.6 (d, $^{2}J_{C–F}$ = 52.8 Hz, **C**$_{Ph}$(COF)), 21.6 (s, CH_3), 20.7 (s, CH_3). **^{19}F NMR** (377 MHz, $CDCl_3$) δ 52.51.

1c, 4-bromobenzoyl fluoride

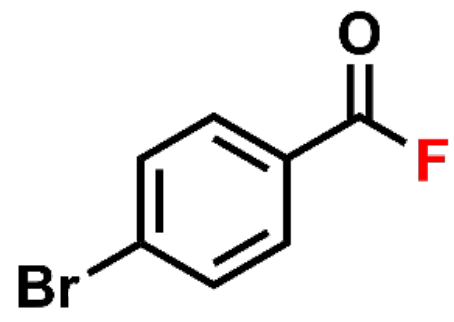

Yield monitored by quantitative ^{19}F NMR spectroscopy using a fluorobenzene internal standard using **Procedure C** (^{19}F NMR yield **= 52 ± 5 %**). Reaction performed for seven hours. Isolated as a white solid using **Procedure D** (20:1 toluene to acetone, R_f = 0.8) (206 mg, 1.01 mmol, **84 %**). The spectroscopic data matched those reported in the literature.[11]

^{1}H NMR (400 MHz, $CDCl_3$) δ 7.99 – 7.87 (m, 2H, C_{Ph}H), 7.71 (d, $^3J_{H–H}$ = 8.8 Hz, 2H, C_{Ph}H). **^{13}C{^{1}H} NMR** (101 MHz, $CDCl_3$) δ 156.8 (d, $^1J_{C–F}$ = 343.8 Hz, COF), 132.8 (d, $^3J_{C–F}$ = 3.7 Hz, C_{Ph}H), 132.6 (s, C_{Ph}), 131.0 (s, C_{Ph}Br), 123.8 (d, $^2J_{C–F}$ = 62.6 Hz, **C_{Ph}**COF). **^{19}F NMR** (377 MHz, $CDCl_3$) δ 18.4 (s, 1F, COF).

1d, 2,6-difluorobenzoyl fluoride

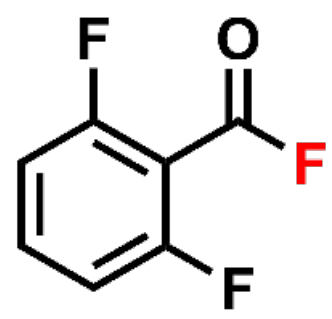

Yield monitored by quantitative ^{19}F NMR spectroscopy using a fluorobenzene internal standard using **Procedure C** (^{19}F NMR yield **= 65 %**). Reaction performed for eight hours. The spectroscopic data matched those reported in the literature.[11] Product not isolated.

^{1}H NMR (400 MHz, C_6D_6) δ 6.96 – 6.90 (m, 1H, C_{Ph}H), 6.65 – 6.51 (m, 2H, C_{Ph}H). **^{19}F NMR** (377 MHz, C_6D_6) δ 47.1 (t, $^4J_{F–F}$ = 39.0, 1F, COF), -108.7, (m, 2F, C_{Ph}F)

1e, 4-trifluoromethylbenzoyl fluoride

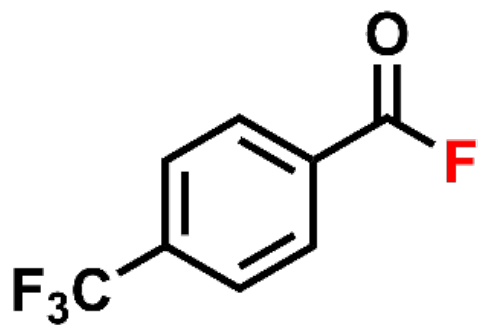

Yield monitored by quantitative ^{19}F NMR spectroscopy using a fluorobenzene internal standard using **Procedure C** (^{19}F NMR yield **= 62** ± **4 %**). Reaction performed for seven hours. Isolation achieved by chromatography in 20:1 toluene : acetone using **Procedure D** (R_f = 0.76). The spectroscopic data matched those reported in the literature.[10]

^{1}H NMR (400 MHz, $CDCl_3$) δ 8.22 (d, $^3J_{H–H}$ = 8.2 Hz, 2H, C_{Ph}H), 7.84 (d, $^3J_{H–H}$ = 8.2 Hz, 2H, C_{Ph}H). **^{13}C{^{1}H} NMR** (126 MHz, $CDCl_3$) δ 156.3 (d, $^1J_{C–F}$ = 345.7 Hz, COF), 136.8 (q, $^2J_{C–F}$ = 33.0 Hz, **C_{Ph}**CF_3), 132.0 (d, $^3J_{C–F}$ = 4.2 Hz, **C_{Ph}**H), 126.3 (q, $^3J_{C–F}$ = 3.8 Hz, **C_{Ph}**H) 123.3 (q, $^1J_{C–F}$ = 273.1 Hz, CF_3). ***C_{Ph}****COF peak obscured by toluene impurity.* **^{19}F NMR** (377 MHz, $CDCl_3$) δ 20.01 (s, 1F, COF), -63.4 (s, 3F, CF_3).

1f, 4-methoxybenzoyl fluoride

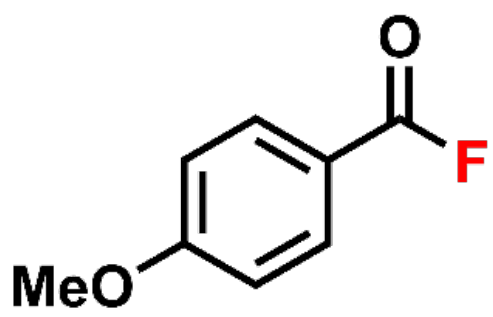

Yield monitored by quantitative ^{19}F NMR spectroscopy using a fluorobenzene internal standard using **Procedure C** (^{19}F NMR yield **= 55** ± **6 %**). Reaction performed for seven hours. Isolated as a white solid using P**rocedure D** (20:1 toluene to acetone) (97 mg, 0.62 mmol, **52 %**). The spectroscopic data matched those in literature.[10]

^{1}H NMR (400 MHz, $CDCl_3$) δ 8.17 – 7.81 (m, 2H, C_{Ph}H), 7.08 – 6.92 (m, 2H, C_{Ph}H), 3.93 (s, 3H, OCH_3). **^{13}C{^{1}H} NMR** (126 MHz, $CDCl_3$) δ 165.4 (s, **C_{Ph}**OCH_3), 157.4 (d, $^1J_{C–F}$ = 339.8 Hz, COF), 133.9 (d, $^3J_{C–F}$ = 3.9 Hz, C_{Ph}H), 117.0 (d, $^2J_{C–F}$ = 61.8 Hz, **C_{Ph}**COF), 114.6 (s, C_{Ph}H), 55.8 (OCH_3). **^{19}F NMR** (377 MHz, $CDCl_3$) δ 16.0 (s, 1F, COF).

1g, naphthoic fluoride

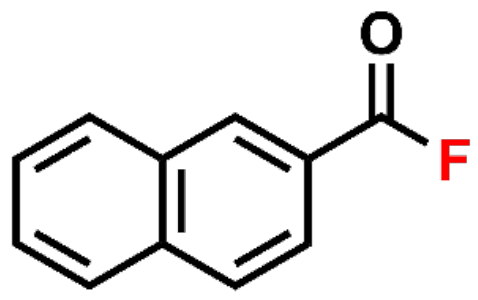

Yield monitored by quantitative ^{19}F NMR spectroscopy using a fluorobenzene internal standard using **Procedure C** (^{19}F NMR yield **= 81** ± **4 %**). Reaction performed for seven hours. Isolated as a white solid by column chromatography in n-pentane using **Procedure D** (16 mg, 70 %, R_f = 0.6). The spectroscopic data matched those reported in the literature.[11]

^{1}H NMR (400 MHz, $CDCl_3$) δ 8.65 (s, 1H, C_{Ph}H), 8.18 – 7.83 (m, 4H, C_{Ph}H), 7.65 (m, 2H, C_{Ph}H). **^{19}F NMR** (377 MHz, $CDCl_3$) δ 18.1. **^{13}C{^{1}H} NMR** (101 MHz, $CDCl_3$) δ 157.7 (d, $^1J_{C-F}$ = 343.4 Hz, COF), 136.5 (s, C_{Ph}), 134.0 (s, C_{Ph}), 132.3 (s, C_{Ph}), 129.7 (s, C_{Ph}H), 129.1 (s, C_{Ph}H), 128.0 (s, C_{Ph}H), 127.4 (s, C_{Ph}H), 125.6 (s, C_{Ph}H), 122.4 (s, C_{Ph}H), 121.8 (s, C_{Ph}H).

1h, acetyl fluoride

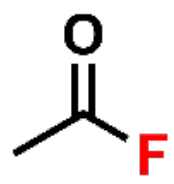

Yield monitored by quantitative ^{19}F NMR spectroscopy using a fluorobenzene internal standard using **Procedure C** (^{19}F NMR yield **= 67 %**). Reaction performed for ten hours. Product not isolated. The spectroscopic data matched those reported in the literature.[12]

^{19}F NMR (377 MHz, None) δ 49.99 (q, J = 6.9 Hz).

3.6 Mechanistic investigation

3.6.1 Control Reactions

Control reactions were conducted (**Scheme S.3.3**). To show the $BF_3{\cdot}OEt_2$ and $BF_3{\cdot}PCy_3$ catalysts were not acting as an HF source, a reaction was performed in the absence of fluoroethane. No product formation was observed, confirming that the catalyst and cocatalyst species were not directly reacting with the acid anhydride independently of the fluoroethane.

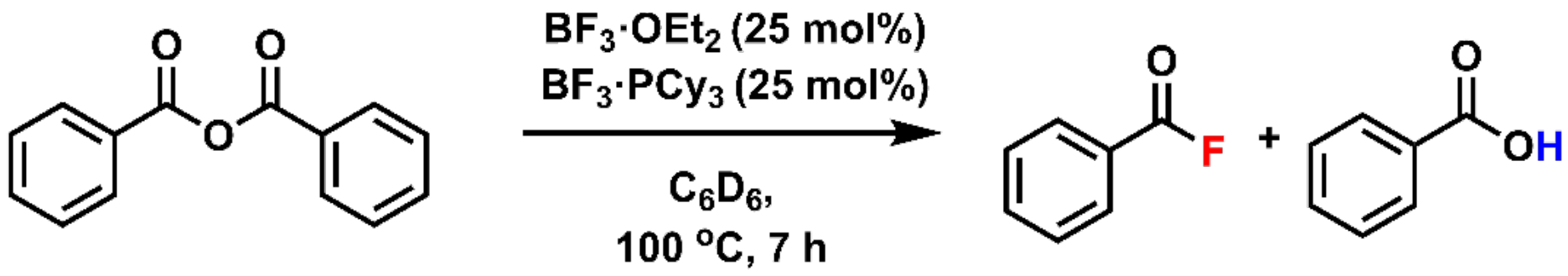

1a 0 %   2a 0 %

Scheme S.3.3 Control reaction of benzoic anhydride (0.0444 mmol, 1 equiv.) with $BF_3{\cdot}OEt_2$ (0.0111 mmol, 25 mol%) and $BF_3{\cdot}PCy_3$ (0.0111 mmol, 25 mol%) in the absence of fluoroethane in C_6D_6 (0.6 mL). Yields calculated by quantitative ^{1}H and ^{19}F NMR spectroscopy against a fluorobenzene internal standard.

To show both catalyst $BF_3{\cdot}OEt_2$ and cocatalyst $BF_3{\cdot}PCy_3$ were necessary for the reaction to proceed, control reactions were performed in their absence (**Table S.3.14**). In the absence of only $BF_3{\cdot}PCy_3$, the reaction proceeds more sluggishly, with only 12 % formation of the benzoyl fluoride product. In the absence of $BF_3{\cdot}OEt_2$, no formation of the acyl fluoride product was observed.

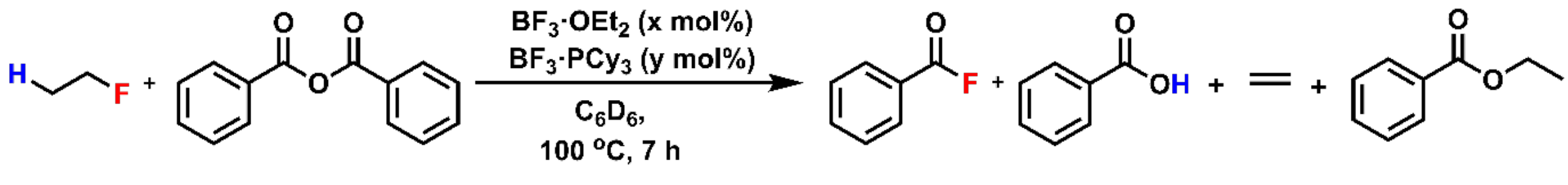

1a **2a** **3a** **4a**

Entry n°.	x equiv.	y equiv.	Yield **1a** (%)
1	0	0	**0**
2	0	1	**0**
3	1	0	**12**
4	0.1	0.1	**72**

Table S.3.14 Control reactions performed in the shuttle catalysis reaction between fluoroethane (0.133 mmol, 3 equiv.) and benzoic anhydride (0.044 mmol, 1 equiv.) in the absence of catalyst $BF_3{\cdot}OEt_2$, cocatalyst $BF_3{\cdot}PCy_3$, and both in C_6D_6 (0.6 mL). Yields monitored by quantitative ^{1}H and ^{19}F NMR spectroscopy against a fluorobenzene internal standard.

Shuttle catalysis reactions are necessarily reversible. To show the reaction remains partially reversible when using benzoic anhydride as an acceptor, a control reaction was performed between benzoyl fluoride, benzoic acid and 4-phenyl-1-butyne, generating **S1a** and **S1b** in 20 % overall yield (**Scheme S.3.4**).

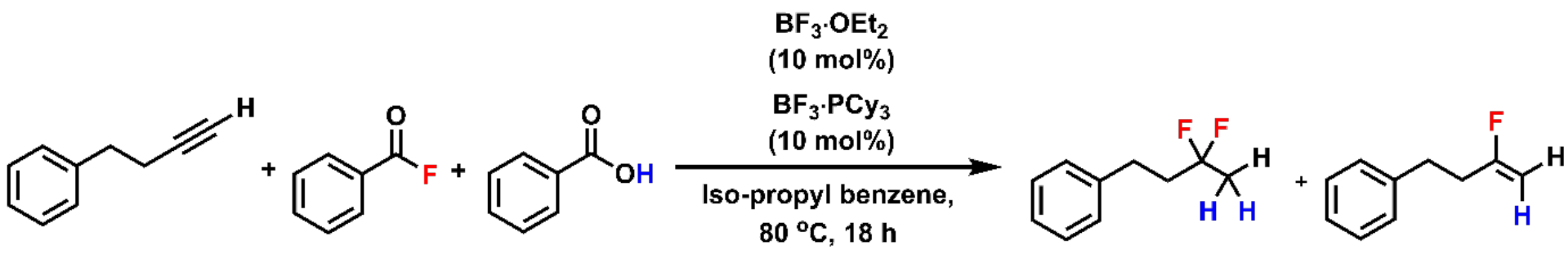

1a **2a** **S1a** 8 % **S1b** 12 %

Scheme S.3.4 Reaction of benzoyl fluoride (0.76 mmol, 2 equiv.) and benzoic acid (0.76 mmol, 2 equiv.) with 4-phenyl-1-butyne (0.38 mmol, 1 equiv.) catalysed by $BF_3{\cdot}OEt_2$ (0.038 mmol, 10 mol%) and $BF_3{\cdot}PCy_3$ (0.038 mmol, 10 mol%) in iso-propyl benzene (0.6 mL). Yields monitored by quantitative ^{19}F NMR spectroscopy against a fluorobenzene internal standard.

3.6.2 Role of $BF_3{\cdot}PCy_3$

Experimental investigation into the role of $BF_3{\cdot}PCy_3$ was performed. Similar to its effect on the reaction of PVDF and 4-phenyl-1-butyne, addition of 5 mol% of $BF_3{\cdot}PCy_3$ reduced the reaction time and increased yield for the reaction of 1-dodecyne with 1-fluoroheptane (**Scheme S.3.5)**. In this reaction, the generation of $[BF_4][HPCy_3]$ was observed.

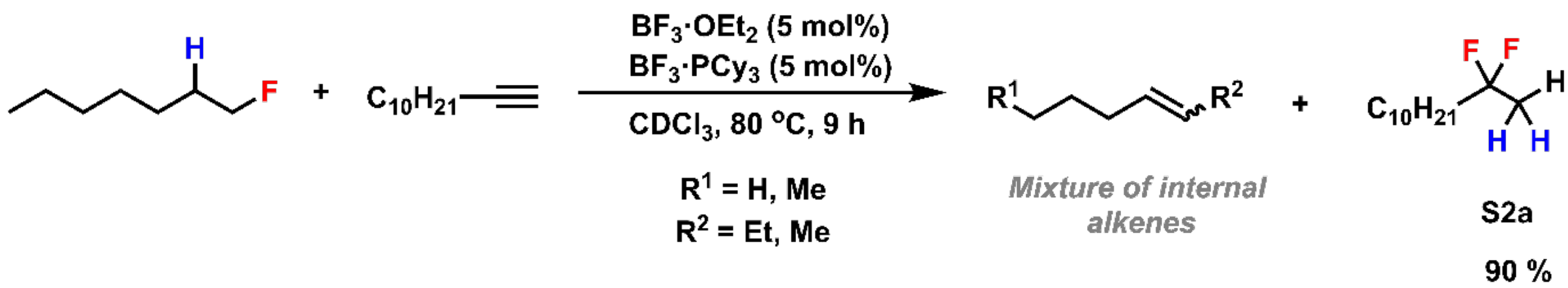

Scheme S.3.5 Reaction of 1-dodecyne (0.384 mmol, 1 equiv.) with 1-fluoroheptane (0.768 mmol, 2 equiv.), catalysed by $BF_3{\cdot}OEt_2$ (0.0192 mmol, 5 mol%) and $BF_3{\cdot}PCy_3$ (0.0192 mmol, 5 mol%) in $CDCl_3$ (0.6 mL). Yields calculated by ^{19}F NMR spectroscopy against a fluorobenzene internal standard.

The ability of $BF_3{\cdot}PCy_3$ to act as a defluorination reagent was investigated. In the absence of $BF_3{\cdot}OEt_2$ and an HF acceptor, incomplete reactivity was observed (**Scheme S.3.6.a**), with slow consumption of 1-fluoroheptane and the formation of a phosphonium salt and $[BF_4][HPCy_3]$. In the presence of a $BF_3{\cdot}OEt_2$, complete consumption of 1-fluoroheptane to generate a mixture of heptene isomers was observed, with the formation of $[BF_4][HPCy_3]$ and a phosphonium salt assigned as $[n\text{-}heptPCy_3][BF_4]$ (**Scheme S.3.6.b**).

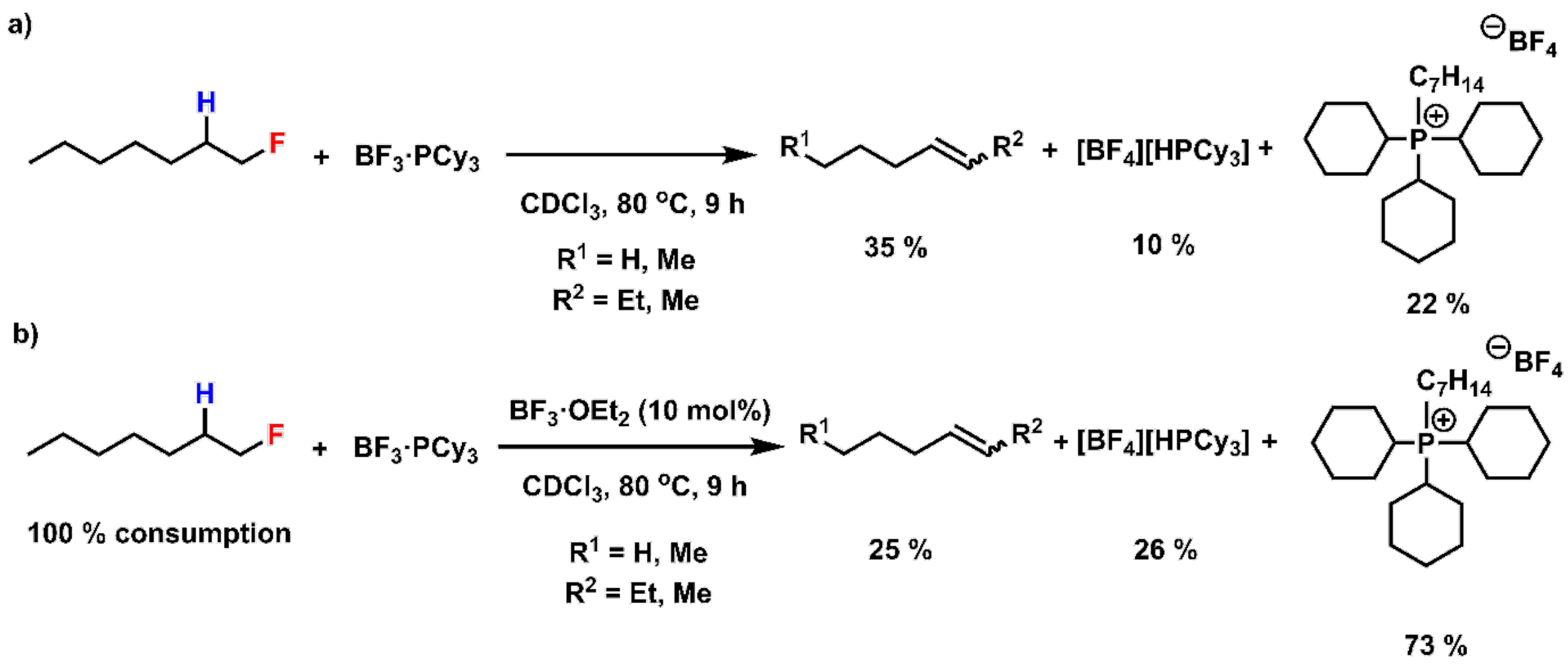

Scheme S.3.6 **a**) Reaction of BF_3·PCy_3 (0.384 mmol, 1 equiv.) with fluoroheptane (0.768 mmol, 1 equiv.) in $CDCl_3$ (0.6 mL) in the absence of BF_3·OEt_2, showing slow conversion to a mixture of heptane isomers, $HPCy_3$·BF_4 and a phosphonium salt. **b**) Reaction of BF_3·PCy_3 (0.384 mmol, 1 equiv.) with 1-fluoroheptane (0.768 mmol, 2 equiv.) in $CDCl_3$ (0.6 mL), with the addition of BF_3·OEt_2 (0.0384 mmol, 10 mol%), generating complete consumption of 1-fluoroheptane into a mixture of heptene isomers, a phosphonium salt, and $HPCy_3$·BF_4.

Experiments were also performed to investigate the effect of $HPCy_3$·BF_4 on reactivity – whilst $HPCy_3$·BF_4 improves reactivity when compared to the reaction in the absence of a phosphine co-catalyst, the effect was less pronounced than that of BF_3·PCy_3, generating a 51 % yield of a mixture of fluorinated products in the reaction of 1-dodecyne with PVDF (**Table S.3.15**).

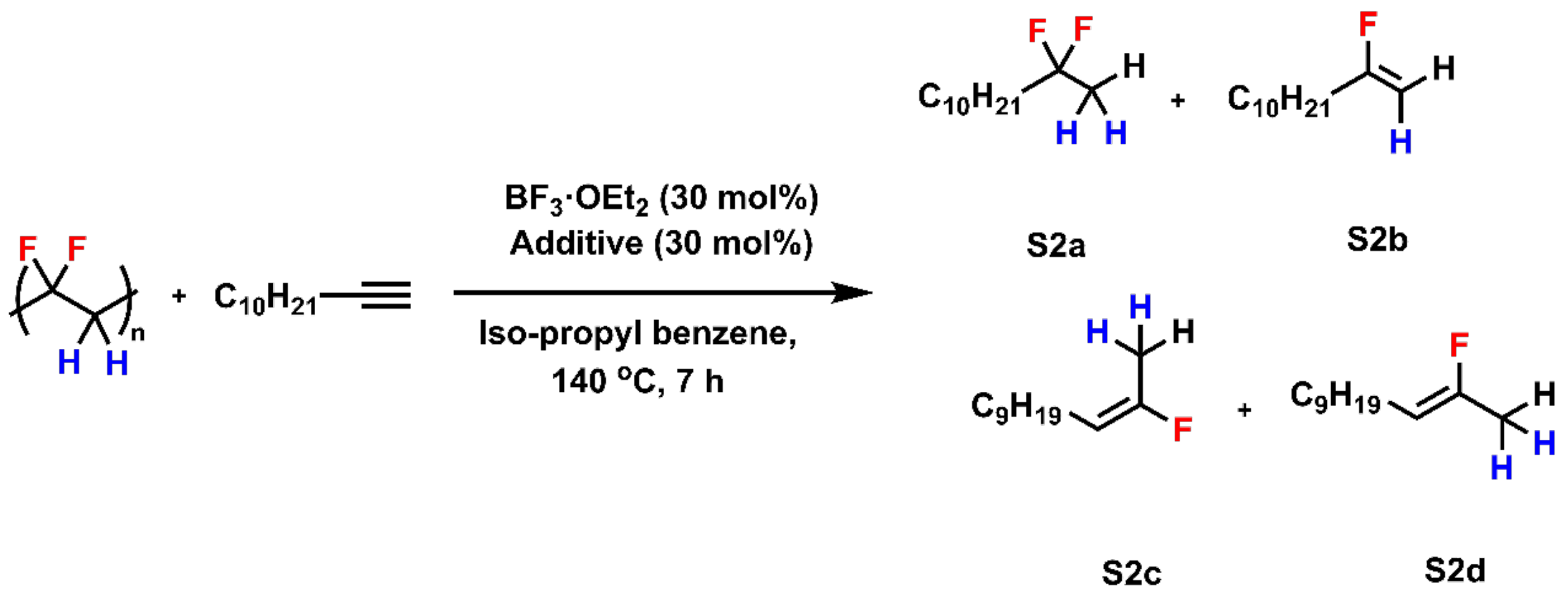

Entry n°.	Additive	Yield **S2a** (%)	Yield **S2b** (%)	Yield **S2c** (%)	Yield **S2d** (%)	Total yield (**S2a** + **S2b** + **S2c** + **S2d**) (%)
1	None	1	2	0	4	**7**
2	$BF_3{\cdot}PCy_3$	23	14	4	44	**94**
3	$[BF_4][HPCy_3]$	17	2	8	24	**51**

Table S.3.15 Reaction of PVDF (0.384 mmol, 2 equiv.) with 1-dodecyne (0.192 mmol, 1 equiv.) in iso-propyl benzene (0.2 mL) catalysed by $BF_3{\cdot}OEt_2$ (30 mol%) and different phosphine additives generating a mixture of fluorinated products. Yields calculated by ^{19}F NMR spectroscopy against a fluorobenzene internal standard.

On mixing $[BF_4][HOEt_2]$ with $BF_3{\cdot}PCy_3$, complete conversion to generate $HPCy_3{\cdot}BF_4$ and $BF_3{\cdot}OEt_2$ was observed (**Scheme S.3.7**).

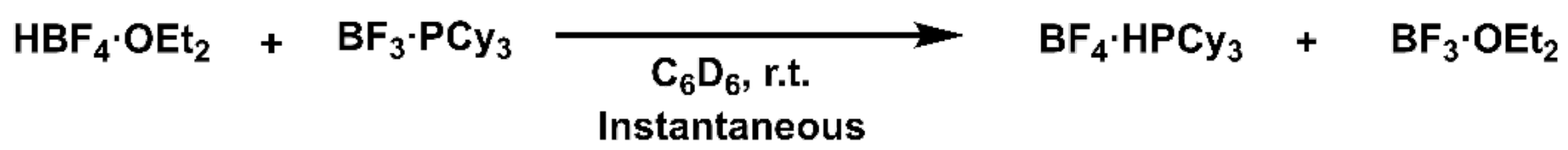

Scheme S.3.7 Reaction of $[BF_4][HOEt_2]$ and $BF_3{\cdot}PCy_3$ in C_6D_6 to generate $BF_4{\cdot}HPCy_3$ and $BF_3{\cdot}PCy_3$.

It was hypothesised the $BF_3{\cdot}PCy_3$ cocatalyst plays a role in stabilising the $BF_3{\cdot}OEt_2$ catalyst for high temperature reactions, reducing the rate of decomposition such that there is a steadier state of BF_3 throughout the reaction. $BF_3{\cdot}PCy_3$ has a melting point of 145 – 150 °C. On packing large volumes of $BF_3{\cdot}PCy_3$ in a capillary with rapid temperature increase, slight yellowing began at 180 °C.

Reaction of $BF_3 \cdot PCy_3$ with benzoic anhydride generated a phosphonium salt intermediate, which was tentatively assigned as the activated benzoic acid salt product (**Figure S.3.2**). Similar acyl phosphonium salts have previously been shown to be potent acylating reagents.[13]

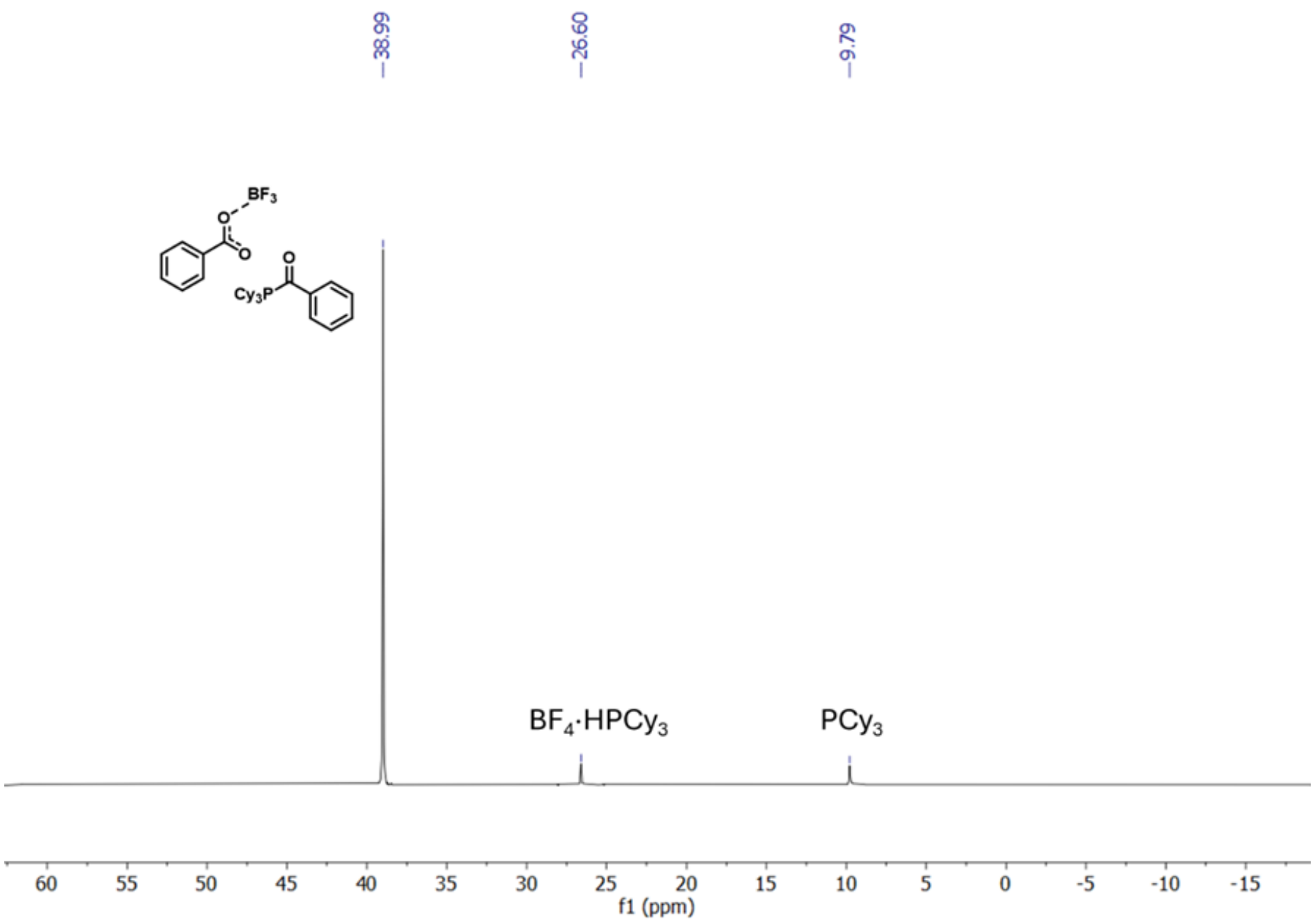

Figure S.3.2 $^{31}P\{^{1}H\}$ NMR of the reaction of benzoic anhydride (0.19 mmol, 1 equiv.) with $BF_3 \cdot PCy_3$ (0.19 mmol, 1 equiv.) generating a phosphonium salt which was tentatively assigned as the activated acylphosphonium salt.

3.6.3 Hydrofluorination of acid anhydrides

Reaction of $[BF_4][OEt_2]$ (0.768 mmol, 2 equiv.) with benzoic anhydride (0.192 mmol, 1 equiv.) resulted in complete conversion to benzoyl fluoride and benzoic acid instantaneously at room temperature (**Scheme S.3.8.c**) – no ester formation was observed.

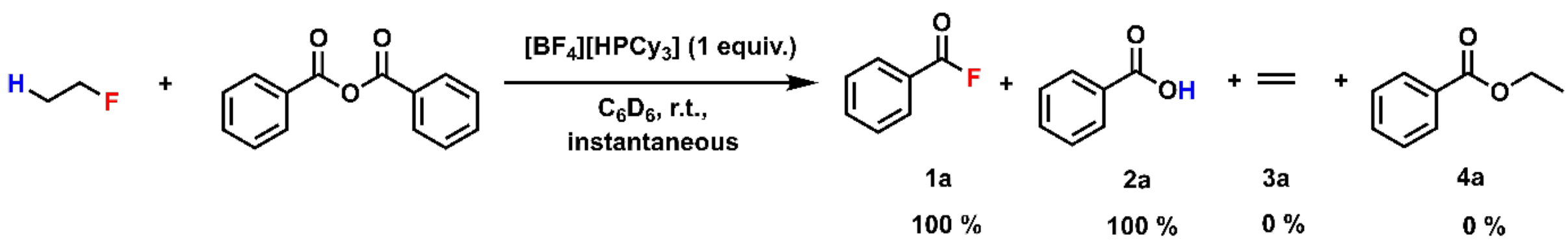

Scheme S.3.8 Reaction of $[BF_4][HOEt_2]$ (0.768 mmol, 2 equiv.) with benzoic anhydride (0.368 mmol, 1 equiv.) in C_6D_6, generating benzoyl fluoride. Yields monitored by quantitative ^{1}H and ^{19}F NMR spectroscopy against a fluorobenzene internal standard.

3.6.3 Mechanism of ester formation

Control reactions were performed to elucidate the mechanism of ester side product formation. Ester formation can occur from onwards reaction of the carboxylic acid product of the shuttle catalysis reaction – this was shown by the $BF_3{\cdot}OEt_2$ and $BF_3{\cdot}PCy_3$ catalysed reaction of fluoroethane with 4-methoxybenzoic acid, which generated ethyl 4-methoxybenzoate (**Scheme S.3.9.a**). In the absence of $BF_3{\cdot}OEt_2$, no product formation was observed (**Scheme S.3.9.b**). The reaction could be separated into steps, with the generation of a 1:1 mixture of a tetraalkylphosphonium salt intermediate, $[BF_4][EtPCy_3]$, and $HPCy_3{\cdot}BF_4$ from reaction of fluoroethane with $BF_3{\cdot}PCy_3$ in the presence of $BF_3{\cdot}OEt_2$ (**Scheme S.3.9.c**).

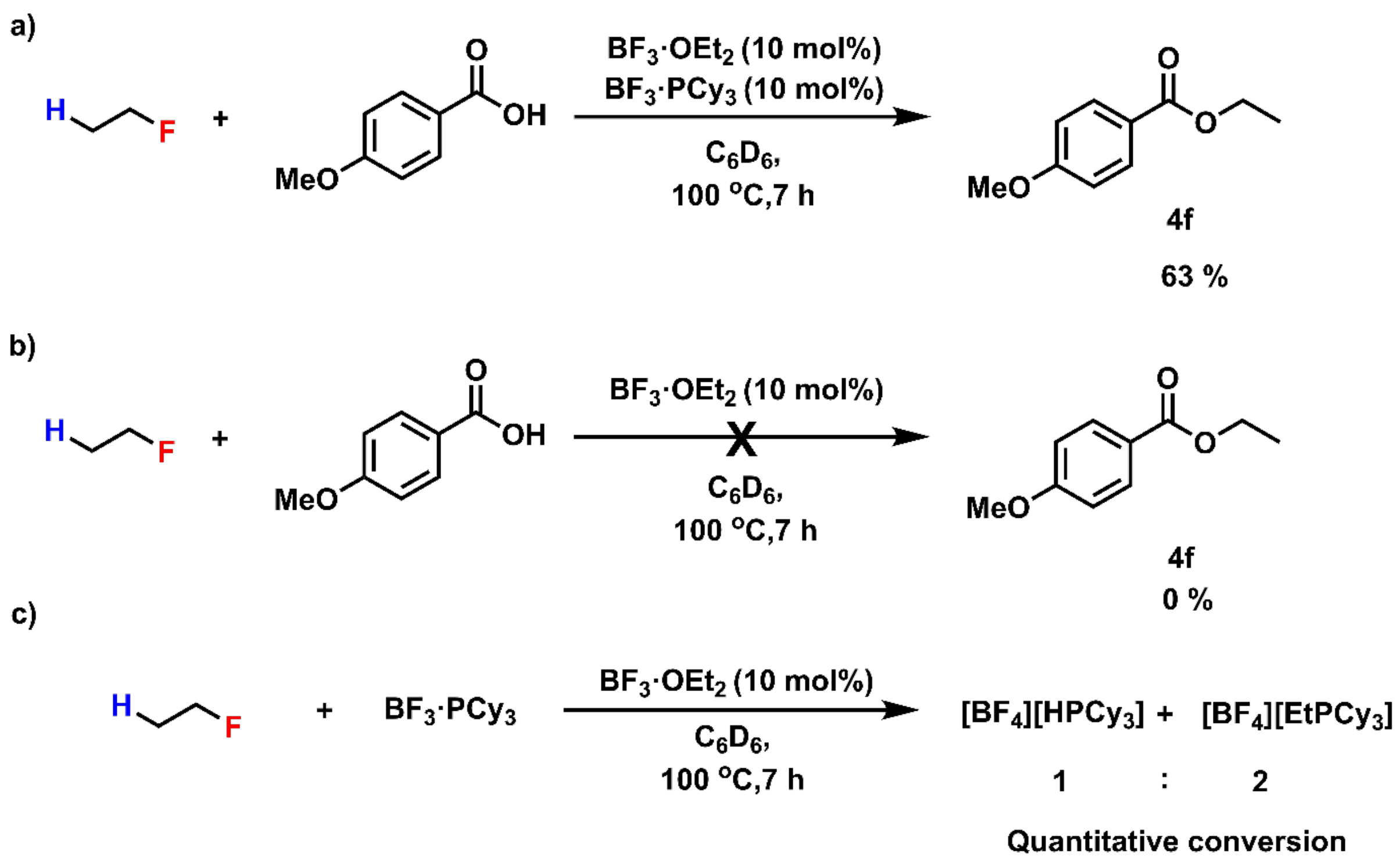

Scheme S.3.9 **a**) Reaction of fluoroethane with $BF_3{\cdot}PCy_3$ to generate a mixture of $HPCy_3{\cdot}BF_4$ and a tetraalkylphosphonium salt. **b**) Reaction of fluoroethane with $BF_3{\cdot}PCy_3$ to generate an intermediate phosphonium salt in the presence of $BF_3{\cdot}OEt_2$, followed by addition of 4-methoxybenzoic acid, resulting in the formation of ethyl 4-methoxybenzoate. **c**) Reaction of flurooethane with $BF_3{\cdot}PCy_3$ to generate an intermediate phosphonium salt in the absence of $BF_3{\cdot}OEt_2$, followed by addition of 4-methoxybenzoic acid, in which no formation of ethyl 4-methoxybenzoate was observed. Yields monitored by quantitative ^{1}H NMR spectroscopy against a fluorobenzene internal standard.

To confirm the identity of the phosphonium salt as $[BF_4][EtPCy_3]$, it was independently synthesised. A solution of $BF_4{\cdot}OEt_3$ (72 mg, 0.38 mmol, 1 equiv.) and PCy_3 (106 mg, 0.38 mmol, 1 equiv.) in C_6D_6 was heated at 100 °C for 8 hours, after which time the solution was concentrated in vacuo and recrystallised from diethyl ether, yielding $[BF_4][EtPCy_3]$ as a crystalline solid (107 mg, 0.35 mmol, 91 %). The spectroscopic data matched those reported in the literature.[14]

^{1}H NMR (400 MHz, C_6D_6) δ 2.27 (td, J = 13.8, 6.9 Hz, 3H), 2.01 – 1.43 (m, 23H), 1.44 – 0.84 (m, 21H). **^{13}C{^{1}H} NMR** (101 MHz, C_6D_6) δ 29.5 (d, $^{1}J_{C-P}$ = 40.7 Hz, P**C**$(CH_2)_2$), 27.0 (d, $^{3}J_{C-P}$ = 4.1 Hz, CH_2), 26.4 (d, $^{2}J_{C-P}$ = 12.0 Hz, CH_2), 25.8 (s, Cy, CH_2), 8.69 (d, $^{1}J_{C-P}$ = 45.3 Hz, P**C**H_2CH_3), 6.83 (d, $^{2}J_{C-P}$ = 5.8 Hz, CH_3). **^{19}F NMR** (377 MHz, C_6D_6) δ -149.85 (s, BF_4)**.** **^{31}P{^{1}H} NMR** (162 MHz, C_6D_6) δ 32.64 (s, 1P, $BF_4{\cdot}P(C_2H_5)(C_6H_{11})_3$)**.** **^{11}B NMR** (128 MHz, C_6D_6) δ -0.27 (s, BF_4). **MS(EI)**: 281.22 ($[C_{18}H_{34}P]^+$, M^+), 309.27 ($[C_{20}H_{38}P]^+$, M^+).

To show $[BF_4][EtPCy_3]$ was not acting as an alkylating reagent by itself, it was independently synthesised by addition of PCy_3 to $BF_4{\cdot}OEt_3$ – on addition of benzoic anhydride, no product formation was observed (**Scheme S.3.10**).

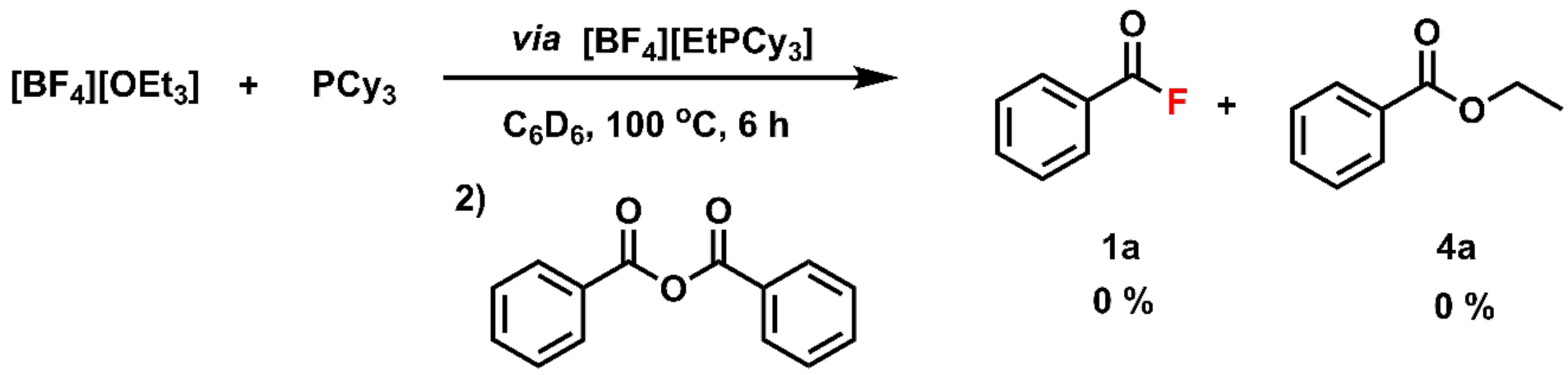

Scheme S.3.10 Reaction of $BF_4{\cdot}OEt_3$ (0.19 mmol, 1 equiv.) with PCy_3 (0.23 mmol, 1.2 equiv.) and benzoic anhydride (0.19 mmol, 1 equiv.) in C_6D_6 (0.6 mL). Yields monitored by quantitative ^{19}F and ^{1}H NMR spectroscopy against a fluorobenzene internal standard.

As ester products were only formed in reactions with $BF_3{\cdot}OEt_2$, controls were performed to ensure their generation was not a result of background reactivity between the benzoic acid product of the shuttle catalysis reaction and $BF_3{\cdot}OEt_2$ (**Scheme S.3.11**).

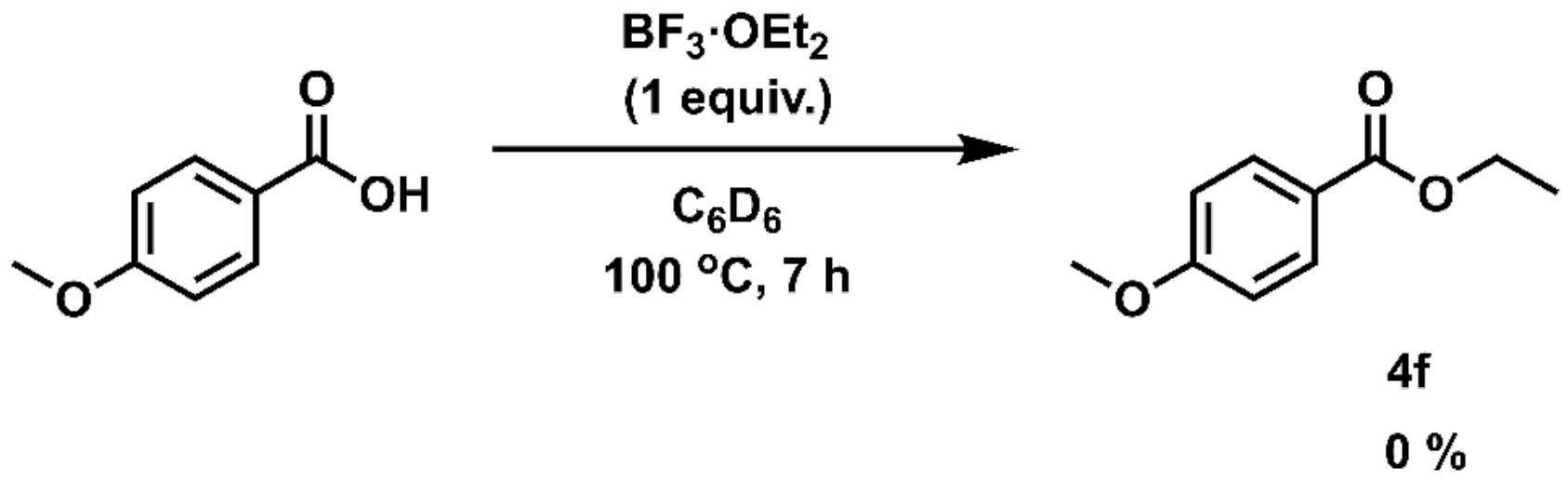

Scheme S.3.11 **a**) Reaction of 4-methoxybenzoic acid (0.38 mmol, 1 equiv.) with $BF_3 \cdot OEt_2$ (0.38 mmol, 1 equiv.) in C_6D_6, showing no ester formation. Yields monitored by quantitative ^1H NMR spectroscopy against a fluorobenzene internal standard.

Ester formation by side reactivity was further investigated by either the reaction between the benzoic acid and ethylene (**Scheme S.3.12**) and the reaction between the benzoyl fluoride with the catalyst, $BF_3 \cdot OEt_2$ (**Scheme S.3.13**).

When benzoic acid, charged under an atmosphere of ethylene, was subjected to catalytic conditions (10 mol% $BF_3 \cdot OEt_2$, 10 mol% $BF_3 \cdot PCy_3$), no reaction was observed and thus, no formation of the ester (**Scheme S.3.12**).

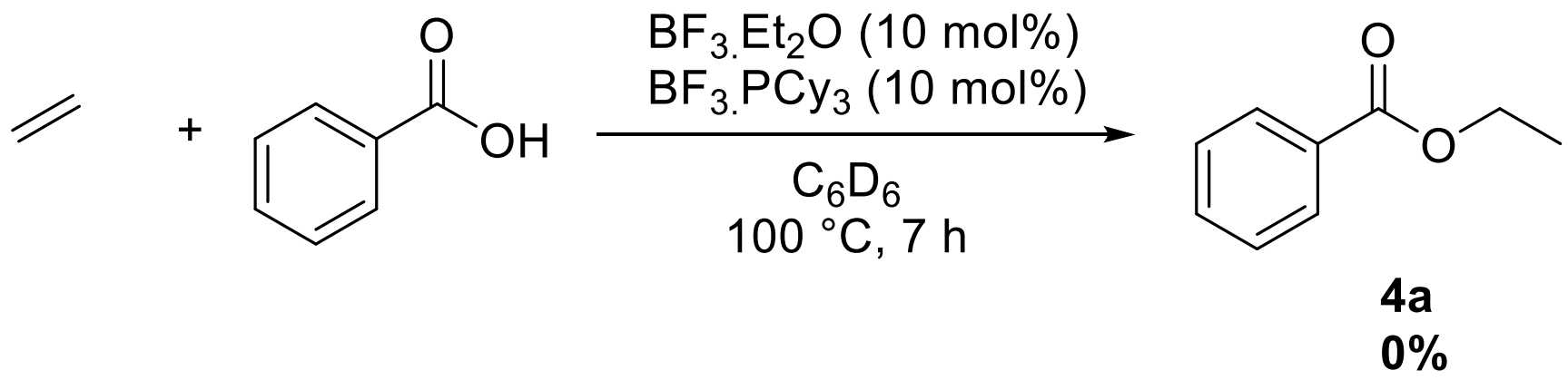

Scheme S.3.12 Reaction of benzoic acid (0.082 mmol, 1 equiv.), ethylene (excess, 1.0 bar), $BF_3 \cdot PCy_3$ (0.008 mmol, 0.1 equiv.) and $BF_3 \cdot OEt_2$ (0.008 mmol, 0.1 equiv.) in C_6D_6, showing no ester formation. Yields monitored by quantitative ^1H NMR spectroscopy against a 1,2-difluorobenzene internal standard.

When benzoyl fluoride was treated with stochiometric quantities of $BF_3 \cdot OEt_2$ and heated at 100 °C for 7 hours, only trace amounts of the ester was observed (< 2%), indicating this was not the primary route for ester formation (**Scheme S.3.13**).

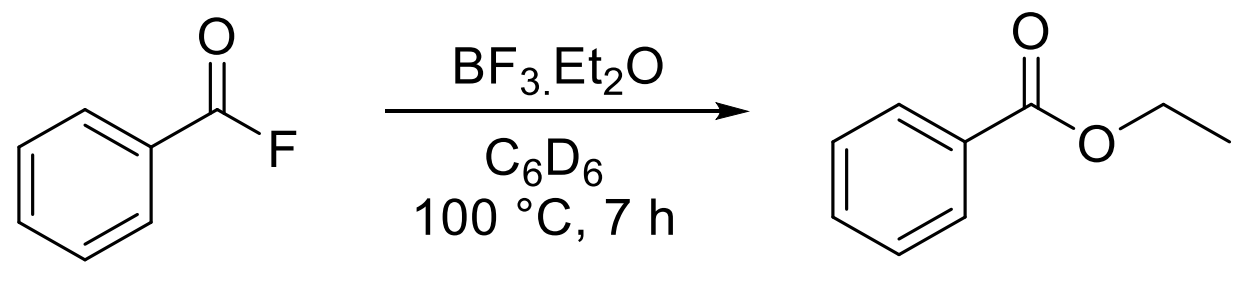

Scheme S.3.13 Reaction of benzoic fluoride (0.09 mmol, 1 equiv.) with $BF_3{\cdot}OEt_2$ (0.09 mmol, 1 equiv.) in C_6D_6, showing no ester formation. Yields monitored by quantitative ^1H NMR spectroscopy against a 1,2-difluorobenzene internal standard.

To support the proposed mechanism the ethyl group within the formed ethyl benzoate could originate from the ethyl group within the catalyst, $BF_3{\cdot}OEt_2$, a control reaction was conducted using 1-fluoroheptane as the fluorine donor (**Scheme S.3.14**). When using 1 equivalent of $BF_3{\cdot}OEt_2$, a mixture of ethyl benzoate and heptyl benzoate was observed.

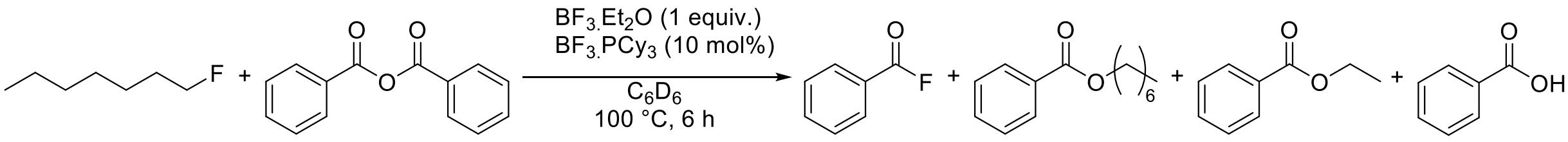

Scheme S.3.14 Control reaction of 1-fluoroheptane (0.19 mmol, 2 equiv.), benzoic anhydride (0.095, 1 equiv.), $BF_3{\cdot}OEt_2$ (0.095 mmol, 1 equiv.) and $BF_3{\cdot}PCy_3$ (0.0095 mmol, 0.1 equiv.) in C_6D_6 (0.5 mL) forming a mixture of ethyl benzoate and heptyl benzoate. Yields monitored by quantitative ^1H NMR spectroscopy against a 1,4-difluorobenzene internal standard.

To test the reactivity of triethyloxonium tetrafluoroborate with benzoic anhydride under the reaction conditions, two controls were performed. The reaction of triethyloxonium tetrafluoroborate with benzoic anhydride generated ethyl benzoate in 28 % yield, with 10 % conversion to **1a** and 58 % **3a** (**Scheme S.3.15.a**). When the same reaction was performed in the presence of $BF_3{\cdot}PCy_3$, 100 % conversion to ethyl benzoate was observed, with a 48 % yield of **1a** (**Scheme S.3.15.b**).

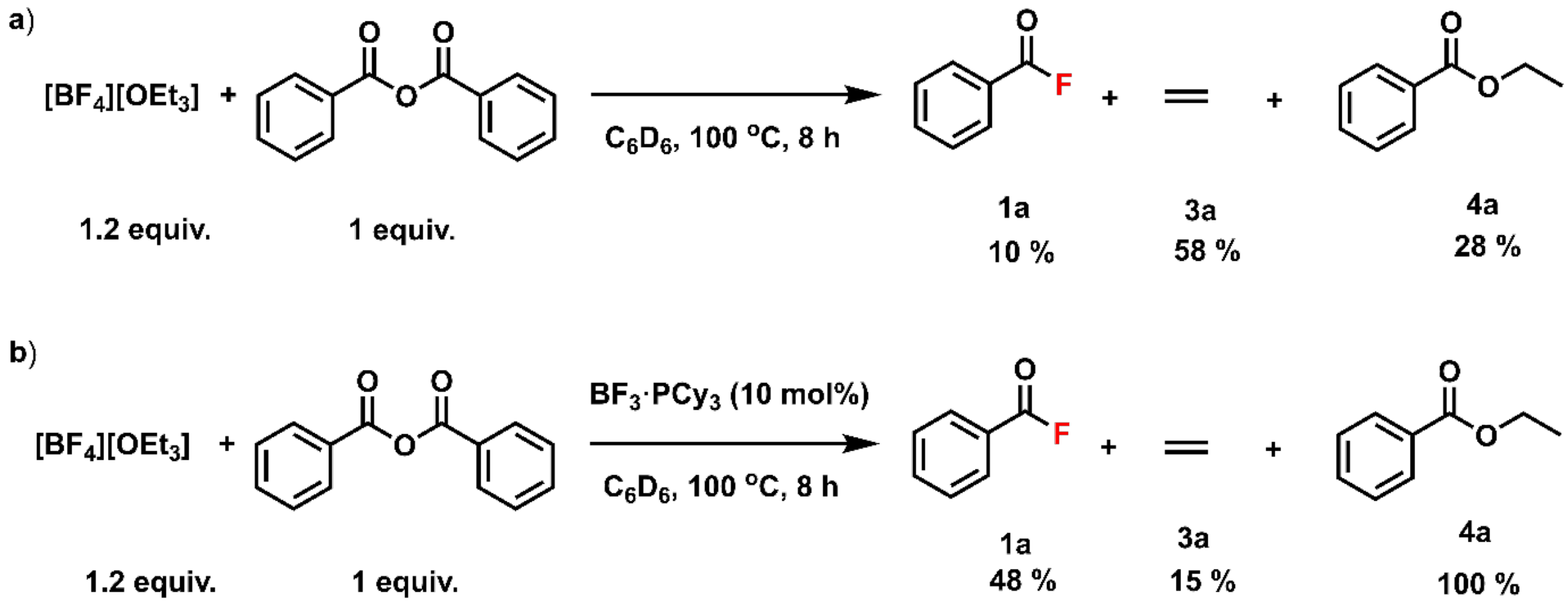

Scheme S.3.15 **a**) Reaction of triethyloxonium tetrafluoroborate with benzoic anhydride to generate a mixture of ethyl benzoate, benzoyl fluoride and ethene in C_6D_6. **b**) Reaction of triethyloxonium tetrafluoroborate with benzoic anhydride in the presence of $BF_3{\cdot}PCy_3$ (10 mol%) generating ethyl benzoate, benzoyl fluoride and ethene. Yields monitored by quantitative ^{1}H and ^{19}F NMR spectroscopy against a fluorobenzene internal standard.

3.7 Shuttle catalysis reactions between fluoropolymers and benzoic anhydride

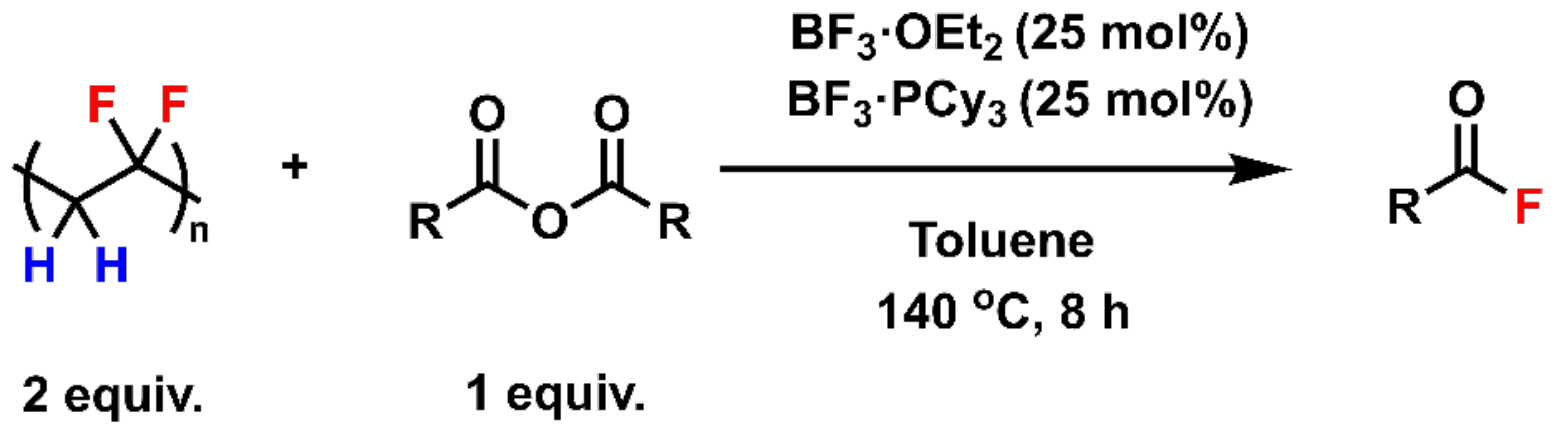

Procedure C

In an N_2 filled glovebox, PVDF (24 mg, 0.384 mmol, 2 equiv. of $C_2F_2H_2$ repeat units), acid anhydride (0.192 mmol, 1 equiv.), $BF_3{\cdot}PCy_3$ (16 mg, 0.048 mmol, 25 mol%), $BF_3{\cdot}OEt_2$ (5.9 µL, 0.048 mmol, 25 mol%), fluorobenzene internal standard (36 µL, 0.384 mmol) and toluene (0.6 mL) were added to a J. Young NMR tube. The reaction mixture was heated in a silicone oil bath at 140 °C for 8 hours, after which time the mixture was removed from the oil bath, allowed to cool to room temperature and ^{19}F NMR spectra were obtained.

3.7.1 Scope in fluoropolymer donors

PVDF molecular weight study

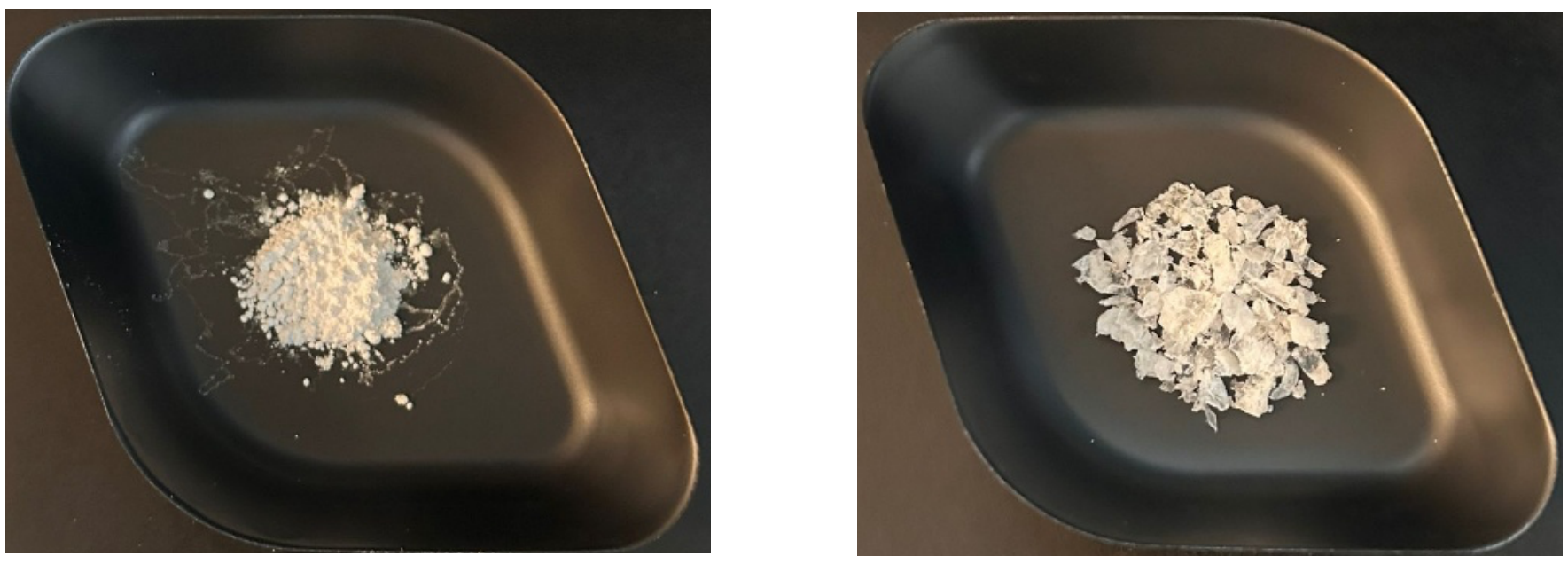

Figure S.3.3 PVDF powder (average M_w = 534,000 g mol $^{-1}$) (left) and PVDF pellets (average M_w = 180,000 g mol $^{-1}$) (right).

Two forms of PVDF were employed in the reaction according to **Procedure A**, with average molecular weight of 534,000 g mol^{-1} (powder) and 180,000 g mol^{-1} (pellets) respectively (**Figure S.3.3**). PVDF pellets (180,000 g mol $^{-1}$) were dissolved in acetonitrile to form a gel, then dried *in vacuo* and crushed into finer pellets with a mortar and pestle prior to use to increase the available surface area. Crushed

pellets were then used according to **Procedure A** (**Table S.3.16**). The resulting polymer product, ***poly-s1***, was characterised by IR spectroscopy (**Figure S.5.5**) and elemental analysis (**Table S.5.5**).

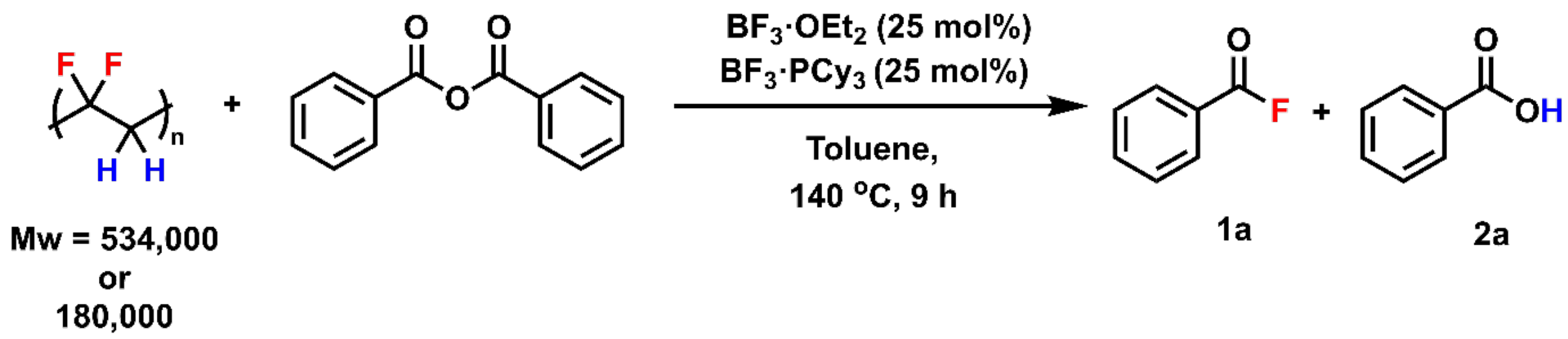

Entry n°.	Molecular weight of PVDF (g mol $^{-1}$)	Product	Yield of **1a** (%)
1	534,000	***poly-1***	**76**
2	180,000	***poly-s1***	**71**

Table S.3.16 Yield of benzoyl fluoride for the reaction of different molecular weights of PVDF (0.38 mmol, 2 equiv.) with benzoic anhydride (0.19 mmol, 1 equiv.) catalysed by $BF_3·OEt_2$ (0.048 mmol, 25 mol%) and $BF_3·PCy_3$. (0.048 mmol, 25mol%) in toluene (0.6 mL). Yields monitored by quantitative ^{19}F NMR spectroscopy against a fluorobenzene internal standard.

The reaction was also performed on a preparative scale using PVDF (average M_w = 534,000 g mol^{-1}). In an N_2 filled glovebox, PVDF (250 mg, 3.90 mmol, 2 equiv. of repeat units $C_2F_2H_2$), benzoic anhydride (450 mg, 1.99 mmol, 1 equiv.), $BF_3·PCy_3$ (173 mg, 0.50 mmol, 25 mol%), $BF_3·OEt_2$ (61 µL, 0.50 mmol, 25 mol%) and toluene (6 mL) were added to a J. Young ampoule. The ampoule was sealed, removed from the glovebox and heated in a silicone oil bath at 140 °C for 8 hours, after which time the mixture was removed from the oil bath and allowed to cool to room temperature. The resulting crude mixture was purified by flash column chromatography (10:1 n-hexane : ethyl acetate) to yield benzoyl fluoride (R_f = 0.6) as a colourless oil (**153 mg, 1.23 mmol, 62 %**). Characterisation data listed below.

PVDF post-consumer material – Li-ion battery material

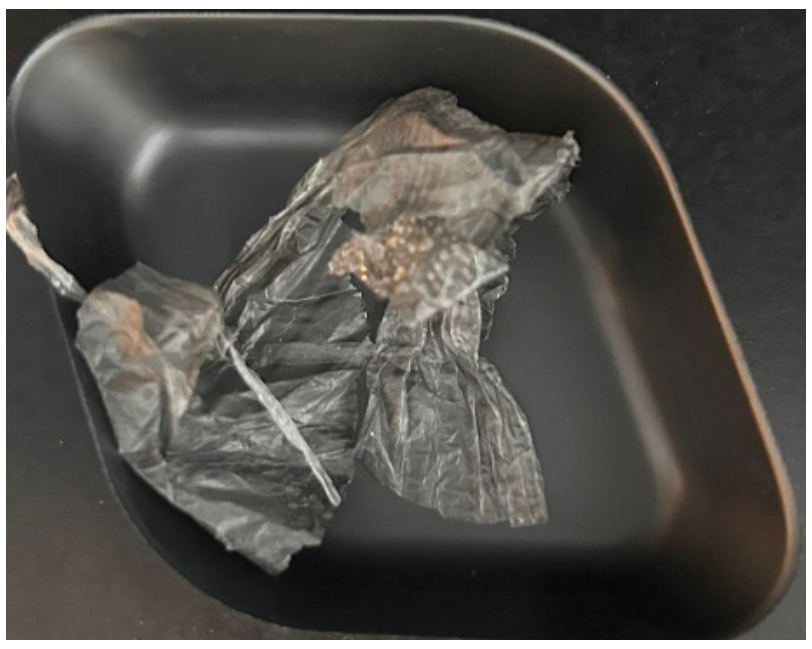

Figure S.3.4 PVDF from a Li-ion battery obtained from ReLiB.

To demonstrate the applicability of the procedure to post-consumer materials of PVDF, a sample of PVDF abstracted from a lithium-ion battery donated by The ReLiB Project (**Figure S.3.4**) in Procedure A with benzoic anhydride (**Scheme S.3.16**).

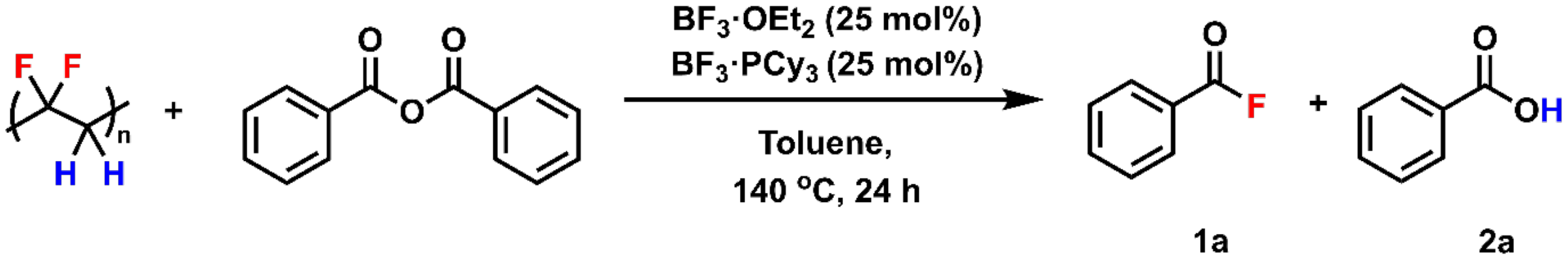

Scheme S.3.16 Reaction of PVDF from a Li-ion battery (0.38 mmol, 2 equiv.) with benzoic anhydride (0.19 mmol, 1 equiv.) in toluene (0.6 mL) to generate benzoyl fluoride, catalysed by BF_3·OEt_2 (0.048 mmol, 25 mol%) and BF_3·PCy_3 (0.048 mmol, 25 mol%) in toluene (0.6 mL). Yields monitored by quantitative ^{19}F NMR spectroscopy against a fluorobenzene internal standard.

PVDF post-consumer material – PVDF tubing

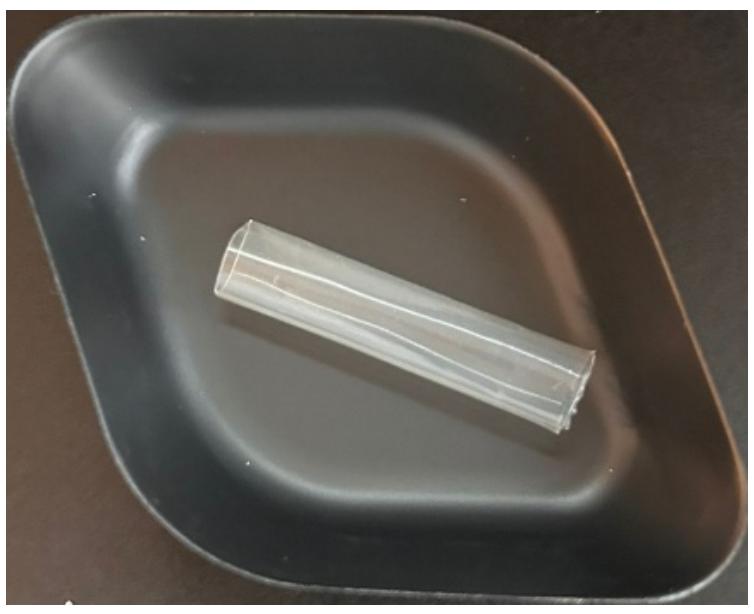

Figure S.3.5 PVDF tubing

A sample of PVDF tubing from Pro Powder (SHF175-6.4MM-1.2M) (**Figure S.3.5**) was used in **Procedure A** with benzoic anhydride (**Scheme S.3.17**). The resulting polymer product, ***poly-s2***, was characterised by IR spectroscopy (**Figure S.5.6**).

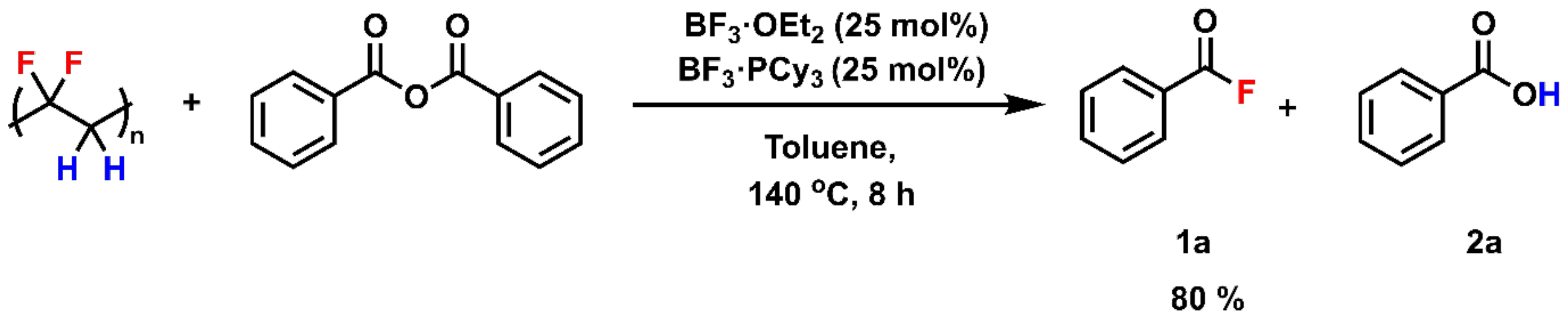

Scheme S.3.17 Reaction of PVDF tubing (0.38 mmol, 2 equiv.) with benzoic anhydride (0.19 mmol, 1 equiv.) in toluene (0.6 mL) to generate benzoyl fluoride, catalysed by $BF_3{\cdot}OEt_2$ (0.048 mmol, 25 mol%) and $BF_3{\cdot}PCy_3$ (0.048 mmol, 25 mol%) in toluene (0.6 mL). Yields monitored by quantitative ^{19}F NMR spectroscopy against a fluorobenzene internal standard.

Poly(vinyl fluoride)

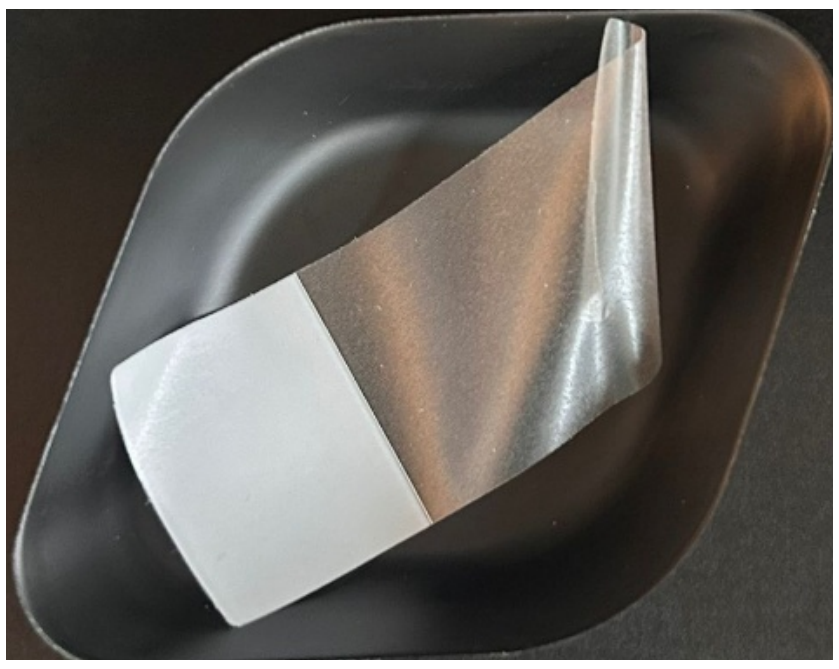

Figure S.3.6 Poly(vinyl fluoride) stickers

Poly(vinyl fluoride) (PVF), the monofluorinated analogue of PVDF, was obtained from Brady Tedlar (M71-21-634/BMP71) as stickers with an adhesive gel backing (**Figure S.3.6**). Prior to use, the PVF stickers were soaked in acetone and wiped clean of the adhesive gel – although this was not necessary for the success of the reaction, performing the reaction with the adhesive gel resulted in more varied yields, likely because of inconsistency in the percentage by weight of poly(vinylidene difluoride) repeat units in the polymer starting material. The PVF stickers also had a paper section, which was removed and not used in the reaction. PVF was then employed according to **Procedure A** for reaction with

benzoic anhydride (**Scheme S.3.18**). The resulting polymer product, ***poly-s3***, was characterised by IR spectroscopy (**Figure S.5.7**).

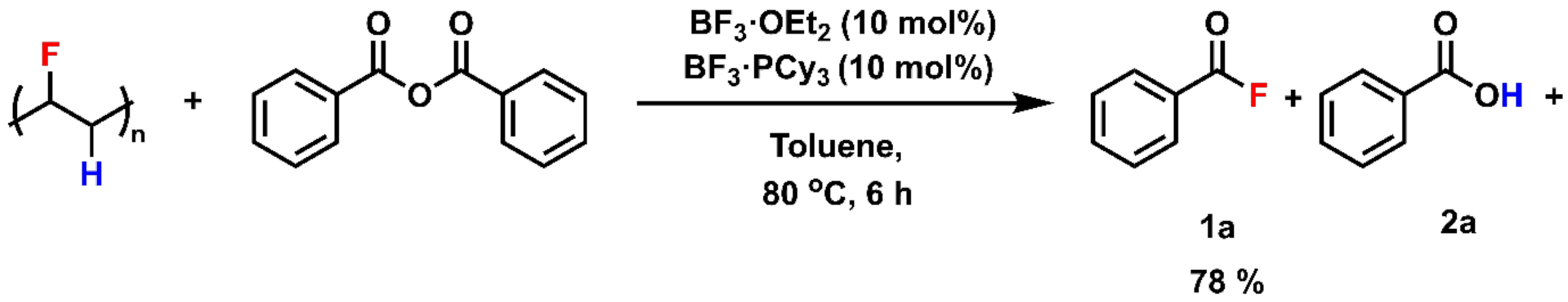

Scheme S.3.18 Reaction of PVF (0.38 mmol, 2 equiv.) with benzoic anhydride (0.19 mmol, 1 equiv.) in toluene (0.6 mL) to yield benzoyl fluoride, catalysed by $BF_3{\cdot}OEt_2$ (0.048 mmol, 25 mol%) and $BF_3{\cdot}PCy_3$ (0.048 mmol, 25 mol%). Yields monitored by quantitative ^{19}F NMR spectroscopy against a fluorobenzene internal standard.

Poly(vinylidene)-co-(hexafluoropropylene)

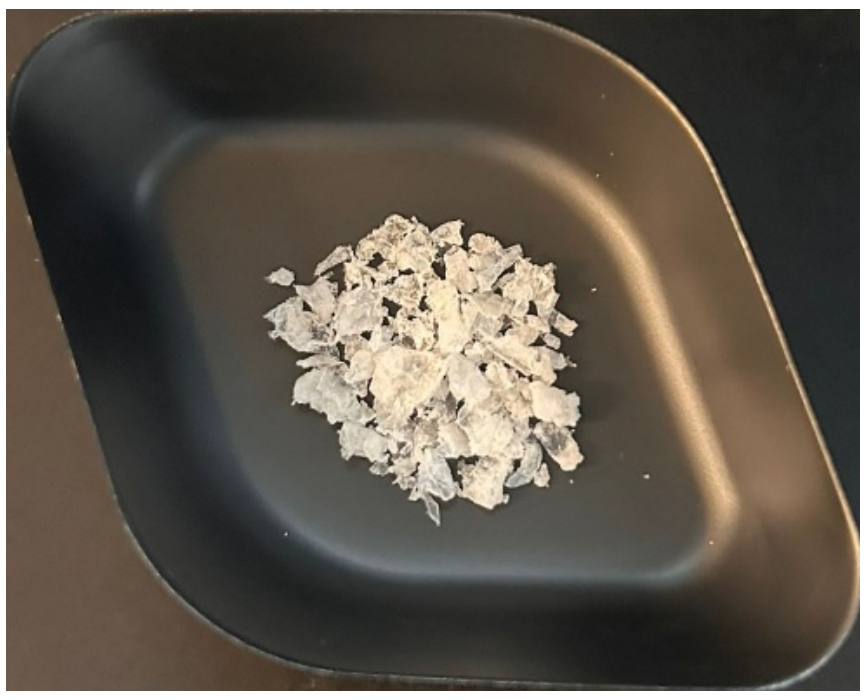

Figure S.3.7 Poly(vinylidene difluoride)-co-(hexafluoropropylene) pellets.

Poly(vinylidene difluoride)-co-(hexafluoropropylene) (PVDF-co-HFP) (average M_w = 400,000 g mol^{-1}) (**Figure S.3.7**) was also used according to **Procedure A** for reaction with benzoic anhydride (**Scheme S.3.19**). PVDF-co-HFP pellets were processed prior to use by dissolution in acetonitrile to form a gel, then dried in vacuo with regular crushing with a pestle and mortar to yield smaller pellets to increase the surface area available for reactivity. The resulting polymer product, ***poly-s4***, was characterised by IR spectroscopy (**Figure S.5.8**).

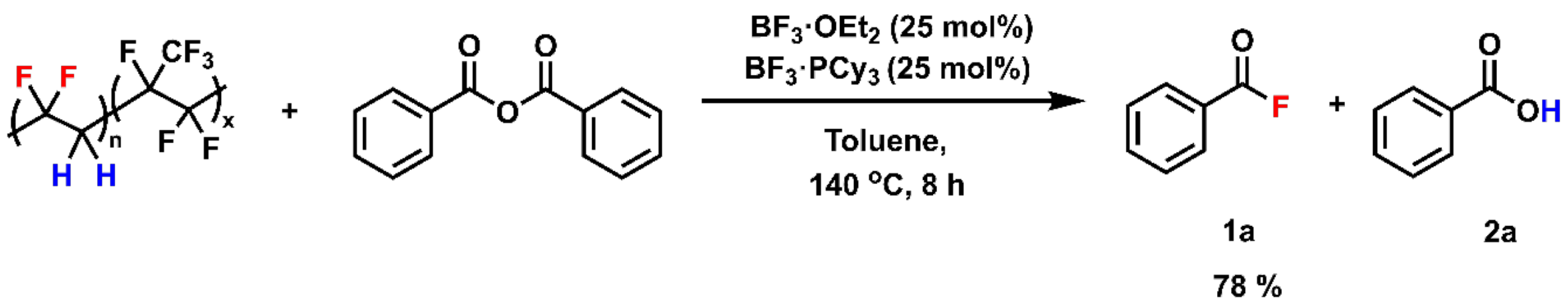

Scheme S.3.19 Reaction of PVDF-co-HFP (0.37 mmol, 1.9 equiv.) with benzoic anhydride (0.19 mmol, 1 equiv.) in toluene (0.6 mL) to generate benzoyl fluoride, catalysed by BF·OEt_2 (0.048 mmol, 25 mol%) and BF_3·PCy_3 (0.048 mmol, 25 mol%). Yields monitored by quantitative ^{19}F NMR spectroscopy against a fluorobenzene internal standard.

^{19}F NMR spectroscopy of the pellets prior to use indicated an approximate ratio of vinylidene difluoride to hexafluoropropylene (HFP) repeat units of 24:1 (**Figure S.3.8**). Consistent with literature, these are largely in the head-to-tail configuration with vinylidene difluoride units.[15] Repeat units of HFP consist of $F_2C=C(CF_3)F$; as they contain no hydrogen atoms, they are unable to act as a source of HF without abstracting a H from a neighbouring vinylidene difluoride repeat unit.

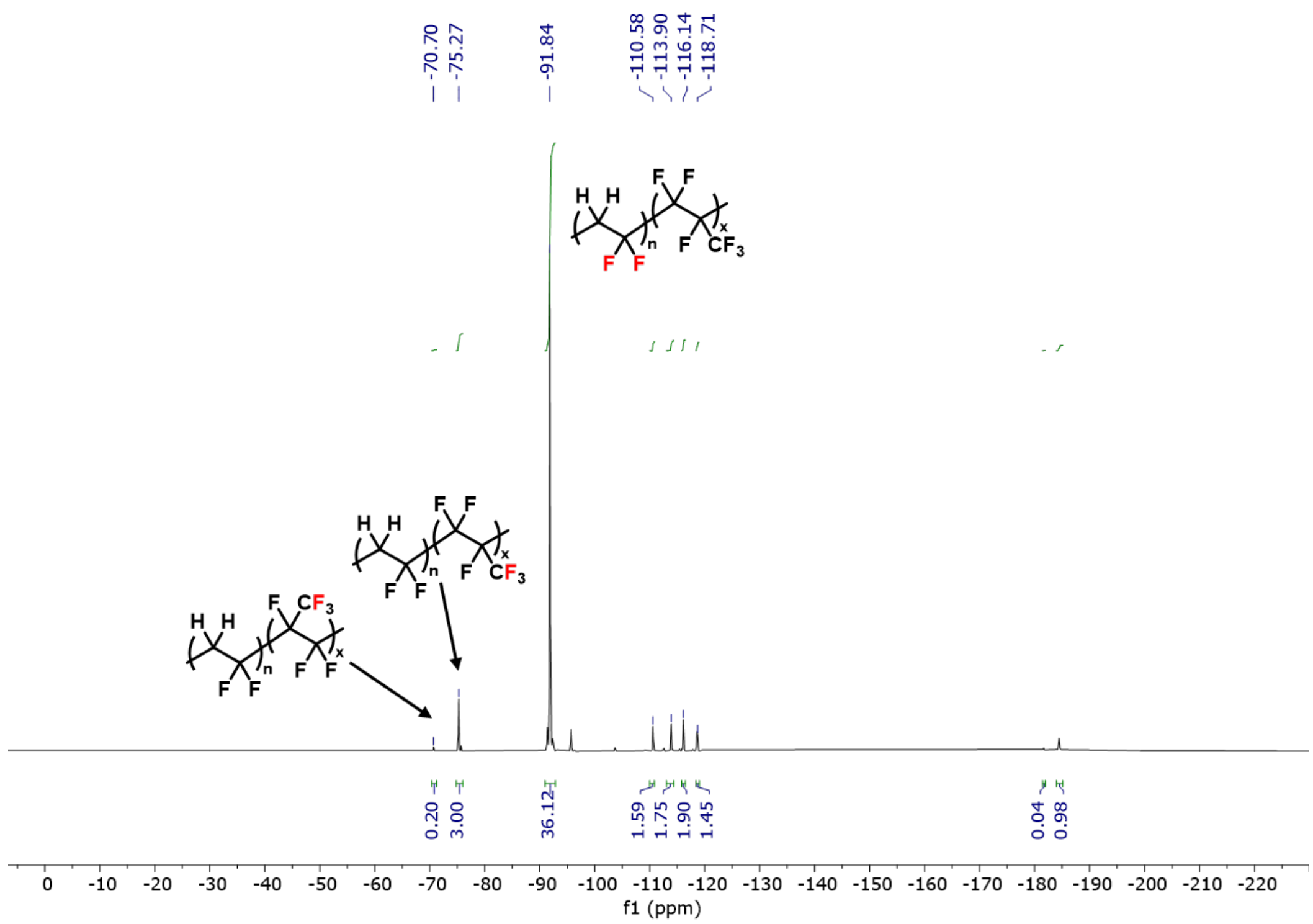

Figure S.3.8 Quantitative ^{19}F spectrum NMR of PVDF-co-HFP in acetonitrile-d_3, showing the ratio of vinylidene difluoride to hexafluoropropylene repeat units, and the configuration of HFP repeat units in relation to the PVDF repeat units as largely in a head-to-tail configuration.

Ethylene tetrafluoroethylene

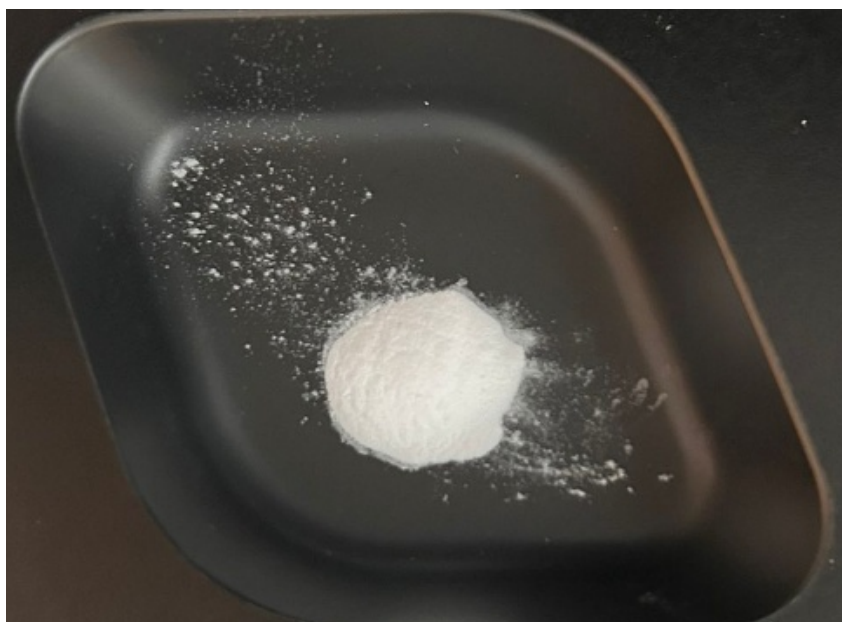

Figure S.3.9 Ethylene tetrafluoroethylene powder obtained from AGC chemicals.

Ethylene tetrafluoroethylene (ETFE) powder obtained from AGC Chemicals (**Figure S.3.9**) was used without further processing according to **Procedure A** with benzoic anhydride (**Scheme S.3.20**).

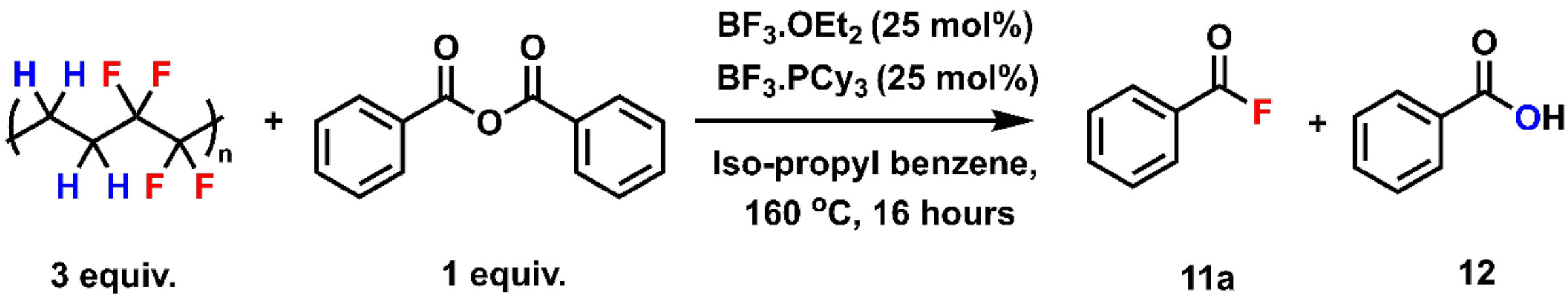

Scheme S.3.20 Reaction of ETFE (0.95 mmol, 5 equiv.) with benzoic anhydride (0.19 mmol, 1 equiv.) in toluene (0.6 mL) to yield benzoyl fluoride, catalysed by $BF_3{\cdot}OEt_2$ (0.048 mmol, 25 mol%) and $BF_3{\cdot}PCy_3$ (0.048 mmol, 25 mol%). Yields monitored by quantitative ^{19}F NMR spectroscopy against a fluorobenzene internal standard.

3.7.2 Successive defluorination trials

Successive defluorination trials were performed on the same sample of PVDF, as per the procedure described above. At the end of each reaction, the reaction mixture was decanted away from the solid polymeric material which was washed with diethyl ether (3 x 10 mL), then dried under vacuum before being resubjected to the reaction conditions. On each successive trial, the yield of **1a** decreased significantly (**Table S.3.17**).

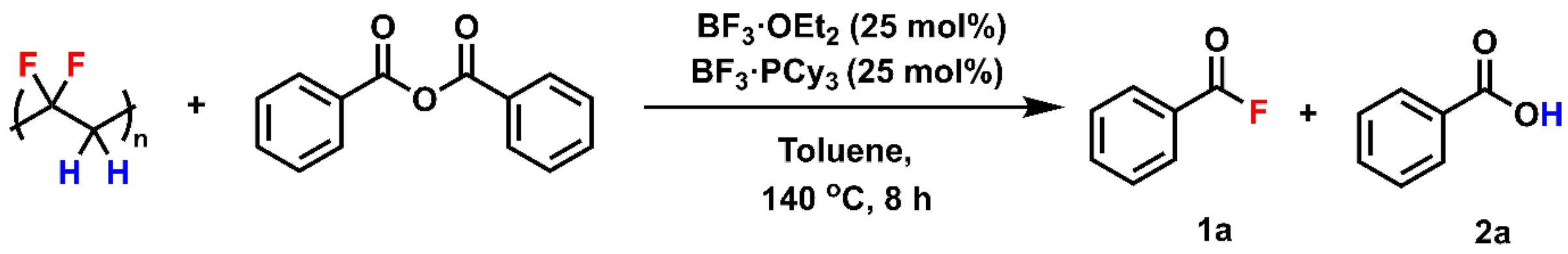

Defluorination cycle	Yield of **1a** (%)
1st	**70**
2nd	**9**
3rd	**4**

Table S.3.17 Yield of benzoyl fluoride for the successive defluorination trial of reaction of the same sample of PVDF (0.38 mmol, 2 equiv.) with benzoic anhydride (0.19 mmol, 1 equiv.) when resubjected to reaction conditions. Yields monitored by quantitative ^{19}F NMR spectroscopy against a fluorobenzene internal standard.

3.7.3 Further fluorine recycling of *poly-1* and *poly-2* by fluorine mineralisation

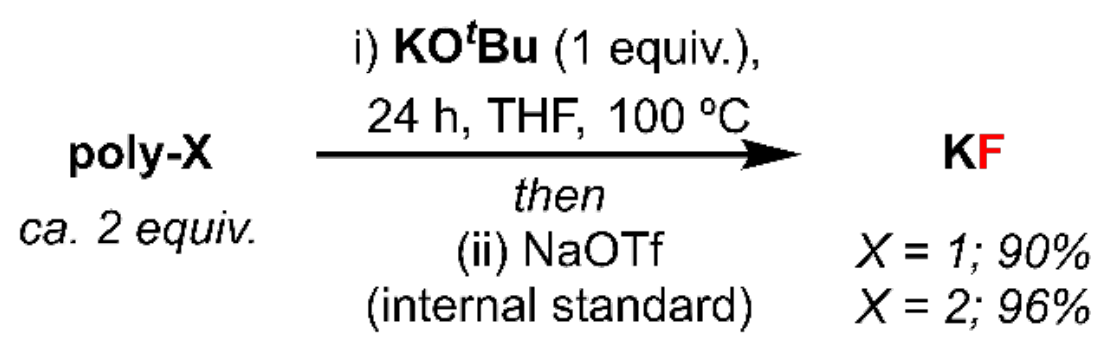

In a N_2 glovebox, ***poly-X*** (10 mg) was weighed in a 1 mL vial and transferred to a J. Young's NMR tube using THF (3 X 100 μL). In another 1 mL vial, KO^{t}Bu (8.8 mg, 0.078 mmol) was weighed out and transferred to the J. Young's NMR tube using THF (3 X 100 μL). The NMR tube before sealed and inverted 5 times to insure thorough mixing. The NMR tube was heated to 100 °C for 24 h. In air, NaOTf (12.2 mg, 0.078 mmol) was weighed out and transferred into the NMR tube using 1 mL of H_2O and mixed thoroughly. A ^{19}F NMR spectrum (D_1 = 55 s) was measured to determine the yield of KF produced compared the internal standard NaOTf.

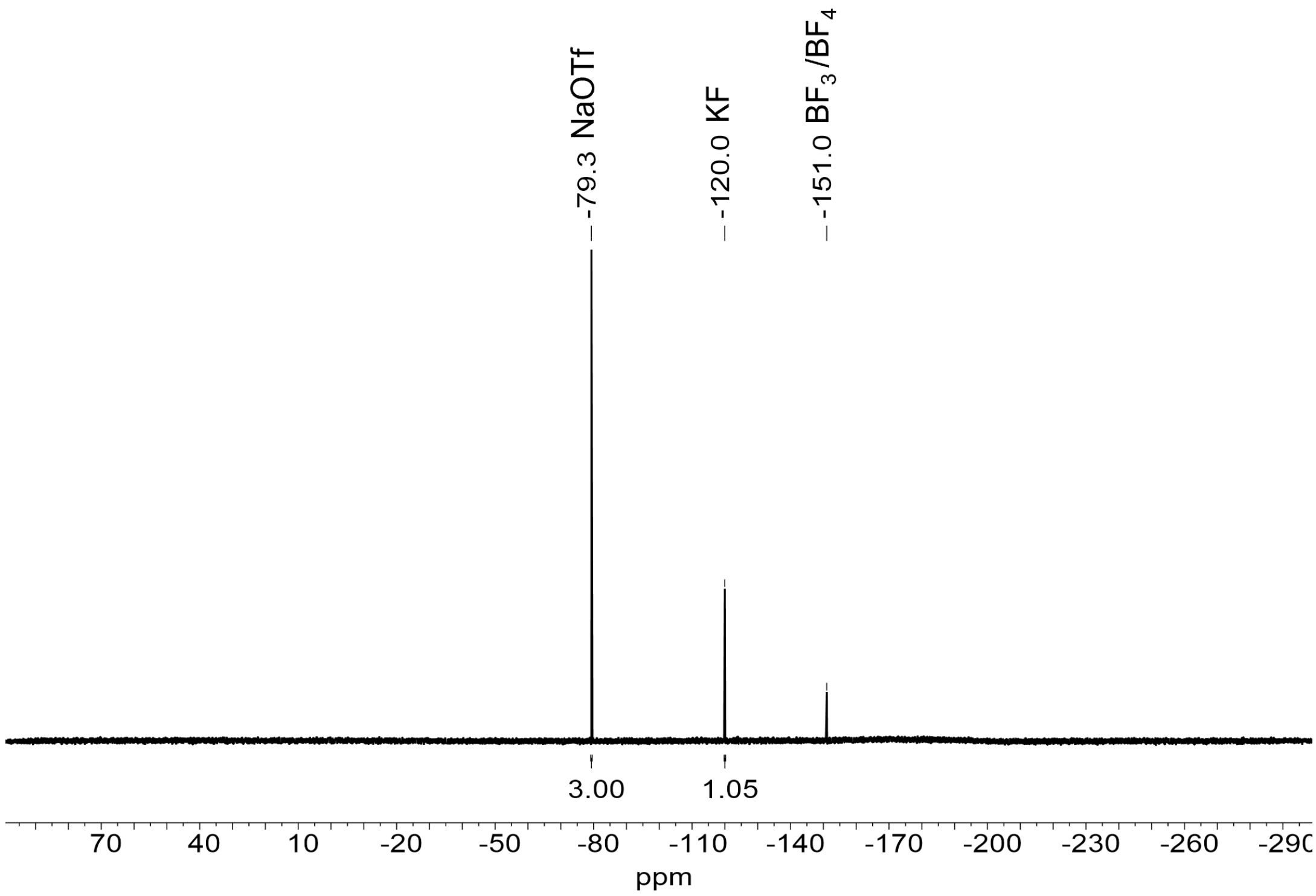

Figure S.3.10 ^{19}F NMR spectrum of fluorination of KF after 24 hours activation of **poly-1** at 100 °C (377 MHz, THF-h_8 + H_2O, 25 °C). 0.16 mmol **poly-1** and 0.078 mmol KO^{t}Bu led to the formation of 0.072 mmol KF. Internal standard NaOTf (10.7 mg, 0.069 mmol).

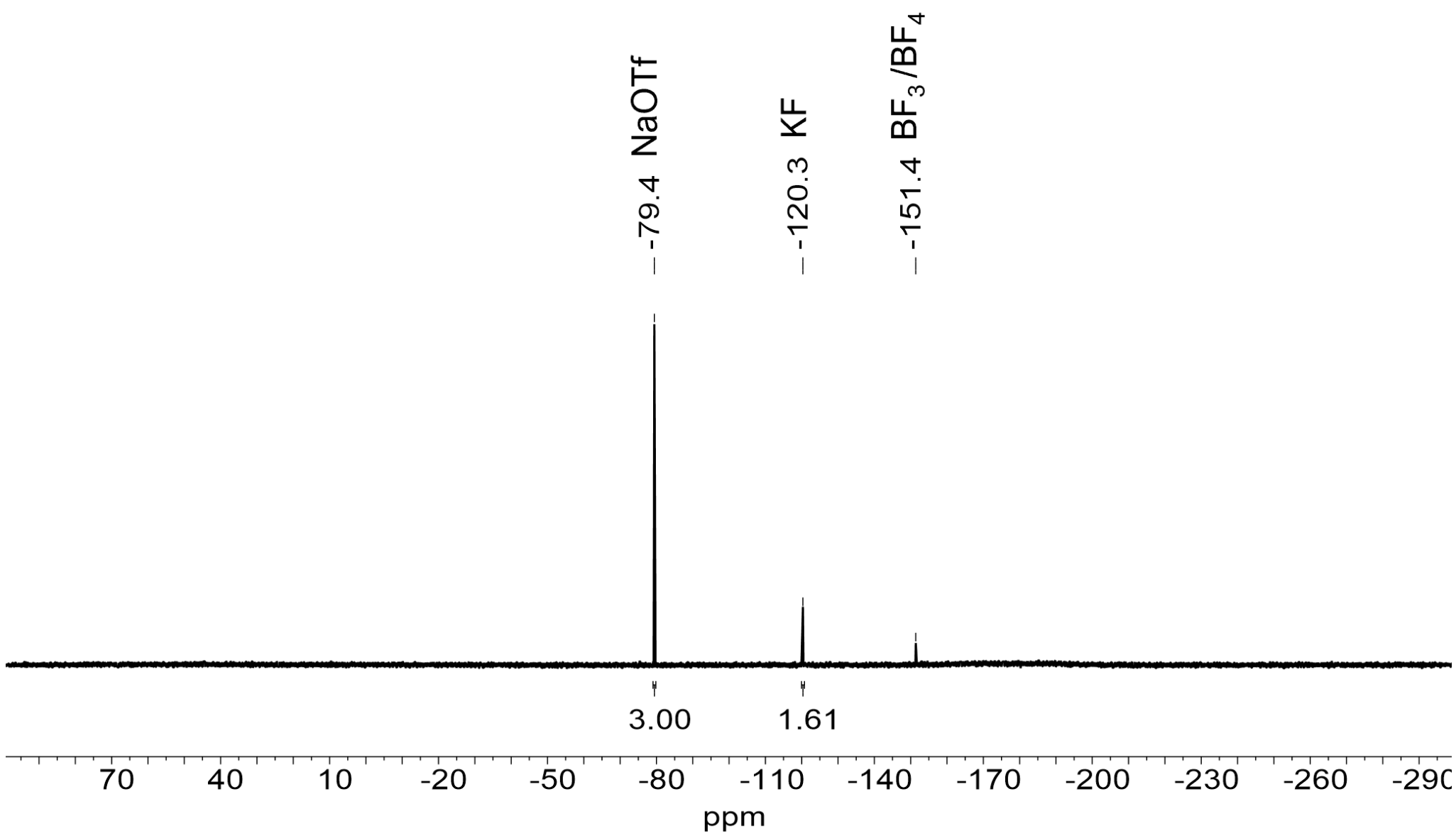

Figure S.3.11 ^{19}F NMR spectrum of fluorination of KF after 24 hours activation of **poly-2** at 100 °C (377 MHz, THF-h_8 + H_2O, 25 °C). 0.23 mmol **poly-2** and 0.078 mmol KO^{t}Bu led to the formation of 0.075 mmol KF. Internal standard NaOTf (7.3 mg, 0.047 mmol).

3.7.4 Further fluorine recycling of *poly-1* and *poly-2* by fluorine transfer

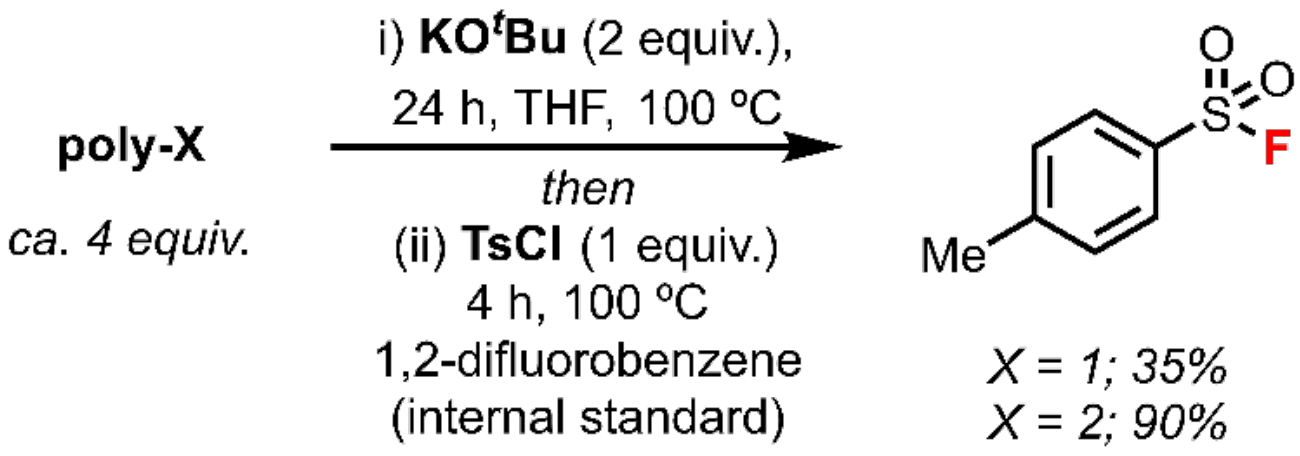

In a N_2 glovebox, ***poly-X*** (10 mg) was weighed in a 1 mL vial and transferred to a J. Young's NMR tube using THF (3 X 100 µL). In another 1 mL vial, KO^{t}Bu (8.8 mg, 0.078 mmol) was weighed out and transferred to the J. Young's NMR tube using THF (3 X 100 µL). The NMR tube before sealed and inverted 5 times to insure thorough mixing. The NMR tube heated to 100 °C for 24 h. TsCl (7.4 mg, 0.039 mmol) was weighed out in a 1 mL vial and transferred into the NMR tube with THF (3 x 50 µL) and 1,2-DFB (8 µL) was added to the NMR tube by micropipette. The NMR tube was sealed and heated at 100 °C for 4 h. A ^{19}F NMR spectrum (D_1 = 55 s) was measured to determine the yield of TsF produced compared the internal standard 1,2-difluorobenzene (1,2-DFB).

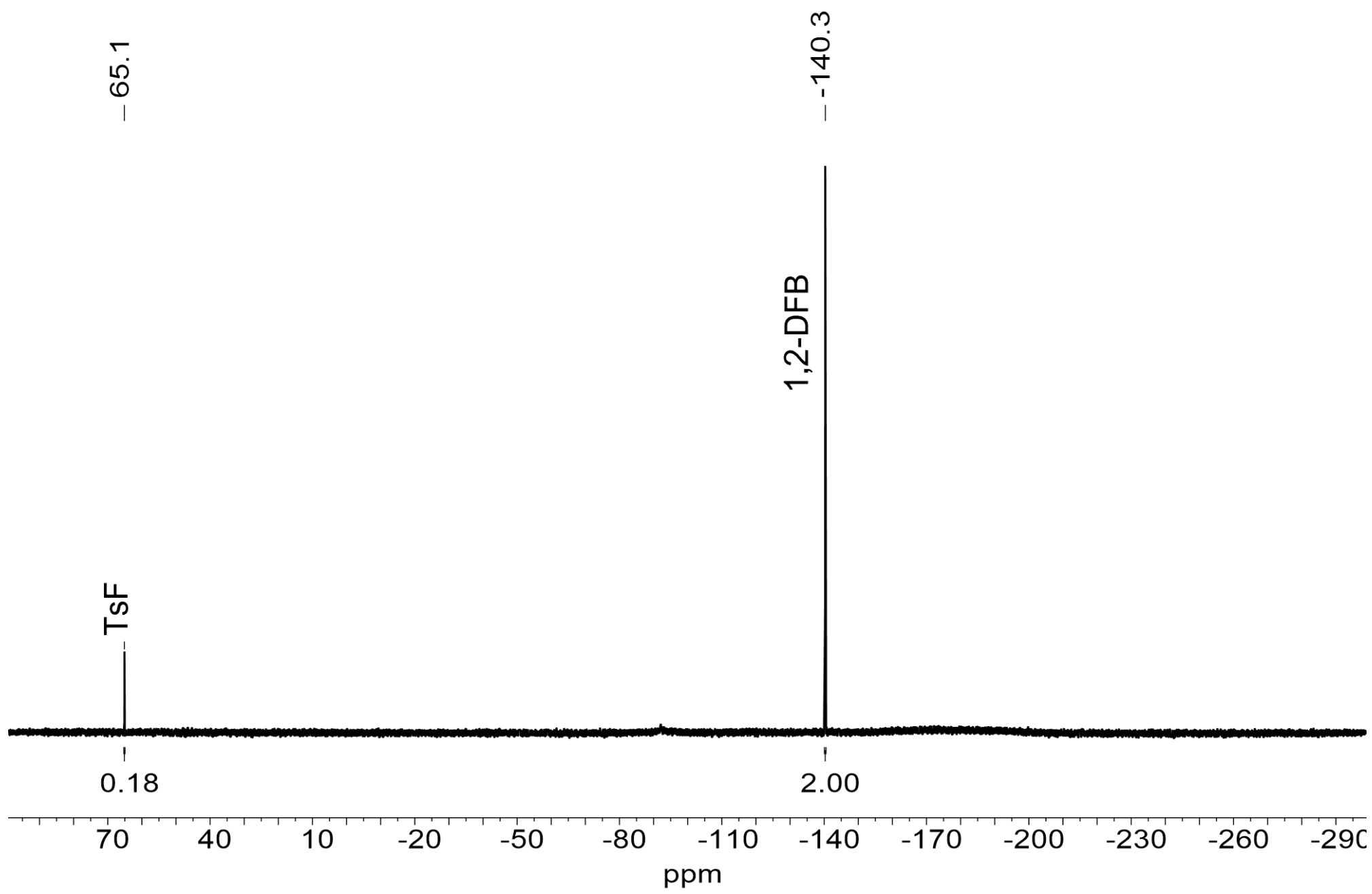

Figure S.3.12 ^{19}F NMR spectrum of fluorination of TsCl after 24 hours activation of **poly-1** at 100 °C and 4 hours fluorination of TsCl at 100 °C (377 MHz, THF-h_8, 25 °C). 0.16 mmol **poly-1**, 0.078 mmol KO^{t}Bu and 0.04 mmol TsCl led to the formation of 0.013 mmol TsF. Internal standard 1,2-DFB (8.5 mg, 0.075 mmol)

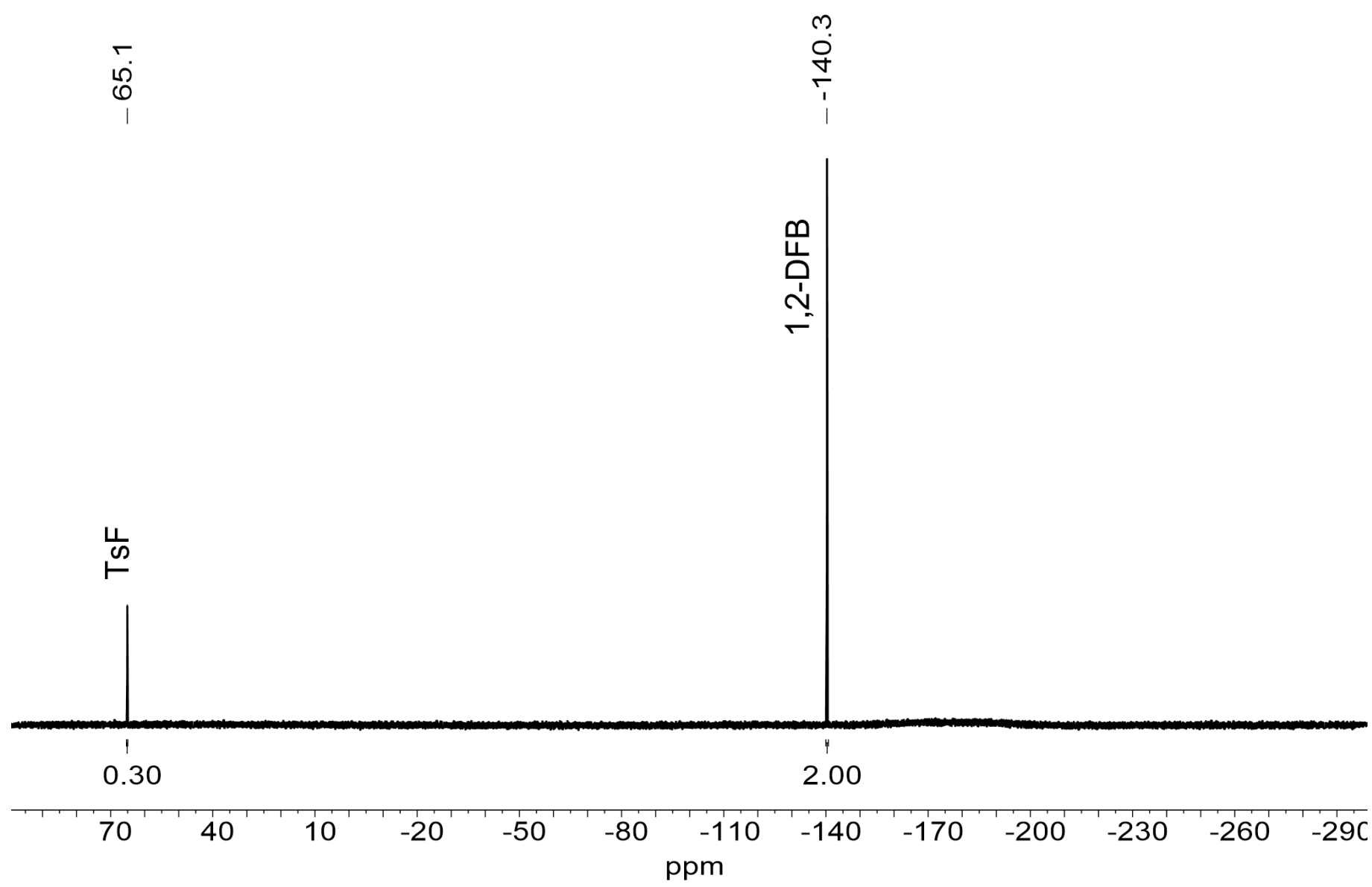

Figure S.3.13 ^{19}F NMR spectrum of fluorination of TsCl after 24 hours activation of **poly-2** at 100 °C and 4 hours fluorination of TsCl at 100 °C (377 MHz, THF-h_8, 25 °C). 0.23 mmol **poly-2**, 0.078 mmol KO^{t}Bu and 0.04 mmol TsCl led to the formation of 0.036 mmol TsF. Internal standard 1,2-DFB (13.5 mg, 0.118 mmol).

3.7.5 Shuttle catalysis reactions between fluoropolymers and alkynes

To show the previously reported scope of acceptors the shuttle catalysis methodology could be accessed using a range of fluoropolymer HF donors, alkynes were used as acceptors for all fluoropolymer donors according to **Procedure B** (**Scheme S.3.21**).

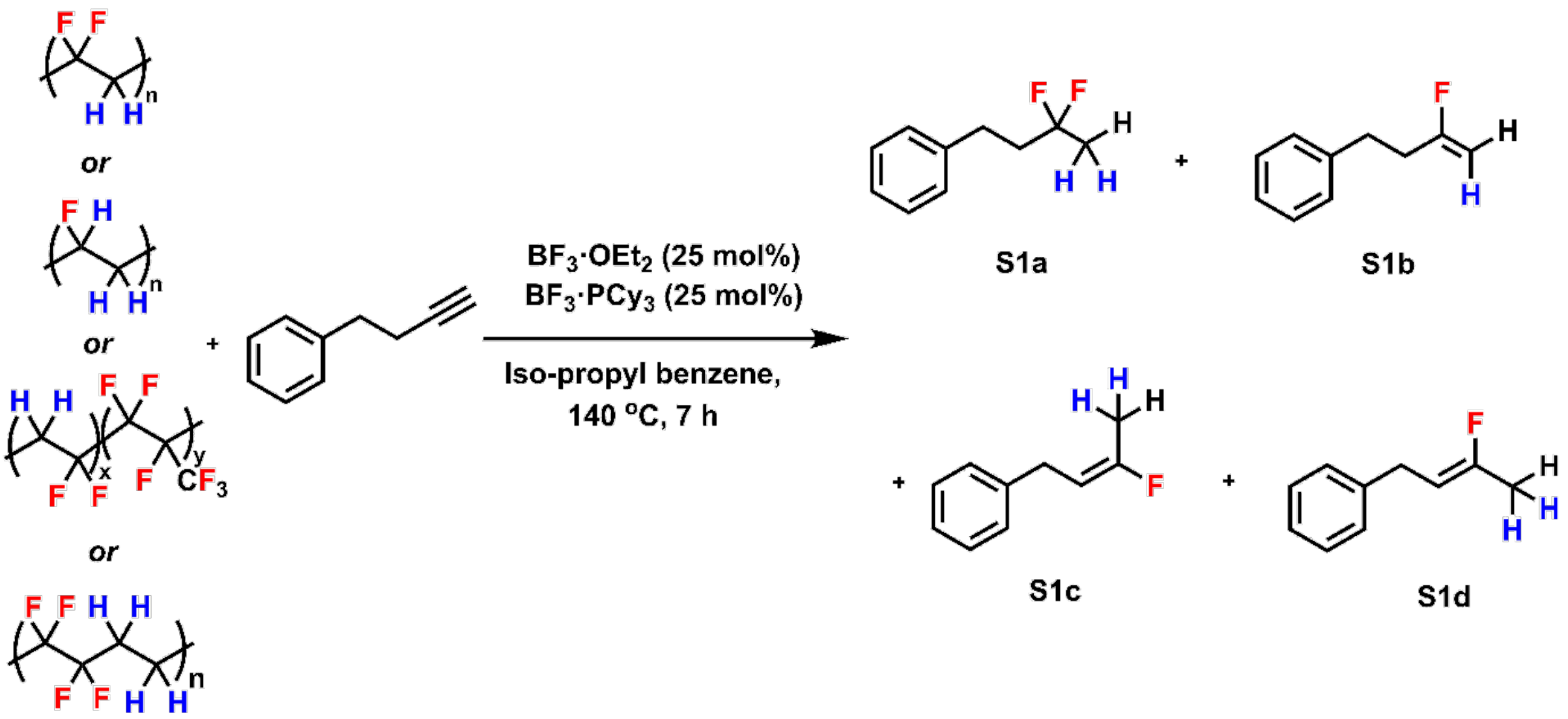

Scheme S.3.21. Reaction of different fluoropolymers (0.38 mmol, 2 equiv.) with 4-phenyl-1-butyne (0.19 mmol, 1 equiv.) generating a mixture of fluoroalkane and fluoroalkene products catalysed by $BF_3 \cdot OEt_2$ (0.048 mmol, 25 mol%) and $BF_3 \cdot PCy_3$ (0.048 mmol, 25 mol%) in iso-propyl benzene (0.6 mL). Yields monitored by quantitative ^{19}F NMR spectroscopy against a fluorobenzene internal standard.

Procedure D

In an N_2 filled glovebox, PVDF (24 mg, 0.384 mmol, 2 equiv. of $C_2F_2H_2$ repeat units), 4-phenyl-1-butyne (27 µL, 0.192 mmol, 1 equiv.), $BF_3 \cdot PCy_3$ (16 mg, 0.048 mmol, 25 mol%), $BF_3 \cdot OEt_2$ (6 µL, 0.048 mmol, 25 mol%), fluorobenzene internal standard (36 µL, 0.384 mmol) and iso-propyl benzene (0.3 mL) were added to a J. Young NMR tube. The reaction mixture was heated in a silicone oil bath at 140 °C for 8 hours, after which time the mixture was removed from the oil bath, allowed to cool to room temperature and ^{19}F NMR spectra were obtained.

Entry n°.	Polymer	Product	Yield of **1a** (%)	Yield of **1b** (%)	Yield of **1c** (%)	Yield of **1d** (%)	Total yield **1a** + **1b** + **1c** + **1d** (%)
1	PVDF (M_W=534,000)	***poly-2***	27	4	14	36	**81**
2	PVDF (M_W=180,000)	***poly-s5***	36	2	5	18	**64**
3	PVDF tubing	***poly-s6***	55	2	30	12	**99**
5	PVF	***poly-s7***	47 – 63	0 - 8	0 – 6	10 – 21	**57 - 98**
4	PVDF-co-HFP	***poly-s8***	10	8	14	38	**70**

Table S.3.18 Yield of a mixture of fluoroalkanes and fluoroalkenes from the reaction of different fluoropolymers (0.38 mmol, 2 equiv.) with 4-phenyl-1-butyne (0.19 mmol, 1 equiv.) $BF_3{\cdot}OEt_2$ (0.048 mmol, 25 mol%) and $BF_3{\cdot}PCy_3$ (0.048 mmol, 25 mol%) in iso-propyl benzene (0.3 mL) according to **Procedure D**. Yields monitored by quantitative ^{19}F NMR spectroscopy against a fluorobenzene internal standard.

4 NMR spectra

Figure S.4.1 ^{1}H NMR spectrum of 2,4,6-trimethylbenzoic anhydride.

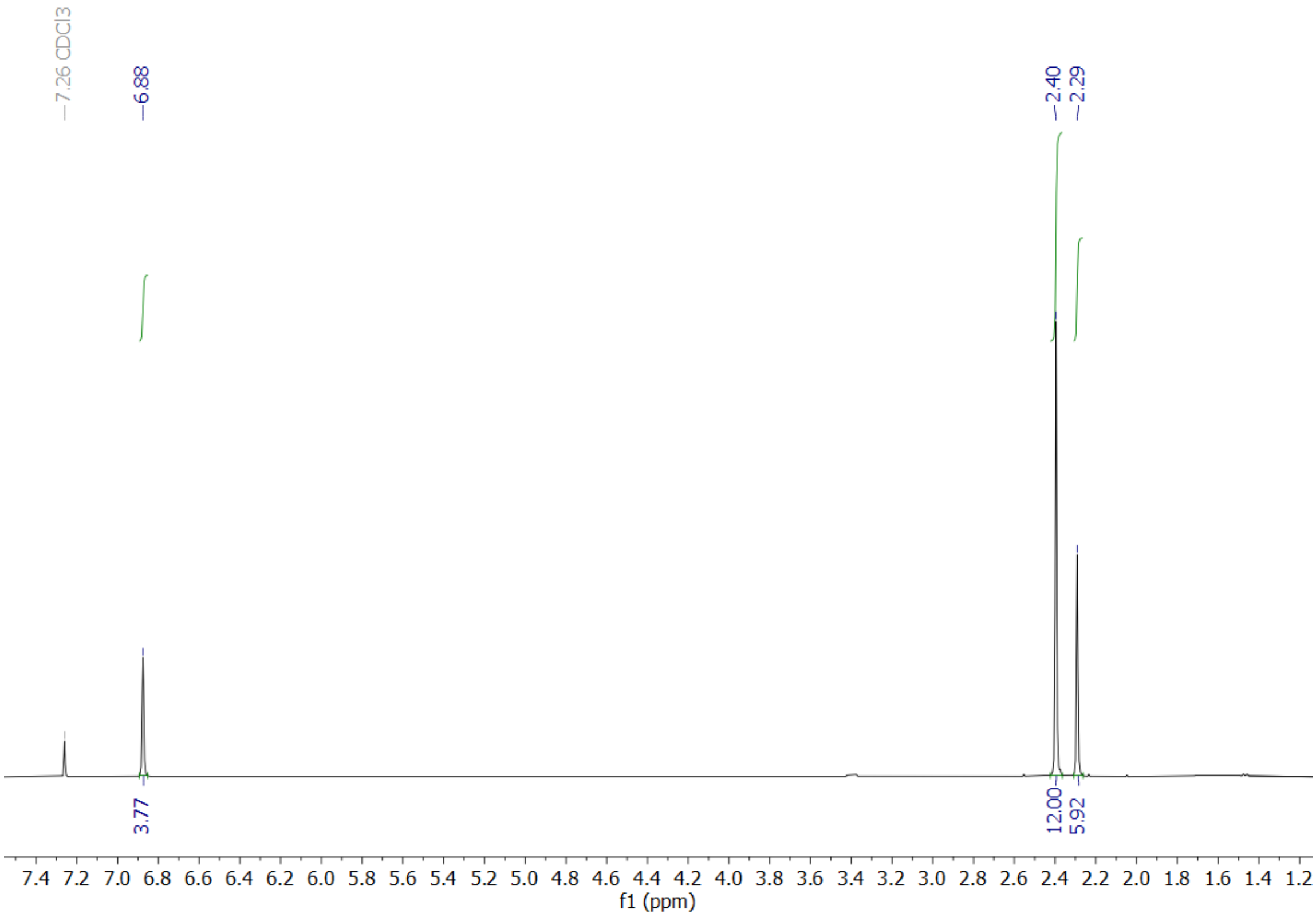

Figure S.4.2 $^{13}C\{^{1}H\}$ NMR spectrum of 2,4,6-trimethylbenzoic anhydride.

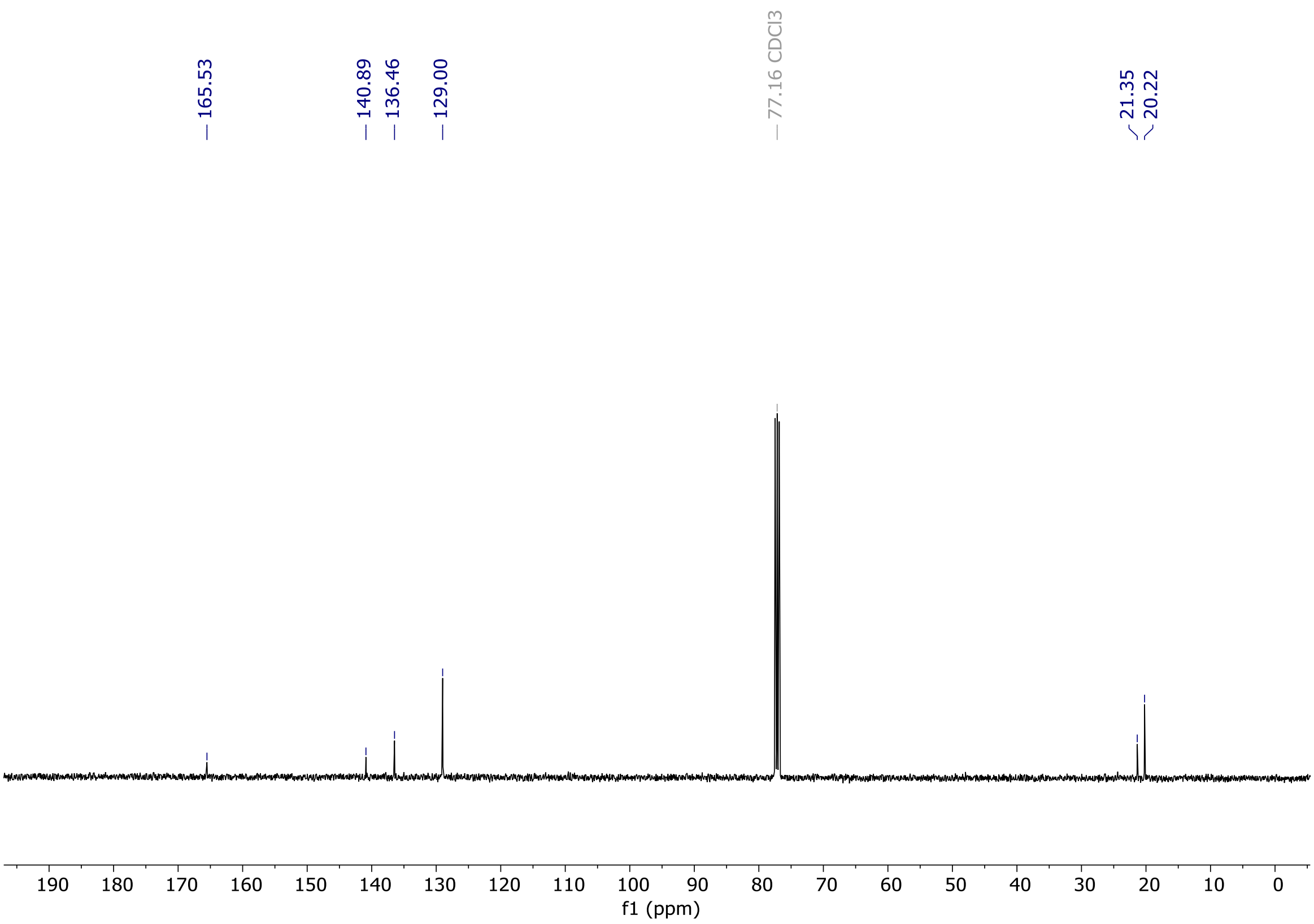

Figure S.4.3 ^1H NMR spectrum of 4-bromobenzoic anhydride.

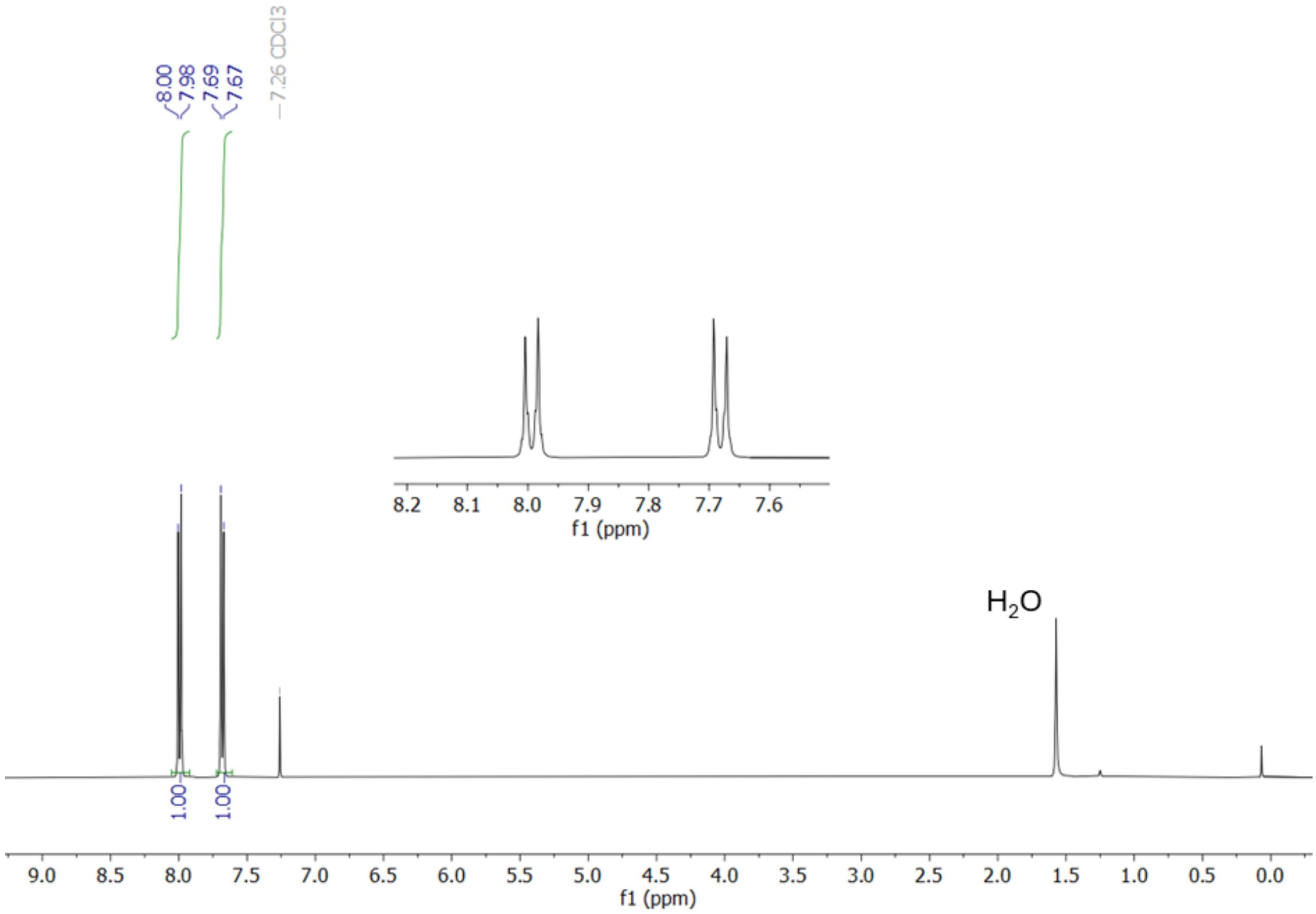

Figure S.4.4 $^{13}C\{^1H\}$ spectrum NMR of 4-bromobenzoic anhydride.

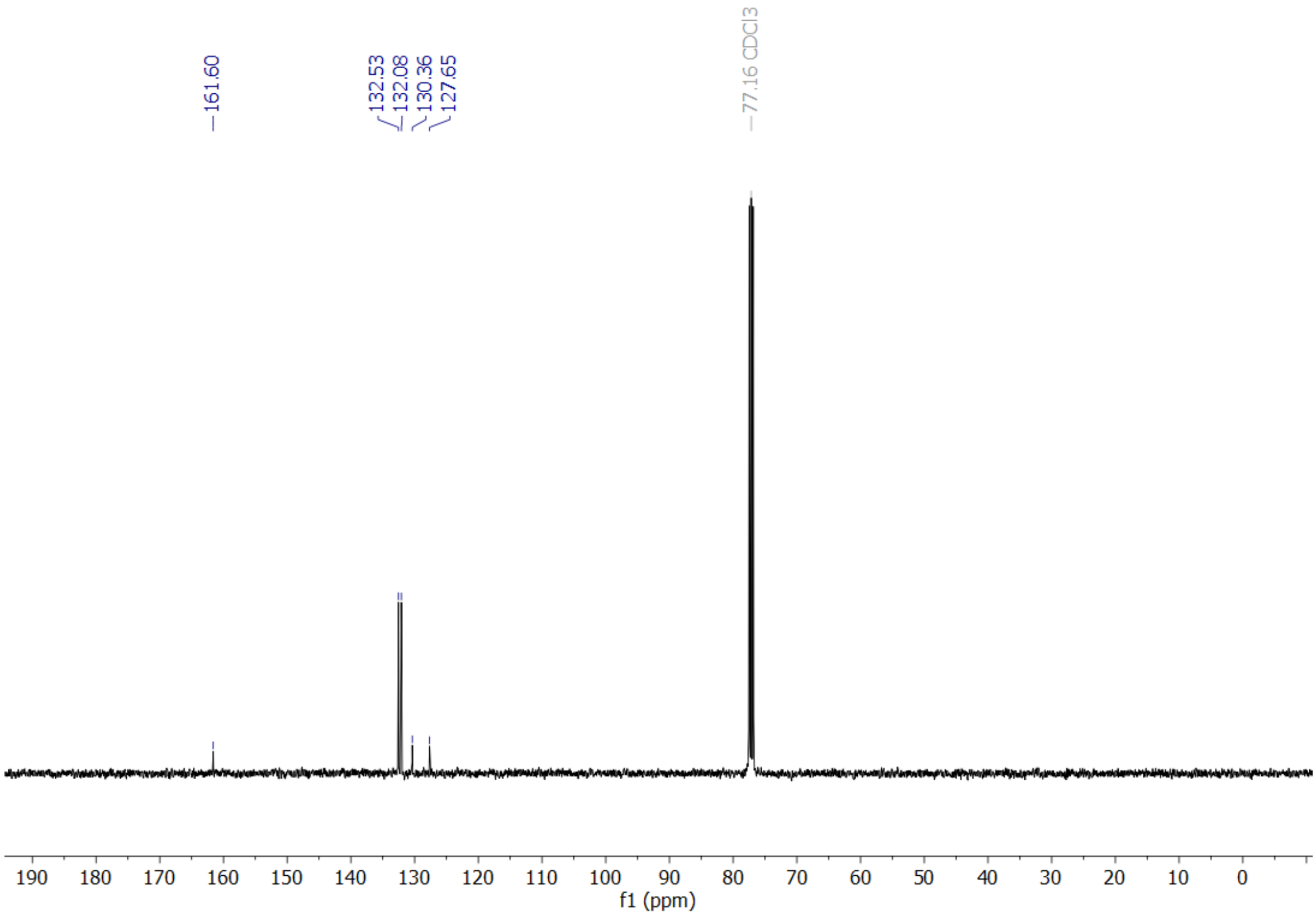

Figure S.4.5 ^{1}H spectrum NMR of 2,6-difluorobenzoic anhydride.

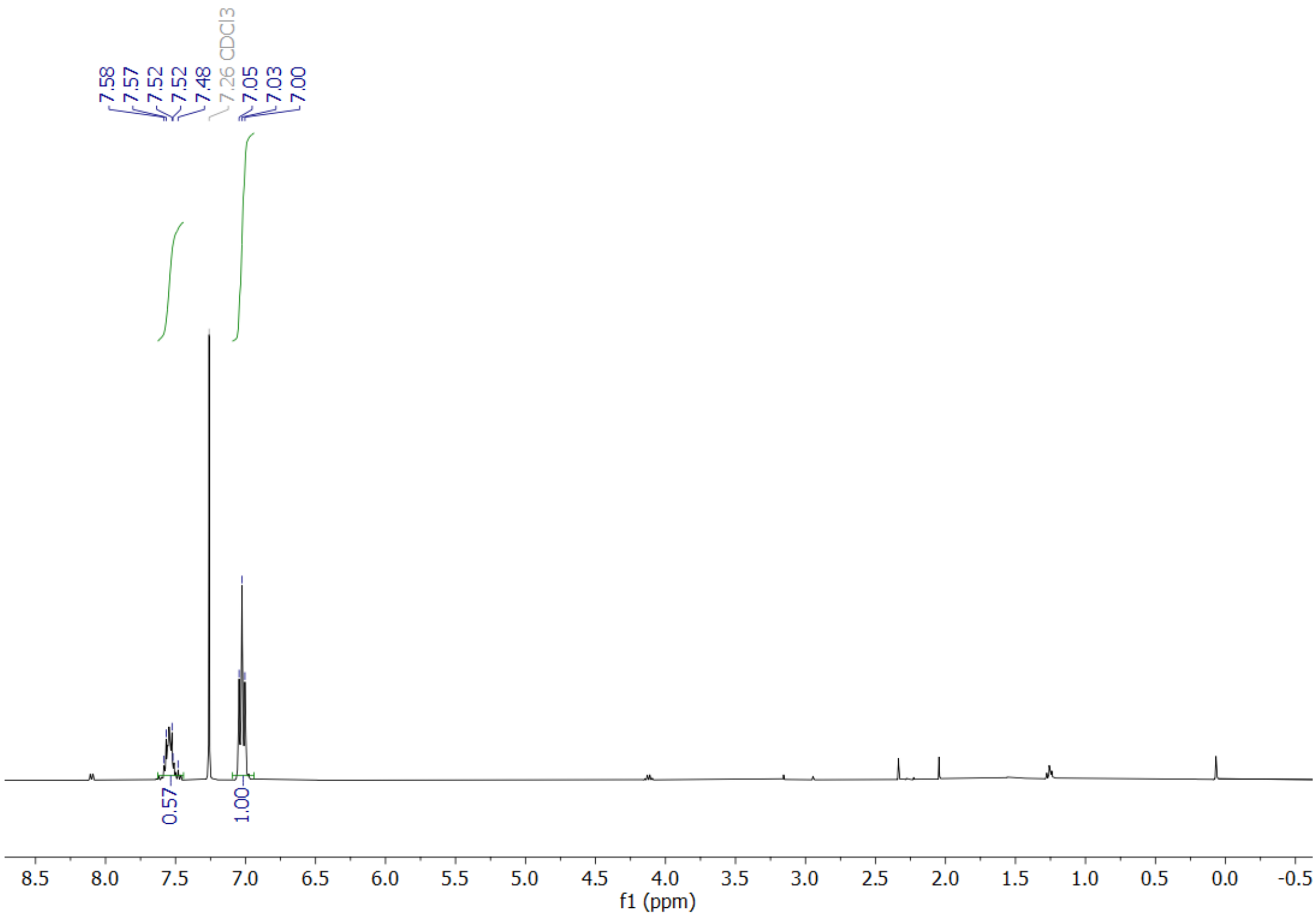

Figure S.4.6 $^{13}C\{^{1}H\}$ NMR spectrum of 2,6-difluorobenzoic anhydride.

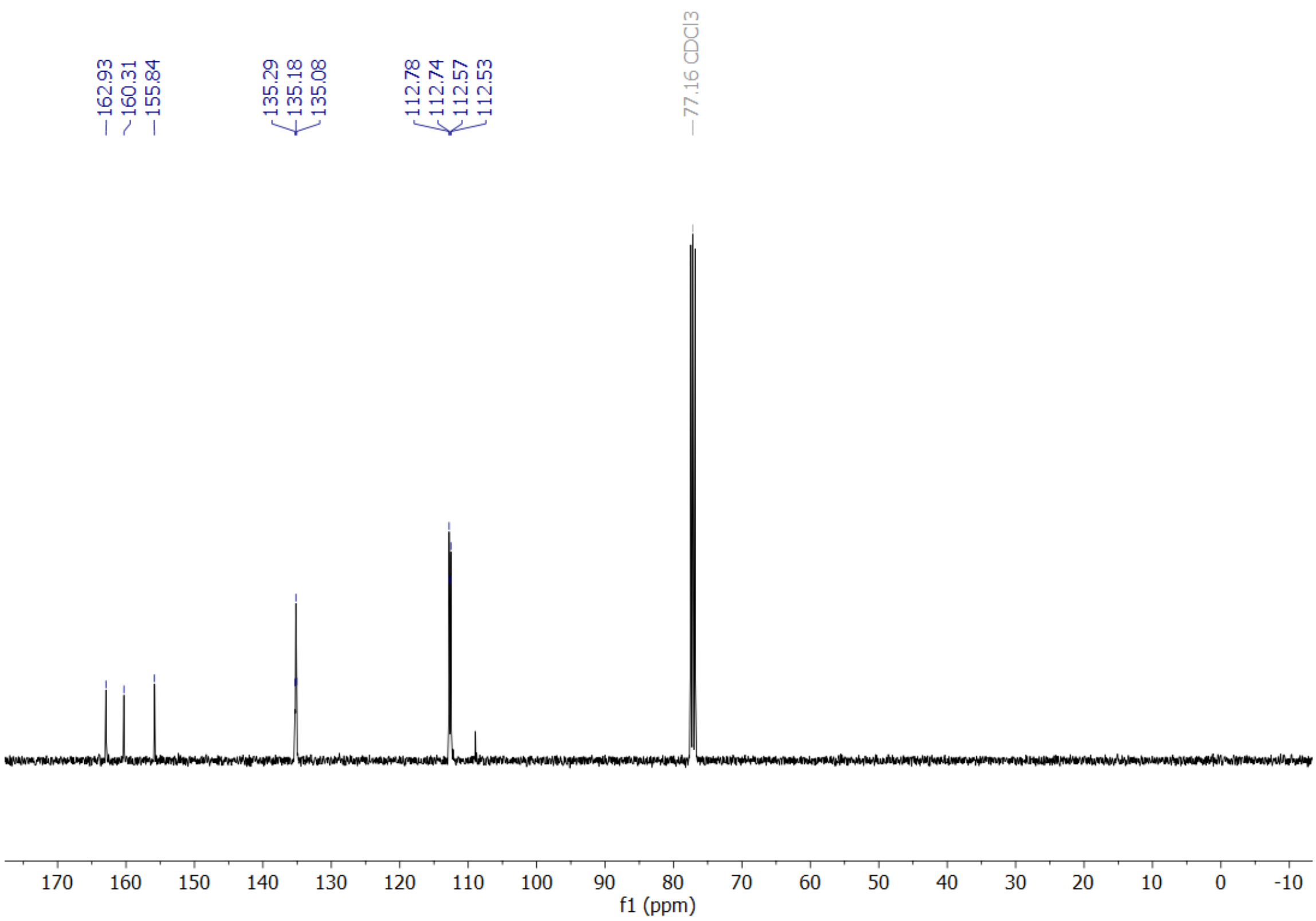

Figure S.4.7 ^{19}F NMR spectrum of 2,6-difluorobenzoic anhydride.

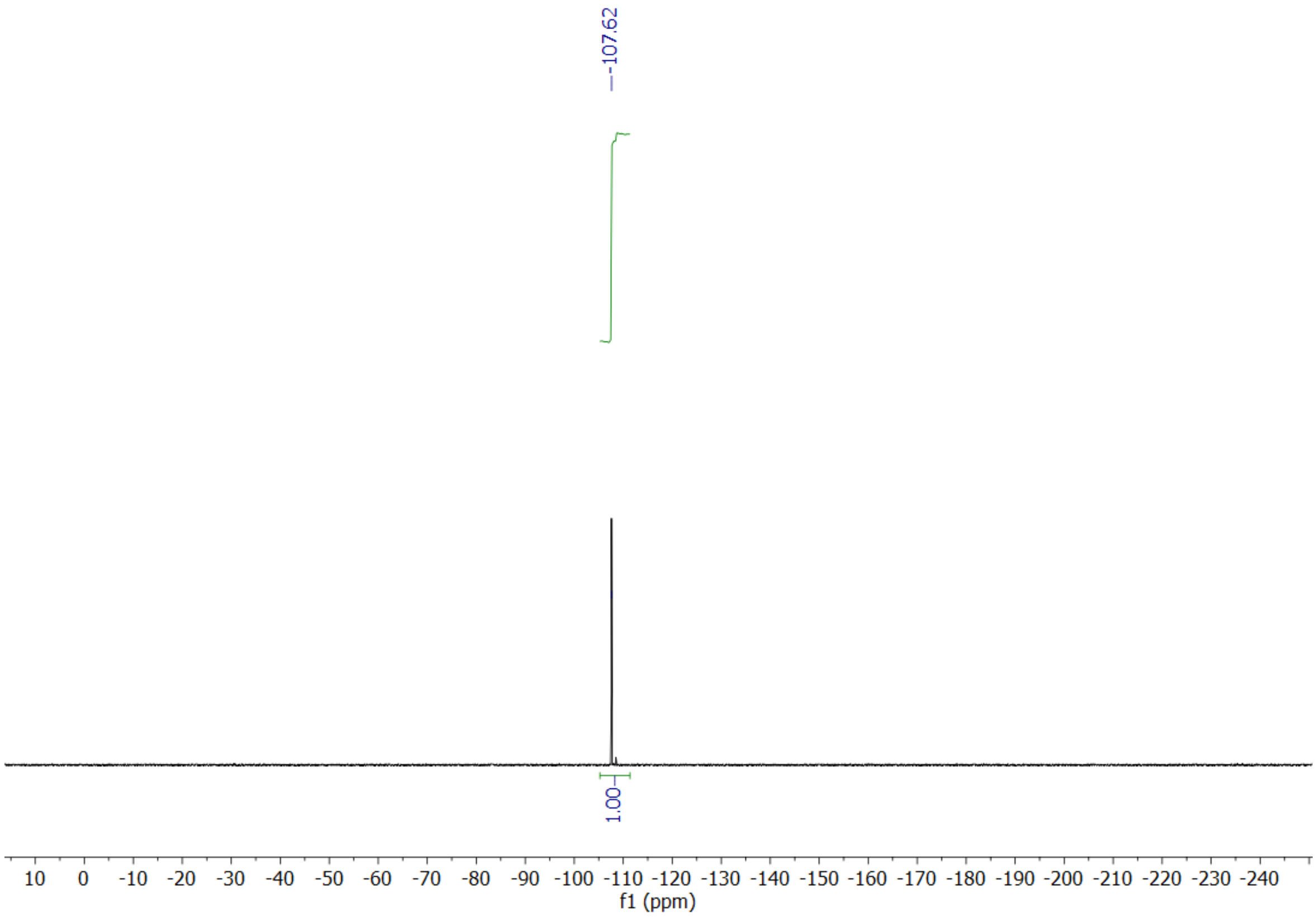

Figure S. 4.8 ^{1}H NMR spectrum of 4-trifluoromethylbenzoic anhydride (*4-trifluoromethylbenzoyl chloride).

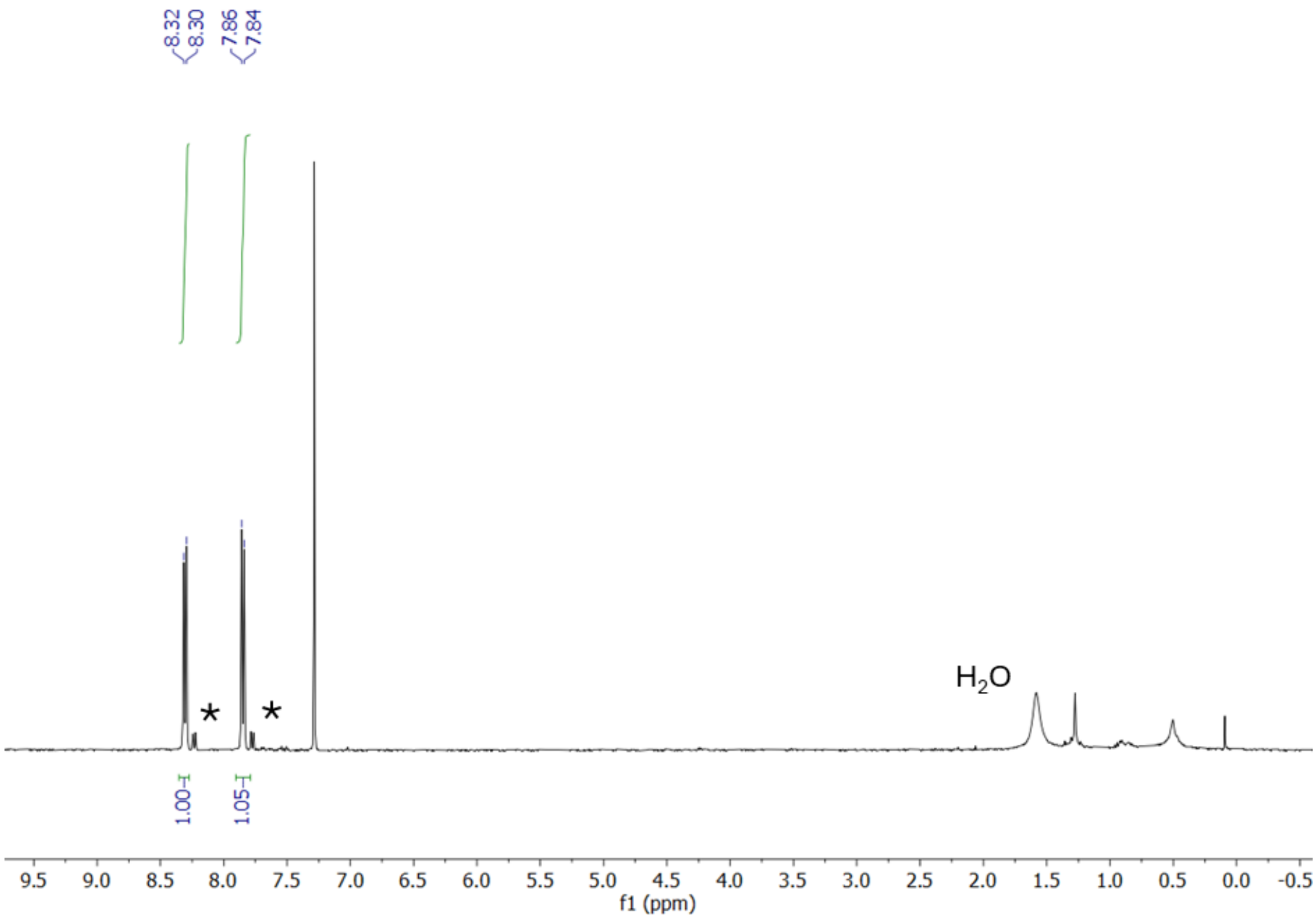

Figure S.4.9 $^{13}C\{^1H\}$ NMR spectrum of 4-trifluoromethylbenzoic anhydride.

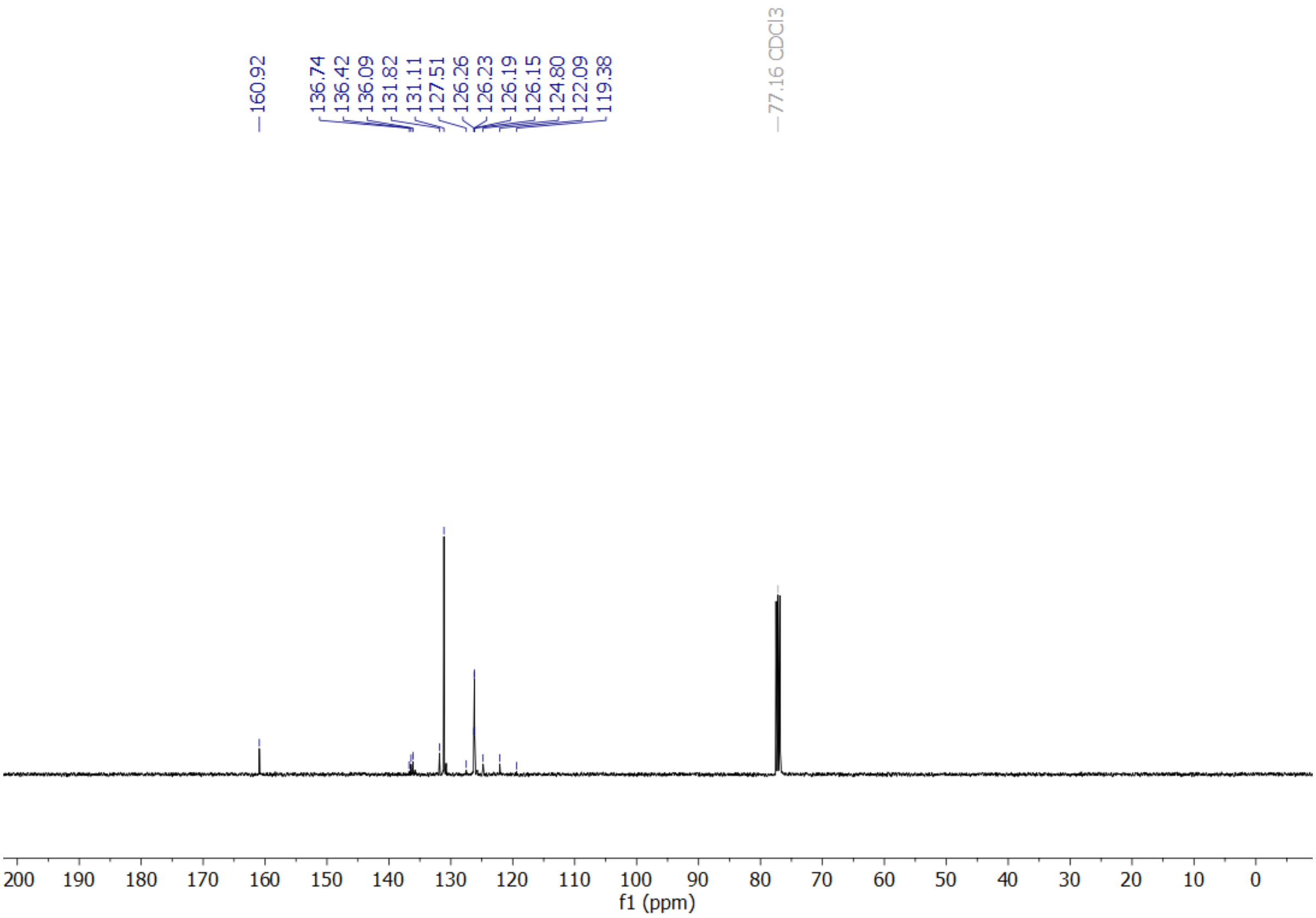

Figure S.4.10 ^{19}F NMR spectrum of 4-trifluoromethylbenzoic anhydride.

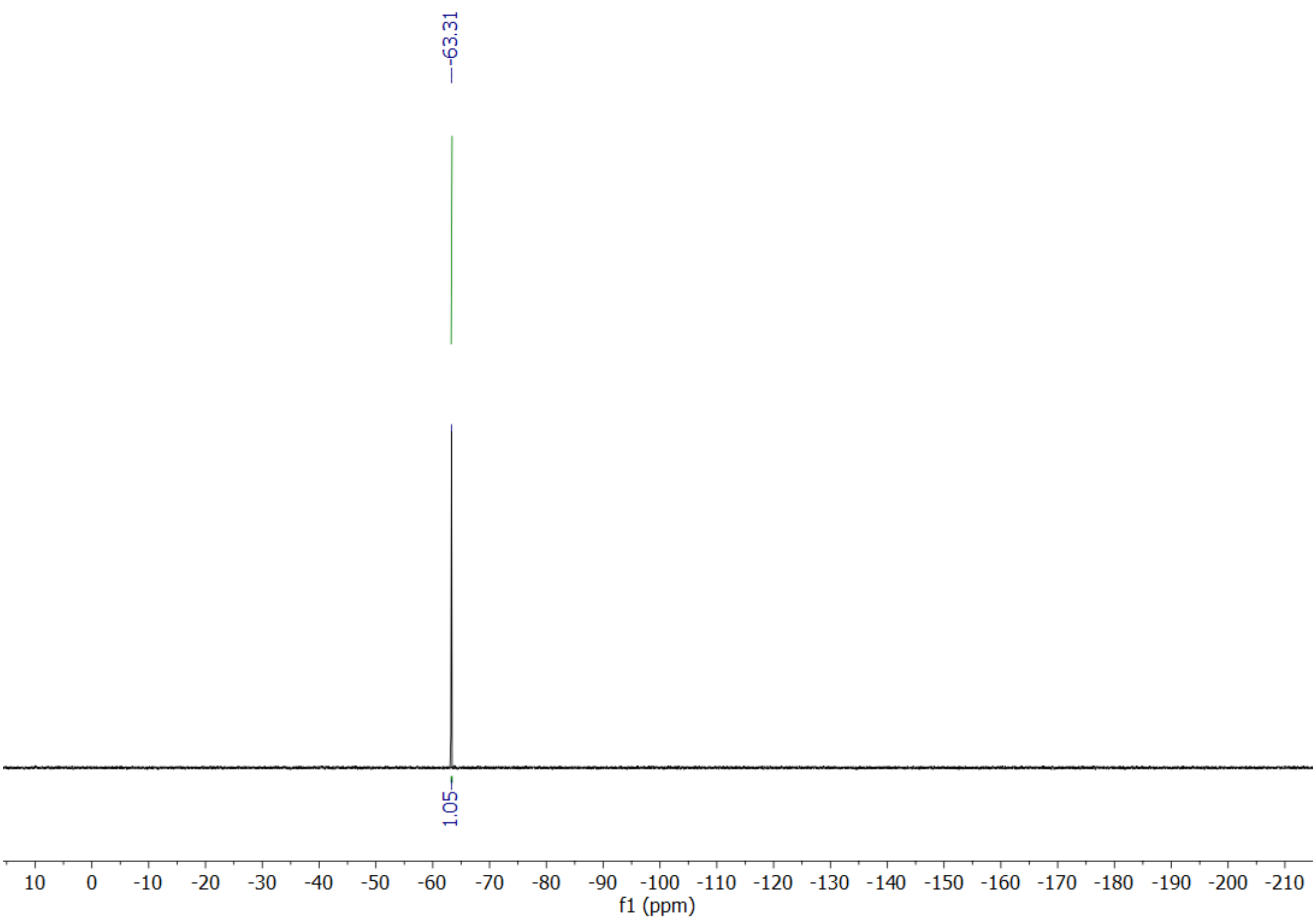

Figure S.4.11 ^{1}H NMR spectrum of 4-methoxybenzoic anhydride.

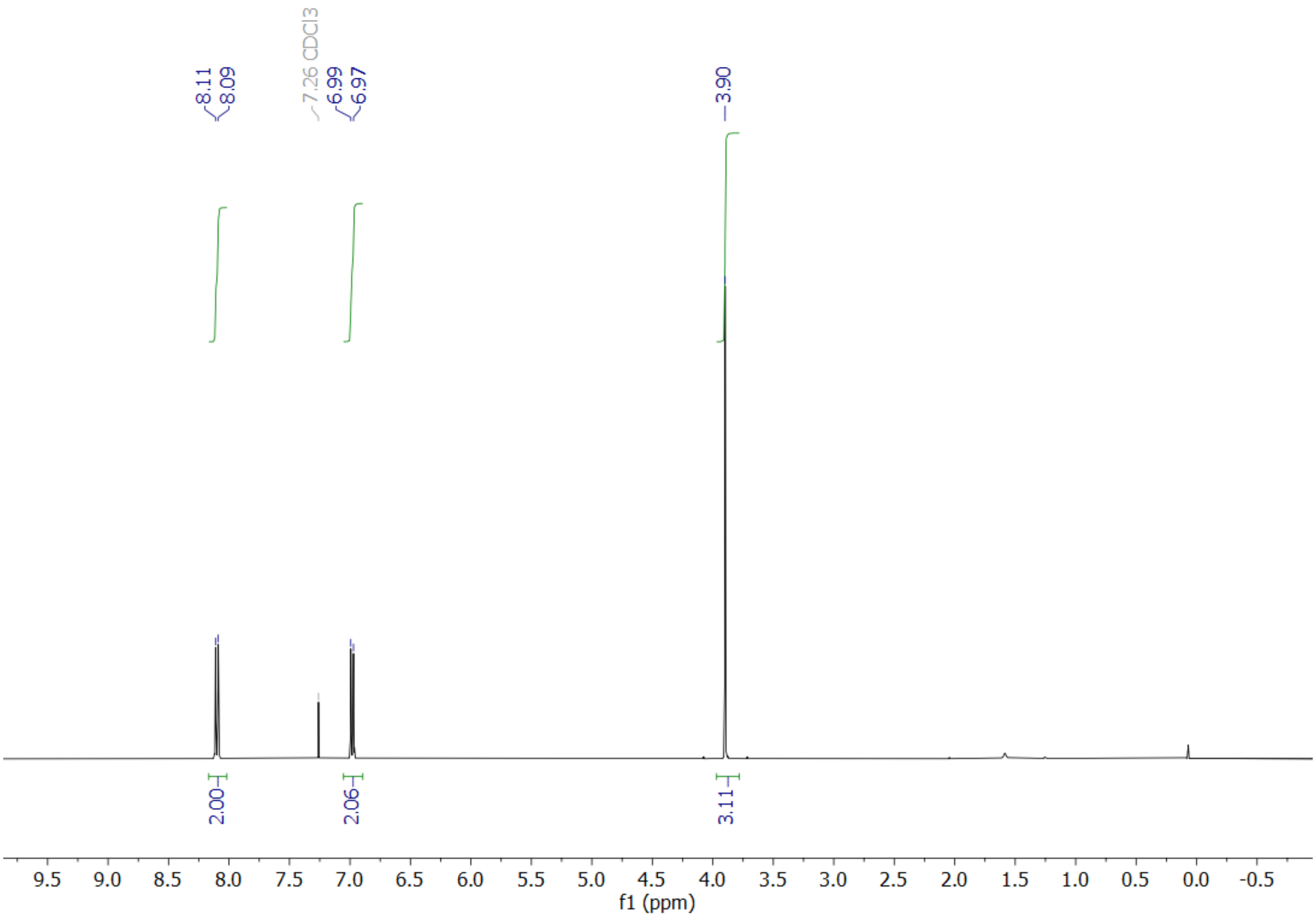

Figure S.4.12 $^{13}C\{^{1}H\}$ NMR spectrum of 4-methoxybenzoic anhydride.

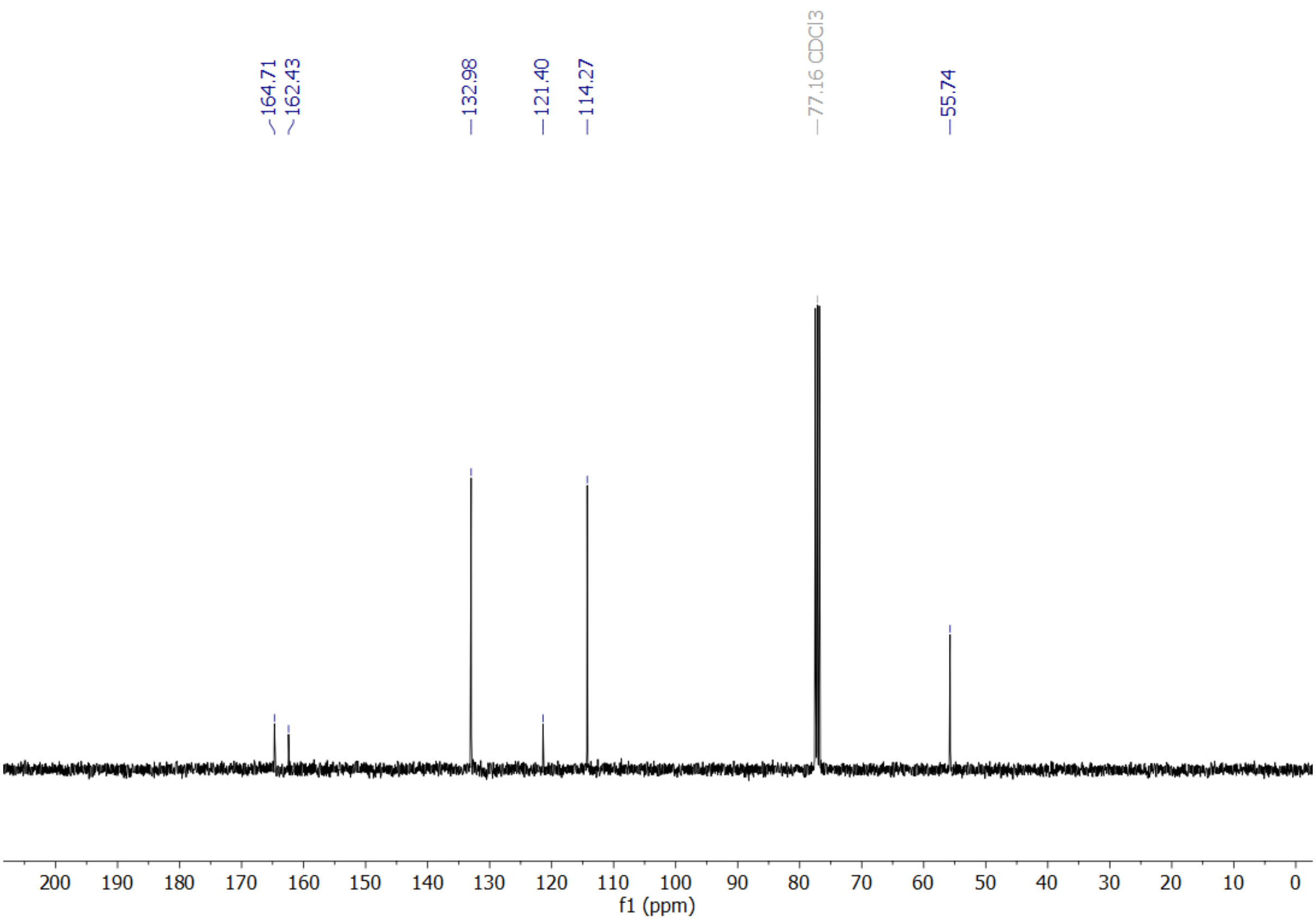

Figure S.4.13 ^{1}H NMR spectrum of 2-naphtholic anhydride.

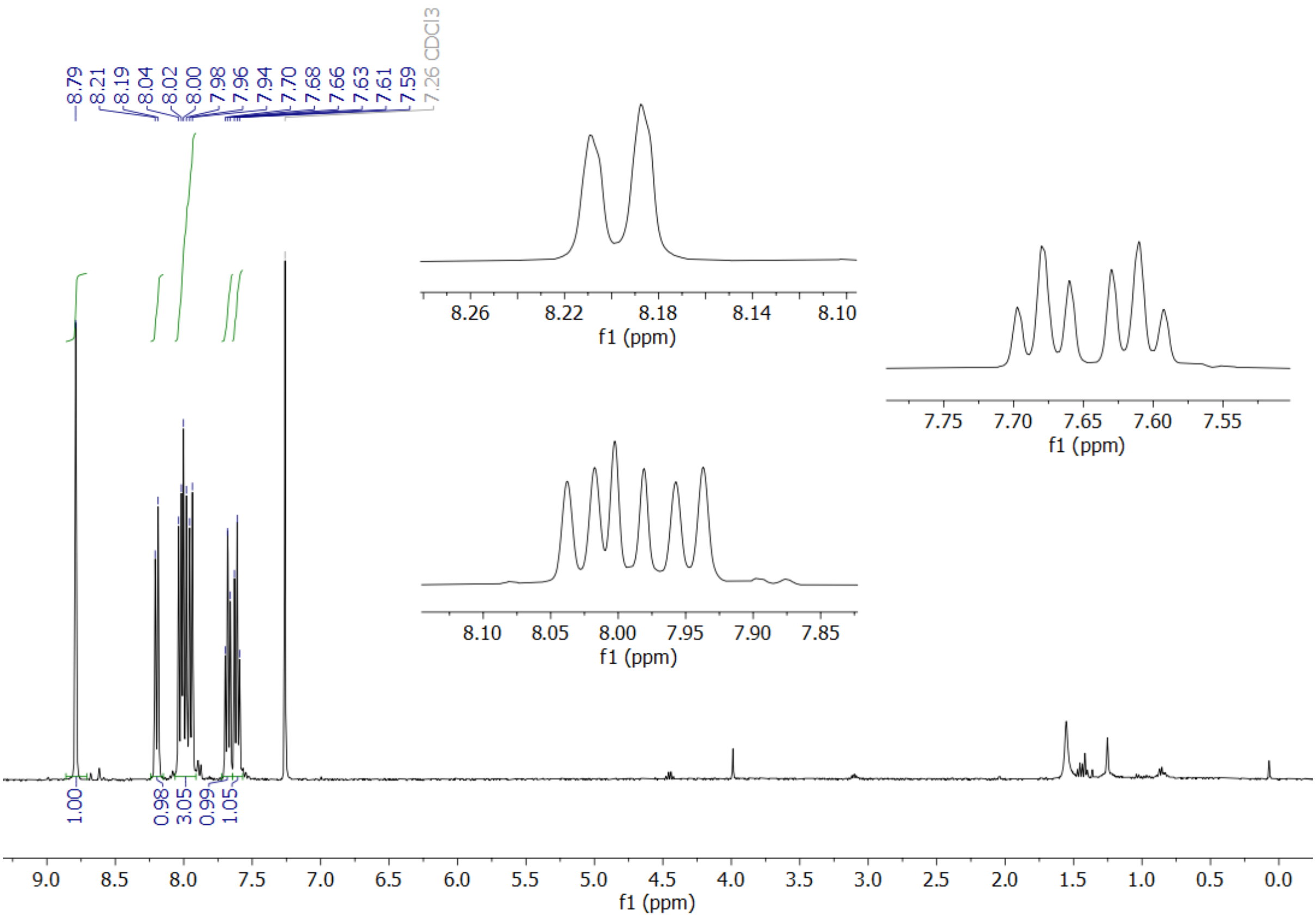

Figure S.4.14 $^{13}C\{^{1}H\}$ NMR spectrum of 2-naphtholic anhydride.

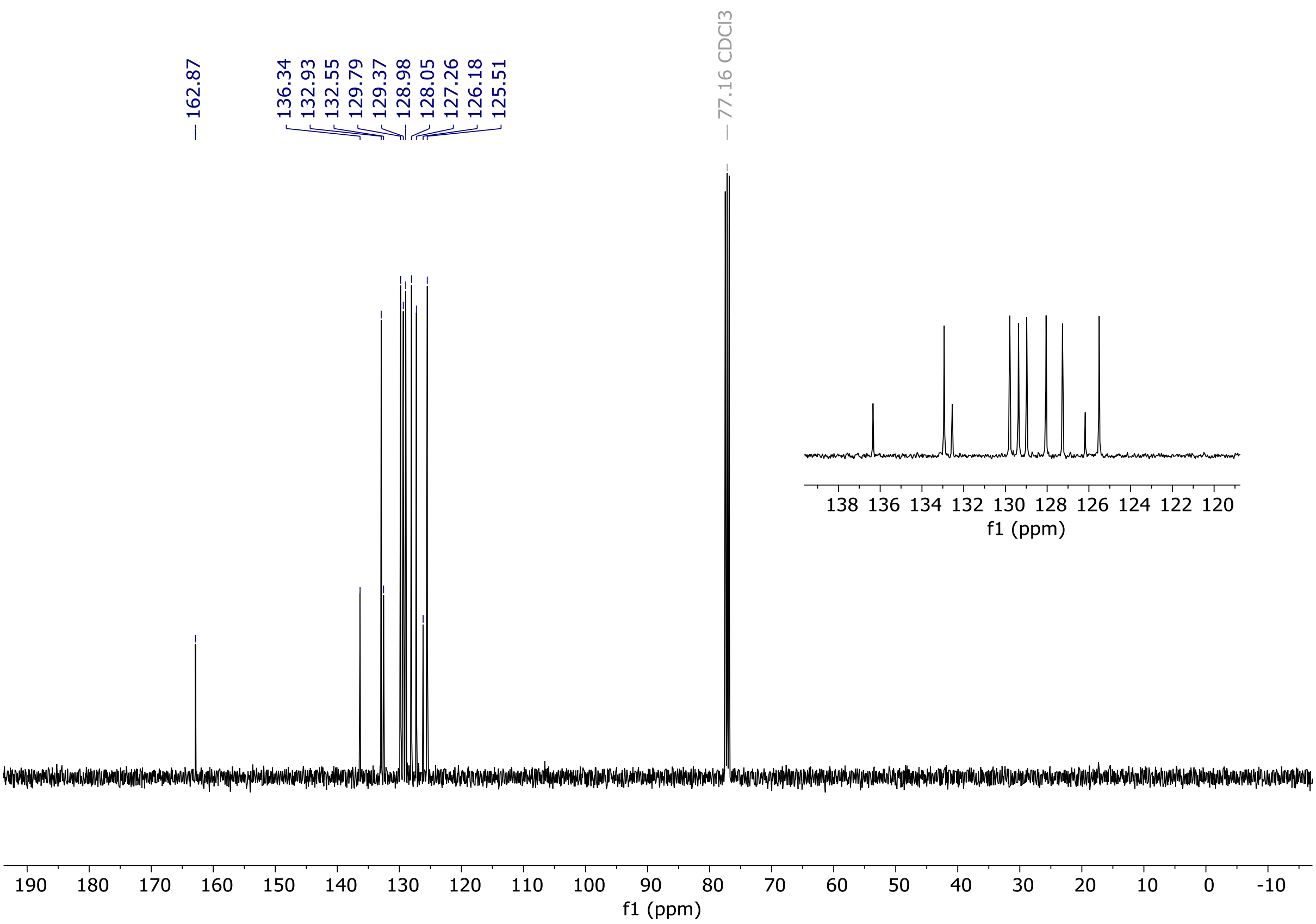

Figure S.4.15 ^{1}H NMR spectrum of BF_3·PCy_3.

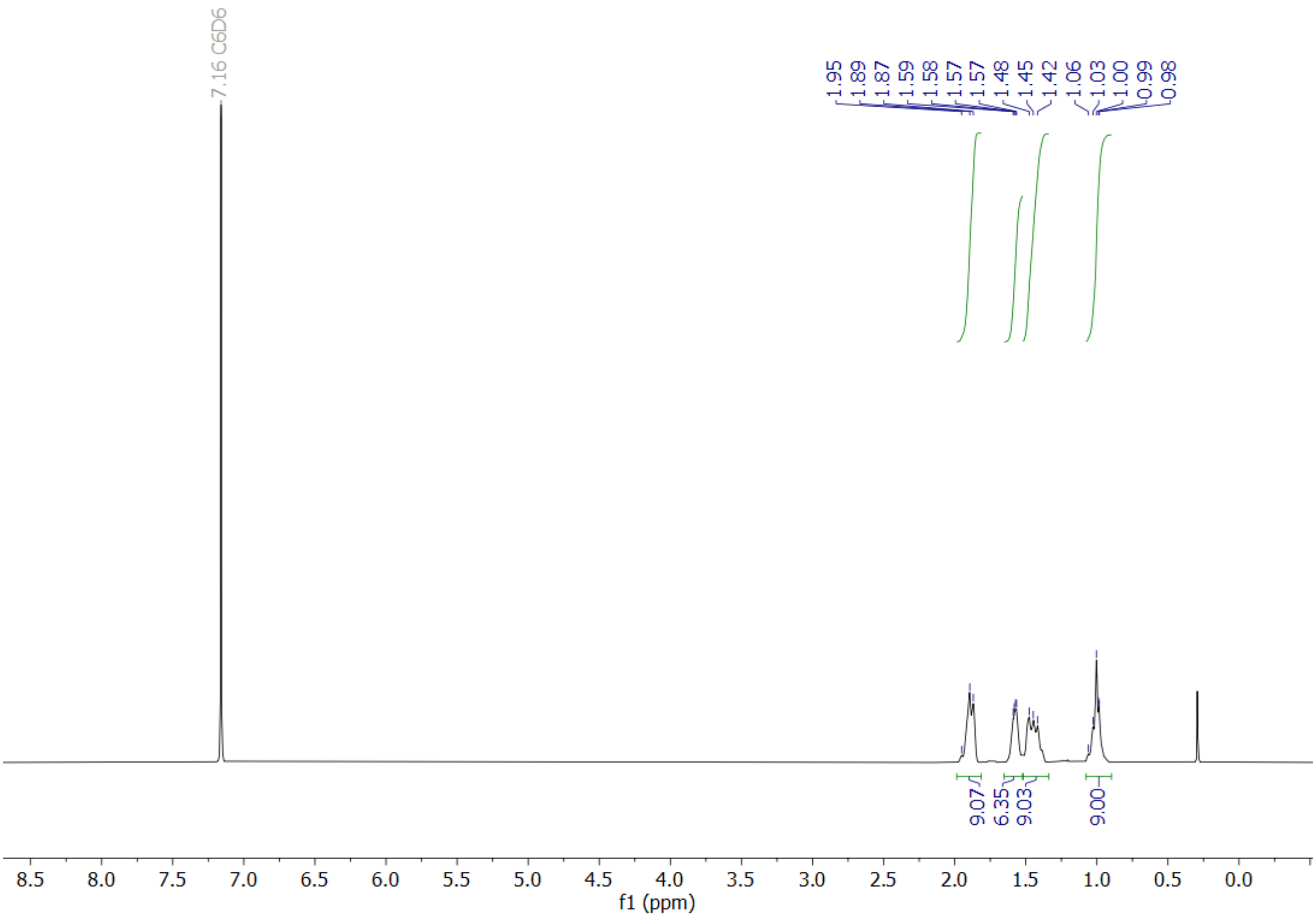

Figure S.4.16 ^{13}C{^{1}H} NMR spectrum of BF_3·PCy_3.

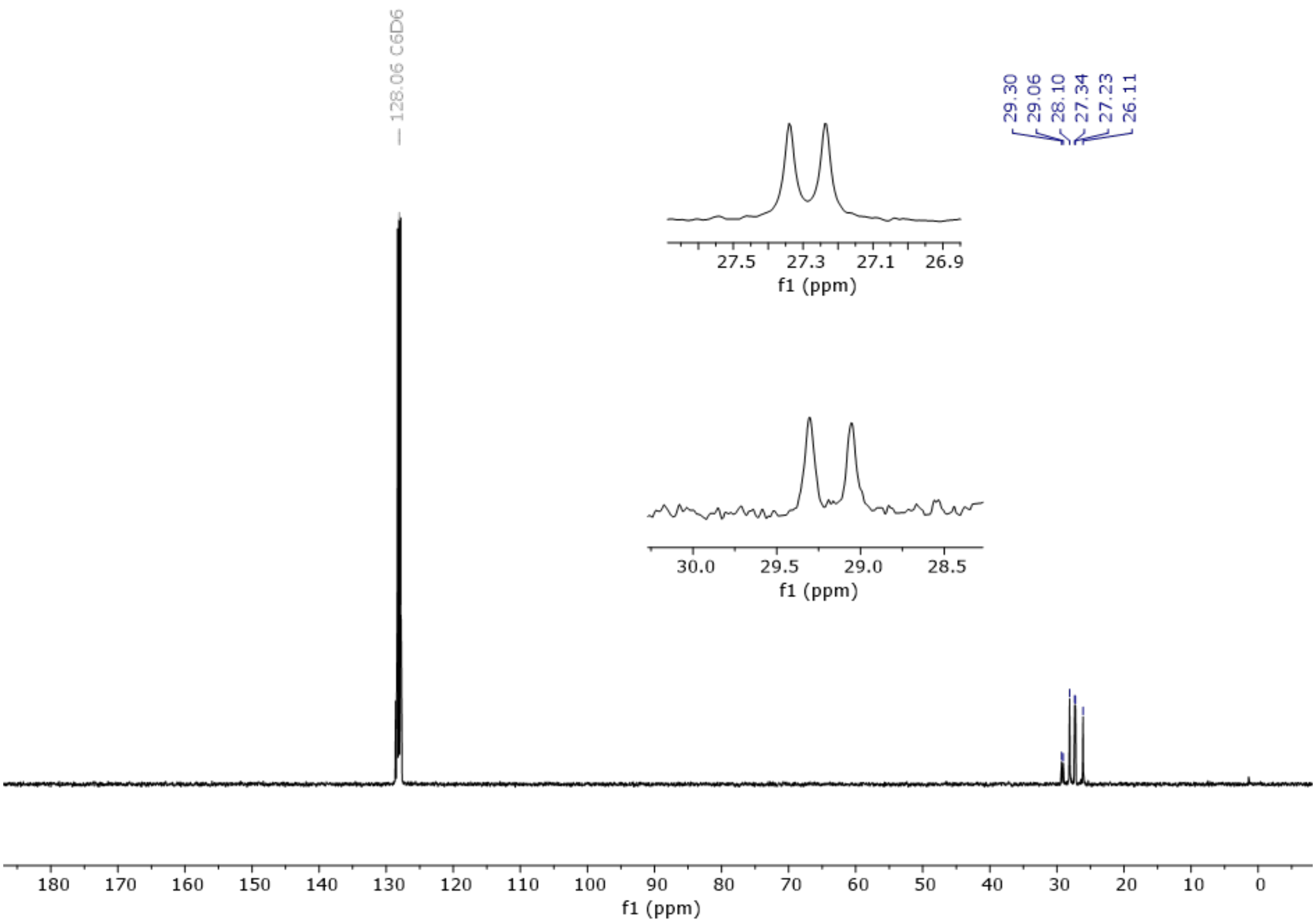

Figure S.4.17 ^{19}F NMR spectrum of $BF_3{\cdot}PCy_3$.

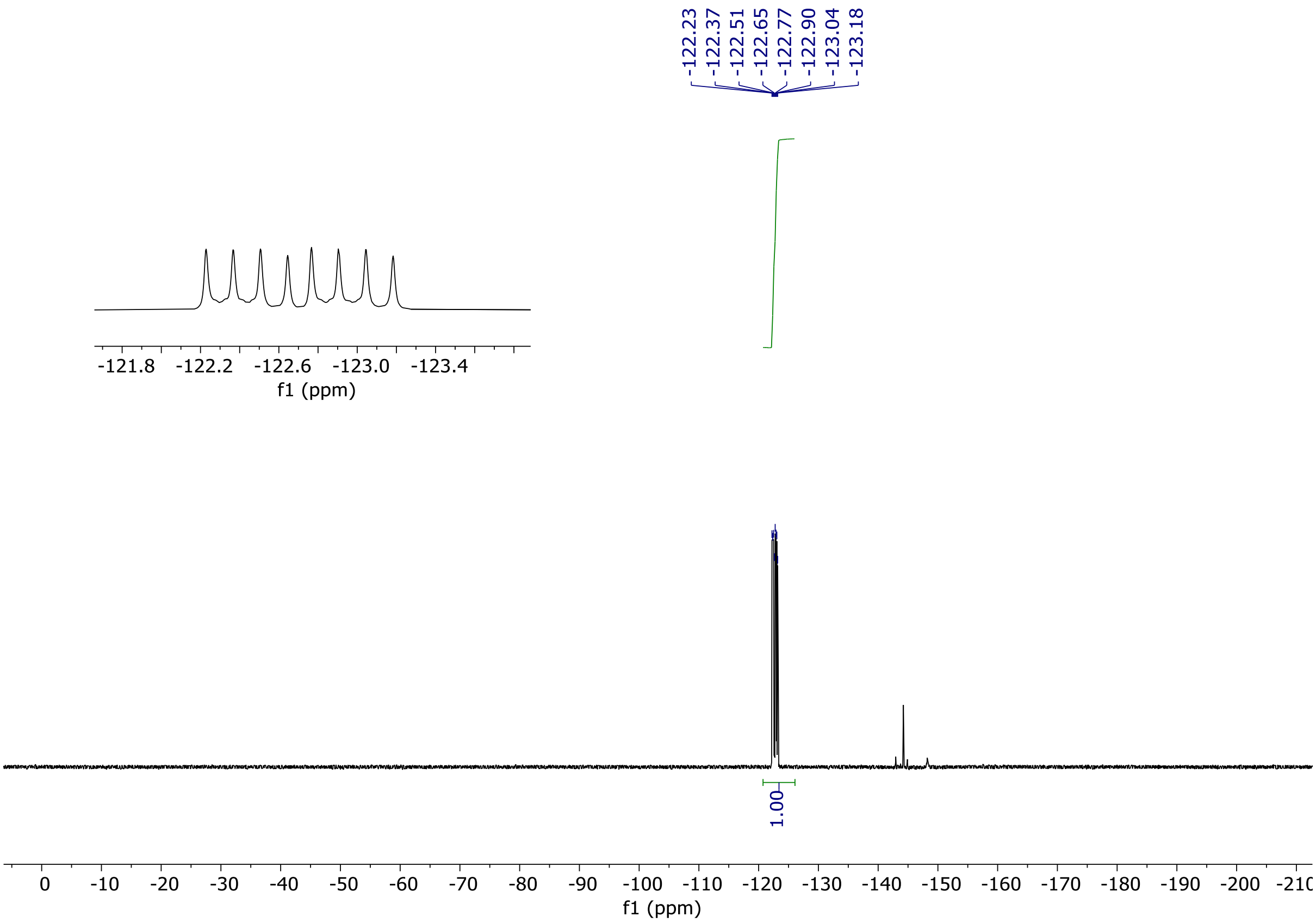

Figure S.4.18 $^{31}P\{^1H\}$ NMR spectrum of $BF_3{\cdot}PCy_3$ (*$HPCy_3{\cdot}BF_4$).

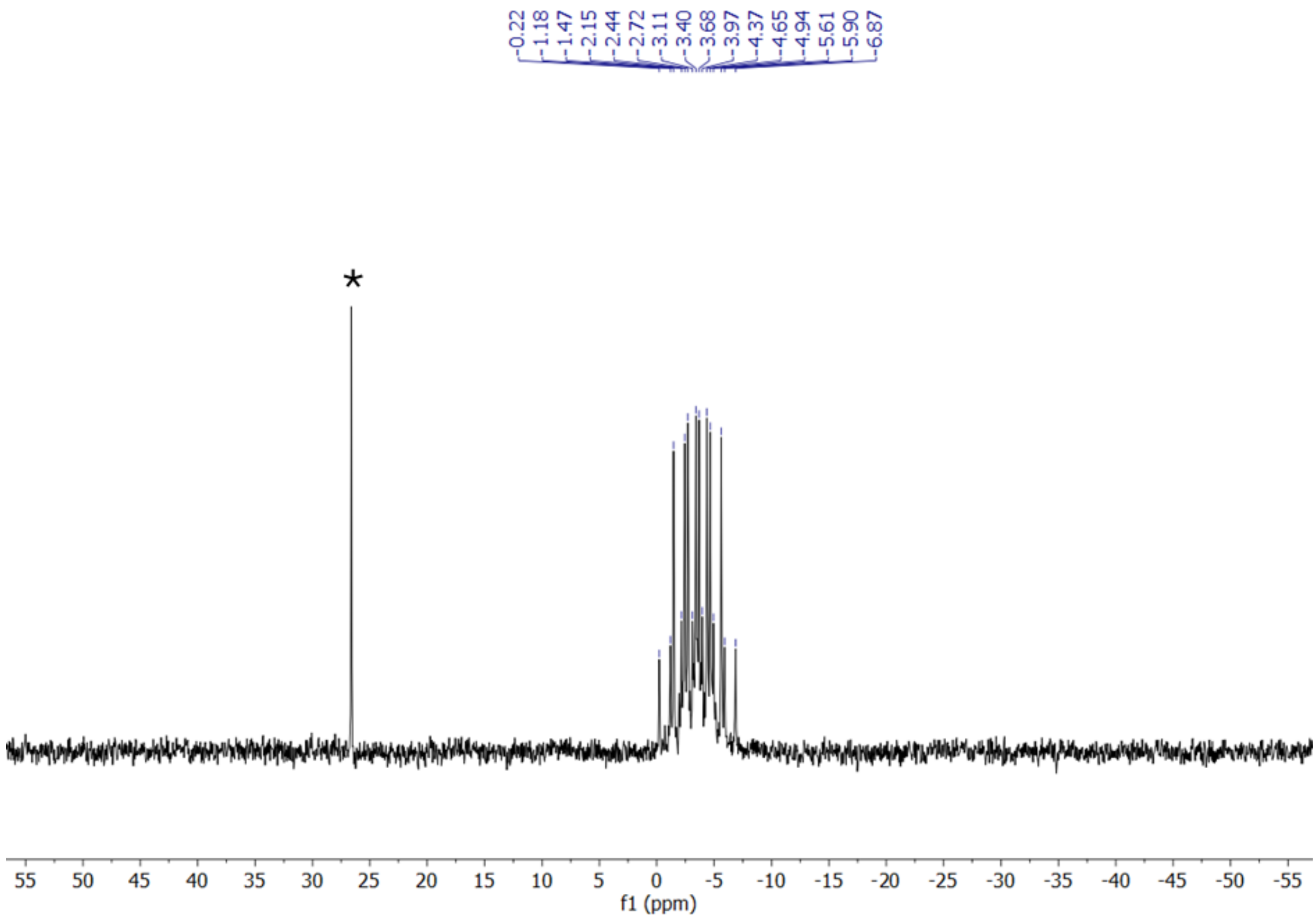

Figure S.4.19 ^{11}B NMR spectrum of $BF_3{\cdot}PCy_3$.

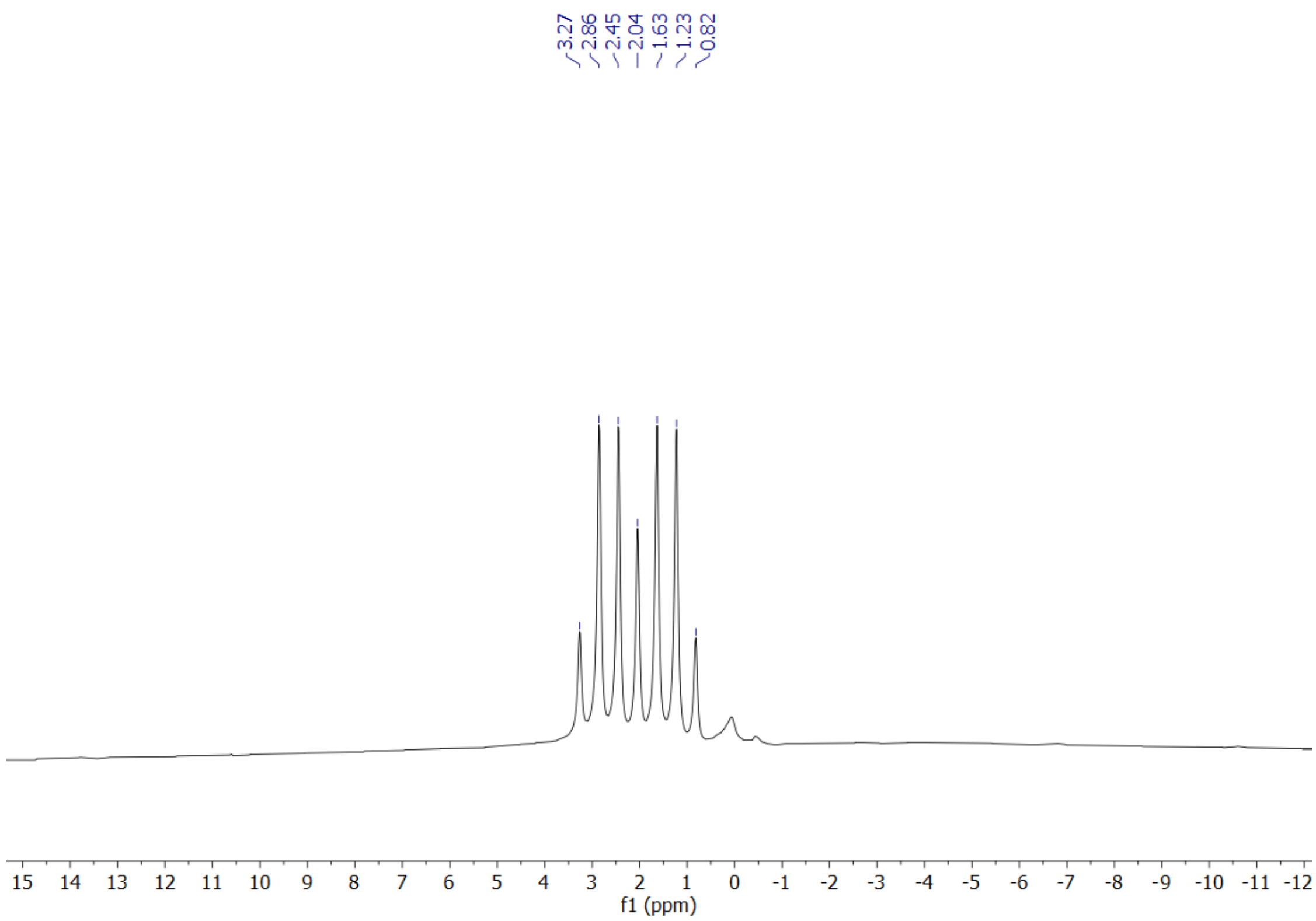

Figure S.4.20 ^{19}F NMR spectrum of the reaction of PVDF (0.384 mmol) with 4-phenyl-1-butyne (0.192 mmol) catalysed by $BF_3{\cdot}OEt_2$ (0.048 mmol) and $BF_3{\cdot}PCy_3$ (0.048 mmol) generating the mixture of fluorocarbons **S1a**, **S1b**, **S1c** and **S1d**. Yields monitored by quantitative ^{19}F NMR spectroscopy against a fluorobenzene internal standard.

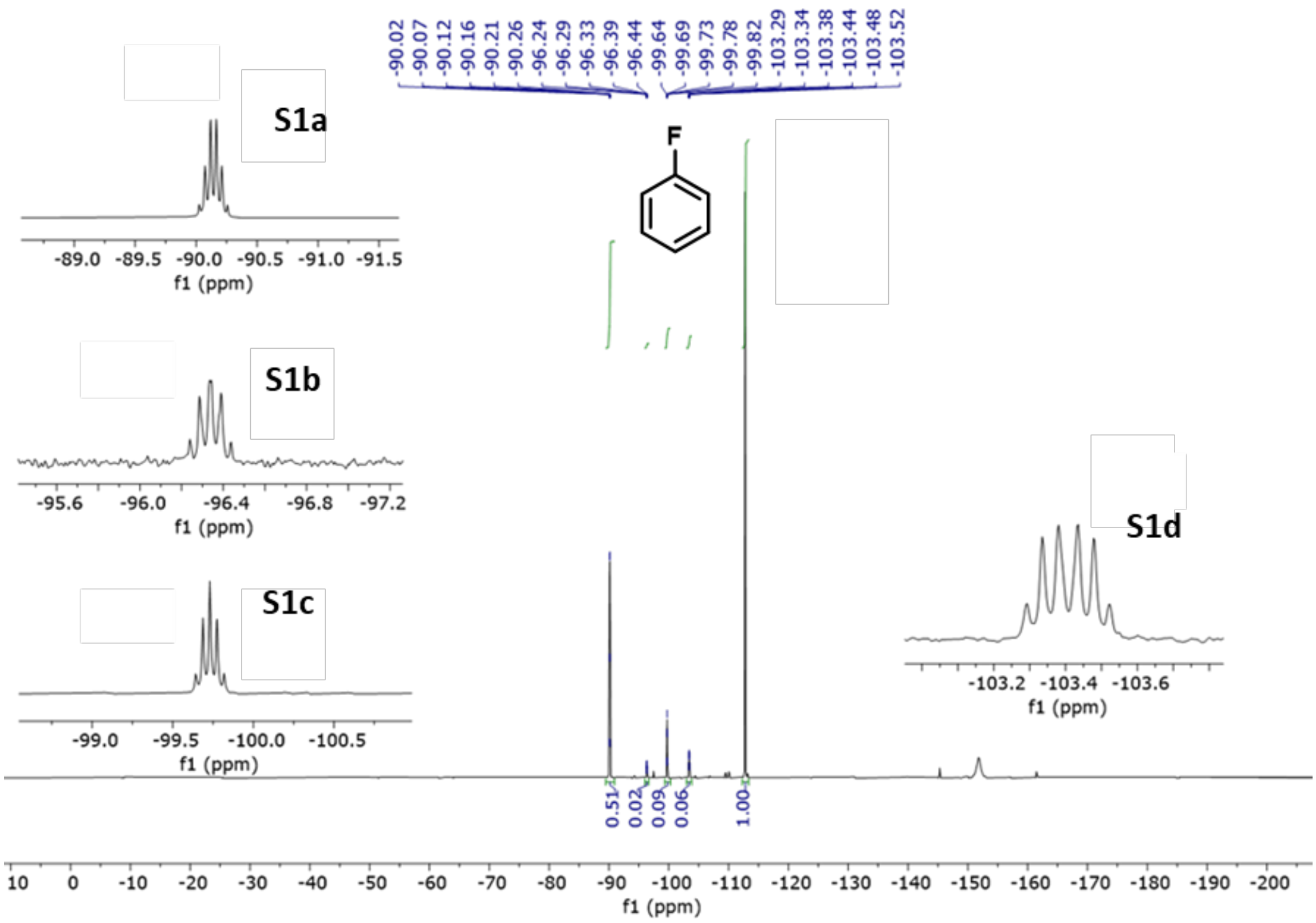

Figure S.4.21 ^{19}F NMR spectrum of the reaction of PVDF (0.384 mmol) with 1-dodecyne (0.192 mmol) catalysed by $BF_3{\cdot}OEt_2$ (0.048 mmol) and $BF_3{\cdot}PCy_3$ (0.048 mmol) generating the mixture of fluorocarbons **S2a**, **S2b**, **S2c** and **S2d**. Yields monitored by quantitative ^{19}F NMR spectroscopy against a fluorobenzene internal standard. (*Unidentified fluoroalkene isomer)

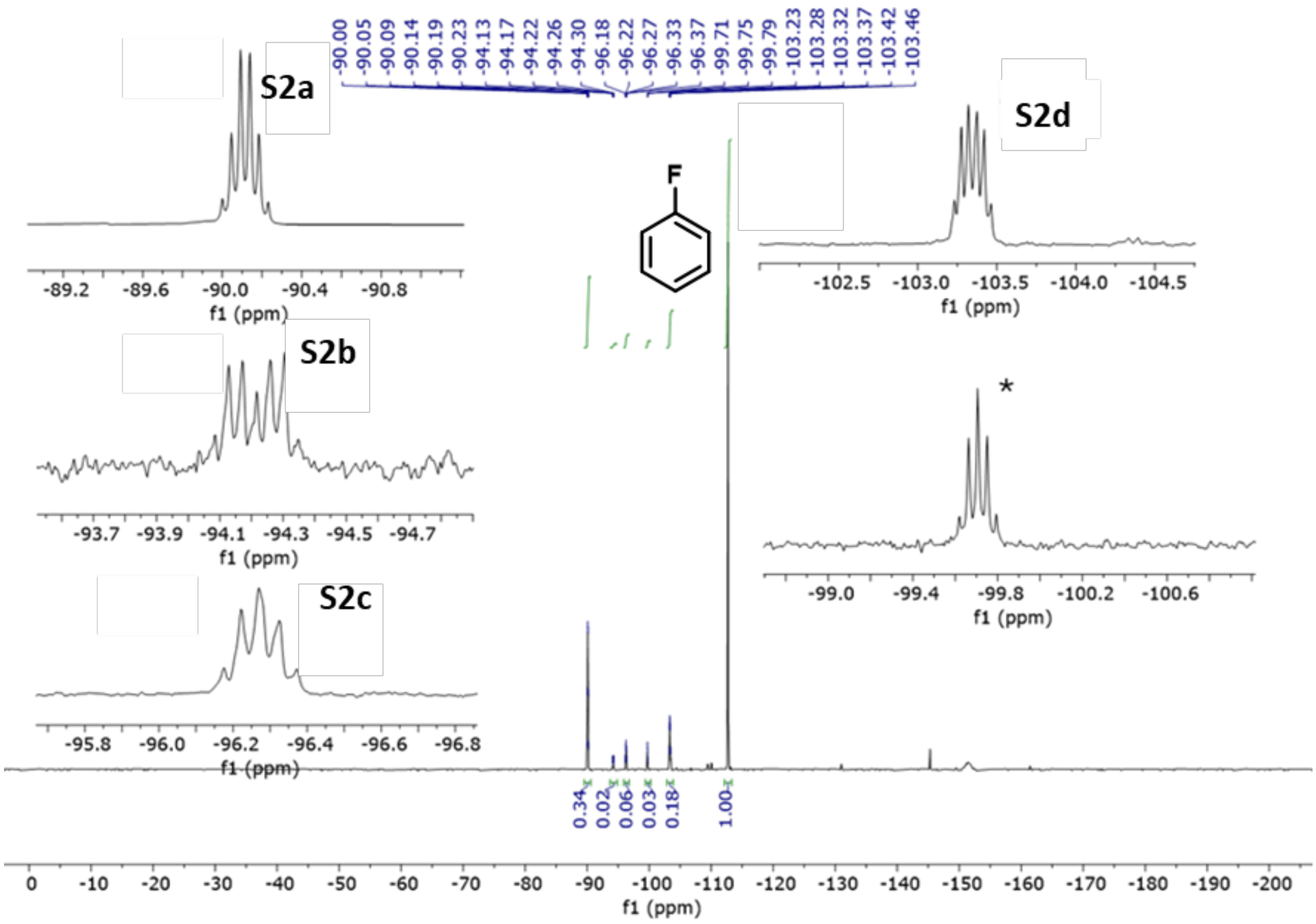

Figure S.4.22 ^{19}F NMR spectrum of the reaction of PVDF (0.384 mmol) with 6-chlorohex-1-yne (0.192 mmol) catalysed by $BF_3 \cdot OEt_2$ (0.048 mmol) and $BF_3 \cdot PCy_3$ (0.048 mmol) generating the mixture of fluorocarbons **S3a**, **S3b**, **S3c** and **S3d**. Yields monitored by quantitative ^{19}F NMR spectroscopy against a fluorobenzene internal standard.

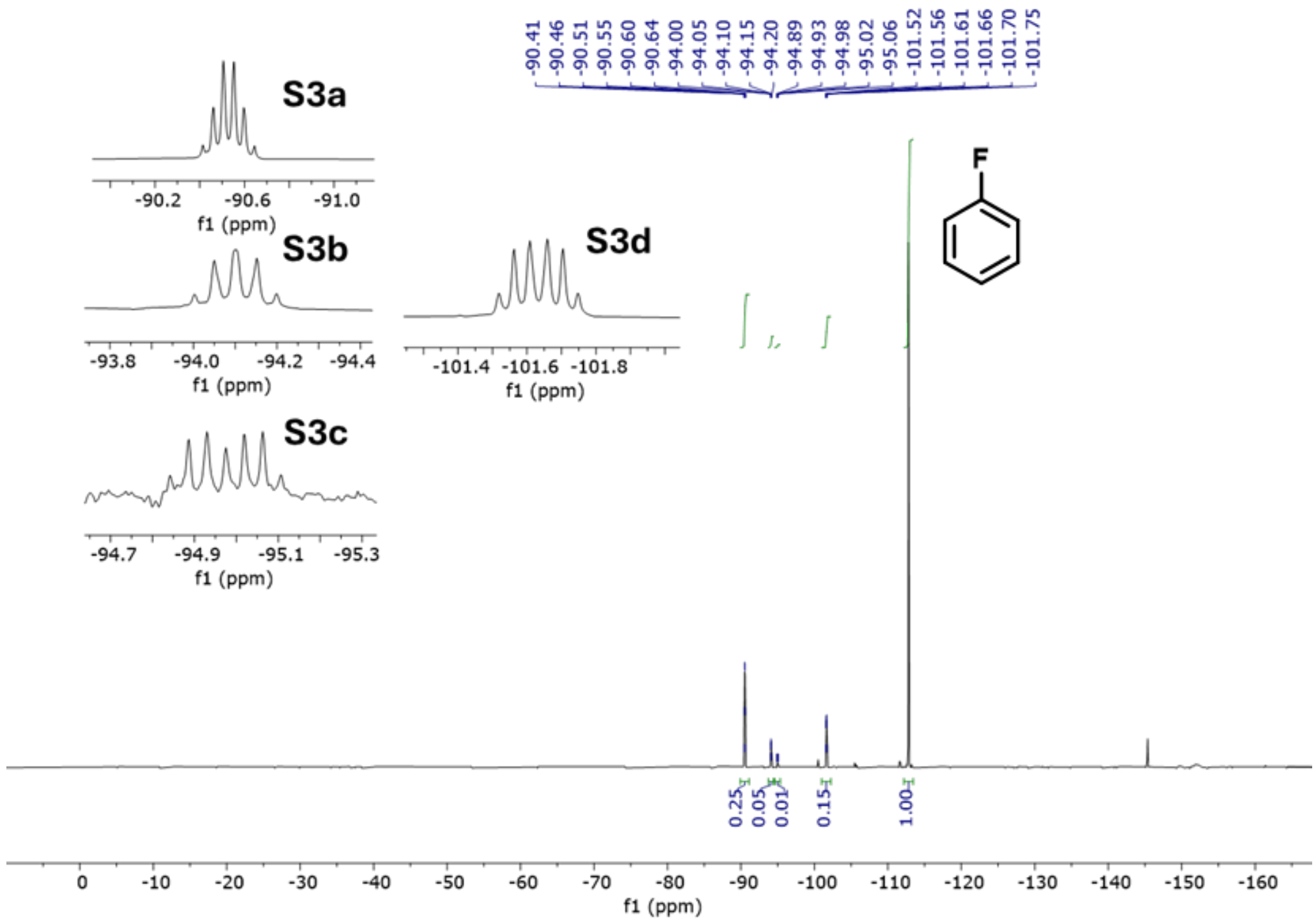

Figure S.4.23 ^{19}F NMR spectrum of the reaction of PVDF (0.384 mmol) with 6-bromo-hex-1-yne (0.192 mmol) catalysed by $BF_3 \cdot OEt_2$ (0.048 mmol) and $BF_3 \cdot PCy_3$ (0.048 mmol) generating the mixture of fluorocarbons **S4a**, **S4b**, **S4c** and **S4d**. Yields monitored by quantitative ^{19}F NMR spectroscopy against a fluorobenzene internal standard.

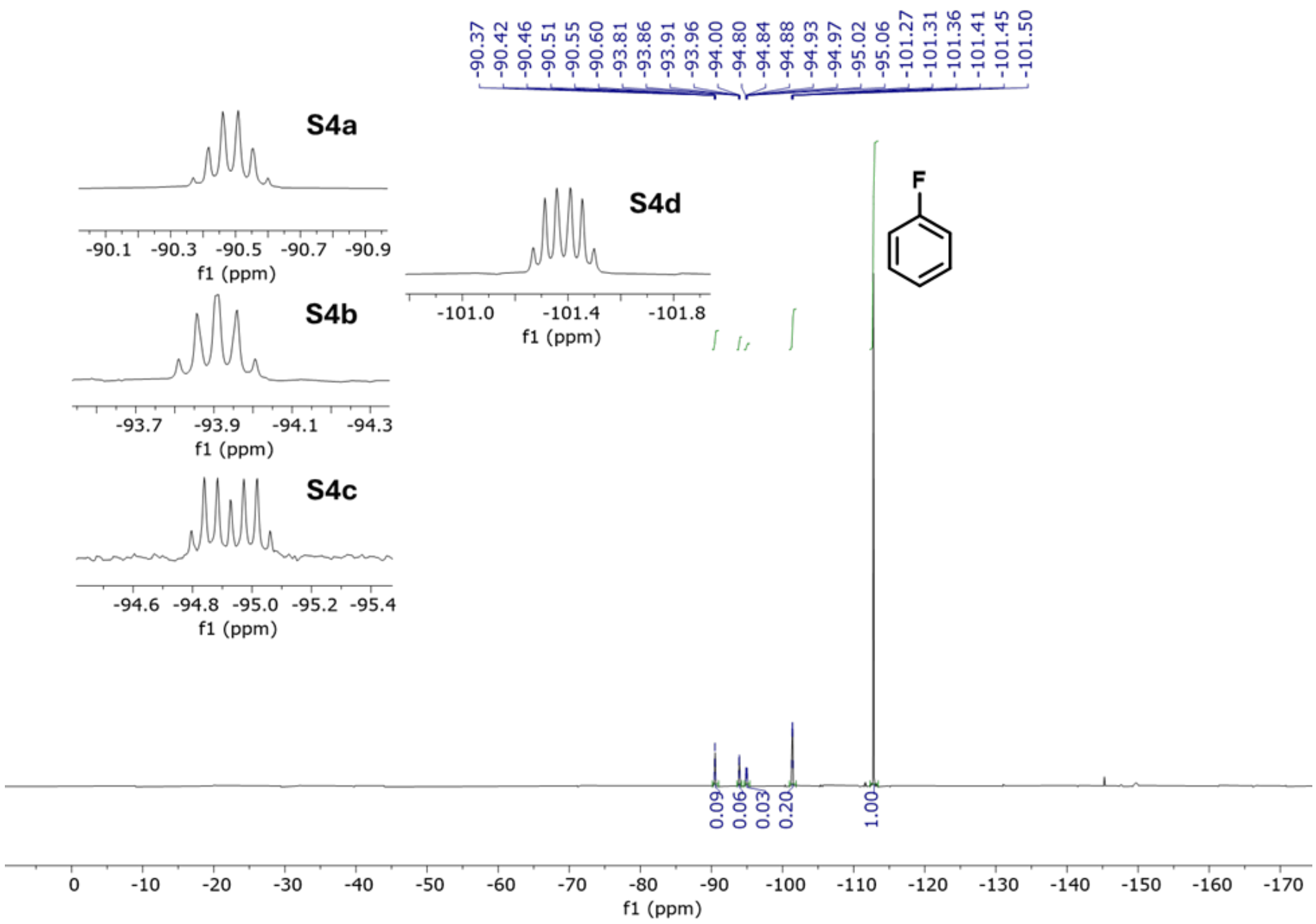

Figure S.4.24 ^{1}H NMR spectrum of benzoyl fluoride, **1a**.

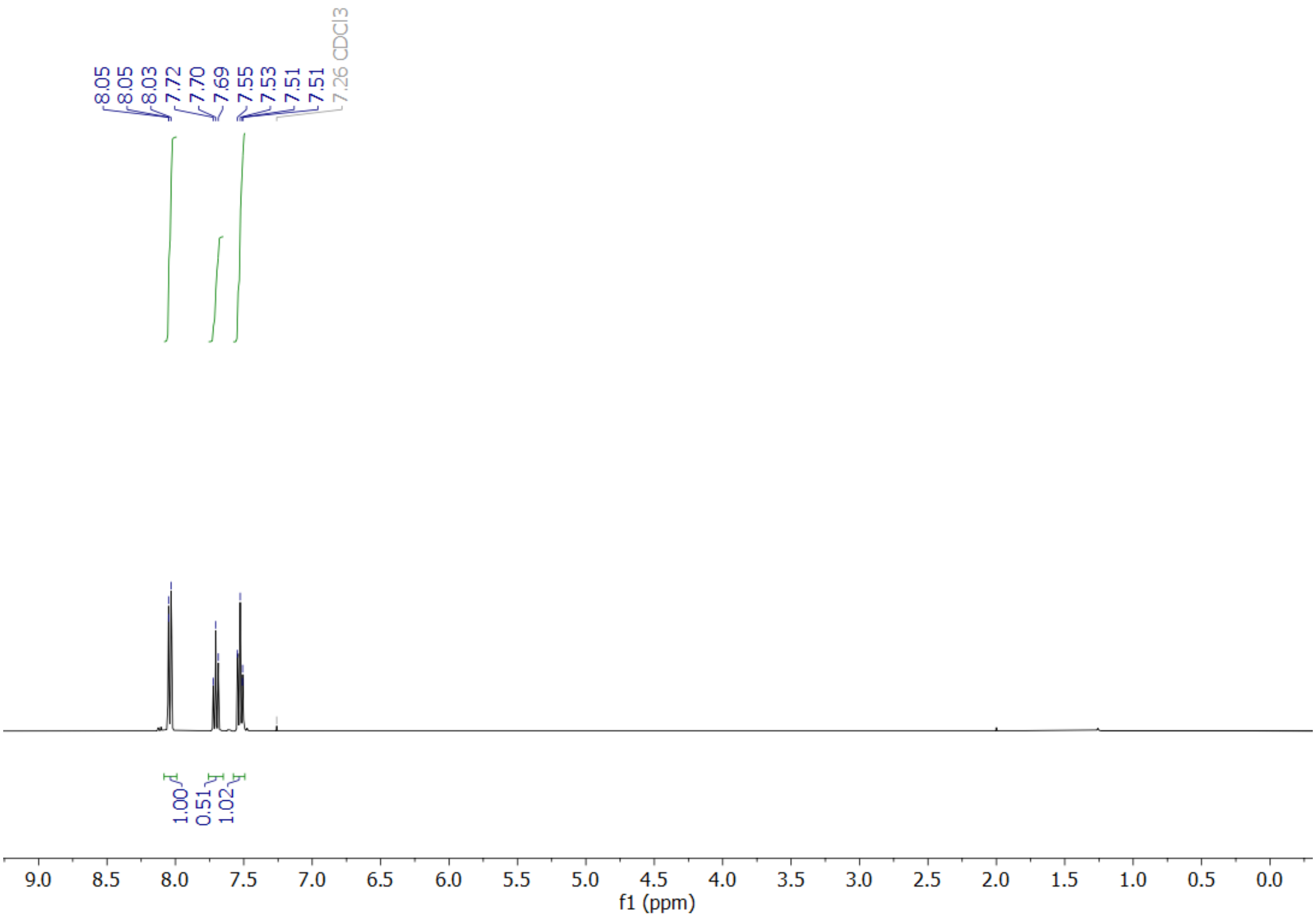

Figure S.4.25 $^{13}C\{^{1}H\}$ NMR spectrum of benzoyl fluoride, **1a**.

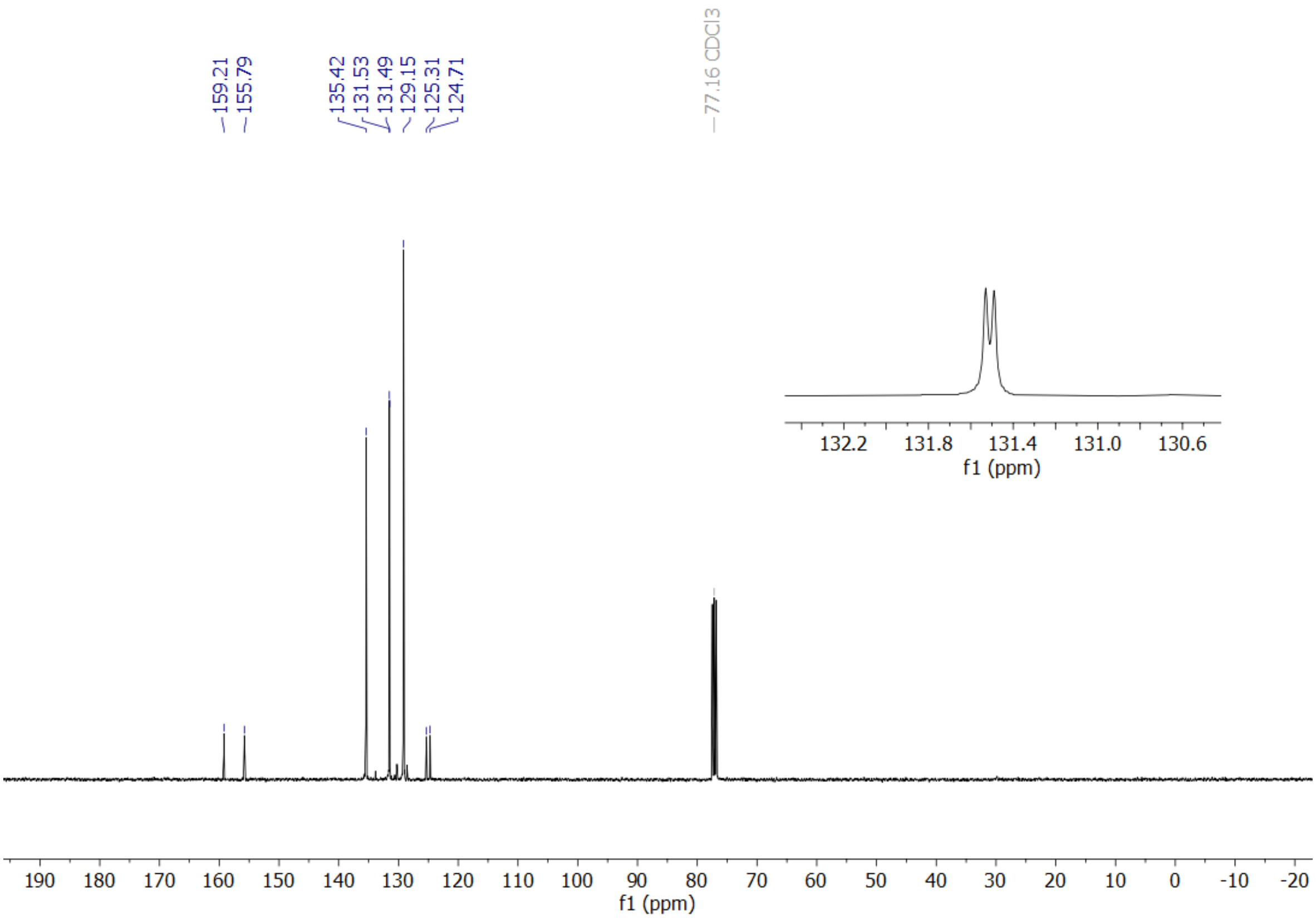

Figure S.4.26 ^{19}F NMR spectrum of benzoyl fluoride, **1a**.

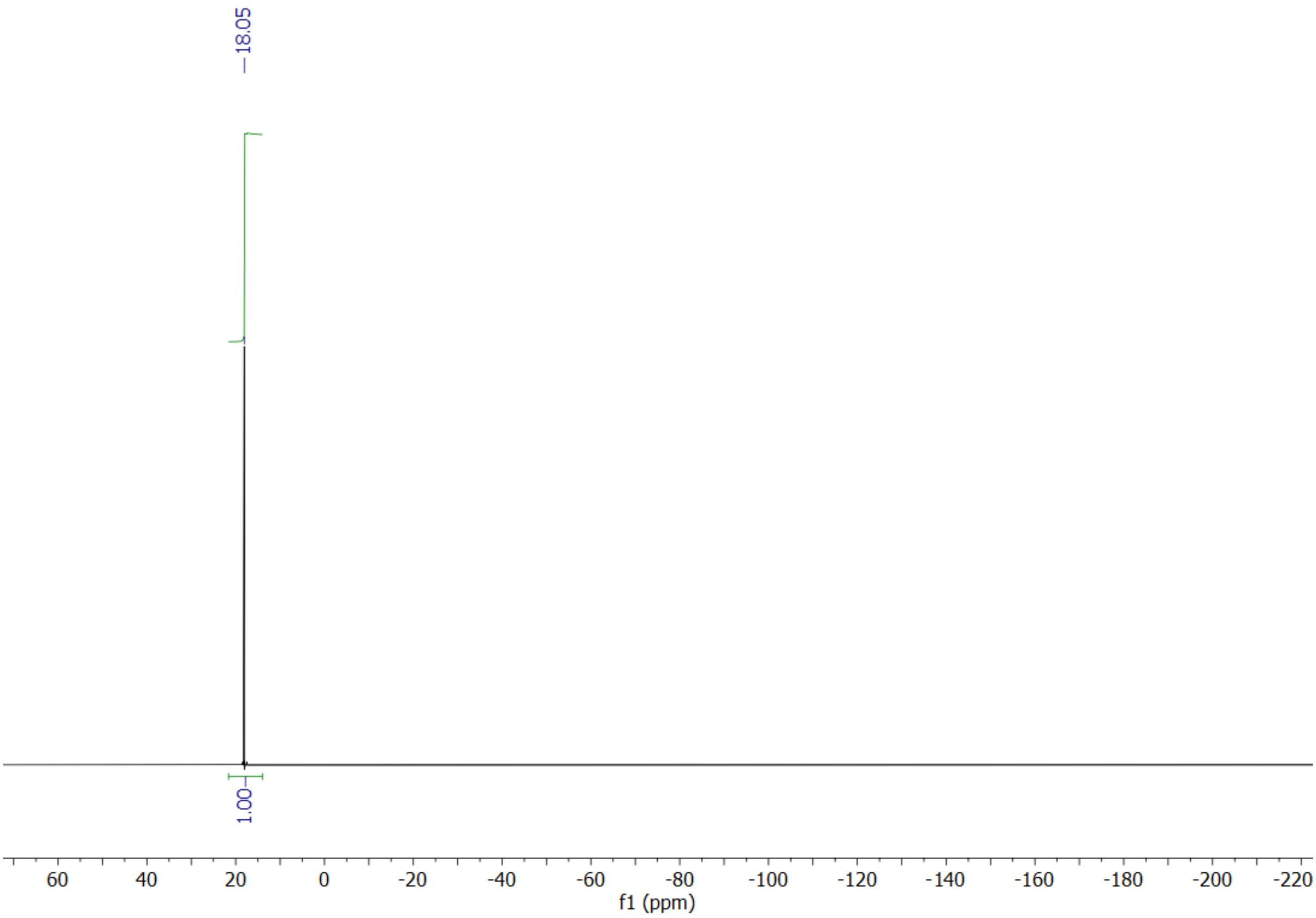

Figure S.4.27 ^{1}H NMR spectrum of 2,4,6-trimethylbenzoyl fluoride (*toluene), **1b**.

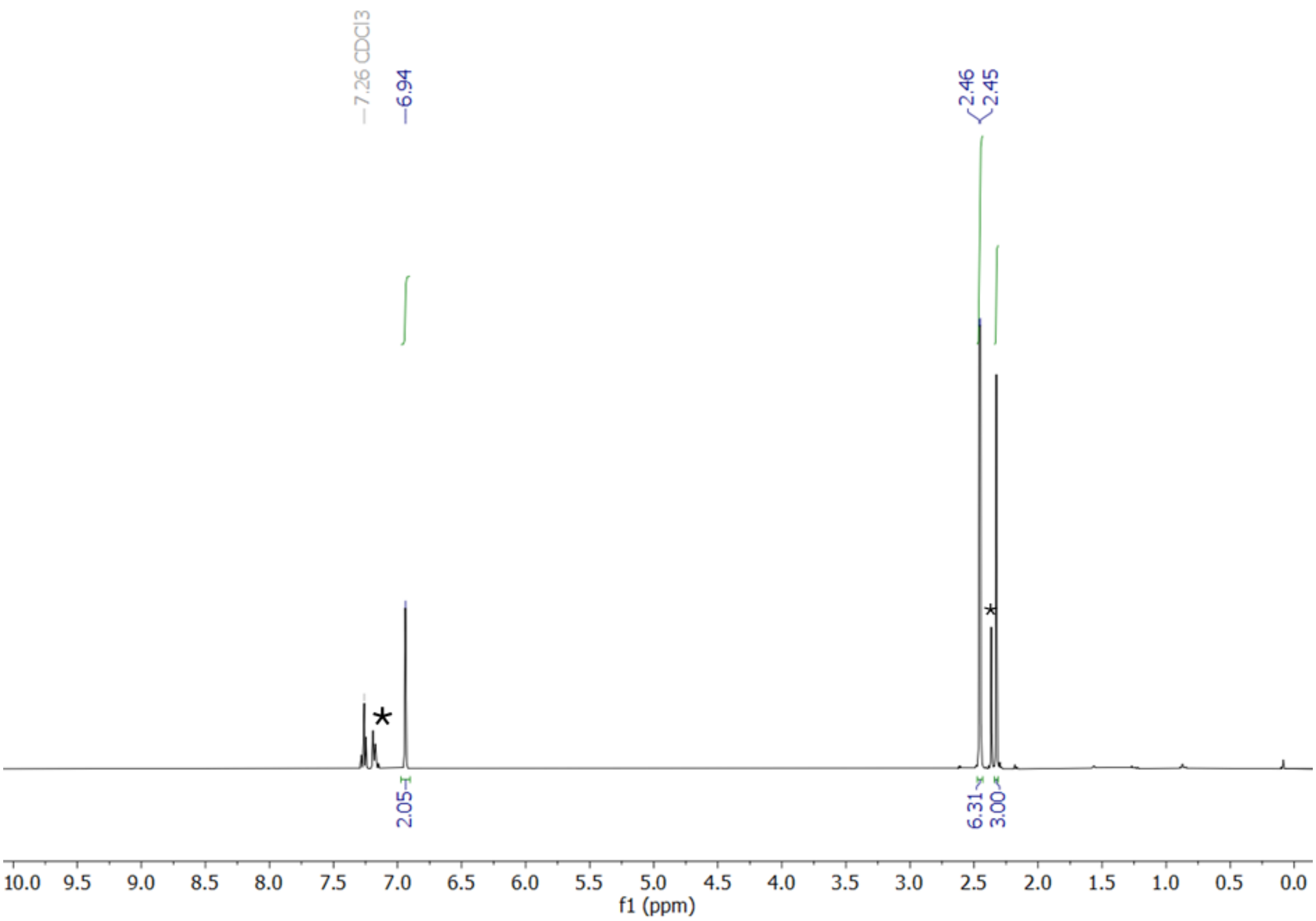

Figure S.4.28 ^{13}C NMR spectrum of 2,4,6-trimethylbenzoyl fluoride, **1b**.

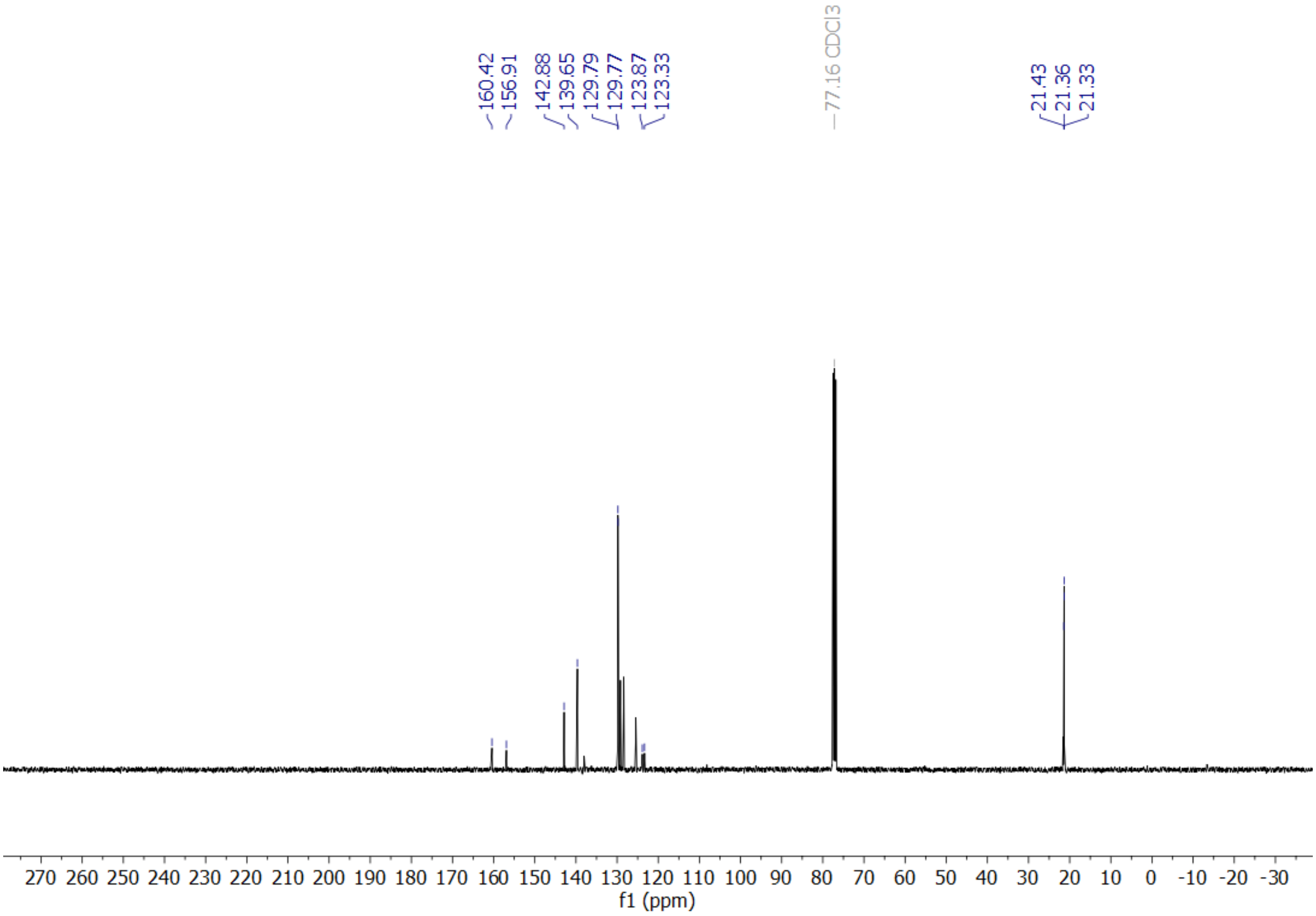

Figure S.4.29 ^{19}F NMR spectrum of 2,4,6-trimethylbenzoyl fluoride, **1b**.

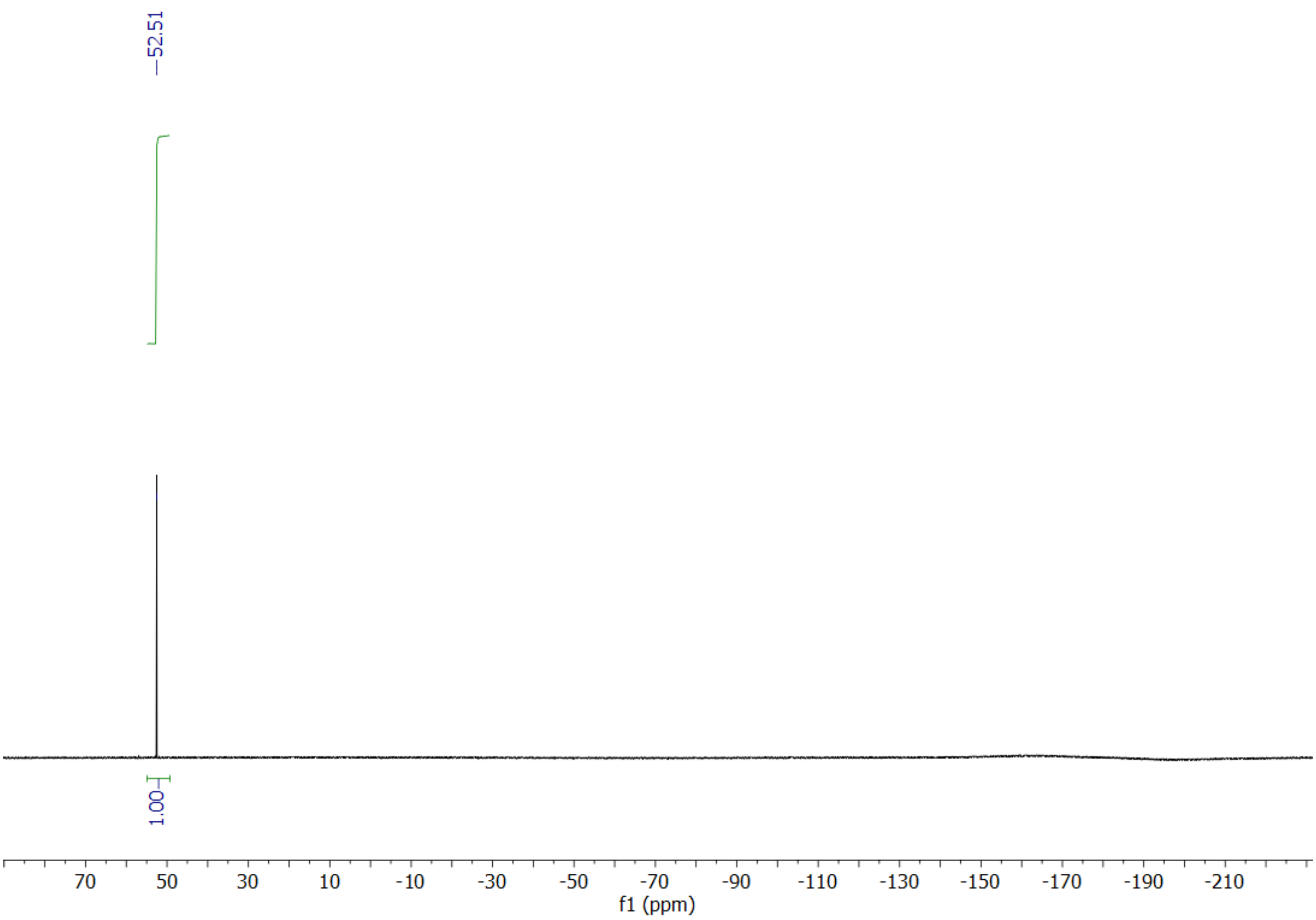

Figure S.4.30 ^{1}H NMR spectrum of 4-bromobenzoyl fluoride (*acetone), **1c**.

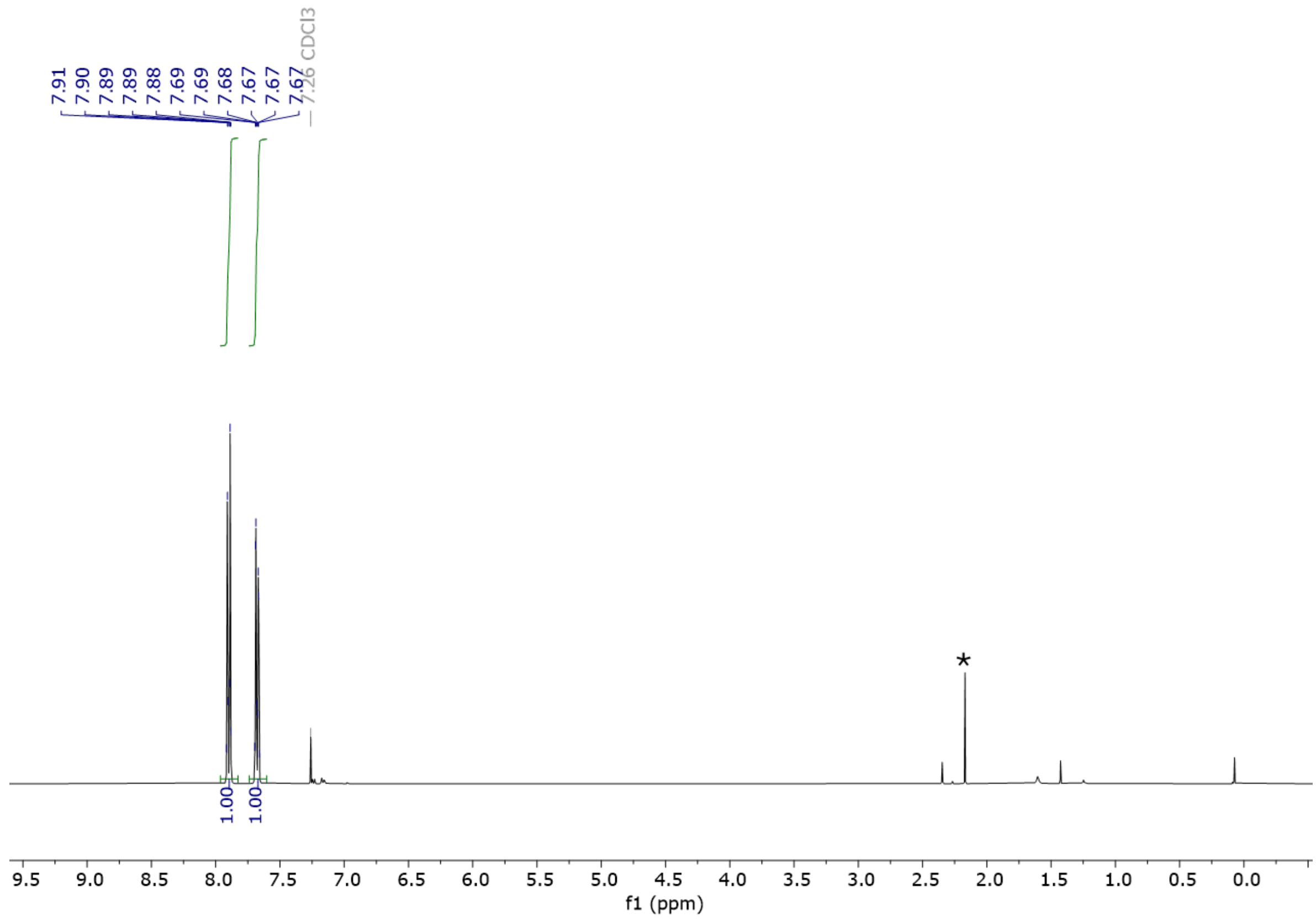

Figure S.4.31 ^{13}C{^{1}H} NMR spectrum of 4-bromobenzoyl fluoride, **1c**.

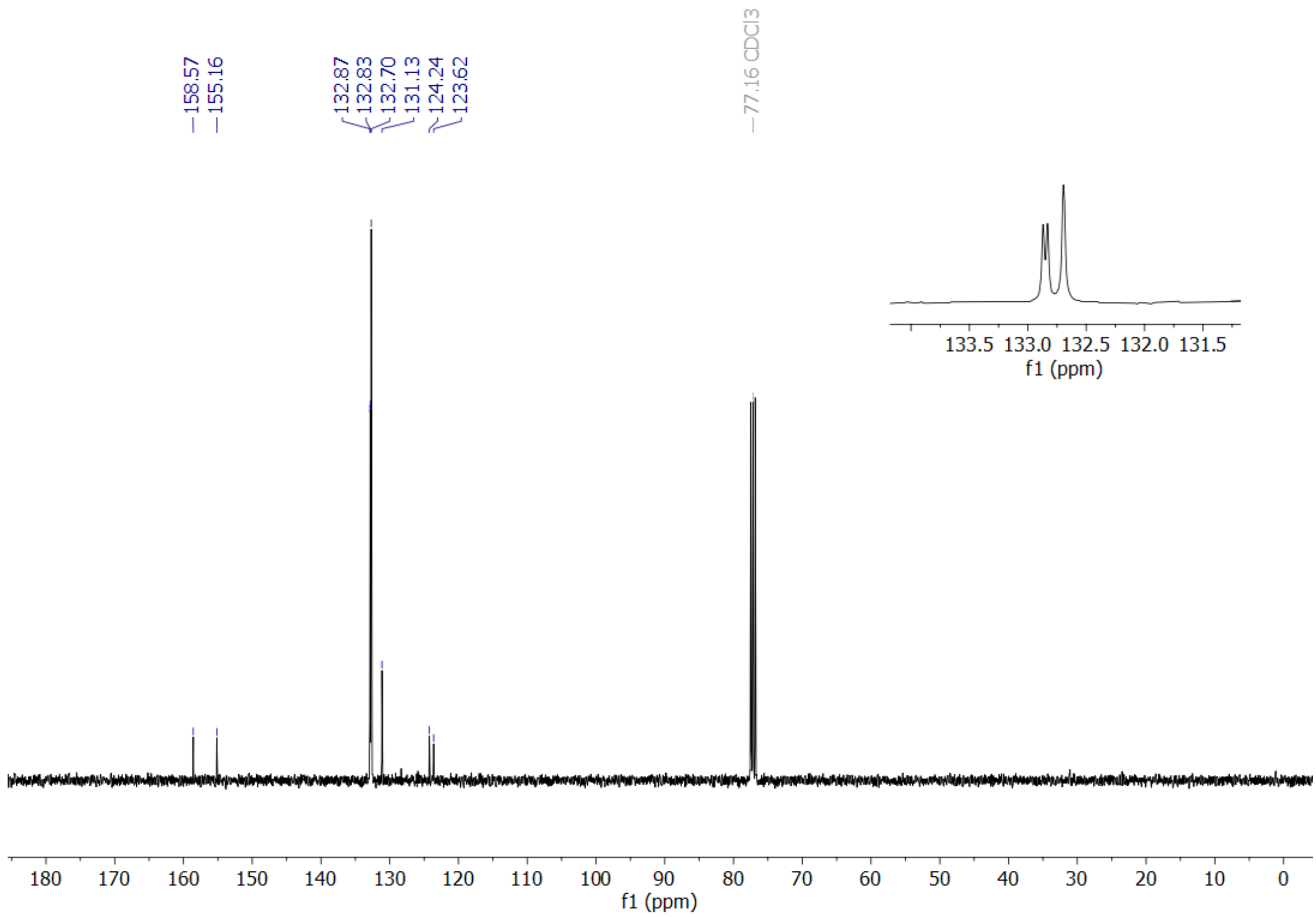

Figure S.4.32 ^{9}F NMR spectrum of 4-bromobenzoyl fluoride, **1c**.

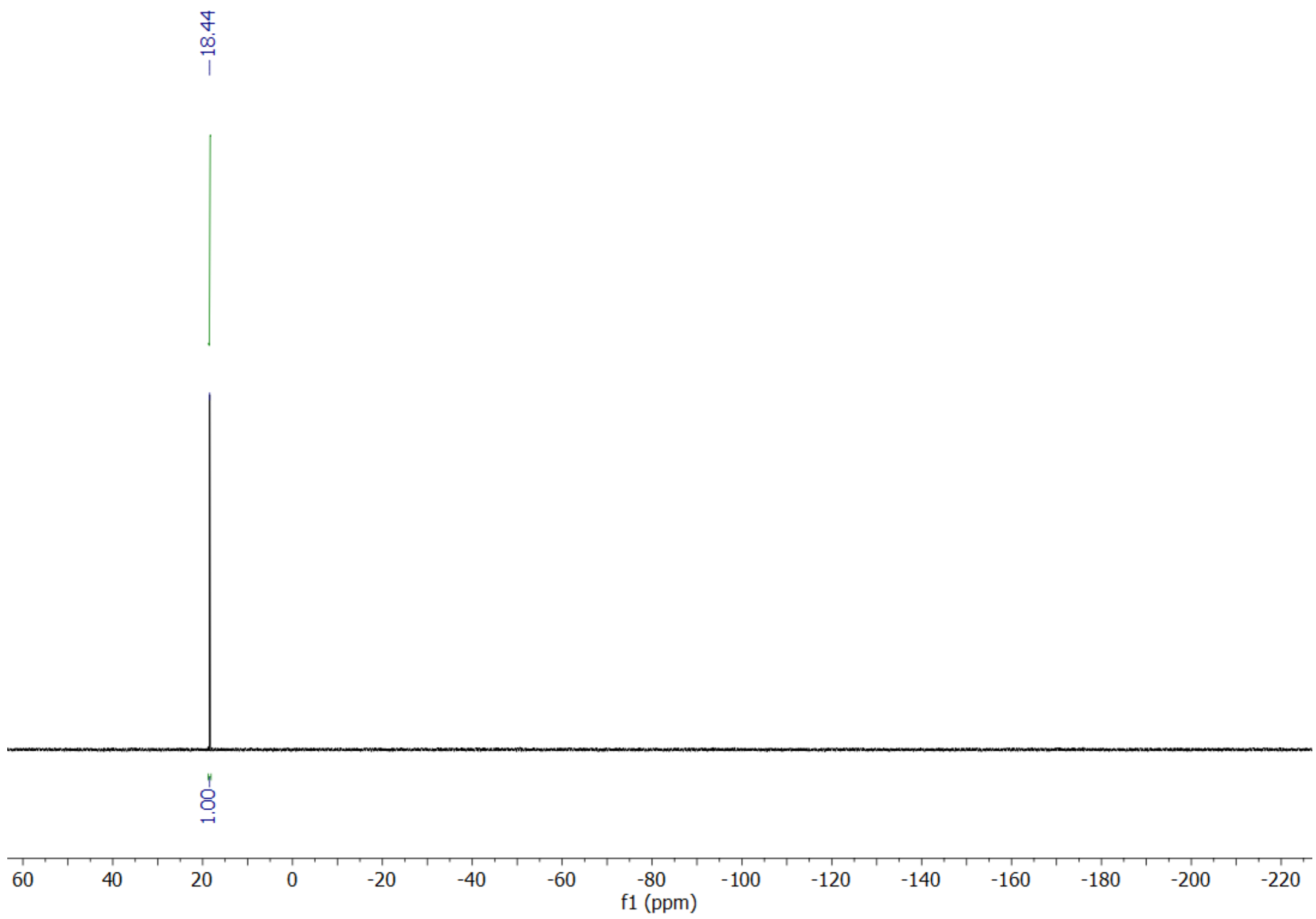

Figure S.4.33 ^{1}H NMR spectrum of crude reaction mixture of 2,6-difluorobenzoic anhydride (**1d**) with fluoroethane, catalysed by $BF_3{\cdot}OEt_2$ (10 mol%) and $BF_3{\cdot}PCy_3$ (10 mol%), generating 2,6-difluorobenzoyl fluoride in C_6D_6. Yields monitored by quantitative ^{19}F NMR spectroscopy against a fluorobenzene (2.7 µL, 0.029 mmol) internal standard.

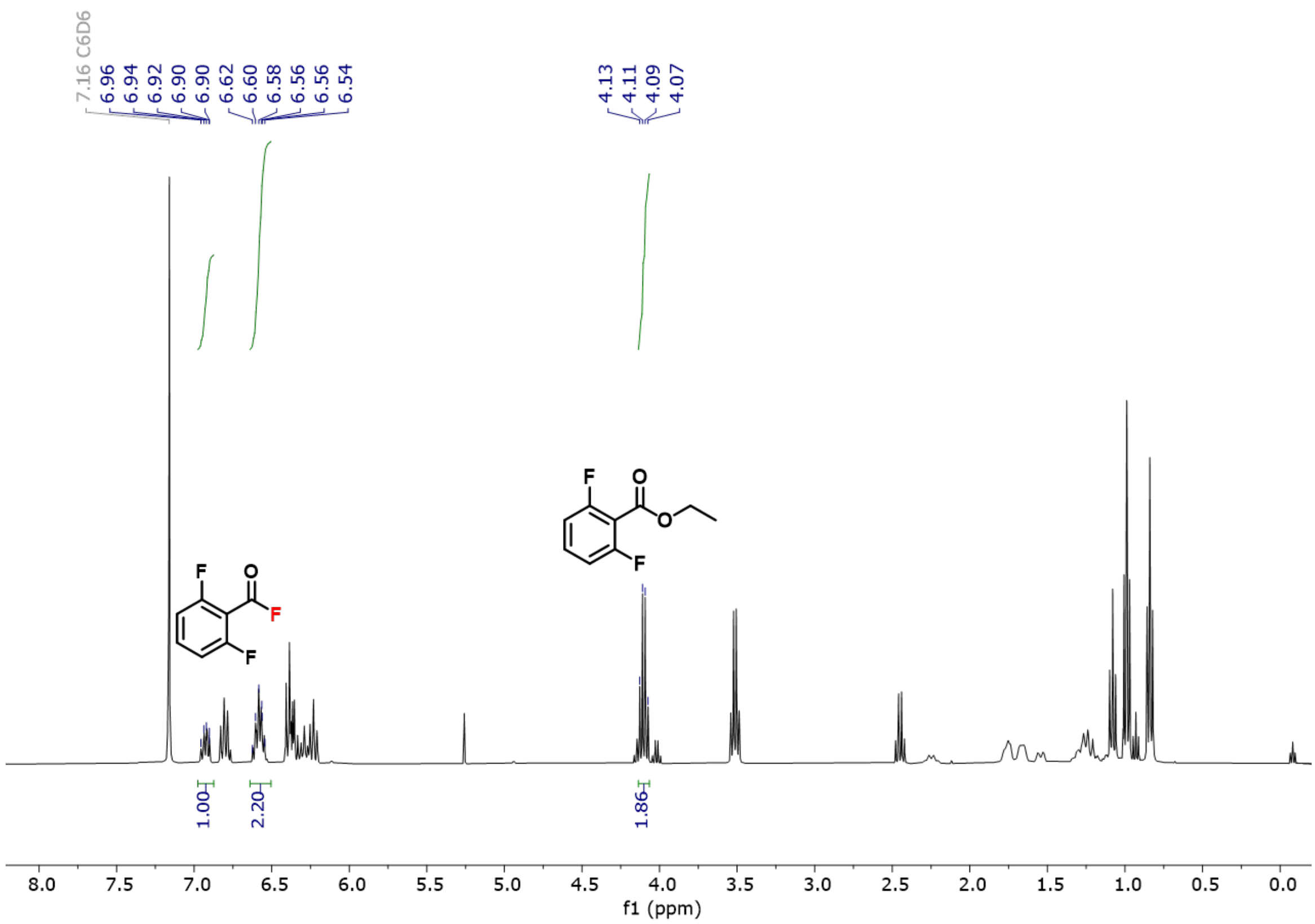

Figure S.4.34 ^{19}F NMR spectrum of 2,6-difluorobenzoyl fluoride (**1d**) of crude reaction mixture of 2,6-difluorobenzoic anhydride with fluoroethane, catalysed by $BF_3{\cdot}OEt_2$ (10 mol%) and $BF_3{\cdot}PCy_3$ (10 mol%), generating 2,6-difluorobenzoyl fluoride in C_6D_6. Yields monitored by quantitative ^{19}F NMR spectroscopy against a fluorobenzene (2.7 µL, 0.029 mmol) internal standard.

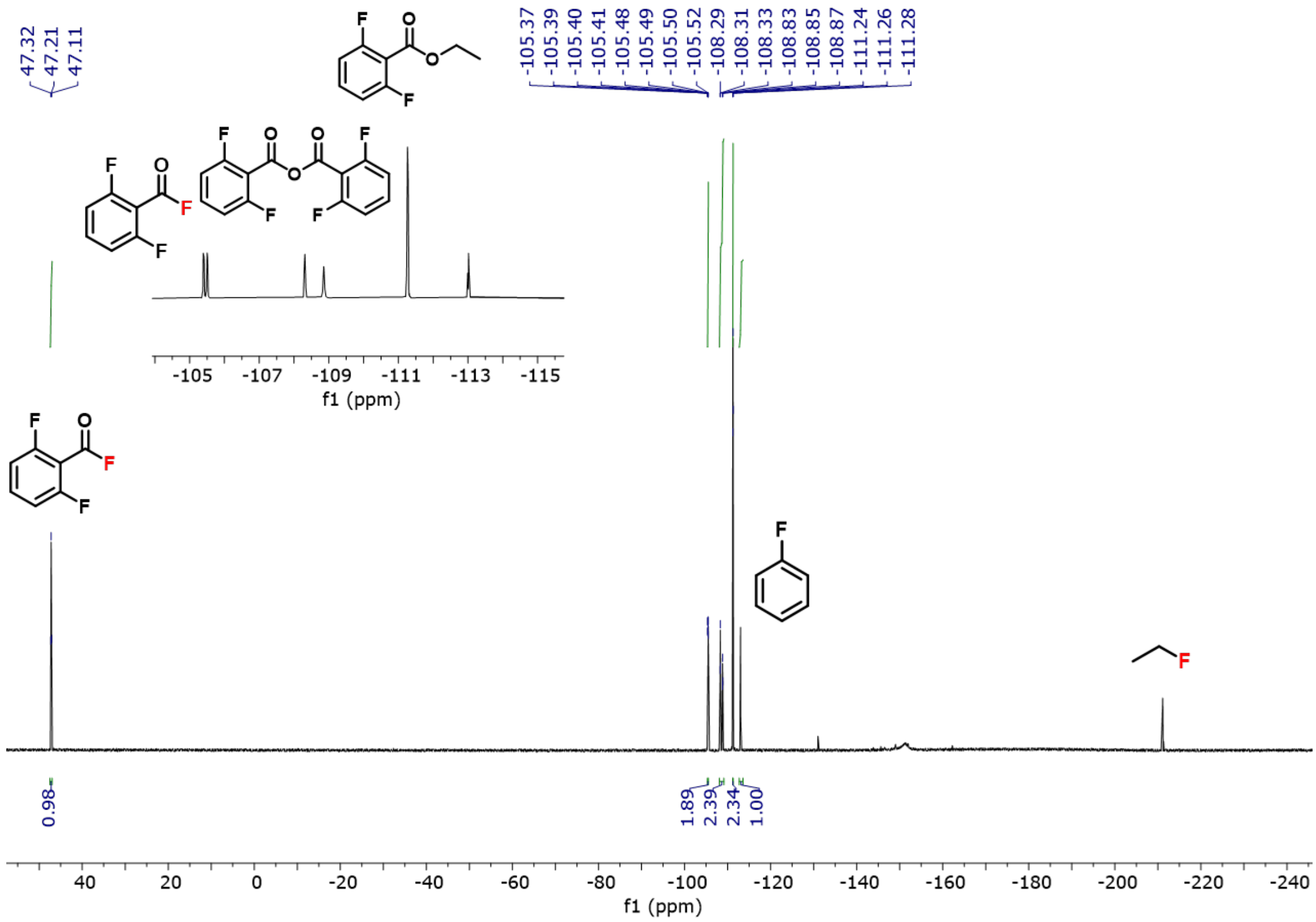

Figure S. 4.35 ^{1}H NMR spectrum of 4-trifluoromethylbenzoyl fluoride, **1e** (*toluene).

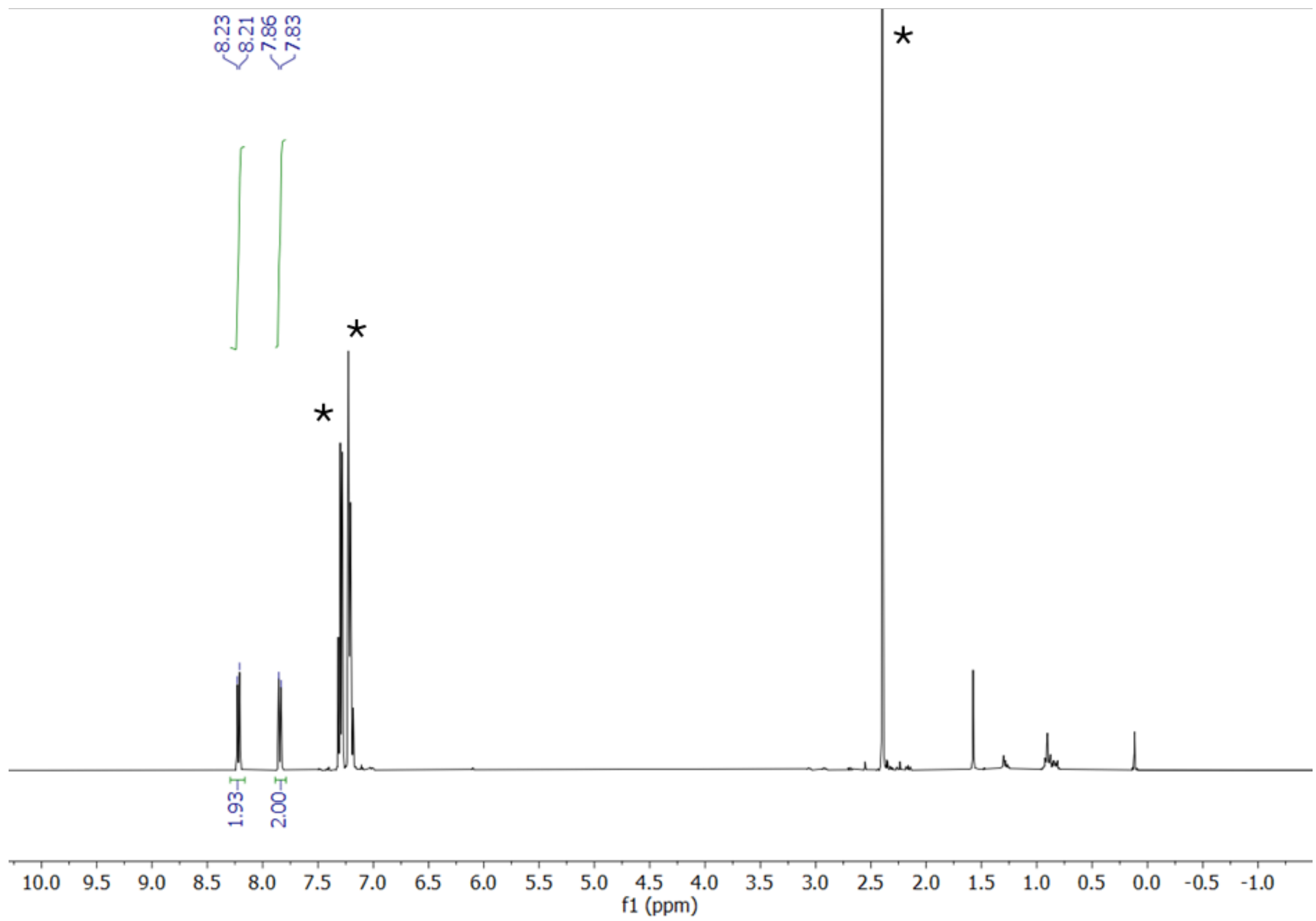

Figure S.4.36 $^{13}C\{^{1}H\}$ NMR spectrum of 4-trifluoromethylbenzoyl fluoride, **1e** (*toluene).

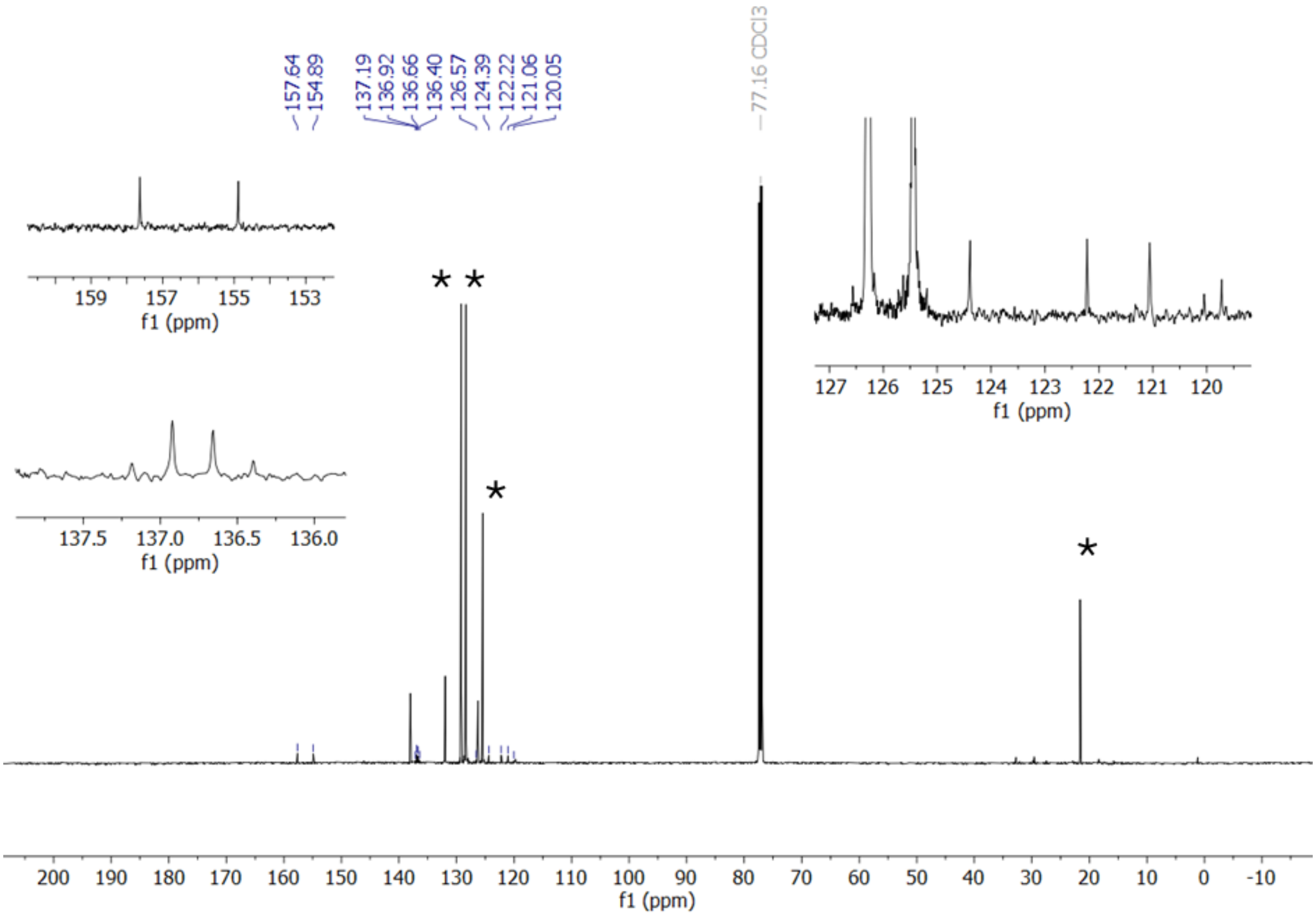

Figure S. 4.37 ^{19}F NMR spectrum of 2,4,6-trimethylbenzoyl fluoride, **1e**.

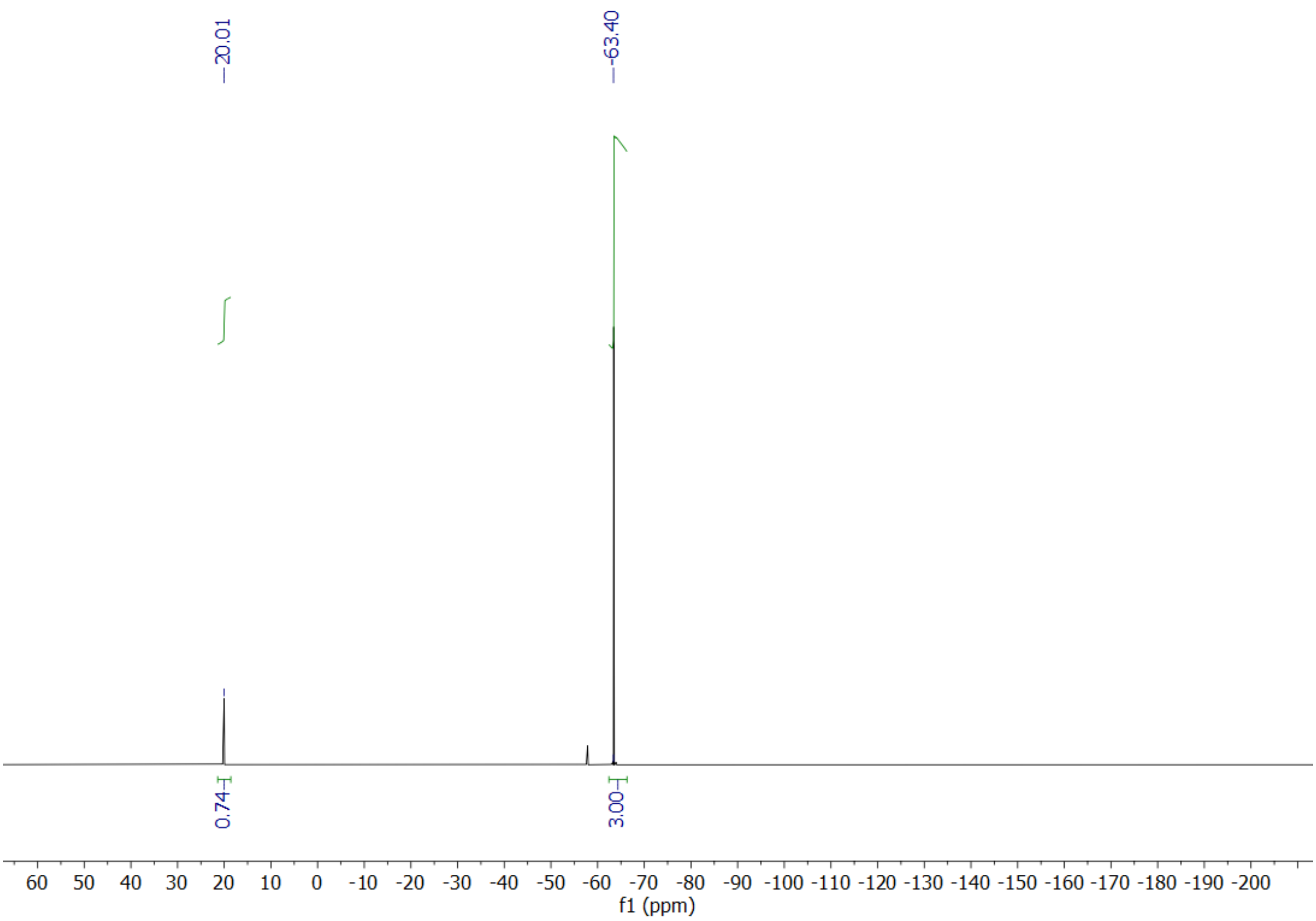

Figure S.4.38 ^{1}H NMR spectrum of crude reaction mixture of 4-trifluoromethyl benzoic anhydride with fluoroethane in C_6D_6, catalysed by $BF_3{\cdot}OEt_2$ (10 mol%) and $BF_3{\cdot}PCy_3$ (10 mol%), generating 4-trifluoromethylbenzoyl fluoride (**1e**). Yields monitored by quantitative ^{19}F NMR spectroscopy against a fluorobenzene (6.19 µL, 0.66 mmol) internal standard.

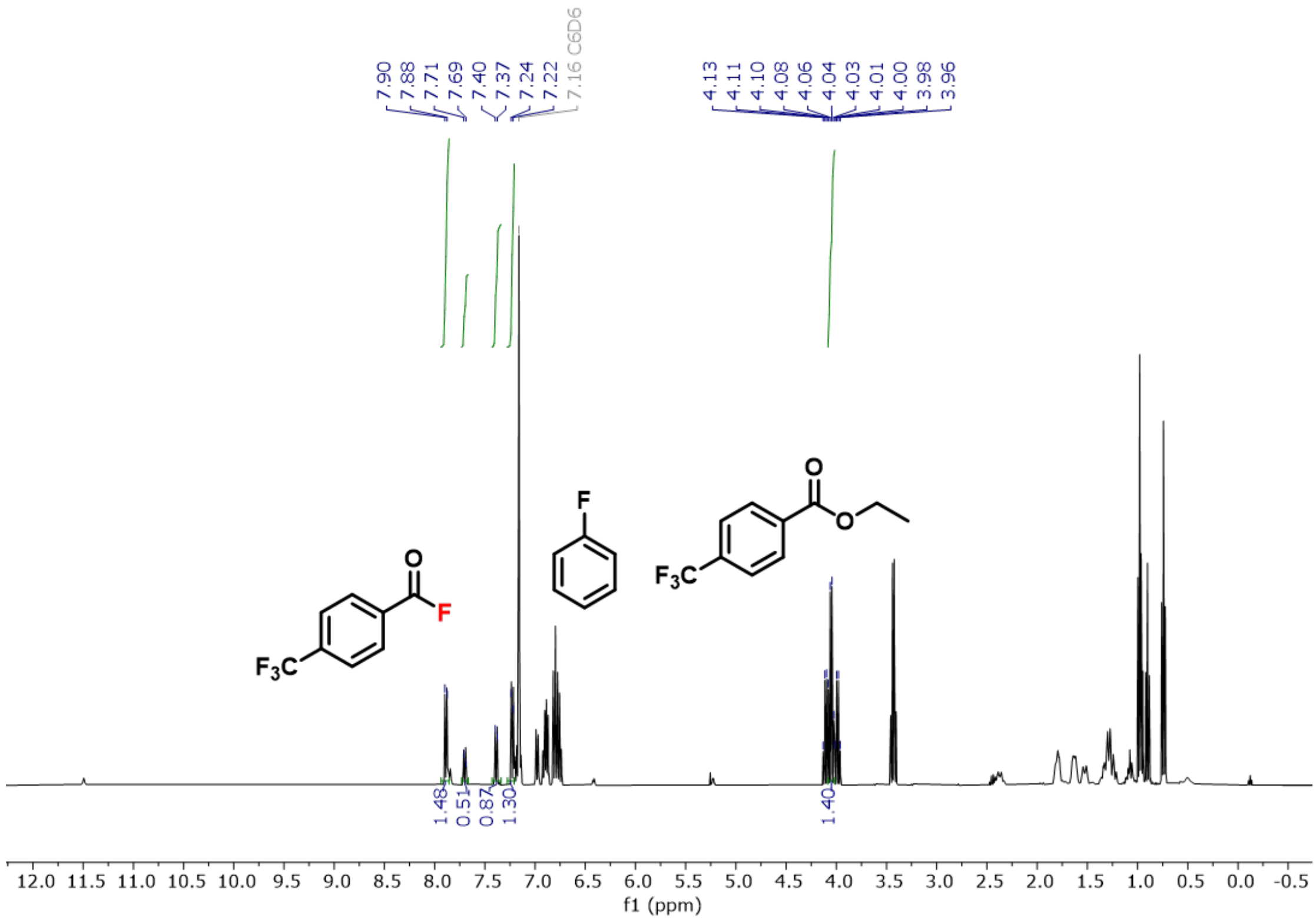

Figure S.4.39 ^{19}F NMR spectrum of crude reaction mixture of 4-trifluoromethylbenzoic anhydride with fluoroethane in C_6D_6, catalysed by $BF_3{\cdot}OEt_2$ (10 mol%) and $BF_3{\cdot}PCy_3$ (10 mol%), generating 4-trifluoromethylbenzoyl fluoride, **1e**. Yields monitored by quantitative ^{19}F NMR spectroscopy against a fluorobenzene (6.19 µL, 0.66 mmol) internal standard.

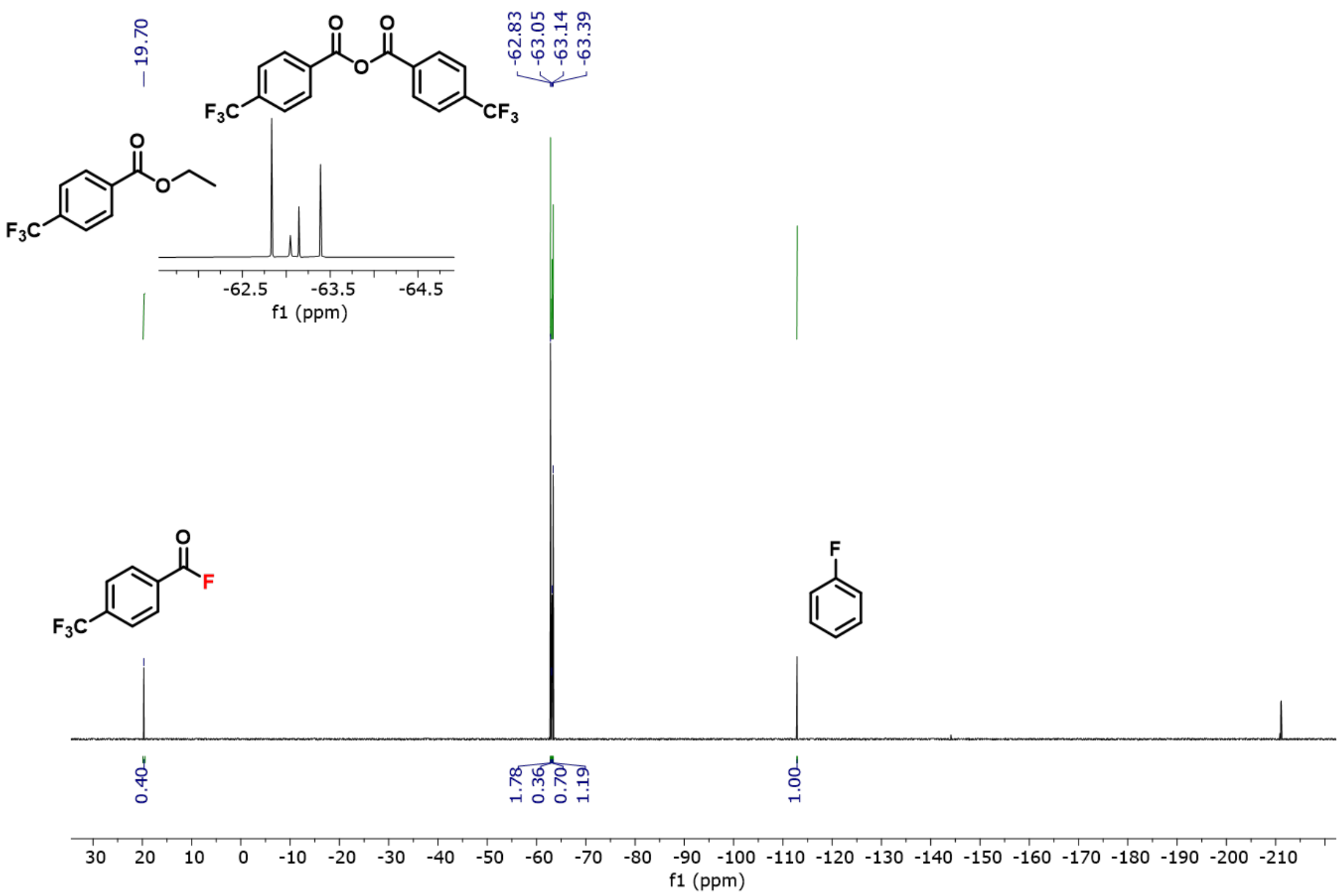

Figure S.4.40 ^{1}H NMR spectrum of 4-methoxybenzoyl fluoride, **1f**,

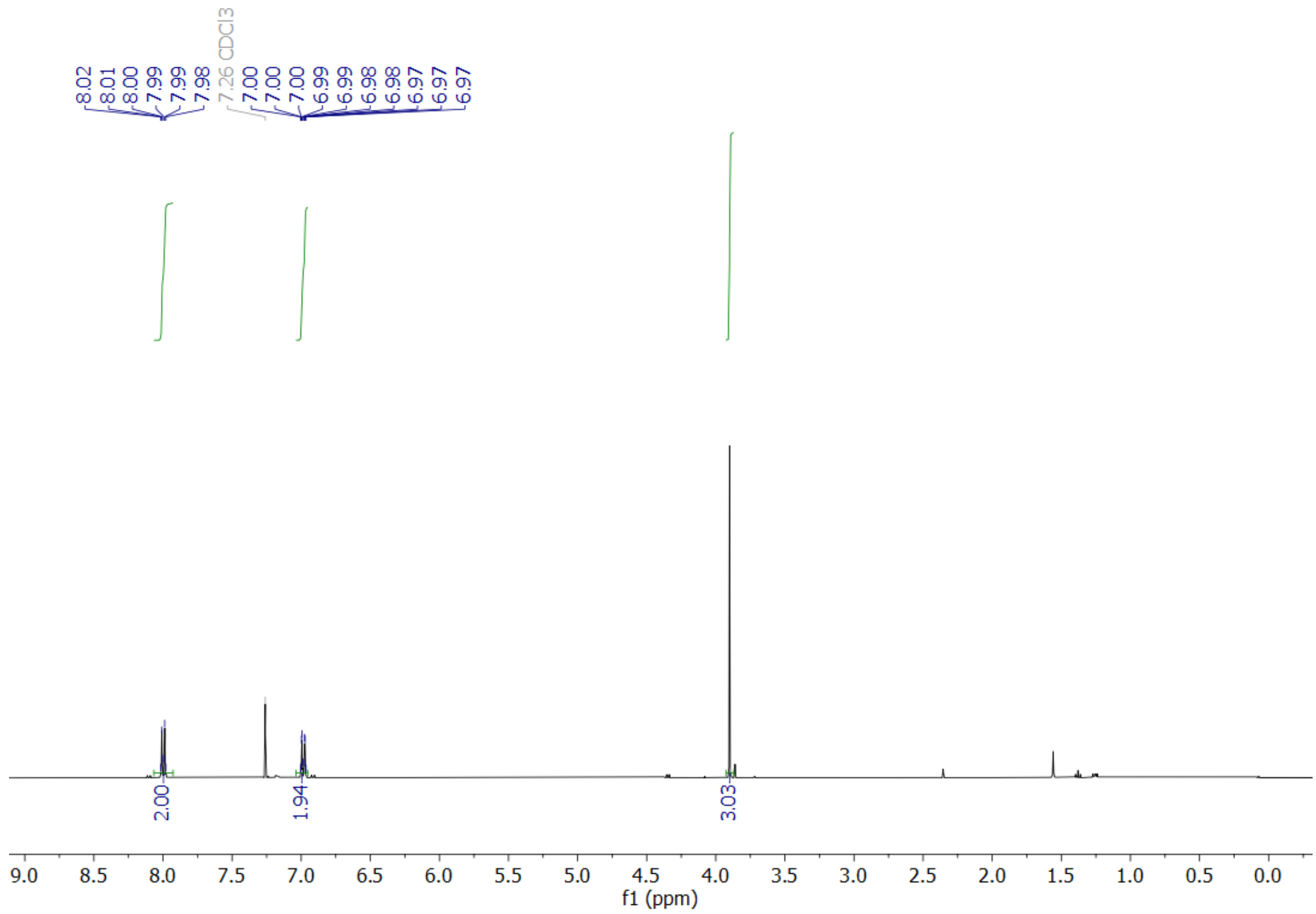

Figure S.4.41 $^{13}C\{^{1}H\}$ NMR spectrum of 4-methoxybenzoyl fluoride, **1f**.

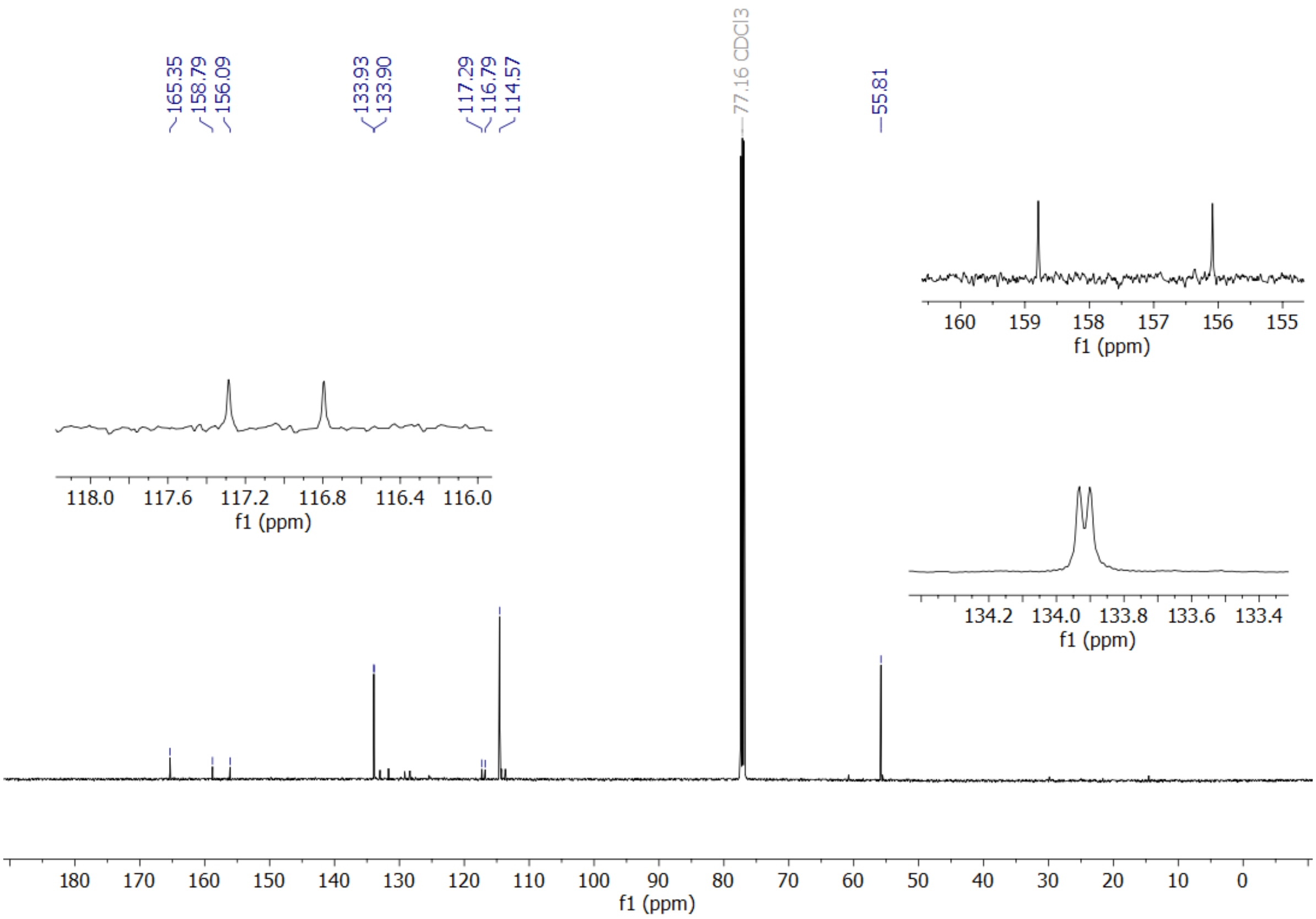

Figure S.4.42 ^{19}F NMR spectrum of 4-methoxybenzoyl fluoride, **1f**.

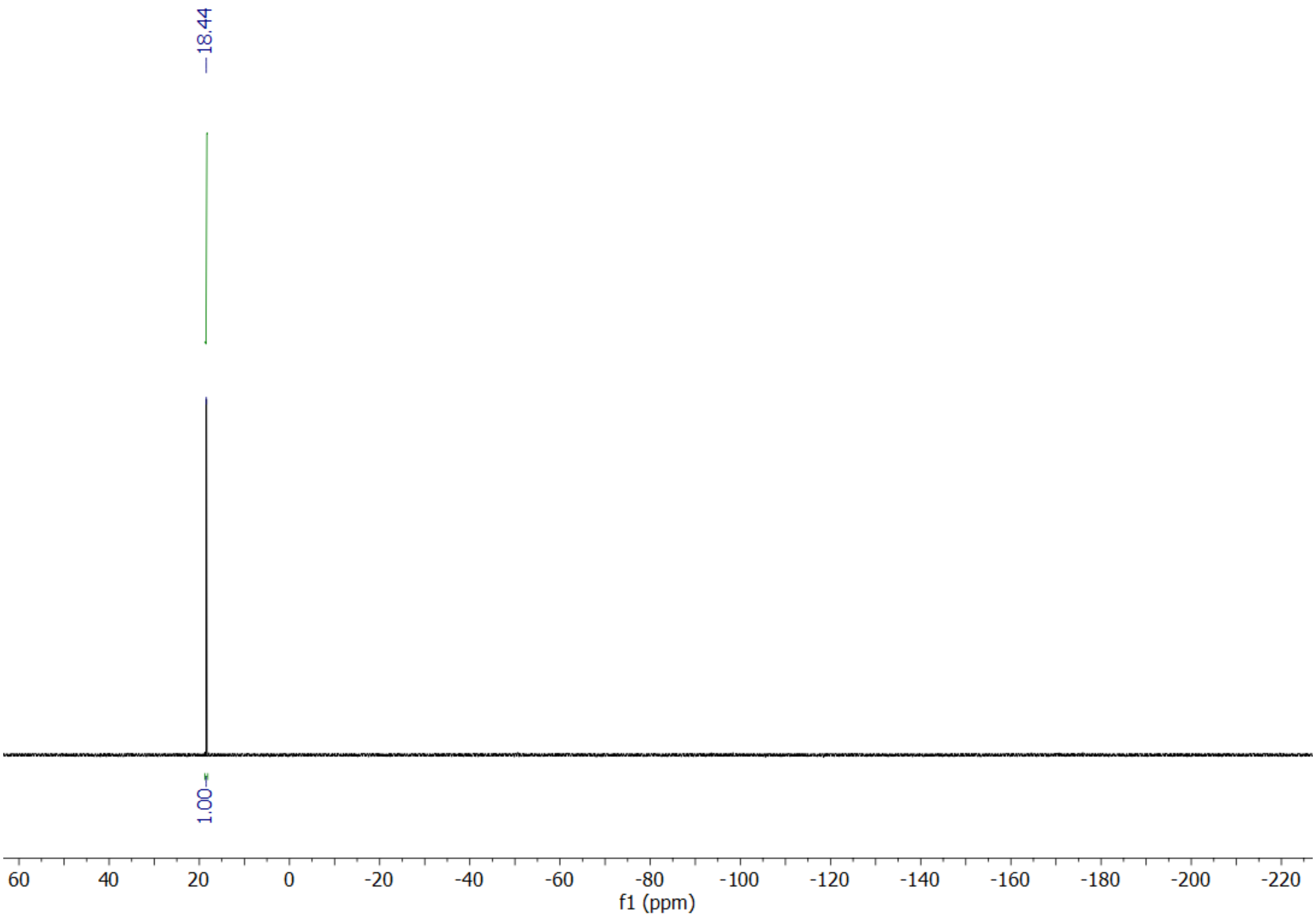

Figure S.4.43 ^{1}H NMR spectrum of naphthoic fluoride, **1g**.

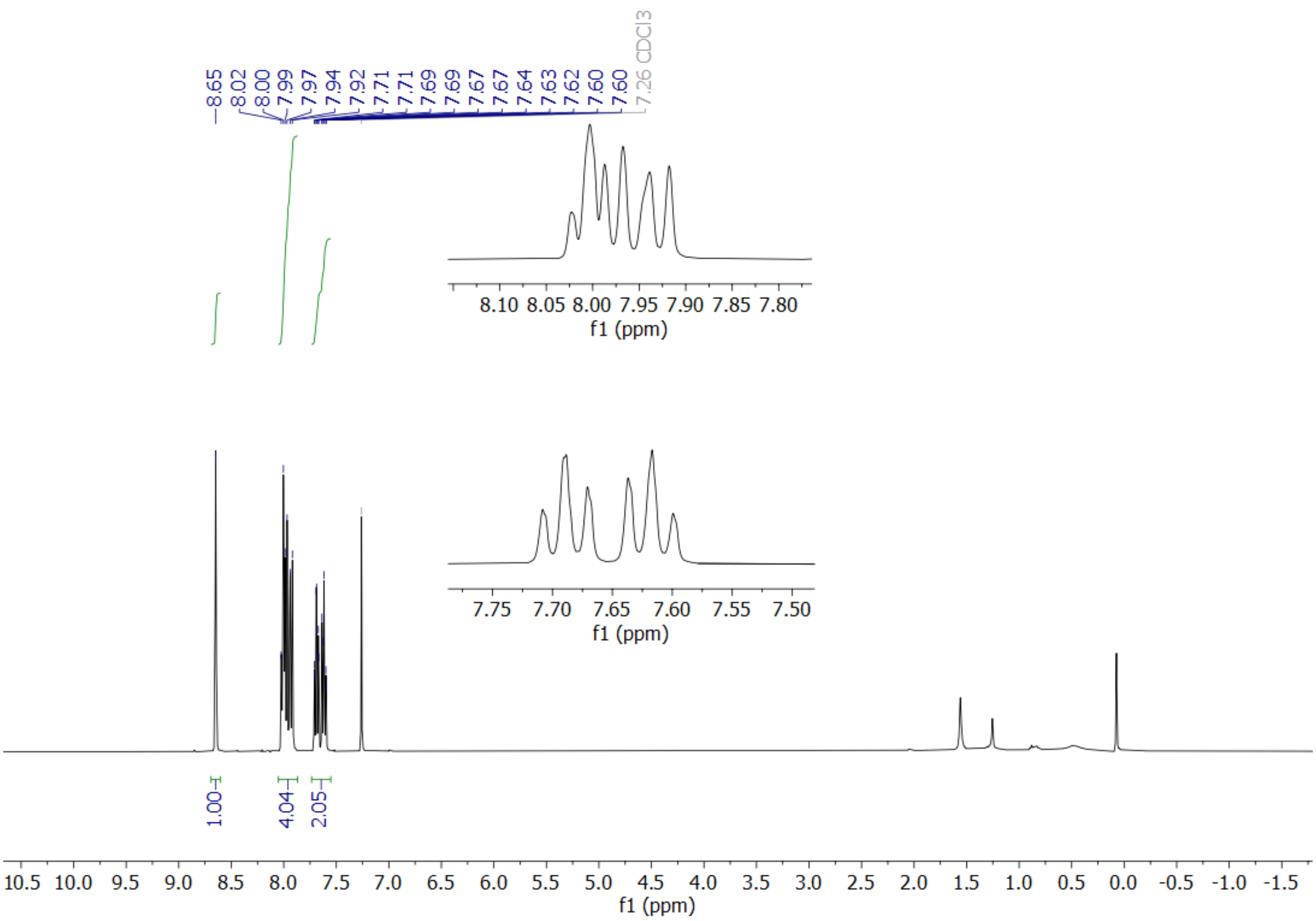

Figure S.4.44 $^{13}C\{^{1}H\}$ NMR spectrum of naphthoic fluoride, **1g**.

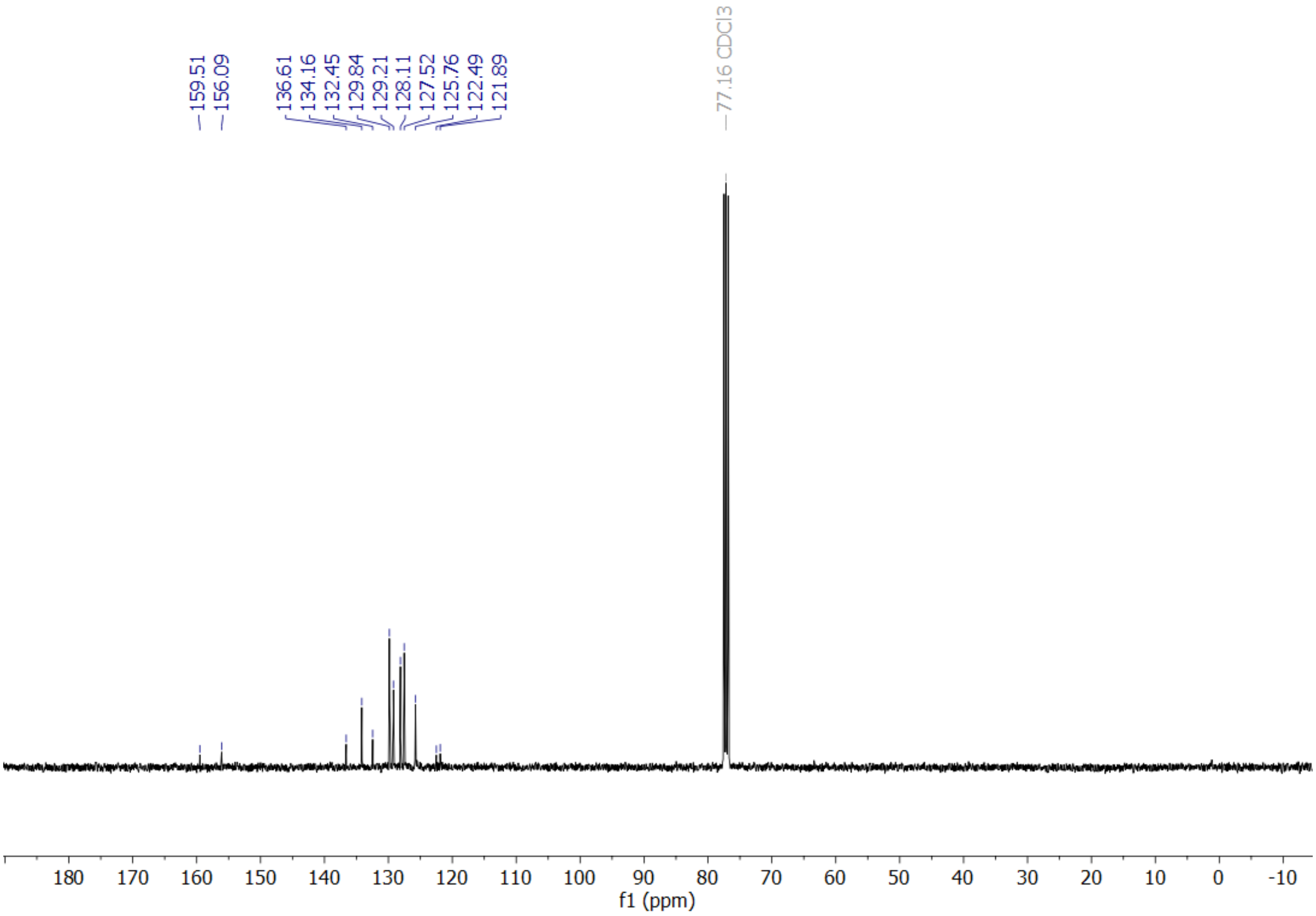

Figure S.4.45 ^{19}F NMR spectrum of naphthoic fluoride, **1g**.

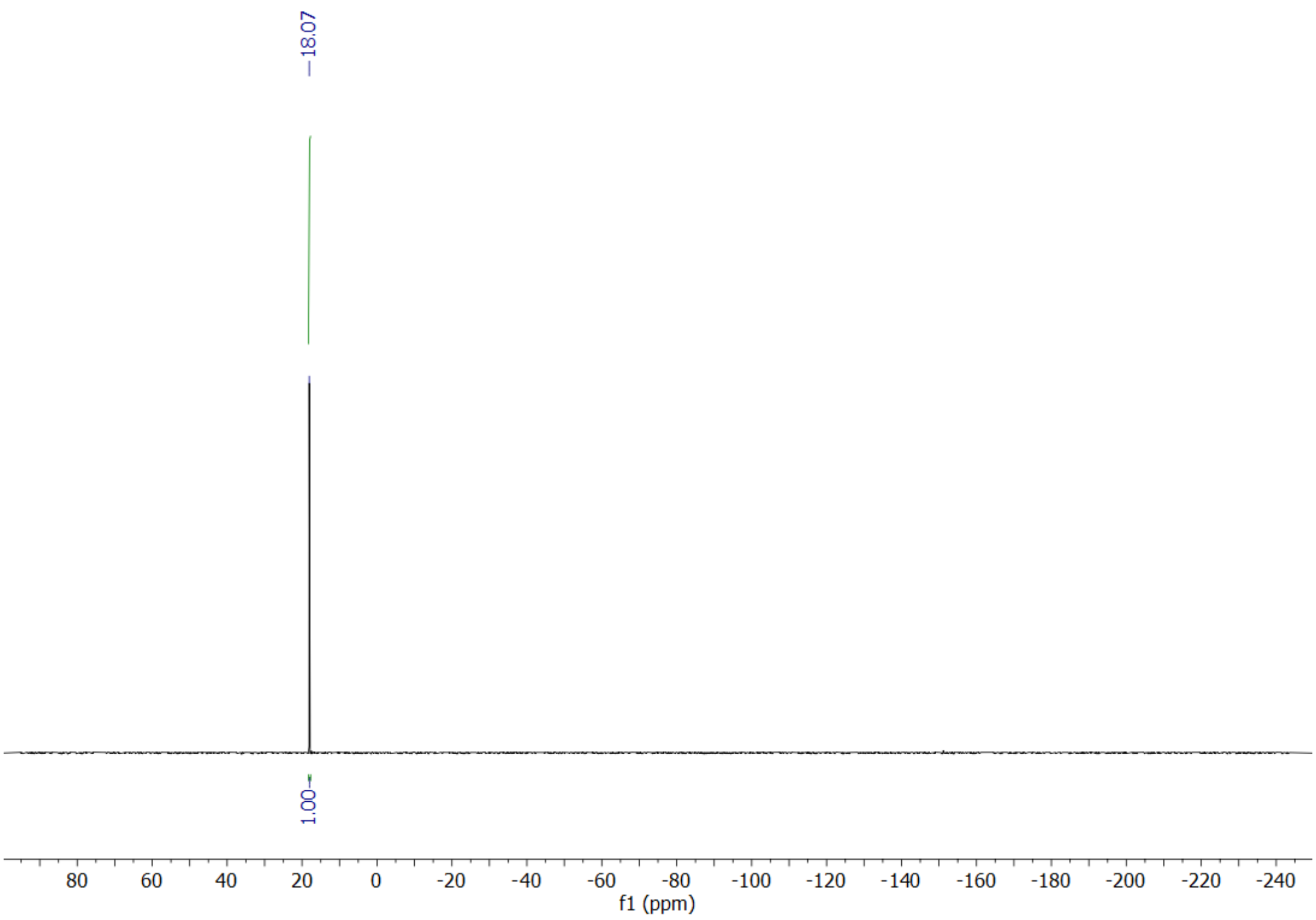

Figure S.4.46 ^{19}F NMR spectrum of crude reaction mixture of acetic anhydride with fluoroethane catalysed by $BF_3{\cdot}OEt_2$ (10 mol%) and $BF_3{\cdot}PCy_3$ (10 mol%) in toluene, generating acetyl fluoride, **1h**. Yields monitored by quantitative ^{19}F NMR spectroscopy against a fluorobenzene internal standard.

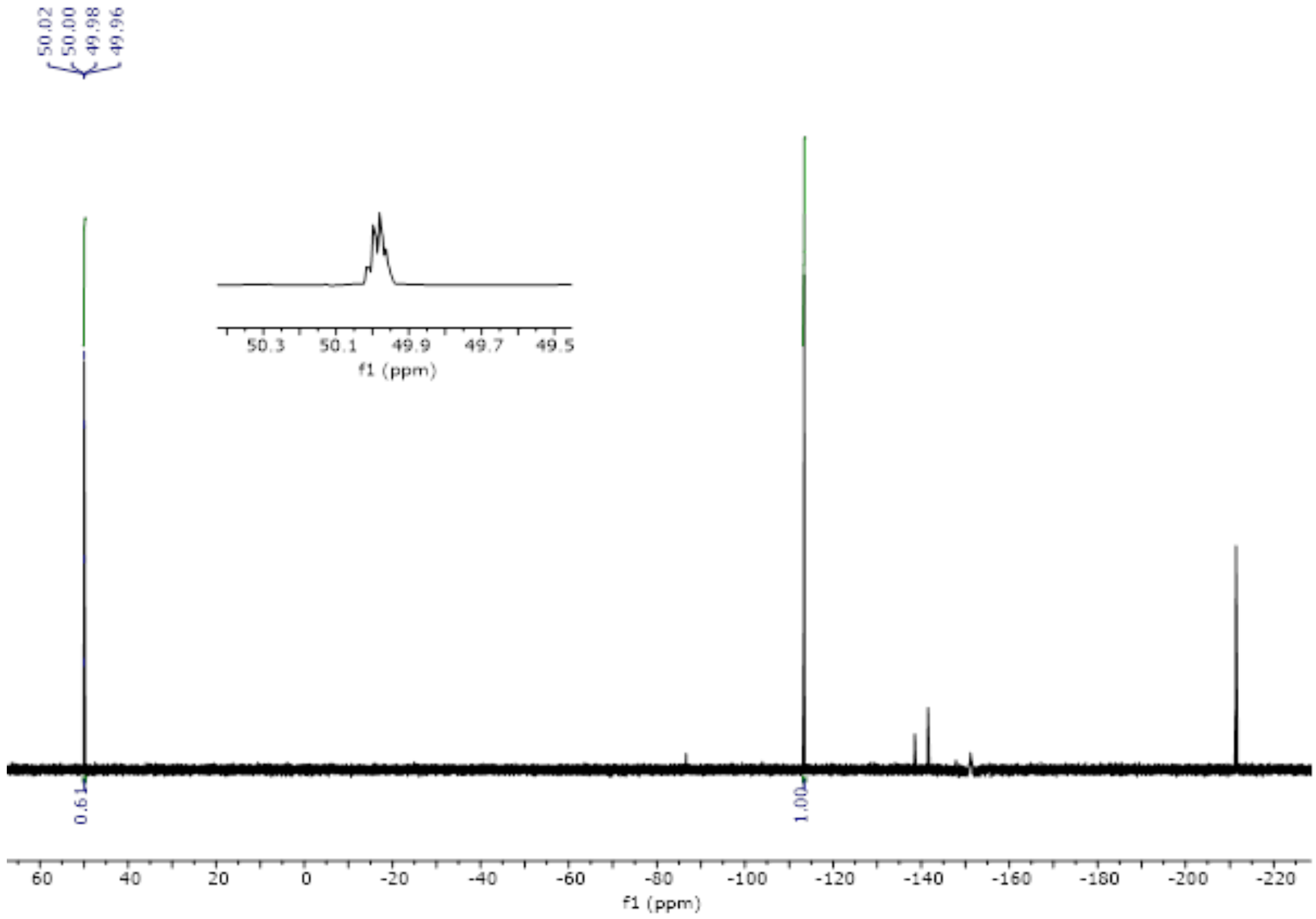

Figure S.4.47 ^{1}H NMR spectrum of tricyclohexyl(ethyl)phosphonium tetrafluoroborate and tricyclohexylphosphonium tetrafluoroborate (*Et_2O).

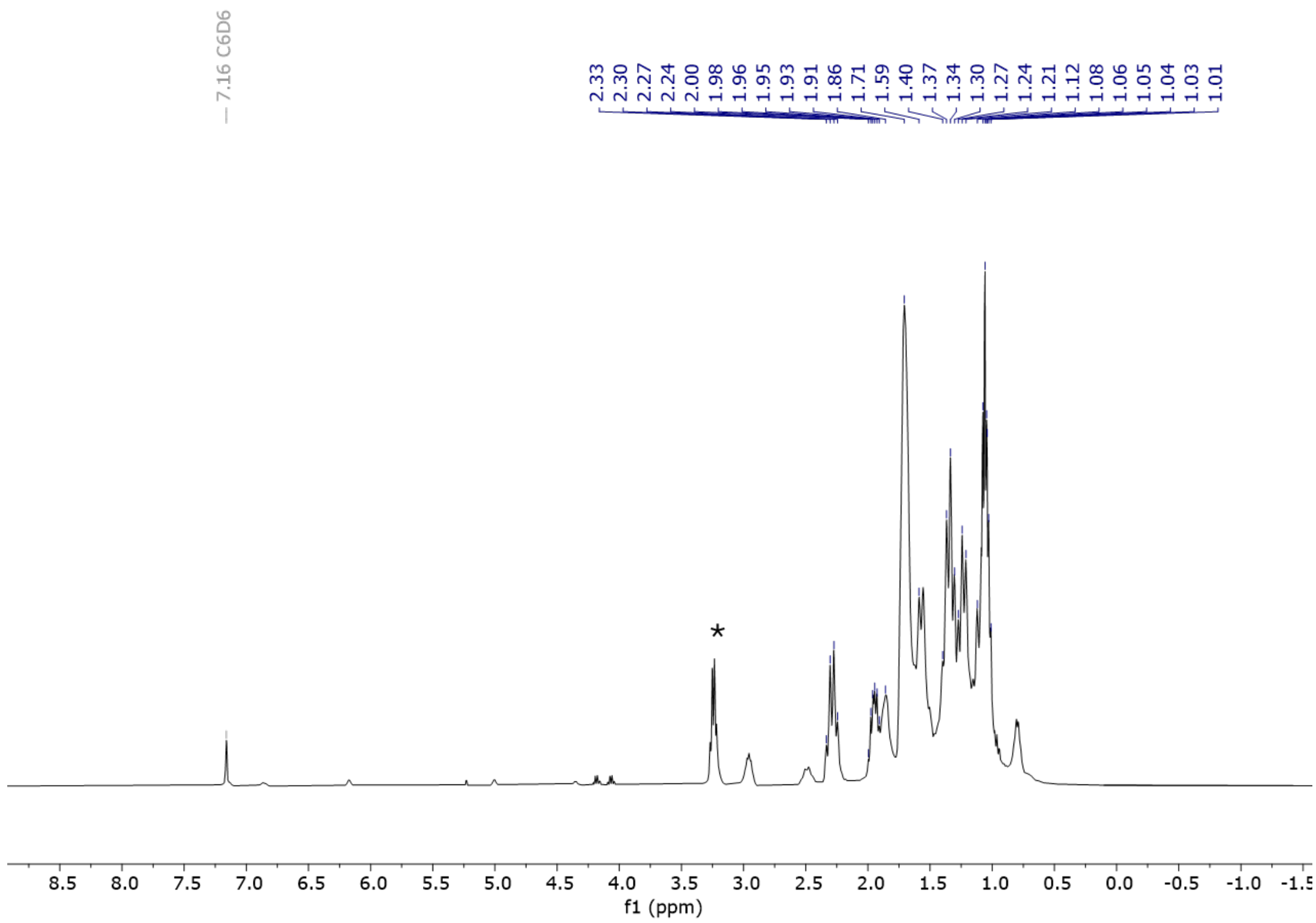

Figure S.4.48 ^{13}C NMR spectrum of tricyclohexyl(ethyl)phosphonium tetrafluoroborate and tricyclohexylphosphonium tetrafluoroborate (*Et_2O).

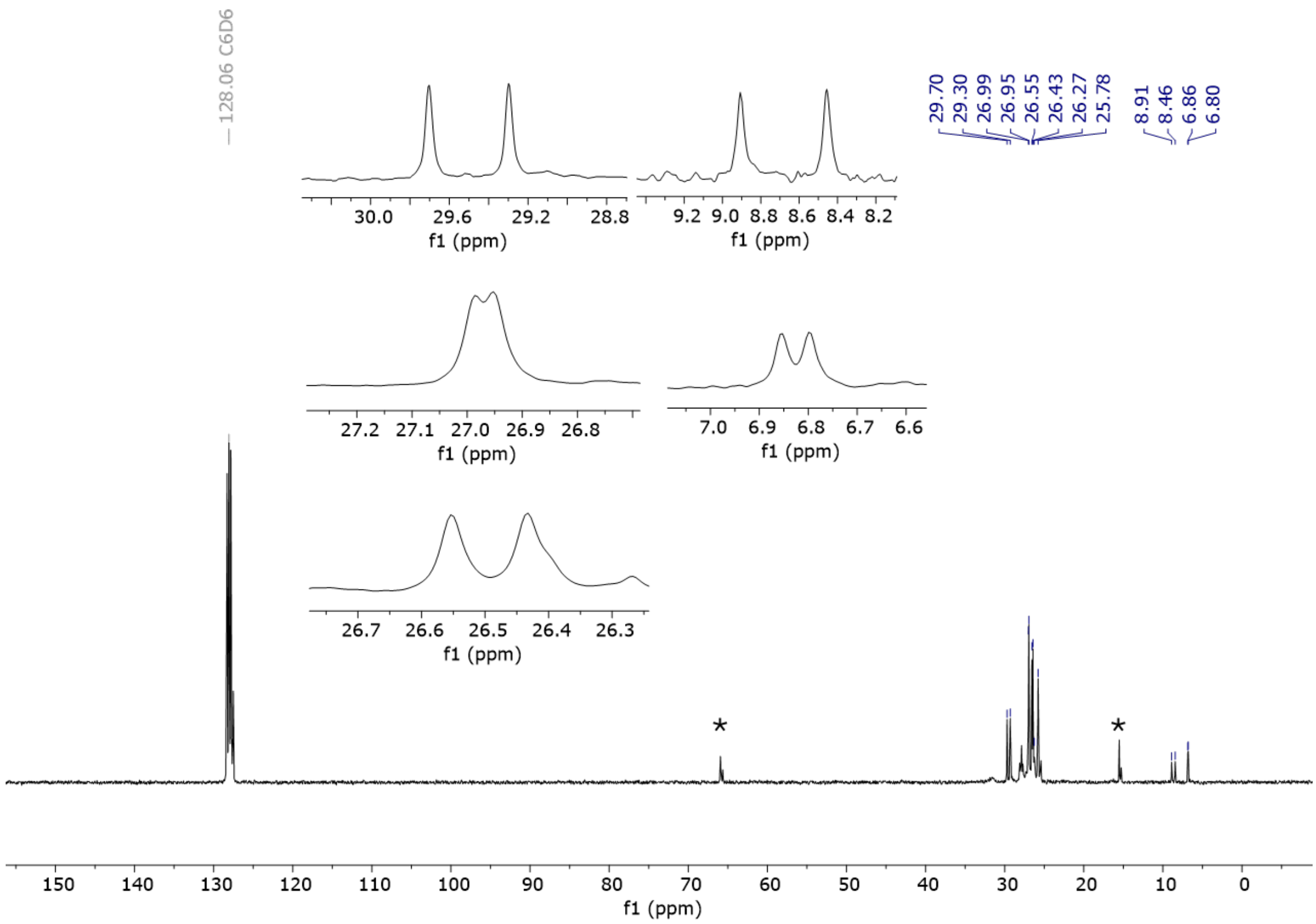

Figure S.4.49 ^{19}F NMR spectrum of tricyclohexyl(ethyl)phosphonium tetrafluoroborate and tricyclohexylphosphonium tetrafluoroborate.

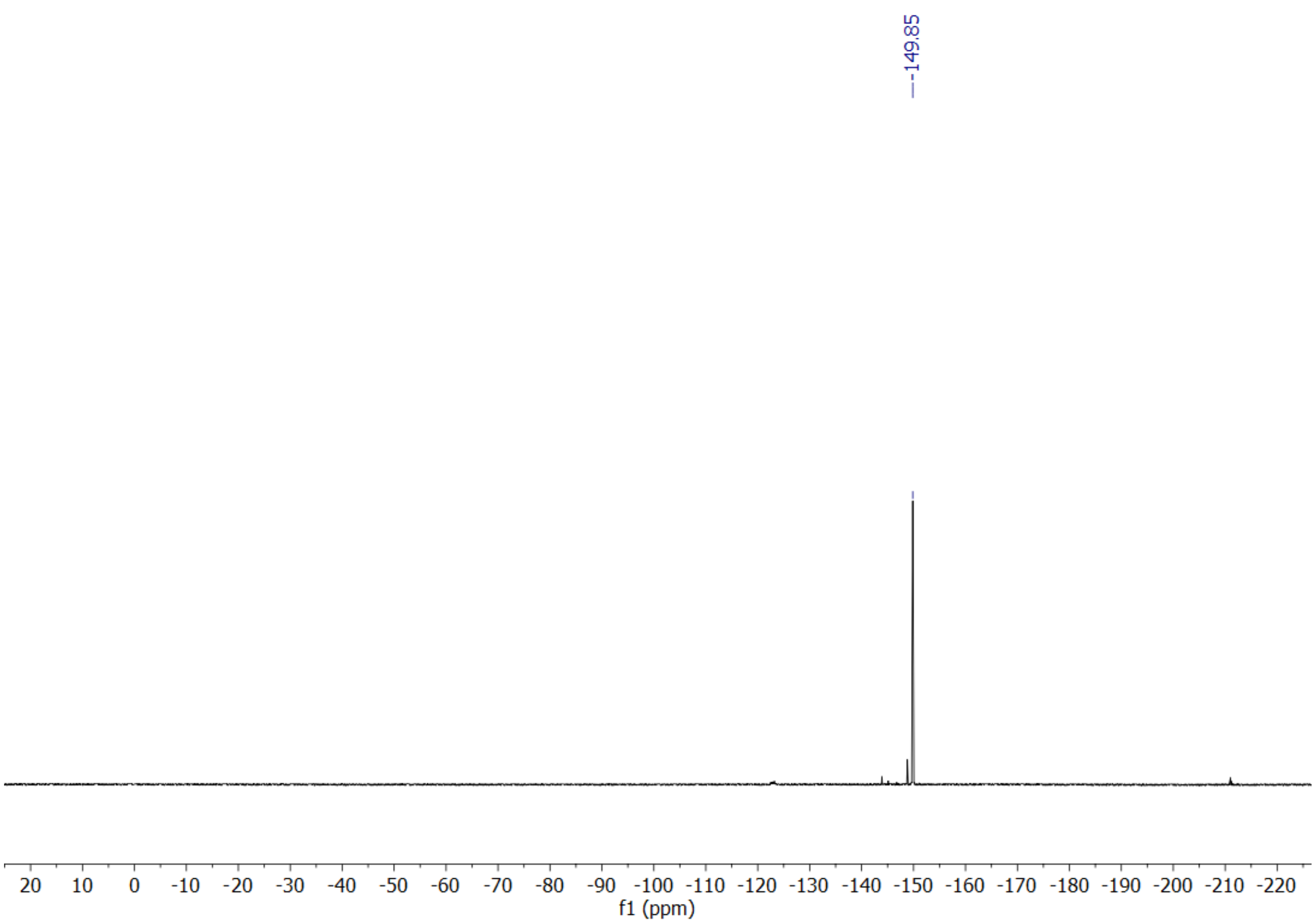

Figure S.4.50 $^{31}P\{^{1}H\}$ NMR spectrum of tricyclohexyl(ethyl)phosphonium tetrafluoroborate and tricyclohexylphosphonium tetrafluoroborate (*$Cy_3PH{\cdot}BF_4$)

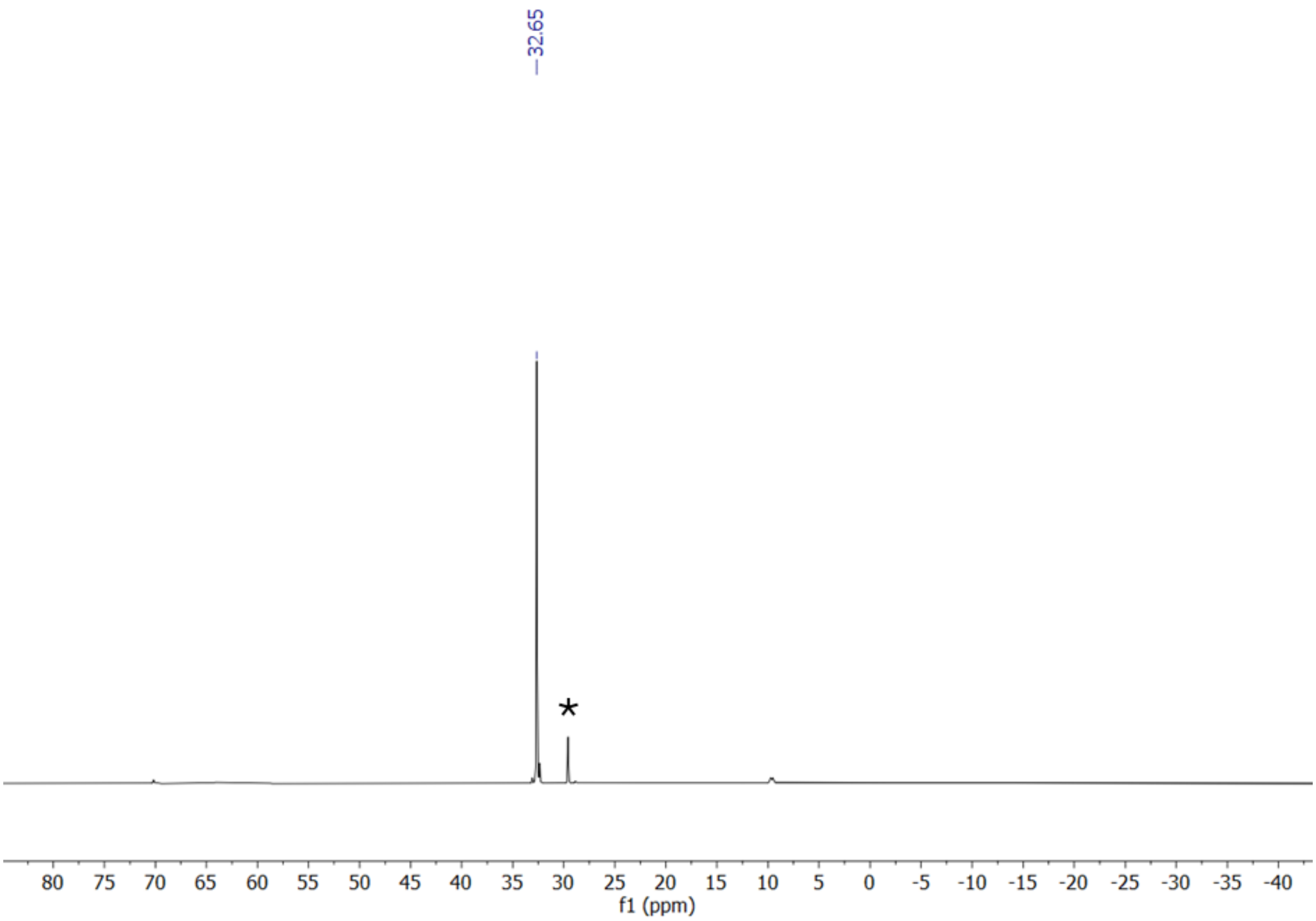

Figure S.4.51 ^{31}P NMR spectrum of tricyclohexyl(ethyl)phosphonium tetrafluoroborate and tricyclohexylphosphonium tetrafluoroborate (*$Cy_3PH{\cdot}BF_4$).

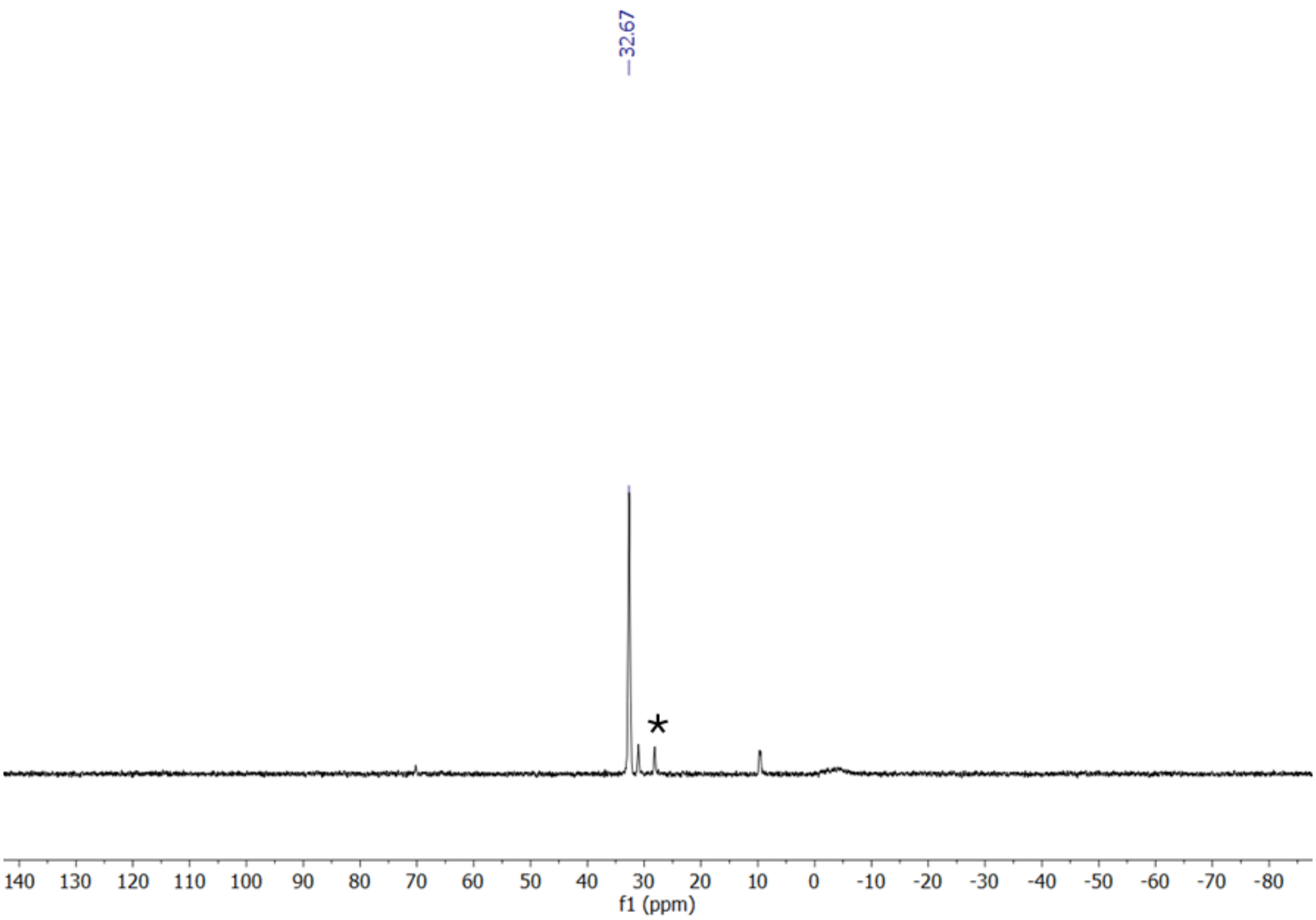

Figure S.4.52 ^{11}B NMR spectrum of tricyclohexyl(ethyl)phosphonium tetrafluoroborate and tricyclohexylphosphonium tetrafluoroborate.

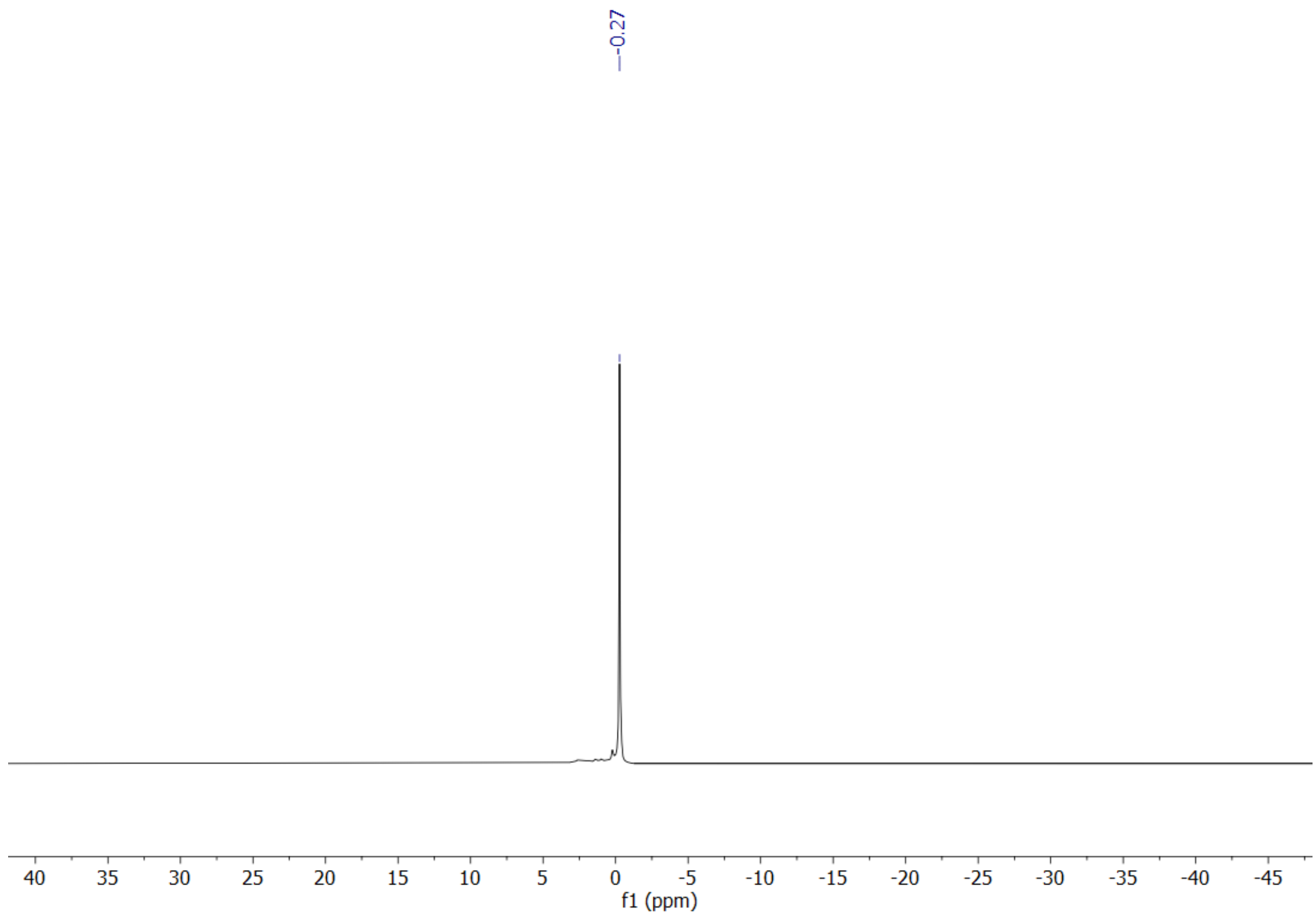

Figure S.4.53 ^{1}H NMR spectrum of ethyl benzoate formed within the control reaction of using 1-fluoroheptane as the fluorine donor. Insets are of zoomed in resonances of ethyl benzoate and heptyl benzoate. 1,4-difluorobenzene was used as an internal standard. (**Scheme S.4.14**)

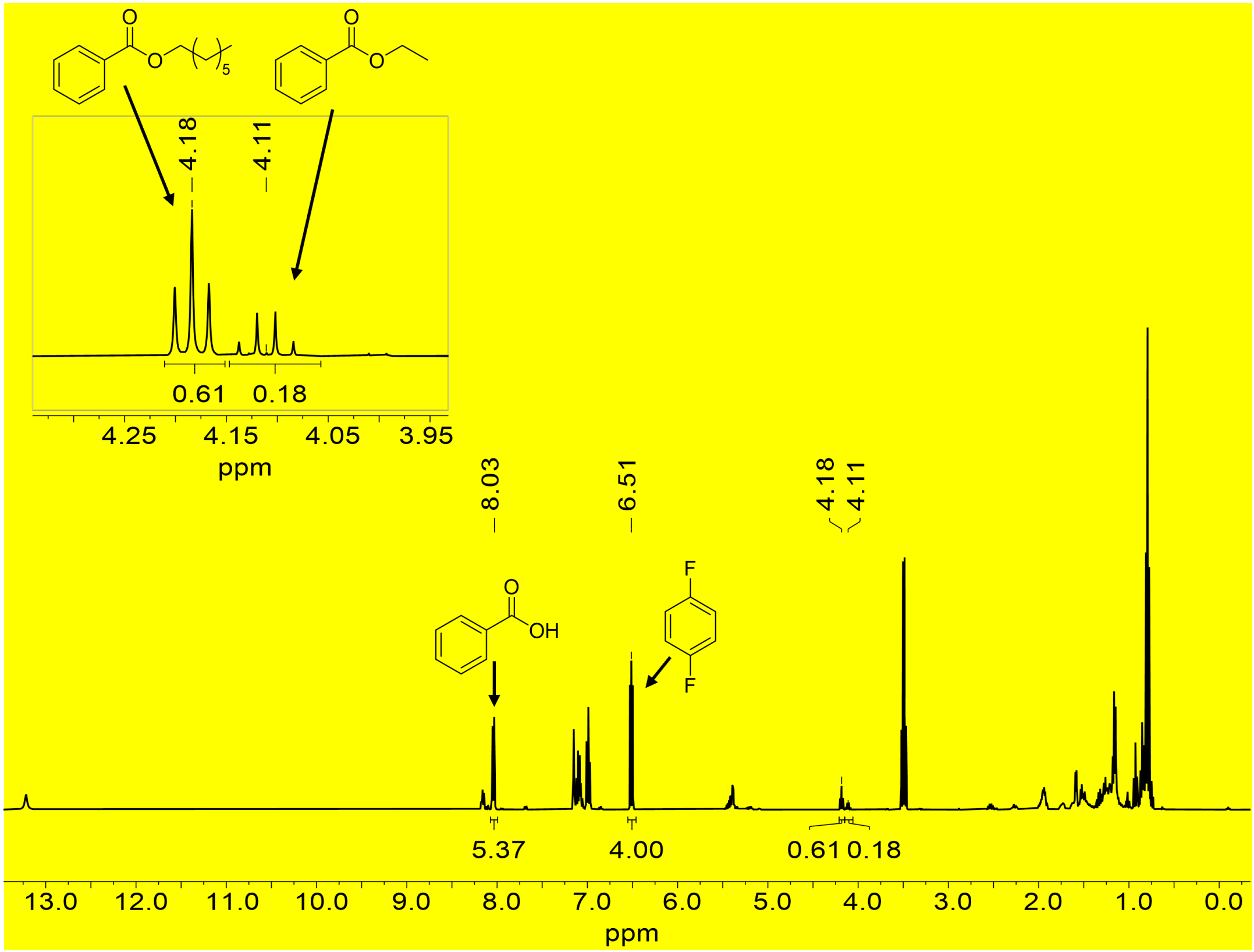

5 Solid state characterisation of polymers

5.1 Infrared spectroscopy

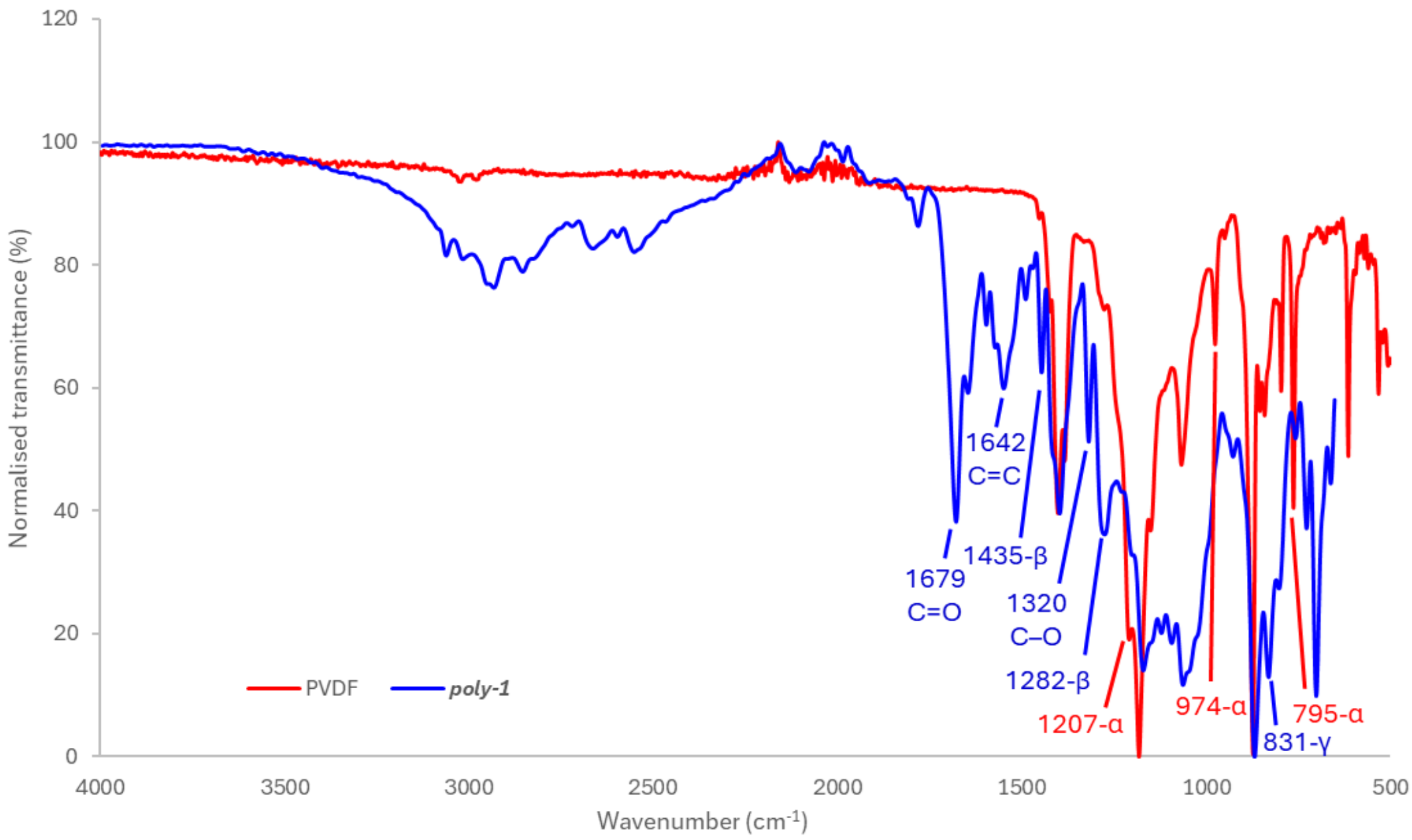

Figure S.5.1 Infrared spectrum of PVDF (red) and ***poly-1*** (blue).

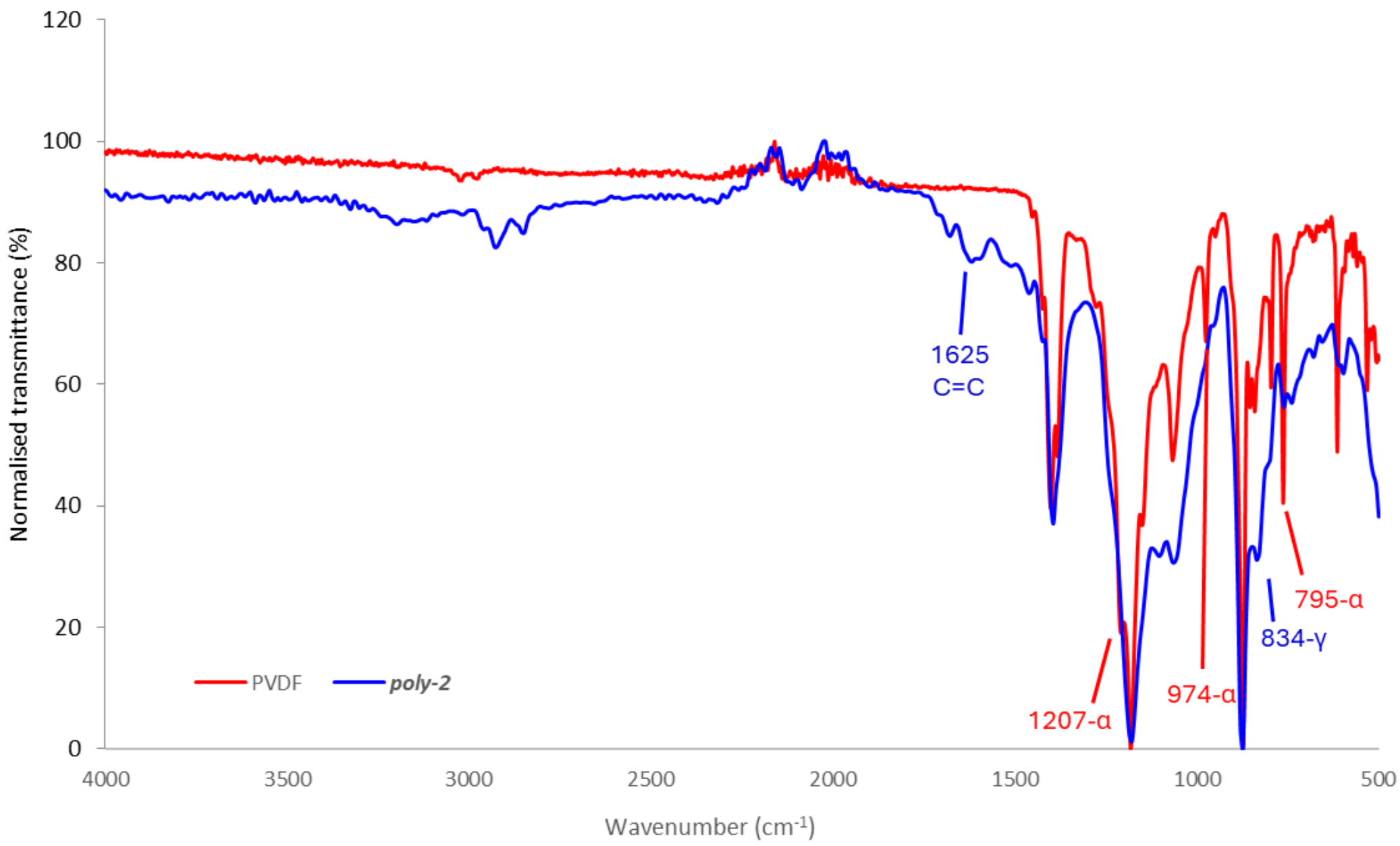

Figure S.5.2 Infrared spectrum of PVDF (red) and ***poly-2*** (blue).

To confirm the identity of the peaks assigned C=C vibration modes, a sample of ***poly-1*** was reacted with excess BH_3·THF for 24 h. The polymer was then isolated by filtration and washed with diethyl

ether, then dried under vacuum, generating ***poly-s9***, before an IR spectrum was obtained (**Figure S.5.3**). The loss of peaks associated with C=C and C=O was observed.

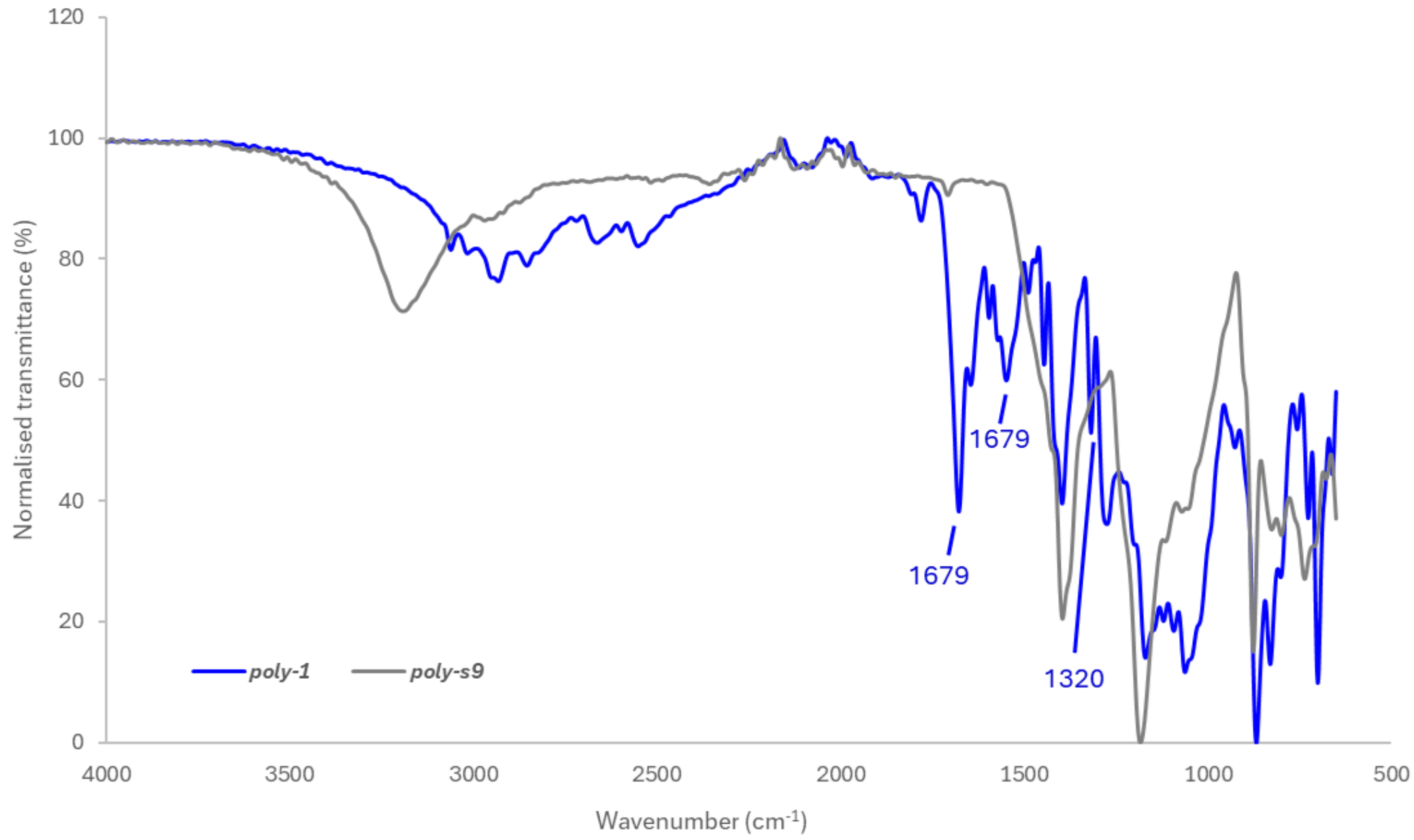

Figure S.5.3 Infrared spectrum of ***poly-1*** (blue) and ***poly-s9*** (grey) after subjection to BH_3·THF.

Similarly, in the case of ***poly-2***, analysis of the hydroborated product, ***poly-s10***, showed the disappearance of peaks associated with C=C vibrational modes (**Figure S.5.4**).

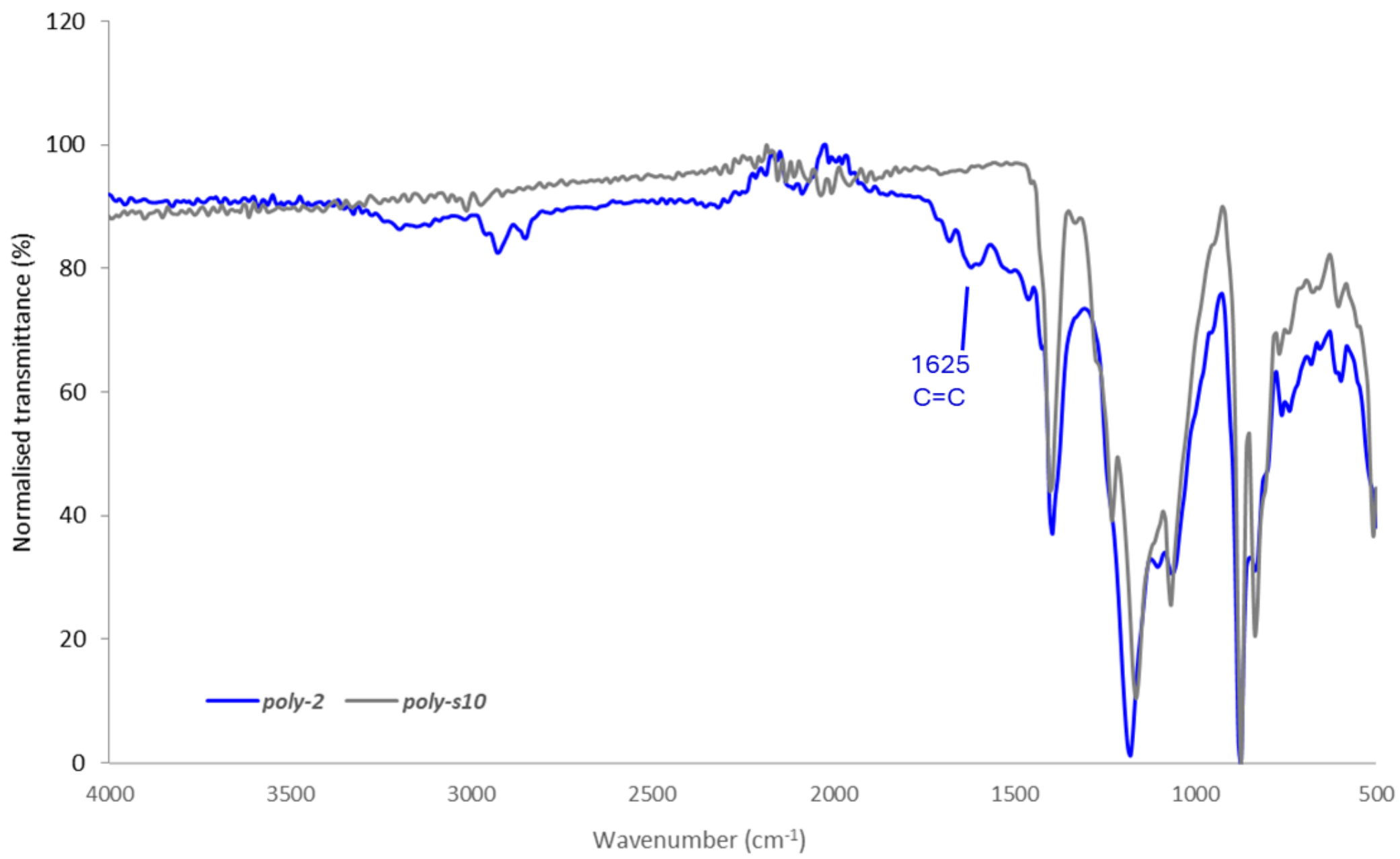

Figure S.5.4 Infrared spectrum of ***poly-2*** (blue) and ***poly-s10*** (grey) after subjection to BH_3·THF.

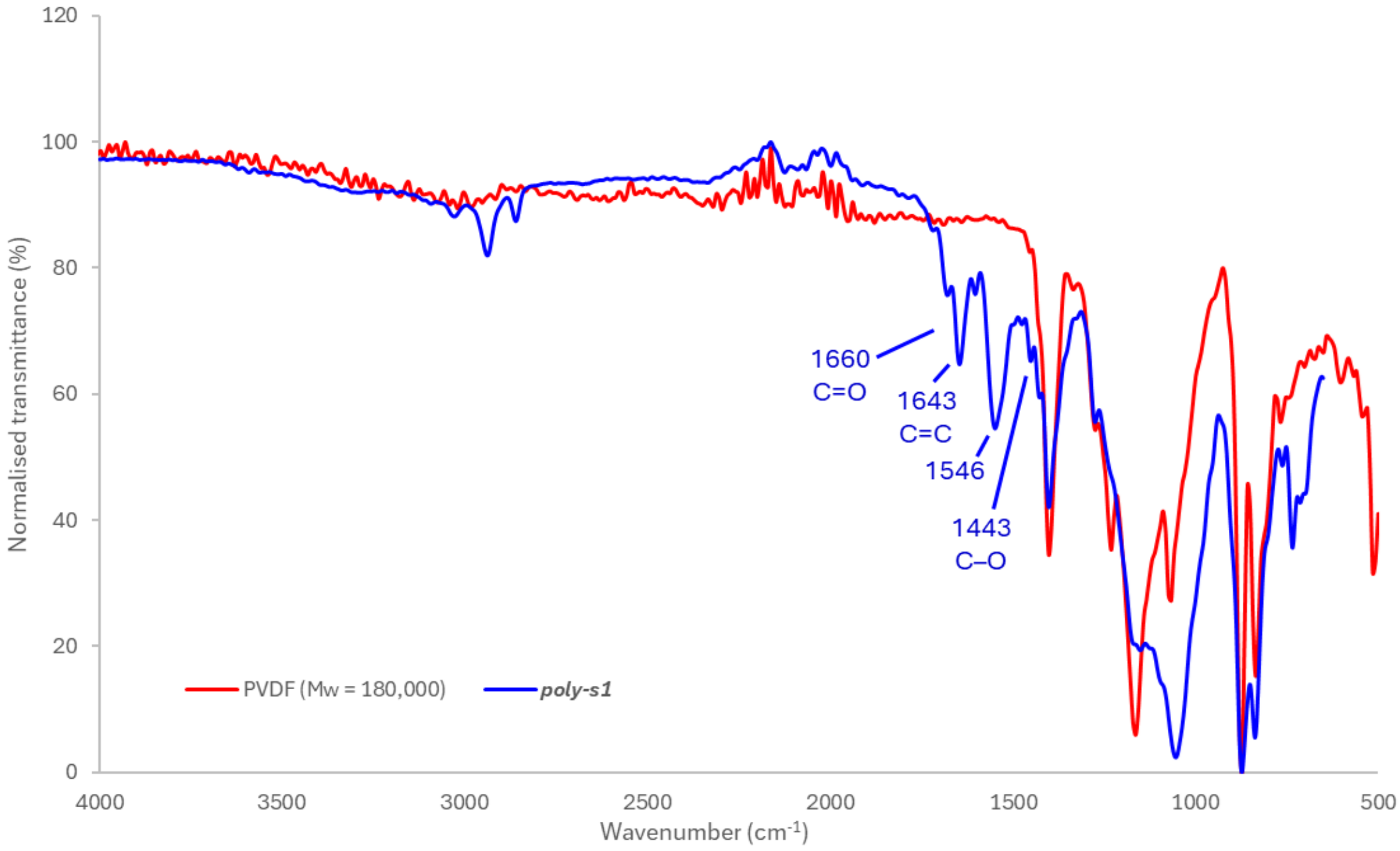

Figure S.5.5 Infrared spectrum of PVDF (red) and ***poly-s1*** (blue).

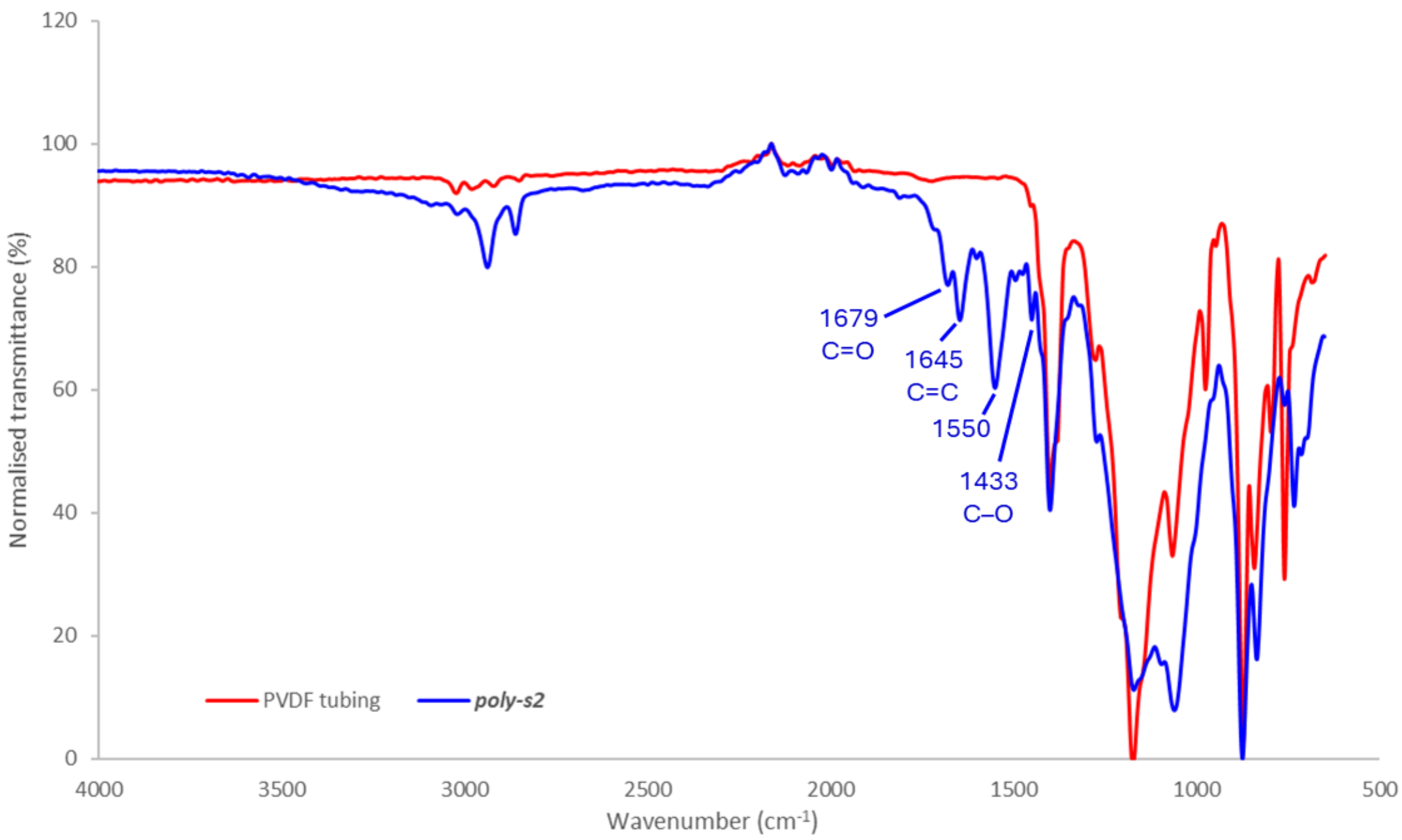

Figure S.5.6 Infrared spectrum of PVDF tubing (red) and ***poly-s2*** (blue).

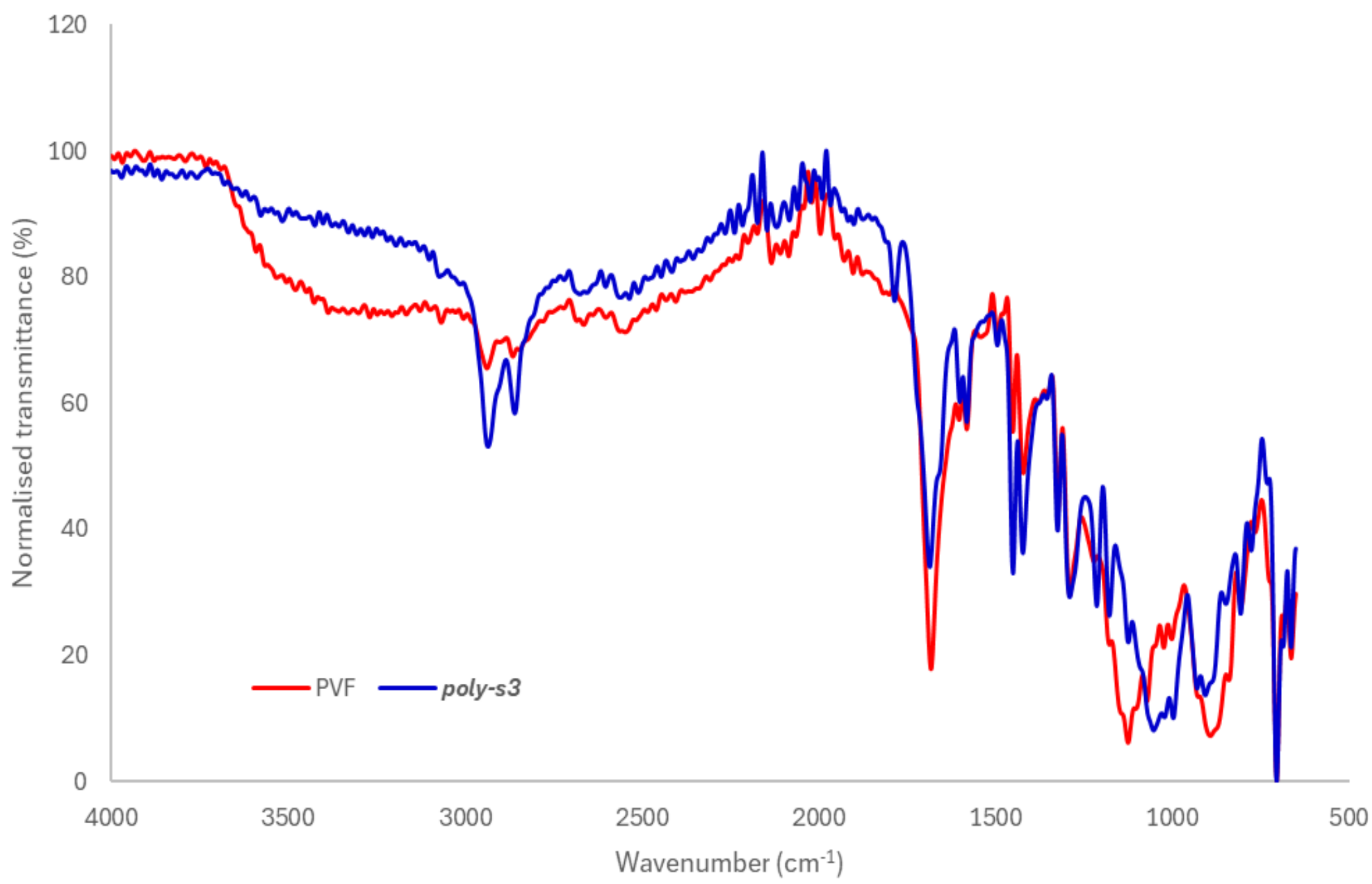

Figure S.5.7 Infrared spectrum of PVF stickers (red) and ***poly-s3*** (blue).

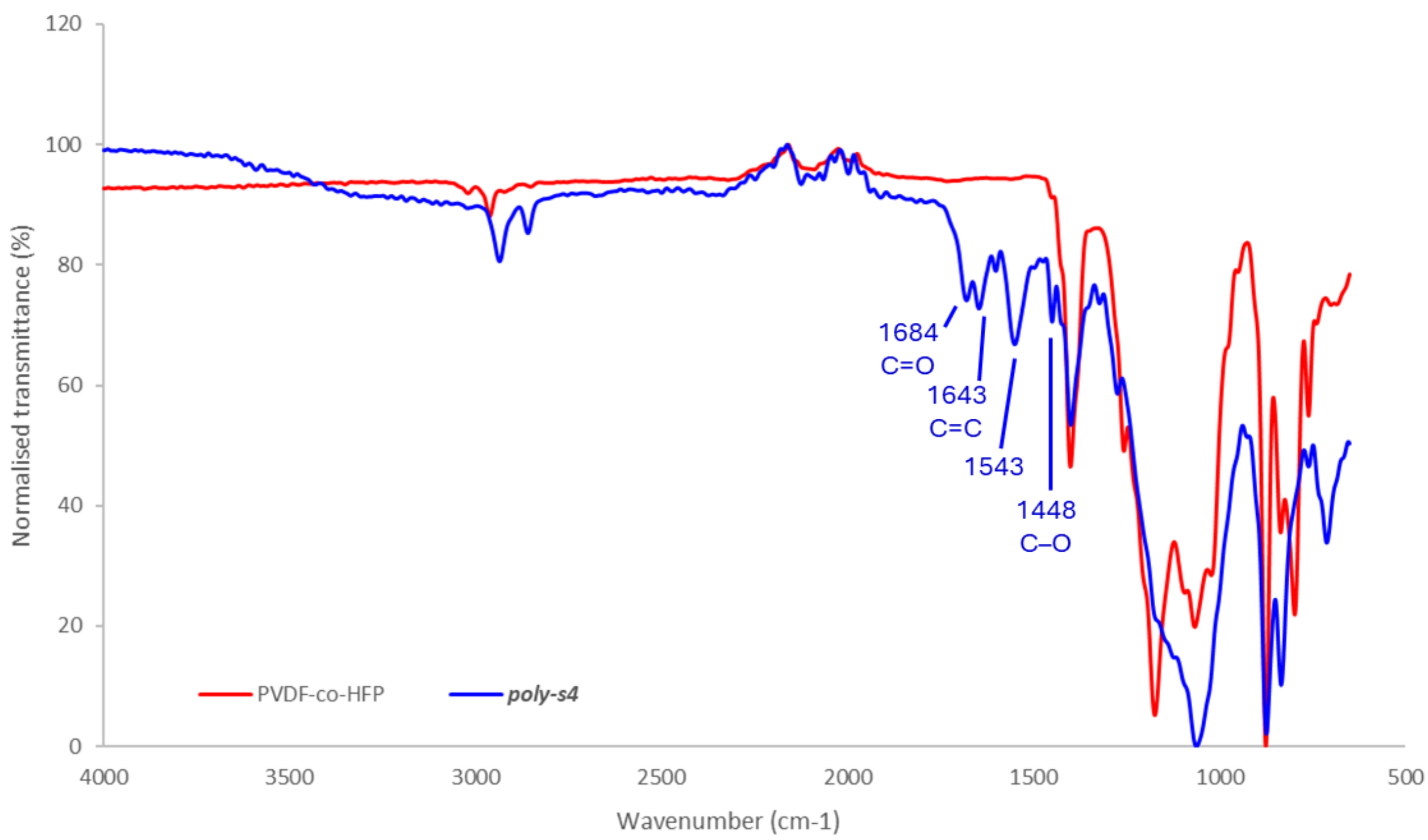

Figure S.5.8 Infrared spectrum of PVDF-co-HFP (red) and ***poly-s4*** (blue).

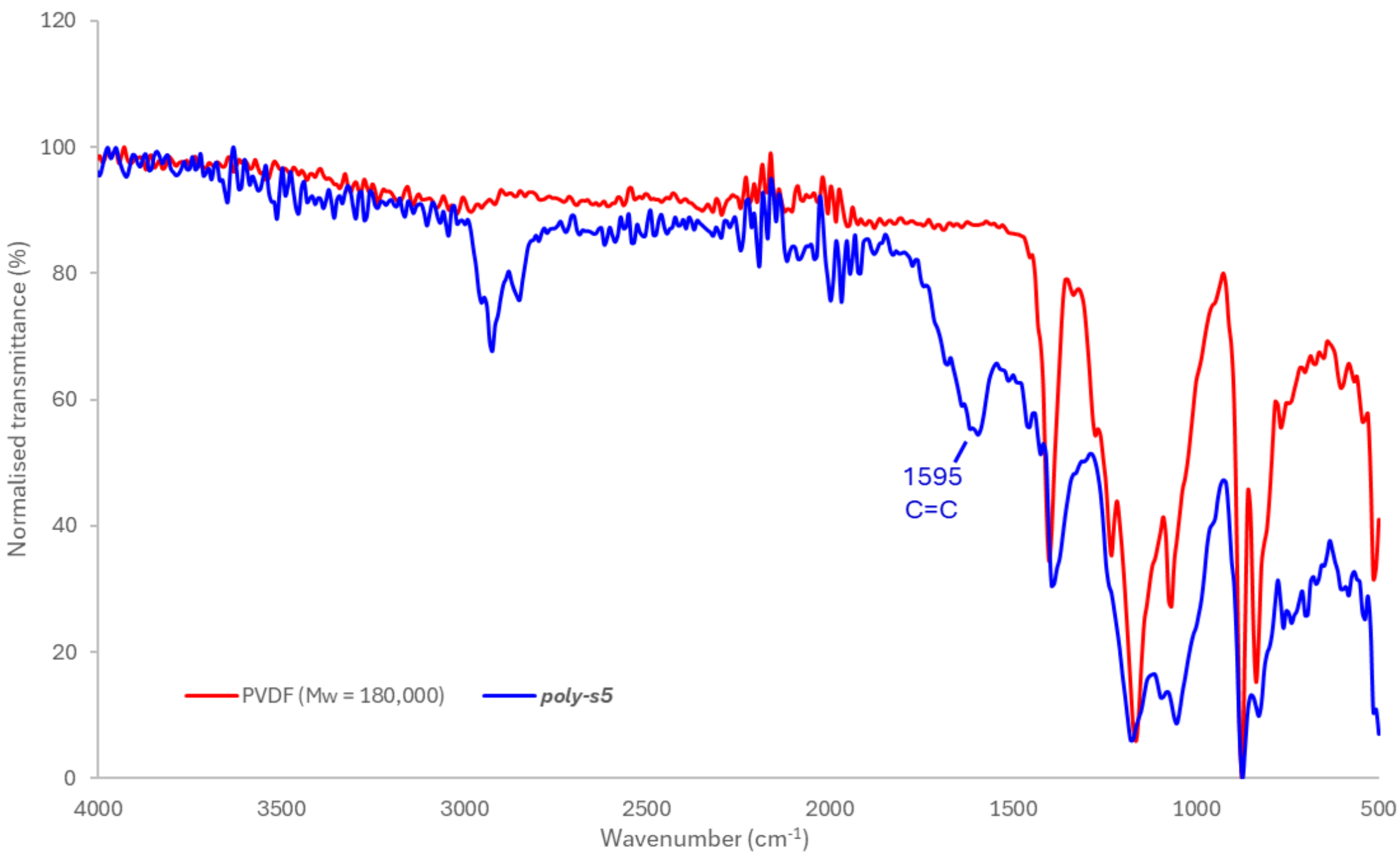

Figure S.5.9 Infrared spectrum of PVDF (M_W = 180,000) and ***poly-s5*** (blue).

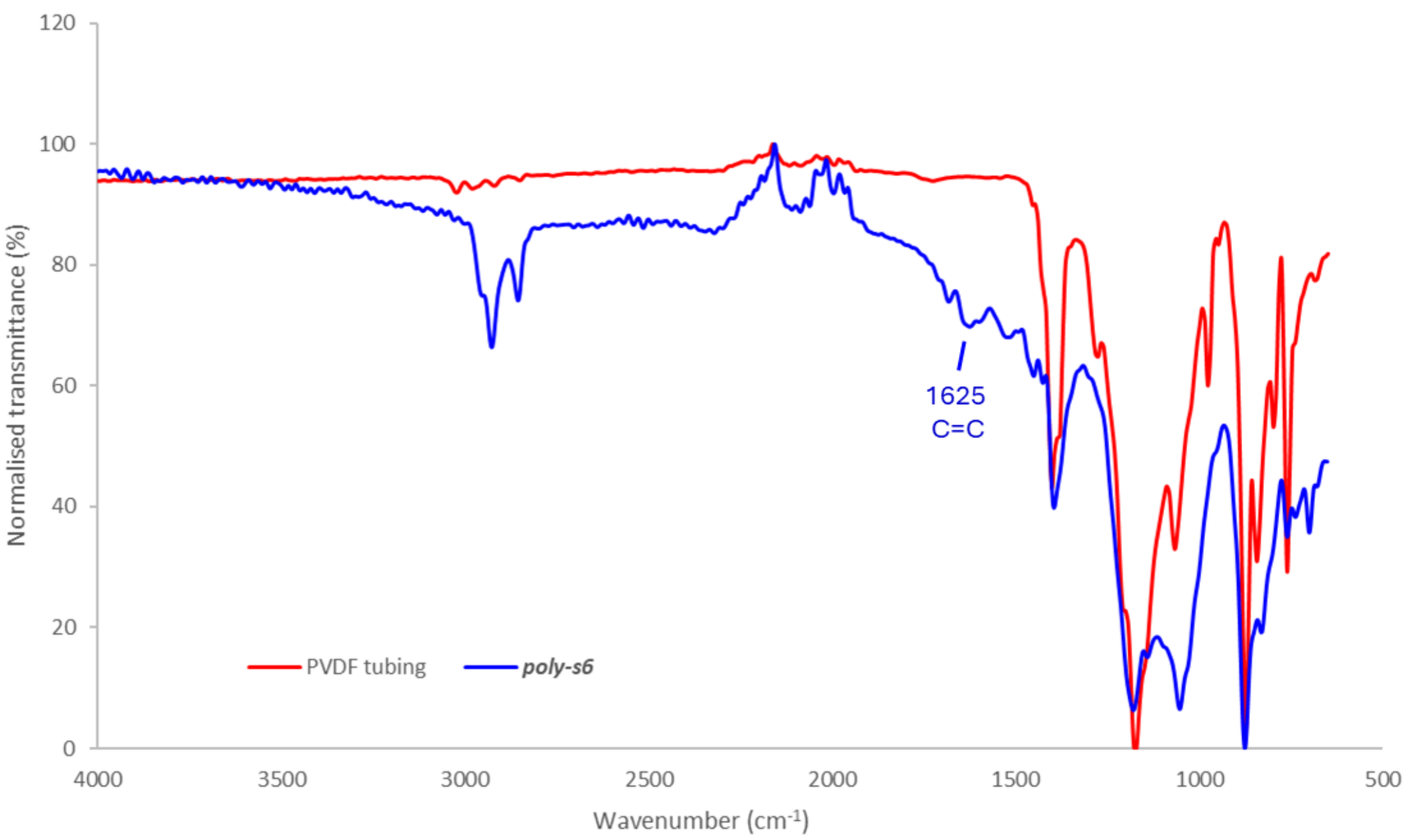

Figure S.5.10 Infrared spectrum of PVDF tubing (red) and ***poly-s6*** (blue).

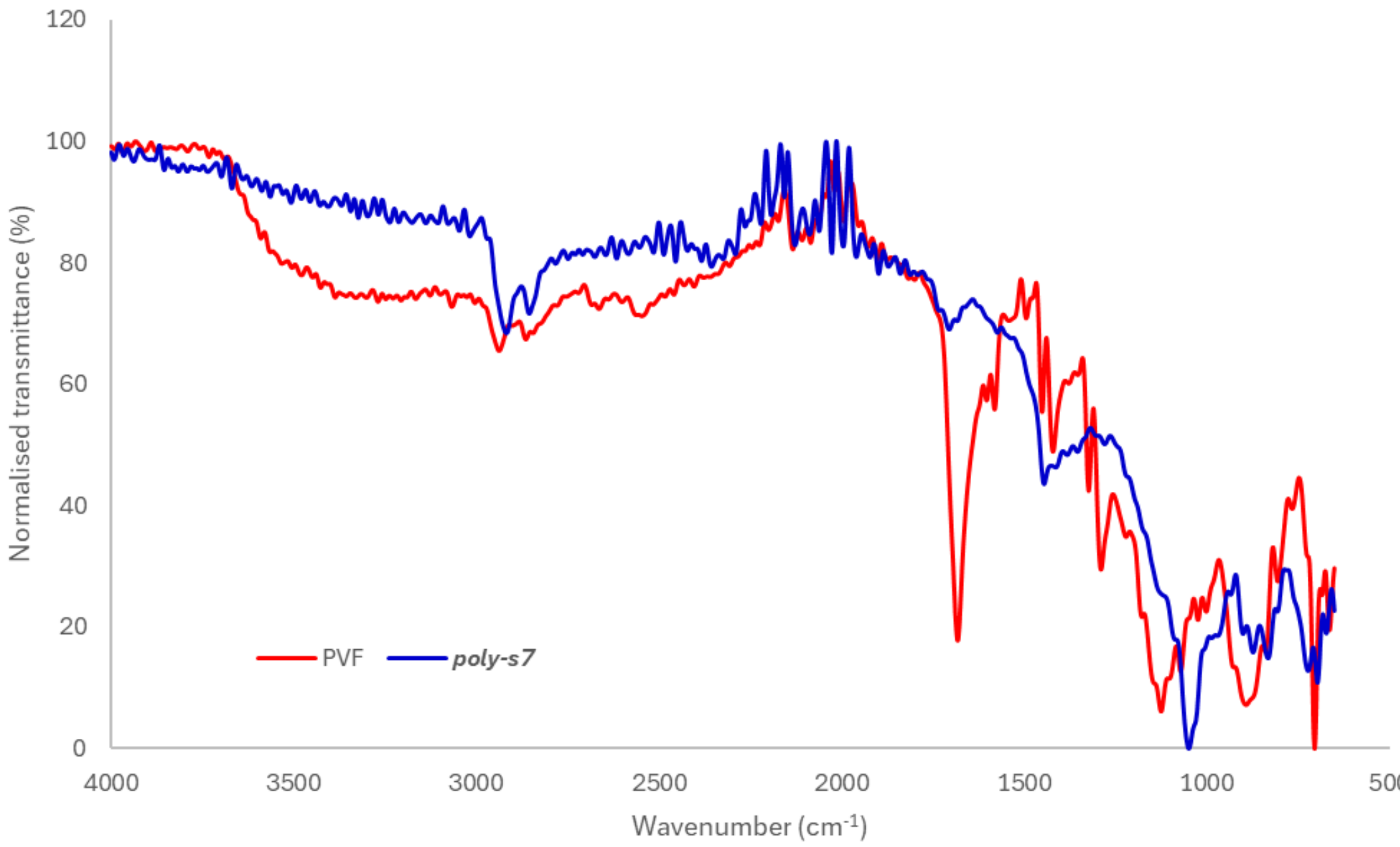

Figure S.5.11 Infrared spectrum of PVF (red) and ***poly-s7*** (blue).

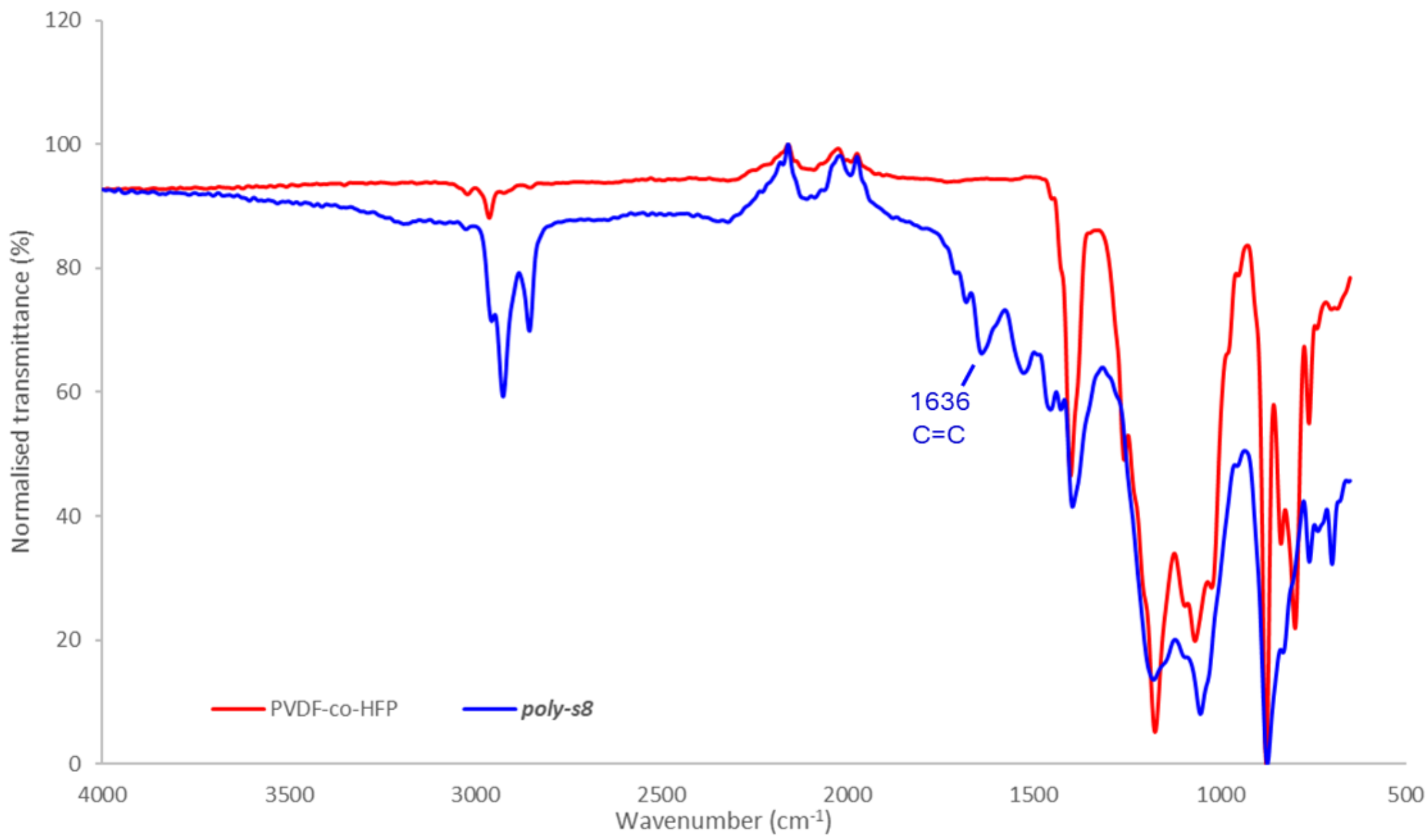

Figure S.5.12 Infrared spectrum of PVDF-co-HFP (red) and ***poly-s8*** (blue).

5.2 X-ray photoelectron spectroscopy

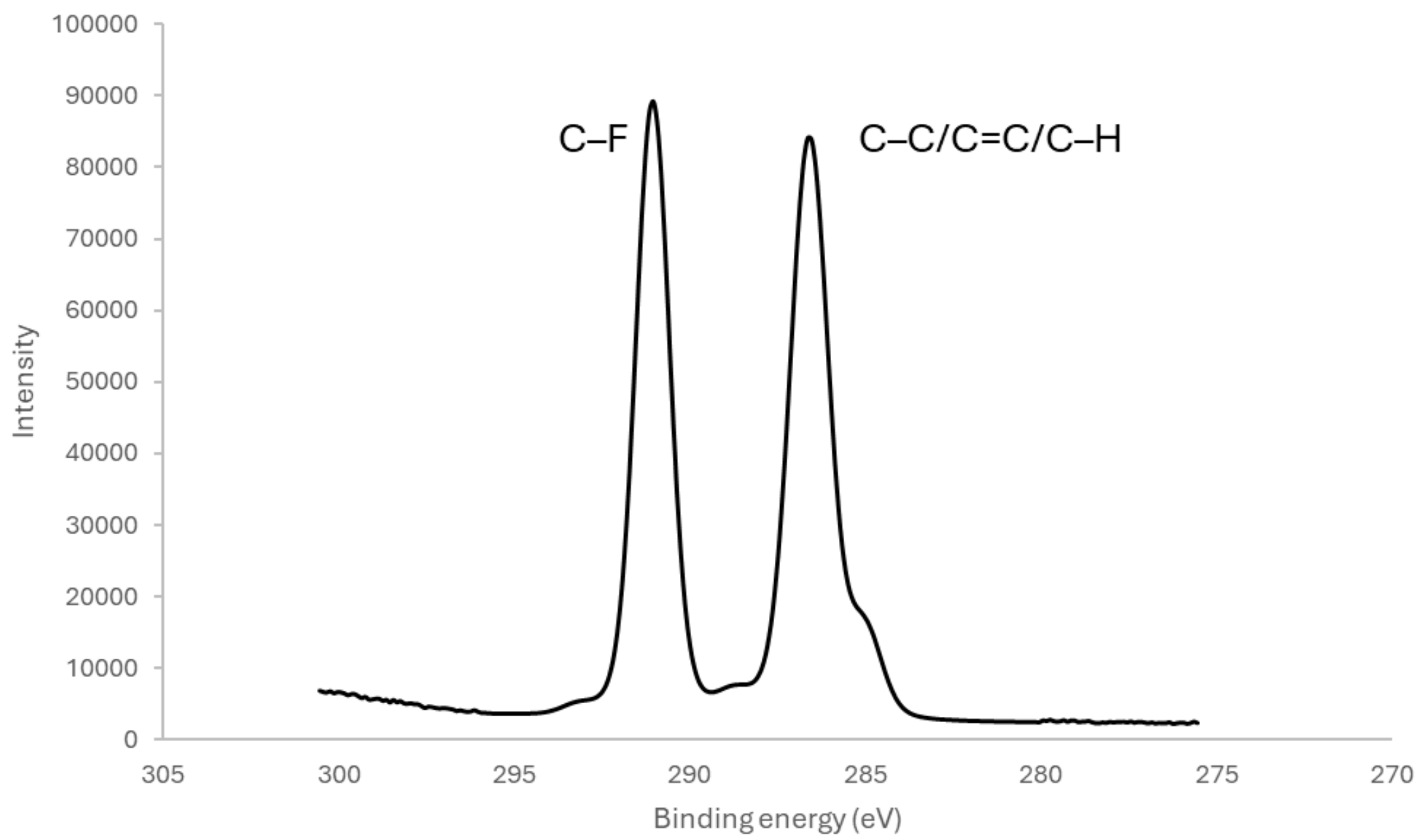

Figure S.5.13 1Cs X-ray photoelectron spectrum of PVDF.

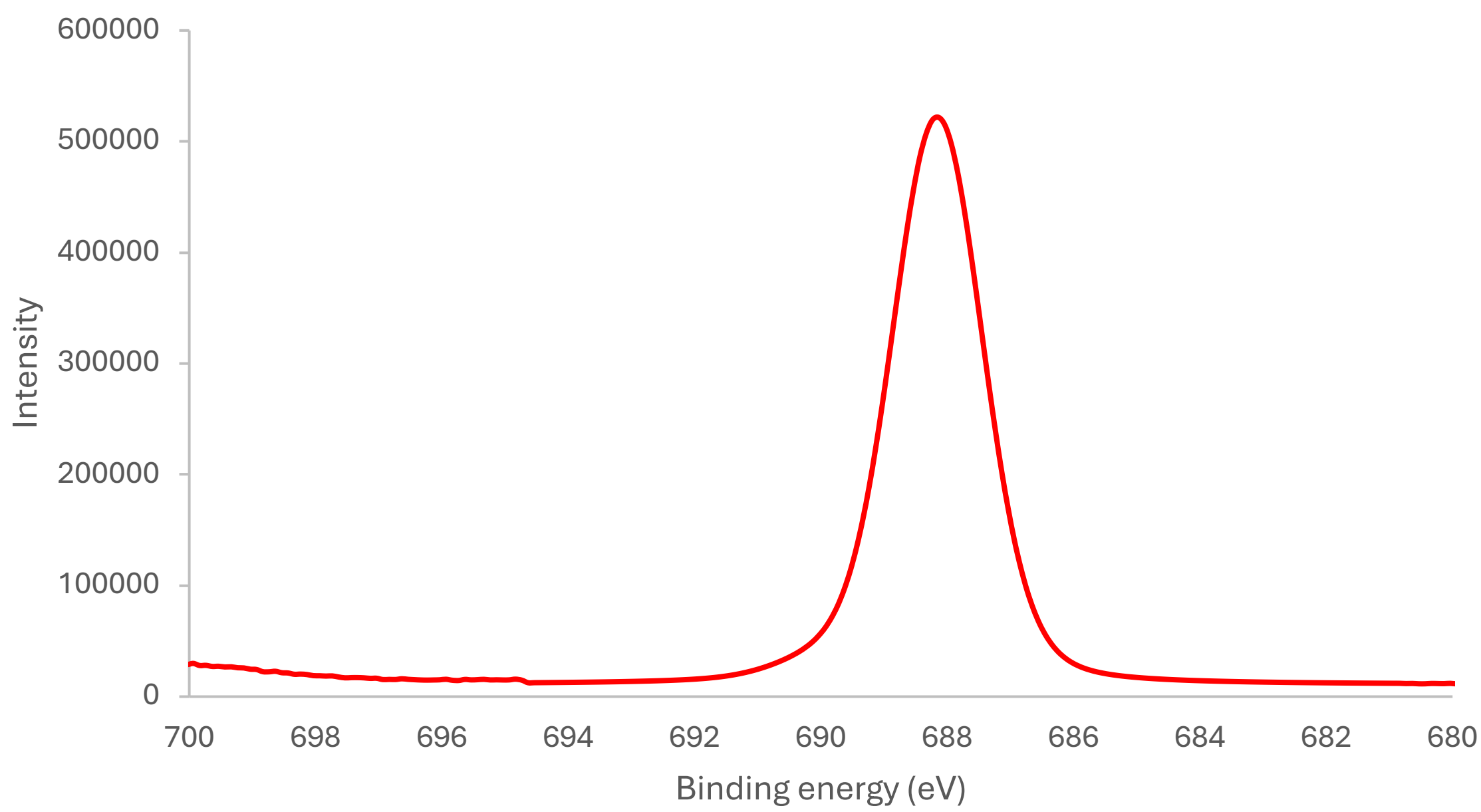

Figure S.5.14 1Fs X-ray photoelectron spectrum of PVDF.

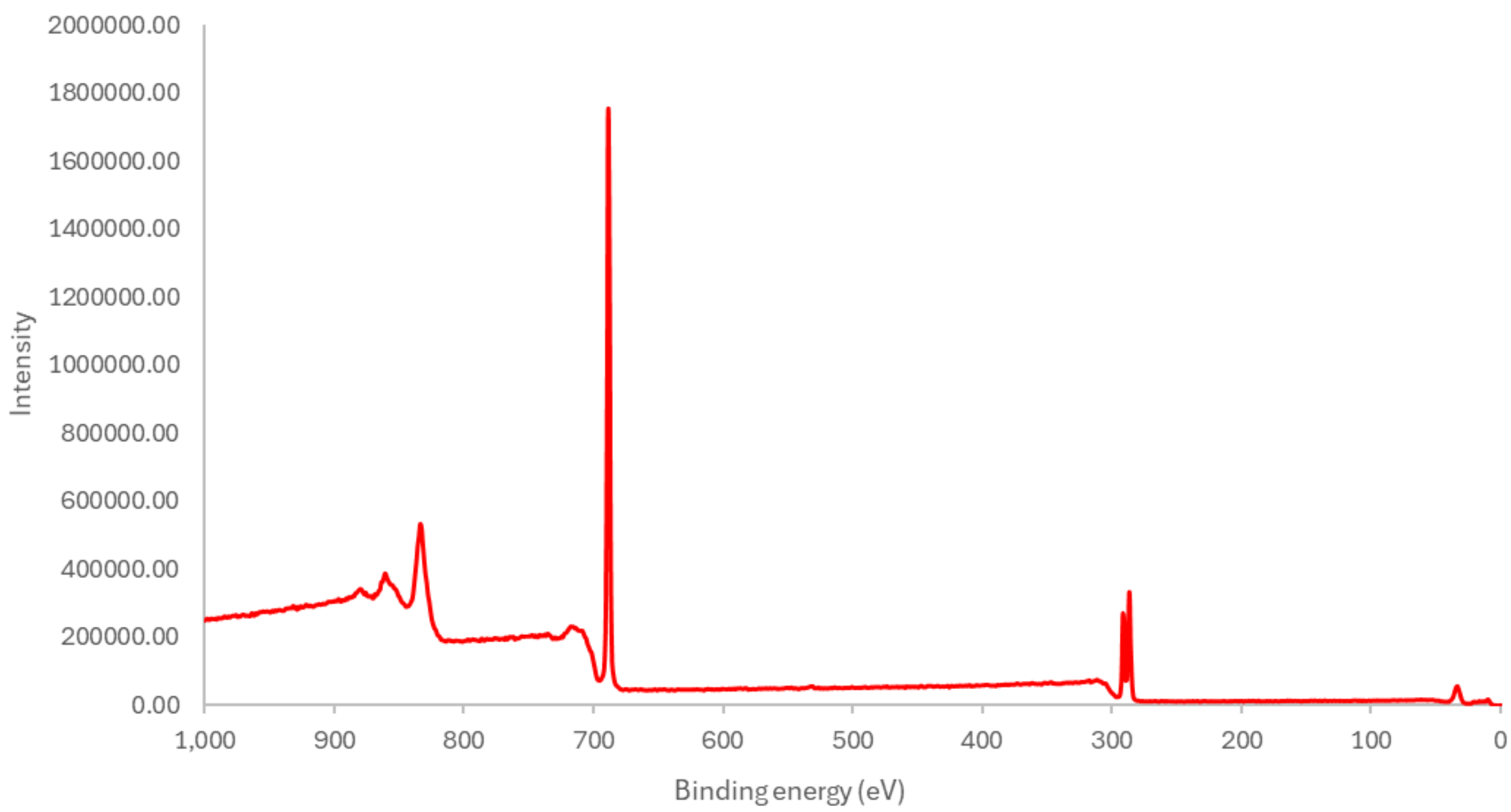

Figure S.5.15 X-Ray photoelectron survey of PVDF.

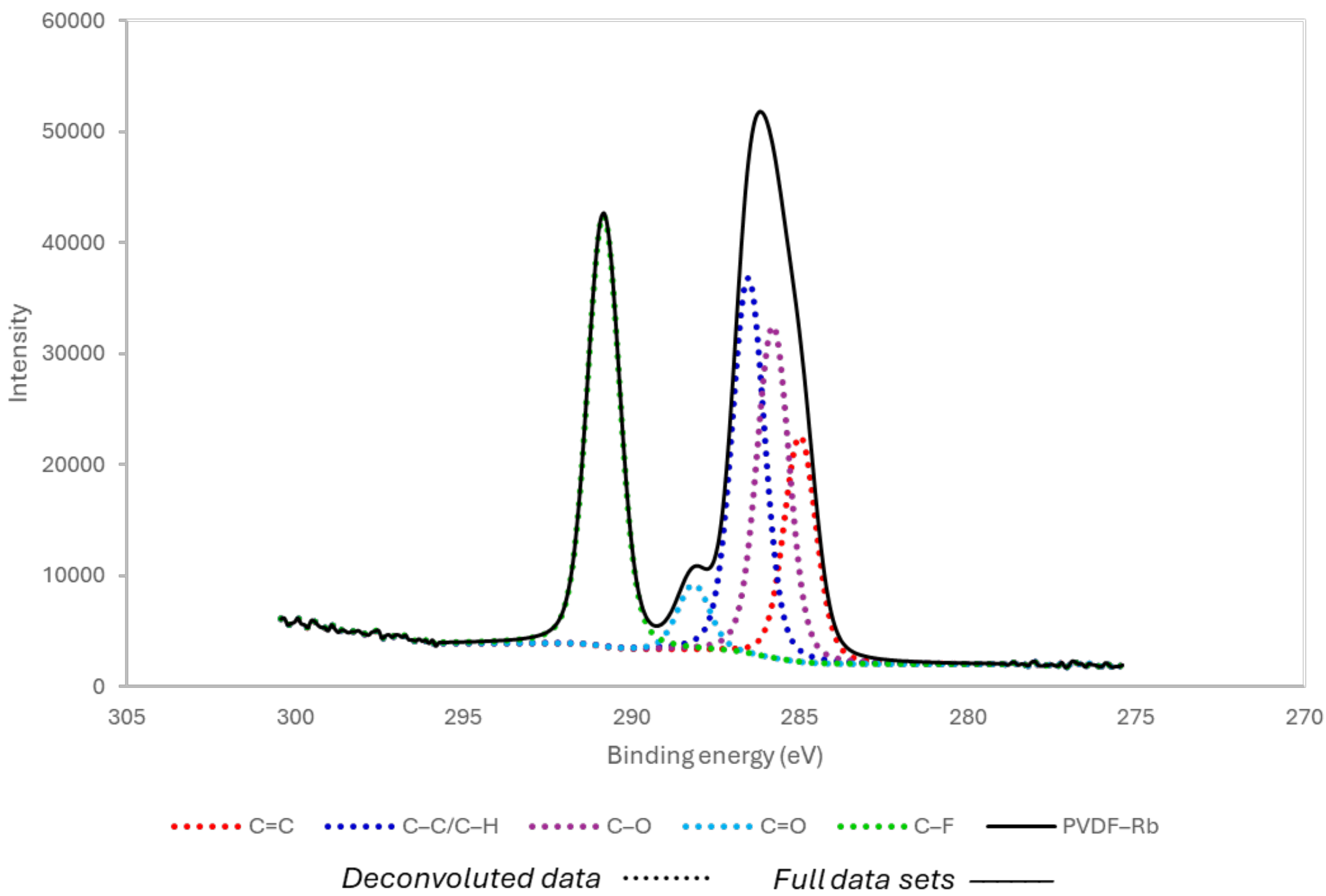

Figure S.5.16 1Cs X-ray photoelectron spectrum of ***poly-1***.

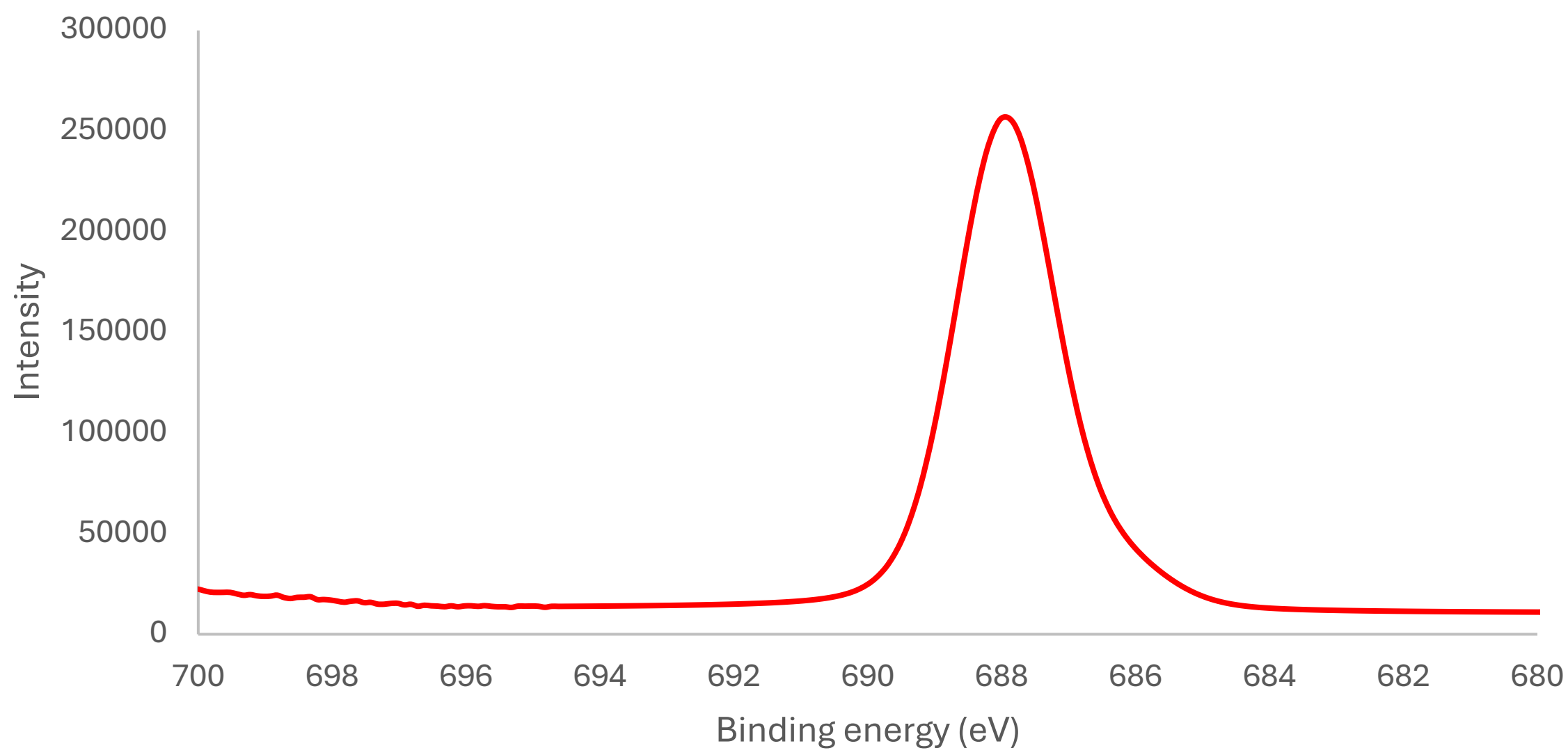

Figure S.5.17 1Fs X-ray photoelectron spectrum of ***poly-1***.

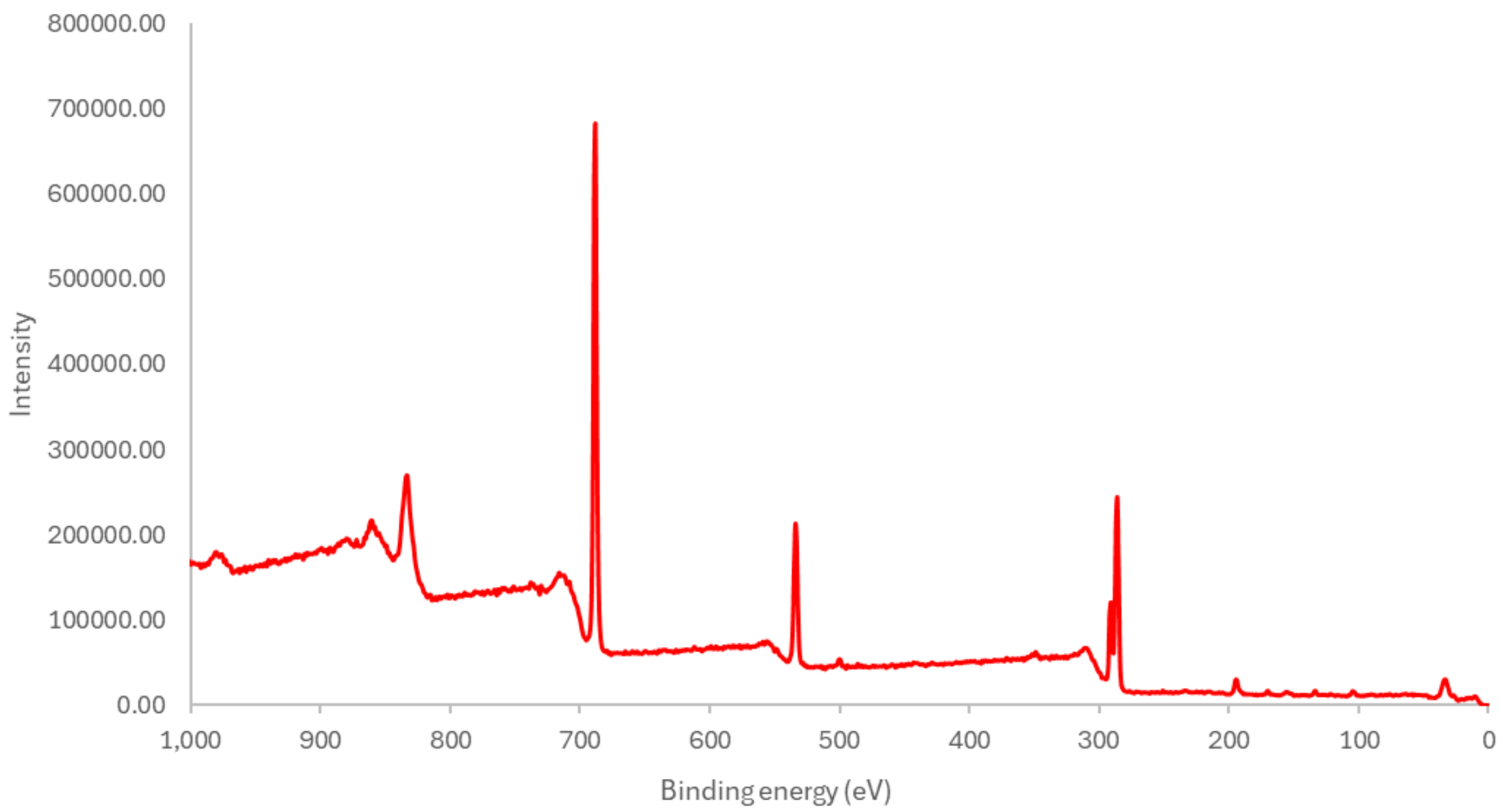

Figure S.5.21 X-ray photoelectron spectrum survey of ***poly-1***.

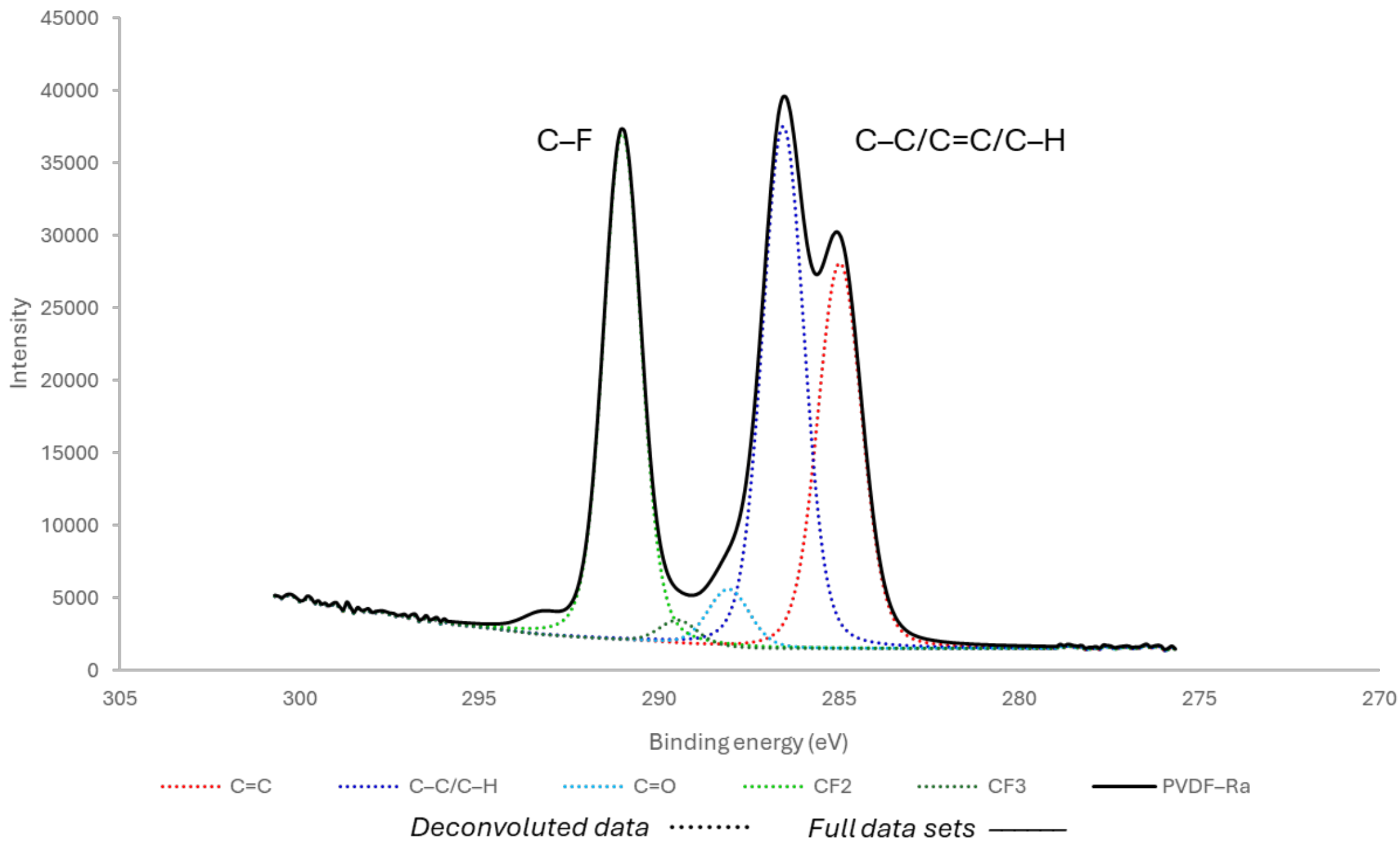

Figure S.5.18 1Cs X-ray photoelectron spectrum of ***poly-2***.

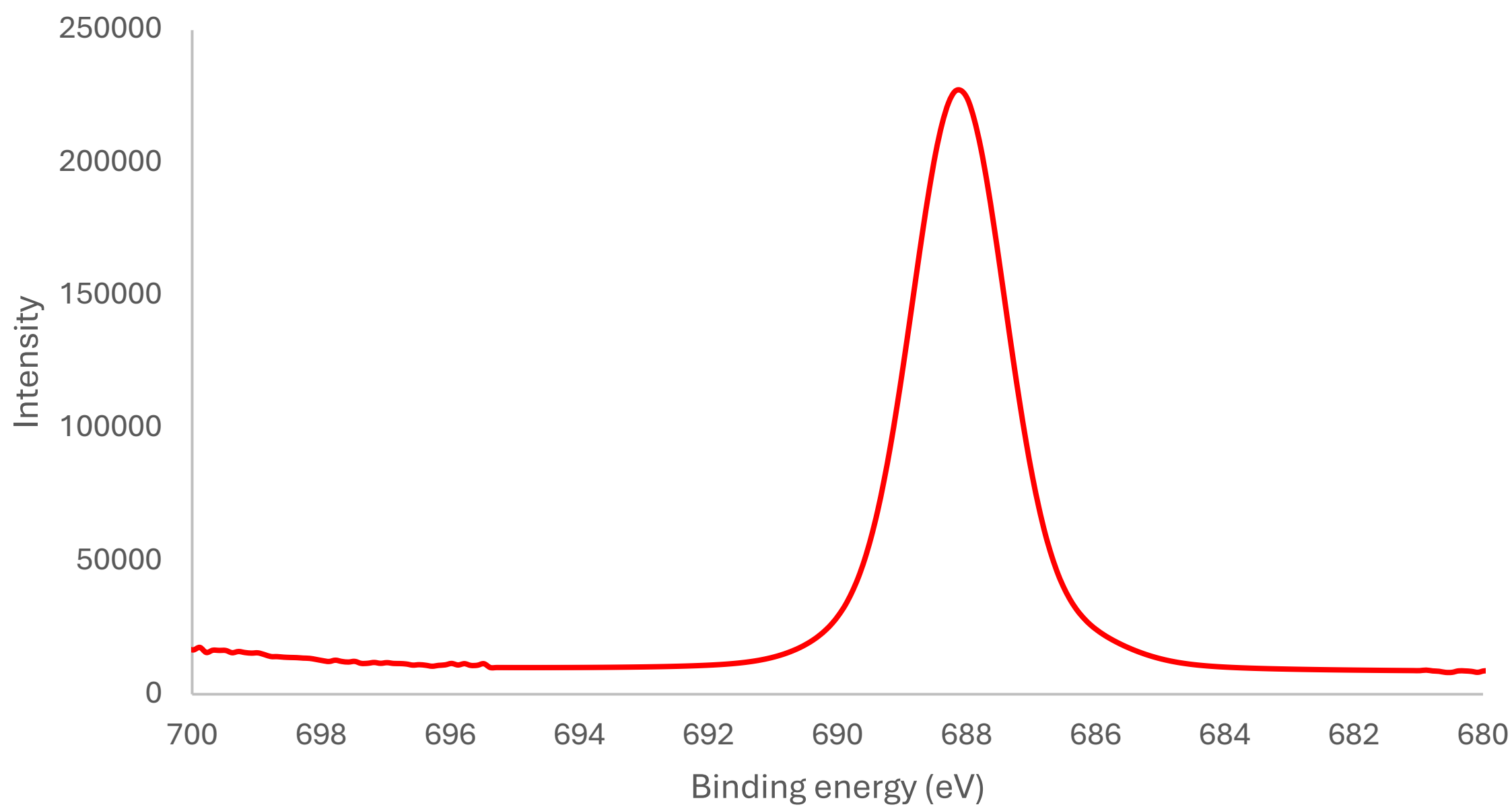

Figure S.5.19 1Fs X-ray photoelectron spectrum of ***poly-2*.**

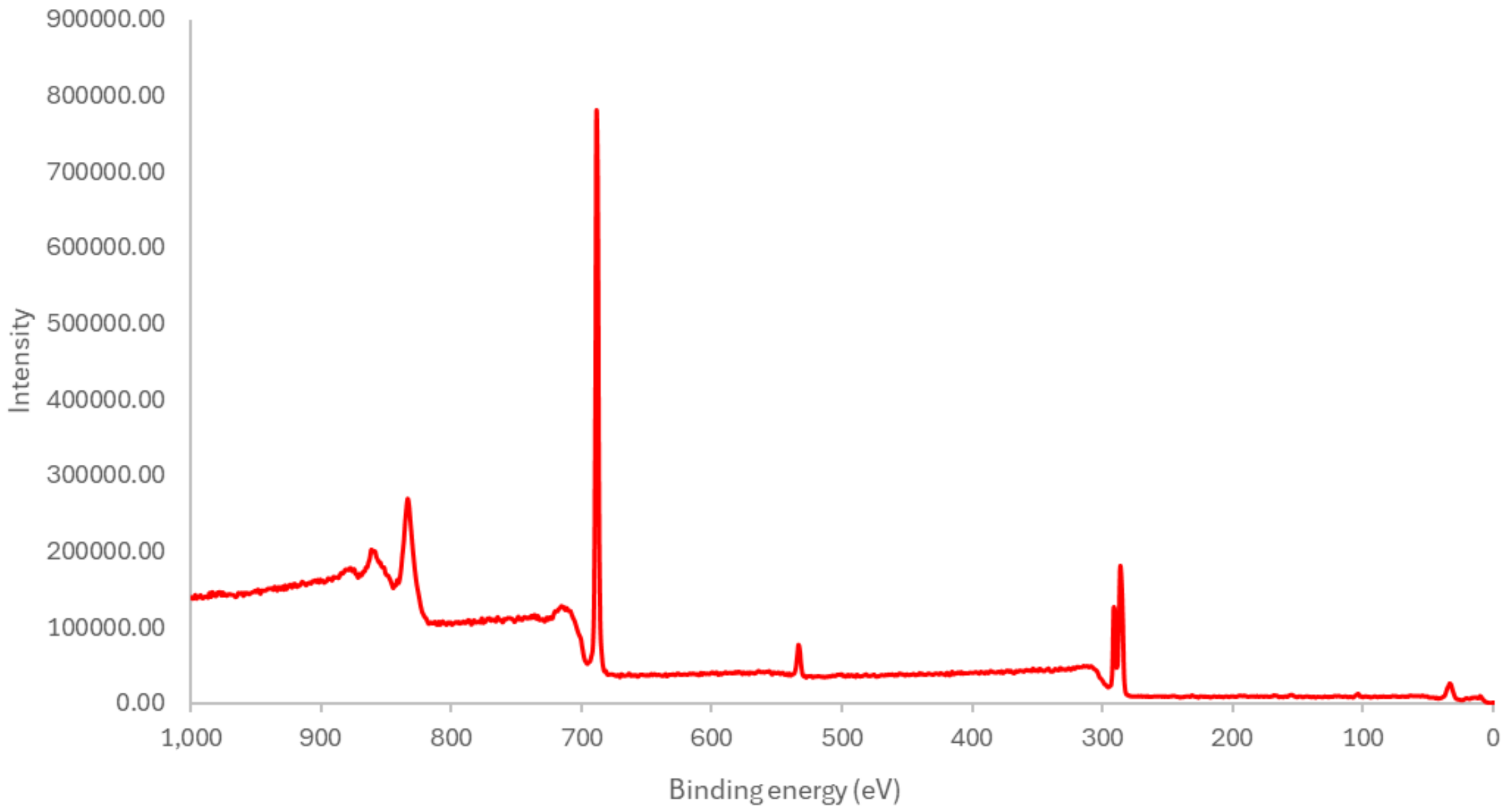

Figure S.5.20 X-ray photoelectron spectrum survey of ***poly-2***.

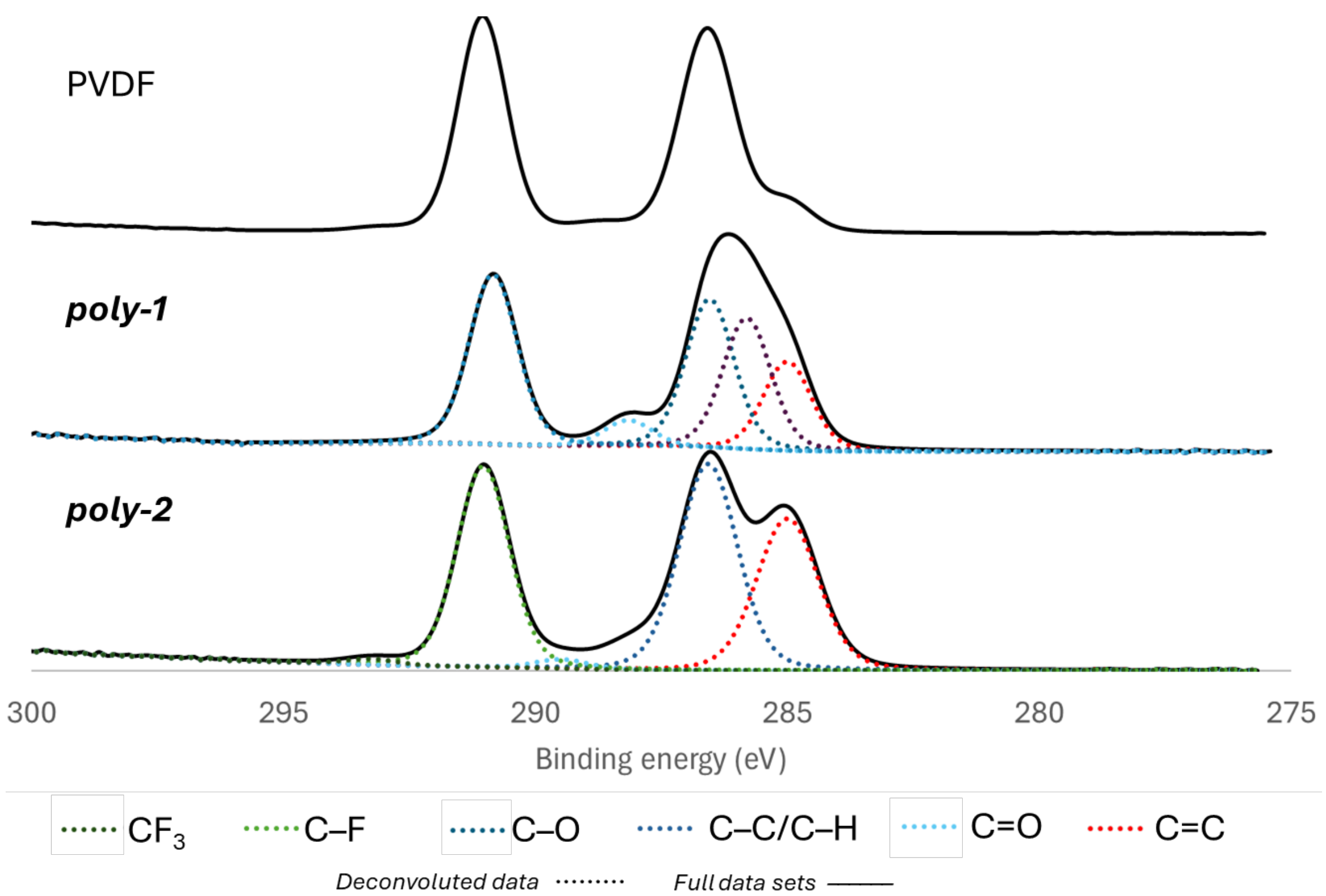

Figure S.5.21 Stacked C1s X-ray photoelectron spectrums of PVDF, ***poly-1*** and ***poly-2***.

Assignment	C 1s (C=C)	C 1s (C–C)	C 1s (C–O)	C 1s (CF_2)
Position	285	286.59	288.72	291.05
FWHM	1.15	1.29	1.29	1.14
Area	15099.64	121289.5	4599.88	113792.4
Area as percentage (%)	5.89	47.27	1.79	44.36

Table S.5.1 Integrated C1s scan of PVDF.

Assignment	C 1s (C=C)	C 1s (C–CF or C–O)	C 1s (C–H)	C 1s (O–C=O)	C 1s (C–F)
Position	285	285.8	286.54	288.16	290.84
FWHM	1.13	1.13	1.13	1.13	1.13
Area	26800.85	39343.56	44508.34	7792.94	51106.5
Area as percentage (%)	15.8	23.2	26.3	4.60	30.2

Table S.5.2 Integrated C1s scan of ***poly-1***.

Assignment	C 1s (C=C)	C 1s (C–C)	C 1s (C–O)	C 1s (CF_2)	C 1s (COO)
Position	285	286.57	288.08	291.04	289.48
FWHM	1.44	1.37	1.37	1.23	1.23
Area	44227.5	56710.54	6292.72	49658.98	2204.5
Area as percentage (%)	27.55	35.31	3.91	30.93	1.37

Table S.5.3 Integrated C1s scan of ***poly-2*.**

5.3 Powder X-ray diffraction spectroscopy

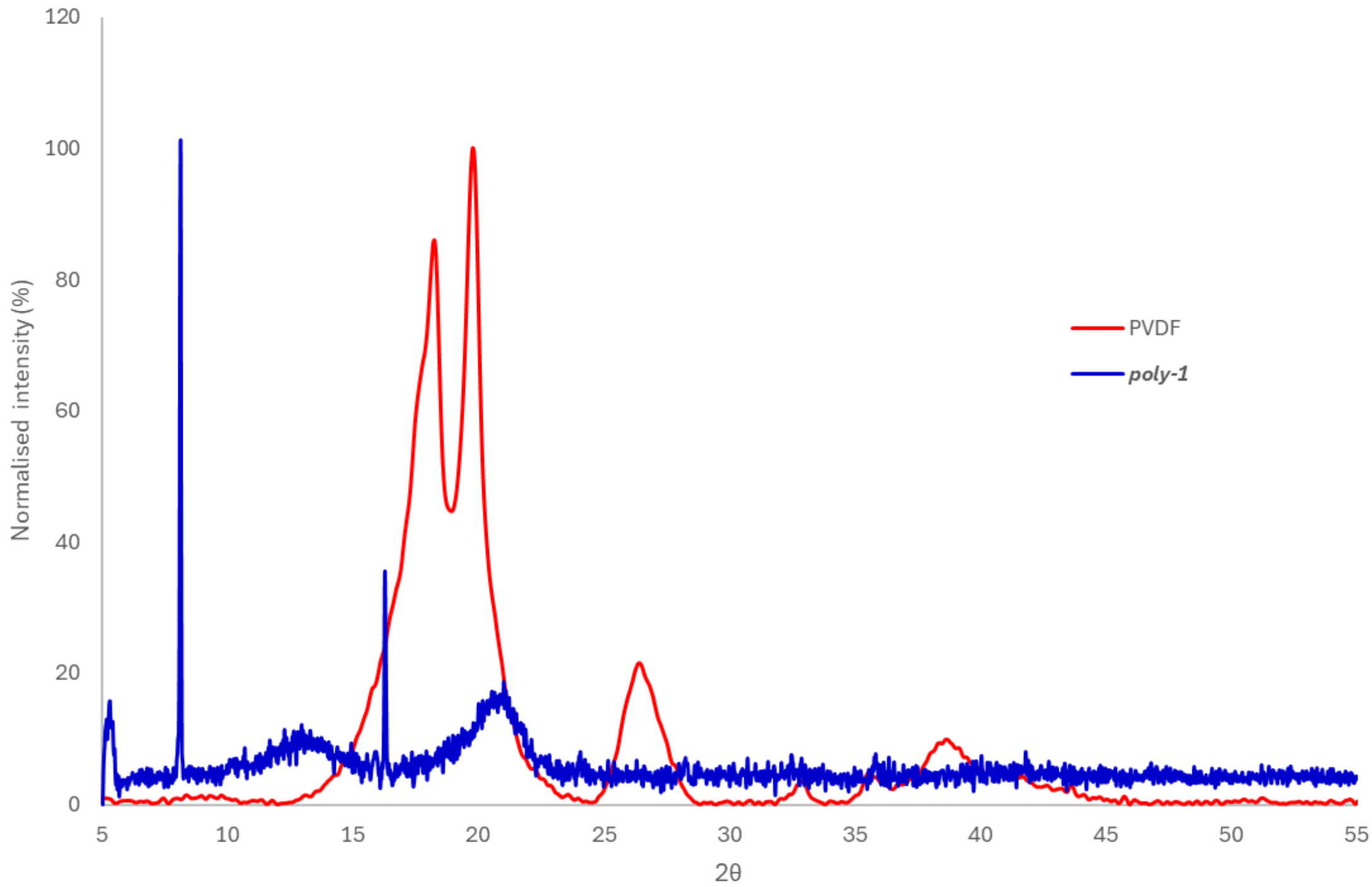

Figure S.5.22 Normalised powder XRD spectrum of PVDF (red) and ***poly-1*** (blue).

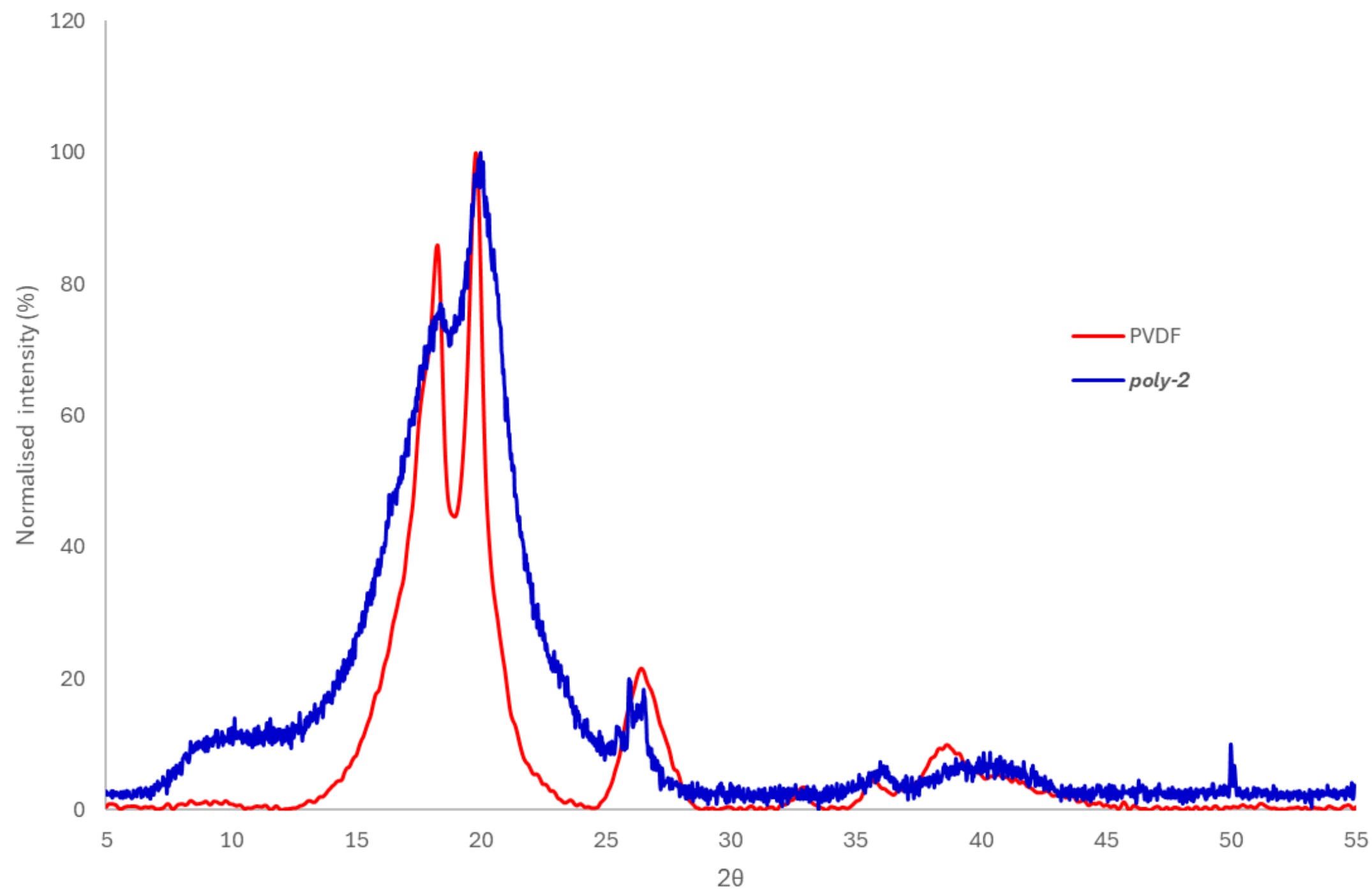

Figure S.5.23 Normalised powder XRD spectrum of PVDF (red) and ***poly-2*** (blue).

5.4 Differential scanning calorimetry

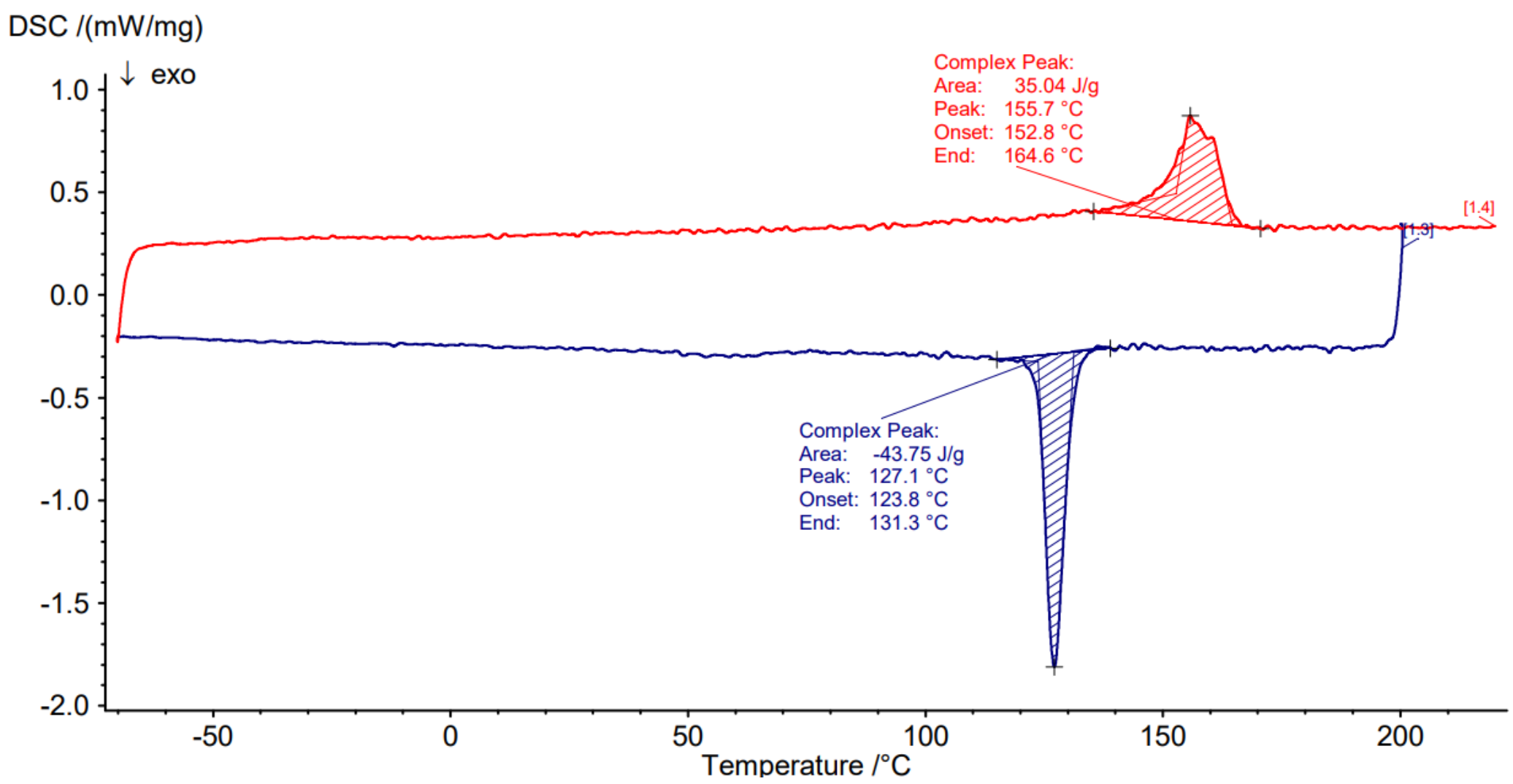

Figure S.5.24 Differential scanning calorimetry (DSC) of PVDF.

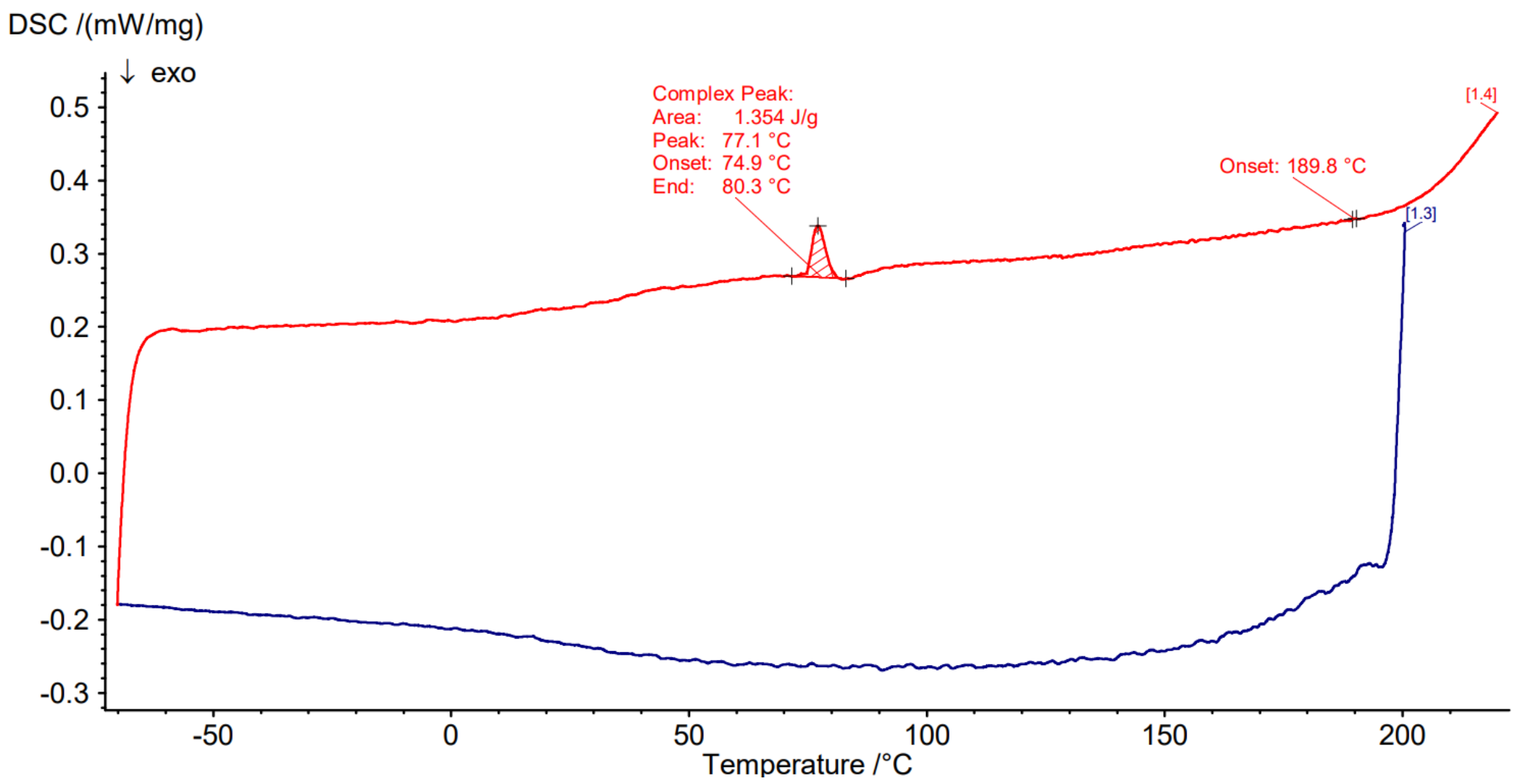

Figure S.5.25 Differential scanning calorimetry (DSC) of ***poly-1***.

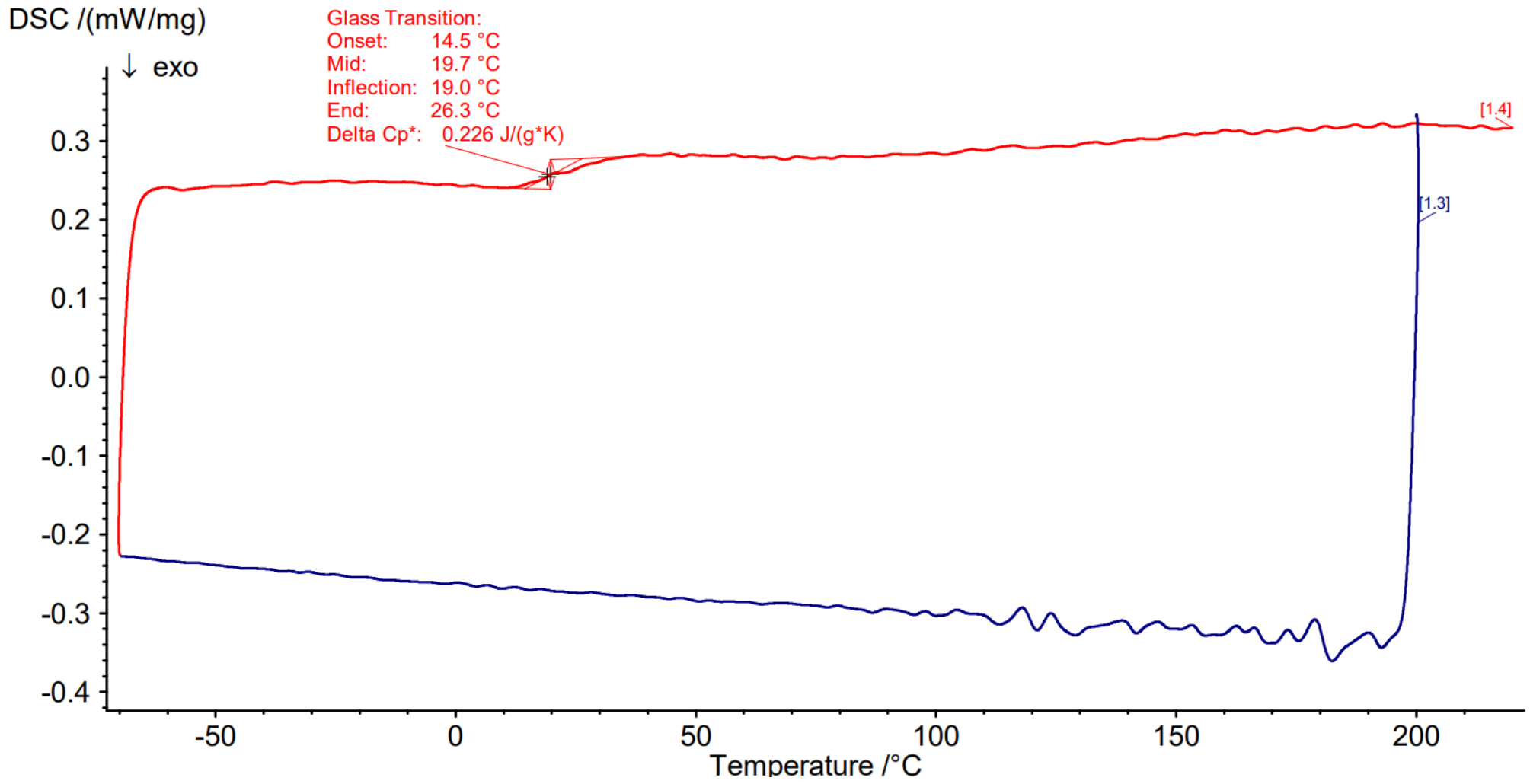

Figure S.5.26 Differential scanning calorimetry (DSC) of ***poly-2***.

5.5 Thermogravimetric-mass spectrum analysis

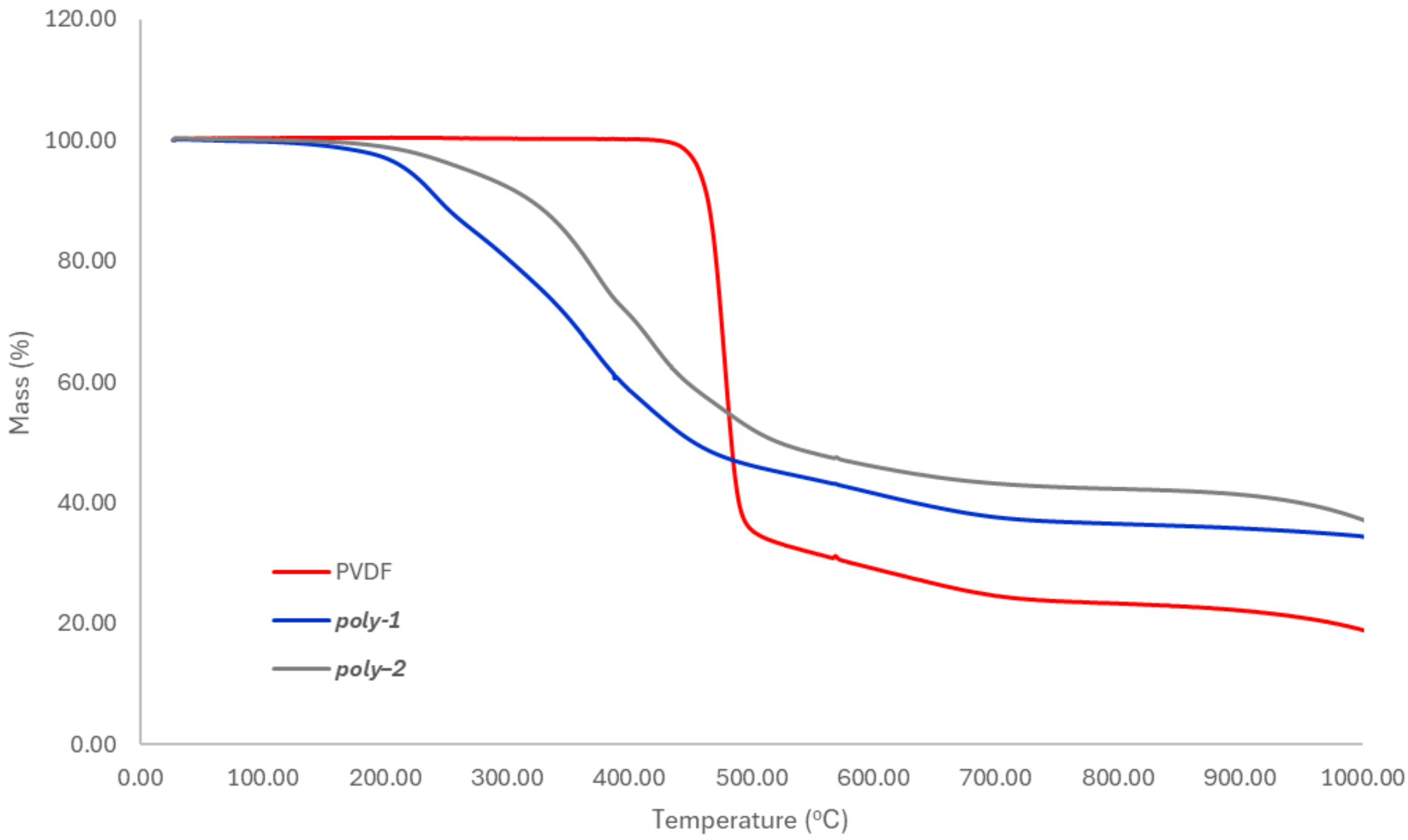

Figure S.5.27 TGA of PVDF (red) ***poly-1*** (blue) and ***poly-2*** (grey).

Entry nº.	m/z	Expected assignment
1	15	CH_3^+
2	16	CH_4^+
3	19	H_3O^+
4	20	HF^+
5	29	$C_2H_5^+$ or CHO^+
6	30	$C_2H_6^+$ or CH_2O^+
7	33	$CH_3OH_2^+$
8	41	$C_3H_5^+$

Table S.5.4 Plausible TGA/MS mass assignments.

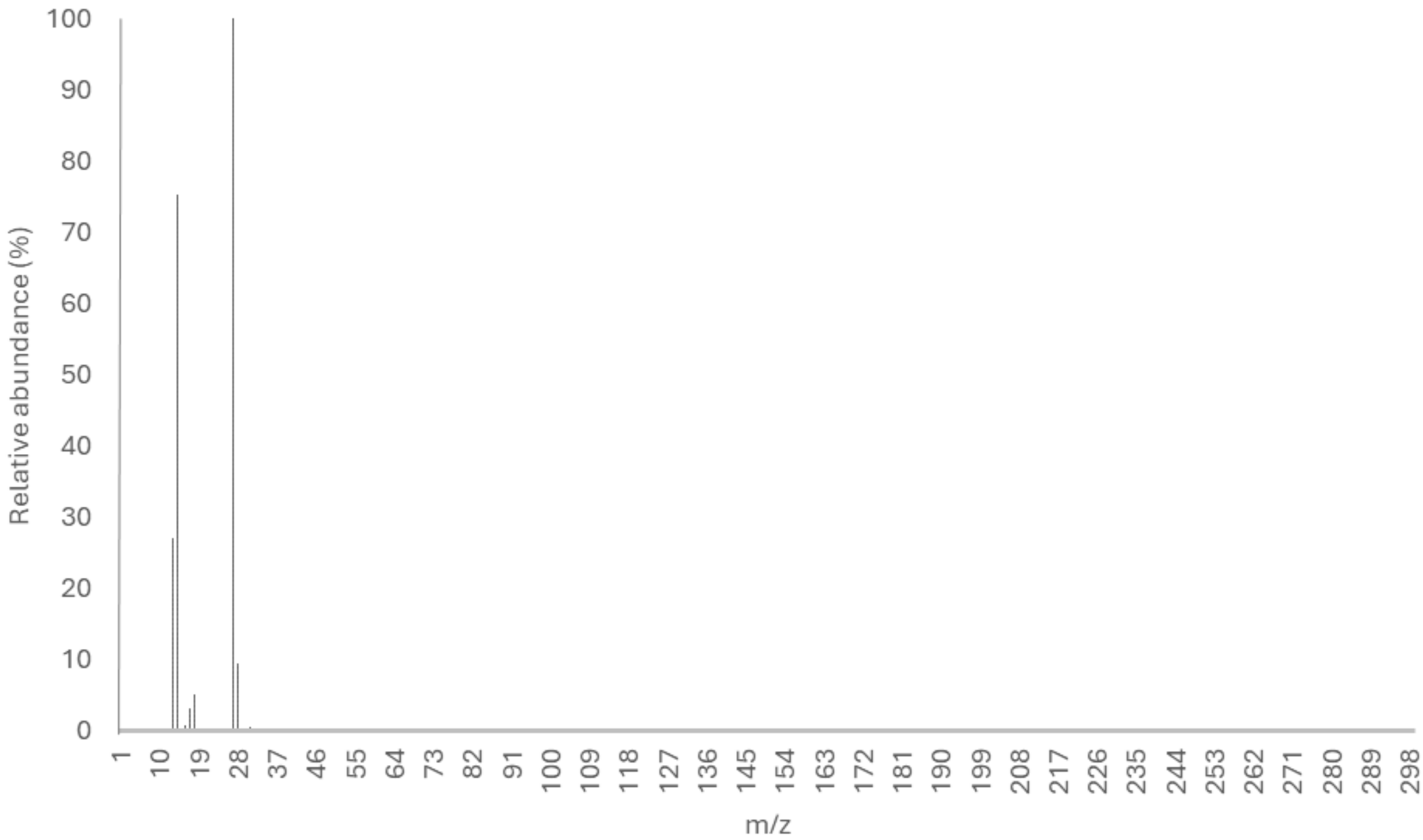

Figure S.5.28 Mass spectrum of products of decomposition formed during TGA analysis of PVDF taken at 475 °C.

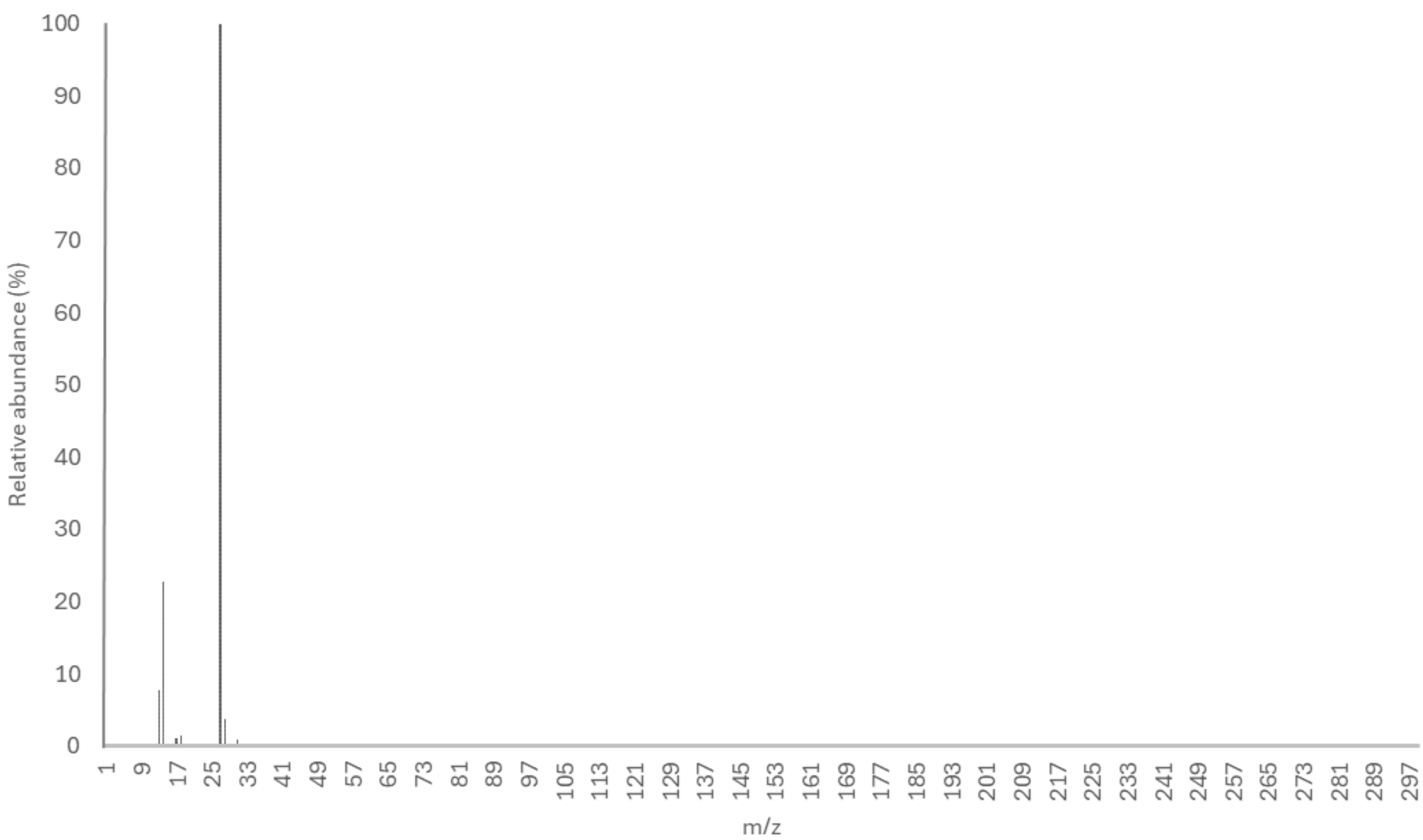

Figure S.5.29 Mass spectrum of products of decomposition formed during TGA analysis of ***poly-1*** taken at 250 °C.

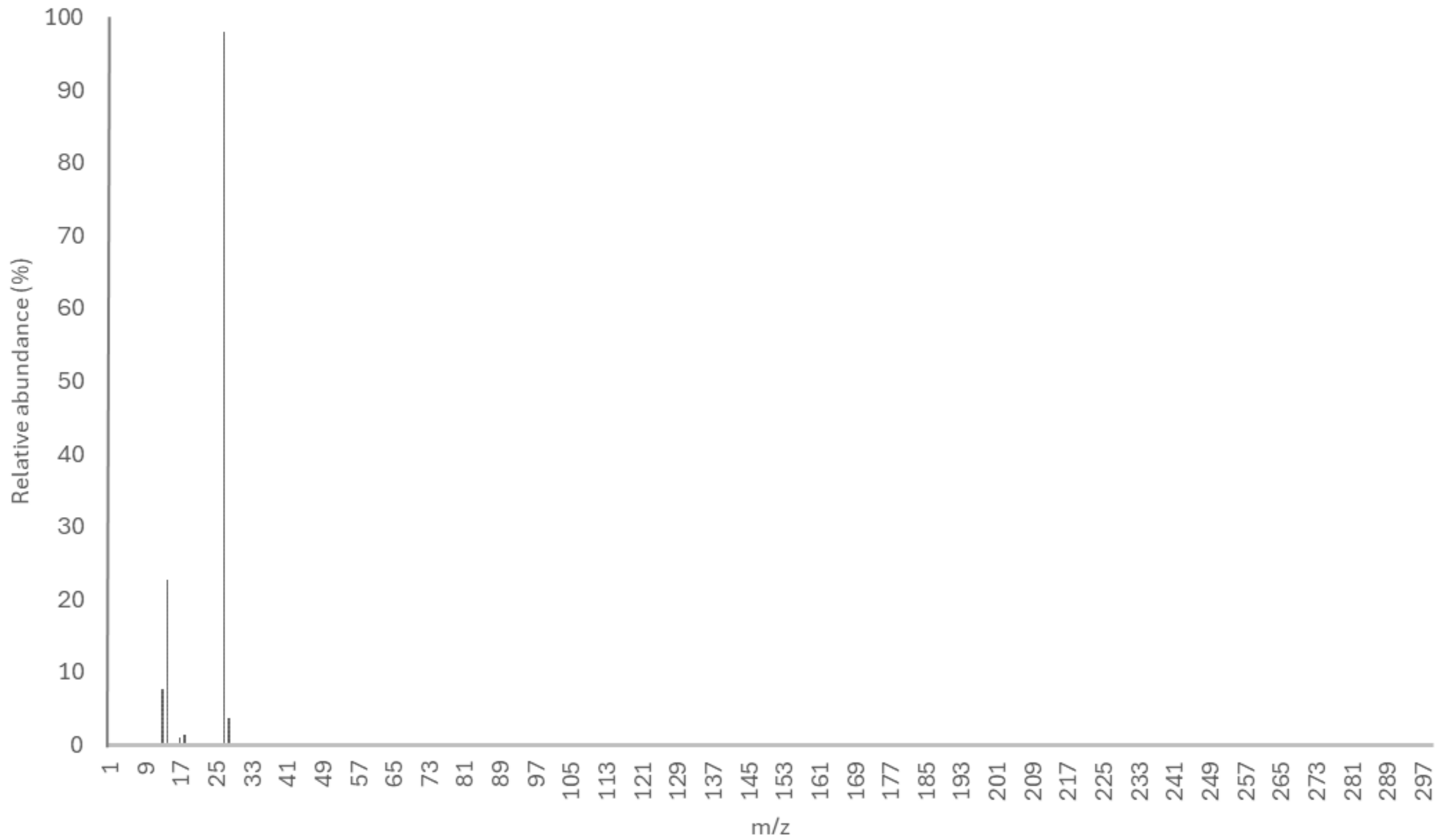

Figure S.5.30 Mass spectrum of products of decomposition formed during TGA analysis of ***poly-1*** taken at 450 °C.

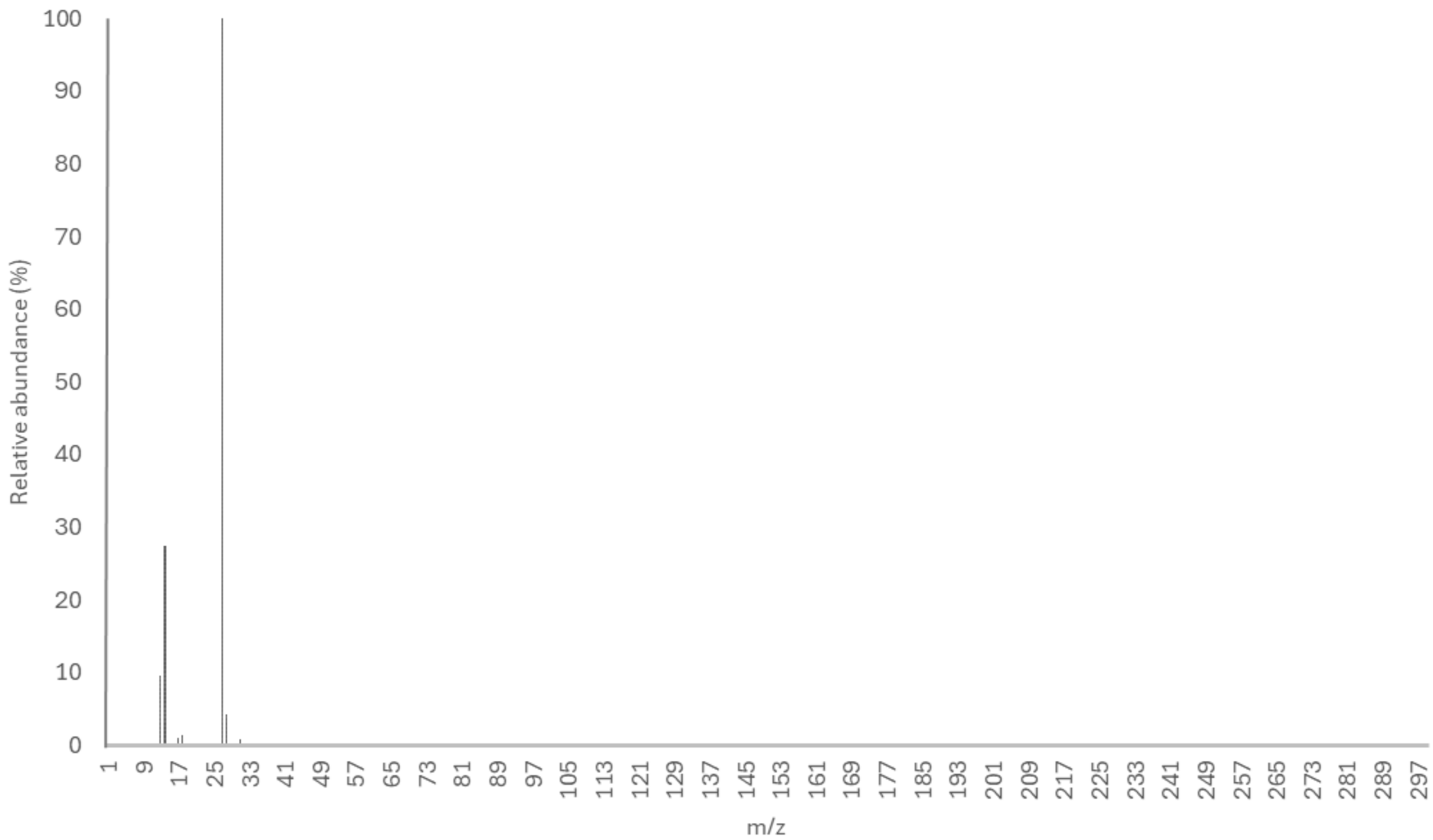

Figure S.5.31 Mass spectrum of products of decomposition formed during TGA analysis of ***poly-2*** taken at 250 °C.

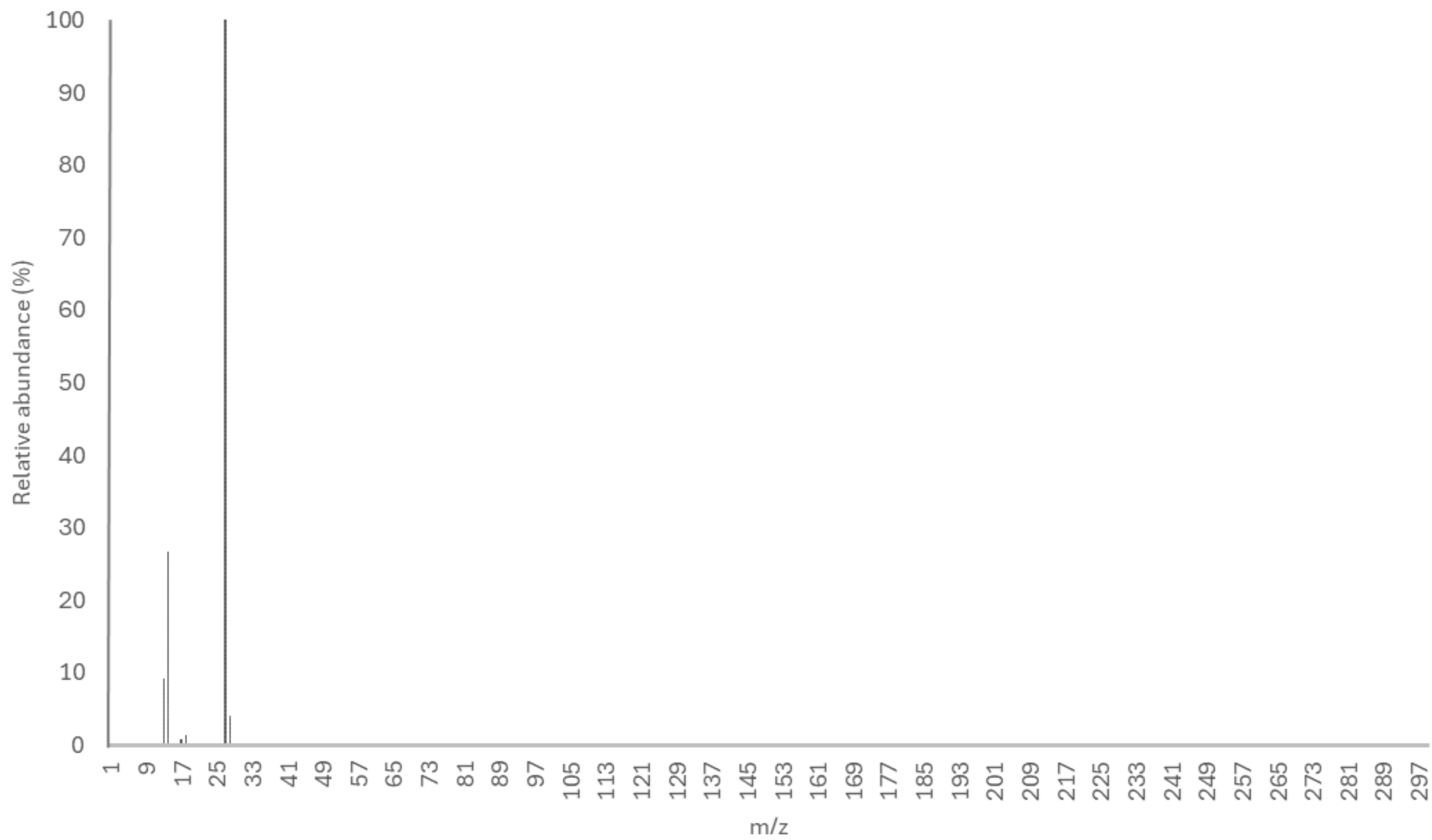

Figure S.5.32 Mass spectrum of products of decomposition formed during TGA analysis of ***poly-2*** taken at 450 °C.

5.6 Elemental analysis

	Carbon (%)	Hydrogen (%)	Nitrogen (%)
PVDF (M_w = 534,000)	37.52	2.71	0.18
poly-1	47.07	4.16	0.00
poly-2	61.47	5.18	0.00
PVDF (M_w = 180,000)	39.45	3.55	1.74
poly-s5	60.49	4.72	0.00
PVDF-co-HFP	36.16	3.12	0.00
poly-s8	52.54	2.28	0.00

Table S.5.5 Elemental analysis of polymer starting materials and their corresponding products of the shuttle catalysis reaction.

6 Computational details

6.1 Methods

All DFT calculations were carried out using Gaussian16 (Revision C.01).[16] Geometry optimisations were performed using the hybrid exchange-correlation B3LYP functional including D3BJ dispersion corrections described by Grimme's D3 corrections with added Becke-Johnson dampening.[171819] A functional screen was performed using the following functionals: ωB97X-D, B3PW91, PBE1PBE and M062x. The triple-ζ basis set with double polarisation and double additional diffuse functions 6-311++g** was used for geometry optimisations (BS1) and single point energy corrections of the optimised structures was performed using the larger quadruple--ζ basis set with double polarisation and diffuse functions def2-QZVPPD (BS2, see below).[2021]

Geometry optimisations were performed without symmetry constraints (nosymm) and an improved numerical integration grid using a pruned grid with 99 radial shells and 590 angular points per shell (int=ultrafine) was used. Solvation was added to geometry optimisations using the toluene as the solvent using the polarisable continuum model (PCM).[22] Single point energy corrections used toluene as the solvent using universal solvation model (SMD).[23]

BS2 is not natively incorporated into the Gaussian suite of programmes, so this basis set was defined manually. The specific functions for each element (C, H, O, B, P and F) were obtained from the Basis Set Exchange (https://www.basissetexchange.org/).[24] Each element was defined as follows:

Hydrogen

```
S   4   1.00
    190.6916900          0.70815167D-03
     28.6055320          0.54678827D-02
      6.5095943          0.27966605D-01
      1.8412455          0.10764538
S   1   1.00
      0.59853725         1.0000000
S   1   1.00
      0.21397624         1.0000000
S   1   1.00
      0.80316286D-01     1.0000000
P   1   1.00
      2.29200000         1.0000000
P   1   1.00
      0.83800000         1.0000000
P   1   1.00
      0.29200000         1.0000000
P   1   1.00
      0.84063199228D-01  1.0000000
D   1   1.00
      2.06200000         1.0000000
D   1   1.00
      0.66200000         1.0000000
F   1   1.00
      1.39700000         1.0000000
```

Boron
S 8 1.00
46447.6670560 0.38388681014D-04
6957.6889042 0.29836770197D-03
1583.4428403 0.15645478806D-02
448.46601009 0.65476770742D-02
146.28639262 0.23139008388D-01
52.784386084 0.69615799357D-01
20.519396170 0.17119636876
8.4185659258 0.31913191837
S 2 1.00
36.510018312 0.78990617244D-01
10.541854005 0.78926384265
S 1 1.00
3.6004091387 1.0000000
S 1 1.00
1.5617023749 1.0000000
S 1 1.00
0.44997370775 1.0000000
S 1 1.00
0.18075230237 1.0000000
S 1 1.00
0.71596696319D-01 1.0000000
S 1 1.00
0.29941402533D-01 1.0000000
P 5 1.00
72.240462760 0.86539170411D-03
16.807707208 0.68762067365D-02
5.2259410747 0.30976687148D-01
1.8508350671 0.10432357913
0.72206782663 0.26164137577
P 1 1.00
0.29491018056 1.0000000
P 1 1.00
0.12201141839 1.0000000
P 1 1.00
0.49865393625D-01 1.0000000
D 1 1.00
1.11000000 1.0000000
D 1 1.00
0.40200000 1.0000000
D 1 1.00
0.14500000 1.0000000
D 1 1.00
0.50896593879D-01 1.0000000
F 1 1.00
0.88200000 1.0000000
F 1 1.00
0.31100000 1.0000000
G 1 1.00
0.67300000 1.0000000

Carbon
S 8 1.00
67025.0710290 0.38736308501D-04
10039.9865380 0.30107917575D-03
2284.9316911 0.15787918095D-02
647.14122130 0.66087087195D-02
211.09472335 0.23367123250D-01
76.177643862 0.70420716898D-01
29.633839163 0.17360344953
12.187785081 0.32292305648
S 2 1.00
53.026006299 0.74897404492D-01
15.258502776 0.76136220983
S 1 1.00
5.2403957464 1.0000000
S 1 1.00
2.2905022379 1.0000000
S 1 1.00
0.69673283006 1.0000000
S 1 1.00
0.27599337363 1.0000000
S 1 1.00
0.10739884389 1.0000000
S 1 1.00
0.44981404899D-01 1.0000000
P 5 1.00
105.12555082 0.84647553844D-03
24.884461066 0.66274038534D-02
7.8637230826 0.30120390419D-01
2.8407001835 0.99951435476D-01
1.1227137335 0.23826299282
P 1 1.00
0.46050725555 1.0000000
P 1 1.00
0.18937530913 1.0000000
P 1 1.00
0.75983791611D-01 1.0000000
D 1 1.00
1.84800000 1.0000000
D 1 1.00
0.64900000 1.0000000
D 1 1.00
0.22800000 1.0000000
D 1 1.00
0.76889830417D-01 1.0000000
F 1 1.00
1.41900000 1.0000000
F 1 1.00
0.48500000 1.0000000

```
G   1   1.00
     1.01100000           1.0000000
```

Oxygen

```
S   8   1.00
 116506.4690800           0.40383857939D-04
  17504.3497240           0.31255139004D-03
   3993.4513230           0.16341473495D-02
   1133.0063186           0.68283224757D-02
    369.99569594          0.24124410221D-01
    133.62074349          0.72730206154D-01
     52.035643649         0.17934429892
     21.461939313         0.33059588895
S   2   1.00
     89.835051252         0.96468652996D-01
     26.428010844         0.94117481120
S   1   1.00
     9.2822824649         1.0000000
S   1   1.00
     4.0947728533         1.0000000
S   1   1.00
     1.3255349078         1.0000000
S   1   1.00
     0.51877230787        1.0000000
S   1   1.00
     0.19772676454        1.0000000
S   1   1.00
     0.69638535104D-01    1.0000000
P   5   1.00
    191.15255810          0.25115697705D-02
     45.233356739         0.20039240864D-01
     14.353465922         0.93609064762D-01
     5.2422371832         0.30618127124
     2.0792418599         0.67810501439
P   1   1.00
     0.84282371424        1.0000000
P   1   1.00
     0.33617694891        1.0000000
P   1   1.00
     0.12863997974        1.0000000
P   1   1.00
     0.43598162776D-01    1.0000000
D   1   1.00
     3.77500000           1.0000000
D   1   1.00
     1.30000000           1.0000000
D   1   1.00
     0.44400000           1.0000000
D   1   1.00
     0.12546378695        1.0000000
F   1   1.00
     2.66600000           1.0000000
F   1   1.00
     0.85900000           1.0000000
G   1   1.00
     1.84600000           1.0000000
```

Fluorine

```
S   8   1.00
 132535.9734500           0.47387482743D-04
  19758.1125880           0.37070120897D-03
   4485.1996947           0.19450784713D-02
   1273.8151020           0.80573291994D-02
    418.93831236          0.27992880781D-01
    152.55721985          0.82735120175D-01
     59.821524823         0.19854169012
     24.819076932         0.34860632233
S   2   1.00
    100.74446673          0.10505068816
     30.103728290         0.94068472434
S   1   1.00
     10.814283272         1.0000000
S   1   1.00
     4.8172886770         1.0000000
S   1   1.00
     1.6559334213         1.0000000
S   1   1.00
     0.64893519582        1.0000000
S   1   1.00
     0.24778104545        1.0000000
S   1   1.00
     0.87626236800D-01    1.0000000
P   5   1.00
    240.96654114          0.30389933451D-02
     57.020699781         0.24357738582D-01
     18.126952120         0.11442925768
     6.6457404621         0.37064659853
     2.6375722892         0.79791551766
P   1   1.00
     1.0638217200         1.0000000
P   1   1.00
     0.41932562750        1.0000000
P   1   1.00
     0.15747588299        1.0000000
P   1   1.00
     0.46772400332D-01    1.0000000
D   1   1.00
     5.01400000           1.0000000
D   1   1.00
     1.72500000           1.0000000
D   1   1.00
     0.58600000           1.0000000
```

D 1 1.00
0.15967986245 1.0000000
F 1 1.00
3.56200000 1.0000000
F 1 1.00
1.14800000 1.0000000
G 1 1.00
2.37600000 1.0000000

Phosphorus
S 10 1.00
1090561.7138000 0.12142449664D-04
163316.3946100 0.94395292614D-04
37166.6074510 0.49622262177D-03
10526.8809450 0.20900040747D-02
3433.9976028 0.75489230025D-02
1239.5360480 0.24010423937D-01
483.27456199 0.67231473697D-01
200.16911586 0.15978669881
86.960394829 0.29735906782
39.211283369 0.36187171850
S 3 1.00
336.75883662 0.19154721050D-01
103.72179793 0.17134079100
39.771861240 0.63689655985
S 1 1.00
17.888612952 1.0000000
S 1 1.00
6.9644556879 1.0000000
S 1 1.00
3.2198092087 1.0000000
S 1 1.00
1.4669943979 1.0000000
S 1 1.00
0.47765437532 1.0000000
S 1 1.00
0.21637789241 1.0000000
S 1 1.00
0.90235894336D-01 1.0000000
S 1 1.00
0.40836372978D-01 1.0000000
P 8 1.00
2019.6711374 0.21359172406D-03
478.60125090 0.18568771399D-02
155.14942504 0.10070690115D-01
58.816356575 0.39605153679D-01
24.544512785 0.11736067844
10.883571061 0.24950540642
4.9624791285 0.36421287984
2.3002912343 0.31764127123
P 2 1.00
59.371345016 0.39432918004
3.0694590986 -6.3522960431
P 1 1.00
1.0634401739 1.0000000
P 1 1.00
0.45022152161 1.0000000
P 1 1.00
0.18267271344 1.0000000
P 1 1.00
0.71610333771D-01 1.0000000
D 1 1.00
3.34300000 1.0000000
D 1 1.00
0.807000000 1.0000000
D 1 1.00
0.365000000 1.0000000
D 1 1.00
0.154000000 1.0000000
D 1 1.00
0.59687731846D-01 1.0000000
F 1 1.00
0.703000000 1.0000000
F 1 1.00
0.280000000 1.0000000
G 1 1.00
0.597000000 1.0000000

Frequency analysis for all stationary points were performed using BS1 to confirm the nature of the structures as either minima (no imaginary frequencies) or transition states (precisely one imaginary frequency). Intrinsic reaction coordinate (IRC) calculations followed by full geometry

optimisations on final points were used to connect transition states and minima located on the potential energy surface.

Thermal corrections were applied using Paton's GoodVibes software, which incorporates Grimme's quasi-harmonic approximation to the vibration entropy below a cut-off of 100 cm^{-1} and a frequency scaling factor = 1.0.[25] The entropic terms for frequencies below the cut-off are obtained from the free rotor approximation while the standard rigid-rotor harmonic oscillator (RRHO) approximation is retained for those above the cut-off. A dampening function is used to interpolate between these two expressions close to the cut-off. To account for experimental conditions, a temperature of 373.15 K and concentrations of 0.270 mol L^{-1} (fluoroethane), 0.0901 mol L^{-1} (benzoic anhydride), and 0.00901 mol L^{-1} (catalyst species) were also applied.

6.2 Reaction pathway for the boron trifluoride diethyl etherate and boron trifluoride tricyclohexylphosphine catalysed reaction of fluoroethane and benzoic anhydride

6.2.1 Description of possible pathways

Following the procedure we previously described, the shuttle catalysis reaction pathway was modelled as a sequence of defluorination of a fluoroalkane followed by HF addition to benzoic anhydride. Overall, this results in the formation of benzoyl fluoride and benzoic acid.

Fluoroethane was chosen as a model fluoroalkane to reduce the effect of conformational flexibility and reproduce experimental conditions using fluoroethane as an HF donor. Three pathways were modelled for the defluorination pathway – one involving BF_3 acting to abstract both hydrogen (**TS-S1a = TS-1**) and fluorine from fluoroethane in a concerted step, and two involving the explicit modelling of the OEt_2 or PCy_3. In explicit modelling of OEt_2 and PCy_3, two pathways of defluorination were considered – a *cis* (*gauche*) and *trans* (*anti*) approach for defluorination of the fluoroalkane, where the OEt_2 or PCy_3 acts to abstract hydrogen (**TS-s1b**-**TS-s1e**) All three pathways were modelled with both $BF_3{\cdot}OEt_2$ and $BF_3{\cdot}PCy_3$ acting as sole catalysts. In all cases, pathways involving $BF_3{\cdot}PCy_3$ were higher in energy, likely a result of the higher energy for dissociation of BF_3 and PCy_3. Defluorination therefore occurs through **TS-s1a** (**TS-1**), which does not involve explicit modelling of the OEt_2, generating $[BF_4][HOEt_2]$ (**Int-s2a**) as an intermediate. The generation of $[BF_4][HPCy_3]$ (**Int-s2b**) is significantly more energetically favourable, in line with experimental conditions.

Hydrofluorination of benzoic anhydride was modelled through three possible pathways. One involved protonation occurring before fluorination without a defined transition state to generate a protonated intermediate (**Int-s3**). Fluorination was then mediated by a $^{-}BF_4$ anion (**TS-s2 = TS2**) without explicit modelling of OEt_2 or PCy_3, with fluorination occurring at the protonated carbonyl. Proton exchange and C–O bond cleave occur in a concerted transition state (**TS-s3 = TS3**) to liberate benzoyl fluoride and benzoic acid. This pathway was also found to be higher in energy when mediated by

$[BF_4][HPCy_3]$than $[BF_4][HOEt_2]$, likely as a result of the higher barrier for proton transfer from $[HPCy_3]^+$ than from $[HOEt_2]^+$ as a stronger Lewis base.

Pathways were also modelled involving explicit solvation of OEt_2 and PCy_3. A pathway involving explicit modelling of OEt_2 showed an initial protonation event (**TS-s5a**) resulting in C–O bond cleavage, followed by fluorination at the non-protonated carbonyl (**TS-s6a**). Although higher in energy than the pathway omitting explicit modelling of OEt_2, this pathway is low enough in energy to also be considered operational. A transition state involving explicit modelling of OEt_2 with fluorination occurring at the protonated carbonyl could not be located. Conversely, a pathway involving explicit modelling of PCy_3 showed protonation (**TS-s4b**) resulting in the formation of a protonated anhydride intermediate (**Int-s6b**). Proton exchange and C–O bond cleavage then occur in a concerted transition state (**TS-s5b**) liberating benzoic acid and benzoyl fluoride as products of the reaction. Ultimately, pathways involving $HPCy_3{\cdot}BF_4$ were found to be too high in energy to be in operation. The computed energies for the hydrofluorination and defluorination steps are tabulated in **Tables S.6.1**-**S.6.3** respectively.

	SCF hartrees	G (BS1) hartrees	SCF (BS2) hartrees	G goodvibes hartrees	G (BS2) hartrees	G (BS2) kcal/mol	ΔG kcal/mol
BF_3	-324.667370	-324.679890	-324.736141	-0.021505	-324.757646	-203788.508235	
BF_4	-424.682801	-424.696808	-424.818273	-0.014097	-424.832370	-266586.347852	
OEt_2	-233.753160	-233.646913	-233.800014	0.095493	-233.704521	-146651.807265	
OEt_3	-312.740330	-312.569576	-312.895823	0.158812	-312.737011	-196245.445666	
BF_3·OEt_2	-558.448190	-558.333648	-558.559214	0.101923	-558.457291	-350437.255479	
BF_3 + OEt_2							**-3.2**
HBF_4·OEt_2	-658.951391	-658.828514	-659.081363	0.110589	-658.970774	-413510.420670	
BF_3·PCy_3	-1372.206665	-1371.764455	-1372.425002	0.425920	-1371.999082	-860942.458166	
BF_3 + PCy_3							**7.0**
PCy_3	-1047.495024	-1047.060169	-1047.649417	0.419109	-1047.230308	-657146.967008	
$HPCy_3$·BF_4	-1472.727674	-1472.277493	-1472.968987	0.435022	-1472.533965	-924029.052054	
Fluoroethane	-179.131054	-179.088654	-179.165677	0.037152	-179.128525	-112404.851136	
Ethene	-78.620097	-78.590883	-78.635915	0.025144	-78.610771	-49329.005349	
Benzoic anhydride	-765.475223	-765.314078	-765.624555	0.154442	-765.470113	-480339.768110	
Benzoyl fluoride	-444.991459	-444.921240	-445.078187	0.061570	-445.016617	-279252.155074	
Benzoic acid	-420.978297	-420.895324	-421.060685	0.074329	-420.986356	-264172.937819	
Benzoic ester	-499.625680	-499.491180	-499.724779	0.124937	-499.599842	-313503.647128	
BF_3·OEt_2 + BF_3·PCy_3 + fluoroethane + benzoic anhydride							**0.0**

Table S.6.1 Computed energies for starting materials and products.

	SCF<br>hartrees	G (BS1)<br>hartrees	SCF (BS2)<br>hartrees	G goodvibes<br>hartrees	G (BS2)<br>hartrees	G (BS2)<br>kcal/mol	ΔG<br>kcal/mol
**Int1a**	-503.807047	-503.760708	-503.908545	0.035142	-503.873403	-316185.347430	
**Int-S1a** + OEt_2							**5.0**
**Int-S1a** + PCy_3							**15.0**
**TS-S1a** = **TS-1**	-503.763150	-503.721280	-503.864645	0.030807	-503.833838	-316160.51948	
T**S-S1a** = **TS-1** + OEt_2							**29.8**
**TS-S1a** = **TS1** + PCy_3							**39.8**
$HBF_4 \cdot OEt_2$ + benzoic anhydride							**2.5**
$HPCy_3 \cdot BF_4$ + benzoic anhydride							**-10.7**
**Int-S1b**	-737.563744	-737.398355	-737.709882	0.154571	-737.555311	-462822.964686	**19.1**
**TS-S1b**	-737.535116	-737.368081	-737.679018	0.154737	-737.524281	-462803.492983	**38.6**
**Int-S1c**	-737.564410	-737.400050	-737.711040	0.153948	-737.557092	-462823.713300	**18.0**
**TS-S1c**	-737.528830	-737.363610	-737.675552	0.152602	-737.522950	-462802.657493	**39.4**
**Int-S1d**	-1551.290000	-1550.792600	-1551.541702	0.457050	-1551.084653	-973320.354873	**27.0**
**TS-S1d**	-1551.313500	-1550.814800	-1551.566555	0.508447	-1551.058108	-973303.697828	**43.6**
**Int-S1e**	-1551.311268	-1550.815553	-1551.564893	0.481510	-1551.083383	-973319.558225	**27.8**
**TS-S1e**	-1551.245803	-1550.746892	-1551.500605	0.482553	-1551.018052	-973278.562458	**68.7**
**Int-S2a**	-658.951391	-658.828514	-659.081363	0.110589	-658.970774	-413510.420670	**2.5**
**Int-S2b**	-1472.727674	-1472.277493	-1472.968987	0.435022	-1472.533965	-924029.052054	**-10.7**

Table S.6.2 Computed energies for the defluorination of fluoroethane catalysed by boron trifluoride diethyl etherate or boron trifluoride tricyclohexylphosphine.

	SCF<br>hartrees	G (BS1)<br>hartrees	SCF (BS2)<br>hartrees	G goodvibes<br>hartrees	G (BS2)<br>hartrees	G (BS2)<br>kcal/mol	ΔG<br>kcal/mol
**Int-S3**	-1190.656105	-1190.475400	-1190.888488	0.166904	-1190.721584	-747188.510400	**12.0**
**TS-S2** = **TS-2**	-1190.634382	-1190.453300	-1190.866065	0.161624	-1190.704441	-747177.753300	**22.7**
**Int-S4**	-1190.637481	-1190.459000	-1190.870475	0.165851	-1190.704624	-747177.867700	**22.6**
**TS-S3** = **TS-3**	-1190.620856	-1190.446400	-1190.868271	0.167378	-1190.700893	-747175.526600	**24.9**
Benzoic acid + benzoyl fluoride + ethene + $BF_3 \cdot OEt_2$							**-7.6**
**Int-S5a**	-1424.422476	-1424.119200	-1424.714331	0.292379	-1424.421952	-893837.594400	**14.6**
**Int-S5b**	-2238.227370	-2237.590800	-2238.610518	0.619079	-2237.991439	-2237.991439	**-2.8**
**TS-S4a**	-1424.414793	-1424.111200	-1424.689215	0.288778	-1424.400437	-893824.094100	**30.8**
**TS-S4b**	-2238.150763	-2237.516800	-2238.531488	0.617422	-2237.914066	-2237.914066	**45.7**
**Int-S6a**	-1424.417924	-1424.116226	-1424.691203	0.284144	-1424.407059	-893828.961408	**23.9**
**Int-S6b**	-2238.152328	-2237.519600	-2238.554487	0.616793	-2237.937694	-2237.937694	**30.9**
**TS-S5a**	-1190.644581	-1190.470211	-1190.875429	0.175465	-1190.699964	-747175.538840	**25.5**
**TS-S5b**	-865.943895	-865.773252	-866.111745	0.183317	-865.928428	-543378.315050	**37.2**
Benzoic acid + benzoyl fluoride + ethene + $BF_3 \cdot PCy_3$							**-7.6**

Table S.6.3 Computed energies for hydrofluorination of benzoic anhydride catalysed by boron trifluoride diethyl etherate or boron trifluoride tricyclohexylphosphine.

Several pathways were also modelled to account for the formation of ethyl benzoate observed experimentally. Calculations suggest this pathway begins with the addition of BF_3·PCy_3 across benzoic anhydride (**TS-s6**) to generate a carbanion coordinated to PCy_3 and a carboxylate fragment coordinated to BF_3 (**Int-s8**).

The formation of triethyloxonium tetrafluoroborate (**Int-S10a**) and tricyclohexyl(ethyl)phosphonium tetrafluoroborate (**Int-s10b**) were also observed experimentally. Pathways for the formation of **Int-S10a** (**TS-s7a = TS-4**) and **Int-s10b** (**TS-s7b**) were modelled, involving BF_3 abstracting fluorine, followed by C–O or C–P bond formation respectively. Although this pathway is feasibly low in energy for the formation of triethyloxonium tetrafluoroborate (**Int-s10a**), the pathway for formation of **Int-s10b** is too high to be operational. However, **Int-s10b** is significantly more stable than **Int-s10a** – it is expected conversion occurs between the two, although no TS could be located for this.

Pathways to affect the fluorination and alkylation of **Int-s8a** were then modelled. A pathway was located for the fluorination of **Int-s8b**. It was assumed both fluorination and alkylation pathways occurred independently and simultaneously – the energies of optimised products for the alternative pathway was used to account for atom stoichiometry. Fluorination of **Int-s8b** is mediated by BF_4^- (**TS-s8 = TS-5**), liberating benzoyl fluoride and BF_3·PCy_3

Pathways were also modelled for the alkylation of **Int-S8c**. Two pathways were modelled, one mediated by the triethyloxonium ion generated in **Int-s10a** (**TS-s9a = TS-6**) and one mediated by the tricyclohexyl(ethyl)phosphonium ion generated in **Int-s10b** (**TS-s9b**).

Finally, a pathway was modelled for formation of ethyl benzoate occurring from benzoic acid generated as a product of the shuttle catalysis reaction. Calculations show the benzoic acid can, with BF_3 from the dissociation of BF_3·OEt_2, act to deprotonate a further equivalent of fluoroethane (**TS-s10**), generating ethyl benzoate and $[BF_4][HOEt_2]$. Calculated pathways are tabulated in **Table S.6.4**.

	SCF hartrees	G (BS1) hartrees	SCF (BS2) hartrees	G goodvibes hartrees	G (BS2) hartrees	G (BS2) kcal/mol	ΔG kcal/mol
**Int-S7**	-2137.687195	-2137.057800	-2138.035174	0.597275	-2137.437899	-1341261.518708	**19.6**
**TS-S6**	-2137.661873	-2137.034200	-2138.031850	0.610196	-2137.421654	-1341251.324517	**29.8**
**Int-S8a**	-2137.687040	-2137.056600	-2138.051142	0.611552	-2137.439590	-1341262.579700	**15.1**
**Int-S8b**	-1392.433595	-1391.901500	-1392.665470	0.514247	-1392.151223	-873587.422070	**9.0**
**Int-S8c**	-745.208182	-745.130580	-745.349882	0.065282	-745.284600	-467672.794243	**18.1**
**Int-S9a**	-737.564401	-737.400960	-737.710788	0.153261	-737.557527	-462823.986273	**17.8**
**Int-S9b**	-1551.308167	-1550.811300	-1551.561346	0.470238	-1551.091108	-973323.629801	**22.9**
**TS-S7a = TS-4**	-737.548381	-737.377770	-737.693865	0.158751	-737.535114	-462809.922073	**31.8**
**TS-S7b**	-1551.298460	-1550.798900	-1551.551013	0.476868	-1551.074145	-973312.985435	**33.5**
**Int-S10a**	-737.579129	-737.401790	-737.724366	0.162986	-737.561380	-462826.403810	**15.3**
**Int-S10b**	-1551.389504	-1550.883600	-1551.646358	0.489198	-1551.157160	-973365.078045	**-18.5**
**Int-S11**	-1817.217590	-1816.681200	-1817.539580	0.568256	-1816.971324	-1140165.858477	**16.7**
**TS-S8 = TS-5**	-1817.165237	-1816.637200	-1817.466741	0.512099	-1816.954642	-1140155.390259	**27.1**
**Int-S12**	-1817.171897	-1816.648415	-1817.475794	0.509438	-1816.966356	-1140162.741012	**19.8**
**Int-S13a**	-1058.064192	-1057.796900	-1058.269938	0.253517	-1058.016421	-663914.826325	**21.3**
**Int-S13b**	-1871.867498	-1871.266195	-1872.184049	0.582864	-1871.601185	-1174446.587835	**-5.6**
**TS-S9a = TS-6**	-1058.042956	-1057.775670	-1058.247628	0.242620	-1058.005008	-663907.664810	**28.5**
**TS-S9b**	-1871.791215	-1871.196700	-1872.104579	0.577835	-1871.526744	-1174399.875663	**41.1**

Table S.6.4 Computed energies for formation of ethyl benzoate from reaction of benzoic anhydride with fluoroethane, catalysed by boron trifluoride diethyl etherate and boron trifluoride tricyclohexylphosphine.

	SCF hartrees	G (BS1) hartrees	SCF (BS2) hartrees	G goodvibes hartrees	G (BS2) hartrees	G (BS2) kcal/mol	ΔG kcal/mol
**Int-S14a**	-1058.074806	-1057.819520	-1058.281816	0.243357	-1058.038460	-663928.655764	**7.5**
**Int-S14b**	-1871.822505	-1871.224899	-1871.130128	0.579877	-1871.550251	-1174414.626210	**26.3**
**TS-S10**	-924.774644	-924.624990	-924.997365	0.136291	-924.861074	-580332.348085	**-5.4**
**Int-S13**	-924.815229	-924.664780	-924.997365	0.134477	-924.862888	-580359.785999	**-6.5**

Table S.6.4 (Continued) Computed energies for formation of ethyl benzoate from reaction of benzoic anhydride with fluoroethane, catalysed by boron trifluoride diethyl etherate and boron trifluoride tricyclohexylphosphine.

6.2.2 Key geometrical parameters of intermediates

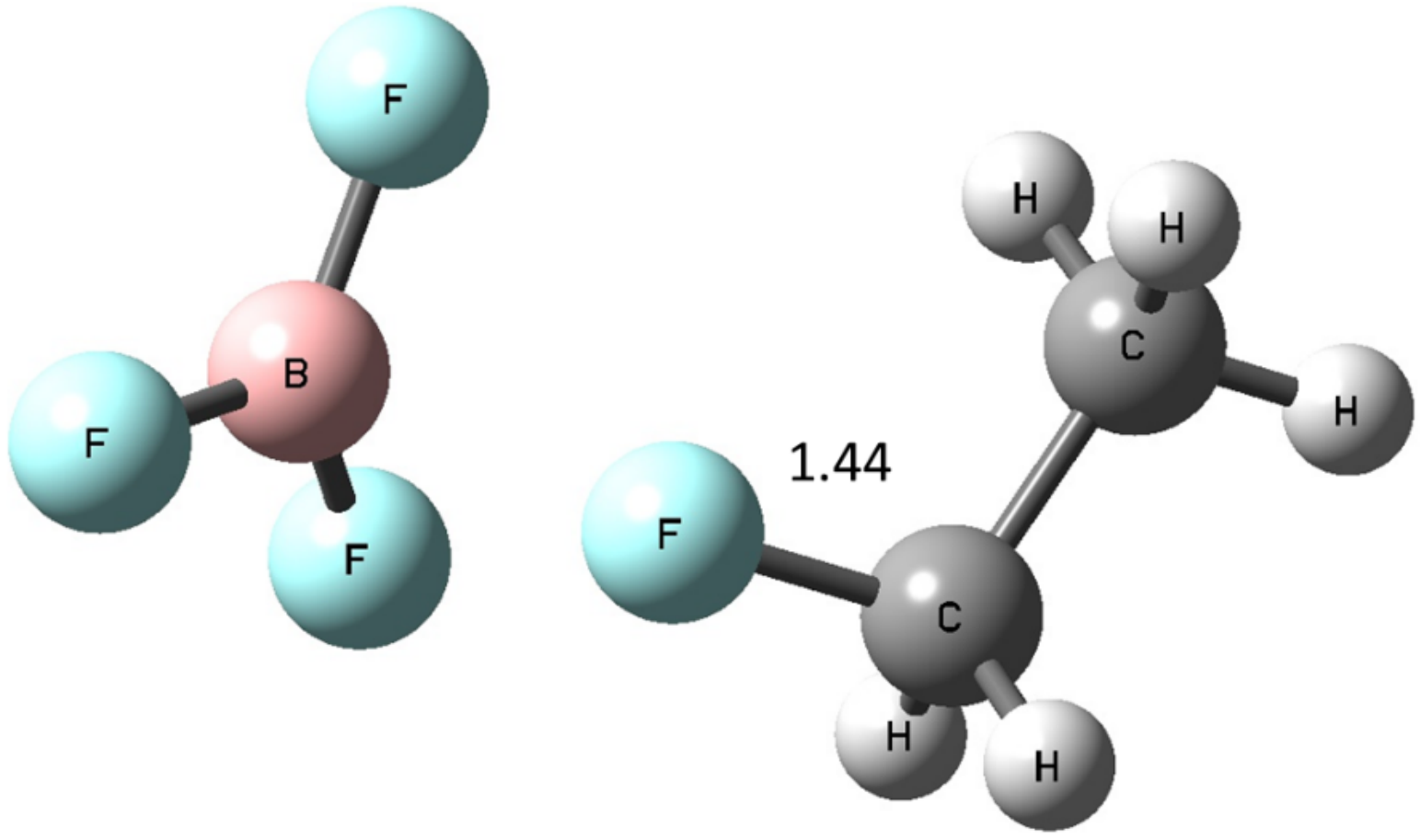

Figure S.6.1 Selected bond lengths (Å) for **Int-s1a**

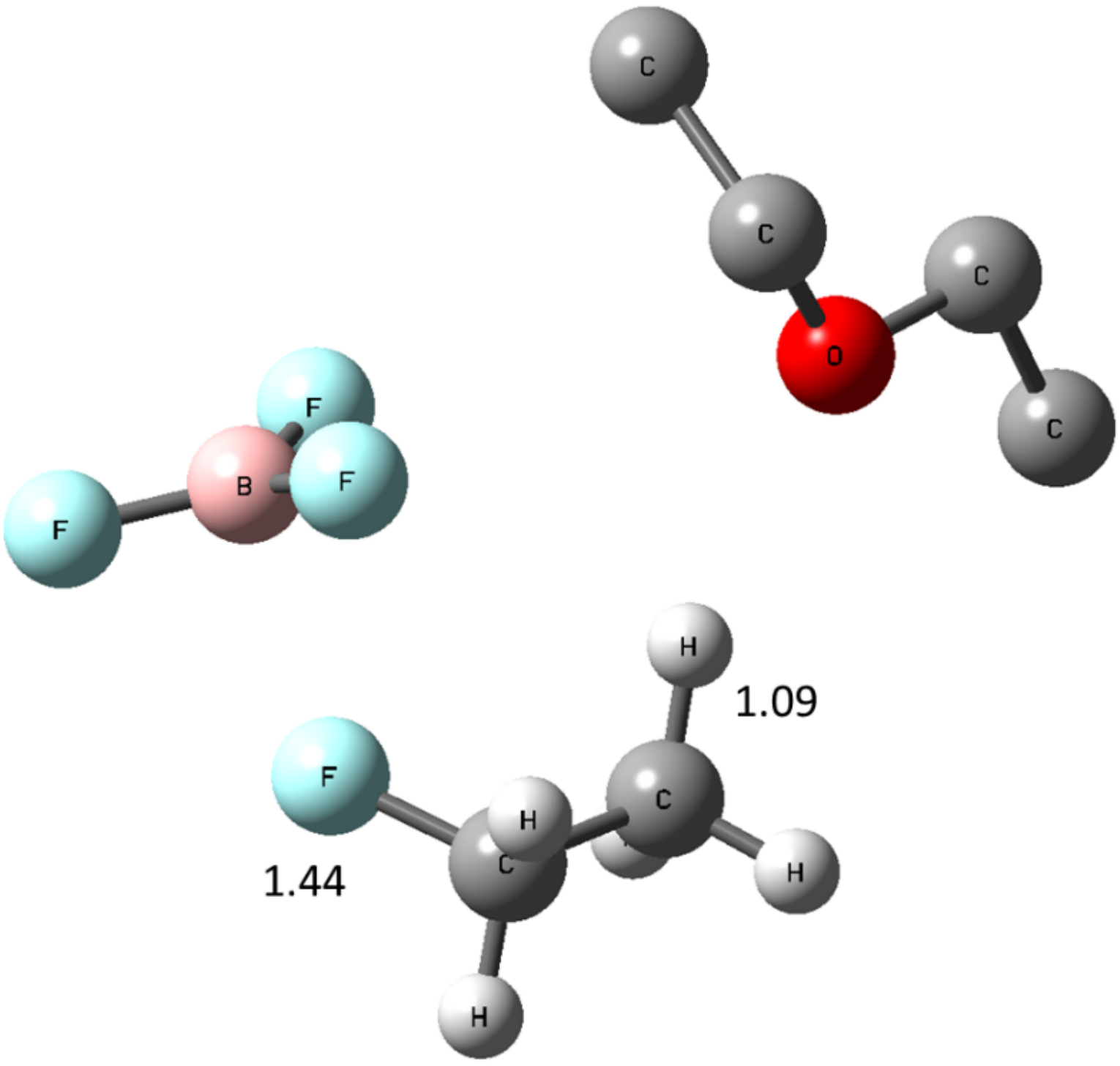

Figure S.6.2 Selected bond lengths (Å) for **Int-s1b**. Some hydrogens have been omitted for clarity.

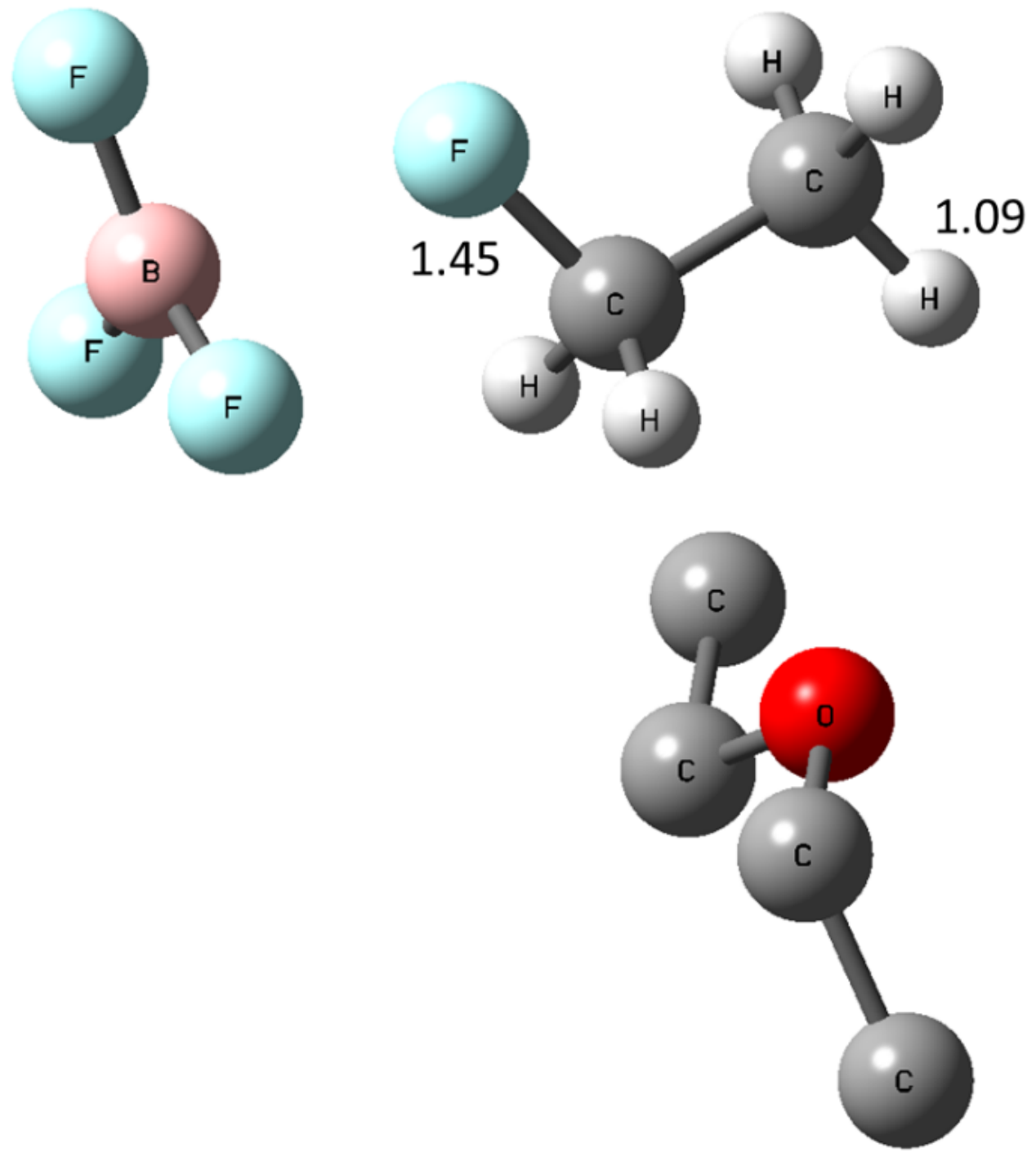

Figure S.6.3 Selected bond lengths (Å) for **Int-s1c**. Some hydrogens have been omitted for clarity.

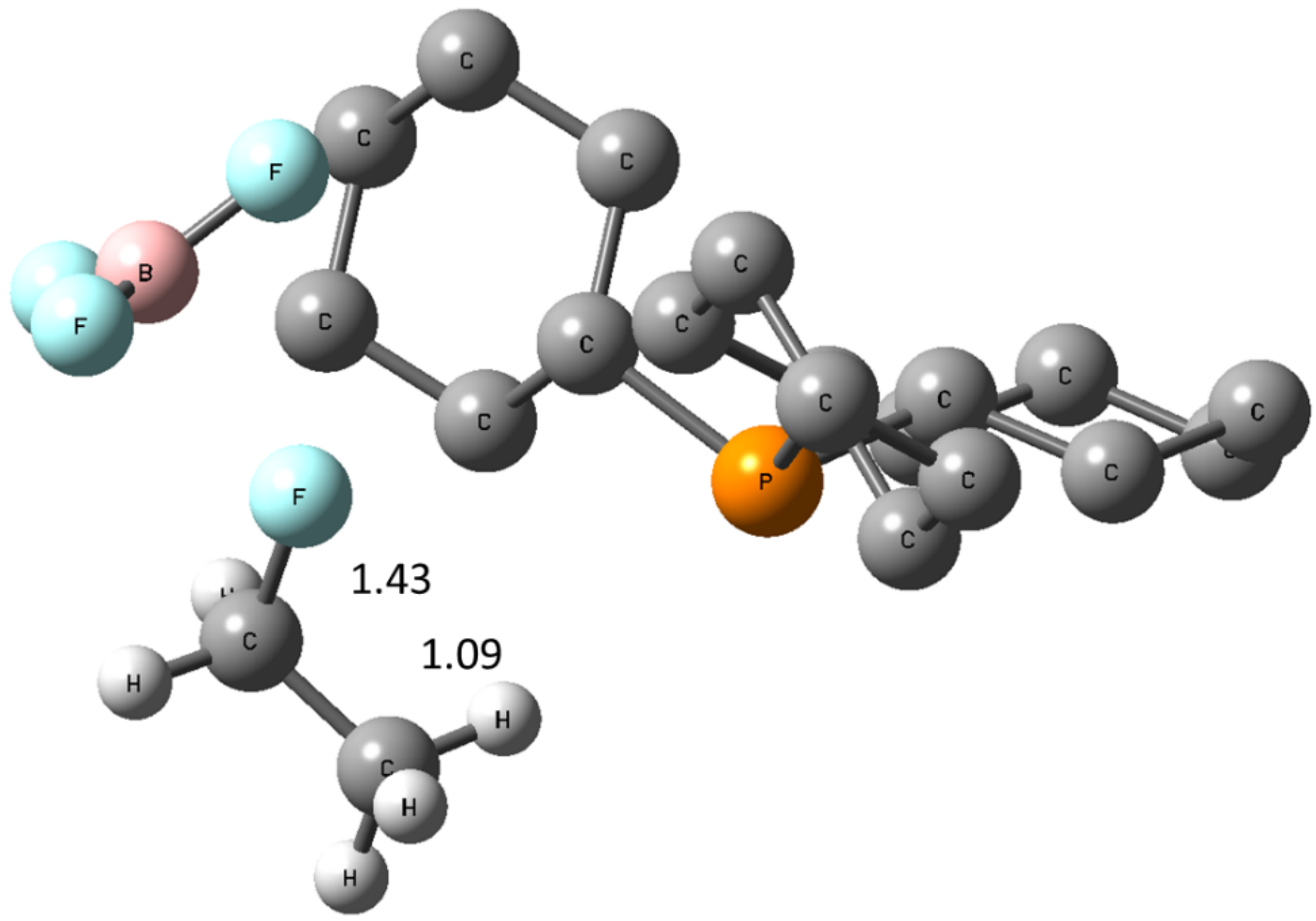

Figure S.6.4 Selected bond lengths (Å) for **Int-s1d**. Some hydrogens have been omitted for clarity.

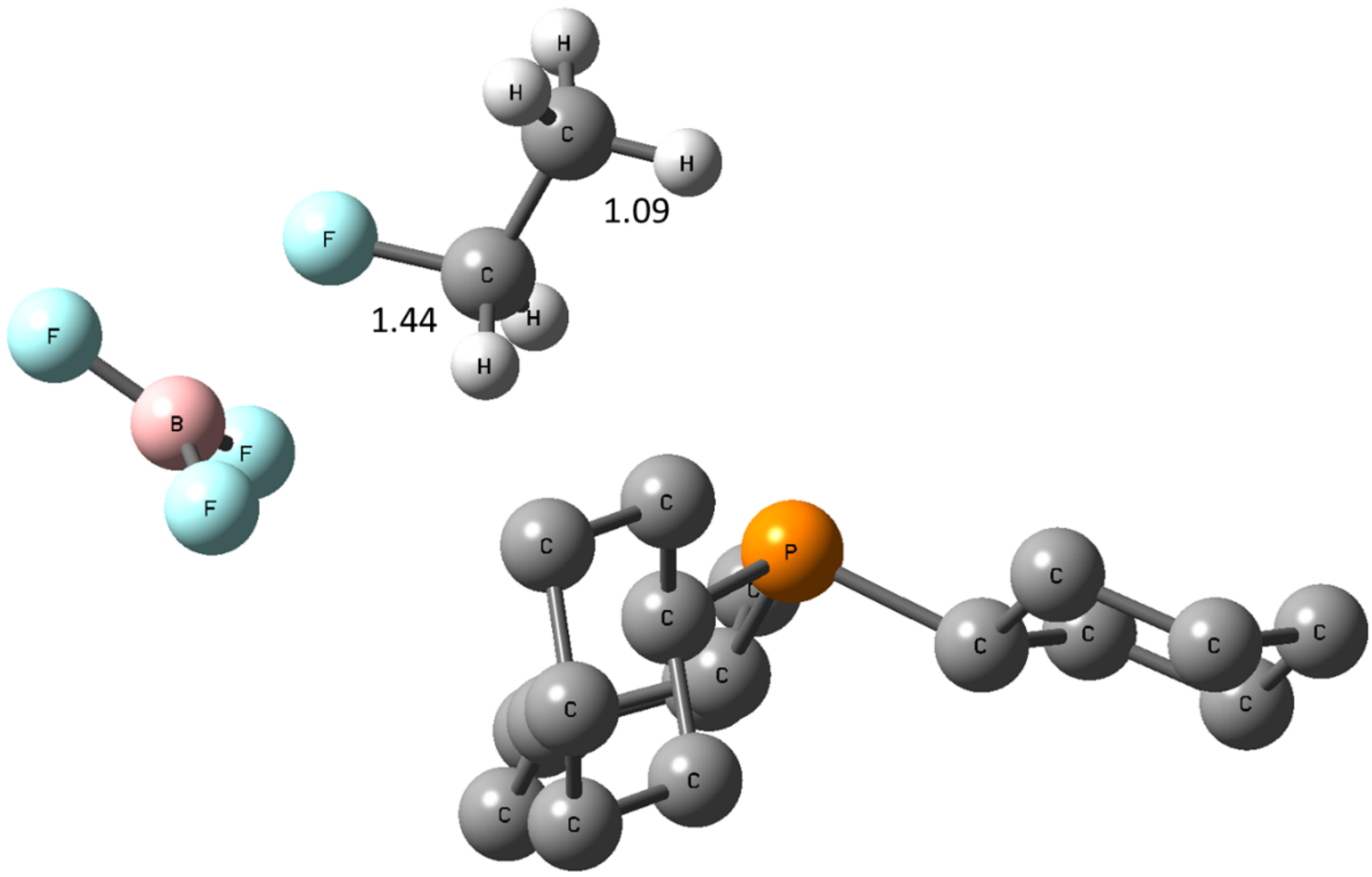

Figure S.6.5 Selected bond lengths (Å) for **Int-s1e**. Some hydrogens have been omitted for clarity.

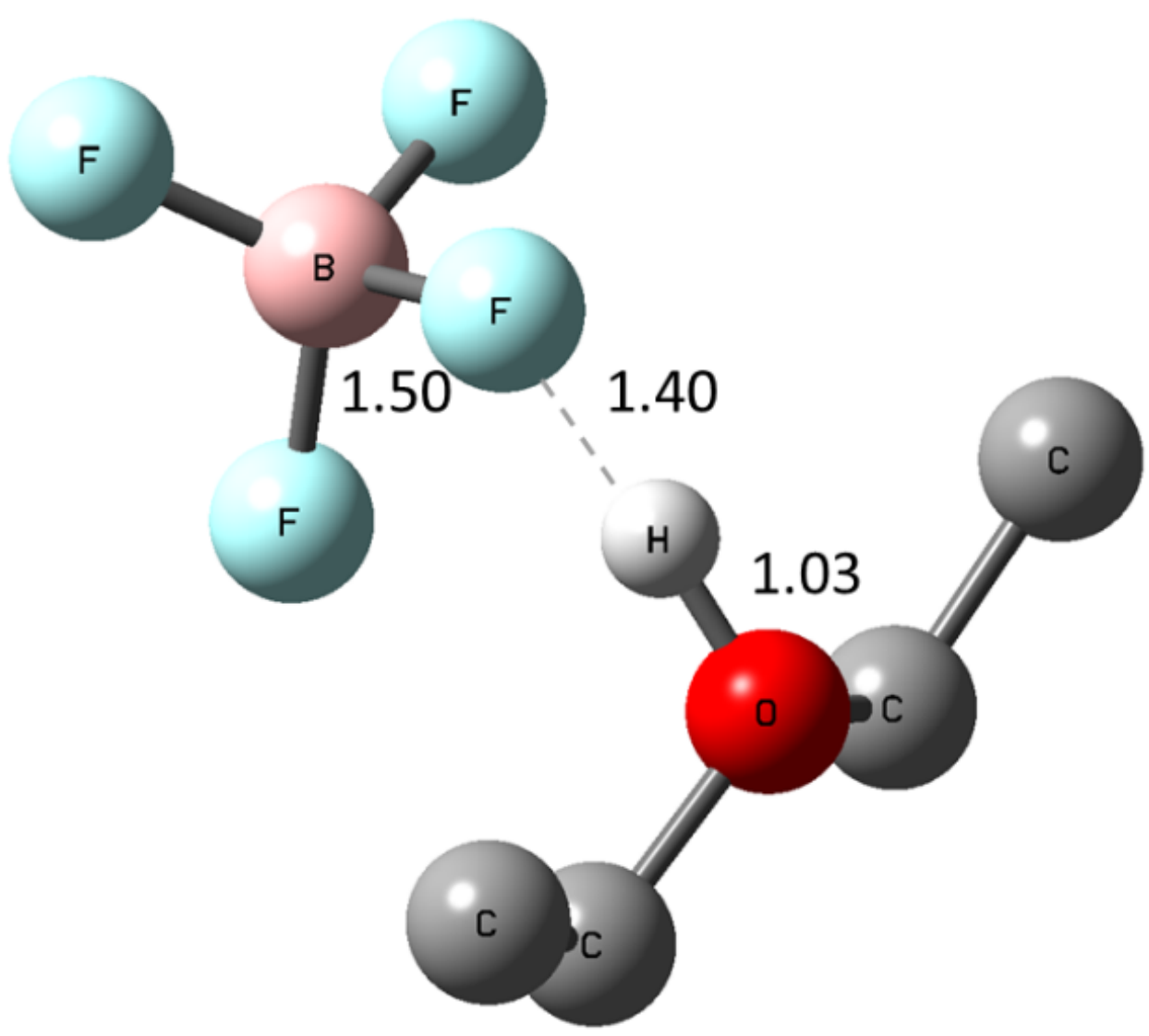

Figure S.6.6 Selected bond lengths (Å) for **Int-s2a**. Some hydrogens have been omitted for clarity.

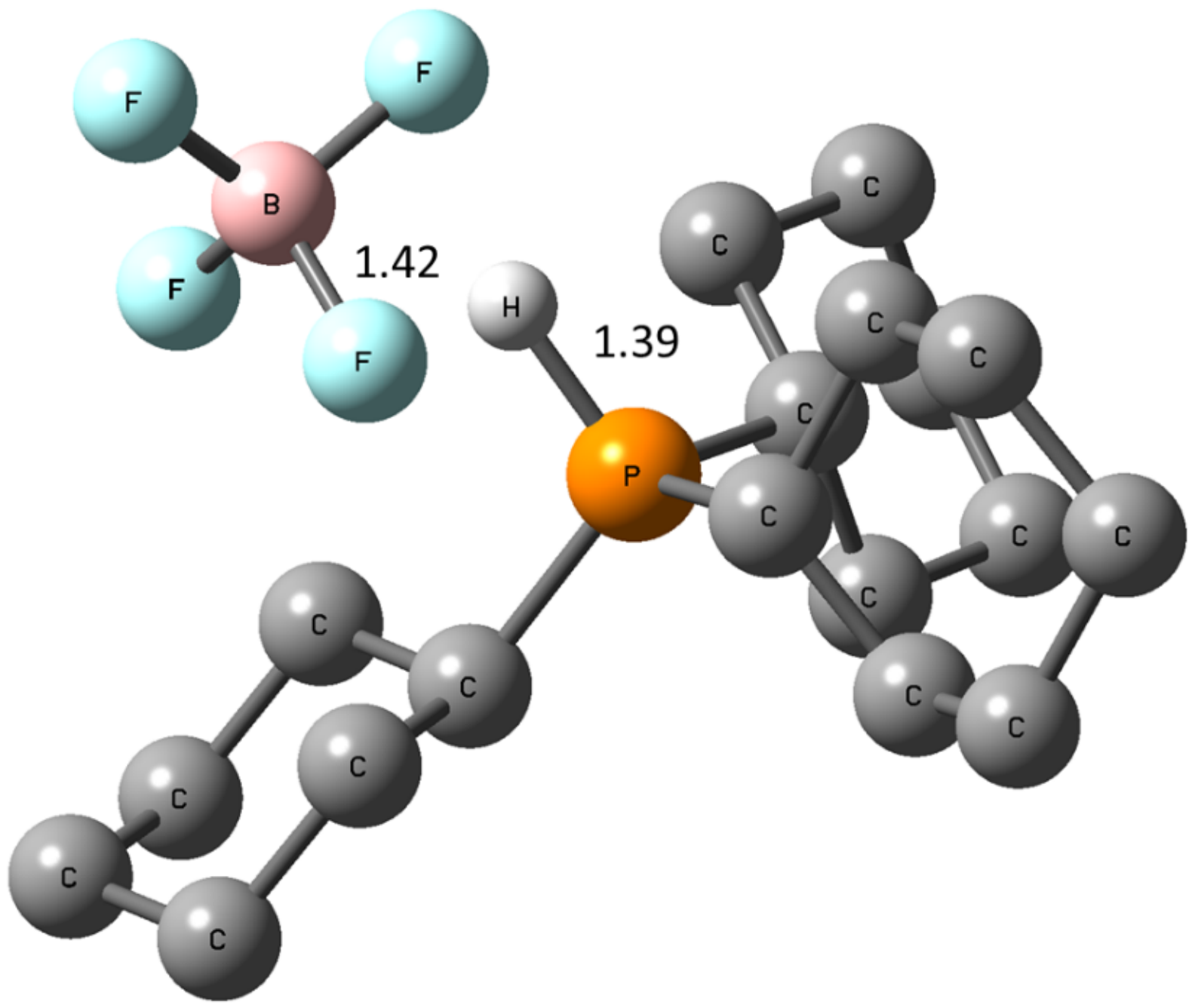

Figure S.6.7 Selected bond lengths (Å) for **Int-s2b**

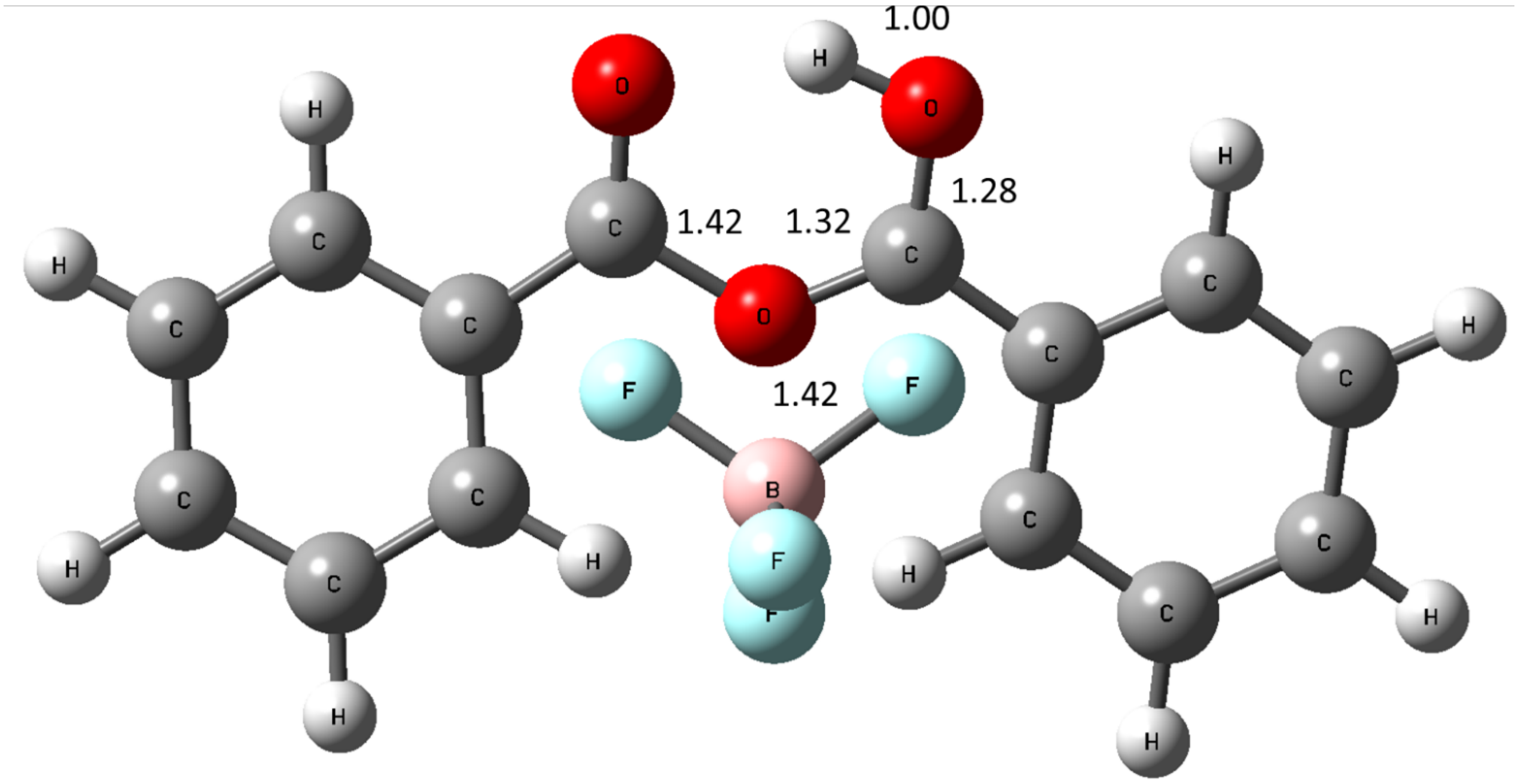

Figure S.6.8 Selected bond lengths (Å) for **Int-s3**.

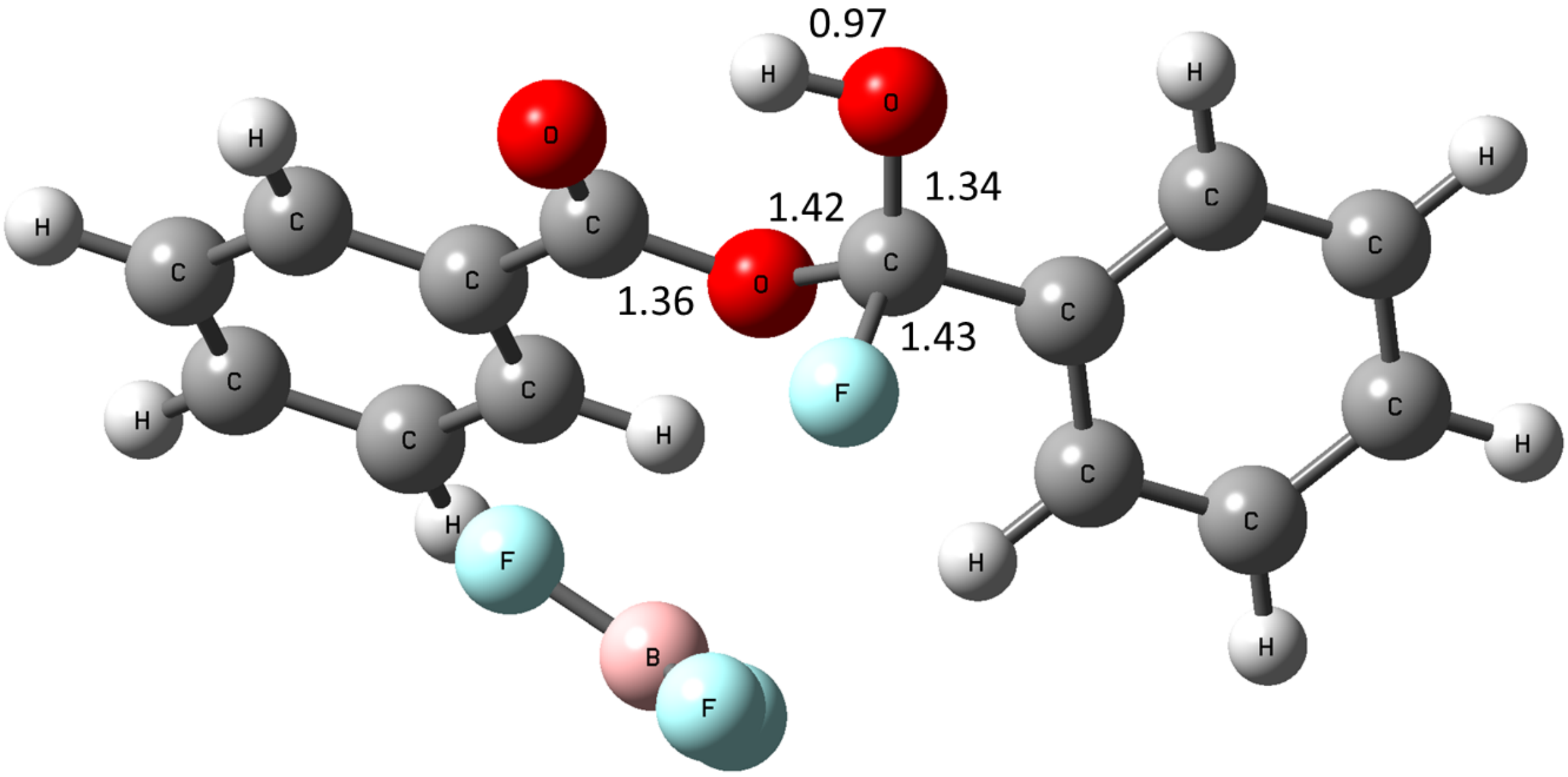

Figure S.6.9 Selected bond lengths (Å) for **Int-s4**.

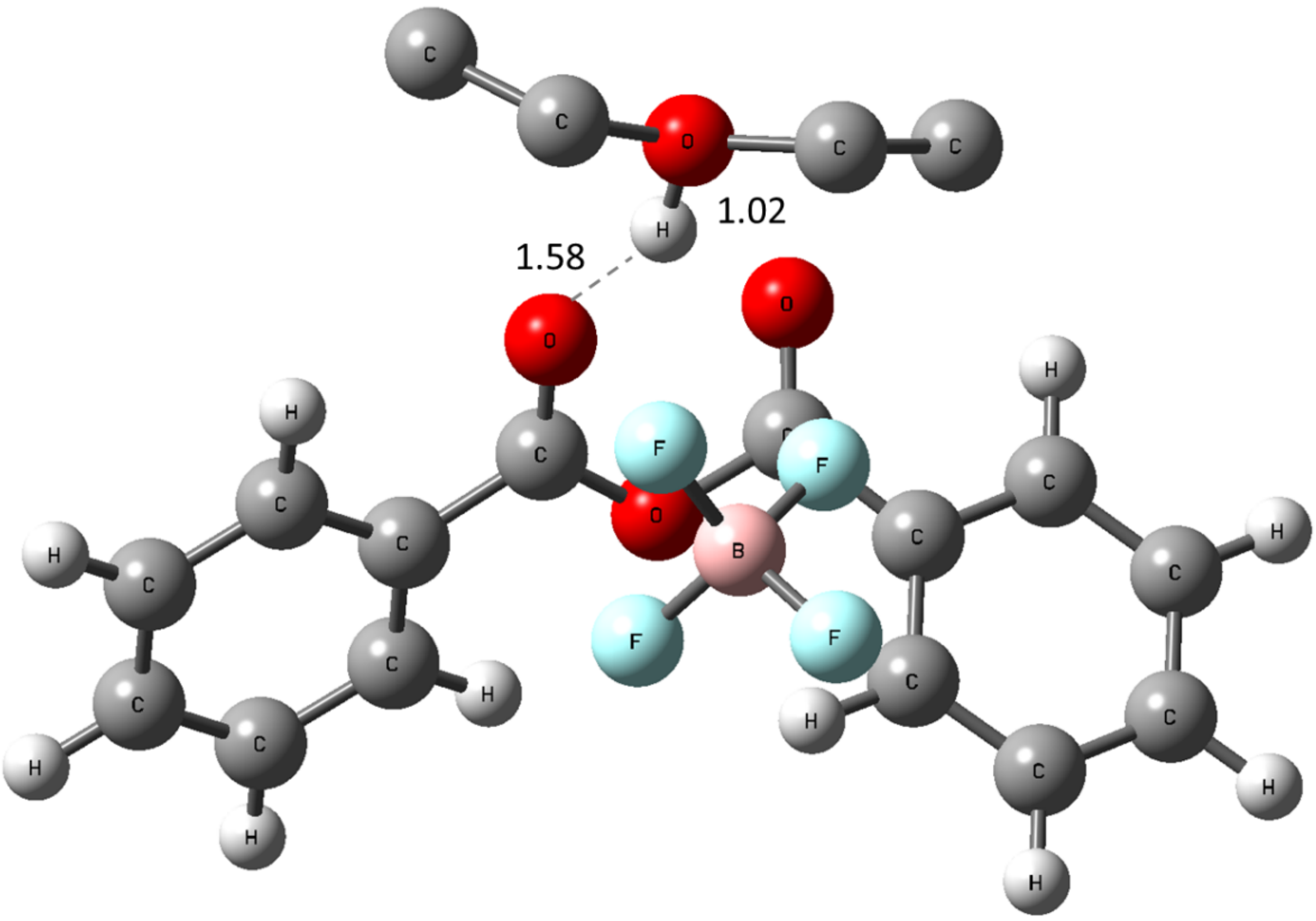

Figure S.6.10 Selected bond lengths (Å) for **Int-s5a**. Some hydrogens have been omitted for clarity.

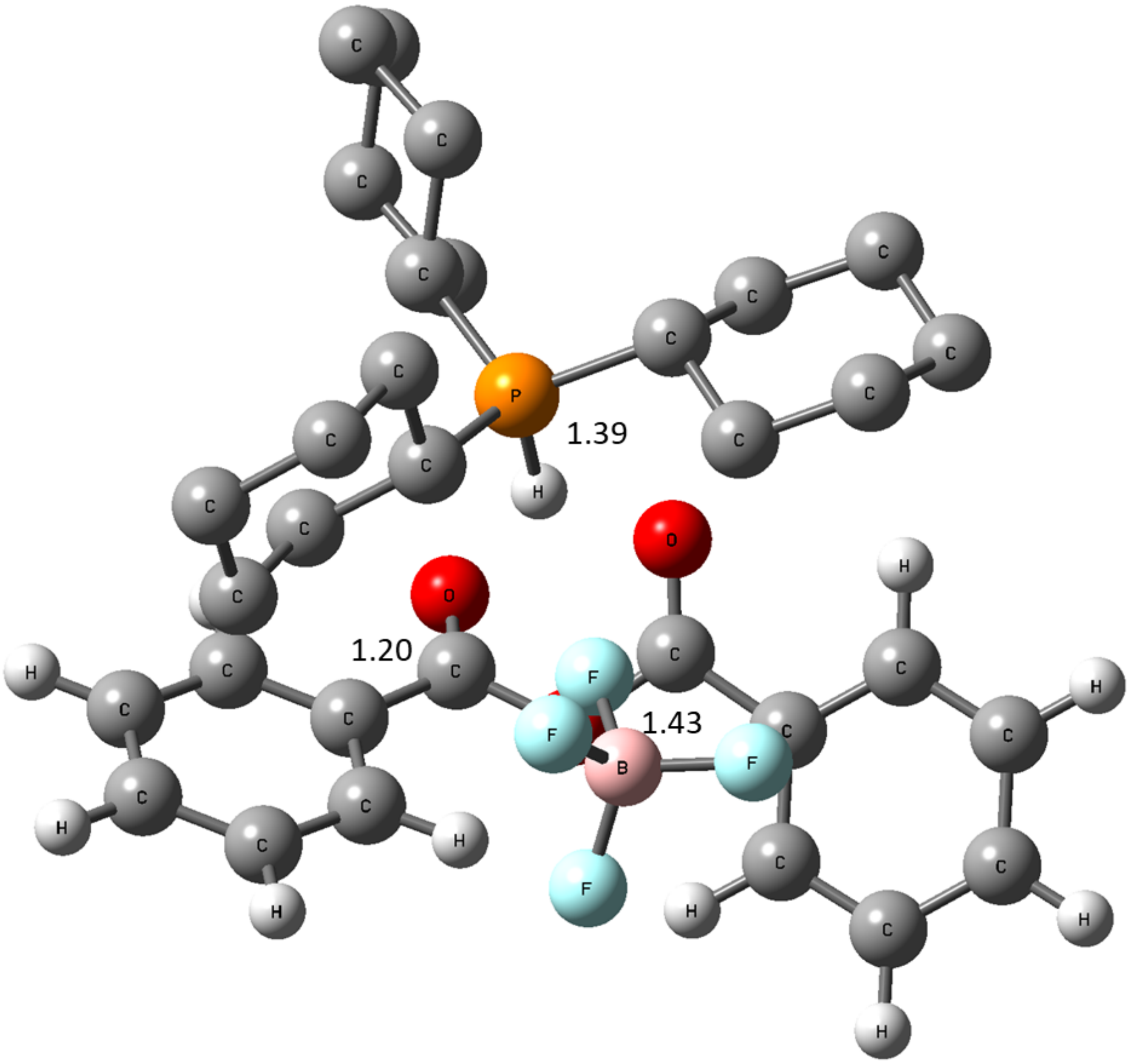

Figure S.6.11 Selected bond lengths (Å) for **Int-s5b**. Some hydrogens have been omitted for clarity.

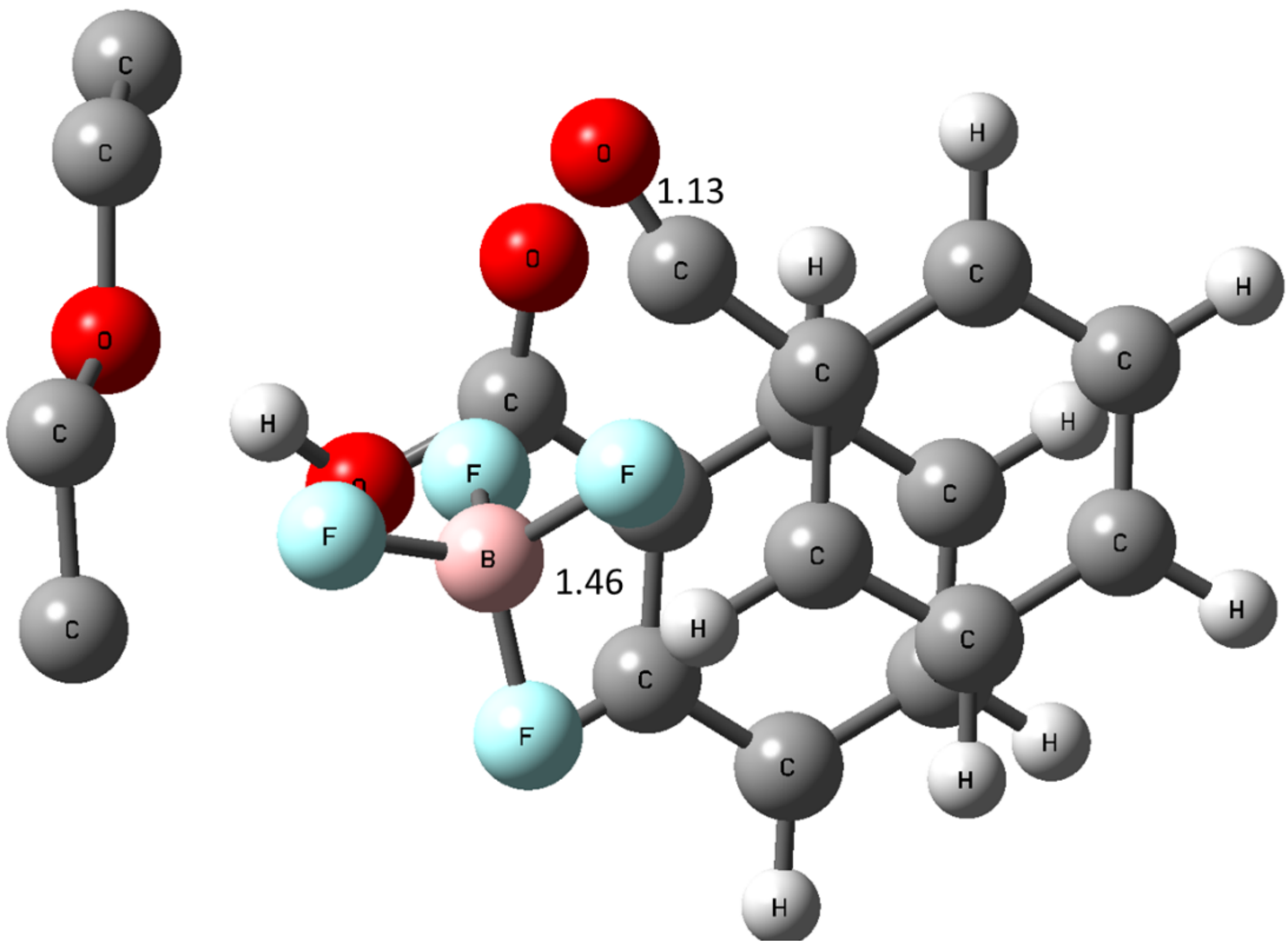

Figure S.6.12 Selected bond lengths (Å) for **Int-s6a**. Some hydrogens have been omitted for clarity.

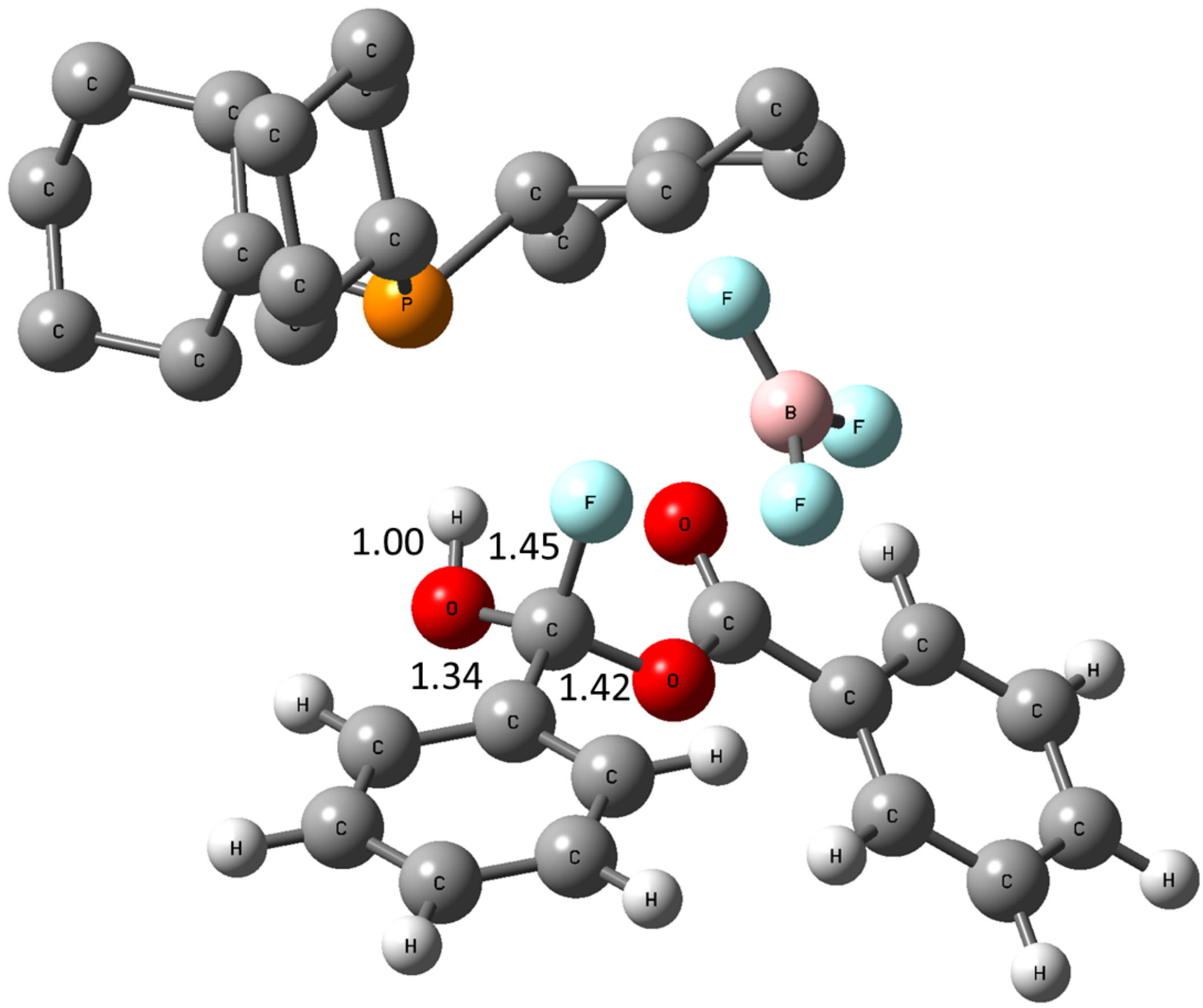

Figure S.6.13 Selected bond lengths (Å) for **Int-s6b**. Some hydrogens have been omitted for clarity.

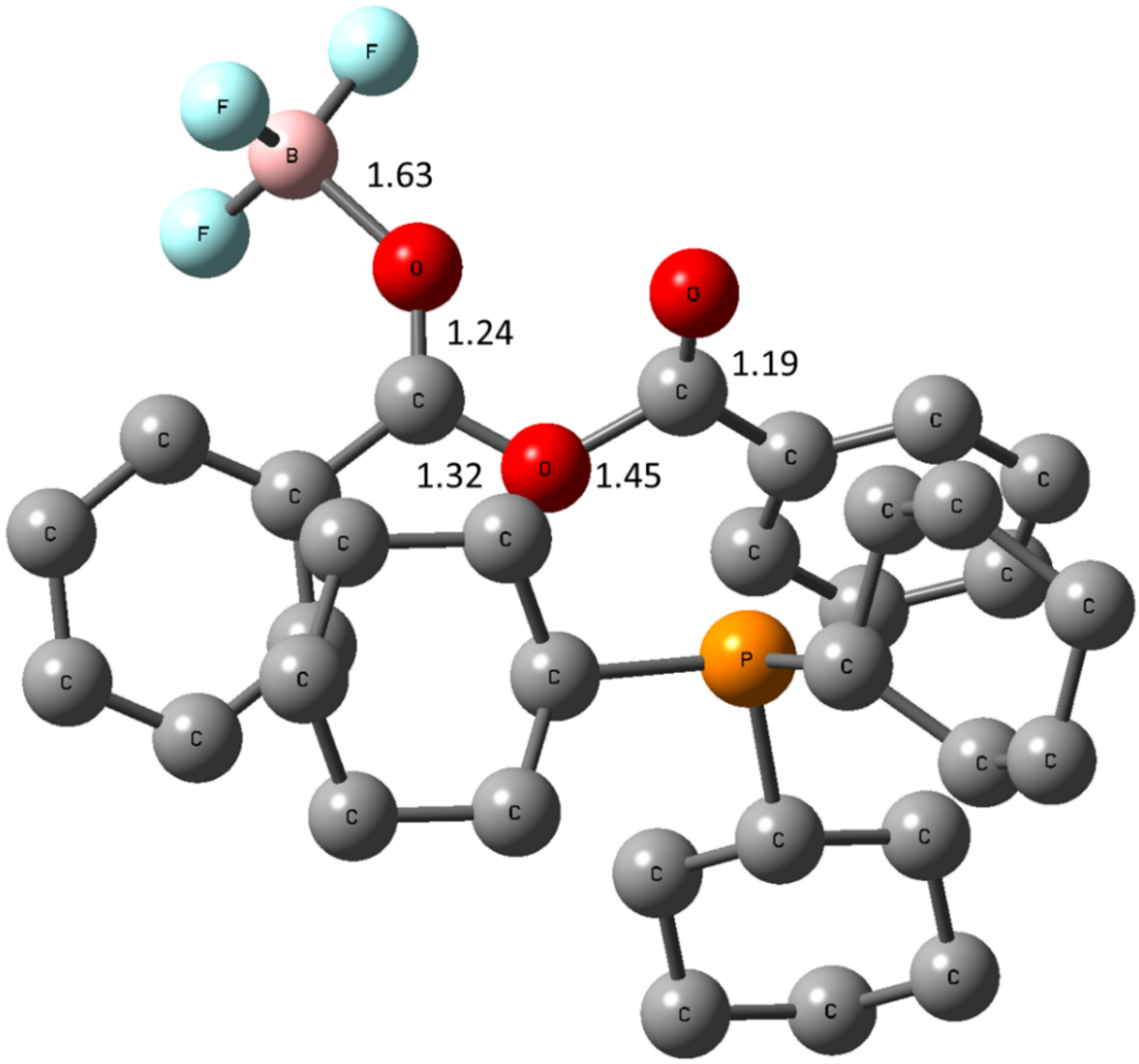

Figure S.6.14 Selected bond lengths (Å) for **Int-s7**. Some hydrogens have been omitted for clarity.

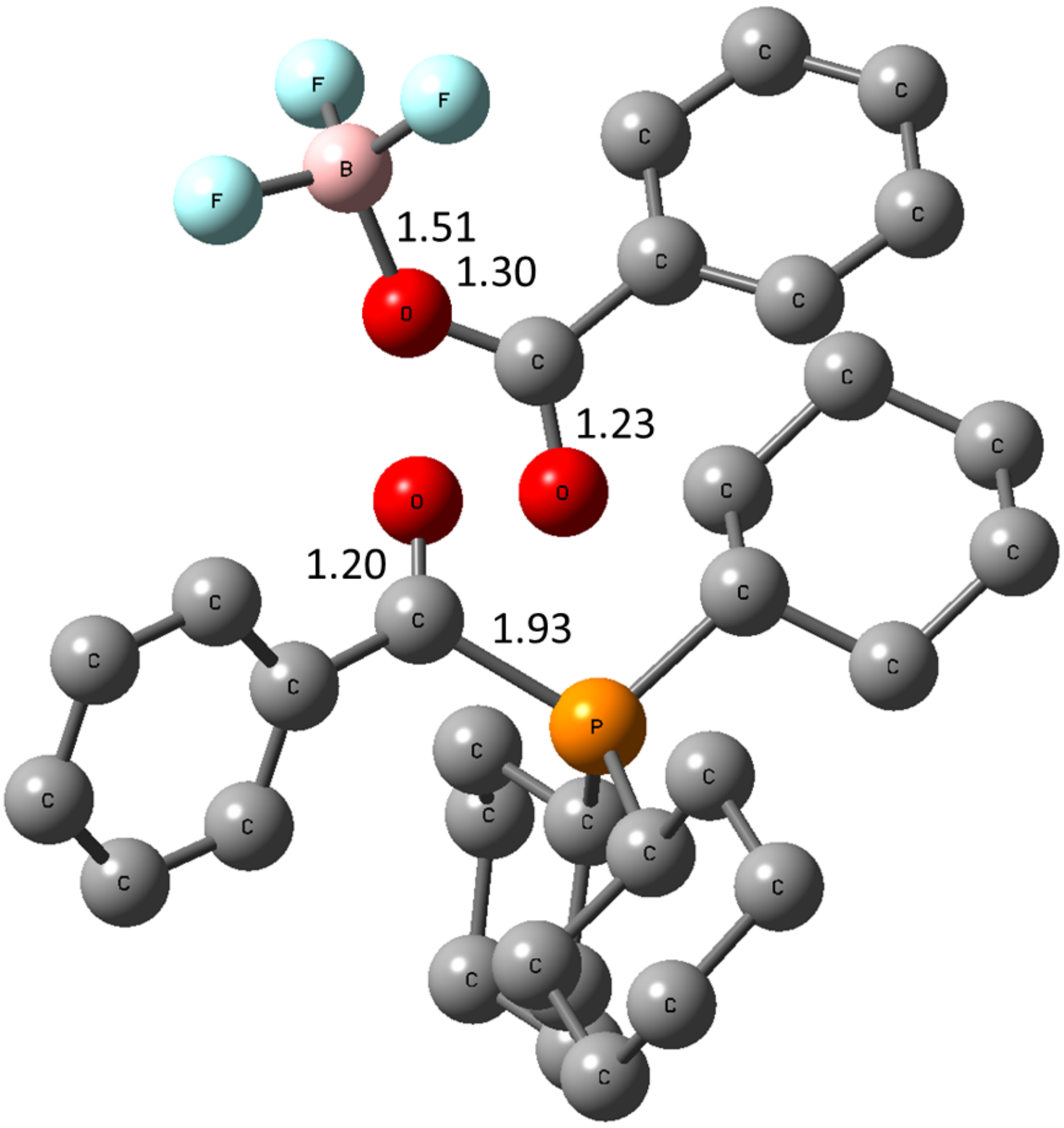

Figure S.6.15 Selected bond lengths (Å) for **Int-s8a**. Some hydrogens have been omitted for clarity.

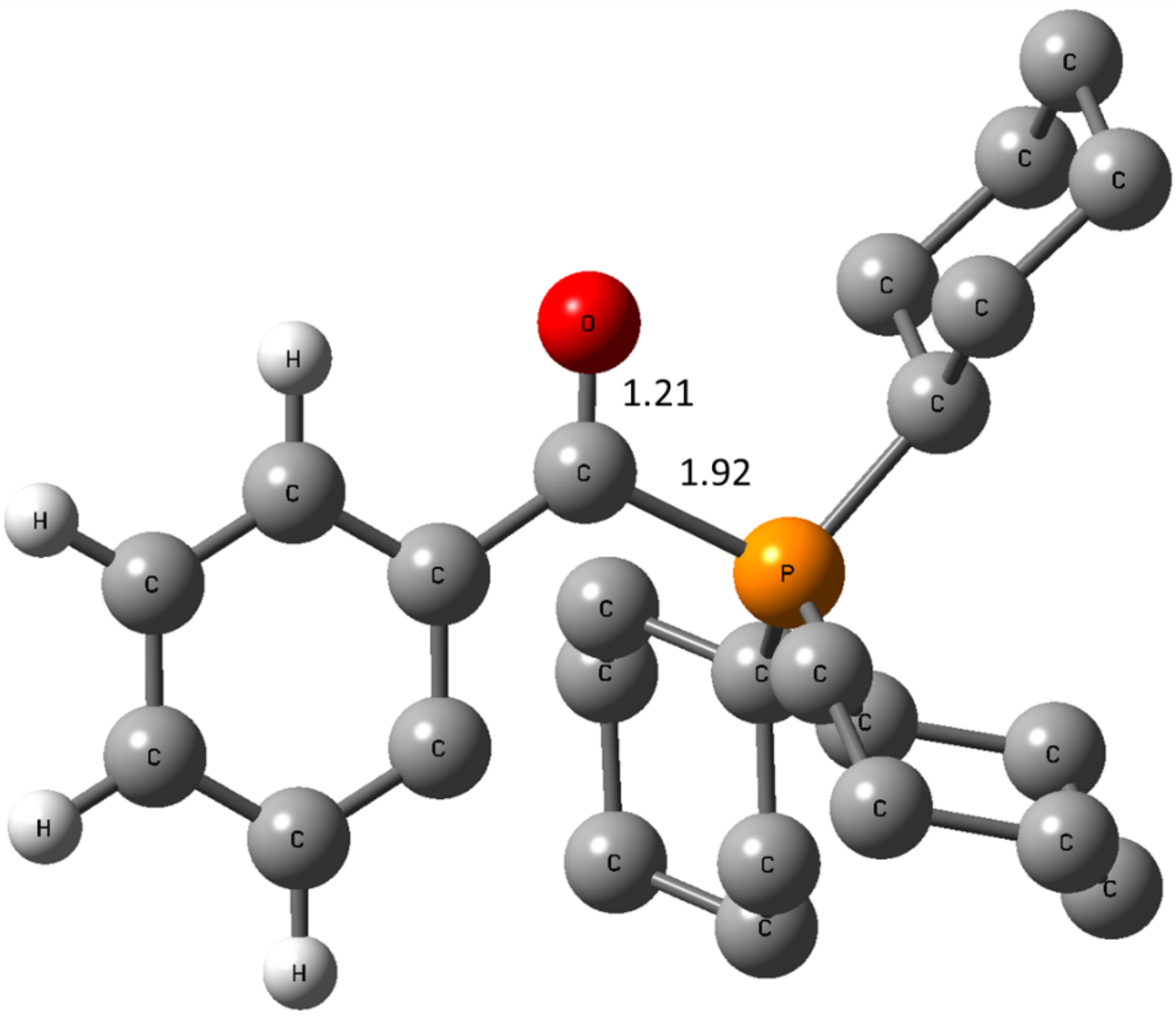

Figure S.6.16 Selected bond lengths (Å) for **Int-s8b**. Some hydrogens have been omitted for clarity.

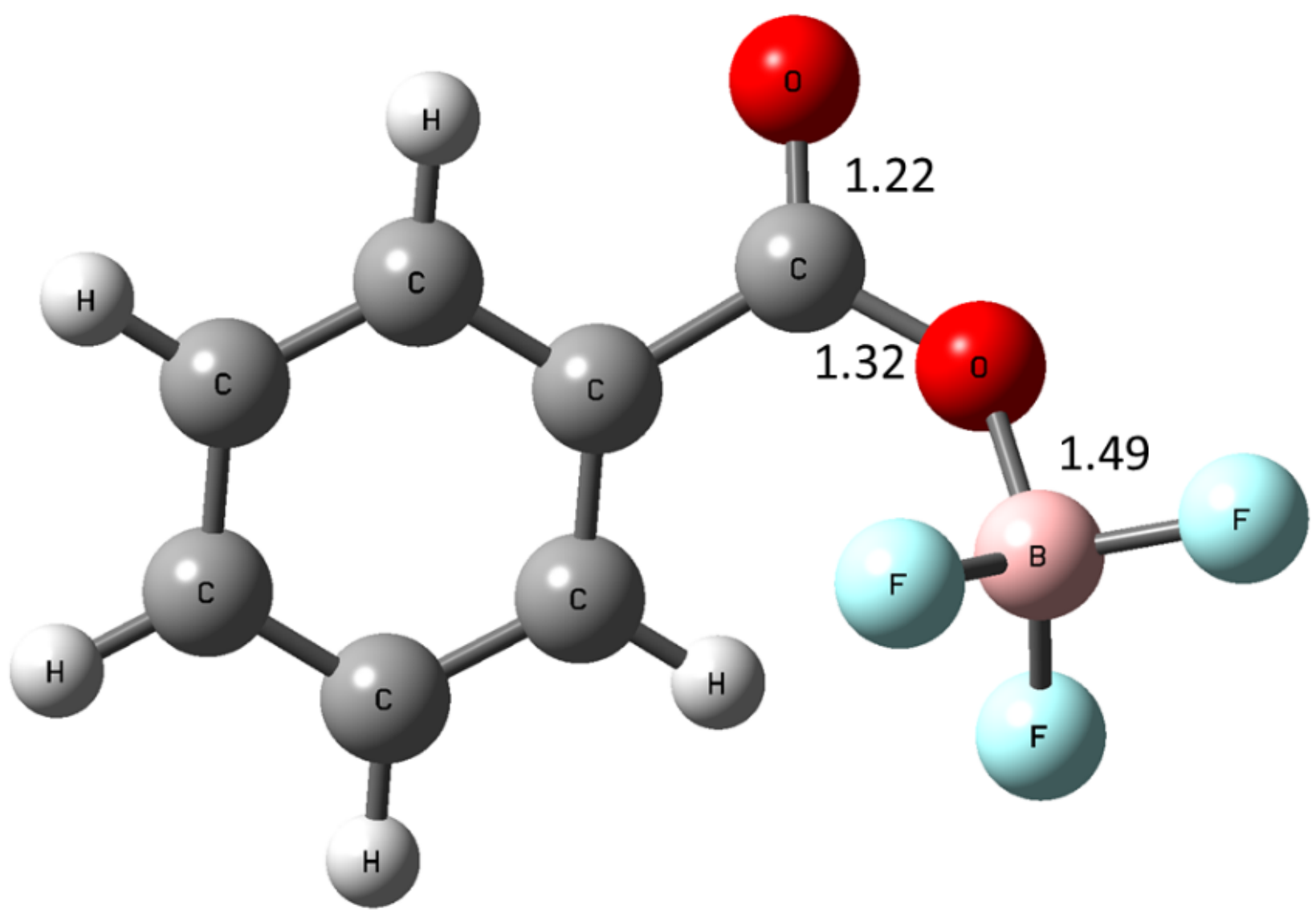

Figure S.6.17 Selected bond lengths (Å) for **Int-s8c**.

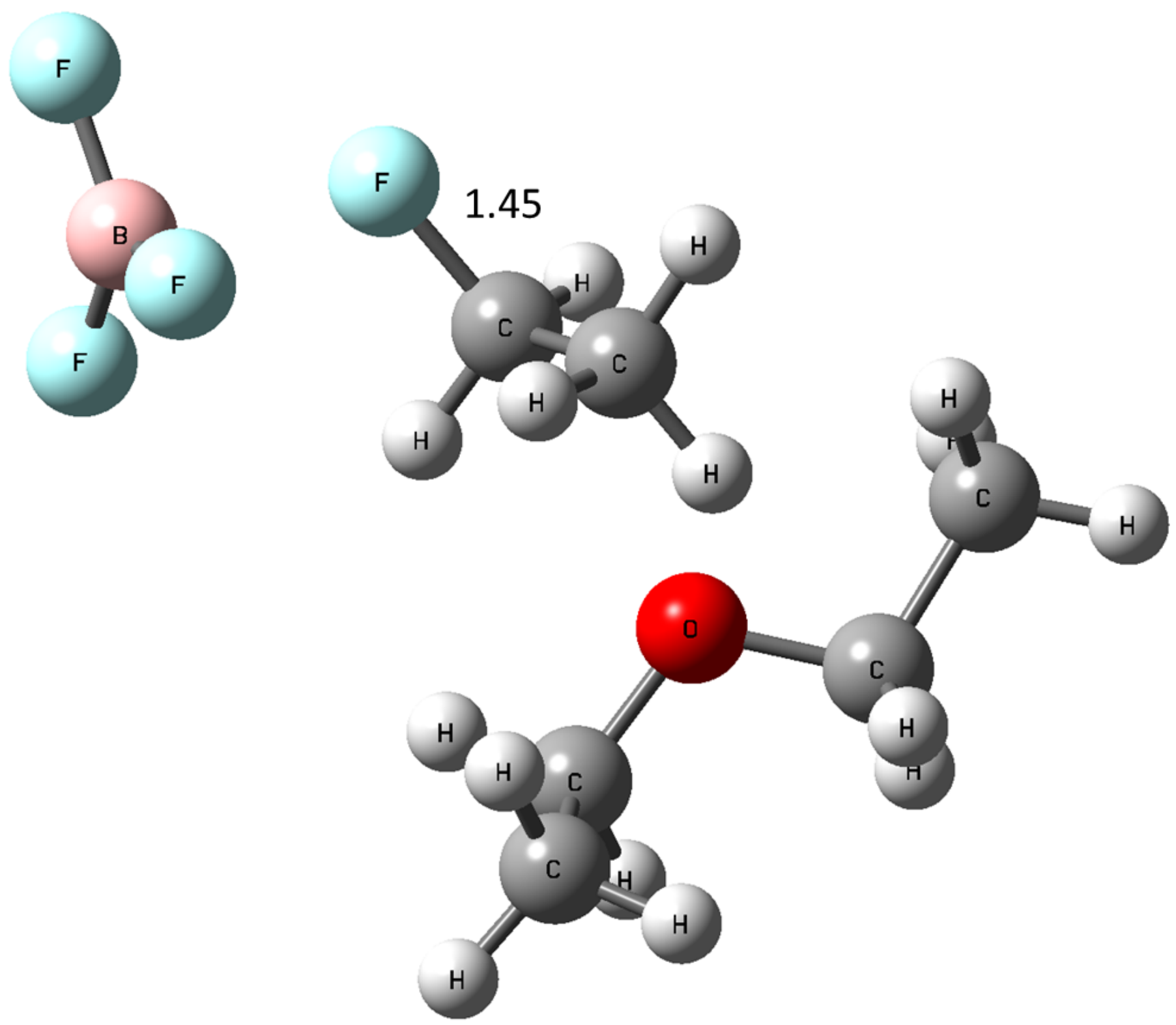

Figure S.6.18 Selected bond lengths (Å) for **Int-s9a**.

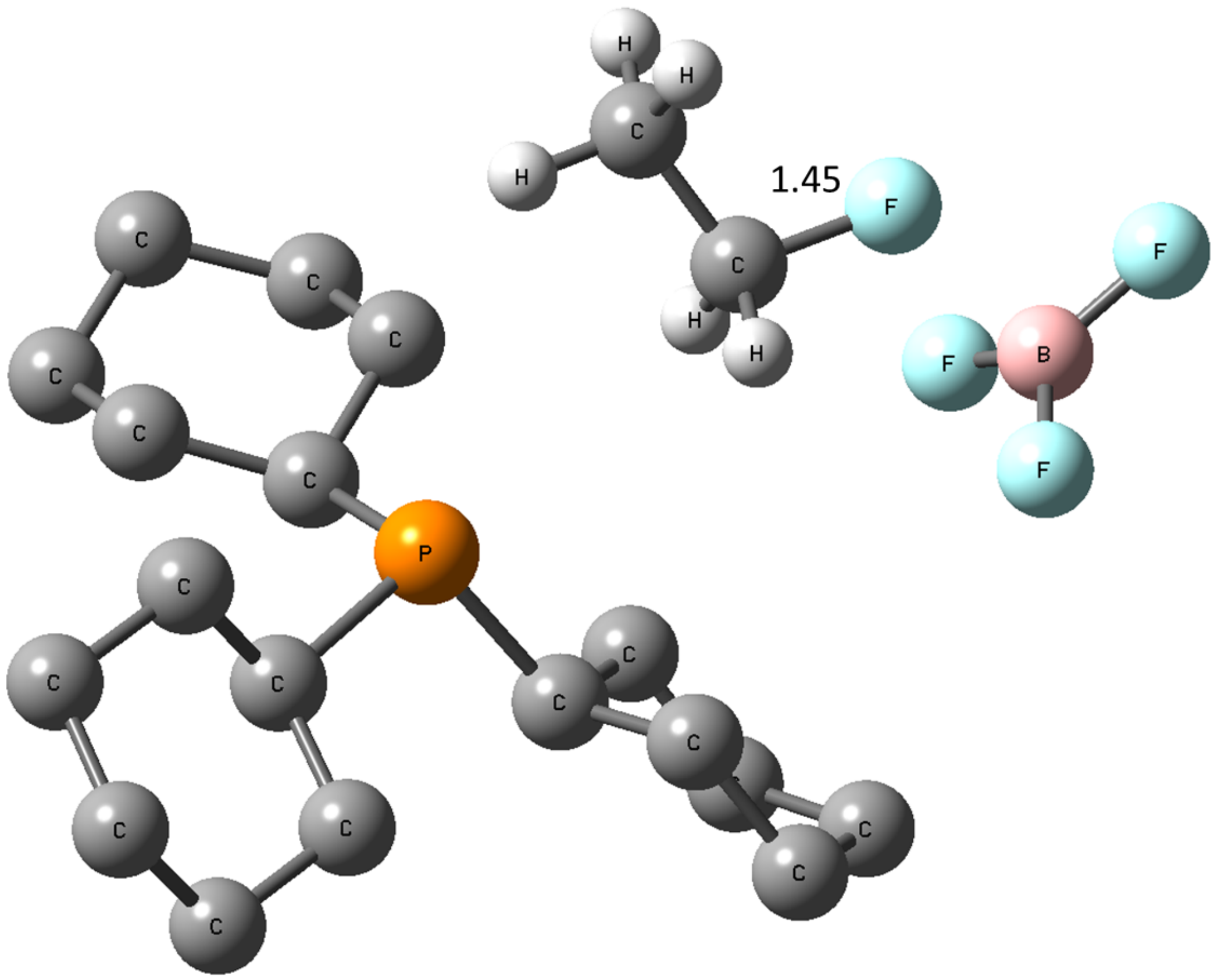

Figure S.6.19 Selected bond lengths (Å) for **Int-s9b**. Some hydrogens have been omitted for clarity.

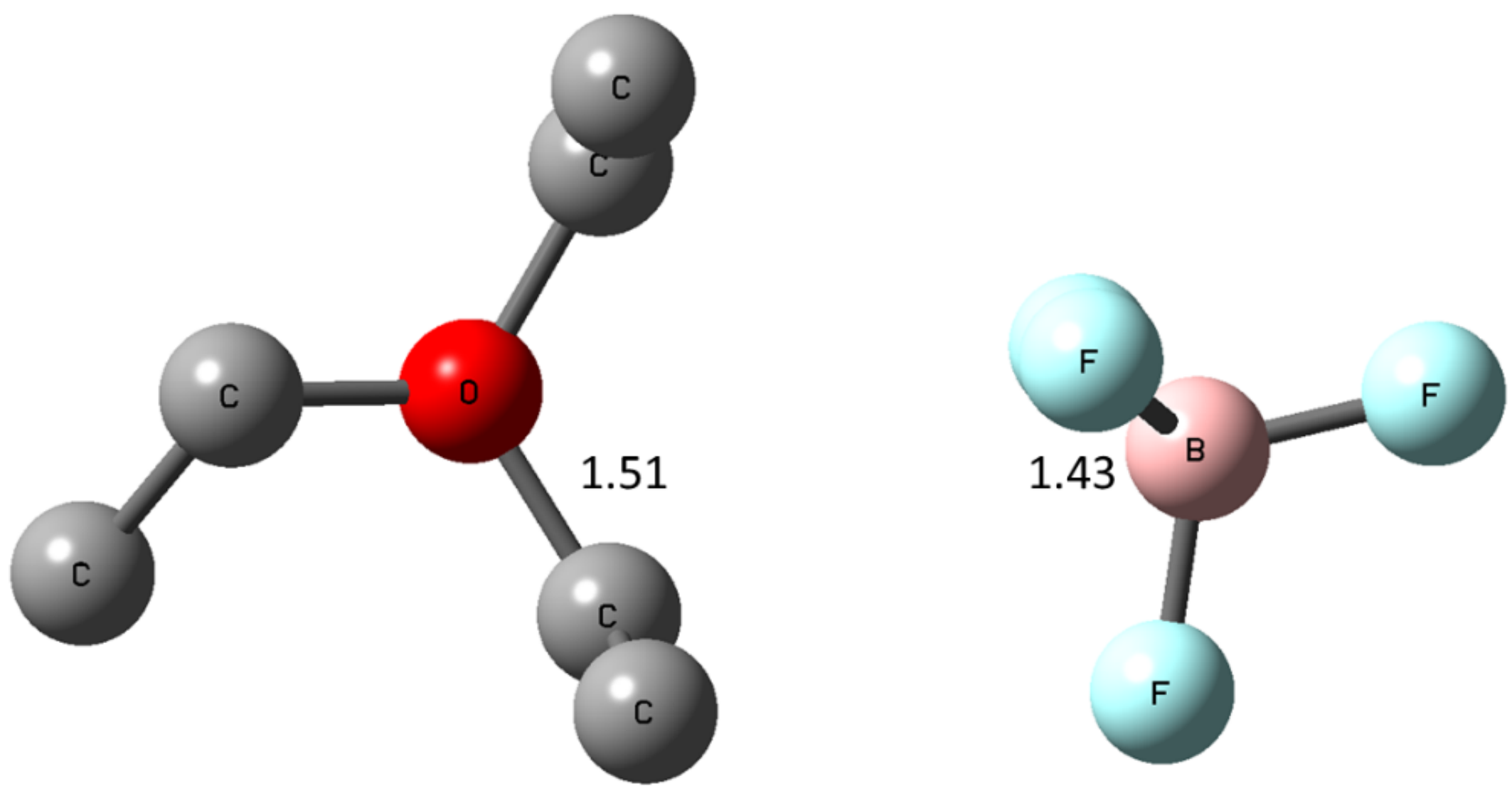

Figure S.6.20 Selected bond lengths (Å) for **Int-s10a**. Some hydrogens have been omitted for clarity.

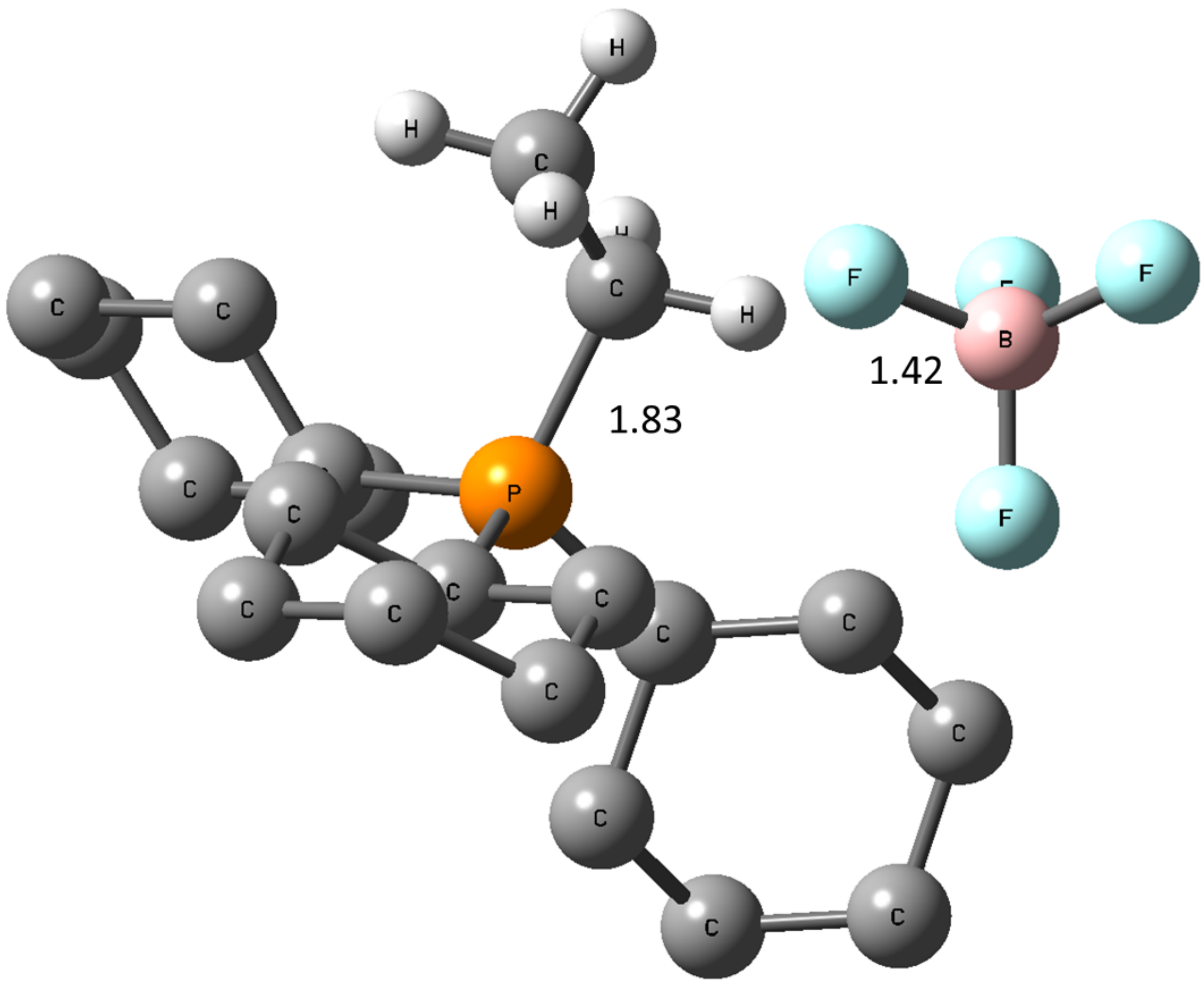

Figure S.6.21 Selected bond lengths (Å) for **Int-s10b**. Some hydrogens have been omitted for clarity.

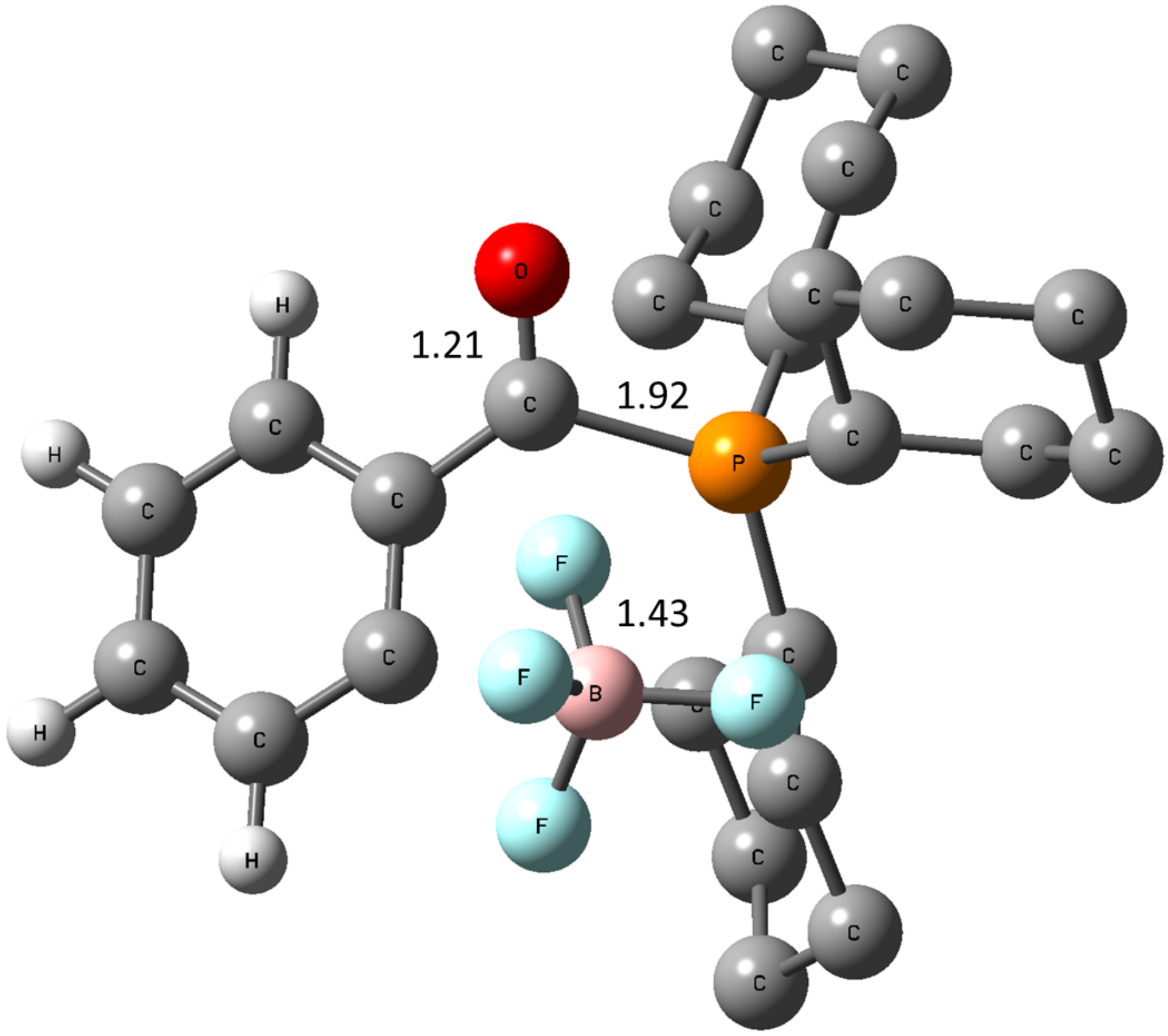

Figure S.6.22 Selected bond lengths (Å) for **Int-s11**. Some hydrogens have been omitted for clarity.

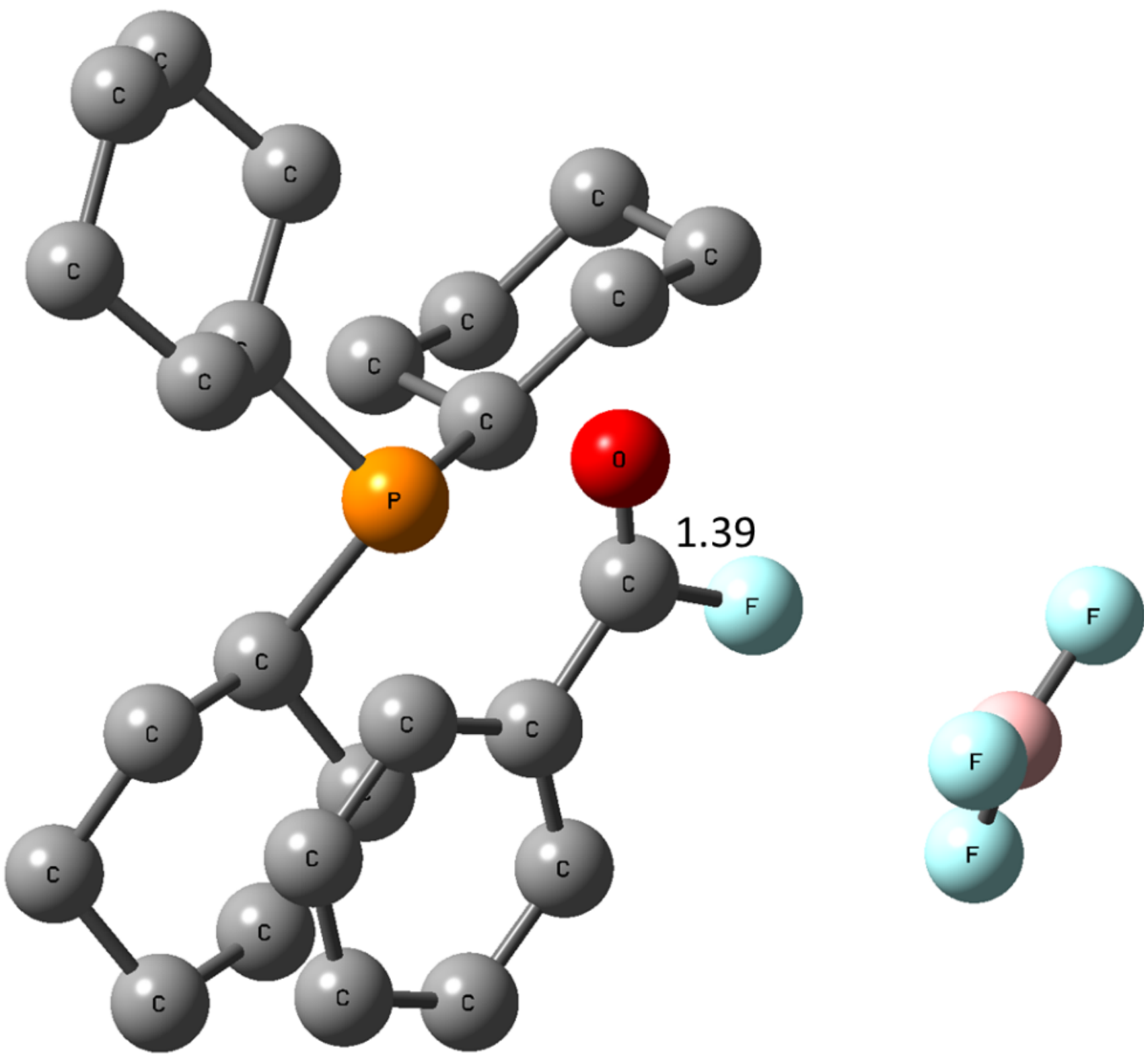

Figure S.6.23 Selected bond lengths (Å) for **Int-s12**. Some hydrogens have been omitted for clarity.

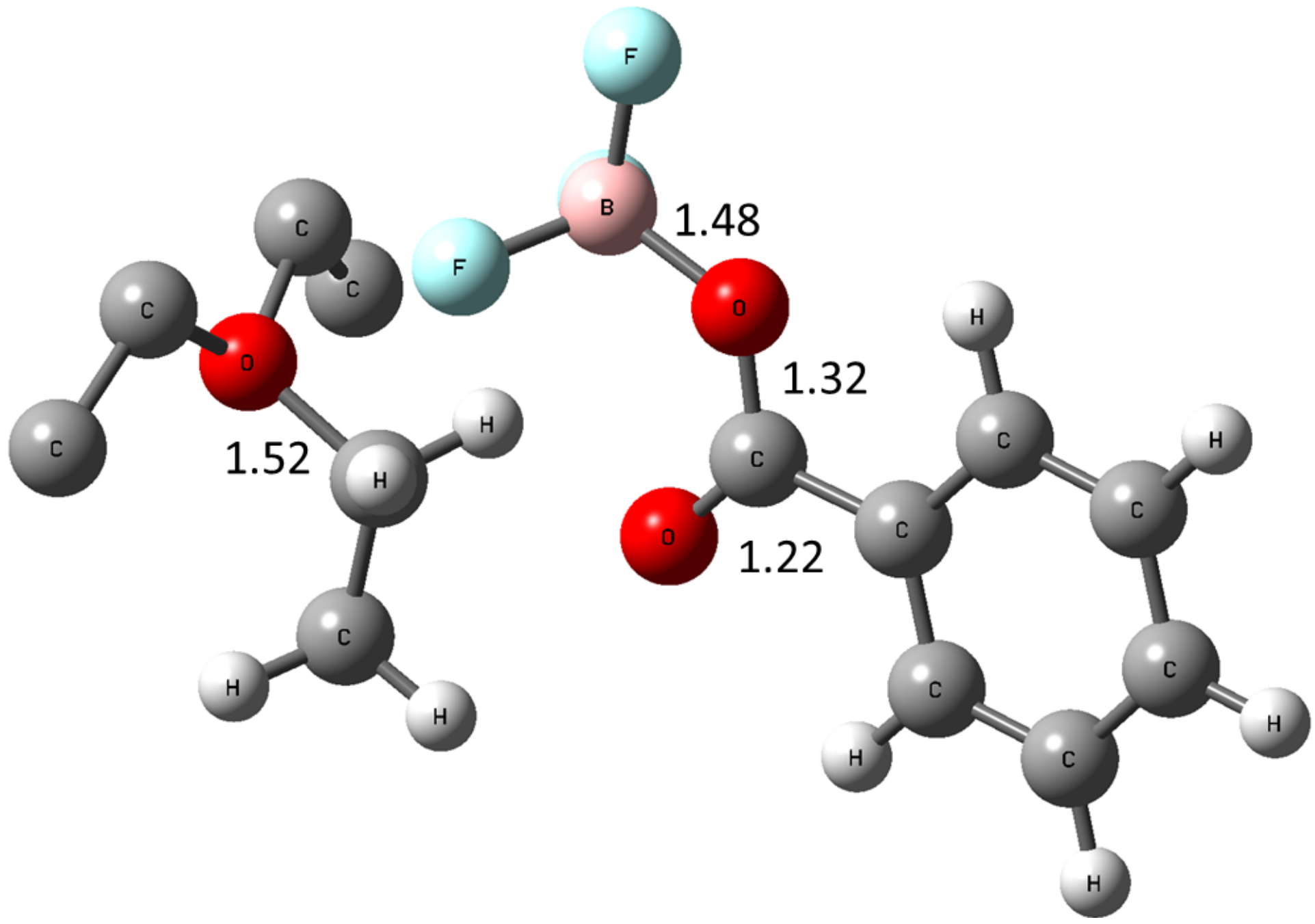

Figure S.6.24 Selected bond lengths (Å) for **Int-s13a**. Some hydrogens have been omitted for clarity.

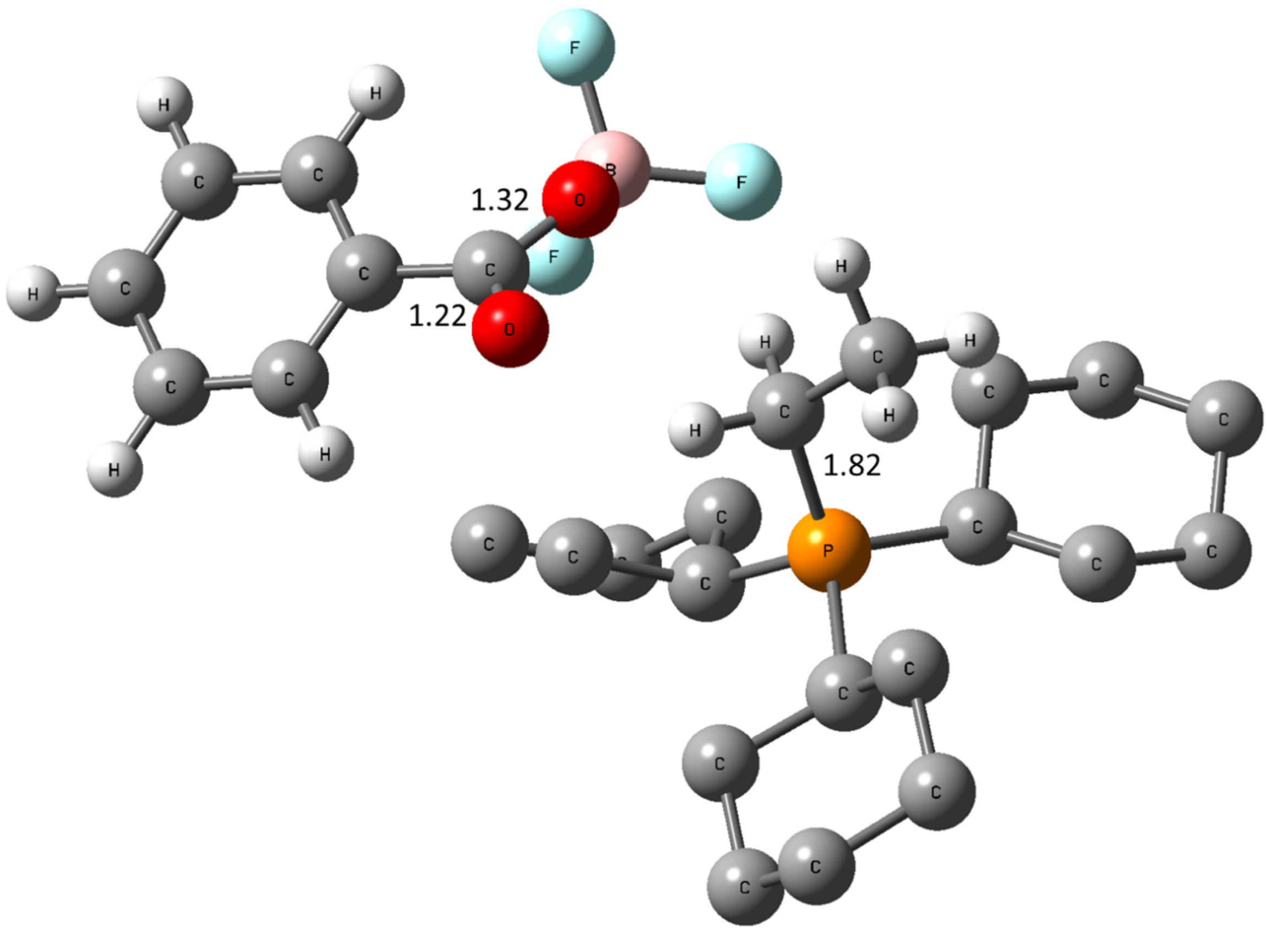

Figure S.6.25 Selected bond lengths (Å) for **Int-s13b**. Some hydrogens have been omitted for clarity.

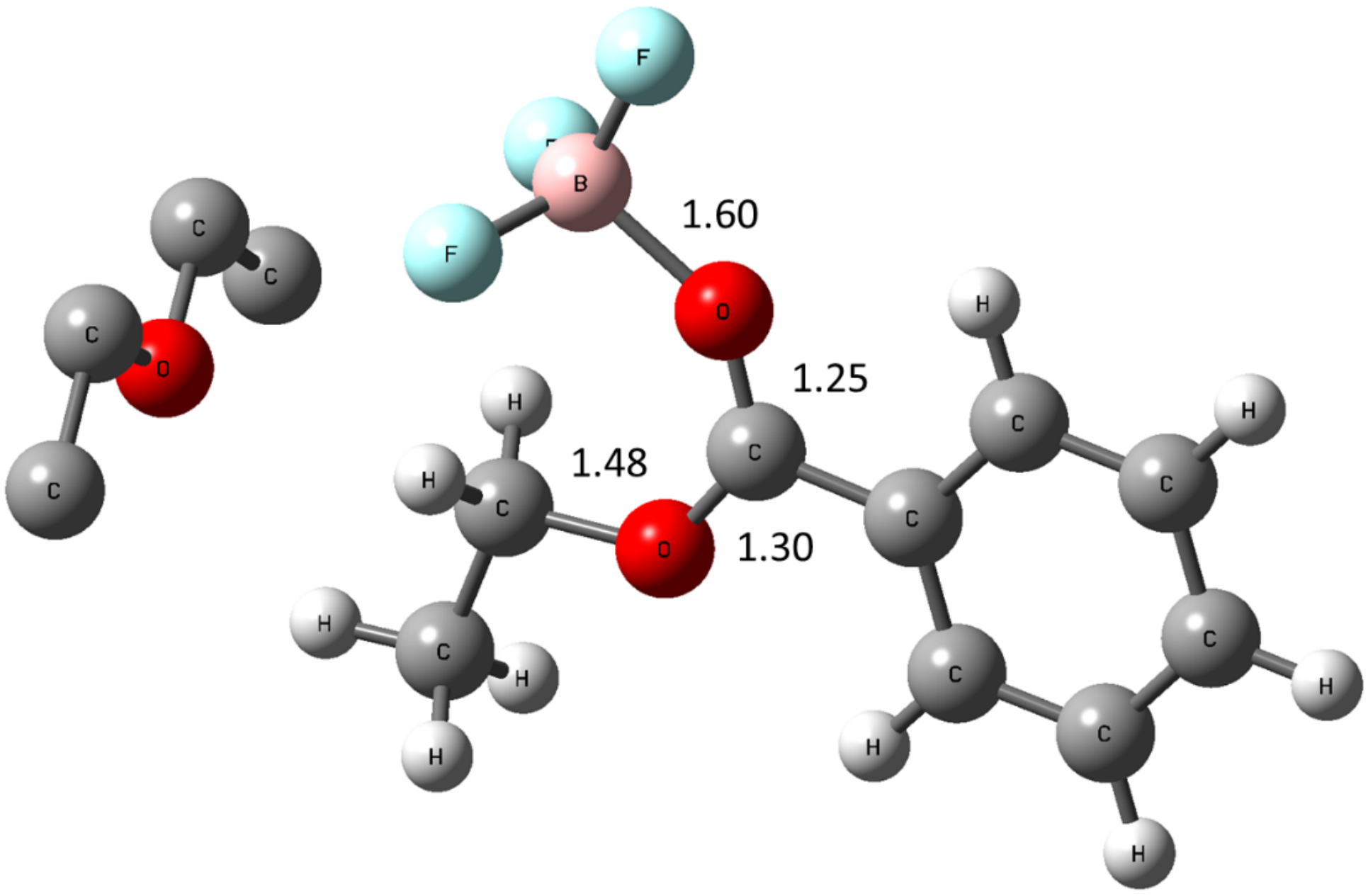

Figure S. 6.26 Selected bond lengths (Å) for **Int-s14a**. Some hydrogens have been omitted for clarity.

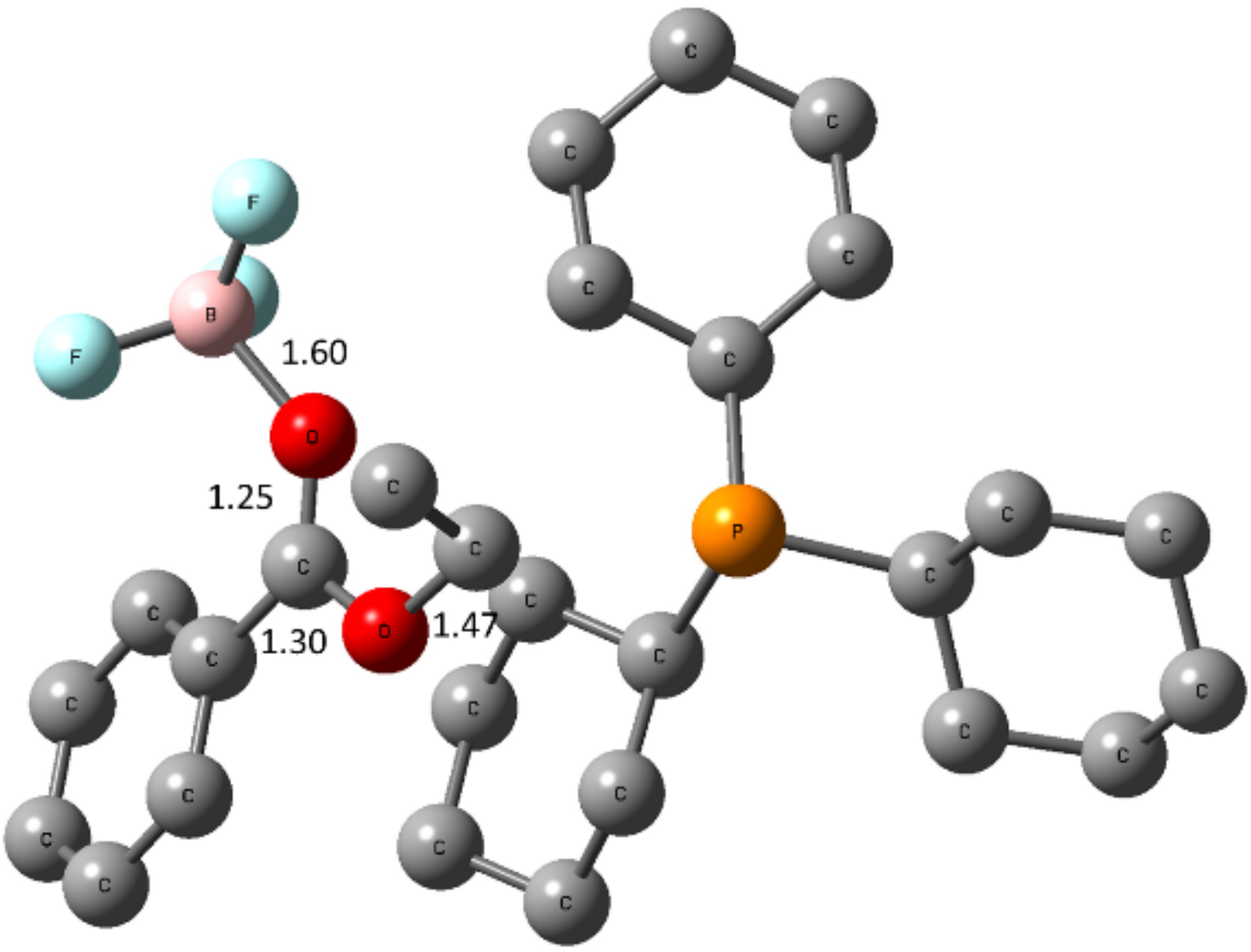

Figure S. 6.27 Selected bond lengths (Å) for **Int-s14b**. Some hydrogens have been omitted for clarity.

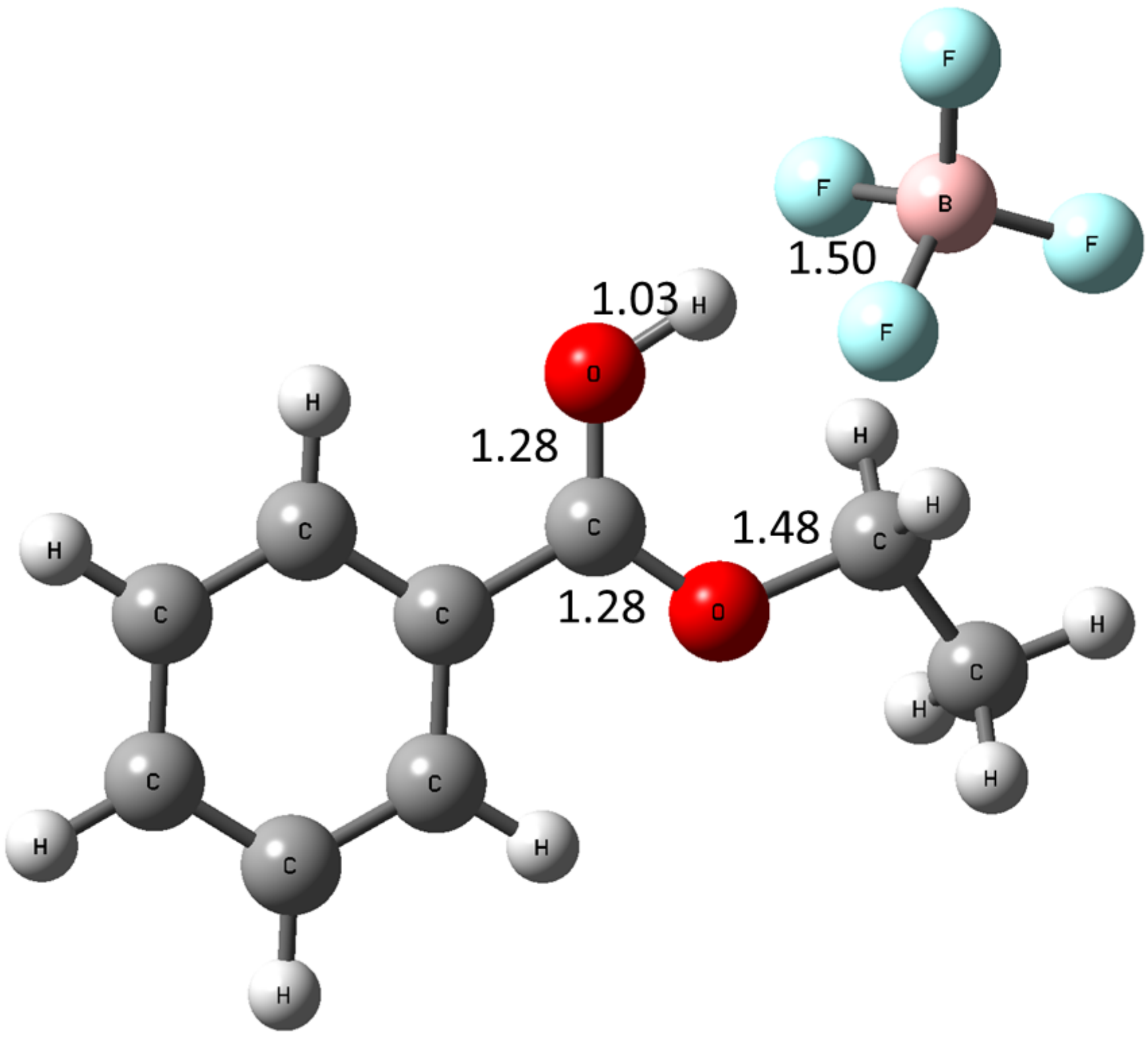

Figure S.6.28 Selected bond lengths (Å) for **Int-s15**. Some hydrogens have been omitted for clarity.

6.2.3 Key geometrical parameters of transition states

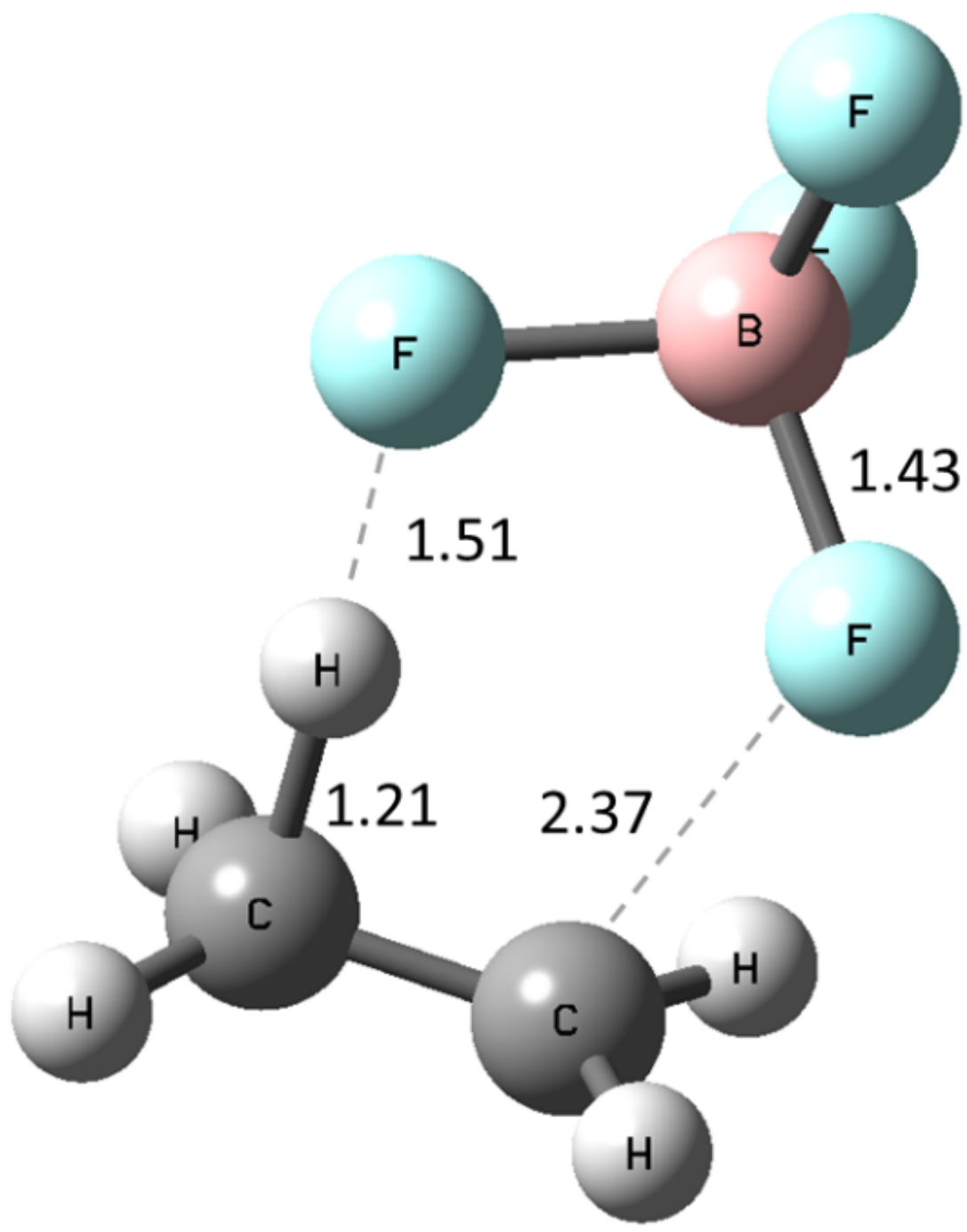

Figure S. 6.29 Selected bond lengths (Å) for **TS-s1a = TS-1**.

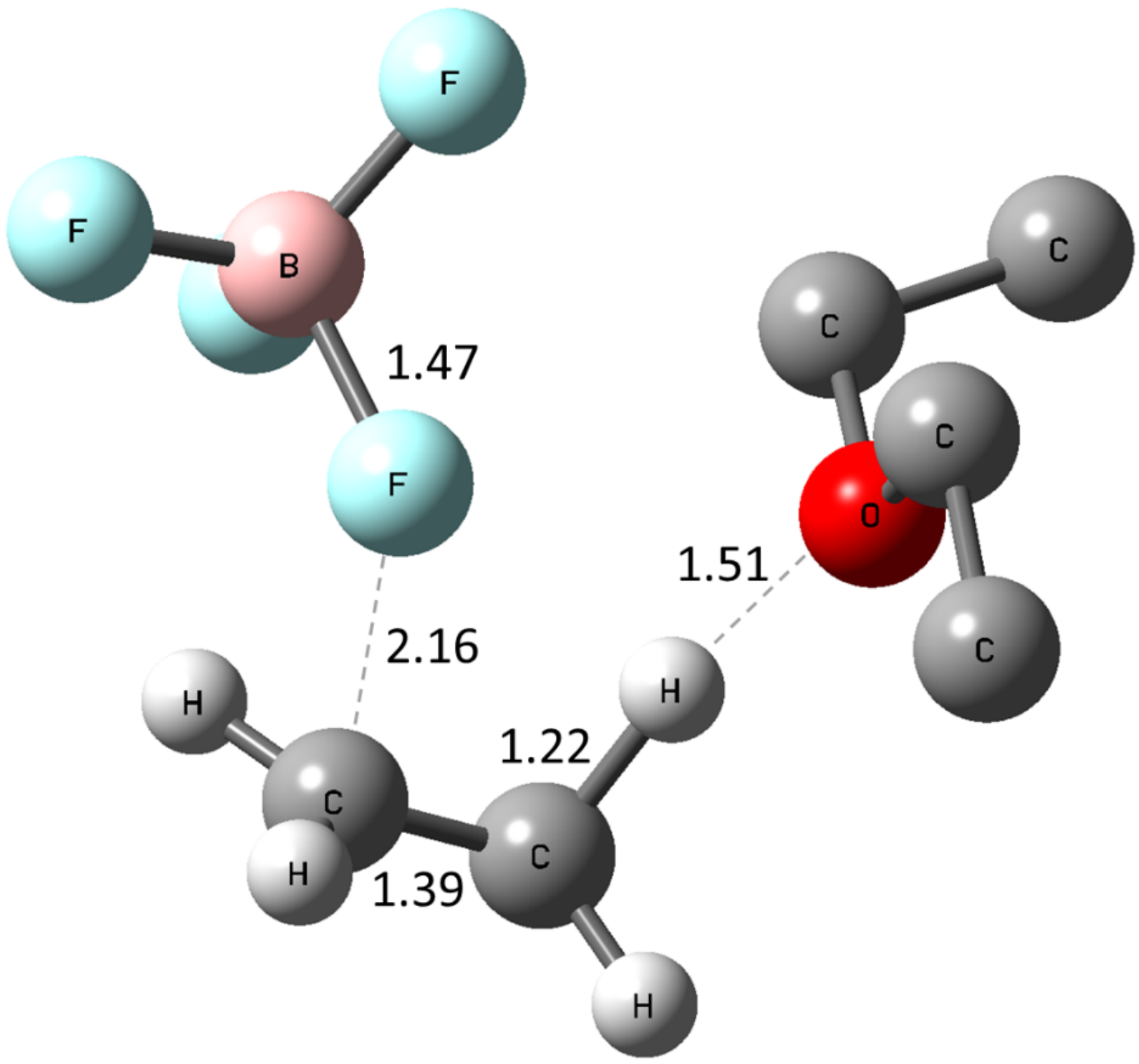

Figure S.6.30 Selected bond lengths (Å) for **TS-s1b**. Some hydrogens have been omitted for clarity.

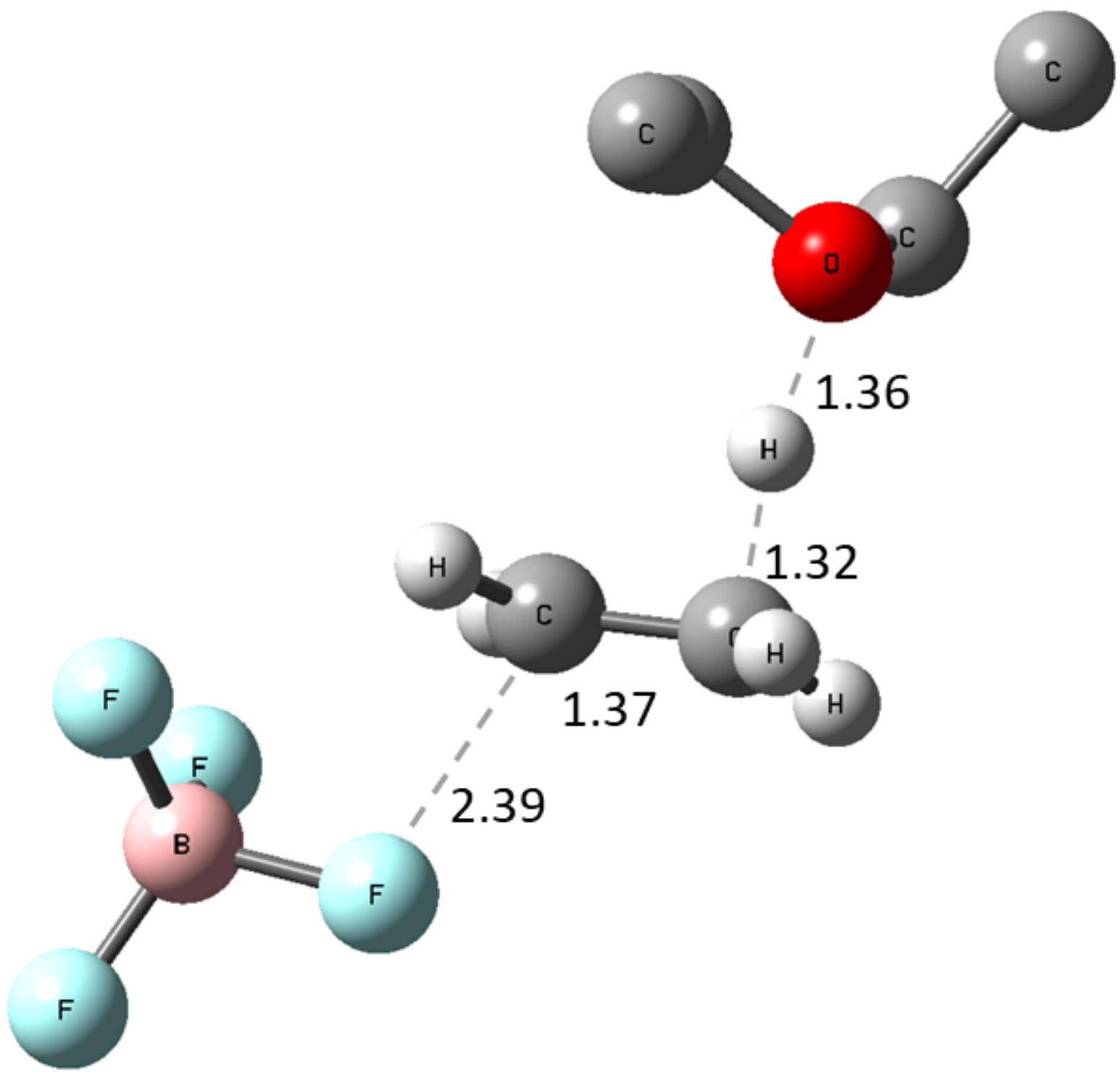

Figure S.6.31 Selected bond lengths (Å) for **TS-s1c**. Some hydrogens have been omitted for clarity.

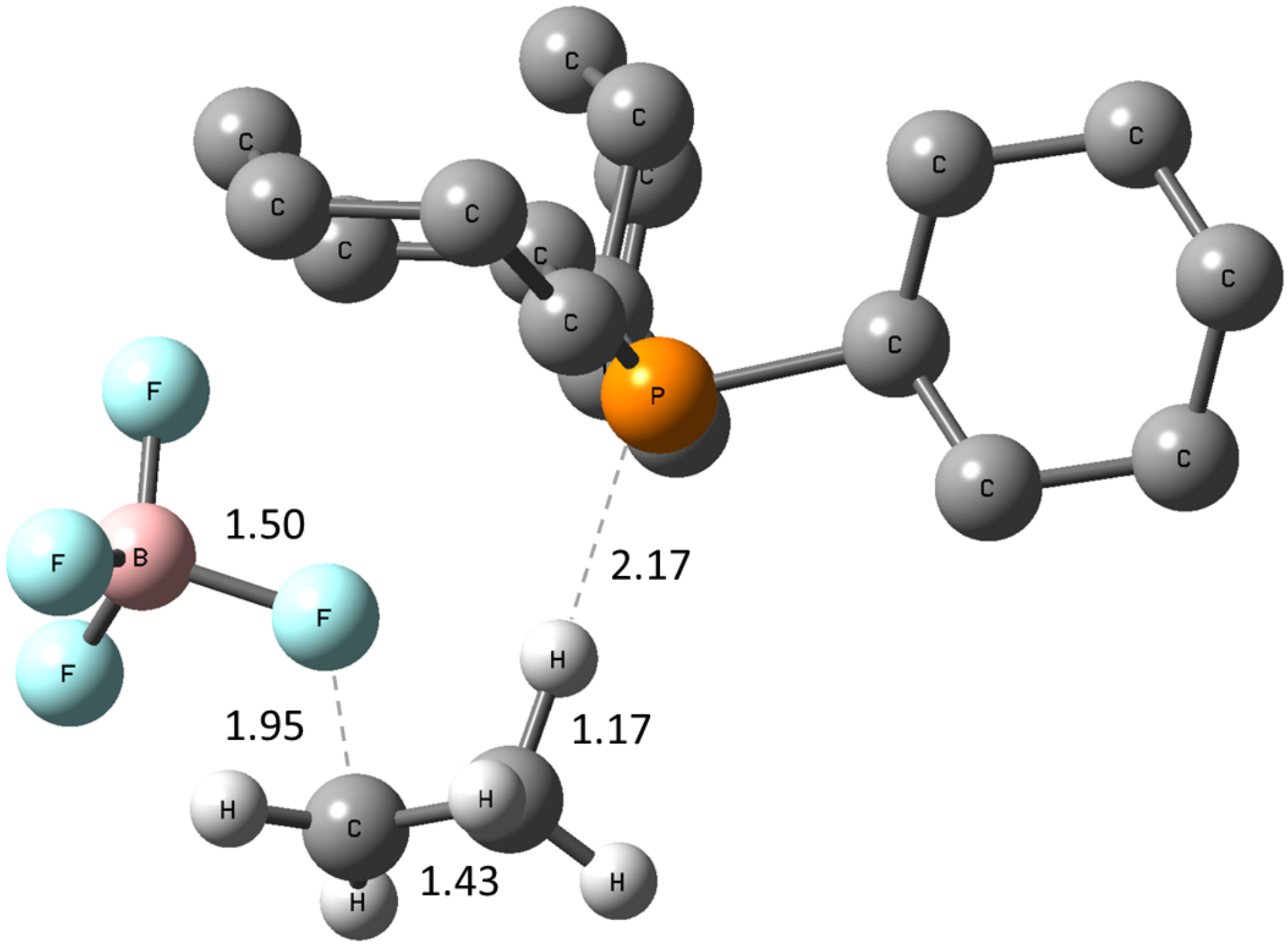

Figure S.6.32 Selected bond lengths (Å) for **TS-s1d**. Some hydrogens have been omitted for clarity.

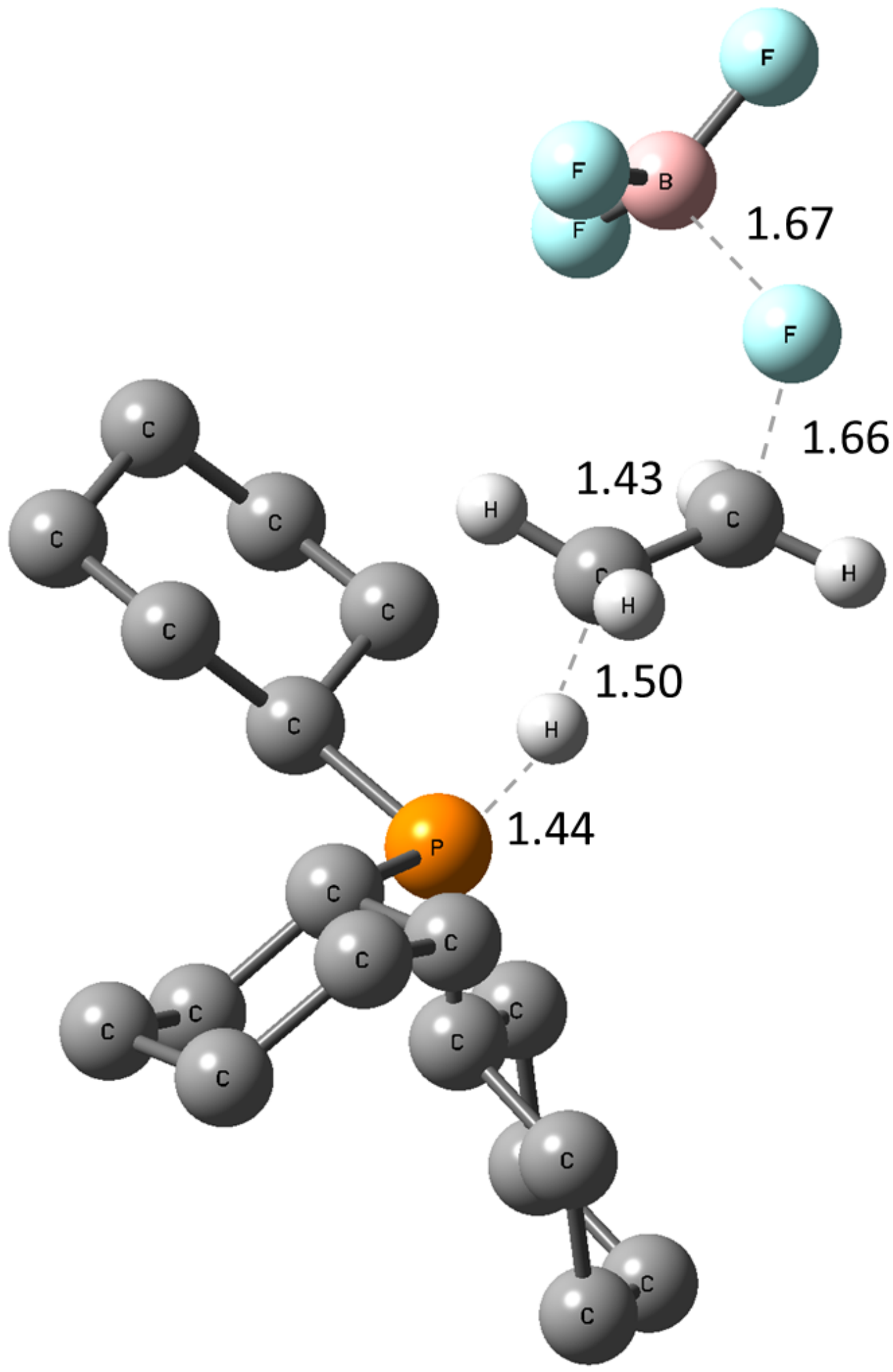

Figure S.6.33 Selected bond lengths (Å) for **TS-s1e**. Some hydrogens have been omitted for clarity.

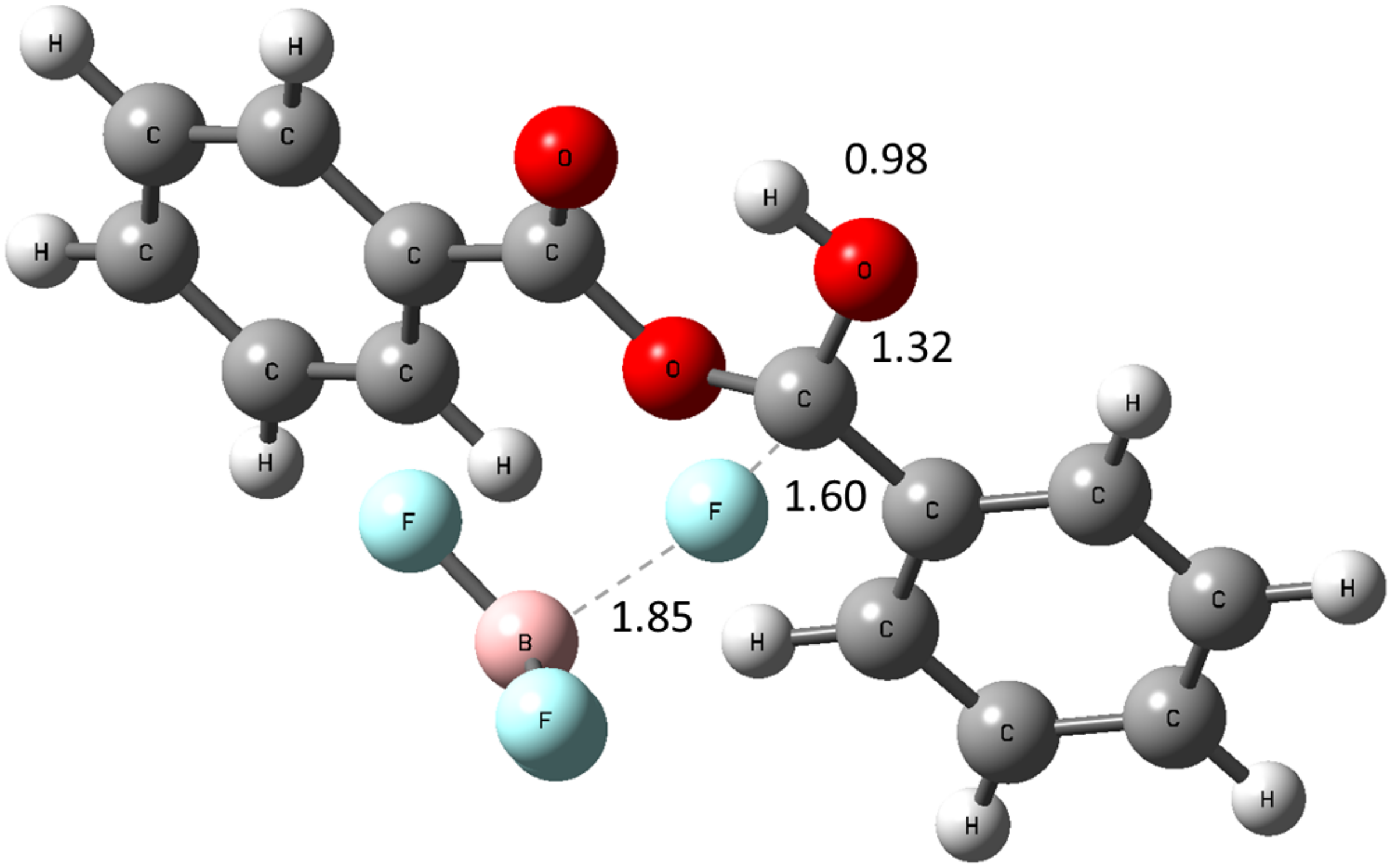

Figure S.6.34 Selected bond lengths (Å) for **TS-s2** = **TS-2**.

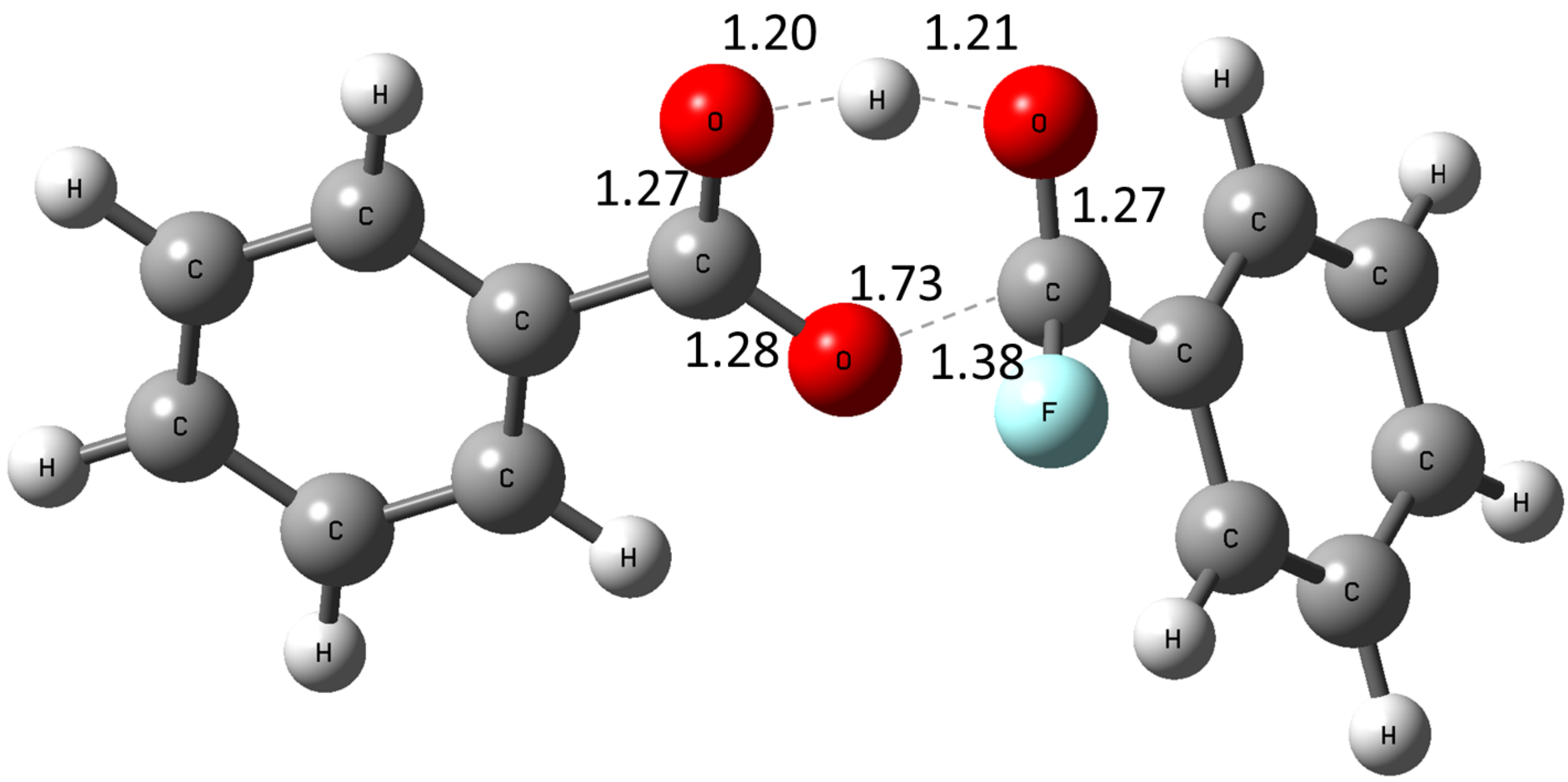

Figure S.6.35 Selected bond lengths (Å) for **TS-s3** = **TS-3**.

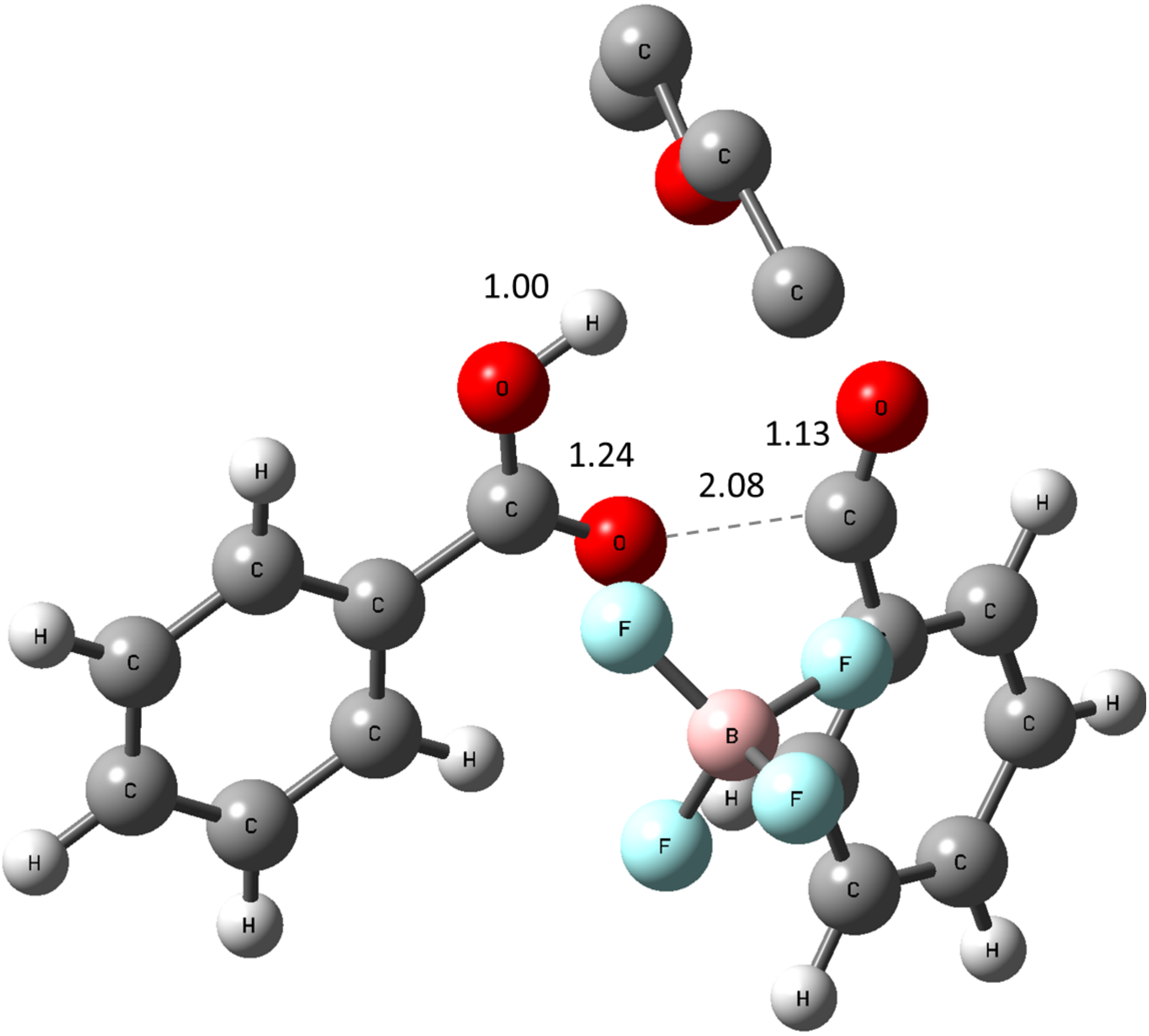

Figure S.6.36 Selected bond lengths (Å) for **TS-s4a**. Some hydrogens have been omitted for clarity.

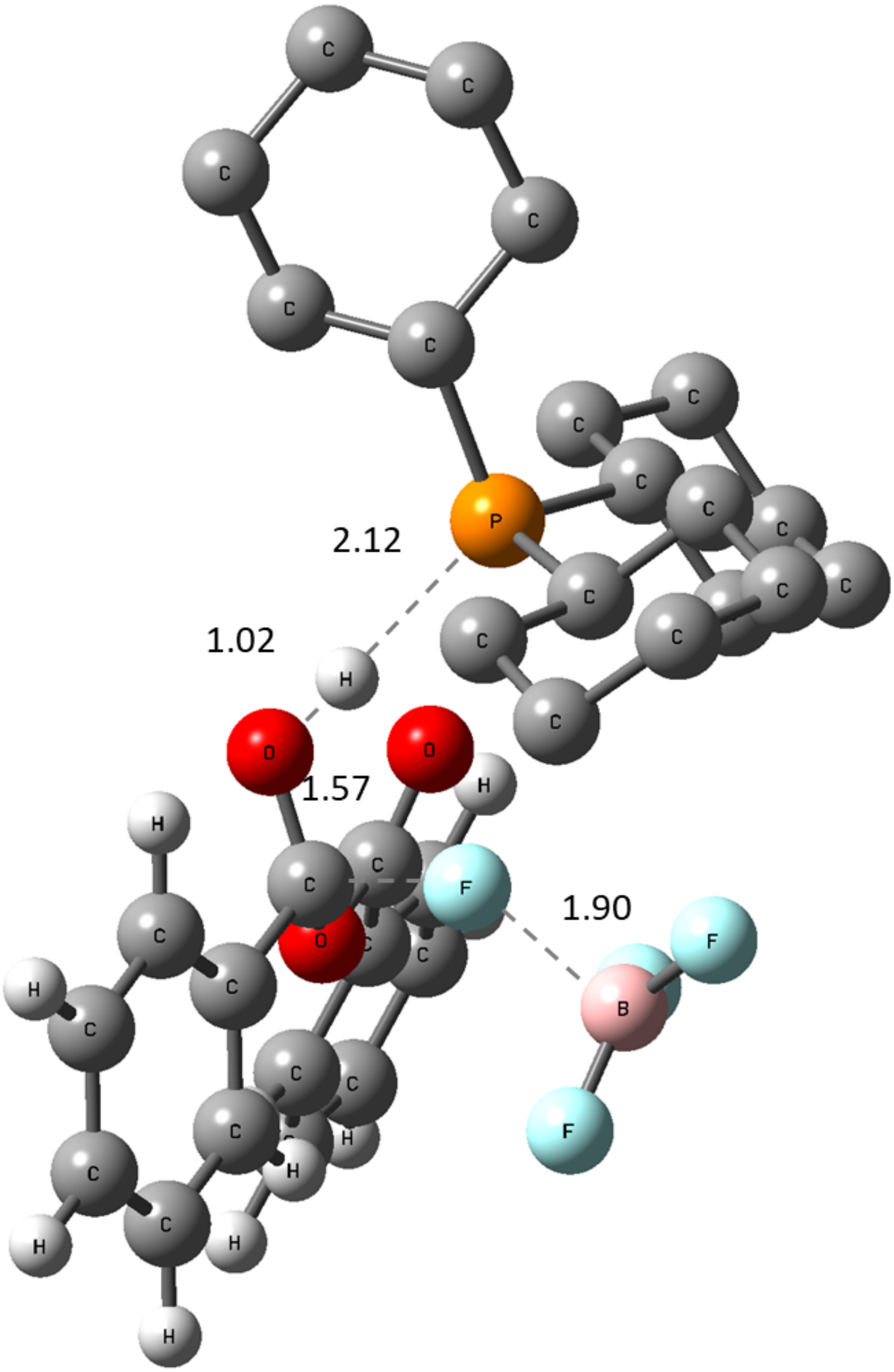

Figure S.6.37 Selected bond lengths (Å) for **TS-s4b**. Some hydrogens have been omitted for clarity.

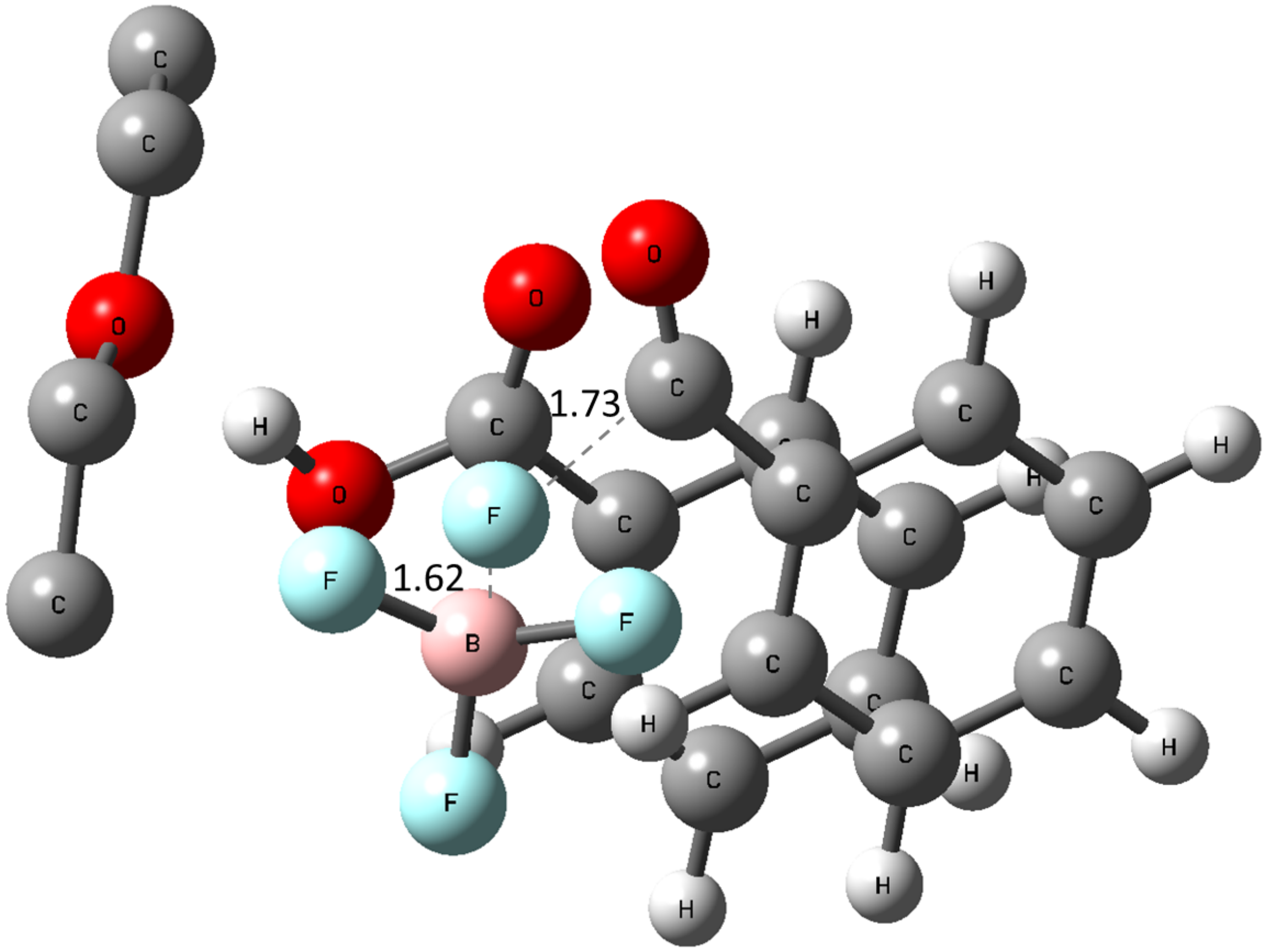

Figure S.6.38 Selected bong lengths (Å) for **TS-s5a**. Some hydrogens have been omitted for clarity.

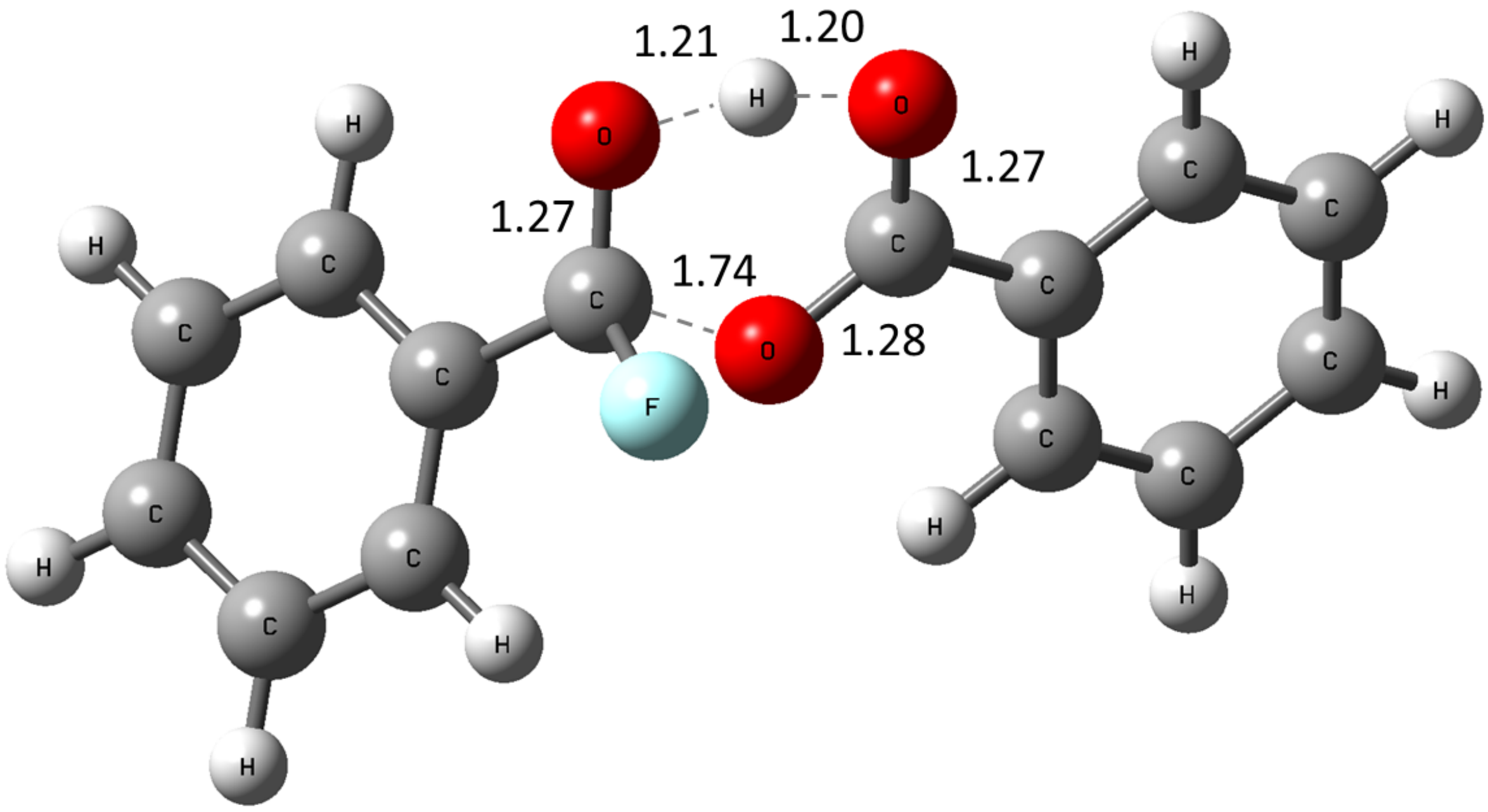

Figure S.6.39 Selected bong lengths (Å) for **TS-s5b**. Some hydrogens have been omitted for clarity.

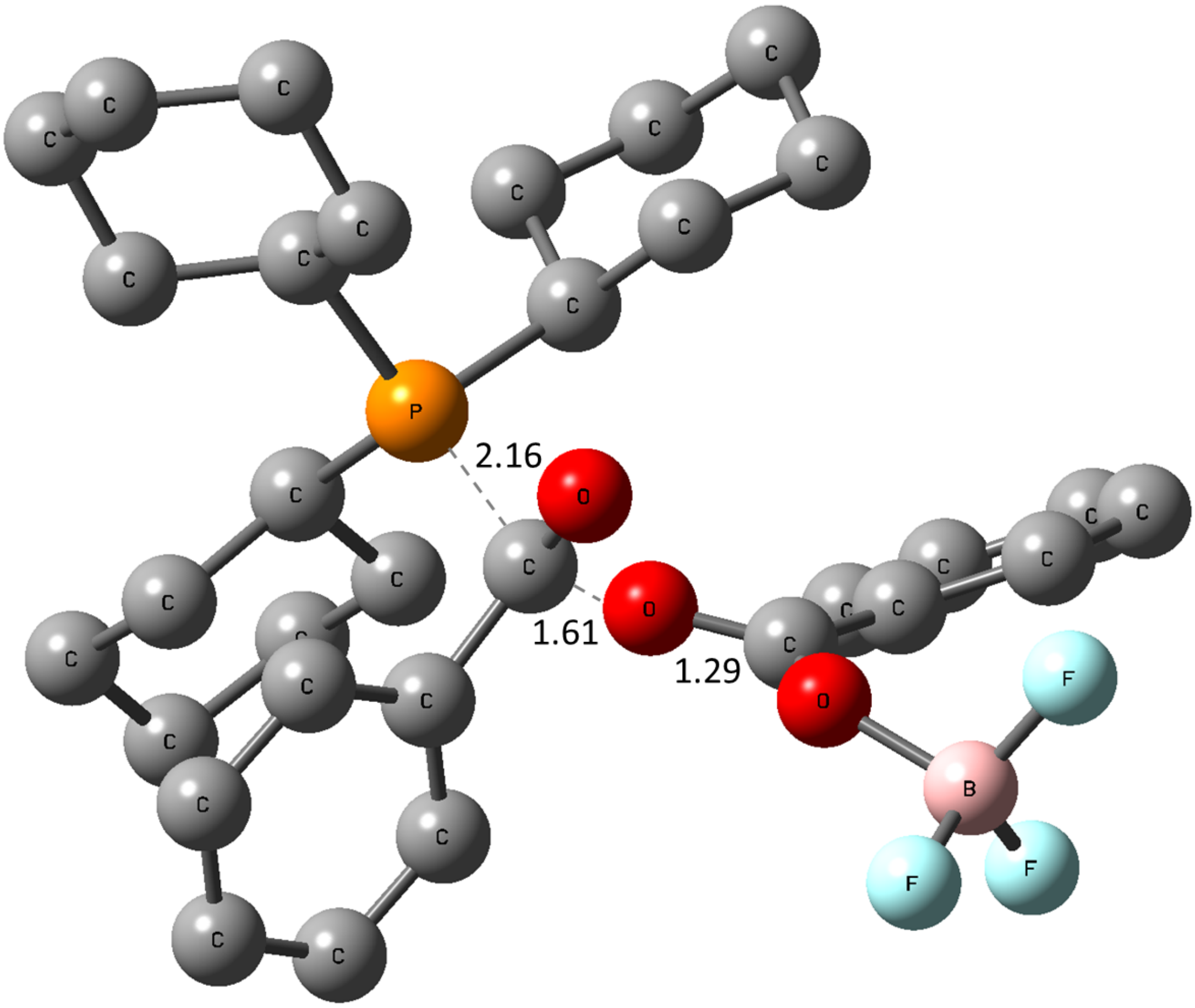

Figure S.6.40 Selected bong lengths (Å) for **TS-s6**. Some hydrogens have been omitted for clarity.

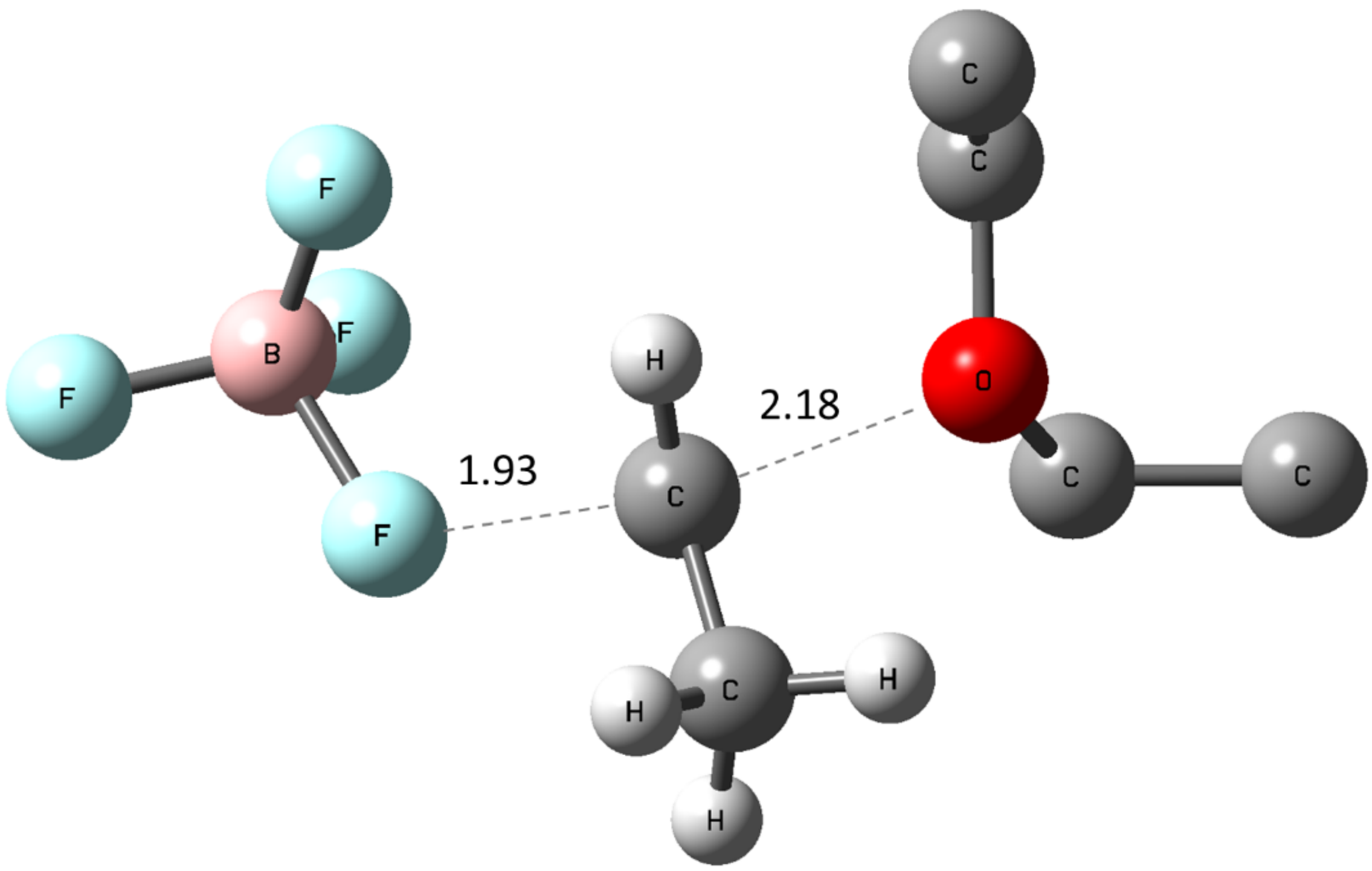

Figure S.6.41 Selected bond lengths (Å) for **TS-s7a** = **TS-4**. Some hydrogens have been omitted for clarity.

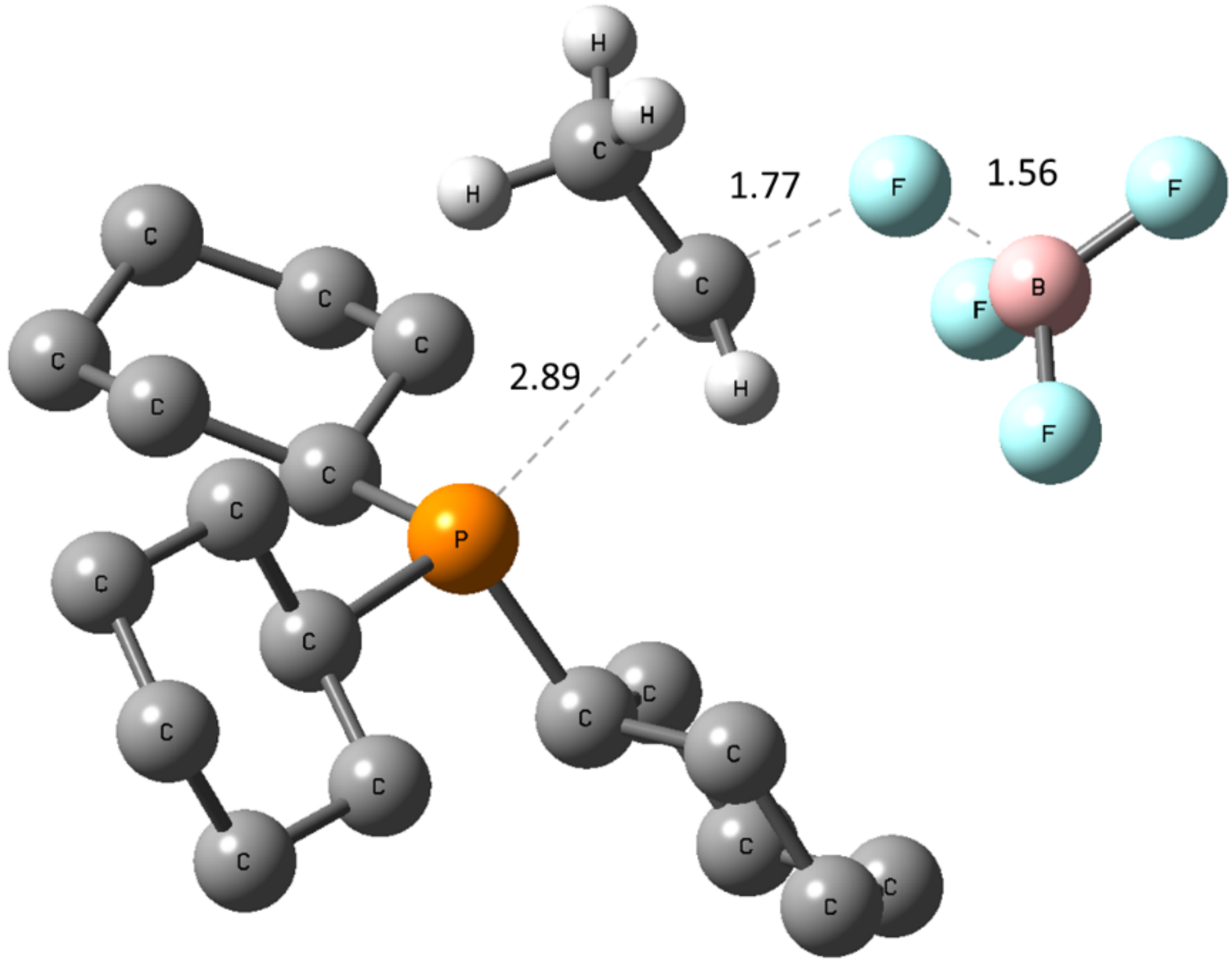

Figure S. 6.42 Selected bond lengths (Å) for **TS-s7b**. Some hydrogens have been omitted for clarity.

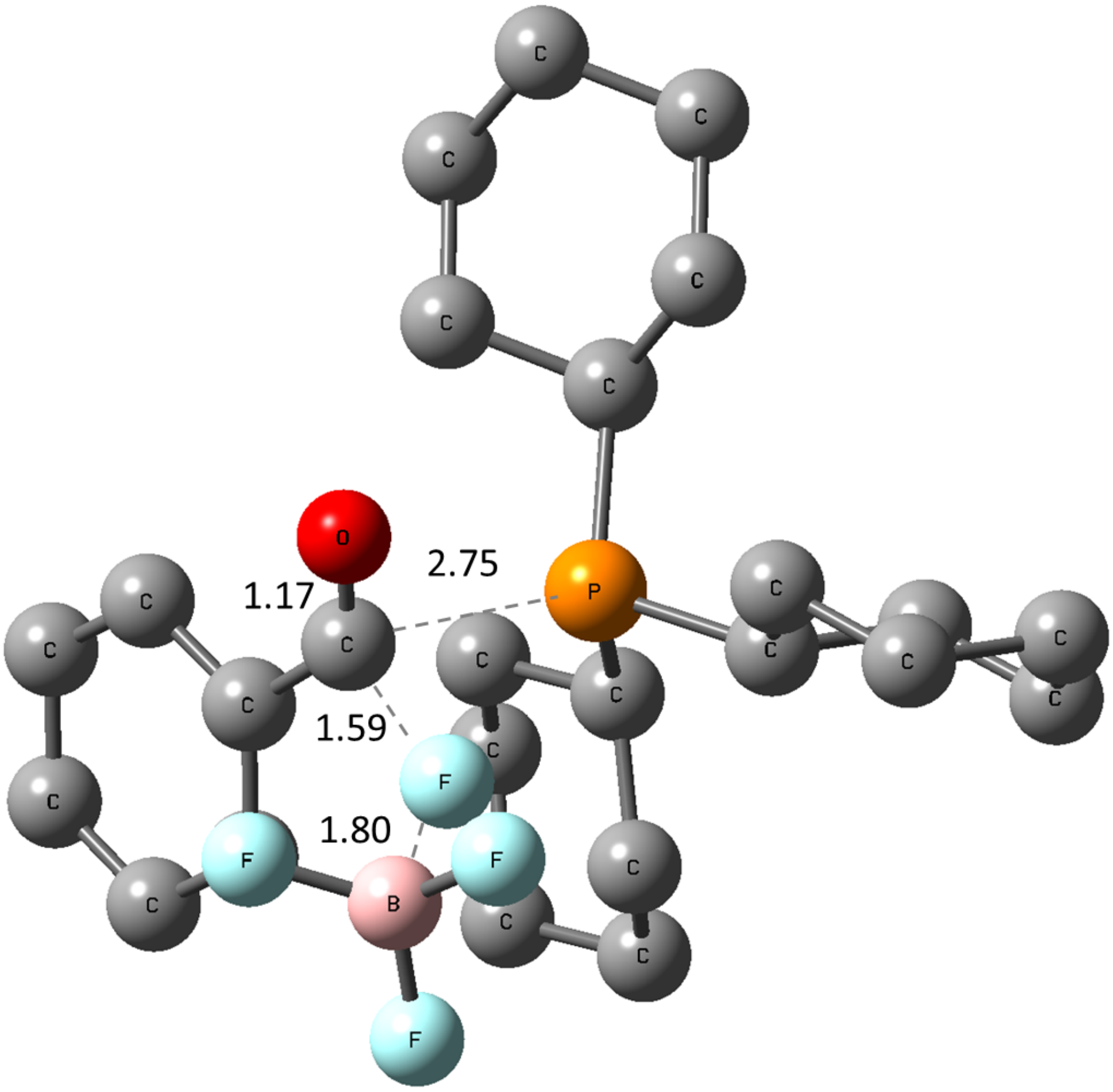

Figure S.6.43 Selected bond lengths (Å) for **TS-s8** = **TS-5**. Some hydrogens have been omitted for clarity.

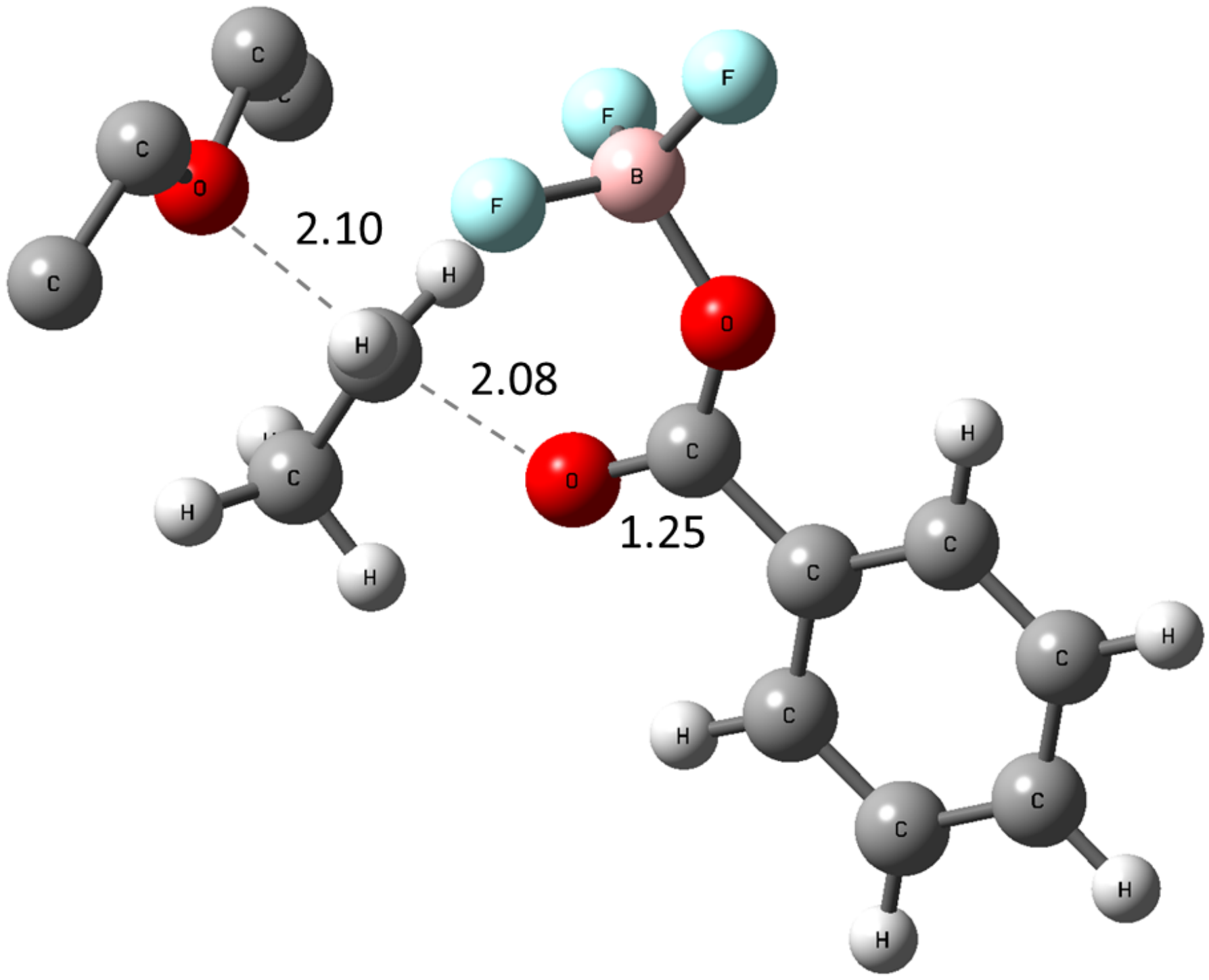

Figure S.6.44 Selected bond lengths (Å) for **TS-s9a** = **TS-6**. Some hydrogens have been omitted for clarity.

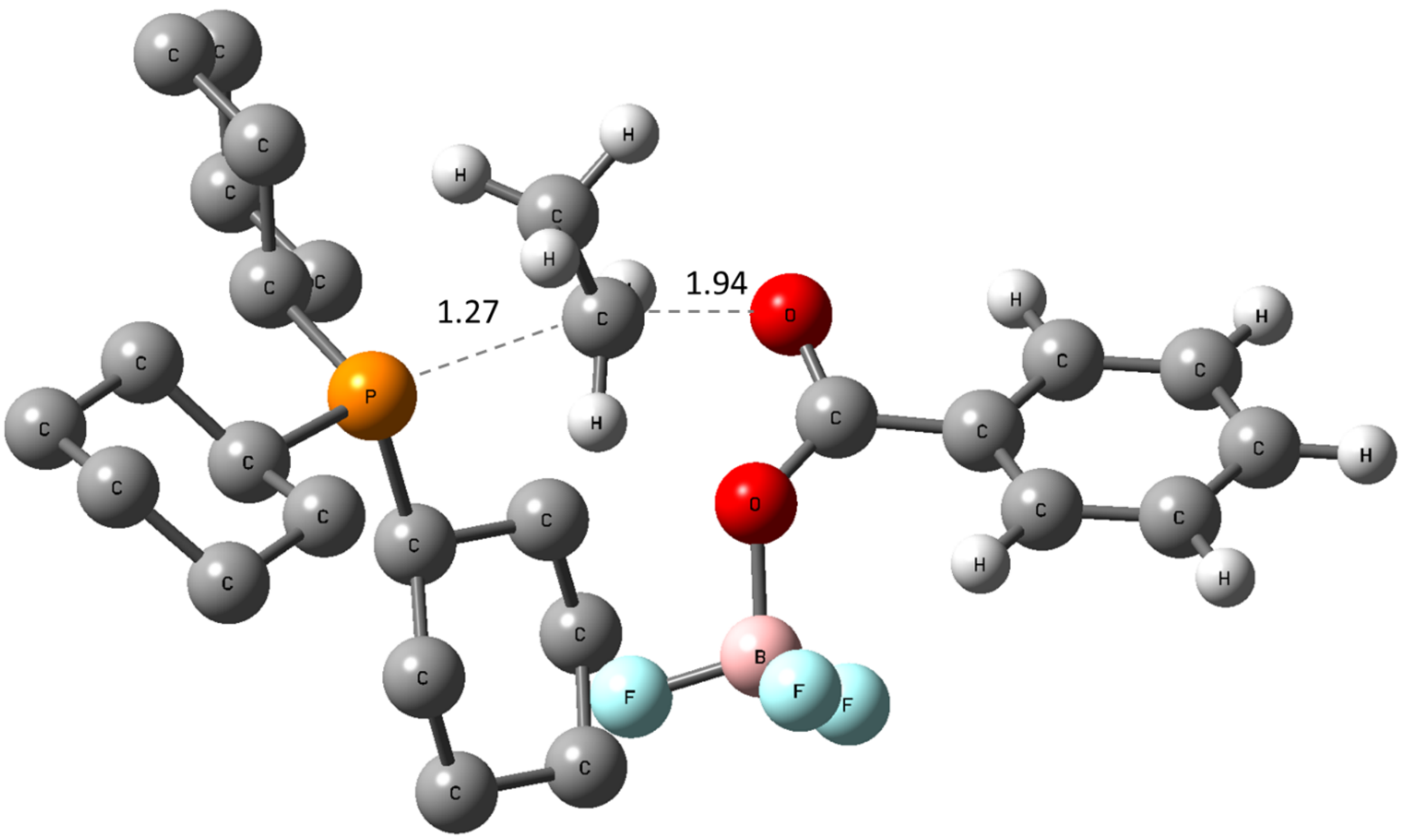

Figure S.6.45 Selected bond lengths (Å) for **TS-s9b**. Some hydrogens have been omitted for clarity.

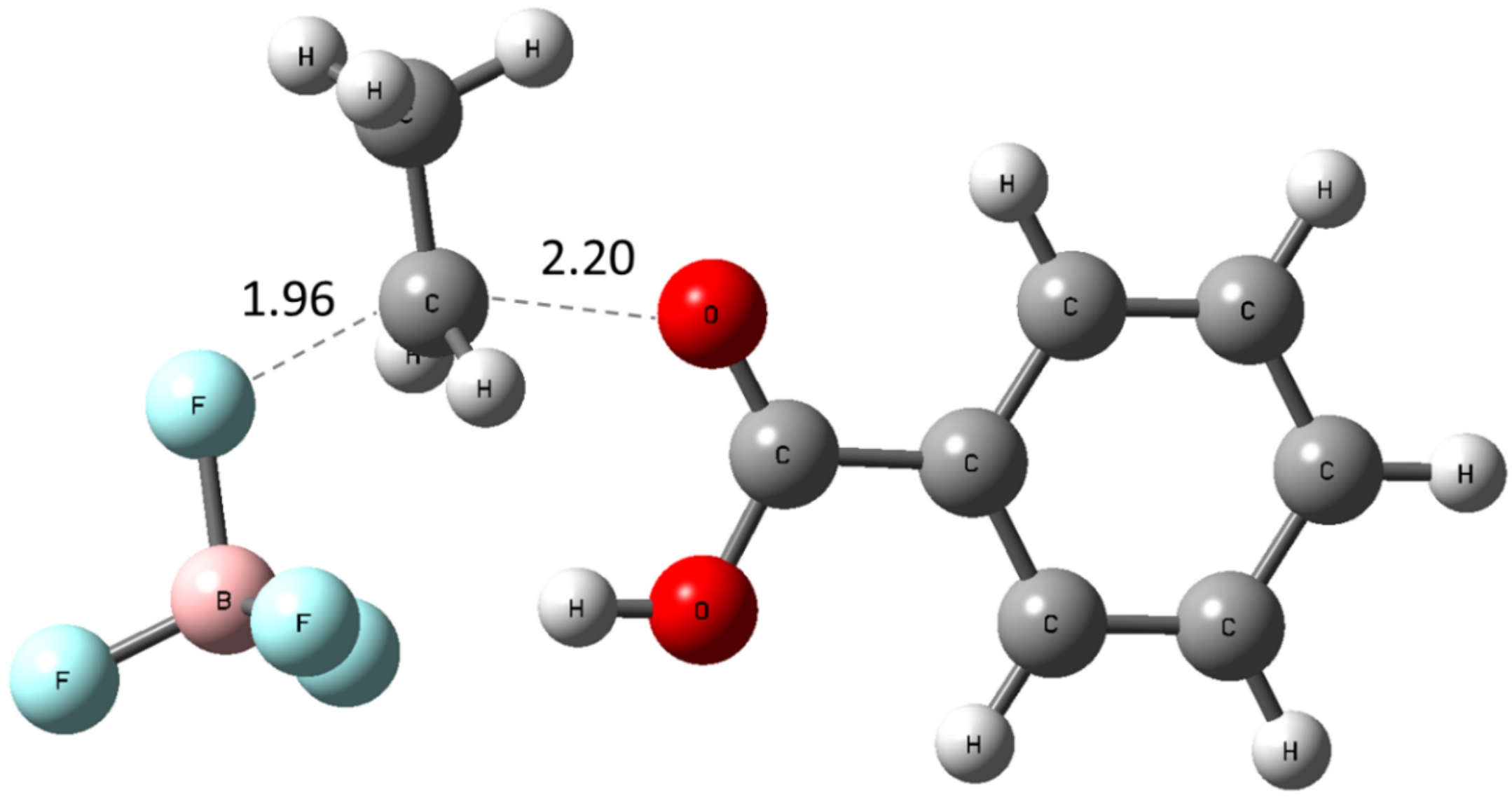

Figure S.6.46 Selected bond lengths (Å) for **TS-s10**. Some hydrogens have been omitted for clarity.

6.2.4 Intrinsic reaction coordinate plots

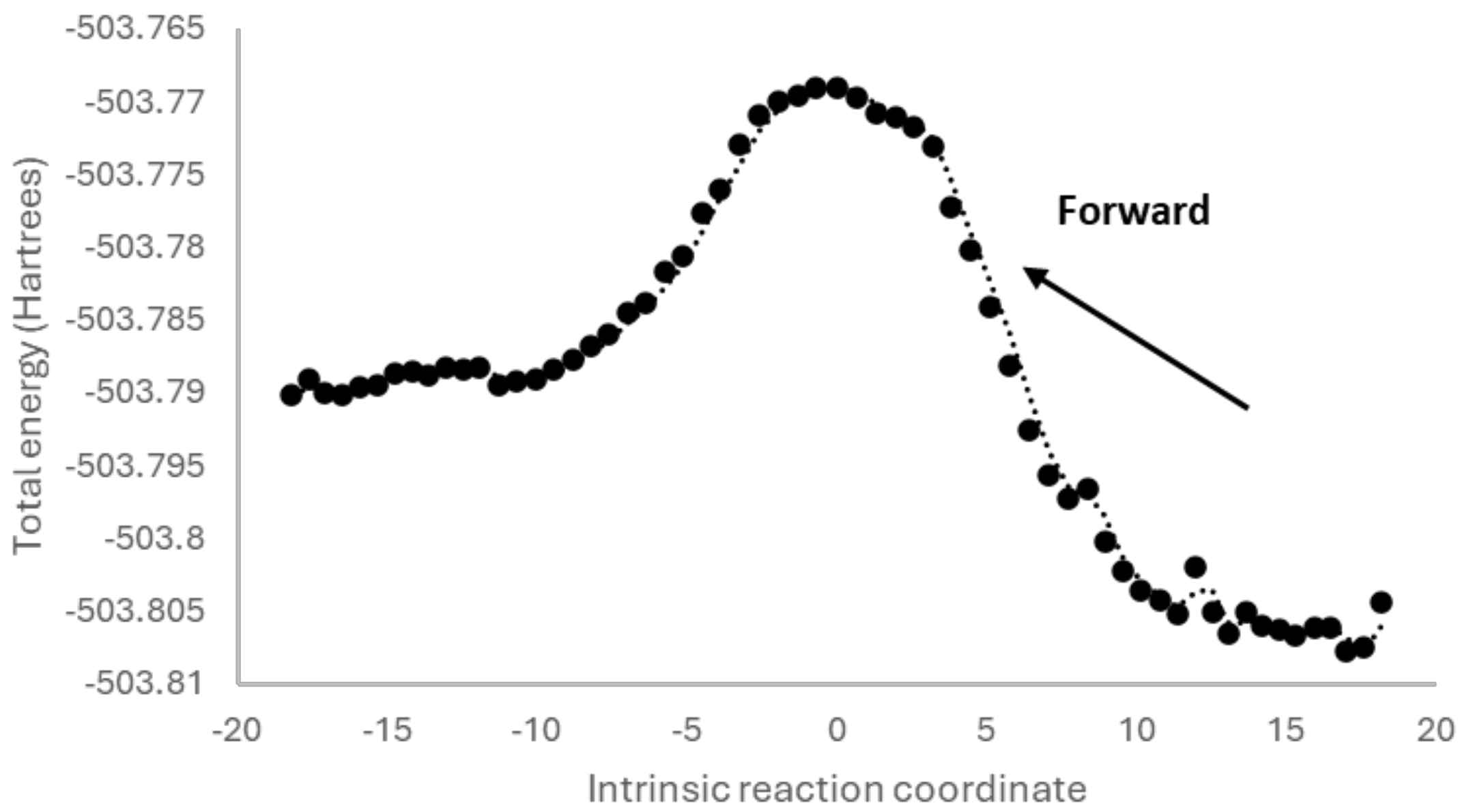

Figure S.6.47 Intrinsic reaction coordinate plot for **TS-s1a** = **TS-1**.

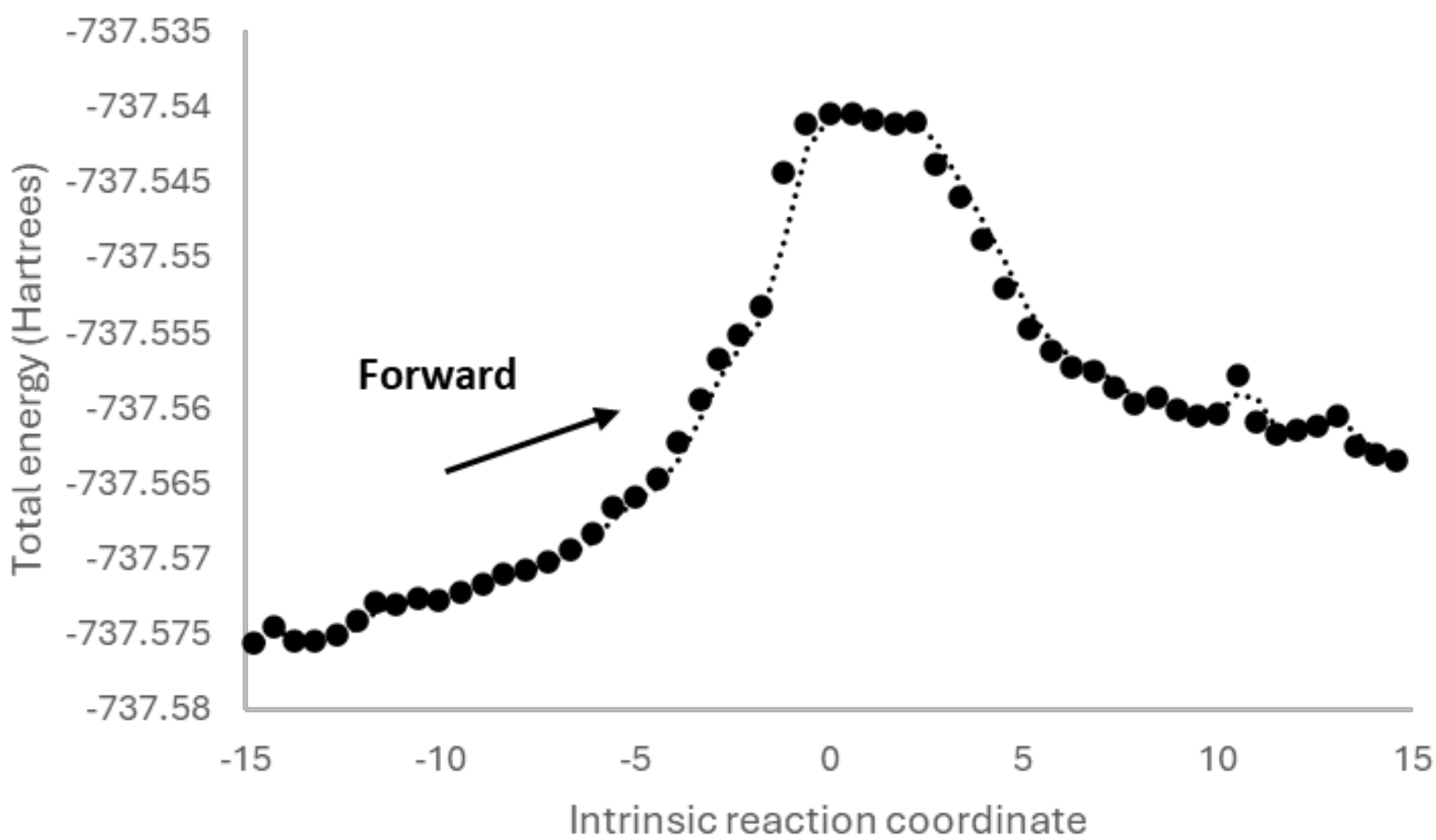

Figure S.6.48 Intrinsic reaction coordinate plot for **TS-s1b**.

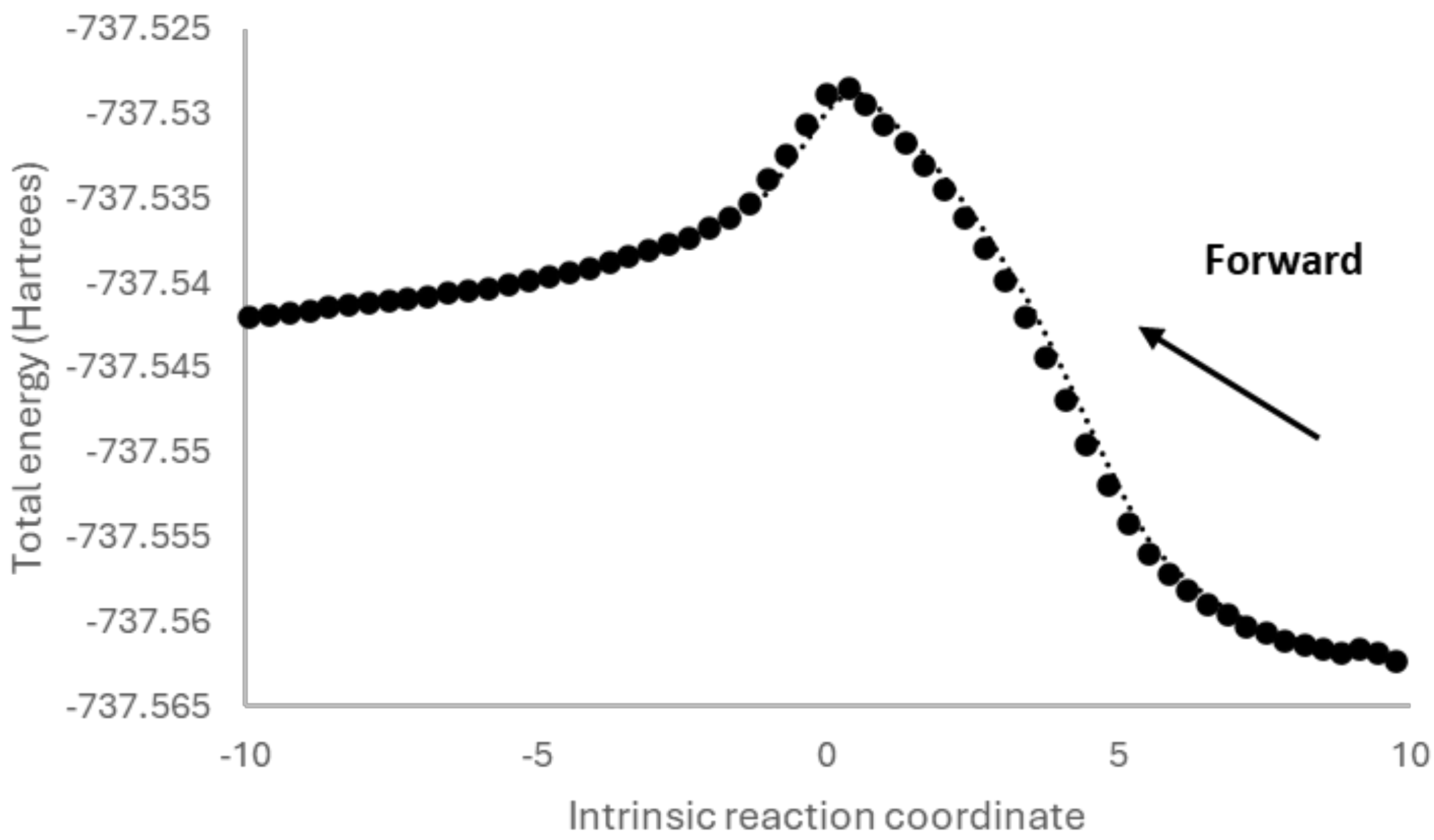

Figure S.6.49 Intrinsic reaction coordinate plot for **TS-s1c**.

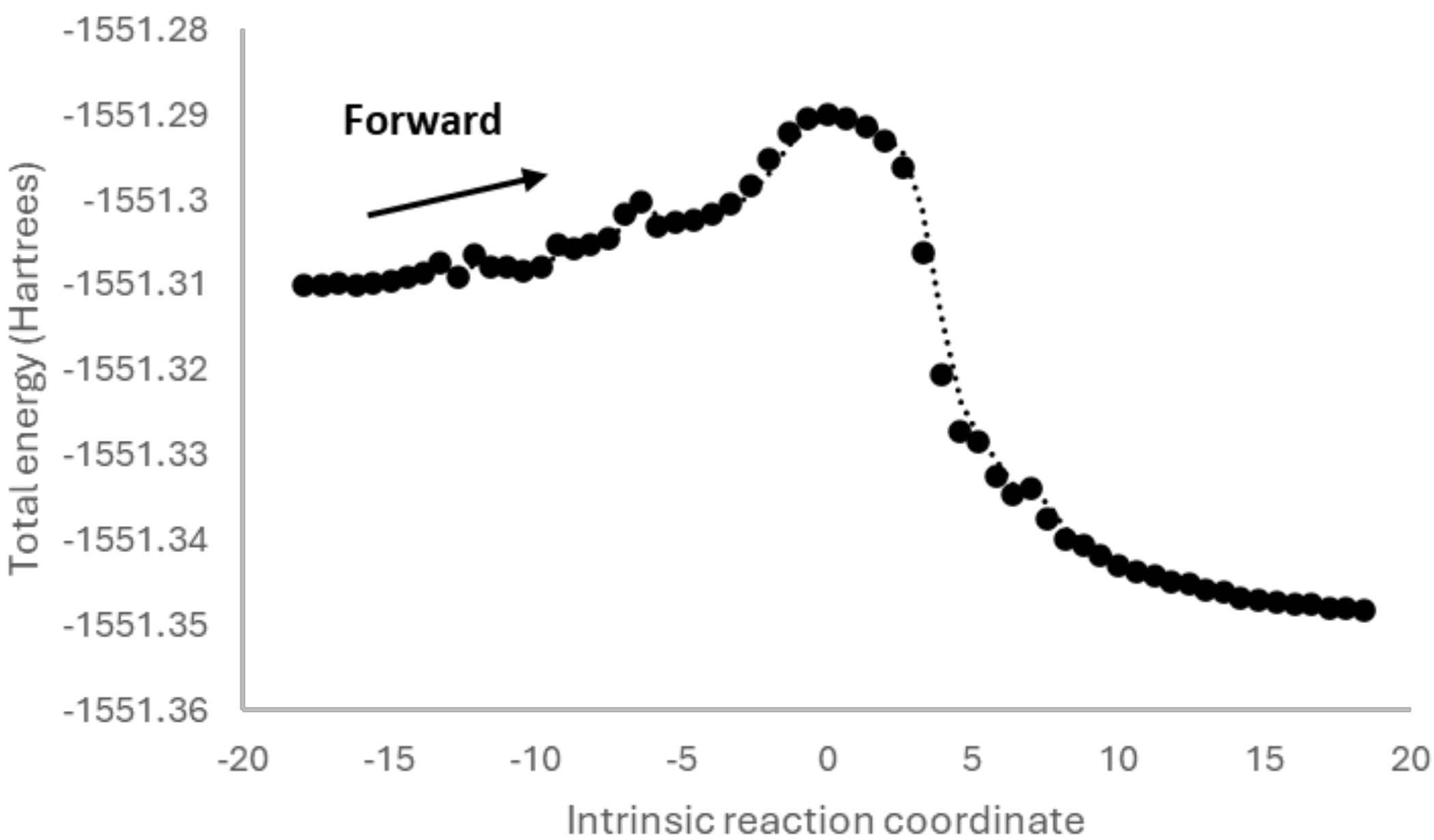

Figure S.6.50 Intrinsic reaction coordinate plot for **TS-s1d**.

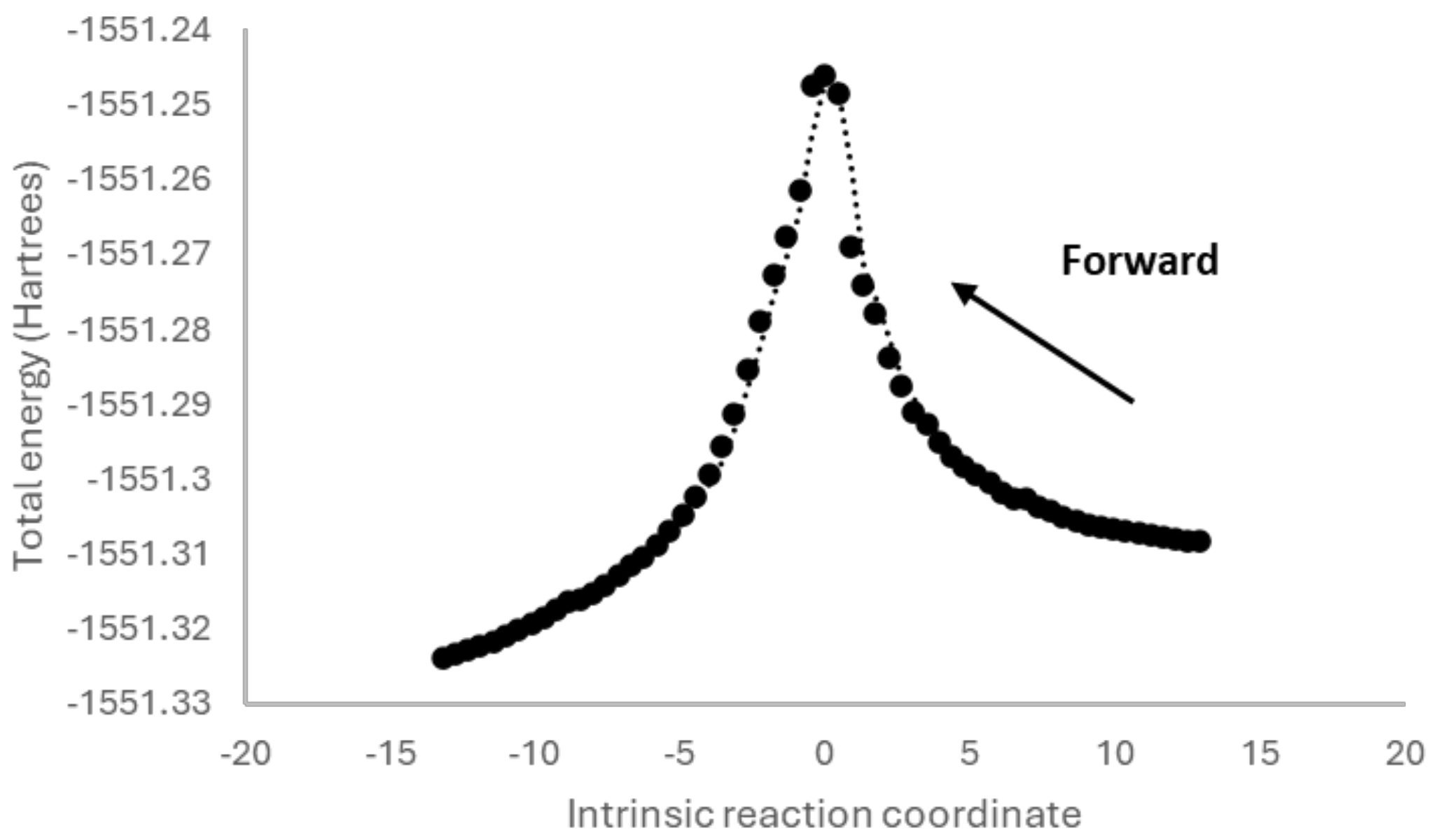

Figure S.6.51 Intrinsic reaction coordinate plot for **TS-s1e**.

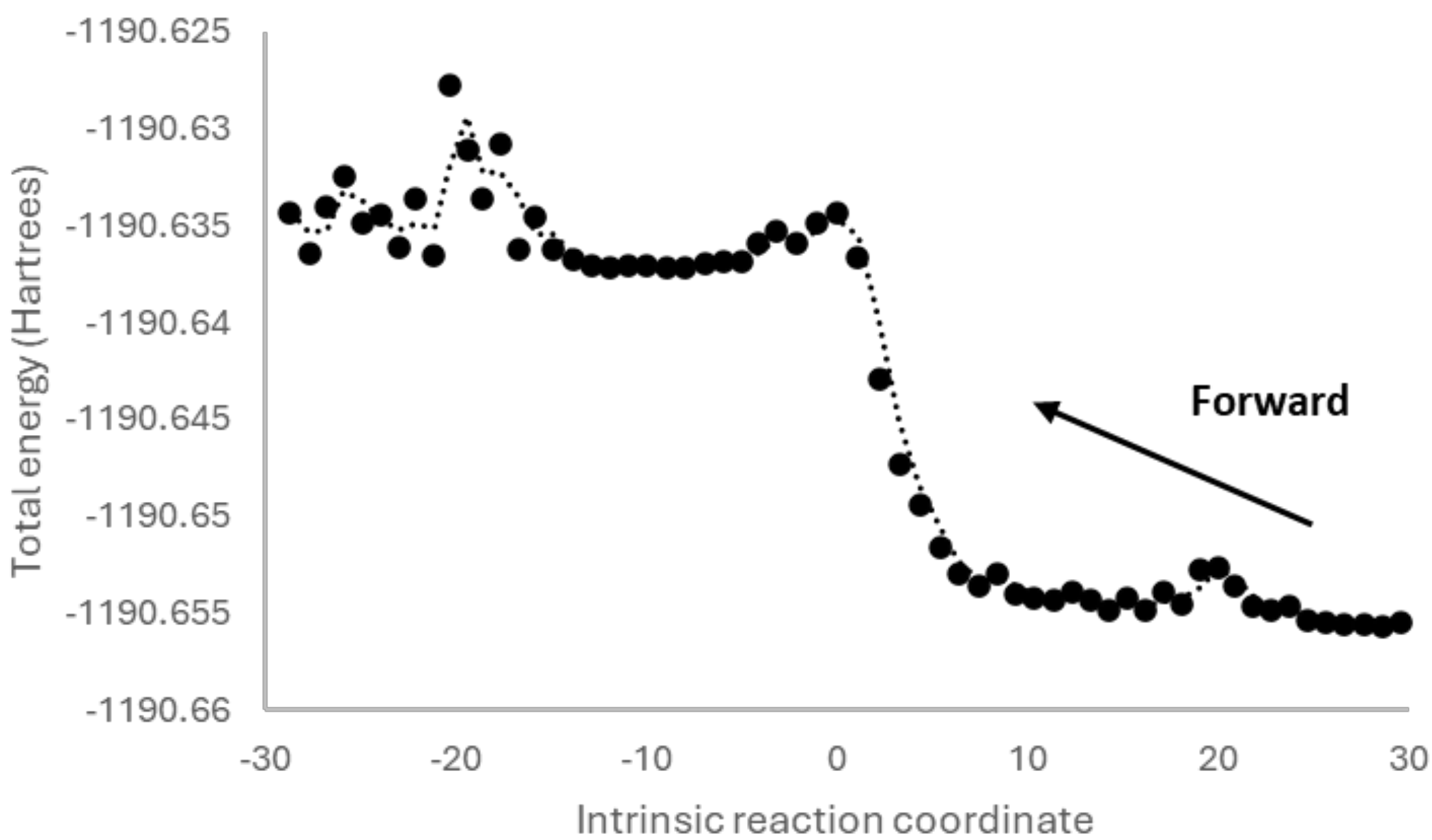

Figure S.6.52 Intrinsic reaction coordinate plot for **TS-s2** = **TS-2**.

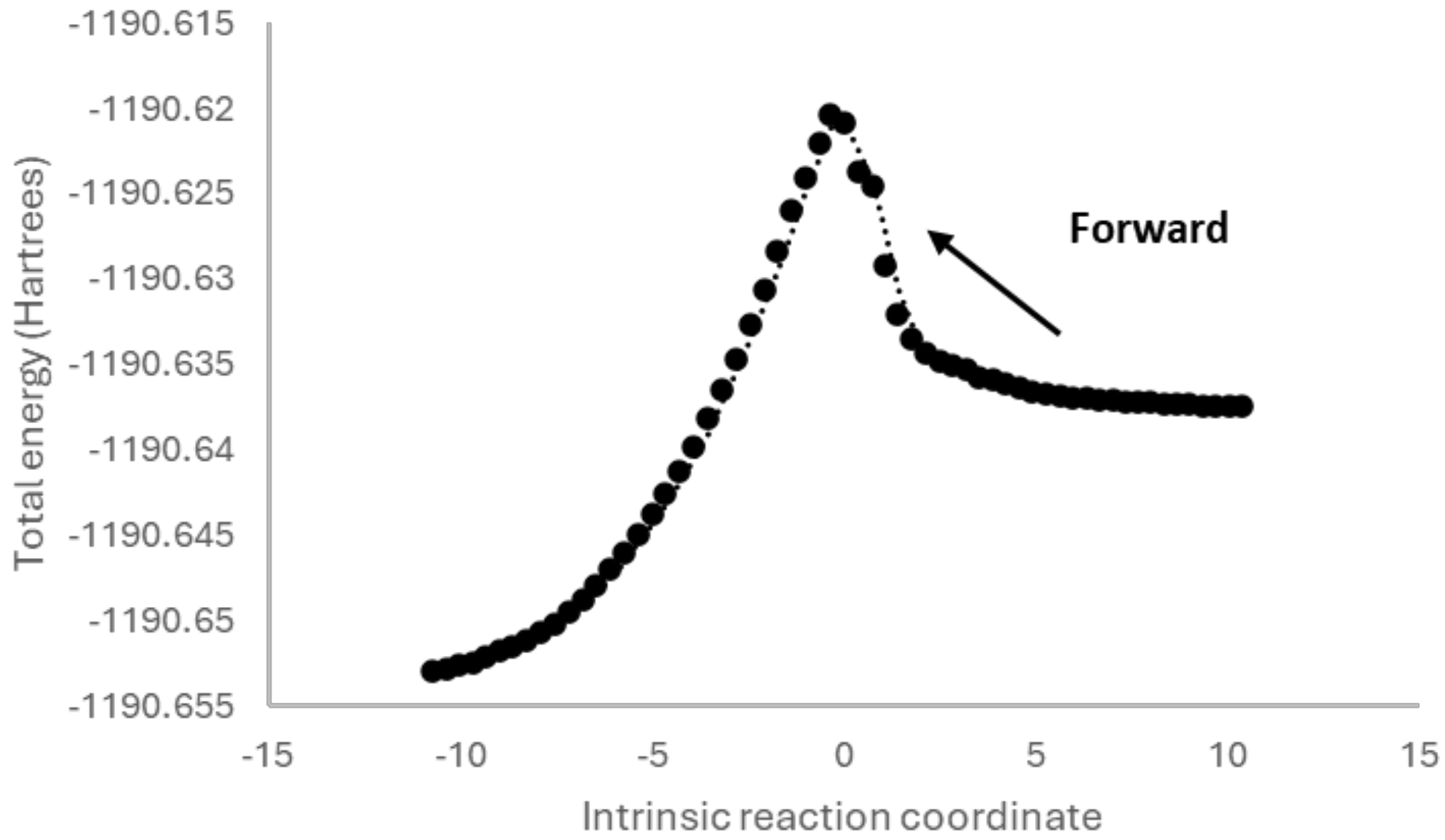

Figure S.6.53 Intrinsic reaction coordinate plot for **TS-s3** = **TS-3**.

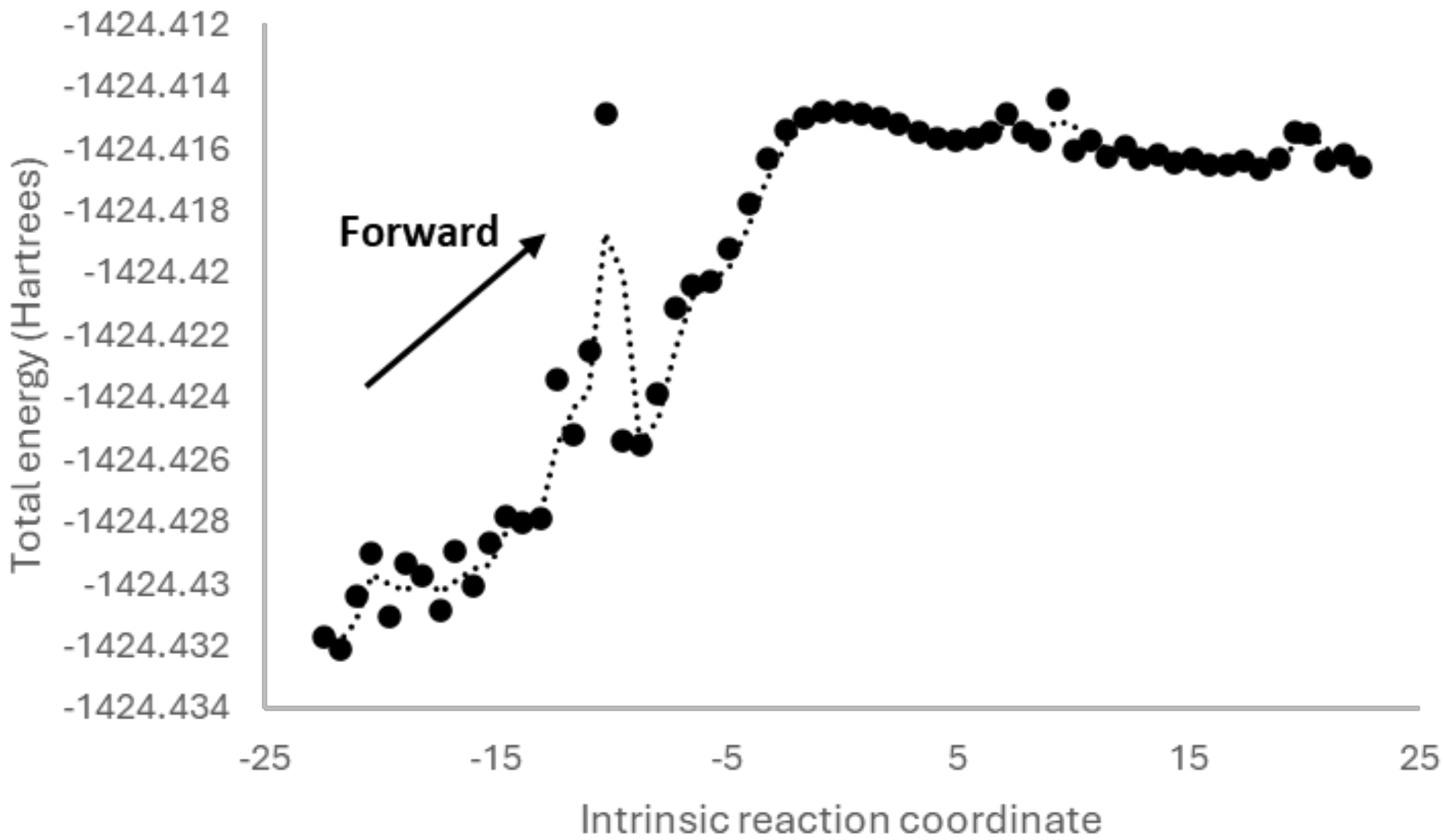

Figure S.6.54 Intrinsic reaction coordinate plot for **TS-s4a**.

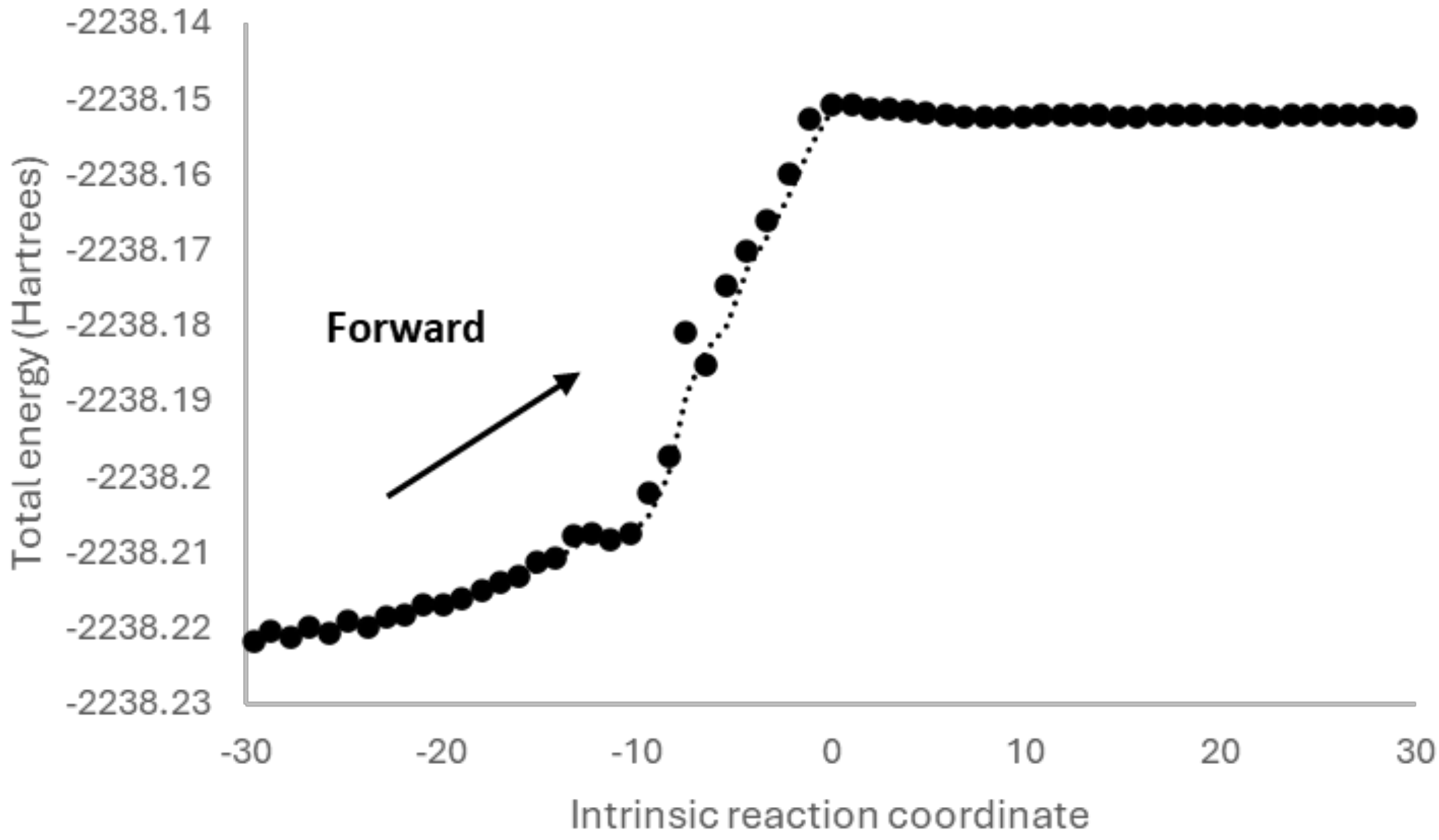

Figure S.6.55 Intrinsic reaction coordinate plot for **TS-s4b**.

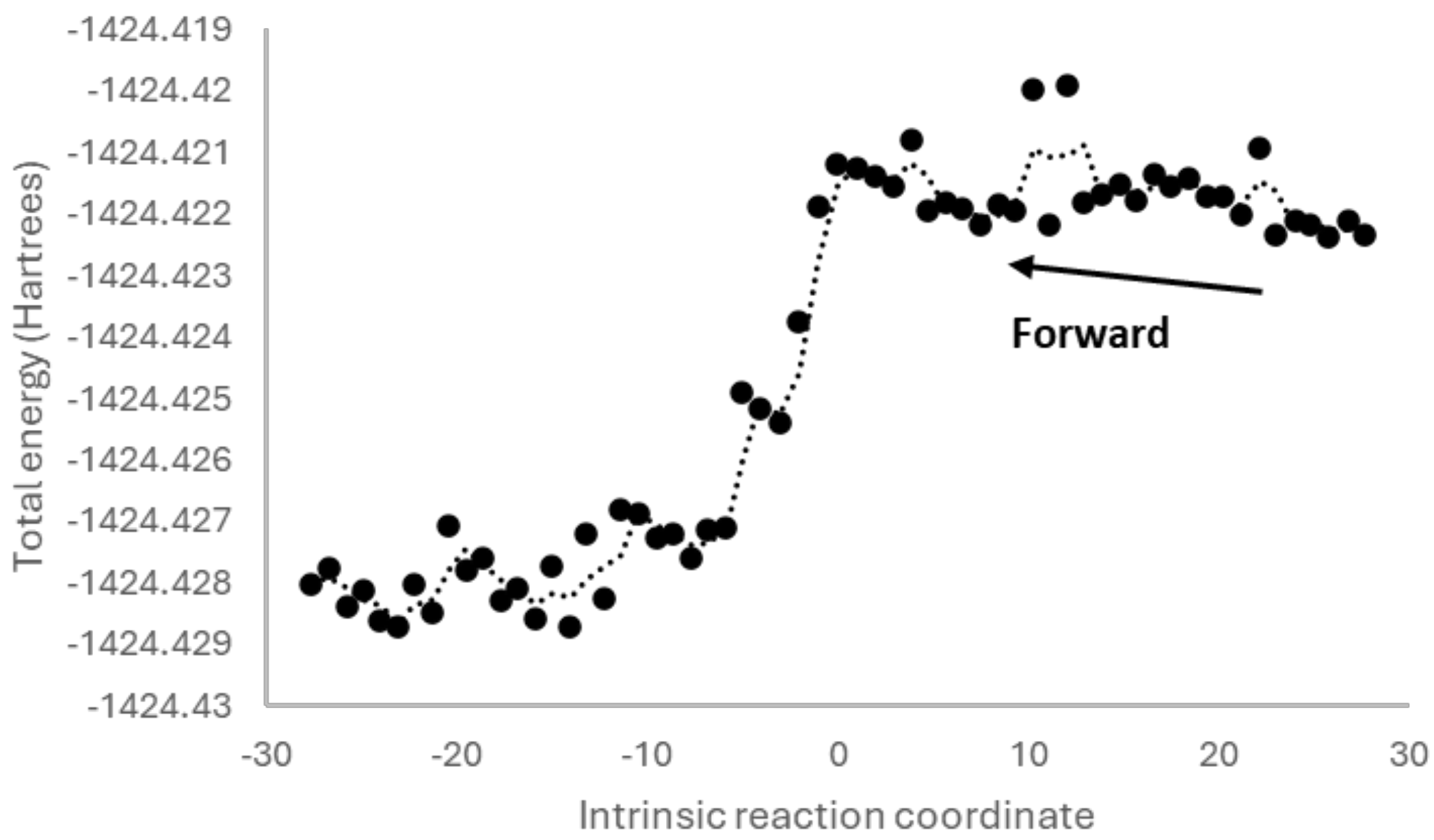

Figure S.6.56 Intrinsic reaction coordinate plot for **TS-s5a**.

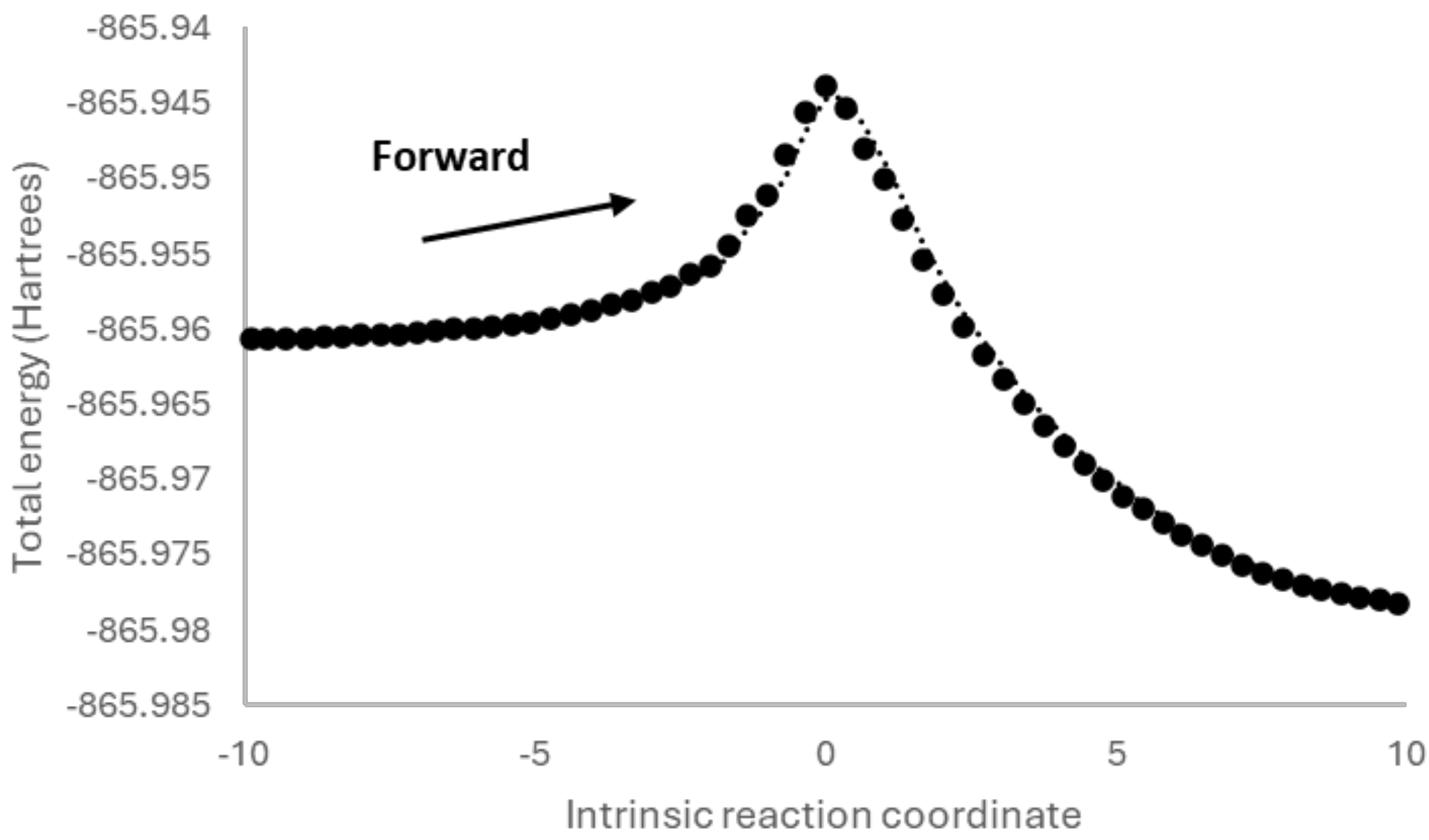

Figure S.6.57 Intrinsic reaction coordinate plot for **TS-s5b.**

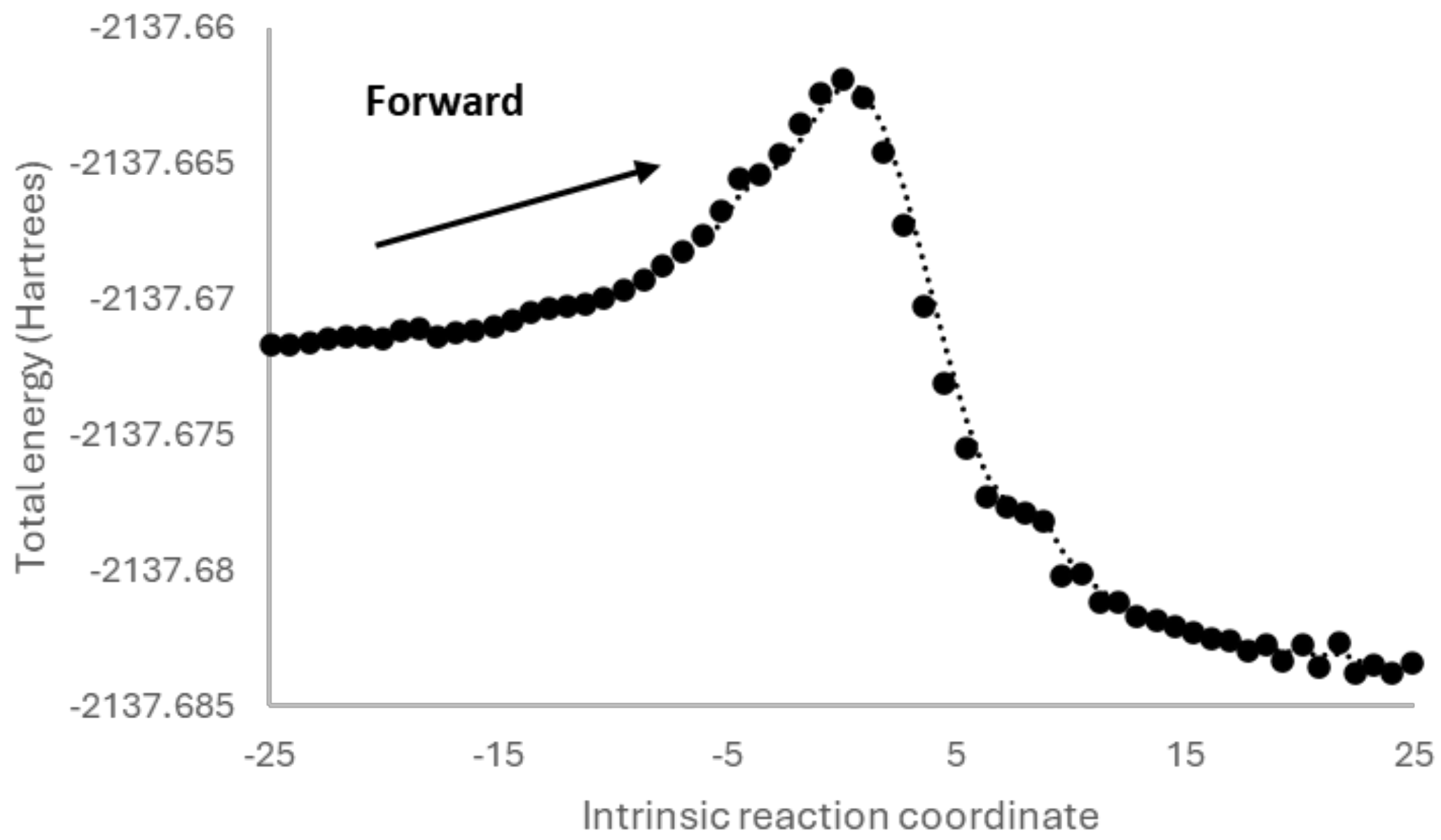

Figure S.6.58 Intrinsic reaction coordinate plot for **TS-s6**.

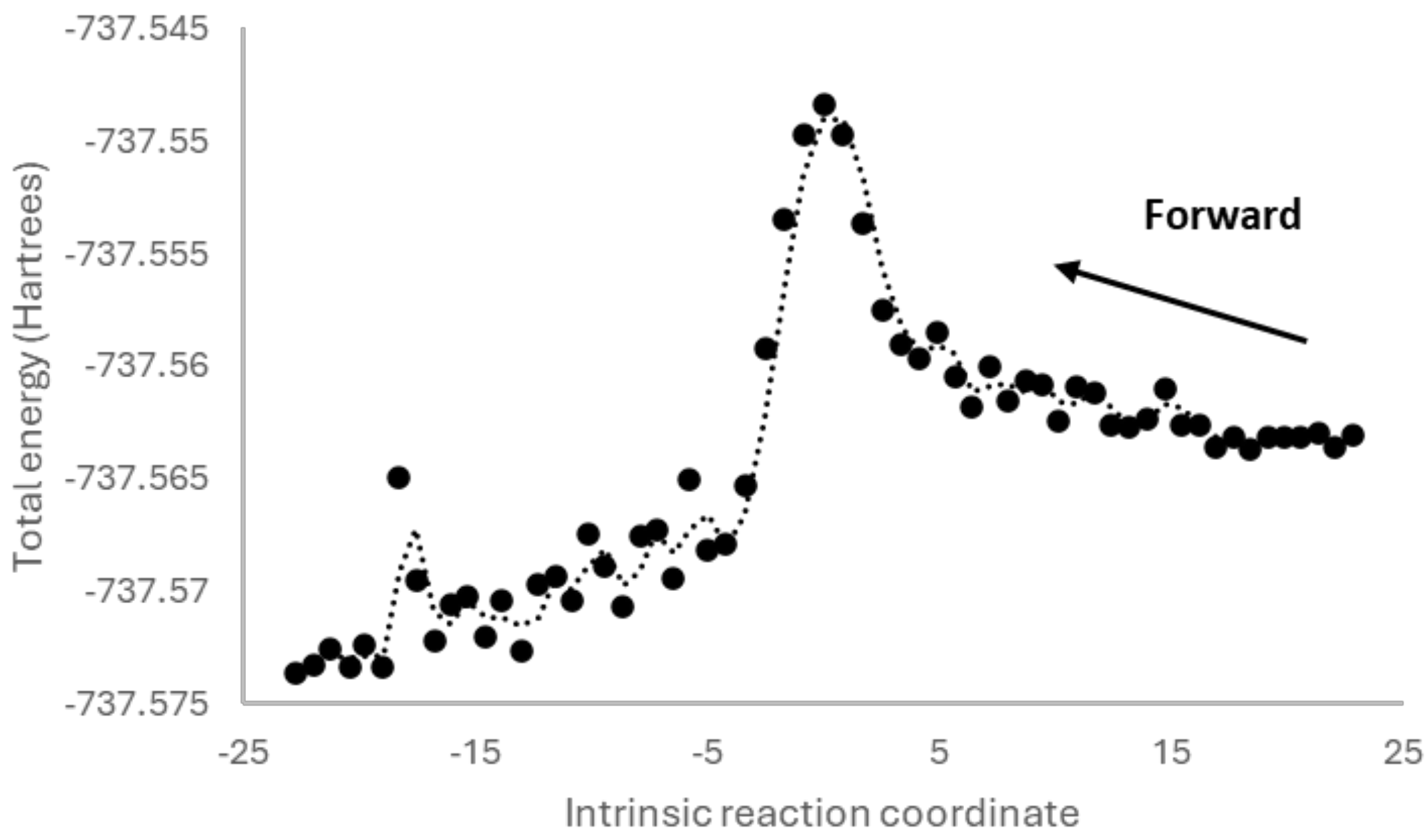

Figure S.6.59 Intrinsic reaction coordinate plot for **TS-s7a** = **TS-4**.

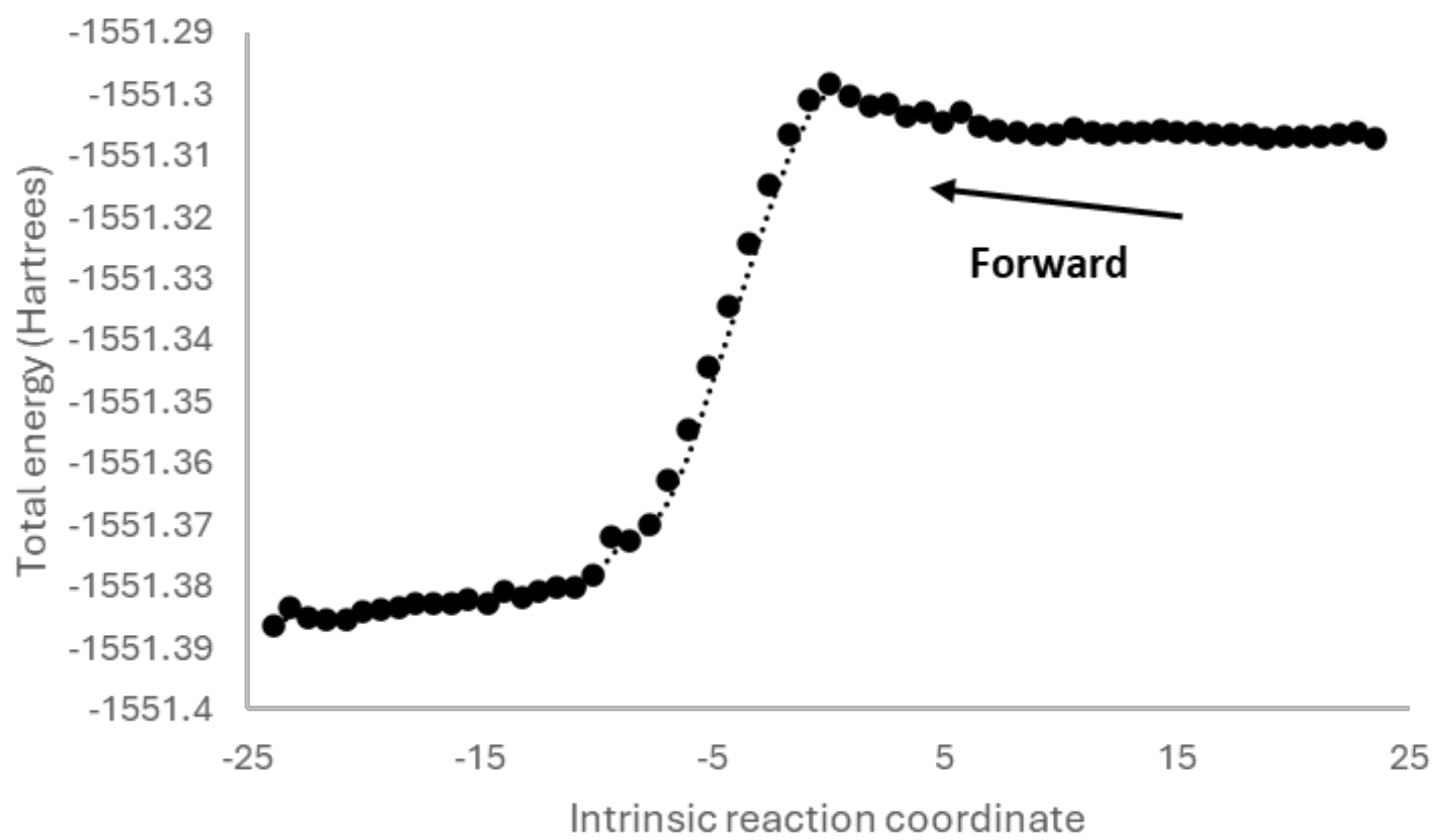

Figure S.6.60 Intrinsic reaction coordinate plot for **TS-s7b**.

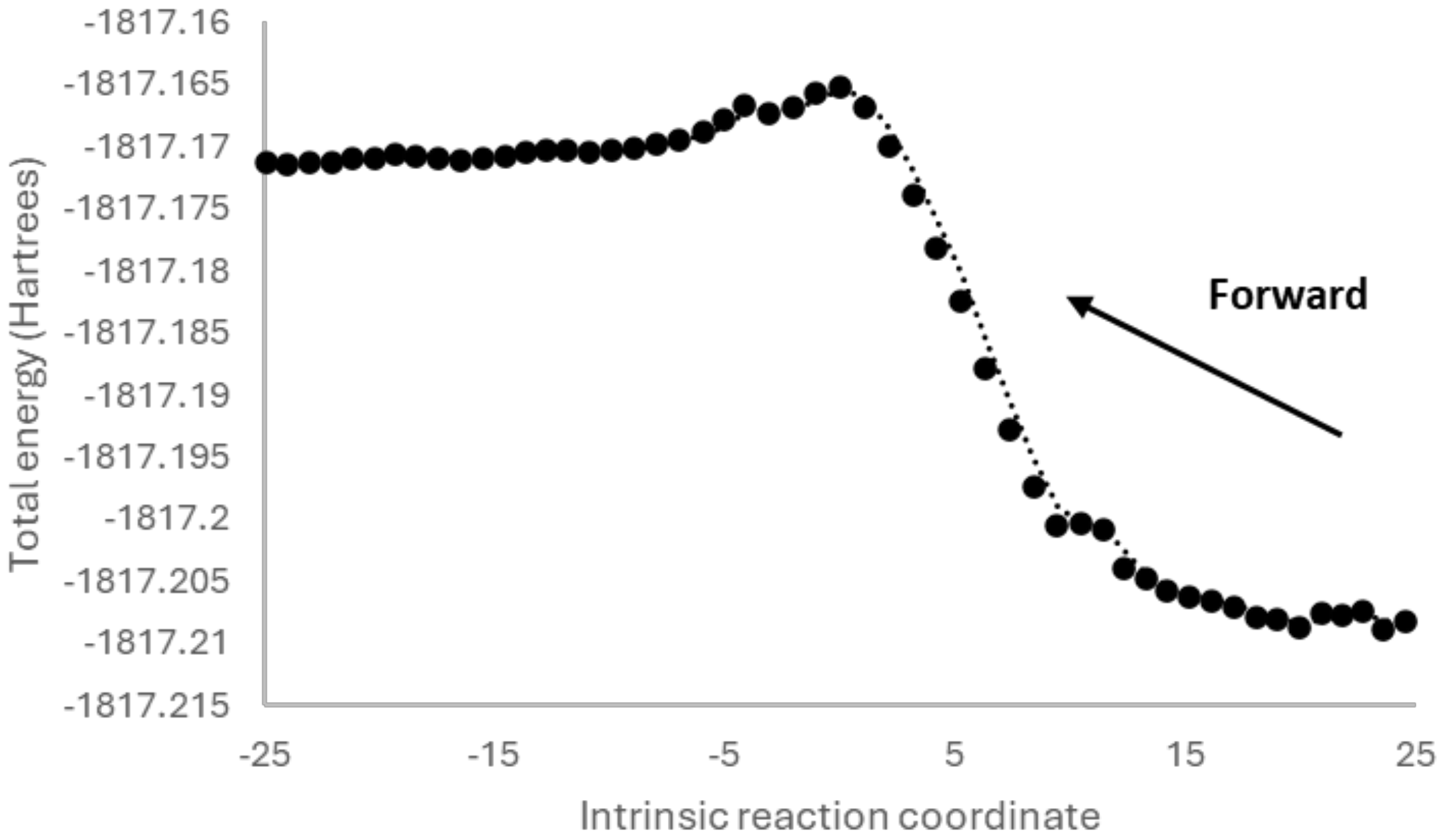

Figure S.6.61 Intrinsic reaction coordinate plot for **TS-s8** = **TS-5**.

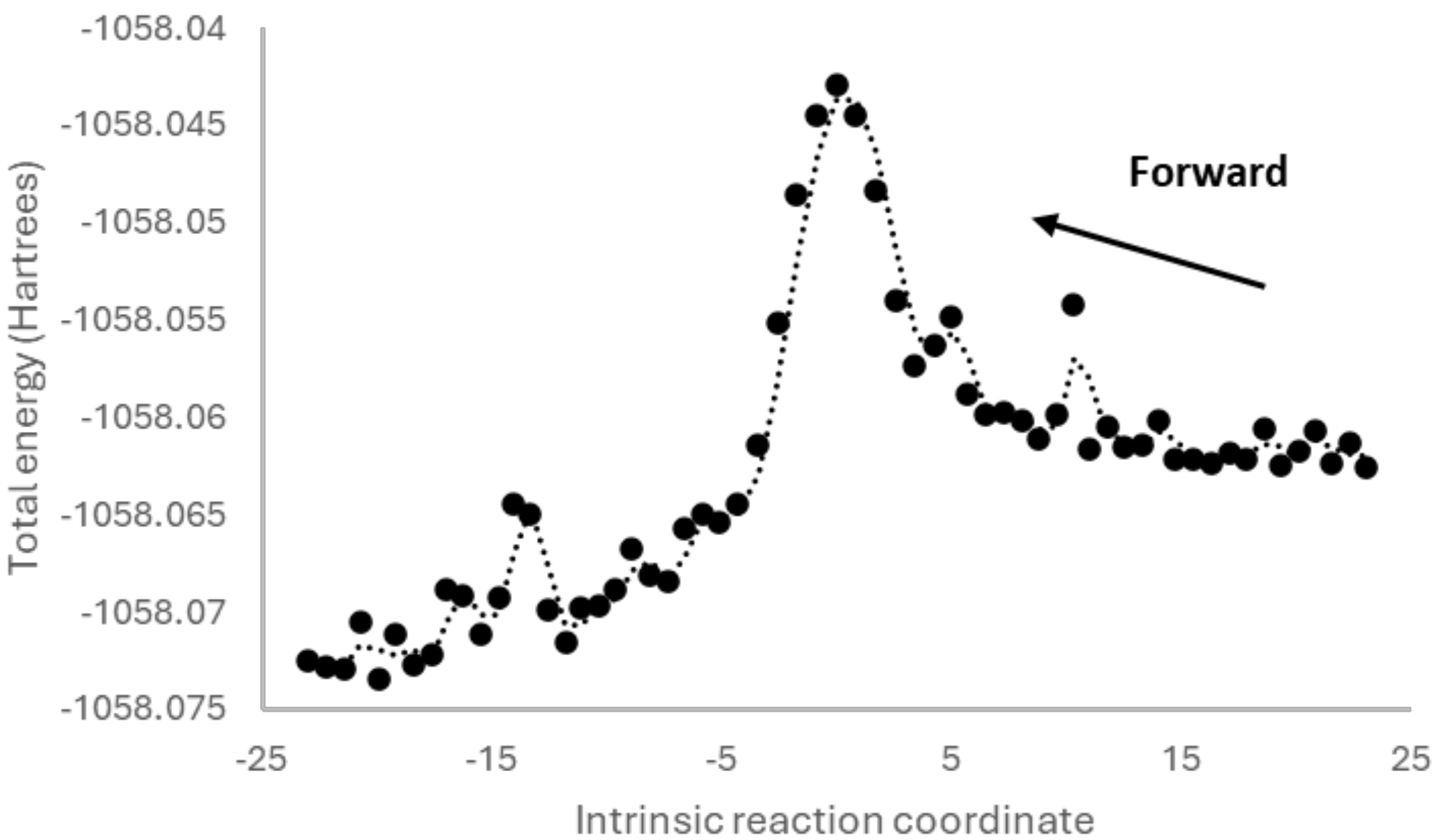

Figure S.6.62 Intrinsic reaction coordinate plot for **TS-s9a** = **TS-6**.

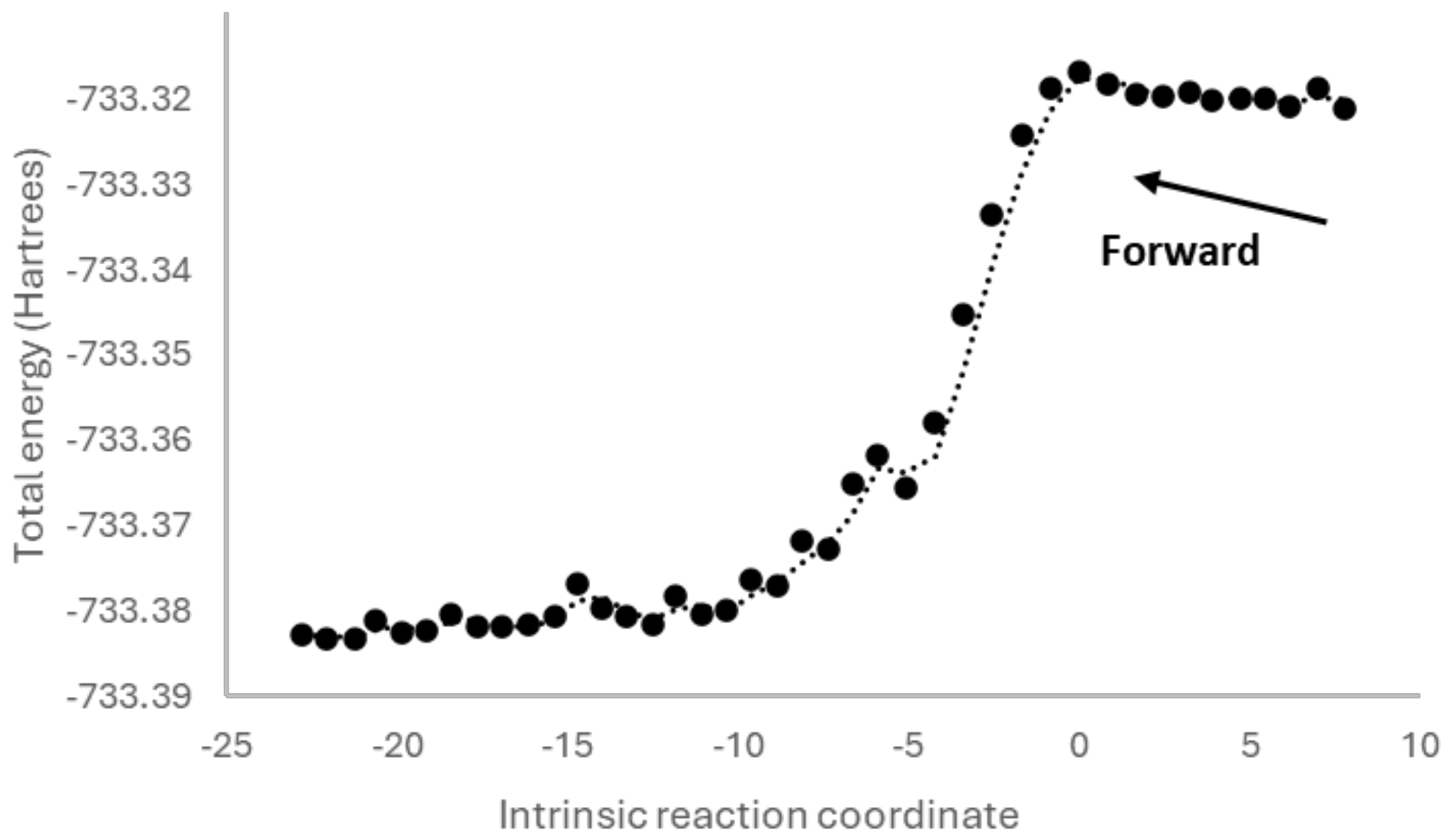

Figure S.6.63 Intrinsic reaction coordinate plot for **TS-s9b**.

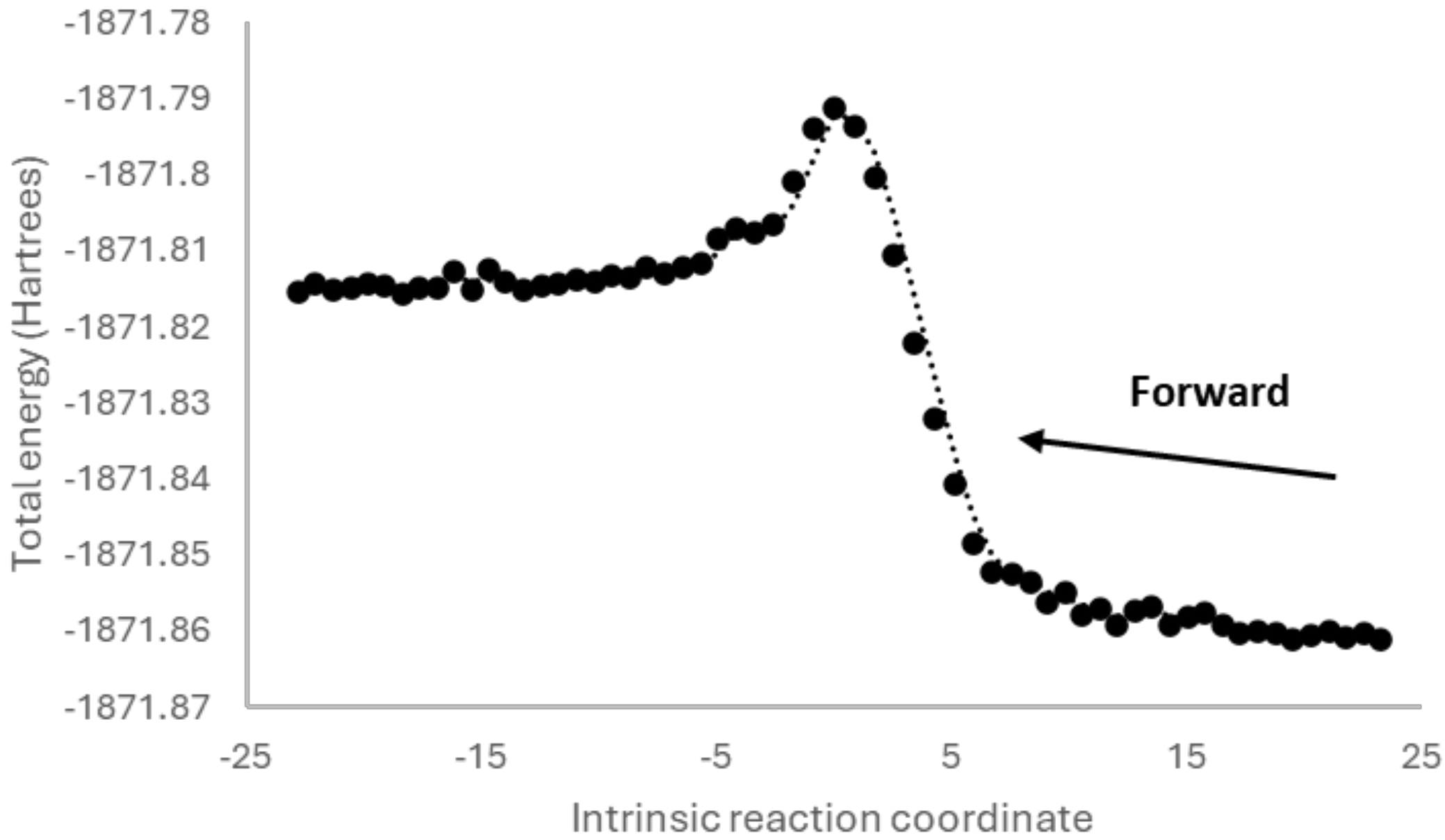

Figure S.6.64 Intrinsic reaction coordinate plot for **TS-s9c**.

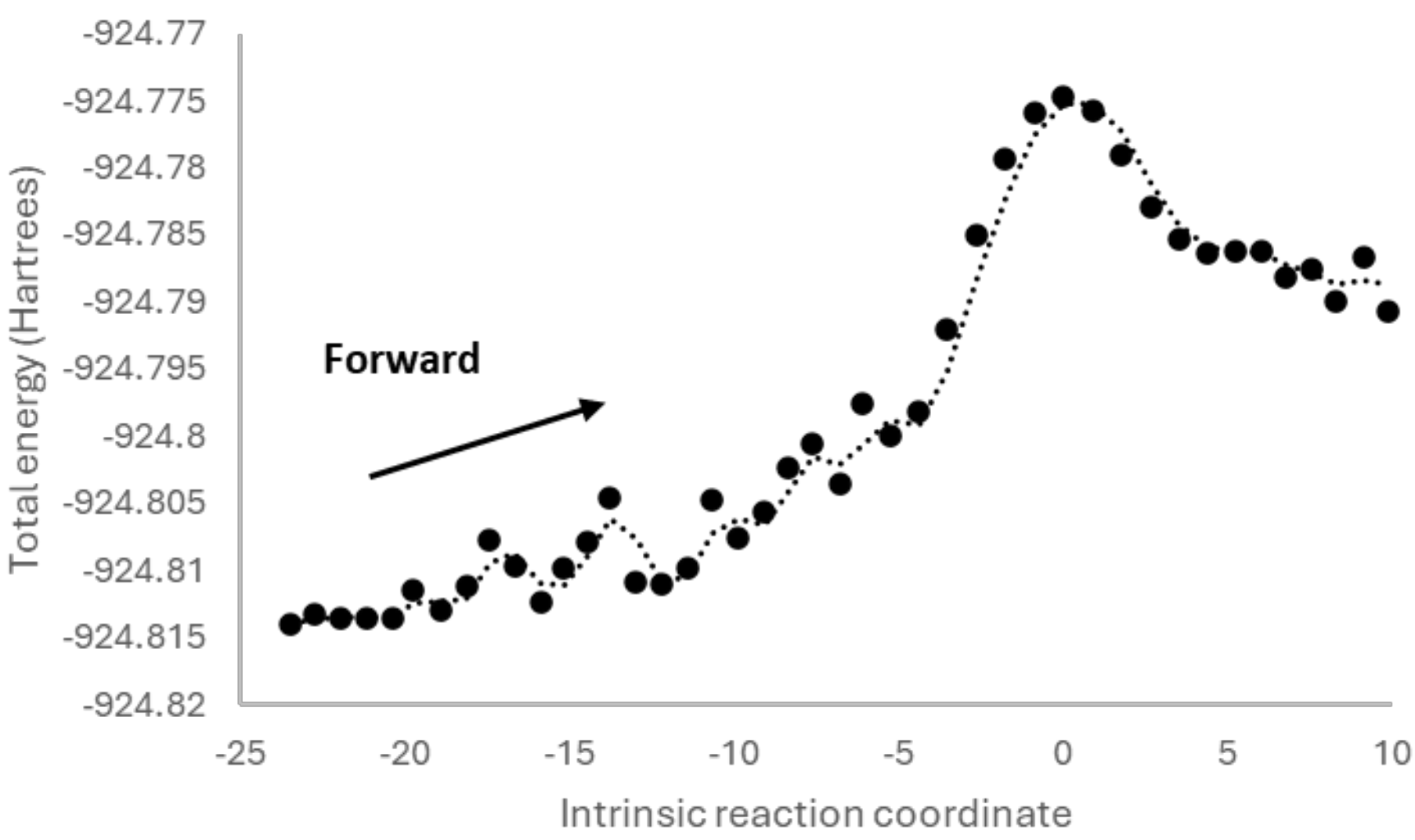

Figure S.6.65 Intrinsic reaction coordinate plot for **TS-s10**.

6.2.5 General pathway

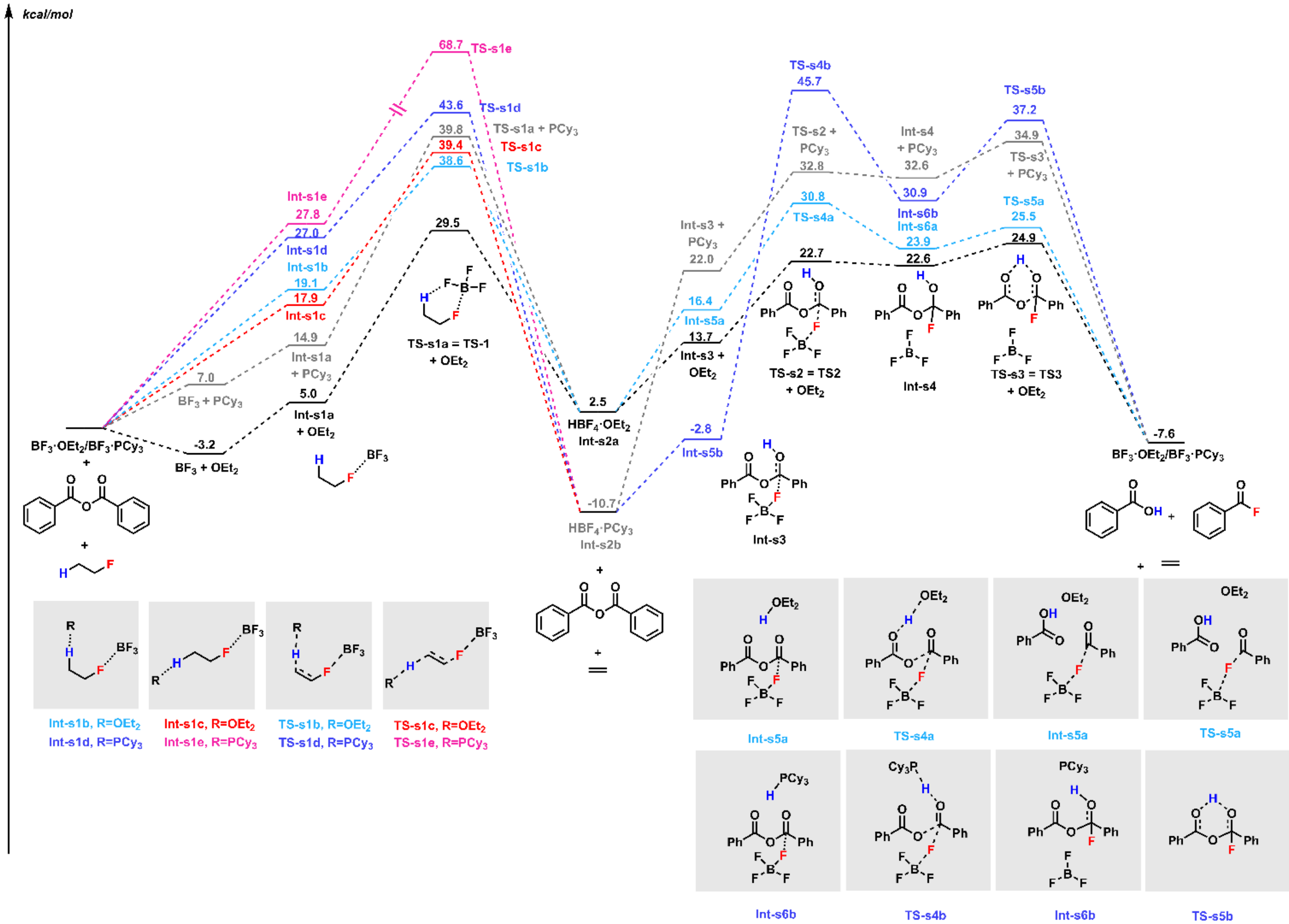

Figure S.6.66 DFT calculated pathway for the boron trifluoride catalysed shuttle reaction of fluoroethane and benzoic anhydride. Calculated using the B3LYP hybrid functional. The 6-311++G** basis set was used for optimisation with a PCM solvation model, and single-point corrections were done using def2-QZVPPD with a SMD solvation model. Dispersion was included (GD3BJ). GoodVibes corrections were performed (373.15 K, 0.0901 mol L^{-1} in benzoic anhydride). Energy values reported in kcal mol^{-1}. (Cy = cyclohexyl, Ph = phenyl).

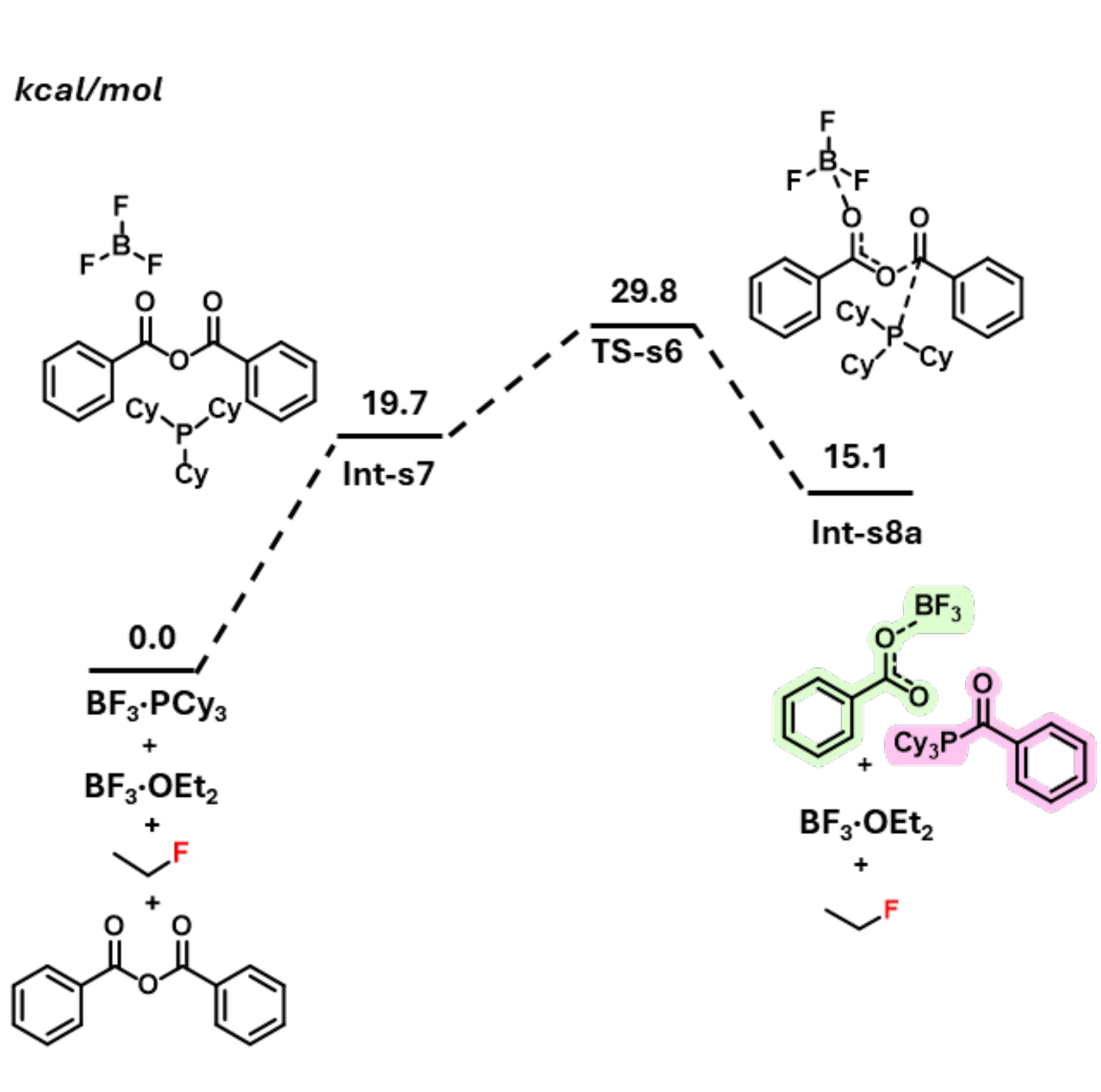

Figure S.6.67 DFT calculated pathway for the reaction of boron trifluoride tricyclohexylphosphine with benzoic anhydride to generate a phosphonium salt intermediate. Calculated using the B3LYP hybrid functional. The 6-311++G** basis set was used for optimisation with a PCM solvation model, and single-point corrections were done using def2-QZVPPD with a SMD solvation model. Dispersion was included (GD3BJ). GoodVibes corrections were performed (373.15 K, 0.0901 mol L^{-1} in benzoic anhydride). Energy values reported in kcal mol^{-1}. (Cy = cyclohexyl).

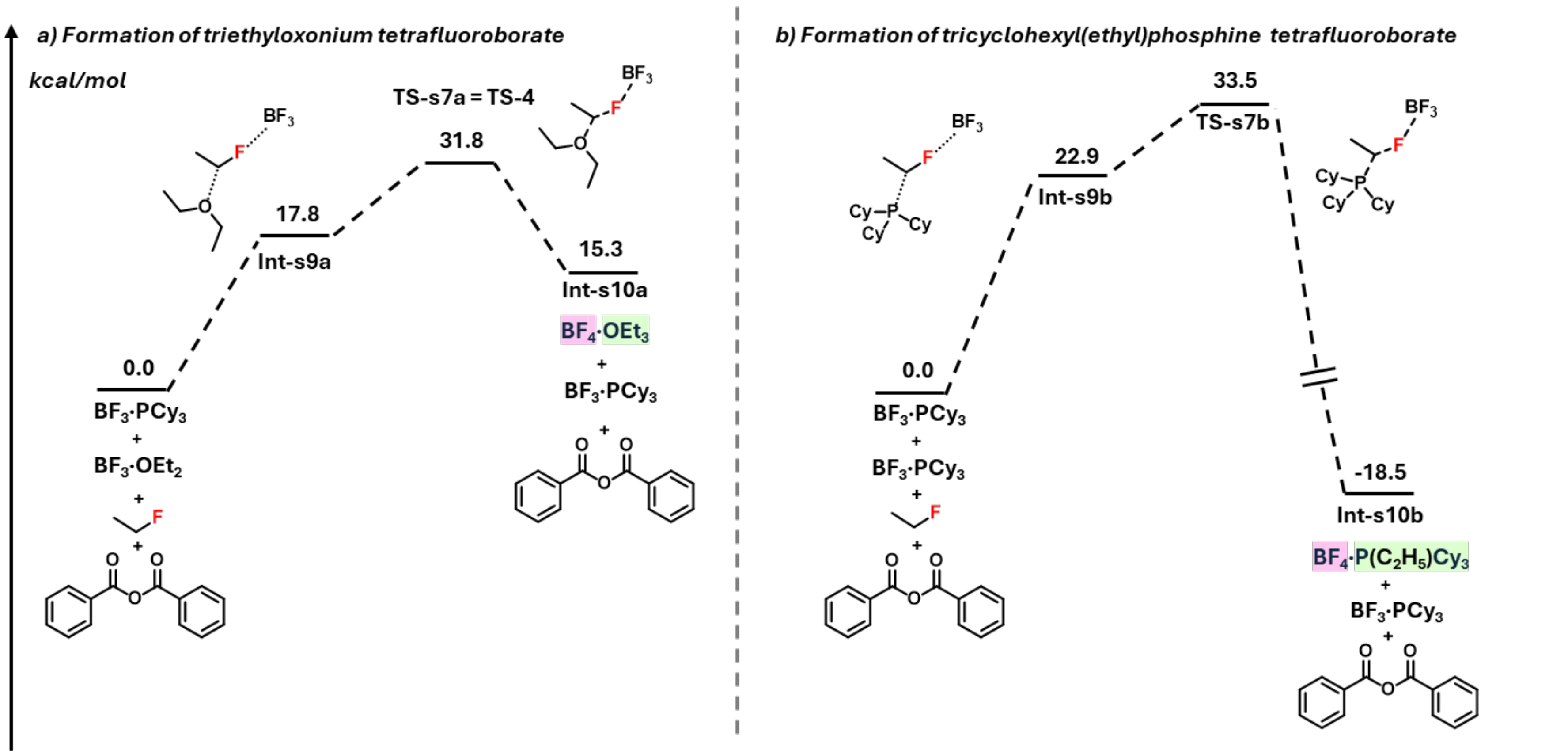

Figure S.6.68 **a**) DFT calculated pathway for the reaction of boron trifluoride diethyl etherate with fluoroethane, generating triethyloxonium tetrafluoroborate. **b**) DFT calculated pathway for the reaction of boron trifluoride tricyclohexylphosphine with fluoroethane, generating tricyclohexyl(ethyl)phosphine tetrafluoroborate. Calculated using the B3LYP hybrid functional. The 6-311++G** basis set was used for optimisation with a PCM solvation model, and single-point corrections were done using def2-QZVPPD with a SMD solvation model. Dispersion was included (GD3BJ). GoodVibes corrections were performed (373.15 K, 0.0901 mol L^{-1} in benzoic anhydride). Energy values reported in kcal mol^{-1}. (Cy = tricyclohexyl)

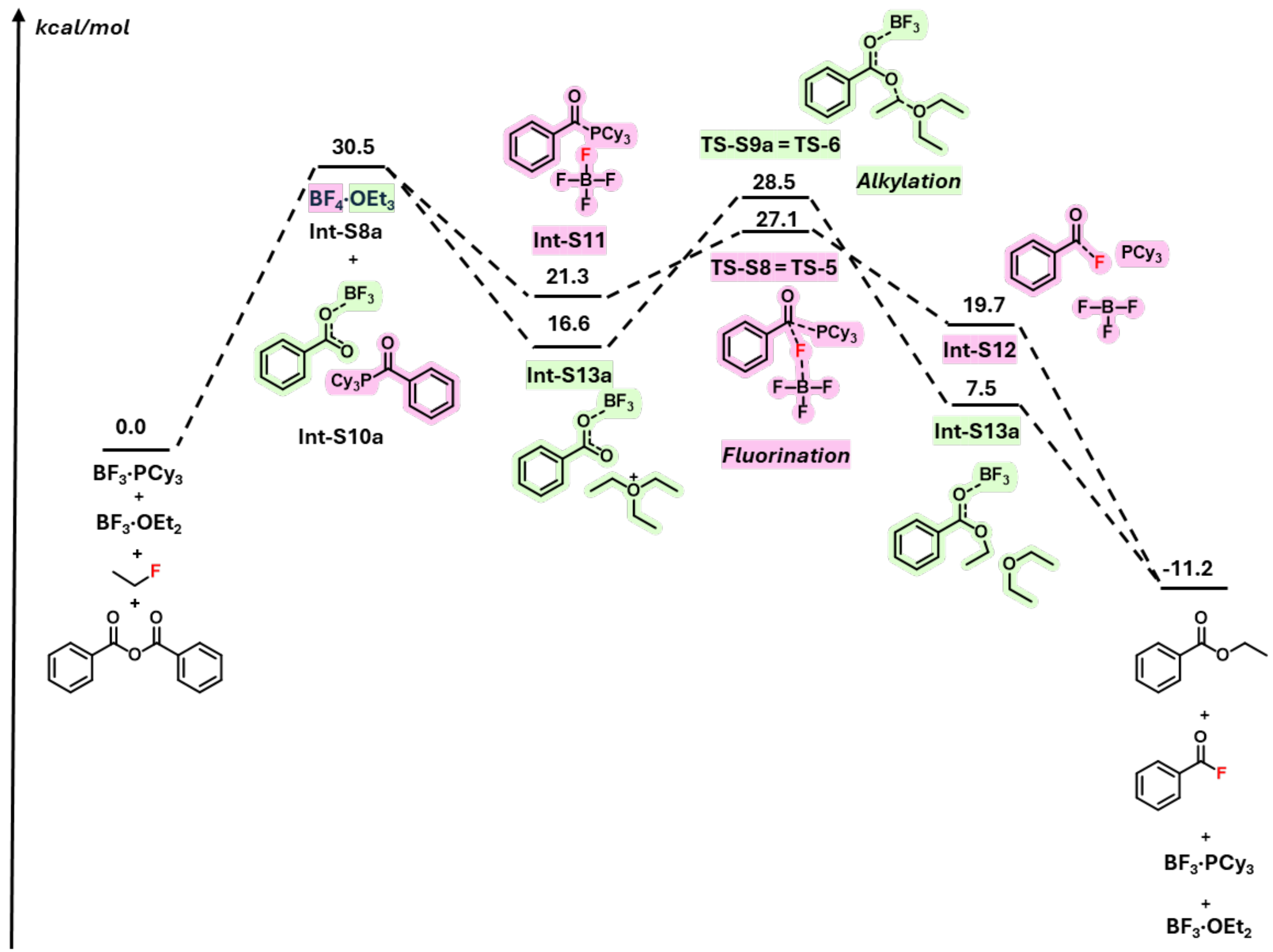

Figure S.6.69 DFT calculated mechanism for the fluoroalkylation of activated benzoic anhydride mediated by $BF_4{\cdot}OEt_3$ generating benzoyl fluoride and ethyl benzoate. Calculated using the B3LYP hybrid functional. The 6-311++G** basis set was used for optimisation with a PCM solvation model, and single-point corrections were done using def2-QZVPPD with a SMD solvation model. Dispersion was included (GD3BJ). GoodVibes corrections were performed (373.15 K, 0.0901 mol L^{-1} in benzoic anhydride). Energy values reported in kcal mol^{-1}. (Cy = cyclohexyl).

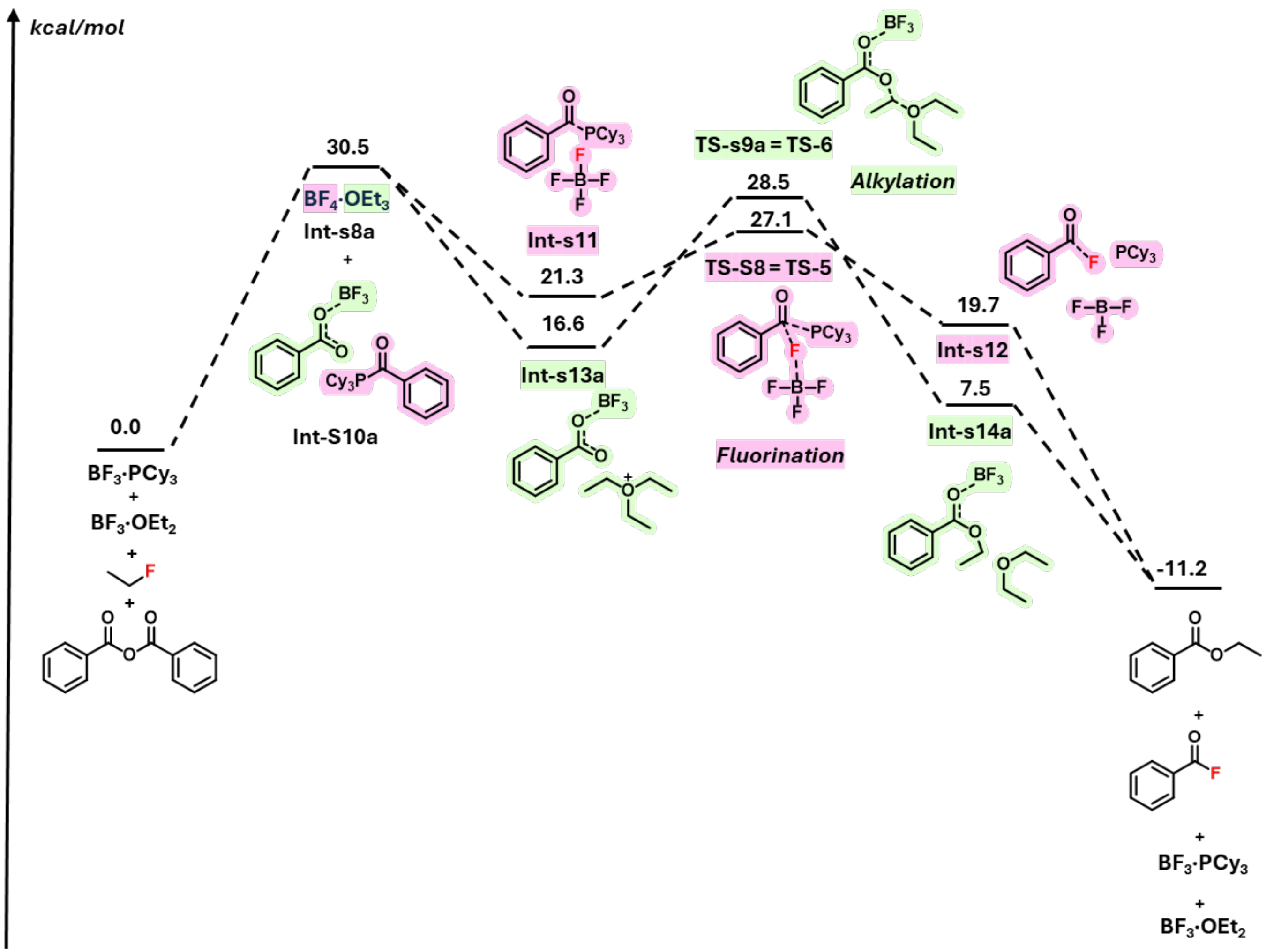

Figure S.6.70 DFT calculated mechanism for the fluoroalkylation of activated benzoic anhydride mediated by $BF_4{\cdot}EtPCy_3$ generating benzoyl fluoride and ethyl benzoate. Calculated using the B3LYP hybrid functional. Calculated using the B3LYP hybrid functional. The 6-311++G** basis set was used for optimisation with a PCM solvation model, and single-point corrections were done using def2-QZVPPD with a SMD solvation model. Dispersion was included (GD3BJ). GoodVibes corrections were performed (373.15 K, 0.0901 mol L^{-1} in benzoic anhydride). Energy values reported in kcal mol^{-1}. (Cy = cyclohexyl)

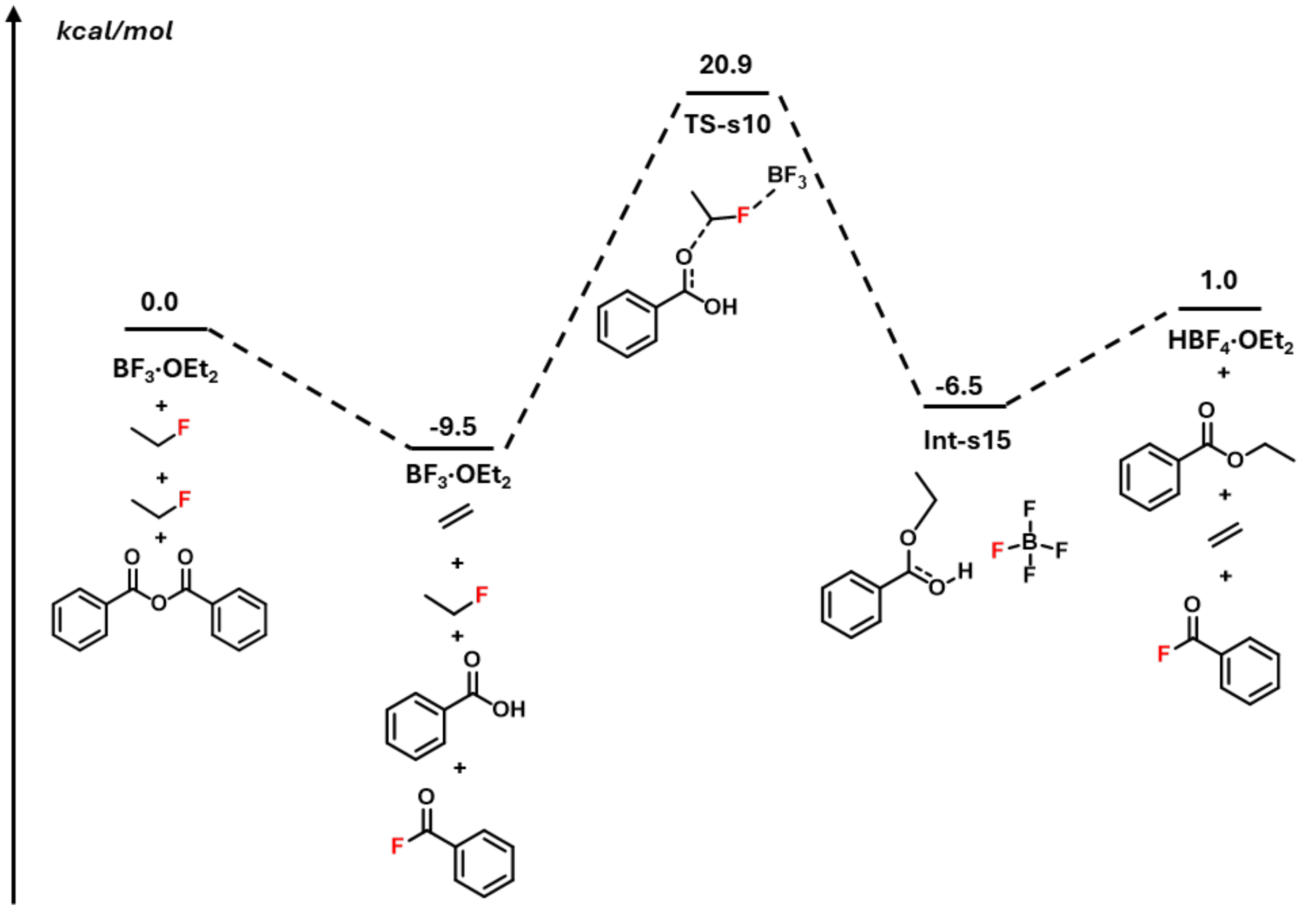

Figure S.6.71 DFT calculated mechanism for the reaction of benzoic acid generated as a byproduct of the shuttle catalysis reaction with fluoroethane, generating benzoic ester and $[BF_4][HOEt_2]$. Calculated using the B3LYP hybrid functional. The 6-311++G** basis set was used for optimisation with a PCM solvation model, and single-point corrections were done using def2-QZVPPD with a SMD solvation model. Dispersion was included (GD3BJ). GoodVibes corrections were performed (373.15 K, 0.0901 mol L^{-1} in benzoic anhydride). Energy values reported in kcal mol^{-1}. (Cy = cyclohexyl)

	B3LYP/ 6311++g** (ΔG kcal mol^{-1})	M062x/ 6311++g** (ΔG kcal mol^{-1})	WB97XD/ 6311++g** (ΔG kcal mol^{-1})	PBE1PBE/ 6311++g** (ΔG kcal mol^{-1})	B3PW91/ 6311++g** (ΔG kcal mol^{-1})
Overall reaction – carboxylic acid product	-9.5	-5.9	-6.5	-3.9	-10.1
Overall reaction – ester product	-11.2	-10.0	-10.3	-8.5	-16.9
BF_3 + OEt_2	-3.6	4.6	-3.9	-2.8	-3.5
BF_3 + PCy_3	7.0	13.8	11.1	8.8	8.5
**Int-s1a**	5.0	5.8	3.5	5.4	4.4
**Int-s1b**	19.1	16.8	18.7	19.8	20.7
**Int-s1c**	18.0	15.7	18.4	19.5	19.3
**Int-s1d**	27.0	42.6	31.8	29.6	29.5
**Int-s1e**	27.8	15.7	-	-	30.6
**TS-s1a** = **TS-1**	29.8	33.4	32.5	33.2	32.0
**TS-s1b**	38.6	40.2	42.9	42.9	41.6
**TS-s1c**	39.4	37.0	43.4	42.9	41.7
**TS-s1d**	43.6	44.5	47.5	46.7	45.6
**TS-s1e**	68.7	55.8	-	-	69.7
**Int-s2a**	2.7	6.0	6.0	6.3	5.6
**Int-s2b**	-10.7	-22.3	-16.8	-5.0	-23.6

Table S.6.5 Functional scope for energies of intermediates and transition states for defluorination steps.

	B3LYP/ 6311++g** (ΔG kcal mol^{-1})	M062x/ 6311++g** (ΔG kcal mol^{-1})	WB97XD/ 6311++g** (ΔG kcal mol^{-1})	PBE1PBE/ 6311++g** (ΔG kcal mol^{-1})	B3PW91/ 6311++g** (ΔG kcal mol^{-1})
**Int-s3**	12.0	12.5	16.8	15.3	16.0
**TS-s2 = TS-2**	22.7	24.1	28.6	27.3	28.4
**Int-s4**	22.6	20.2	17.2	22.8	25.0
**TS-s3 = TS-3**	24.9	25.5	27.3	24.8	31.9
**Int-s5a**	14.6	14.6	13.7	20.1	20.6
**Int-s5b**	-2.8	-0.8	1.5	-3.3	6.8
**TS-s4a**	30.9	28.2	32.8	29.1	34.2
**TS-s4b**	45.7	51.3	48.9	47.0	50.6
**Int-s6a**	23.9	29.7	22.0	25.2	30.3
**Int-s6b**	30.9	36.0	38.6	32.6	34.2
**TS-s5a**	25.5	29.5	31.5	27.9	25.8
**TS-s5b**	37.3	34.0	43.3	39.7	41.1

Table S.6.6 Functional scope for energies of intermediates and transition states for hydrofluorination steps.

	B3LYP/ 6311++g** (ΔG kcal mol^{-1})	M062x/ 6311++g** (ΔG kcal mol^{-1})	WB97XD/ 6311++g** (ΔG kcal mol^{-1})	PBE1PBE/ 6311++g** (ΔG kcal mol^{-1})	B3PW91/ 6311++g** (ΔG kcal mol^{-1})
**Int-s7**	19.6	23.5	27.2	28.3	22.1
**TS-s6**	29.8	36.0	23.9	30.3	30.5
**Int-s8a**	15.1	11.9	14.7	19.2	19.9
**Int-s8b**	9.0	3.8	6.7	22.1	12.8
**Int-s8c**	18.1	19.5	22.9	49.5	25.1
**Int-s9a**	17.8	15.4	17.8	19.9	19.3
**Int-s9b**	22.9	23.7	24.3	32.9	33.0
**TS-s7a** = **TS-4**	31.8	31.9	35.7	36.7	35.0
**TS-s7b**	33.5	35.0	36.2	42.0	41.1
**Int-s10a**	15.3	10.2	16.7	18.2	18.0
**Int-s10b**	-18.5	-24.3	-19.1	-17.4	-18.0
**Int-s11**	16.7	18.2	18.8	15.2	18.1
**TS-s8** = **TS-5**	27.1	26.8	30.2	29.1	27.9
**Int-s13a**	21.3	25.2	27.2	28.2	28.1
**Int-s13b**	-5.6	-1.9	-5.1	-1.7	-2.7
**TS-s9a** = **TS-6**	28.5	29.9	29.6	29.6	26.0
**TS-s9b**	41.1	46.6	51.1	48.3	48.6
**TS-s10**	20.9	28.5	27.4	29.2	31.6
**Int-s15**	-6.5	-5.8	-4.9	-0.6	0.2

Table S.6.7 Functional scope for energies of intermediates and transition states for ester forming steps.

6.2.6 NBO analysis: Wiberg Bond Indices (WBI) and NPA charges

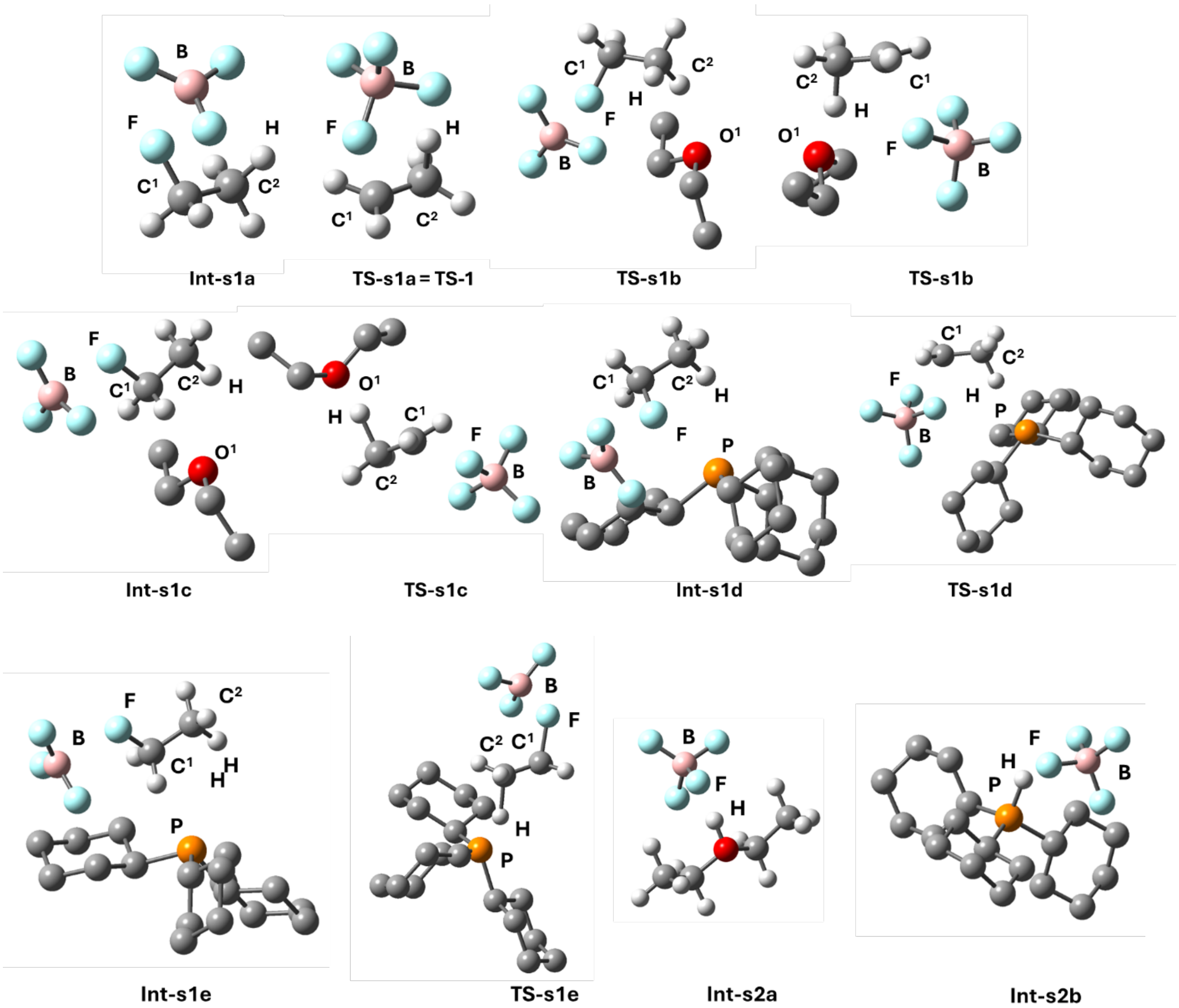

Figure S.6.72 Models showcasing the atoms and bonds relevant to NBO analysis for defluorination steps. Some hydrogens have been omitted for clarity.

	B–F	O–H	P–H	C^1–F	C^2–H	C–C
**Int-s1a**	0.07	-	-	0.78	0.93	1.05
**TS-s1a = TS-1**	0.79	-	-	0.33	0.74	1.21
**Int-s1b**	0.10	0.00	-	0.76	0.93	1.05
**TS-s1b**	0.59	0.23	-	0.16	0.48	1.50
**Int-s1d**	0.08	0.01	-	0.77	0.92	1.05
**TS-s2c**	0.56	0.17	-	0.18	0.57	1.40
**Int-s1d**	0.08	-	0.01	0.77	0.92	1.05
**TS-s1d**	0.50	-	0.21	0.30	0.66	1.27
**Int-s1d**	0.09	-	0.00	0.77	0.92	1.05
**TS-s1d**	0.37	-	0.62	0.44	0.32	1.36
**Int-s2a**	0.52	0.51	-	-	-	-
**Int-s2b**	0.65	-	0.93	-	-	-

Table S.6.8 Wiberg Bond Indices (WBI) calculated using NBO v7.0 on stationary points along the reaction pathway for defluorination steps.

	B	F	O[1]	P	C[1]	C[2]	H
**Int-s1a**	1.42	-0.41	-	-	0.09	-0.62	0.22
**TS-s1a = TS-1**	1.34	-0.54	-	-	0.11	-0.71	0.43
**Int-s1b**	1.42	-0.42	-0.62	-	0.09	-0.64	0.24
**TS-s1b**	1.34	-0.53	-0.61	-	0.11	-0.75	0.41
**Int-s1d**	1.43	-0.42	-0.63	-	0.07	-0.62	0.22
**TS-s2c**	1.33	-0.52	-0.60	-	0.00	-0.65	0.40
**Int-s1d**	1.43	-0.41	-	0.83	0.09	-0.63	0.23
**TS-s1d**	1.35	-0.48	-	0.98	0.09	-0.75	0.29
**Int-s1d**	1.42	-0.41	-	0.83	0.08	-0.62	0.21
**TS-s1d**	1.38	-0.45	-	1.13	-0.02	-0.73	0.11
**Int-s2a**	1.34	-0.56	-0.56	-	-	-	0.54
**Int-s2b**	1.33	-0.54	-	1.35	-	-	0.09

Table S.6.9 Natural population analysis (NPA) charges calculated using NBO v7.0 on stationary points along the reaction pathway for defluorination steps.

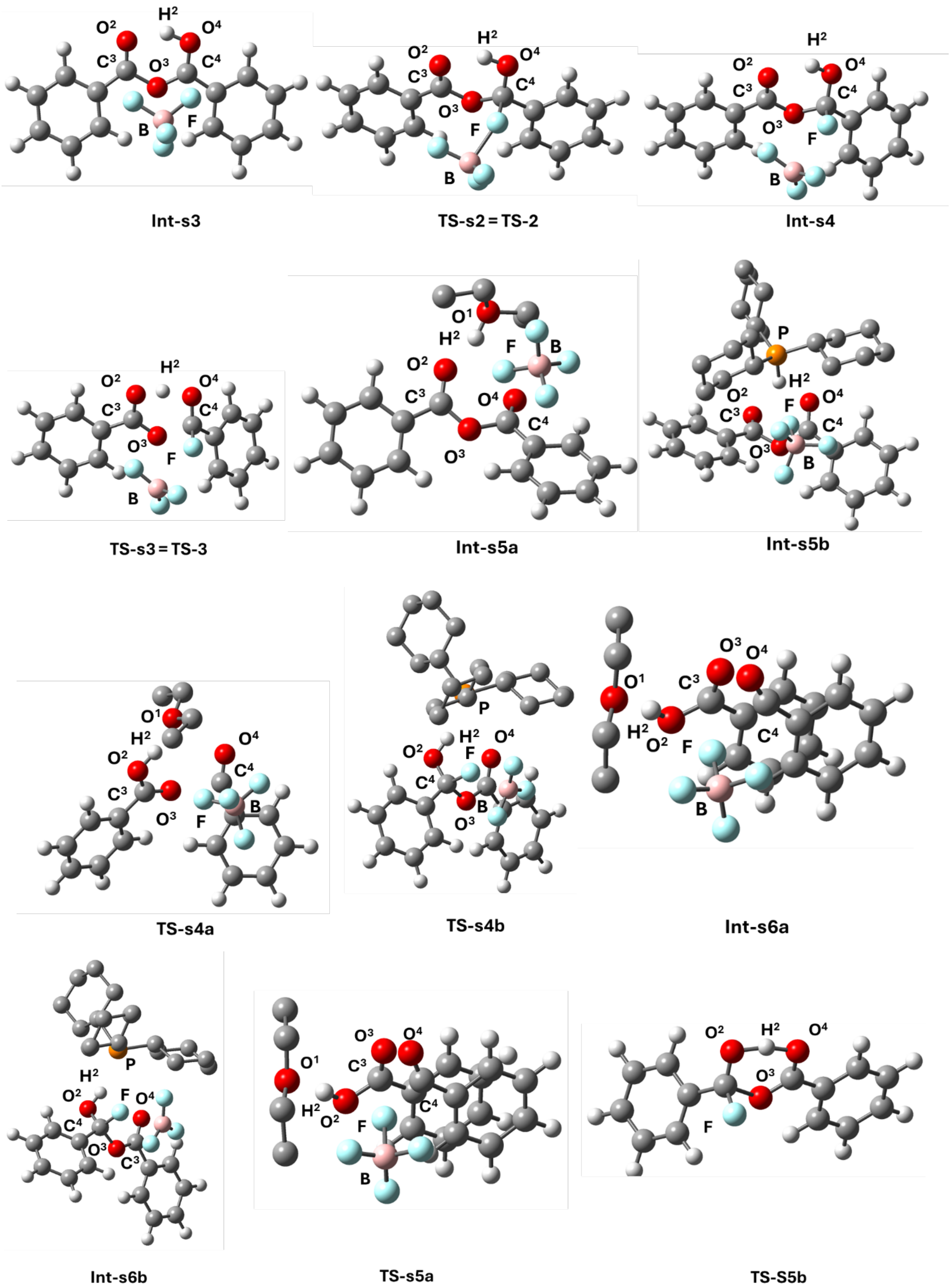

Figure S.6.73 Models showcasing the atoms and bonds relevant to NBO analysis for hydrofluorination steps. Some hydrogens have been omitted for clarity.

	B–F	C^4–F	C^3–O^2	C^3–O^3	C^4–O^3	C^4–O^4	O^2–H^2	O^1–H^2	O^4–H^2	P–H^2
**Int-s3**	0.64	0.03	1.70	0.84	1.30	1.12	0.10	-	0.61	-
**TS-s2** = **TS-2**	0.21	0.54	1.70	0.94	0.93	1.15	0.06	-	0.66	-
**Int-s4**	0.03	0.74	1.69	0.99	0.86	1.07	0.04	-	0.68	-
**TS-s3** = **TS-3**	0.03	0.73	1.37	1.26	0.58	1.36	0.42	-	0.31	-
**Int-s5a**	0.62	0.01	1.62	1.02	0.85	1.77	0.12	0.53	0.00	-
**Int-s5b**	0.62	0.01	1.80	0.89	0.95	1.71	0.01	-	0.01	0.93
**TS-s4a**	0.60	0.04	1.21	1.48	0.20	2.17	0.59	0.11	0.00	-
**TS-s4b**	0.18	0.56	1.75	0.93	0.91	1.15	0.01	-	0.51	0.22
**Int-s6a**	0.57	0.10	1.14	1.62	0.03	2.24	0.61	0.10	0.00	-
**Int-s6b**	0.04	0.72	1.73	0.97	0.86	1.09	0.01	-	0.56	0.16
**TS-s5a**	0.38	0.37	1.12	1.64	0.01	2.10	0.62	0.10	0.00	-
**TS-s5b**	-	0.83	0.50	1.30	1.37	1.01	0.35	-	0.36	-

Table S.6.10 Wiberg Bond Indices (WBI) calculated using NBO v7.0 on stationary points along the reaction pathway for hydrofluorination steps.

	B	F	P	C^3	C^4	H^2	O^1	O^2	O^3	O^4
**Int-s3**	1.33	-0.58	-	0.86	0.91	0.53	-	-0.58	-0.52	-0.59
**TS-s2** = **TS-2**	1.41	-0.46	-	0.84	0.93	0.52	-	-0.60	-0.58	-0.68
**Int-s4**	1.43	-0.43	-	0.83	0.92	0.51	-	-0.62	-0.58	-0.71
**TS-s3** = **TS-3**	1.43	-0.43	-	0.86	0.93	0.50	-	-0.64	-0.57	-0.74
**Int-s5a**	1.33	-0.60	-	0.86	0.84	0.56	-0.58	-0.64	-0.57	-0.58
**Int-s5b**	1.34	-0.61	1.42	0.82	0.84	0.06	-	-0.55	-0.59	-0.62
**TS-s4a**	1.33	-0.59	-	0.85	0.94	0.52	-0.65	-0.66	-0.67	-0.35
**TS-s4b**	1.42	-0.46	0.94	0.83	0.93	0.49	-	-0.58	-0.59	-0.77
**Int-s6a**	1.34	0.57	-	0.81	0.96	0.52	-0.64	-0.69	-0.66	-0.30
**Int-s6b**	1.43	-0.44	0.91	0.82	0.93	0.50	-	-0.59	-0.60	-0.78
**TS-s5a**	1.37	-0.48	-	0.80	0.93	0.51	-0.64	-0.70	-0.65	-0.39
**TS-s5b**	-	-0.39	-	0.84	0.95	0.49	-	-0.66	-0.58	-0.73

Table S.6.11 Natural population analysis (NPA) charges calculated using NBO v7.0 on stationary points along the reaction pathway for hydrofluorination steps.

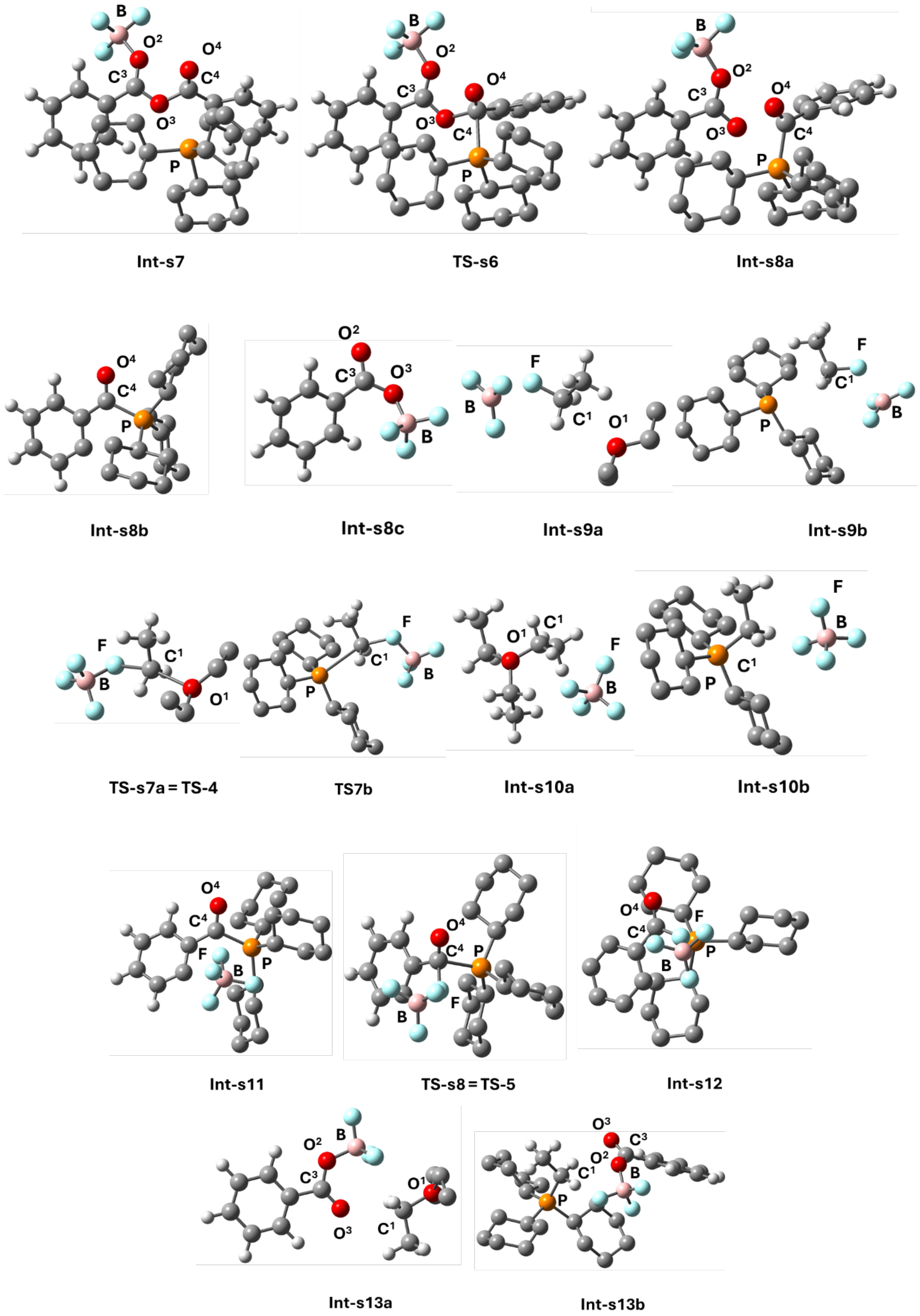

Figure S.6.74 Models showcasing the atoms and bonds relevant to NBO analysis for ester forming steps. Some hydrogens have been omitted for clarity.

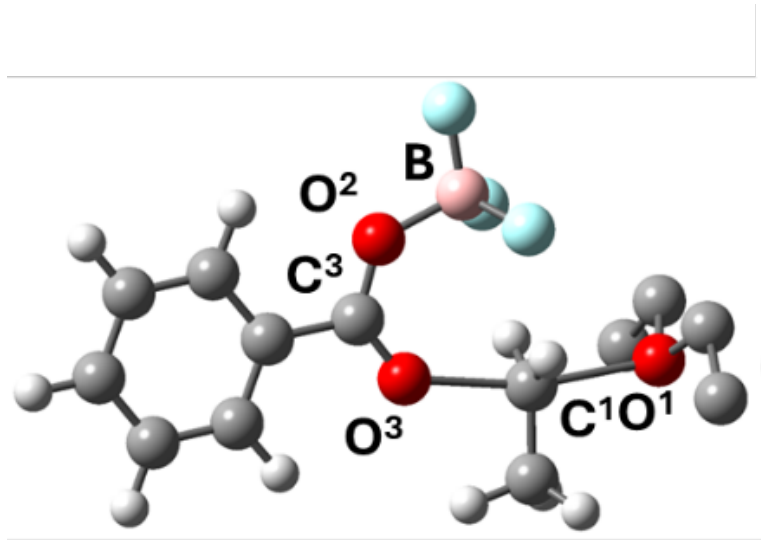

TS-s9a = TS=6

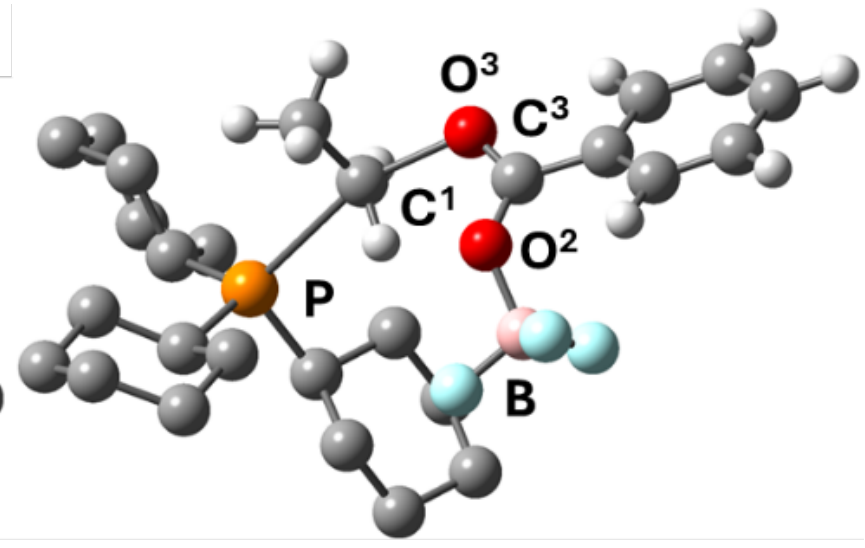

TS-s9b

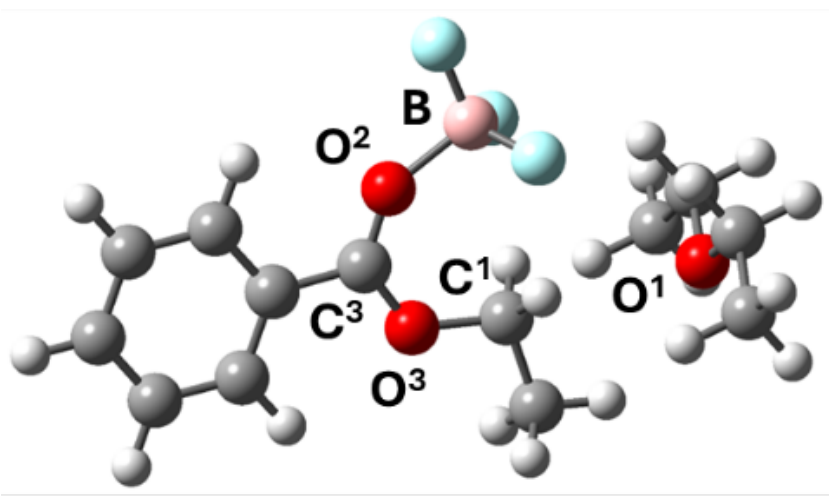

Int-s14a

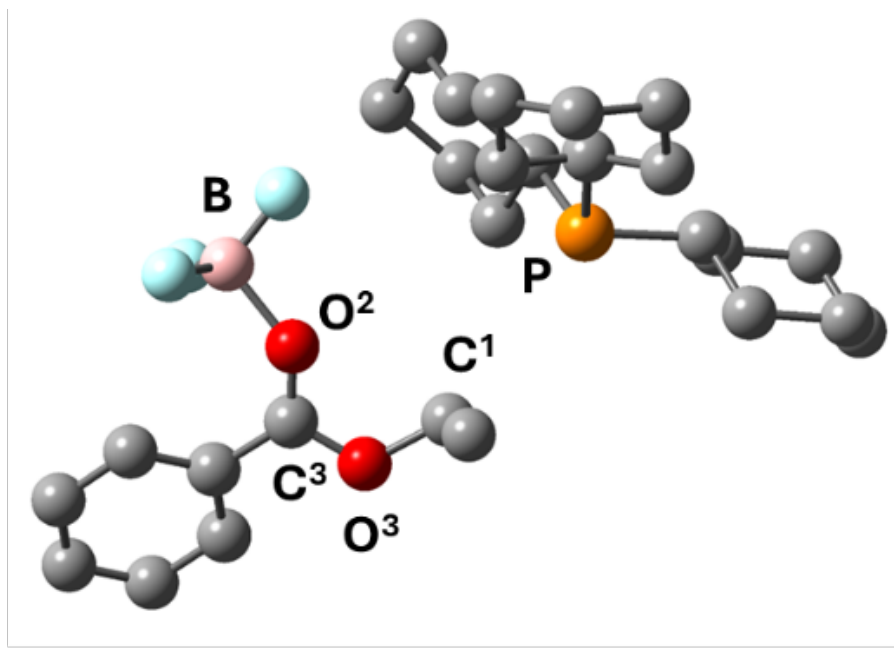

Int-s14b

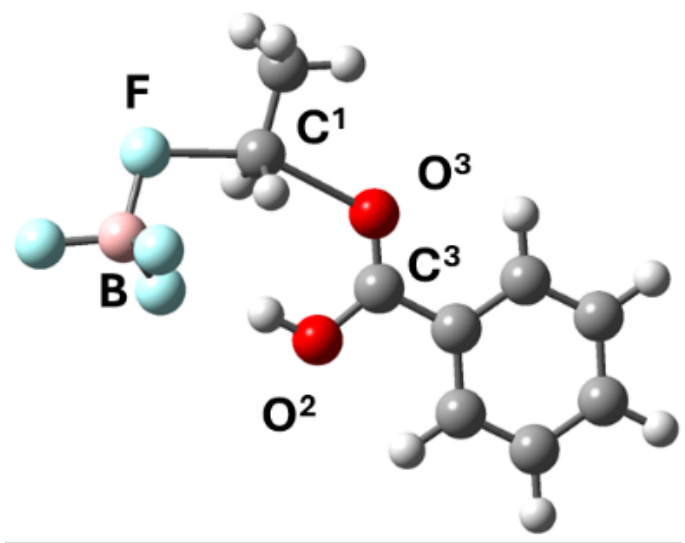

TS-s10

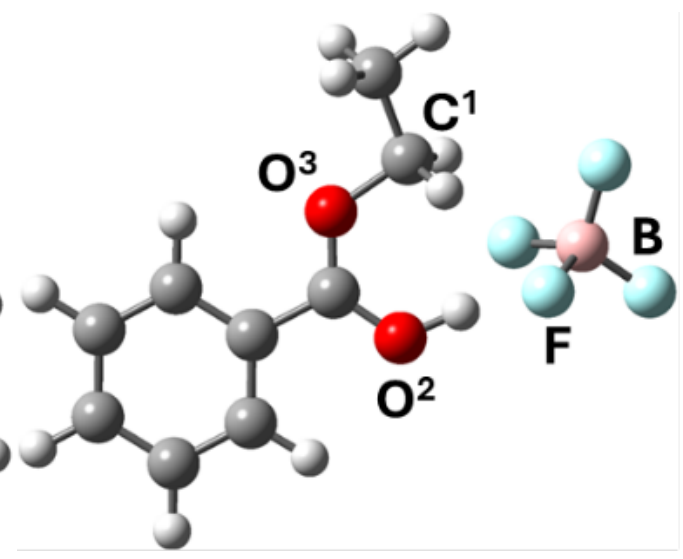

Int-s15

Figure S.6.74 **(Continued)** Models showcasing the atoms and bonds relevant to NBO analysis for ester forming steps. Some hydrogens have been omitted for clarity.

	B–F	B–O^2	C^1–F	C^1–O^2	C^1–O^3	C^3–O^2	C^3–O^3	C^4–O^3	C^4–O^4	C^4–F	P–C^1	P–C^4
**Int-s7**	-	0.43	-	-	-	1.45	1.10	0.76	1.87	-	-	0.02
**TS-s6**	-	0.48	-	-	-	1.41	1.20	0.55	1.61	-	-	0.61
**Int-s8a**	-	0.57	-	-	-	1.22	1.50	0.05	1.85	-	-	0.81
**Int-s8b**	-	-	-	-	-	-	-	-	1.79	-	-	0.83
**Int-s8c**	-	0.61	-	-	-	1.13	1.66	-	-	-	-	-
**Int-s9a**	0.09	-	0.76	0.01	-	-	-	-	-	-	-	-
**Int-s9b**	0.10	-	0.76	-	-	-	-	-	-	-	0.01	-
**TS-s7a** = **TS-4**	0.51	-	0.30	0.23	-	-	-	-	-	-	-	-
**TS-s7b**	0.44	-	0.42	-	-	-	-	-	-	-	0.22	-
**Int-s10a**	0.67	-	0.00	0.73	-	-	-	-	-	-	-	-
**Int-s10b**	0.67	-	0.00	-	-	-	-	-	-	-	0.95	-
**Int-s11**	0.62	-	-	-	-	-	-	-	1.79	0.01	-	0.83
**TS-s8**	0.79	-	-	-	-	-	-	-	1.58	0.52	-	0.67
**Int-s12**	0.00	-	-	-	-	-	-	-	1.86	0.78	-	0.00
**Int-s13a**	-	0.63	-	0.71	0.01	1.12	1.63	-	-	-	-	-
**Int-s13b**	-	0.62	-	-	-	1.11	1.65	-	-	-	0.94	-
**TS-s9a** = **TS-5**	-	0.58	-	0.27	0.31	1.22	1.47	-	-	-	-	-
**TS-s9b**	-	0.53	-	-	-	1.24	1.42	-	-	-	0.35	-

Table S.6.12 Wiberg Bond Indices (WBI) calculated using NBO v7.0 on stationary points along the reaction pathway for ester forming steps.

	B–F	B–O²	C¹–F	C¹–O²	C¹–O³	C³–O²	C³–O³	C⁴–O³	C⁴–O⁴	C⁴–F	P–C¹	P–C⁴
**Int-s14a**	-	0.46	-	0.01	0.79	1.19	1.39	-	-	-	-	-
**Int-s14b**	-	0.46	-	-	0.80	1.18	1.38	-	-	-	0.01	-
**TS-s10**	0.53	-	0.30	0.22	-	1.13	1.56	-	-	-	-	-
**Int-s13**	1.34	-0.57	-	-0.05	0.88	-	-	-0.61	-0.50	-	1.34	-0.57

Table S.6.12 (Continued) Wiberg Bond Indices (WBI) calculated using NBO v7.0 on stationary points along the reaction pathway for ester forming steps.

	B	F	P	C¹	C³	C⁴	O¹	O²	O³	O⁴
**Int-s7**	1.34	-	0.87	-	0.90	0.84	-	-0.59	-0.57	-0.50
**TS-s6**	1.33	-	1.42	-	0.90	0.53	-	-0.59	-0.64	-0.65
**Int-s8a**	1.32	-	1.58	-	0.85	0.40	-	-0.68	-0.74	-0.49
**Int-s8b**	-	-	1.57	-	-	0.34	-	-	-	-0.51
**Int-s8c**	1.32	-	-	-	0.81	-	-	-0.72	-0.64	-
**Int-s9a**	1.42	-0.41	-	0.09	-	-	-	-	-	-
**Int-s9b**	1.42	-0.41	0.82	0.08	-	-	-	-	-	-
**TS-s7a = TS-4**	1.34	-0.48	-	0.12	-	-	-	-	-	-
**TS-s7b**	1.36	-0.44	0.92	0.05	-	-	-	-	-	-
**Int-s10a**	1.33	-0.57	-	-0.02	-	-	-	-	-	-
**Int-s10b**	1.33	-0.58	1.64	-0.75	-	-	-	-	-	-

Table S.6.13 Natural population analysis (NPA) charges calculated using NBO v7.0 on stationary points along the reaction pathway for ester forming steps.

	B	F	P	C^1	C^3	C^4	O^1	O^2	O^3	O^4
**Int-s11**	1.34	-0.59	1.57	-	-	0.38	-	-	-	-0.53
**TS-s8** = **TS-5**	1.41	-0.48	1.45	-	-	0.55	-	-	-	-0.68
**Int-s12**	1.42	-0.47	0.85	-	-	0.90	-	-	-	-0.52
**Int-s13a**	1.32	-	-	-0.03	0.82	-	-0.47	-0.68	-0.71	-
**Int-s13b**	1.32	-	1.70	-	0.81	-	-	-0.73	-0.65	-
**TS-s9a** = **TS-6**	1.32	-	-	0.06	0.86	-	-0.57	-0.68	-0.63	-
**TS-s9b**	1.32	-	1.02	-0.09	0.86	-	-	-0.69	-0.58	-
**Int-s14a**	1.34	-	-	-0.05	0.88	-	-0.63	-0.61	-0.51	-
**Int-s14b**	1.33	-	0.81	-	0.88	-	-	-0.62	-0.50	-
**TS-s10**	1.35	-0.47		-0.67	0.83	-	-	-0.67	-0.61	-
**Int-s15**	0.68	-	0.00	-	-	1.33	1.23	-	-	-

Table S.6.13 (Continued) Natural population analysis (NPA) charges calculated using NBO v7.0 on stationary points along the reaction pathway for ester forming steps.

7 XYZ coordinates

bf3.log

B	0.000000	0.000000	0.000000
F	-0.000000	1.460000	-0.000000
F	-1.264397	-0.730000	-0.000000
F	1.264397	-0.730000	-0.000000

oet2.log

O	-0.000000	0.000000	0.316718
C	0.000000	1.167590	-0.508893
H	0.873651	1.167590	-1.126658
H	-0.873651	1.167590	-1.126658
C	-0.000000	2.424995	0.380226
H	0.873651	2.424995	0.997991
H	-0.000000	3.298646	-0.237538
H	-0.873651	2.424995	0.997991
C	-0.000000	-1.167590	-0.508893
H	0.873651	-1.167590	-1.126658
H	-0.873651	-1.167590	-1.126658
C	-0.000000	-2.424995	0.380226
H	-0.873651	-2.424995	0.997991
H	-0.000000	-3.298646	-0.237538
H	0.873651	-2.424995	0.997991

bf3oet2.log

B	0.114240	1.186810	-0.063854
F	1.436326	1.054477	-0.668979
F	-1.079969	1.146098	-0.902786
F	-0.013637	1.359856	1.380204
O	-0.085803	-0.867408	0.363217
C	-1.255268	-0.907328	-0.458767
H	-1.215828	-0.112453	-1.173973
H	-1.299532	-1.845872	-0.970700
C	-2.507747	-0.743540	0.422180
H	-2.463483	0.195004	0.934113
H	-3.382801	-0.773411	-0.192871
H	-2.547187	-1.538415	1.137386
C	1.077214	-1.019496	-0.454806
H	1.116654	-0.224622	-1.170012
H	1.032949	-1.958040	-0.966738
C	2.336637	-0.976505	0.430409
H	2.297197	-1.771379	1.145615
H	3.206866	-1.090305	-0.181678
H	2.380902	-0.037961	0.942341

hbf4oet2.log

B	-1.315777	-0.487861	-0.009966
F	-1.233027	0.939517	-0.305507
F	-0.222848	-1.187530	-0.678972
F	-1.214530	-0.692957	1.432007
O	1.257452	0.466272	0.306509
C	1.804965	-0.565021	-0.519060
H	1.048176	-0.931758	-1.180630
H	2.618031	-0.169747	-1.091407
C	2.313597	-1.716716	0.367784
H	1.500530	-2.111990	0.940132
H	2.723274	-2.488383	-0.249949
H	3.070385	-1.349979	1.029354
C	0.785151	1.535703	-0.516989
H	0.028362	1.168966	-1.178559
H	1.598217	1.930977	-1.089337
C	0.195522	2.646327	0.372085
H	0.952310	3.013064	1.033655
H	-0.157878	3.446530	-0.244099

H	-0.617545	2.251053	0.944432
F	-2.592703	-1.010474	-0.487391
H	0.527972	0.111634	0.820017

bf3pcy3.log

P	0.051872	-0.213723	0.370930
B	-0.428559	-0.730503	2.167294
F	-0.639069	-2.174323	2.218484
F	-1.656977	-0.045452	2.558668
F	0.646933	-0.362691	3.083532
C	1.583213	-1.067919	-0.116685
C	1.967181	-0.655983	-1.550009
C	2.717352	-0.679999	0.850168
H	1.428742	-2.126063	-0.079094
C	3.262810	-1.379346	-1.962144
H	2.121885	0.402088	-1.587741
H	1.179143	-0.925383	-2.221787
C	4.012928	-1.403261	0.438096
H	2.871973	0.378098	0.812351
H	2.450482	-0.966100	1.846081
C	4.397013	-0.991538	-0.995231
H	3.529746	-1.093301	-2.958078
H	3.108010	-2.437420	-1.924363
H	4.800964	-1.133804	1.109892
H	3.858233	-2.461340	0.475874
H	5.297254	-1.494092	-1.281502
H	4.551727	0.066530	-1.033023
C	0.314384	1.586194	0.307064
C	0.696239	1.998659	-1.126753
C	-0.980671	2.309205	0.721426
H	1.103301	1.855502	0.977831
C	0.918577	3.521564	-1.180915
H	-0.092840	1.729371	-1.797439
H	1.596054	1.496363	-1.414730
C	-0.758164	3.832115	0.667380
H	-1.769723	2.039950	0.050763
H	-1.245958	2.022646	1.717626
C	-0.376363	4.244569	-0.766398
H	1.183794	3.808064	-2.177137
H	1.707668	3.790835	-0.510297
H	-1.657917	4.334526	0.955351
H	0.030957	4.101321	1.338014
H	-0.221733	5.302671	-0.803999
H	-1.165502	3.975396	-1.437029
C	-1.288644	-0.672621	-0.771381
C	-1.509459	-2.195782	-0.717967
C	-2.584936	0.048780	-0.358072
H	-1.022572	-0.385519	-1.767274
C	-2.643664	-2.584251	-1.684471
H	-1.775427	-2.482924	0.277875
H	-0.608792	-2.697069	-1.005177
C	-3.719235	-0.339822	-1.324526
H	-2.850875	-0.238354	0.637774
H	-2.431622	1.107099	-0.395235
C	-3.939951	-1.862983	-1.271065
H	-2.797008	-3.642562	-1.647459
H	-2.377691	-2.297004	-2.680292
H	-4.619916	0.161376	-1.037294
H	-3.453342	-0.052734	-2.320401
H	-4.728060	-2.132958	-1.942565
H	-4.205884	-2.150154	-0.275194

ethylfluoride.log

C	-1.103617	-0.513421	-0.000000

H -2.062807 -0.039233 -0.000000
H -1.007421 -1.123650 0.873651
H -1.007421 -1.123650 -0.873651
C 0.000000 0.560651 0.000000
H -0.096196 1.170880 -0.873651
H -0.096196 1.170880 0.873651
F 1.210193 -0.037623 0.000000

ethene.log
C 0.000000 0.677600 -0.000000
H 0.924521 1.216266 -0.000000
H -0.927705 1.210764 -0.000000
C -0.000000 -0.677600 -0.000000
H -0.924521 -1.216266 -0.000000
H 0.927705 -1.210764 -0.000000

benzoicanhydride.log
O -0.000000 0.000000 0.068298
C 0.000000 -1.167590 -0.757313
C -0.000000 1.167590 -0.757313
O 0.000000 -1.052130 -2.010405
O -0.000000 1.052130 -2.010405
C -0.000000 -2.566292 -0.112928
C -0.000000 -2.694233 1.276353
C -0.000111 -3.705226 -0.918147
C 0.000571 -3.960862 1.860197
H 0.000865 -1.796125 1.910895
C -0.000536 -4.972314 -0.334282
H -0.000291 -3.604571 -2.013134
C -0.000057 -5.100302 1.054658
H 0.001205 -4.061786 2.955236
H -0.001039 -5.870135 -0.969414
H 0.000334 -6.098888 1.515225
C 0.000000 2.566292 -0.112928
C 0.000000 2.694233 1.276353
C 0.000111 3.705226 -0.918147
C -0.000571 3.960862 1.860197
H -0.000865 1.796125 1.910895
C 0.000536 4.972314 -0.334282
H 0.000291 3.604571 -2.013134
C 0.000057 5.100302 1.054658
H -0.001205 4.061786 2.955236
H 0.001039 5.870135 -0.969414
H -0.000334 6.098888 1.515225

benzoylfluoride.log
C -2.536436 -0.004369 -0.128242
C -1.839390 -1.209808 -0.041552
C -0.454613 -1.204436 0.124538
C 0.233895 0.006780 0.202823
C -0.462994 1.211889 0.115671
C -1.848311 1.206345 -0.049481
H -3.628266 -0.008821 -0.258742
H -2.382278 -2.164140 -0.102909
H 0.094799 -2.154534 0.193506
H 0.079345 2.166635 0.177326
H -2.397514 2.156511 -0.117966
C 1.762936 0.012360 0.386134
O 2.309275 1.195523 -0.202542
F 2.292132 -1.074711 -0.214470

benzoicacid.log
C 2.611016 -0.084919 0.009208
C 1.854883 -1.264772 -0.002197

C	0.455015	-1.199829	-0.010816
C	-0.188720	0.044968	-0.008031
C	0.567413	1.224821	0.003376
C	1.967281	1.159878	0.011996
H	3.679846	-0.134504	0.015789
H	2.346389	-2.215202	-0.004325
H	-0.122309	-2.100674	-0.019524
H	0.075907	2.175252	0.005504
H	2.544605	2.060723	0.020704
C	-1.727036	0.116334	-0.017506
O	-2.305084	1.234111	-0.015005
O	-2.498600	-1.087599	-0.029148
H	-3.334094	-0.936576	0.418895

benzoicester.log

C	2.465527	-1.535567	-0.000174
C	1.119068	-1.170164	0.000429
C	0.762198	0.178119	0.000113
C	1.752017	1.161722	0.000392
C	3.098132	0.796332	0.000268
C	3.454943	-0.552407	-0.000412
H	2.746676	-2.598627	-0.000374
H	0.339270	-1.945507	-0.000161
H	1.470396	2.224730	0.000201
H	3.878461	1.571290	0.000496
H	4.516211	-0.840225	-0.000707
C	-0.724016	0.581564	-0.000109
O	-1.050552	1.796860	-0.000395
O	-1.736483	-0.428292	0.000009
C	-3.026385	0.189004	-0.000050
H	-3.130385	0.797838	-0.873781
H	-3.130354	0.798071	0.873522
C	-4.116187	-0.899081	0.000115
H	-4.012192	-1.507908	0.873851
H	-5.081358	-0.437188	0.000060
H	-4.012212	-1.508155	-0.873451

int-s1a.log

C	-1.411717	1.294842	-0.112727
H	-2.166456	2.069983	0.047569
H	-1.039672	1.383915	-1.135081
H	-0.589458	1.467110	0.583773
C	-2.043697	-0.052794	0.115767
H	-2.390091	-0.194514	1.139101
H	-2.848226	-0.271414	-0.585307
F	-1.062930	-1.083796	-0.107292
B	1.159936	-0.041276	0.020548
F	1.742958	1.133446	-0.174830
F	0.989686	-0.868076	-0.998665
F	0.993254	-0.481684	1.261783

ts-s1a.log

C	-2.090556	0.653636	-0.034649
H	-2.693844	0.945743	0.825795
H	-2.574984	0.947422	-0.966482
H	-0.886790	1.153242	0.069603
C	-1.791548	-0.775791	0.022808
H	-1.790178	-1.242802	1.001747
H	-2.117531	-1.452848	-0.759314
F	-0.188446	-0.962540	-0.323767
B	0.900578	0.007839	0.005270
F	0.234849	1.314391	0.117538
F	1.766652	0.016420	-1.021447
F	1.392841	-0.330161	1.213603

int-s1b.log

O	2.131156	0.314859	0.161969
C	3.537911	0.108996	0.257330
H	3.892119	0.463104	1.236585
H	4.063742	0.686444	-0.513549
C	3.821824	-1.371325	0.088162
H	3.470769	-1.721774	-0.885441
H	4.896933	-1.558346	0.153324
H	3.321485	-1.952788	0.866176
C	1.710248	1.643747	0.462912
H	0.627469	1.579797	0.590233
H	2.142720	1.954746	1.424746
C	2.048990	2.657792	-0.624677
H	1.617376	3.629932	-0.369644
H	3.126920	2.792924	-0.738132
H	1.636031	2.340662	-1.585667
C	0.877018	-1.609403	-0.393817
H	1.736090	-2.253184	-0.183913
H	0.987855	-0.690123	0.185311
H	0.885887	-1.361821	-1.457478
C	-0.393834	-2.333164	-0.019006
H	-0.536174	-3.245571	-0.601396
H	-0.433951	-2.572193	1.045503
F	-1.514590	-1.505934	-0.287676
B	-3.264567	0.573840	0.109414
F	-2.667618	1.406012	-0.720658
F	-2.971411	0.601110	1.394402
F	-4.154673	-0.285603	-0.345502

ts-s1b.log

O	1.763589	0.143763	0.152878
C	1.817446	0.509620	-1.253936
H	0.937238	0.089203	-1.748250
H	2.713115	0.054100	-1.682636
C	1.867862	2.019117	-1.388946
H	2.731958	2.431642	-0.863081
H	1.957536	2.274013	-2.447692
H	0.961578	2.487546	-1.004185
C	1.824156	-1.280691	0.422453
H	1.355024	-1.393831	1.402294
H	1.198329	-1.796477	-0.309539
C	3.250302	-1.802040	0.438926
H	3.244249	-2.861031	0.711588
H	3.730400	-1.714877	-0.538377
H	3.851405	-1.262203	1.173924
C	0.340426	1.343631	1.830814
H	0.631592	0.909389	2.793901
H	0.589549	2.407700	1.831383
H	1.055361	0.729250	0.929570
C	-1.090051	1.100360	1.627075
H	-1.484647	0.199441	2.084717
H	-1.799954	1.921214	1.659911
F	-1.292933	0.740879	0.067209
B	-2.119386	-0.517977	-0.413077
F	-3.405739	-0.242652	-0.051166
F	-1.560945	-1.567579	0.270851
F	-1.878755	-0.527902	-1.754613

int-s1c.log

O	2.807268	0.005131	0.260926
C	2.719513	1.233481	-0.455569
H	1.958573	1.145092	-1.247082
H	3.677843	1.461556	-0.942922

C	2.357418	2.335298	0.524016
H	3.115587	2.423931	1.301625
H	2.277491	3.294449	0.010238
H	1.398790	2.128448	1.001576
C	3.240084	-1.107219	-0.519829
H	2.928006	-1.991889	0.041155
H	2.706246	-1.117637	-1.480748
C	4.749360	-1.134415	-0.736435
H	5.036458	-2.050879	-1.255463
H	5.089041	-0.292432	-1.339128
H	5.269862	-1.105920	0.221709
C	0.031679	-0.964943	1.800283
H	1.101143	-0.756495	1.663975
H	-0.114267	-2.045341	1.815389
H	-0.277898	-0.548453	2.756352
C	-0.719545	-0.331854	0.666230
H	-0.618340	0.749837	0.629774
H	-0.493351	-0.769228	-0.303341
F	-2.145045	-0.574938	0.870690
B	-3.385677	0.096596	-0.552770
F	-2.998197	1.372967	-0.481724
F	-2.887249	-0.660555	-1.532999
F	-4.509065	-0.274268	0.047495

ts-s1c.log

O	2.633155	0.011420	-0.078481
C	3.488957	1.139164	-0.376012
H	3.667658	1.157798	-1.457113
H	4.449082	1.004400	0.130240
C	2.814972	2.417648	0.087135
H	2.636474	2.403848	1.164302
H	3.465180	3.266443	-0.138183
H	1.862905	2.574261	-0.424457
C	3.091801	-1.266583	-0.568909
H	2.219747	-1.919625	-0.487791
H	3.338542	-1.162779	-1.630912
C	4.259635	-1.836028	0.222856
H	4.492795	-2.837689	-0.148806
H	5.161543	-1.228432	0.122894
H	4.006810	-1.915969	1.282681
C	0.382356	-0.112862	1.055441
H	1.457795	-0.015891	0.480201
H	0.365606	-1.060727	1.600028
H	0.326373	0.721239	1.760231
C	-0.670893	-0.043650	0.042884
H	-0.766700	0.903560	-0.481059
H	-0.742113	-0.892679	-0.631655
F	-2.086819	-0.121409	0.741619
B	-3.506652	-0.063845	-0.181539
F	-3.398825	1.141574	-0.803544
F	-3.368354	-1.147463	-0.992895
F	-4.443185	-0.156704	0.789772

int-s1d.log

C	-2.011633	-0.516704	3.441350
H	-2.496361	-1.469811	3.661927
H	-1.905659	0.040924	4.376662
H	-1.015301	-0.692090	3.033148
C	-2.836081	0.298398	2.483359
H	-3.840885	0.509693	2.849535
H	-2.342838	1.221732	2.182148
F	-3.018107	-0.455543	1.269484
B	-4.292407	0.231452	-0.376491
F	-3.535725	-0.333313	-1.305943

F	-4.134530	1.519420	-0.094249
F	-5.373301	-0.398696	0.061136
P	0.914082	0.011436	0.686495
C	0.746387	1.532636	-0.400120
C	-0.731595	1.882050	-0.638544
C	1.471040	2.722496	0.254634
H	1.209566	1.338729	-1.373334
C	-0.887051	3.167371	-1.460661
H	-1.230667	2.013407	0.329333
H	-1.244418	1.063217	-1.144943
C	1.325104	4.004680	-0.574017
H	1.048228	2.885898	1.253891
H	2.529762	2.494336	0.402545
C	-0.148947	4.345221	-0.816691
H	-1.949480	3.401286	-1.580839
H	-0.485721	2.997294	-2.467871
H	1.830446	4.834494	-0.069336
H	1.829157	3.869357	-1.539477
H	-0.236594	5.238545	-1.443126
H	-0.624080	4.585769	0.143190
C	-0.075273	-1.273824	-0.258451
C	0.275308	-1.504466	-1.735199
C	-0.084430	-2.610480	0.504115
H	-1.096858	-0.884674	-0.210739
C	-0.698064	-2.497506	-2.386607
H	1.292173	-1.905805	-1.809574
H	0.265831	-0.560001	-2.286092
C	-1.053116	-3.610042	-0.141604
H	0.924373	-3.042304	0.508051
H	-0.357976	-2.444356	1.549771
C	-0.730589	-3.826998	-1.624413
H	-0.417050	-2.665942	-3.431570
H	-1.702826	-2.059385	-2.394377
H	-1.022865	-4.562916	0.397025
H	-2.075018	-3.223648	-0.046006
H	-1.460535	-4.506368	-2.075947
H	0.249185	-4.314893	-1.711295
C	2.699294	-0.547415	0.412919
C	3.402515	-0.087240	-0.874840
C	3.559307	-0.201966	1.643678
H	2.621579	-1.641599	0.373997
C	4.810820	-0.688012	-0.987208
H	3.488517	1.004416	-0.875869
H	2.814368	-0.353834	-1.754245
C	4.973351	-0.785507	1.532408
H	3.624041	0.888511	1.745947
H	3.068917	-0.566717	2.550658
C	5.664952	-0.345385	0.237797
H	5.295706	-0.330284	-1.901508
H	4.730825	-1.778802	-1.079331
H	5.569829	-0.491881	2.402307
H	4.908912	-1.881196	1.550659
H	6.652656	-0.810126	0.156304
H	5.829045	0.739577	0.268115

ts-s1d.log

C	-1.078460	0.960536	3.084871
H	-0.622528	0.400359	3.900507
H	-0.819736	2.019515	3.094921
H	-0.520908	0.570585	2.134712
C	-2.474058	0.718659	2.920688
H	-2.959811	-0.111415	3.415533
H	-3.128159	1.493317	2.542663
F	-2.634824	-0.248762	1.234629

B	-3.847072	0.042682	0.384820
F	-3.589635	-0.524424	-0.847630
F	-3.945916	1.431169	0.331292
F	-4.926981	-0.527952	1.036569
P	0.654086	0.010272	0.387399
C	0.618815	1.427909	-0.823430
C	-0.818362	1.712253	-1.294559
C	1.227399	2.685181	-0.174468
H	1.222786	1.159946	-1.697350
C	-0.866188	2.917831	-2.240976
H	-1.457942	1.908844	-0.427643
H	-1.242708	0.839542	-1.793301
C	1.181451	3.888094	-1.124333
H	0.662039	2.922225	0.735534
H	2.257531	2.498762	0.139850
C	-0.247419	4.165217	-1.602447
H	-1.902769	3.111613	-2.531473
H	-0.319905	2.674662	-3.161390
H	1.597251	4.769886	-0.626530
H	1.821764	3.684765	-1.991987
H	-0.256442	5.000255	-2.309739
H	-0.860568	4.471604	-0.745353
C	-0.204271	-1.390877	-0.483277
C	0.384900	-1.819751	-1.835150
C	-0.348298	-2.596903	0.461973
H	-1.212931	-1.006811	-0.655492
C	-0.476319	-2.915469	-2.479032
H	1.399887	-2.205796	-1.687635
H	0.464118	-0.962483	-2.509537
C	-1.200003	-3.697885	-0.183576
H	0.642026	-3.003883	0.703394
H	-0.802649	-2.276262	1.403178
C	-0.635220	-4.120169	-1.544574
H	-0.032429	-3.226960	-3.430116
H	-1.465391	-2.502374	-2.709146
H	-1.262961	-4.561279	0.486465
H	-2.220757	-3.321463	-0.315177
H	-1.282116	-4.873275	-2.004863
H	0.344827	-4.593398	-1.398381
C	2.449128	-0.524131	0.519099
C	3.350720	-0.199149	-0.684313
C	3.076845	-0.002473	1.825901
H	2.385325	-1.616236	0.603352
C	4.766002	-0.757056	-0.480340
H	3.416410	0.886318	-0.810989
H	2.922254	-0.596660	-1.605920
C	4.497531	-0.544870	2.023916
H	3.106637	1.093593	1.802904
H	2.445219	-0.277840	2.675558
C	5.391497	-0.236746	0.818024
H	5.393575	-0.494378	-1.337803
H	4.720117	-1.852912	-0.447385
H	4.932260	-0.128241	2.937924
H	4.447135	-1.631880	2.166258
H	6.385761	-0.670475	0.961835
H	5.529934	0.849252	0.739841

int-s1e.log

C	-1.574599	-1.542442	-1.100628
H	-0.641235	-0.938448	-0.751076
H	-1.542510	-1.735914	-2.161534
H	-1.639787	-2.527011	-0.538828
C	-2.820903	-0.768546	-0.810793
H	-3.016197	-0.776579	0.219442

H	-2.632203	0.269749	-1.119951
F	-3.823476	-1.352770	-1.515316
B	-5.790738	-0.766896	-0.587454
F	-6.641228	0.049832	-1.401916
F	-5.698502	-0.028611	0.638857
F	-6.544676	-1.924842	-0.277969
P	1.081665	0.176448	-0.105836
C	0.935048	1.941996	0.334605
C	0.368655	2.764218	-0.849480
C	0.063210	2.193025	1.612074
H	1.875957	2.172507	0.525830
C	0.312087	4.288421	-0.482410
H	-0.629661	2.419391	-1.096150
H	1.002509	2.622671	-1.682127
C	0.014521	3.729665	1.939165
H	-0.943134	1.831921	1.452508
H	0.497051	1.674193	2.425912
C	-0.595870	4.484862	0.747224
H	-0.099100	4.828565	-1.309063
H	1.291321	4.652989	-0.255943
H	-0.601771	3.880078	2.801756
H	0.999956	4.103032	2.125577
H	-0.648369	5.542964	0.983994
H	-1.568715	4.125468	0.544306
C	2.198481	0.010361	-1.485507
C	3.590519	0.606253	-1.157096
C	2.345537	-1.475962	-1.821498
H	1.757487	0.477804	-2.317638
C	4.514464	0.445125	-2.391452
H	4.024741	0.085919	-0.315644
H	3.475313	1.633854	-0.921106
C	3.245021	-1.629299	-3.038094
H	2.756324	-1.991244	-0.998922
H	1.367490	-1.883216	-2.050386
C	4.625707	-1.076909	-2.734304
H	5.486759	0.825405	-2.180446
H	4.092044	0.951624	-3.228548
H	3.307667	-2.673470	-3.278702
H	2.805708	-1.111402	-3.873046
H	5.258589	-1.193652	-3.587408
H	5.049267	-1.587210	-1.897800
C	1.799262	-0.625240	1.377463
C	0.929316	-0.434023	2.632168
C	1.964283	-2.139257	1.091422
H	2.710505	-0.233619	1.568927
C	1.567858	-1.151732	3.844218
H	-0.058622	-0.838444	2.475136
H	0.874885	0.597634	2.828889
C	2.585109	-2.854546	2.289201
H	1.010643	-2.576529	0.889345
H	2.606509	-2.258243	0.251004
C	1.662713	-2.677413	3.512486
H	0.942280	-1.027585	4.706807
H	2.537273	-0.773364	4.037404
H	2.662895	-3.904858	2.057750
H	3.550826	-2.465463	2.490500
H	2.094510	-3.182953	4.351419
H	0.699938	-3.052619	3.307130

ts1e.log

C	-1.195812	-0.381278	-1.915478
H	-0.451546	-0.530305	-0.626067
H	-1.564172	0.644912	-1.946027
H	-0.458606	-0.571097	-2.699497

C	-2.213183	-1.377010	-1.806732
H	-1.874606	-2.409335	-1.794955
H	-2.979495	-1.198540	-1.059322
F	-3.191553	-1.519013	-3.140852
B	-4.526565	-0.553539	-3.439109
F	-4.965607	-0.155053	-2.198391
F	-5.382044	-1.396317	-4.091002
F	-4.047495	0.470267	-4.208696
P	0.588024	-0.220813	0.325173
C	0.263341	1.608320	0.627485
C	0.671558	2.552791	-0.520821
C	-1.223227	1.833906	0.996569
H	0.874985	1.869752	1.501528
C	0.398177	4.020249	-0.149875
H	0.109517	2.297343	-1.427189
H	1.730639	2.437721	-0.761353
C	-1.490682	3.299591	1.370959
H	-1.849152	1.563914	0.138766
H	-1.525965	1.181853	1.818678
C	-1.064876	4.251409	0.246706
H	0.669767	4.663380	-0.992753
H	1.051059	4.306365	0.685351
H	-2.553629	3.430769	1.597605
H	-0.940956	3.546566	2.288602
H	-1.213701	5.292034	0.552312
H	-1.707161	4.086514	-0.627236
C	2.035442	-0.293407	-0.856654
C	3.397864	0.156556	-0.293824
C	2.157711	-1.670422	-1.549748
H	1.737964	0.420094	-1.637698
C	4.465766	0.189792	-1.399719
H	3.722150	-0.538593	0.488735
H	3.320483	1.141627	0.176976
C	3.223398	-1.628874	-2.656845
H	2.436050	-2.438785	-0.822044
H	1.194073	-1.969177	-1.971140
C	4.581891	-1.162021	-2.118632
H	5.431188	0.476173	-0.970604
H	4.203493	0.967414	-2.128541
H	3.316305	-2.619099	-3.114261
H	2.891491	-0.947192	-3.449909
H	5.308634	-1.094148	-2.934265
H	4.969500	-1.912345	-1.417046
C	1.201540	-0.831594	2.012068
C	0.170841	-0.563974	3.132067
C	1.574505	-2.328334	1.976801
H	2.101119	-0.243124	2.219799
C	0.659796	-1.077678	4.495740
H	-0.775583	-1.058629	2.881336
H	-0.041989	0.503220	3.213004
C	2.052670	-2.835916	3.347151
H	0.700059	-2.910475	1.661193
H	2.355802	-2.512072	1.237793
C	1.024239	-2.564374	4.449462
H	-0.113804	-0.895013	5.247850
H	1.539269	-0.498068	4.804754
H	2.269156	-3.906366	3.279815
H	2.998404	-2.341592	3.604483
H	1.411178	-2.891222	5.419583
H	0.119403	-3.155215	4.257061

int-s2a.log

B	-1.315777	-0.487861	-0.009966
F	-1.233027	0.939517	-0.305507

F	-0.222848	-1.187530	-0.678972
F	-1.214530	-0.692957	1.432007
O	1.257452	0.466272	0.306509
C	1.804965	-0.565021	-0.519060
H	1.048176	-0.931758	-1.180630
H	2.618031	-0.169747	-1.091407
C	2.313597	-1.716716	0.367784
H	1.500530	-2.111990	0.940132
H	2.723274	-2.488383	-0.249949
H	3.070385	-1.349979	1.029354
C	0.785151	1.535703	-0.516989
H	0.028362	1.168966	-1.178559
H	1.598217	1.930977	-1.089337
C	0.195522	2.646327	0.372085
H	0.952310	3.013064	1.033655
H	-0.157878	3.446530	-0.244099
H	-0.617545	2.251053	0.944432
F	-2.592703	-1.010474	-0.487391
H	0.527972	0.111634	0.820017

int-s2b.log

P	-0.078952	-0.172452	-0.016445
B	1.517563	-2.133414	-2.003609
F	1.665031	-2.659535	-0.649707
F	2.515381	-2.746590	-2.875374
F	0.180425	-2.441361	-2.502438
C	-1.527850	-1.044515	0.656310
C	-2.294538	-0.107674	1.608212
C	-2.452408	-1.471039	-0.499056
H	-1.204588	-1.911782	1.193199
C	-3.520527	-0.845574	2.177468
H	-2.617800	0.759593	1.071324
H	-1.652150	0.188678	2.410966
C	-3.678399	-2.208939	0.070200
H	-2.775670	-0.603772	-1.035944
H	-1.919709	-2.121961	-1.160443
C	-4.445086	-1.272098	1.022103
H	-4.053226	-0.194653	2.838856
H	-3.197264	-1.712841	2.714356
H	-4.320787	-2.505289	-0.732554
H	-3.355137	-3.076206	0.607088
H	-5.296910	-1.784795	1.417625
H	-4.768349	-0.404831	0.485215
C	-0.628800	1.302709	-0.929665
C	-1.395494	2.239553	0.022231
C	0.597191	2.040609	-1.498919
H	-1.271184	1.006353	-1.732420
C	-1.860750	3.487763	-0.750496
H	-0.753110	2.535910	0.824985
H	-2.247318	1.726856	0.417752
C	0.131934	3.288820	-2.271646
H	1.239574	2.336966	-0.696164
H	1.129894	1.389685	-2.160300
C	-0.634760	4.225664	-1.319752
H	-2.393453	4.138687	-0.089115
H	-2.503134	3.191406	-1.553251
H	0.983759	3.801516	-2.667168
H	-0.510450	2.992462	-3.074401
H	-0.958023	5.092927	-1.856647
H	0.007624	4.522022	-0.516997
C	1.013700	0.331626	1.348992
C	1.478952	-0.916588	2.121715
C	2.239693	1.069524	0.779742
H	0.480997	0.982549	2.010374

C	2.403502	-0.490060	3.277086
H	2.011655	-1.567512	1.460333
H	0.627125	-1.429285	2.517233
C	3.164243	1.496053	1.935112
H	2.772396	0.418601	0.118360
H	1.916434	1.936790	0.242848
C	3.629496	0.247839	2.707836
H	2.726762	-1.357326	3.813979
H	1.870799	0.160863	3.938468
H	4.016070	2.008749	1.539594
H	2.631539	2.146976	2.596494
H	4.271878	0.544193	3.510593
H	4.162199	-0.403084	2.046454
F	1.709415	-0.686171	-1.986916
H	0.593149	-0.993709	-0.850900

int-s3.log

B	0.307613	2.696580	-0.762090
F	-0.400675	3.777438	-0.503229
F	0.000213	1.939032	-1.803177
F	1.403061	2.436774	-0.071459
F	-1.096649	1.411165	0.799165
O	-0.000328	-0.526492	0.494935
C	1.189041	-0.346955	1.136436
C	-1.203370	0.001169	1.056218
O	1.271397	0.120182	2.255104
O	-1.335046	-0.186320	2.381958
C	2.333930	-0.796272	0.320807
C	2.168630	-1.275414	-0.984242
C	3.612634	-0.719601	0.885205
C	3.280384	-1.676423	-1.716315
H	1.178720	-1.328154	-1.415549
C	4.719125	-1.122373	0.148624
H	3.720823	-0.342111	1.893693
C	4.553593	-1.600767	-1.151882
H	3.155198	-2.046268	-2.726700
H	5.709080	-1.063178	0.584240
H	5.417886	-1.913528	-1.726101
C	-2.365483	-0.537886	0.268736
C	-2.361969	-0.420784	-1.122897
C	-3.437507	-1.142528	0.921035
C	-3.434432	-0.911812	-1.859217
H	-1.528165	0.052069	-1.624128
C	-4.510458	-1.631561	0.178009
H	-3.428983	-1.229163	1.998796
C	-4.510484	-1.517844	-1.209940
H	-3.432420	-0.819651	-2.938738
H	-5.344285	-2.101484	0.685705
H	-5.345673	-1.899247	-1.785667
H	-0.483789	0.063489	2.793783

ts-s2.log

B	0.156347	1.878916	-1.294070
F	-0.356576	2.933035	-1.903291
F	0.099543	0.714365	-1.945147
F	1.264580	2.064451	-0.573109
F	-1.028328	1.621933	-0.019741
O	-0.000195	-0.362330	0.673587
C	1.198416	0.055592	1.216553
C	-1.157932	0.258667	0.987749
O	1.244980	0.805395	2.163132
O	-1.311349	0.794209	2.163376
C	2.350655	-0.547674	0.551403
C	2.211384	-1.383940	-0.556979

C	3.622898	-0.240588	1.039994
C	3.337258	-1.909260	-1.160111
H	1.229656	-1.602194	-0.942489
C	4.740654	-0.772717	0.430032
H	3.708710	0.418556	1.889792
C	4.598607	-1.607518	-0.668726
H	3.233398	-2.553149	-2.020164
H	5.724603	-0.536373	0.805424
H	5.474164	-2.020927	-1.147182
C	-2.353037	-0.421216	0.418487
C	-2.257221	-1.111023	-0.785501
C	-3.569143	-0.339084	1.088935
C	-3.381945	-1.716176	-1.314668
H	-1.315691	-1.148086	-1.306912
C	-4.686098	-0.952596	0.552759
H	-3.625935	0.200283	2.020631
C	-4.595006	-1.635715	-0.649442
H	-3.311817	-2.244922	-2.252905
H	-5.631042	-0.891366	1.071334
H	-5.473187	-2.100954	-1.071491
H	-0.431987	1.121798	2.492232

int-s4.log

B	0.093187	2.295066	-0.357638
F	0.156973	3.679241	-0.465488
F	0.142452	1.893374	1.001138
F	-1.100910	1.796605	-0.939382
F	1.188406	1.691036	-1.044597
O	-0.030062	-0.808163	-0.252035
C	-1.265827	-0.892161	-0.960356
C	1.140528	-0.859838	-0.852223
O	-1.263038	-1.122052	-2.146851
O	1.243546	-1.034363	-2.117127
C	-2.404088	-0.768929	-0.068905
C	-2.278153	-0.248053	1.226693
C	-3.656006	-1.165775	-0.563494
C	-3.407626	-0.141995	2.026057
H	-1.322495	0.117528	1.572120
C	-4.775183	-1.062658	0.248426
H	-3.733319	-1.550139	-1.572202
C	-4.650273	-0.552829	1.542491
H	-3.323150	0.276336	3.021096
H	-5.743662	-1.371939	-0.124114
H	-5.527748	-0.465915	2.172409
C	2.312839	-0.814542	-0.021897
C	2.198098	-0.495955	1.341340
C	3.569252	-1.071772	-0.597588
C	3.340581	-0.449160	2.123649
H	1.232435	-0.248721	1.755746
C	4.702070	-1.025830	0.197679
H	3.642759	-1.301963	-1.651806
C	4.587402	-0.716194	1.555298
H	3.265060	-0.190435	3.171921
H	5.674416	-1.223877	-0.234851
H	5.477529	-0.674320	2.171804
H	0.324687	-1.073428	-2.513886

ts-s3.log

B	-0.033078	3.267815	-0.523933
F	-0.524531	4.114231	0.359087
F	-0.747728	2.935159	-1.584097
F	1.186943	2.790683	-0.377418
F	-1.223786	1.256048	0.902257
O	0.138949	-0.560267	0.239693

C	1.260101	-0.463636	0.929164
C	-1.303630	-0.104857	1.076029
O	1.309505	-0.111360	2.112929
O	-1.195846	-0.468310	2.332184
C	2.485052	-0.826617	0.161180
C	2.420541	-1.208022	-1.184666
C	3.722500	-0.782160	0.814605
C	3.586193	-1.541884	-1.867893
H	1.462234	-1.239956	-1.686111
C	4.884881	-1.116354	0.127521
H	3.757707	-0.485050	1.855059
C	4.817933	-1.496475	-1.213782
H	3.534818	-1.836496	-2.909640
H	5.841614	-1.080840	0.635518
H	5.724538	-1.756419	-1.748609
C	-2.396086	-0.721042	0.282800
C	-2.850453	-0.108736	-0.889166
C	-2.945798	-1.934899	0.704805
C	-3.861093	-0.711042	-1.632338
H	-2.421455	0.830124	-1.214255
C	-3.957634	-2.530074	-0.042879
H	-2.584451	-2.401066	1.612178
C	-4.415087	-1.920043	-1.210755
H	-4.216939	-0.236127	-2.538943
H	-4.388254	-3.468401	0.286156
H	-5.203156	-2.385582	-1.791575
H	-0.264738	-0.229821	2.615224

int-s5a.log

B	2.186544	-0.106735	1.764031
F	2.278367	-1.466171	2.076029
F	2.462563	0.685900	2.895193
F	3.092407	0.228171	0.728505
O	0.527076	3.290744	-0.477094
C	0.936673	3.561495	-1.867448
H	1.374845	4.567273	-1.823732
H	1.696986	2.812271	-2.124841
C	-0.276125	3.488395	-2.767005
H	-1.050468	4.178732	-2.426808
H	0.030661	3.781822	-3.777199
H	-0.676774	2.474947	-2.818087
C	1.621599	3.207494	0.536463
H	2.279130	4.039192	0.280923
H	2.123647	2.255215	0.378696
C	1.010734	3.334500	1.906986
H	0.198972	2.607850	2.036854
H	1.788184	3.064381	2.647752
H	0.646696	4.353370	2.098273
F	0.861989	0.190526	1.306985
H	-0.108317	2.500748	-0.366766
O	-0.893982	-0.458012	-1.061055
C	-1.632364	0.440690	-0.396477
C	0.493682	-0.438405	-1.327836
O	-1.284603	1.581645	-0.131927
O	1.024003	0.537133	-1.774655
C	-2.937434	-0.123567	-0.002819
C	-3.360104	-1.369641	-0.485236
C	-3.735011	0.587452	0.900838
C	-4.574938	-1.896168	-0.066850
H	-2.738635	-1.914249	-1.181086
C	-4.941841	0.047206	1.327467
H	-3.391046	1.543615	1.272285
C	-5.362539	-1.192156	0.843922
H	-4.902266	-2.857284	-0.442732

H	-5.552731	0.586348	2.040520
H	-6.303899	-1.610910	1.178614
C	1.056532	-1.768466	-1.064388
C	0.343462	-2.710238	-0.311143
C	2.328715	-2.080314	-1.558663
C	0.897463	-3.958441	-0.062674
H	-0.623633	-2.456092	0.096465
C	2.869238	-3.338541	-1.321394
H	2.879330	-1.332647	-2.115105
C	2.156962	-4.275664	-0.572614
H	0.353053	-4.676481	0.536723
H	3.849526	-3.586788	-1.705246
H	2.587920	-5.249334	-0.375003

int-s5b.log

O	-2.400095	0.155640	-0.929408
C	-1.467771	1.174731	-0.725245
C	-2.459136	-0.914090	-0.066180
O	-0.301805	0.943704	-0.556288
O	-1.772119	-0.987710	0.923548
C	-2.089818	2.512884	-0.781489
C	-3.467567	2.680251	-0.966191
C	-1.261942	3.631083	-0.605008
C	-4.009055	3.961730	-0.976396
H	-4.114986	1.822845	-1.064979
C	-1.810838	4.906074	-0.612238
H	-0.199064	3.482054	-0.462171
C	-3.185294	5.071577	-0.801033
H	-5.077048	4.085877	-1.104550
H	-1.173171	5.771024	-0.473494
H	-3.612539	6.067774	-0.804793
C	-3.481656	-1.897607	-0.469837
C	-4.346275	-1.673869	-1.545381
C	-3.610534	-3.056966	0.303974
C	-5.332038	-2.607636	-1.843145
H	-4.261471	-0.765119	-2.123551
C	-4.595063	-3.987307	0.001459
H	-2.938067	-3.208992	1.138057
C	-5.457709	-3.761898	-1.072304
H	-6.013054	-2.426563	-2.665532
H	-4.697806	-4.882530	0.603052
H	-6.234251	-4.482374	-1.301553
B	-5.929558	0.674558	0.944592
F	-6.178050	1.029283	-0.392180
F	-6.475234	-0.580153	1.239912
F	-4.513856	0.627640	1.153109
F	-6.461452	1.655206	1.815558
P	3.208911	-0.052781	0.124300
C	4.266330	1.405167	0.413978
C	5.711155	1.046051	0.797532
C	4.218098	2.366513	-0.787091
H	3.784653	1.912080	1.254046
C	6.494685	2.327616	1.113857
H	6.197811	0.520006	-0.032012
H	5.730111	0.374900	1.660921
C	5.006343	3.640444	-0.462041
H	4.653833	1.889560	-1.672725
H	3.184146	2.618103	-1.023564
C	6.448505	3.318794	-0.054969
H	7.529904	2.072606	1.358510
H	6.060858	2.795334	2.004547
H	4.992549	4.304758	-1.330183
H	4.497915	4.165800	0.352656

H	6.977802	4.237366	0.214061
H	6.980591	2.888505	-0.913588
C	3.756905	-0.993364	-1.357169
C	4.783443	-2.095825	-1.036698
C	2.555552	-1.538149	-2.157146
H	4.253257	-0.237623	-1.977145
C	5.246992	-2.783941	-2.326895
H	4.327598	-2.841660	-0.378116
H	5.642338	-1.682355	-0.502188
C	3.039477	-2.233597	-3.434154
H	1.993707	-2.249272	-1.544691
H	1.862068	-0.731126	-2.402095
C	4.061397	-3.333507	-3.127422
H	5.947854	-3.586225	-2.079578
H	5.798066	-2.061293	-2.941215
H	2.180019	-2.646177	-3.969195
H	3.495762	-1.488620	-4.097934
H	4.414790	-3.791252	-4.055663
H	3.573783	-4.128722	-2.549768
C	3.076044	-1.101331	1.621970
C	2.742669	-0.240248	2.857894
C	2.035012	-2.221489	1.418728
H	4.066930	-1.551863	1.755442
C	2.604648	-1.115625	4.107864
H	1.810271	0.300462	2.686897
H	3.513327	0.516106	3.019303
C	1.896229	-3.073890	2.686084
H	1.072271	-1.768880	1.168472
H	2.314451	-2.859166	0.577256
C	1.558706	-2.215758	3.908411
H	2.336513	-0.479743	4.955333
H	3.573544	-1.573242	4.345730
H	1.126035	-3.833515	2.523390
H	2.835755	-3.611032	2.867716
H	1.489626	-2.842070	4.802617
H	0.574880	-1.752641	3.767559
H	1.927090	0.414058	-0.165483

ts-s4a.log

B	-1.110339	1.379474	2.269725
F	0.033988	1.939088	2.782903
F	-1.717683	0.483172	3.109053
F	-1.969251	2.291934	1.701069
O	-2.709552	-2.144767	-0.723451
C	-3.103283	-1.994701	-2.091953
H	-3.947278	-2.668780	-2.284378
H	-3.441679	-0.966234	-2.258016
C	-1.926850	-2.327570	-2.987852
H	-1.562466	-3.338597	-2.790695
H	-2.233248	-2.272767	-4.035289
H	-1.107868	-1.621146	-2.837187
C	-3.724694	-1.741881	0.209540
H	-4.655926	-2.258957	-0.052554
H	-3.889486	-0.663121	0.108498
C	-3.279990	-2.100757	1.612010
H	-2.375794	-1.559979	1.892134
H	-4.061057	-1.824820	2.324428
H	-3.099060	-3.175105	1.695710
F	-0.606454	0.487421	1.040441
H	-1.063628	-1.925297	-0.256105
O	0.549868	-0.341402	-1.109503
C	0.782218	-1.353662	-0.428765
C	-0.790315	1.004616	-0.705965
O	-0.136432	-2.134189	0.045323

O	-1.825141	0.659537	-1.078184
C	2.169191	-1.745489	-0.114463
C	3.217735	-0.927601	-0.553468
C	2.443219	-2.904835	0.621885
C	4.531375	-1.266673	-0.255040
H	2.988062	-0.036950	-1.123215
C	3.759457	-3.237435	0.918994
H	1.626963	-3.529061	0.959227
C	4.802421	-2.420376	0.481574
H	5.343431	-0.634740	-0.593509
H	3.973624	-4.131023	1.492412
H	5.827547	-2.682699	0.715777
C	0.066092	2.153964	-0.795221
C	1.258202	2.291886	-0.078708
C	-0.367390	3.159765	-1.670235
C	2.015065	3.442387	-0.239496
H	1.566629	1.517646	0.605950
C	0.402171	4.306461	-1.823956
H	-1.296722	3.039416	-2.211539
C	1.591091	4.447204	-1.111105
H	2.933570	3.560962	0.321408
H	0.069781	5.089395	-2.494074
H	2.186672	5.344692	-1.228920

ts-s4b.log

O	2.685178	-0.060942	-0.815467
C	1.769768	-1.086708	-0.907142
C	2.309610	1.253683	-0.739555
O	0.729611	-0.921425	-1.691341
O	1.160422	1.611872	-0.715986
C	2.488540	-2.387742	-1.140471
C	3.800093	-2.566893	-0.698528
C	1.808586	-3.434720	-1.763451
C	4.431603	-3.791702	-0.888376
H	4.316866	-1.760048	-0.198772
C	2.445363	-4.657906	-1.952066
H	0.792144	-3.284730	-2.100732
C	3.756656	-4.838033	-1.515799
H	5.449087	-3.930342	-0.543077
H	1.917541	-5.469044	-2.439202
H	4.251196	-5.791145	-1.662565
C	3.489420	2.141912	-0.692595
C	4.792424	1.645642	-0.805845
C	3.270933	3.513799	-0.524797
C	5.871200	2.521306	-0.752979
H	4.945432	0.583492	-0.937793
C	4.352347	4.383819	-0.468804
H	2.255221	3.877412	-0.436840
C	5.652191	3.888234	-0.583141
H	6.881128	2.139829	-0.842375
H	4.185726	5.445827	-0.335203
H	6.494858	4.568355	-0.539239
B	1.987467	-0.762585	2.288062
F	3.051515	-1.564831	2.223159
F	2.173420	0.548113	2.108914
F	1.203128	-1.179191	0.517227
F	0.918765	-1.167102	2.974885
P	-2.037954	0.179944	-0.265294
C	-2.087890	-1.103475	1.111727
C	-3.343534	-1.131478	1.992269
C	-1.780778	-2.497135	0.536968
H	-1.235795	-0.831611	1.741328
C	-3.210842	-2.174887	3.110757
H	-4.215759	-1.382481	1.378174

H	-3.532152	-0.143629	2.422605
C	-1.667805	-3.546748	1.650467
H	-2.573655	-2.795467	-0.161204
H	-0.848059	-2.455985	-0.028831
C	-2.915784	-3.566459	2.539899
H	-4.124909	-2.195852	3.713454
H	-2.395159	-1.879160	3.781983
H	-1.495115	-4.536051	1.214118
H	-0.790052	-3.314064	2.264356
H	-2.792630	-4.291423	3.350801
H	-3.777578	-3.900554	1.947037
C	-3.686248	-0.073708	-1.165543
C	-4.905000	0.684961	-0.617596
C	-3.512992	0.199438	-2.671126
H	-3.879764	-1.148003	-1.050112
C	-6.177406	0.332908	-1.400923
H	-4.730792	1.763205	-0.698619
H	-5.049485	0.471088	0.442813
C	-4.785599	-0.136992	-3.458001
H	-3.262460	1.256949	-2.820552
H	-2.665635	-0.374852	-3.056329
C	-6.005428	0.603685	-2.899369
H	-7.026010	0.901212	-1.006255
H	-6.411433	-0.728644	-1.249213
H	-4.645779	0.102602	-4.517041
H	-4.964555	-1.218512	-3.401068
H	-6.909289	0.314877	-3.445013
H	-5.874208	1.682204	-3.055785
C	-2.224189	1.815420	0.646153
C	-1.173633	1.941748	1.763583
C	-2.084843	2.969894	-0.363498
H	-3.219248	1.863541	1.101299
C	-1.229353	3.317922	2.439358
H	-0.177000	1.785402	1.341467
H	-1.324639	1.164741	2.516478
C	-2.146699	4.339628	0.322974
H	-1.125285	2.866845	-0.880285
H	-2.863852	2.902554	-1.128277
C	-1.086478	4.454797	1.422785
H	-0.444735	3.384821	3.199825
H	-2.187472	3.424958	2.964547
H	-2.015529	5.133346	-0.419875
H	-3.141673	4.482191	0.764270
H	-1.158562	5.425705	1.923534
H	-0.089522	4.405176	0.967051
H	0.082020	-0.255164	-1.341117

int-s6a.log

B	-2.024370	1.271625	1.768603
F	-1.231787	2.288859	2.310917
F	-2.751649	0.603530	2.745003
F	-2.881665	1.806056	0.773254
O	-1.968377	-2.770204	-0.505864
C	-2.525424	-2.872148	-1.820140
H	-3.235654	-3.706886	-1.831688
H	-3.079399	-1.956037	-2.050303
C	-1.403949	-3.095044	-2.814495
H	-0.860385	-4.004142	-2.570172
H	-1.805543	-3.196852	-3.820837
H	-0.706582	-2.259023	-2.804102
C	-2.938054	-2.458753	0.513225
H	-3.794453	-3.143223	0.389620
H	-3.292227	-1.428016	0.368043
C	-2.297062	-2.635565	1.876160

H	-1.474401	-1.927892	2.018130
H	-3.045952	-2.441115	2.649569
H	-1.922698	-3.662067	1.997229
F	-1.154206	0.328648	1.090868
H	-0.376749	-2.469015	-0.229739
O	0.734138	-0.643979	-1.285590
C	1.232529	-1.423188	-0.481436
C	-1.160624	1.033515	-1.180111
O	0.583476	-2.441289	0.053382
O	-1.922016	0.304695	-1.581691
C	2.640374	-1.294929	-0.017634
C	3.444747	-0.298709	-0.582633
C	3.162873	-2.129604	0.977187
C	4.759217	-0.138133	-0.157565
H	3.027333	0.336892	-1.352928
C	4.478244	-1.964748	1.401542
H	2.535026	-2.894212	1.414361
C	5.276763	-0.970695	0.835860
H	5.381338	0.632488	-0.598220
H	4.880587	-2.608110	2.175619
H	6.301376	-0.842282	1.169861
C	-0.357177	2.154331	-0.962596
C	0.744254	2.151893	-0.090901
C	-0.747102	3.310012	-1.677525
C	1.474909	3.321991	0.045535
H	0.997064	1.258010	0.448859
C	-0.007389	4.464262	-1.504763
H	-1.610129	3.290382	-2.330263
C	1.097942	4.467973	-0.651019
H	2.327169	3.345253	0.707748
H	-0.279321	5.368536	-2.047885
H	1.661009	5.376781	-0.520333

int-s6b.log

O	-2.667661	0.147680	-0.838647
C	-1.626737	1.115248	-0.863880
C	-2.389472	-1.183585	-0.704252
O	-0.610946	0.851553	-1.689640
O	-1.279309	-1.621974	-0.536906
C	-2.263811	2.457732	-1.124137
C	-3.500938	2.770982	-0.557203
C	-1.581881	3.407769	-1.882065
C	-4.053373	4.032959	-0.750734
H	-4.023960	2.033542	0.033689
C	-2.139661	4.669577	-2.076074
H	-0.626319	3.151499	-2.320046
C	-3.373779	4.985195	-1.506722
H	-5.012340	4.276055	-0.303296
H	-1.608862	5.405665	-2.665071
H	-3.806808	5.967687	-1.655648
C	-3.622612	-2.004105	-0.782815
C	-4.877250	-1.445958	-1.051729
C	-3.500331	-3.382280	-0.573579
C	-5.999509	-2.265896	-1.112630
H	-4.966179	-0.380904	-1.215761
C	-4.624619	-4.196457	-0.632105
H	-2.521518	-3.794891	-0.365408
C	-5.875131	-3.638790	-0.901806
H	-6.970850	-1.835011	-1.323958
H	-4.529283	-5.262985	-0.467158
H	-6.751905	-4.274303	-0.947773
B	-2.022705	0.697245	2.623387
F	-2.944492	1.643397	2.530789
F	-2.344471	-0.551846	2.349633

F	-1.119324	1.130369	0.496422
F	-0.852773	0.974213	3.176181
P	1.999574	-0.235809	-0.281439
C	2.151564	1.150187	0.963744
C	3.357262	1.112331	1.913166
C	2.058739	2.515697	0.257968
H	1.241596	1.045848	1.563927
C	3.285434	2.254352	2.937248
H	4.282417	1.212207	1.333221
H	3.409861	0.149750	2.430538
C	1.991756	3.661368	1.276206
H	2.937616	2.660815	-0.383308
H	1.183590	2.541910	-0.395004
C	3.179024	3.619188	2.245165
H	4.163441	2.226340	3.591334
H	2.407348	2.104179	3.578702
H	1.961419	4.623833	0.753757
H	1.055396	3.578896	1.842143
H	3.091919	4.417947	2.988991
H	4.105727	3.809589	1.686173
C	3.626914	-0.170644	-1.223037
C	4.808264	-0.960897	-0.639995
C	3.406361	-0.545462	-2.699399
H	3.896260	0.891647	-1.199585
C	6.082716	-0.743616	-1.468098
H	4.568852	-2.028625	-0.637480
H	4.987055	-0.678857	0.398632
C	4.679607	-0.338323	-3.528645
H	3.096038	-1.594537	-2.764753
H	2.584175	0.044750	-3.111406
C	5.866948	-1.105592	-2.940493
H	6.903482	-1.332609	-1.049176
H	6.381767	0.308804	-1.395001
H	4.506364	-0.641575	-4.564754
H	4.918222	0.731752	-3.550237
H	6.774715	-0.904053	-3.518643
H	5.675279	-2.182373	-3.021300
C	2.087121	-1.785106	0.757369
C	1.035457	-1.754142	1.881218
C	1.877953	-3.019890	-0.139917
H	3.079845	-1.851019	1.216332
C	1.038184	-3.057202	2.690400
H	0.047386	-1.604135	1.439122
H	1.216498	-0.911813	2.552473
C	1.892367	-4.317971	0.676331
H	0.915434	-2.920881	-0.650659
H	2.646460	-3.063286	-0.916415
C	0.842911	-4.283155	1.791963
H	0.253680	-3.015459	3.453258
H	1.993006	-3.150795	3.224697
H	1.720546	-5.174719	0.016174
H	2.886865	-4.457089	1.120566
H	0.887809	-5.201573	2.386192
H	-0.157256	-4.243108	1.343015
H	0.091106	0.268443	-1.270895

ts-s5a.log

B	1.708292	2.627380	-1.006140
F	3.022337	2.495965	-0.788881
F	1.039775	3.538758	-0.286145
F	1.239910	2.322261	-2.225725
O	3.165327	-1.771729	0.147775
C	3.335967	-1.784910	1.576052
H	4.411122	-1.775536	1.790180

H	2.889224	-0.876347	1.992968
C	2.685440	-3.028108	2.147509
H	3.106213	-3.929766	1.696526
H	2.859267	-3.067651	3.225749
H	1.606986	-3.017476	1.982702
C	3.892683	-0.703932	-0.487205
H	4.963190	-0.885316	-0.334061
H	3.628519	0.241517	-0.007480
C	3.551303	-0.674388	-1.962567
H	2.494999	-0.447327	-2.117209
H	4.136589	0.104310	-2.456273
H	3.779739	-1.632562	-2.435537
F	1.105236	1.050496	-0.132071
H	1.646721	-1.986649	-0.360920
O	0.126365	-0.954316	1.058276
C	-0.168791	-1.616968	0.055173
C	0.298137	1.046675	1.215417
O	0.730869	-2.178524	-0.701131
O	0.961793	1.147735	2.171285
C	-1.570396	-1.813460	-0.375022
C	-2.595702	-1.567666	0.544138
C	-1.880304	-2.192809	-1.686188
C	-3.922142	-1.694410	0.154367
H	-2.338885	-1.271358	1.551778
C	-3.210204	-2.303685	-2.076320
H	-1.080694	-2.380239	-2.390646
C	-4.230503	-2.055419	-1.157232
H	-4.715072	-1.502948	0.866921
H	-3.452708	-2.582778	-3.094646
H	-5.266205	-2.144470	-1.464178
C	-1.087569	1.375796	0.910928
C	-1.588364	1.446581	-0.391819
C	-1.928264	1.615648	2.004823
C	-2.925732	1.762911	-0.596183
H	-0.940719	1.254392	-1.233820
C	-3.265125	1.925812	1.790595
H	-1.526667	1.554930	3.008134
C	-3.764734	1.999949	0.490290
H	-3.313535	1.815937	-1.605740
H	-3.915756	2.112773	2.636222
H	-4.808138	2.241315	0.325258

ts-s5b.log

O	-0.110823	-0.211199	0.329270
C	1.329273	-1.182835	0.397023
C	-1.187550	-0.812208	-0.024910
O	1.146617	-2.194904	-0.350807
O	-1.207212	-2.042963	-0.345635
C	2.388410	-0.185203	0.080600
C	2.798789	0.751958	1.031263
C	2.948024	-0.183645	-1.197646
C	3.773718	1.687334	0.700267
H	2.359303	0.743611	2.019939
C	3.925102	0.752414	-1.523125
H	2.618177	-0.919068	-1.919695
C	4.337229	1.688600	-0.575839
H	4.095929	2.413393	1.437055
H	4.364838	0.750612	-2.513283
H	5.097980	2.417375	-0.830276
C	-2.436041	-0.024574	-0.058888
C	-2.428620	1.325458	0.310524
C	-3.626668	-0.637866	-0.464971
C	-3.609751	2.056800	0.272205
H	-1.500642	1.785739	0.622751

C -4.804654 0.098965 -0.500276
H -3.615703 -1.682258 -0.747555
C -4.796609 1.444834 -0.132637
H -3.607321 3.101832 0.557301
H -5.727714 -0.373122 -0.814070
H -5.716341 2.017415 -0.161145
F 1.265407 -1.443693 1.752532
H -0.049730 -2.349971 -0.367975

int-s7.log

C 3.943995 -2.607377 2.276475
C 4.612890 -2.420372 1.068407
C 4.236363 -1.394609 0.211599
C 3.164369 -0.563097 0.560562
C 2.483925 -0.760324 1.772021
C 2.883549 -1.772728 2.630644
H 4.247358 -3.405731 2.942707
H 5.429538 -3.074541 0.791337
H 4.749723 -1.255285 -0.728239
H 1.657737 -0.116903 2.036209
H 2.367250 -1.917000 3.570887
C 2.703180 0.473599 -0.365232
O 1.435786 0.808870 -0.194146
O 3.359705 1.041819 -1.254580
C -1.347250 4.904241 0.436465
C -0.385309 4.255324 1.207999
C 0.288855 3.157832 0.698893
C 0.013472 2.718885 -0.601873
C -0.945617 3.378740 -1.380362
C -1.625217 4.465391 -0.858517
H -1.886715 5.751831 0.846517
H -0.179801 4.595230 2.223204
H -4.593669 0.383818 -1.007165
H -1.140001 3.024328 -2.388818
H -2.376515 4.971176 -1.452810
C 0.715059 1.580249 -1.209630
O 0.738938 1.217750 -2.336080
P -1.695909 -0.394955 -0.129623
C -2.467222 0.028197 1.534669
C -1.379751 0.085528 2.625557
C -3.169060 1.398219 1.439776
H -3.204714 -0.731346 1.820216
C -1.929381 0.562884 3.976380
H -0.589629 0.772563 2.297017
H -0.913341 -0.892810 2.756476
C -3.744325 1.845139 2.787955
H -2.441641 2.140867 1.096602
H -3.960975 1.373586 0.691364
C -2.649088 1.909309 3.856299
H -1.110119 0.632079 4.701259
H -2.629462 -0.188247 4.364828
H -4.234314 2.819517 2.679663
H -4.520590 1.135160 3.105874
H -3.067560 2.204476 4.823381
H -1.923292 2.683457 3.574409
C -0.686056 -1.924877 0.283703
C -1.413893 -3.071735 1.000520
C 0.051785 -2.447862 -0.961144
H 0.084069 -1.543914 0.960822
C -0.443699 -4.205465 1.366594
H -2.197394 -3.475586 0.348724
H -1.917553 -2.707895 1.900463
C 1.032566 -3.566805 -0.589132
H -0.674538 -2.836611 -1.684241

H	0.577949	-1.629150	-1.459394
C	0.318905	-4.713285	0.136577
H	-0.994323	-5.027628	1.837937
H	0.275316	-3.837365	2.110805
H	1.538129	-3.936999	-1.486607
H	1.811561	-3.157730	0.063929
H	1.038683	-5.484891	0.429128
H	-0.389762	-5.189283	-0.554625
C	-3.074319	-1.147013	-1.182621
C	-4.506310	-0.699362	-0.857805
C	-2.764686	-0.859966	-2.665521
H	-3.035268	-2.232170	-1.034812
C	-5.521816	-1.401307	-1.769474
H	1.034871	2.650189	1.293839
H	-4.748422	-0.897652	0.189783
C	-3.774277	-1.545100	-3.594446
H	-2.792016	0.225356	-2.826077
H	-1.748598	-1.177754	-2.915209
C	-5.213647	-1.146419	-3.249642
H	-6.535198	-1.061801	-1.527069
H	-5.494986	-2.481804	-1.572687
H	-3.550349	-1.301837	-4.638369
H	-3.668850	-2.634138	-3.496590
H	-5.921117	-1.689174	-3.883949
H	-5.352448	-0.078935	-3.464762
B	4.954533	1.288082	-1.475675
F	5.396458	0.226514	-2.233861
F	5.506778	1.336057	-0.215302
F	4.977163	2.486534	-2.134056

ts-s6.log

C	3.705633	-1.835876	4.932936
C	3.551106	-0.501625	5.268520
C	2.633302	0.287806	4.599518
C	1.840395	-0.260543	3.590315
C	2.032543	-1.597394	3.229378
C	2.957992	-2.375976	3.897260
H	4.407542	-2.453670	5.473787
H	4.138487	-0.070846	6.064672
H	2.542446	1.325615	4.861853
H	1.440703	-2.025327	2.436016
H	3.090973	-3.411574	3.620416
C	0.769655	0.529558	2.944116
O	0.372890	0.112880	1.784797
O	0.201914	1.518522	3.442967
C	-4.668917	-0.143841	2.079160
C	-3.635463	-1.055287	2.219435
C	-2.337240	-0.690577	1.909232
C	-2.068318	0.590743	1.445715
C	-3.103745	1.514973	1.340248
C	-4.398312	1.146196	1.653489
H	-5.681165	-0.434842	2.317629
H	-3.842108	-2.054071	2.575722
H	-2.868015	0.241671	-2.602255
H	-2.868058	2.523079	1.037258
H	-5.196485	1.868872	1.573848
C	-0.714901	1.062952	1.067434
O	-0.376332	2.215446	0.957945
P	-0.222524	0.129807	-0.820178
C	-0.394948	-1.701695	-1.210678
C	0.494538	-2.542666	-0.296213
C	-1.831861	-2.208071	-1.074234
H	-0.070314	-1.829032	-2.250842
C	0.444961	-4.007801	-0.725825

H	0.126557	-2.453401	0.727123
H	1.527419	-2.193442	-0.312243
C	-1.903456	-3.665529	-1.528247
H	-2.147028	-2.139224	-0.033197
H	-2.525069	-1.609974	-1.661505
C	-0.985081	-4.536485	-0.677181
H	1.085302	-4.605042	-0.073633
H	0.831454	-4.098137	-1.744238
H	-2.931682	-4.023230	-1.451648
H	-1.603689	-3.735560	-2.577059
H	-1.012106	-5.567733	-1.033783
H	-1.338895	-4.530669	0.355985
C	1.611683	0.466015	-0.967197
C	2.258647	-0.156246	-2.208809
C	1.921287	1.966718	-0.925697
H	2.050815	0.009178	-0.071521
C	3.768770	0.081256	-2.182999
H	1.847307	0.294965	-3.114891
H	2.074247	-1.230185	-2.256287
C	3.429962	2.196122	-0.899587
H	1.509946	2.458890	-1.809561
H	1.469264	2.420541	-0.044994
C	4.085047	1.571568	-2.126395
H	4.221349	-0.365631	-3.071015
H	4.196087	-0.410927	-1.306006
H	3.630714	3.268308	-0.871954
H	3.852711	1.755345	0.006037
H	5.165640	1.721234	-2.094555
H	3.707275	2.058751	-3.028665
C	-0.905652	1.082438	-2.291184
C	-1.939688	0.373034	-3.162056
C	-1.488888	2.424959	-1.841106
H	-0.027767	1.279056	-2.919076
C	-2.231593	1.226941	-4.396873
H	-1.528502	-1.392789	2.048807
H	-1.579742	-0.604818	-3.485578
C	-1.782518	3.296847	-3.059416
H	-2.415507	2.242439	-1.293159
H	-0.805499	2.941348	-1.167941
C	-2.764815	2.597858	-3.993859
H	-2.960109	0.714324	-5.028398
H	-1.314383	1.348611	-4.978407
H	-2.194023	4.253517	-2.734651
H	-0.852163	3.500865	-3.595509
H	-2.933180	3.205930	-4.884673
H	-3.723686	2.477049	-3.485242
B	0.282105	2.397088	4.745675
F	1.419302	3.140843	4.644960
F	0.317470	1.540581	5.807664
F	-0.842015	3.138993	4.710304

int-s8a.log

C	5.146462	-2.383328	0.754216
C	5.141223	-1.705207	-0.463143
C	4.282683	-0.627641	-0.665843
C	3.404887	-0.237142	0.348388
C	3.402592	-0.930085	1.564891
C	4.278145	-1.989593	1.772936
H	5.824330	-3.214748	0.909927
H	5.811483	-2.011014	-1.257734
H	4.290659	-0.095306	-1.606292
H	2.713693	-0.622538	2.341418
H	4.283156	-2.509547	2.723940
C	2.379380	0.836793	0.151805

O	1.291022	0.712663	0.730260
O	2.581850	1.845238	-0.636511
C	-2.222944	4.767836	-0.142863
C	-1.078883	4.325370	0.519930
C	-0.551668	3.064851	0.256386
C	-1.169265	2.250670	-0.694642
C	-2.301380	2.700329	-1.381704
C	-2.832583	3.954957	-1.096214
H	-2.630300	5.748509	0.072741
H	-0.588325	4.965903	1.242942
H	-4.353024	0.013344	-0.785263
H	-2.766641	2.074251	-2.133808
H	-3.711967	4.299593	-1.627122
C	-0.688491	0.876415	-1.029932
O	-0.108307	0.578497	-2.045466
P	-1.341907	-0.603877	0.025789
C	-1.979112	-0.181397	1.712236
C	-0.865161	-0.013346	2.764812
C	-2.947007	1.018771	1.778543
H	-2.548966	-1.086753	1.963369
C	-1.481815	0.089417	4.165548
H	-0.289524	0.883921	2.535056
H	-0.160061	-0.843975	2.733034
C	-3.575970	1.098832	3.175134
H	-2.392713	1.936671	1.586592
H	-3.724314	0.955728	1.020012
C	-2.501392	1.228536	4.258881
H	-0.683286	0.231688	4.899128
H	-1.972582	-0.860252	4.414541
H	-4.260544	1.950870	3.212299
H	-4.178606	0.199935	3.357711
H	-2.961228	1.245240	5.251112
H	-1.982527	2.186424	4.133875
C	-0.045591	-1.909164	0.211367
C	-0.561925	-3.099211	1.045890
C	0.548505	-2.391210	-1.125349
H	0.737720	-1.395027	0.768655
C	0.584272	-4.089014	1.300820
H	-1.365451	-3.618526	0.511720
H	-0.975672	-2.766768	2.000110
C	1.675126	-3.400764	-0.868913
H	-0.232312	-2.870926	-1.725695
H	0.926838	-1.547302	-1.699922
C	1.204398	-4.577694	-0.011121
H	0.208983	-4.932044	1.888237
H	1.353248	-3.594571	1.905073
H	2.060065	-3.756297	-1.828683
H	2.502237	-2.891801	-0.368314
H	2.040974	-5.250751	0.197039
H	0.459206	-5.162240	-0.565901
C	-2.706541	-1.383558	-0.987003
C	-4.146063	-1.038799	-0.567055
C	-2.530211	-1.188798	-2.506429
H	-2.553464	-2.447362	-0.771917
C	-5.144121	-1.915894	-1.336035
H	0.332958	2.704963	0.758875
H	-4.288250	-1.182268	0.505370
C	-3.529322	-2.064235	-3.272389
H	-2.705596	-0.138709	-2.759529
H	-1.512007	-1.413895	-2.820078
C	-4.972679	-1.774683	-2.851020
H	-6.161893	-1.648571	-1.038263
H	-4.996595	-2.963725	-1.046703
H	-3.402611	-1.898384	-4.345568

H	-3.297743	-3.120518	-3.087908
H	-5.662522	-2.443291	-3.373322
H	-5.237282	-0.752957	-3.150102
B	3.852851	2.507215	-1.136343
F	4.192827	1.953509	-2.381193
F	4.881251	2.322095	-0.205578
F	3.537587	3.857933	-1.271540

int-s8b.log

C	-4.962900	-0.508421	-1.626361
C	-4.221381	0.626369	-1.951076
C	-2.832988	0.603783	-1.886113
C	-2.179582	-0.571283	-1.509741
C	-2.923090	-1.725862	-1.224054
C	-4.311934	-1.686548	-1.262942
H	-6.045138	-0.481112	-1.675309
H	-4.724559	1.529554	-2.274195
H	-1.513770	-1.788815	1.229514
H	-2.409544	-2.645520	-0.972222
H	-4.882747	-2.576961	-1.028206
C	-0.703116	-0.731081	-1.444432
O	-0.119802	-1.621924	-2.010193
P	0.422286	0.138245	-0.153993
C	-0.139172	1.630423	0.813350
C	0.561488	2.953154	0.453054
C	-1.663608	1.841180	0.846644
H	0.180926	1.351210	1.825675
C	0.196167	4.033884	1.480859
H	0.247589	3.254051	-0.546828
H	1.645566	2.835225	0.431237
C	-2.025451	2.902905	1.891330
H	-1.987431	2.182947	-0.135794
H	-2.198225	0.912152	1.047020
C	-1.318546	4.229075	1.595791
H	0.681024	4.973242	1.199980
H	0.602462	3.751952	2.460685
H	-3.110072	3.042043	1.902903
H	-1.741527	2.546279	2.889752
H	-1.547364	4.964619	2.372276
H	-1.701688	4.635044	0.652035
C	2.110988	0.476338	-0.837613
C	3.158539	0.494996	0.295113
C	2.578816	-0.397318	-2.015243
H	1.994953	1.489759	-1.223051
C	4.498060	1.015589	-0.242428
H	3.301931	-0.515996	0.687722
H	2.828897	1.117716	1.131645
C	3.930032	0.116017	-2.530930
H	2.673259	-1.440100	-1.703882
H	1.847409	-0.379879	-2.820060
C	4.985581	0.171264	-1.423433
H	5.236863	1.015867	0.564267
H	4.375436	2.056962	-0.563132
H	4.265311	-0.527083	-3.348991
H	3.795000	1.115435	-2.954561
H	5.922975	0.577220	-1.814350
H	5.203670	-0.845512	-1.072809
C	0.569186	-1.181755	1.149393
C	-0.750902	-1.405922	1.913952
C	1.151721	-2.514300	0.646794
H	1.289105	-0.742444	1.851248
C	-0.553124	-2.403408	3.062903
H	-2.245662	1.461692	-2.171625
H	-1.130161	-0.465709	2.316928

C	1.345625	-3.492876	1.812440
H	0.479768	-2.958849	-0.090502
H	2.102902	-2.350737	0.139270
C	0.036553	-3.727272	2.570522
H	-1.511068	-2.570602	3.563315
H	0.119126	-1.960911	3.808454
H	1.739949	-4.436992	1.426746
H	2.100016	-3.093535	2.501709
H	0.200353	-4.404786	3.413197
H	-0.683183	-4.219294	1.904713

int-s8c.log

C	-3.275210	-1.003904	0.101989
C	-2.139901	-1.589910	-0.454102
C	-0.956778	-0.864826	-0.566084
C	-0.911222	0.464460	-0.137421
C	-2.057470	1.053474	0.409742
C	-3.230808	0.319995	0.540816
H	-4.191486	-1.575647	0.194457
H	-2.172269	-2.616524	-0.799147
H	-0.076621	-1.324708	-0.992309
H	-2.013963	2.084740	0.734719
H	-4.109491	0.777339	0.980635
C	0.294918	1.323657	-0.345751
O	0.132990	2.545058	-0.488798
O	1.482948	0.815707	-0.450502
B	2.122962	-0.413892	0.170606
F	2.092147	-1.460588	-0.763147
F	1.435562	-0.759271	1.339492
F	3.436775	-0.044533	0.451197

int-s9a.log

C	0.012293	-1.208474	1.367106
H	0.157130	-2.257152	1.615691
H	-1.035994	-0.953101	1.535340
H	0.625840	-0.596575	2.029411
C	0.343749	-0.937795	-0.078085
H	-0.180936	-1.588355	-0.774644
H	0.184736	0.099857	-0.367852
F	1.741345	-1.208809	-0.296111
B	3.109605	0.312934	-0.130418
F	2.464938	1.156355	-0.959707
F	2.972177	0.497620	1.177141
F	4.118150	-0.389678	-0.589281
O	-2.404250	0.222427	-0.630570
C	-2.362821	1.646847	-0.566639
H	-1.550072	1.941174	-1.230523
H	-3.293808	2.051756	-0.981359
C	-2.130720	2.197570	0.833683
H	-1.207751	1.805313	1.261301
H	-2.053234	3.285704	0.792081
H	-2.954344	1.949478	1.506189
C	-3.552486	-0.354394	-0.015826
H	-4.459233	0.167782	-0.358309
H	-3.498150	-0.234026	1.075763
C	-3.613357	-1.828045	-0.375627
H	-2.693258	-2.340322	-0.064787
H	-4.460022	-2.296273	0.142026
H	-3.744372	-1.972972	-1.459738

int-s9b.log

B	-4.575570	-0.113846	-0.878887
F	-5.705505	0.101057	-1.539285
F	-4.182672	0.746357	0.052631

F	-4.040282	-1.343785	-0.878786
C	-1.929320	0.642462	-2.110898
H	-1.825088	1.208536	-1.187131
H	-1.667206	-0.396112	-1.940373
C	-1.214780	1.263647	-3.279765
H	-1.545380	2.293468	-3.448706
H	-0.147753	1.270861	-3.051823
H	-1.371888	0.682315	-4.192632
F	-3.357062	0.639942	-2.386467
P	0.840180	-0.040294	-0.048452
C	2.467524	-0.971214	-0.290003
C	2.397164	-2.496846	-0.116173
C	3.017400	-0.656754	-1.695338
H	3.172947	-0.594294	0.461637
C	3.779980	-3.135046	-0.315091
H	1.700657	-2.917636	-0.851154
H	2.016687	-2.762086	0.871297
C	4.400298	-1.280995	-1.917602
H	2.311774	-1.055543	-2.435061
H	3.062788	0.420123	-1.866802
C	4.372593	-2.795319	-1.685772
H	3.708515	-4.220348	-0.191601
H	4.454085	-2.772627	0.470750
H	4.754556	-1.056687	-2.928847
H	5.116180	-0.820598	-1.224451
H	5.377768	-3.213967	-1.778827
H	3.763347	-3.265879	-2.465426
C	0.077882	-0.948618	1.426097
C	-0.755643	-0.070913	2.374846
C	-0.805333	-2.095516	0.896321
H	0.901707	-1.372343	2.014343
C	-1.349012	-0.899932	3.522263
H	-1.575515	0.395436	1.817125
H	-0.151414	0.737199	2.791033
C	-1.400523	-2.935760	2.032283
H	-1.623149	-1.658232	0.312171
H	-0.245986	-2.734992	0.211154
C	-2.200560	-2.062013	3.002434
H	-1.946063	-0.251779	4.170816
H	-0.532523	-1.296482	4.138148
H	-2.034643	-3.724991	1.617014
H	-0.588735	-3.436490	2.575950
H	-2.576789	-2.661976	3.836195
H	-3.077123	-1.659701	2.480897
C	1.439443	1.569296	0.746415
C	2.711781	2.167332	0.122492
C	0.314553	2.621374	0.679265
H	1.662785	1.352269	1.799290
C	3.125010	3.462249	0.837257
H	2.528488	2.386716	-0.936849
H	3.540133	1.457677	0.161103
C	0.703572	3.919564	1.397196
H	0.112387	2.839044	-0.377196
H	-0.615065	2.231714	1.096344
C	2.000979	4.502841	0.828145
H	4.025860	3.871623	0.368356
H	3.391068	3.225398	1.875444
H	-0.109301	4.647991	1.315171
H	0.834053	3.713335	2.467257
H	2.301108	5.391771	1.391837
H	1.823510	4.829745	-0.204312

ts-s7a.log

C	-0.643697	-1.014696	1.760591

H	-1.316219	-1.871574	1.752398
H	-1.016928	-0.215008	2.399066
H	0.311473	-1.355566	2.190237
C	-0.251819	-0.557020	0.424300
H	-0.077113	-1.272438	-0.366053
H	0.242565	0.397196	0.306235
F	2.171497	-0.979026	0.666523
B	2.796891	-0.006576	-0.196974
F	2.511482	-0.358946	-1.526234
F	2.200724	1.250821	0.092534
F	4.164116	0.024909	0.046993
O	-1.919082	0.126842	-0.507920
C	-1.933315	1.579665	-0.659722
H	-0.955812	1.819568	-1.076918
H	-2.688810	1.791803	-1.420633
C	-2.200071	2.342798	0.624647
H	-1.429358	2.155575	1.375014
H	-2.187885	3.412479	0.399664
H	-3.177037	2.104164	1.050400
C	-3.187751	-0.499806	-0.170376
H	-3.969729	0.131800	-0.598831
H	-3.311074	-0.505358	0.915739
C	-3.234680	-1.894955	-0.759341
H	-2.456852	-2.540293	-0.346317
H	-4.201711	-2.348088	-0.526515
H	-3.119685	-1.861853	-1.844193

ts-s7b.log

B	-4.074900	-0.012604	-1.382082
F	-5.166422	0.241261	-2.094014
F	-3.871260	0.790555	-0.319272
F	-3.818721	-1.309030	-1.144942
C	-1.298630	0.329192	-1.784007
H	-1.525684	0.889952	-0.890698
H	-1.276820	-0.746937	-1.679843
C	-0.563217	0.972271	-2.910194
H	-0.812393	2.031780	-2.976923
H	0.511657	0.874090	-2.784248
H	-0.837982	0.485212	-3.847315
F	-2.868958	0.422613	-2.410300
P	0.697936	-0.039390	-0.003206
C	2.312797	-1.013621	-0.165858
C	2.189646	-2.527368	0.069745
C	2.917022	-0.766103	-1.561969
H	2.996489	-0.618559	0.595925
C	3.554034	-3.217693	-0.070150
H	1.495855	-2.956705	-0.662683
H	1.779153	-2.736717	1.059028
C	4.283335	-1.444858	-1.715334
H	2.226037	-1.169936	-2.312476
H	3.008427	0.301745	-1.767614
C	4.191018	-2.948349	-1.437022
H	3.438890	-4.294105	0.090632
H	4.221489	-2.851259	0.719958
H	4.676928	-1.266043	-2.720765
H	4.991817	-0.986600	-1.013777
H	5.182117	-3.408757	-1.492206
H	3.580460	-3.420486	-2.217065
C	-0.144026	-0.845215	1.485377
C	-1.018020	0.102434	2.326066
C	-1.002800	-2.038813	1.019642
H	0.659504	-1.214671	2.135700
C	-1.631384	-0.637037	3.524103
H	-1.828026	0.503541	1.706549

H	-0.437402	0.952125	2.689895
C	-1.605008	-2.793511	2.210910
H	-1.821325	-1.666503	0.395925
H	-0.423344	-2.724203	0.399753
C	-2.445444	-1.857988	3.085223
H	-2.259076	0.053062	4.096414
H	-0.824305	-0.959511	4.194465
H	-2.214958	-3.626720	1.849128
H	-0.795788	-3.229361	2.811106
H	-2.828785	-2.392610	3.959788
H	-3.317167	-1.521503	2.511457
C	1.285221	1.616287	0.703771
C	2.599766	2.150737	0.111255
C	0.185174	2.681374	0.524502
H	1.451264	1.454186	1.776299
C	3.003712	3.475812	0.773932
H	2.475793	2.315465	-0.965672
H	3.408157	1.427739	0.232397
C	0.570074	4.008126	1.190029
H	0.038137	2.851164	-0.549015
H	-0.771032	2.335936	0.919682
C	1.903019	4.533645	0.649139
H	3.934809	3.840235	0.328699
H	3.212921	3.293209	1.835428
H	-0.223560	4.744937	1.033673
H	0.649340	3.855684	2.273697
H	2.194256	5.449074	1.173006
H	1.780137	4.801403	-0.408005

int-s10a.log

C	-0.533091	-0.508966	1.566324
H	-1.118975	-1.364672	1.948309
H	-0.887407	0.418769	2.055469
H	0.520719	-0.646907	1.841992
C	-0.547745	-0.408241	0.065171
H	-0.359182	-1.344149	-0.446929
H	0.140292	0.331792	-0.320377
F	2.091595	-1.305448	-0.219714
B	2.772071	-0.053228	-0.124714
F	2.114412	0.872519	-0.984770
F	2.666141	0.414460	1.203871
F	4.100868	-0.210907	-0.499745
O	-1.902027	0.049165	-0.466192
C	-2.013985	1.497066	-0.786269
H	-1.107094	1.701372	-1.351519
H	-2.871543	1.549556	-1.459918
C	-2.159174	2.385415	0.428519
H	-1.306480	2.291838	1.145700
H	-2.171863	3.436199	0.044647
H	-3.122988	2.212591	0.951912
C	-3.118550	-0.644721	0.023408
H	-3.940309	-0.097405	-0.436342
H	-3.158211	-0.520502	1.106287
C	-3.084202	-2.094388	-0.411717
H	-2.289416	-2.651825	0.101485
H	-4.041452	-2.552998	-0.138785
H	-2.946899	-2.183441	-1.498223

int-s10b.log

B	-3.895560	0.045991	-1.674606
F	-5.239626	0.176956	-2.064309
F	-3.600716	0.934706	-0.600317
F	-3.638256	-1.277635	-1.237737
C	-0.340807	0.020288	-1.404270

H	-1.308143	0.407613	-1.090810
H	-0.514205	-1.025267	-1.677973
C	0.190158	0.817654	-2.602670
H	0.286520	1.874598	-2.383611
H	1.152212	0.455251	-2.956473
H	-0.531707	0.714577	-3.409922
F	-3.044224	0.349086	-2.761930
P	0.685206	-0.026104	0.098557
C	2.202604	-1.070221	-0.108208
C	2.014638	-2.571256	0.188552
C	2.868129	-0.916090	-1.492066
H	2.881816	-0.667491	0.654644
C	3.378925	-3.276462	0.186801
H	1.375874	-3.017325	-0.579569
H	1.524240	-2.731536	1.148182
C	4.236011	-1.610091	-1.508534
H	2.221231	-1.384977	-2.239755
H	2.979230	0.128897	-1.775933
C	4.113921	-3.092717	-1.144559
H	3.237523	-4.338930	0.402458
H	3.988522	-2.868274	1.002978
H	4.689489	-1.494725	-2.496164
H	4.900533	-1.107587	-0.794973
H	5.103132	-3.558354	-1.099832
H	3.556387	-3.610785	-1.935029
C	-0.321237	-0.756816	1.466701
C	-1.169590	0.241244	2.281217
C	-1.223741	-1.907687	0.971019
H	0.441141	-1.167039	2.141900
C	-1.799335	-0.482442	3.481636
H	-1.960553	0.651882	1.646697
H	-0.564220	1.073335	2.644791
C	-1.831222	-2.640040	2.174393
H	-2.029026	-1.494595	0.359166
H	-0.677390	-2.609377	0.340175
C	-2.649603	-1.677614	3.041046
H	-2.400079	0.226958	4.058311
H	-0.997524	-0.831994	4.146175
H	-2.456885	-3.462091	1.819006
H	-1.026364	-3.086327	2.773543
H	-3.056653	-2.197793	3.913373
H	-3.503330	-1.315722	2.456584
C	1.232316	1.663282	0.619212
C	2.538961	2.165401	-0.025477
C	0.125120	2.727518	0.458548
H	1.427757	1.526297	1.691093
C	2.974955	3.487970	0.622396
H	2.380949	2.323065	-1.095758
H	3.339706	1.433135	0.079842
C	0.557293	4.037658	1.129050
H	-0.046075	2.909691	-0.605641
H	-0.824767	2.390582	0.870010
C	1.877454	4.552823	0.545905
H	3.885496	3.845845	0.133822
H	3.232239	3.302493	1.671742
H	-0.233045	4.783063	1.005955
H	0.667794	3.870849	2.208312
H	2.196337	5.461072	1.064806
H	1.717239	4.830170	-0.502970

int-s11.log

B	-1.668951	3.250712	-1.200332
F	-2.240834	2.587763	-2.291520
F	-2.653120	3.819096	-0.390820

F	-0.976143	2.266496	-0.393374
F	-0.736075	4.201713	-1.621655
C	-4.418810	-1.732040	-1.056101
C	-4.104955	-0.579903	-0.338562
C	-2.808759	-0.040456	-0.399859
C	-1.850463	-0.634409	-1.224224
C	-2.188488	-1.759571	-1.992135
C	-3.464377	-2.316021	-1.889951
H	-5.414007	-2.159580	-0.985365
H	-4.859301	-0.097340	0.272580
H	0.911706	-2.341955	-2.085085
H	-1.466502	-2.196319	-2.659683
H	-3.697120	-3.199364	-2.454353
C	-0.446185	-0.173424	-1.297324
O	0.161163	0.053792	-2.311062
P	0.669295	-0.350280	0.247494
C	-0.113652	-0.965898	1.814268
C	-0.909773	0.120760	2.563909
C	-0.916089	-2.273098	1.646927
H	0.770892	-1.210030	2.419446
C	-1.481040	-0.414675	3.884957
H	-1.724296	0.489224	1.937731
H	-0.265715	0.978121	2.777606
C	-1.422575	-2.769225	3.006055
H	-1.769592	-2.105276	0.986656
H	-0.295960	-3.044854	1.190820
C	-2.281126	-1.704961	3.690380
H	-2.108296	0.362132	4.331828
H	-0.660317	-0.601690	4.589074
H	-1.985410	-3.695223	2.863461
H	-0.565741	-3.015099	3.646093
H	-2.653522	-2.069972	4.650760

H	-3.158167	-1.499578	3.065656
C	1.486926	1.255290	0.657760
C	2.494070	1.098639	1.817035
C	2.083966	2.031352	-0.531132
H	0.639571	1.846419	1.010140
C	2.982001	2.481728	2.267387
H	3.347132	0.494807	1.502583
H	2.038505	0.587589	2.665504
C	2.569224	3.404078	-0.048519
H	2.925494	1.487857	-0.957540
H	1.339310	2.154801	-1.310804
C	3.573622	3.278926	1.101931
H	3.714108	2.361560	3.067785
H	2.138644	3.033718	2.690677
H	3.015219	3.939330	-0.888187
H	1.706275	3.991770	0.271830
H	3.886343	4.269509	1.444723
H	4.476906	2.770379	0.741743
C	1.873421	-1.676541	-0.270036
C	1.210378	-2.777585	-1.128901
C	3.136179	-1.163666	-0.987310
H	2.179817	-2.113931	0.690179
C	2.181931	-3.935877	-1.390297
H	-2.576142	0.870637	0.119495
H	0.305860	-3.161377	-0.655867
C	4.118189	-2.324402	-1.201161
H	2.843576	-0.742491	-1.960159
H	3.624427	-0.365825	-0.420831
C	3.491727	-3.453891	-2.022586
H	1.692677	-4.669694	-2.042923
H	2.398524	-4.450128	-0.442862
H	5.025089	-1.951745	-1.687487

H	4.425013	-2.711902	-0.218428
H	4.196801	-4.288609	-2.102744
H	3.297724	-3.101978	-3.045035

int-s12.log

B	-3.886356	2.947555	-0.535040
F	-4.568603	2.430059	-1.542132
F	-4.091913	2.496582	0.690439
F	-2.074306	1.359137	-0.841342
F	-3.107890	3.989441	-0.730515
C	-3.058113	-3.458441	-0.129065
C	-3.342722	-2.294726	0.583894
C	-2.935277	-1.059844	0.093999
C	-2.243142	-0.991797	-1.119479
C	-1.965763	-2.159553	-1.840544
C	-2.369136	-3.390316	-1.340967
H	-3.369300	-4.420794	0.262154
H	-3.876465	-2.350018	1.524749
H	1.164626	-1.268169	-2.531424
H	-1.425364	-2.088703	-2.775362
H	-2.145196	-4.297189	-1.891207
C	-1.787243	0.283894	-1.683289
O	-1.290492	0.511013	-2.736896
P	0.921867	-0.053852	0.159768
C	0.921660	-1.199354	1.661289
C	-0.111701	-0.711702	2.693374
C	0.628560	-2.656008	1.260299
H	1.915878	-1.170796	2.123414
C	-0.224095	-1.649806	3.901346
H	-1.089355	-0.637054	2.205159
H	0.140561	0.291384	3.042837
C	0.500783	-3.586917	2.473486
H	-0.299963	-2.690838	0.681083
H	1.421438	-3.031120	0.611406
C	-0.543324	-3.082831	3.471332
H	-0.989798	-1.275886	4.587906
H	0.723379	-1.641974	4.453698
H	0.248938	-4.596770	2.133639
H	1.472681	-3.658546	2.977290
H	-0.589930	-3.743249	4.343109
H	-1.533638	-3.106959	3.001277
C	1.493667	1.548850	0.962436
C	2.839368	1.541604	1.705449
C	1.374802	2.744612	-0.001163
H	0.712213	1.705595	1.716569
C	3.095814	2.881772	2.411720
H	3.656527	1.349555	1.003431
H	2.865123	0.730229	2.437361
C	1.630173	4.070149	0.728090
H	2.096872	2.647827	-0.814173
H	0.384113	2.744832	-0.461838
C	2.988049	4.061718	1.440169
H	4.082155	2.869143	2.887712
H	2.359952	3.010295	3.215559
H	1.577053	4.902052	0.019766
H	0.836153	4.235731	1.467530
H	3.147975	5.005903	1.971615
H	3.786720	3.983776	0.690796
C	2.375495	-0.733722	-0.834960
C	1.839650	-1.780364	-1.836243
C	3.160734	0.325109	-1.628454
H	3.067666	-1.225836	-0.138732
C	2.973834	-2.444568	-2.625314
H	-3.143252	-0.155419	0.647857

H	1.239572	-2.539218	-1.334633
C	4.306490	-0.308102	-2.430270
H	2.475986	0.835602	-2.316191
H	3.569397	1.088578	-0.968276
C	3.807925	-1.403404	-3.377995
H	2.561994	-3.179981	-3.325372
H	3.619988	-2.997818	-1.930794
H	4.834549	0.468791	-2.993201
H	5.034136	-0.737537	-1.729282
H	4.652106	-1.881263	-3.885036
H	3.187683	-0.946755	-4.159440

ts-s8.log

B	0.286248	-3.603134	-1.405940
F	1.250769	-3.446171	-2.325274
F	0.606110	-4.263428	-0.276307
F	0.234664	-1.902688	-0.650177
F	-0.984150	-3.701176	-1.832666
C	4.773844	0.097592	-0.895893
C	4.155631	-0.863058	-0.096803
C	2.788194	-1.094876	-0.212138
C	2.026616	-0.367012	-1.128429
C	2.654332	0.568205	-1.952391
C	4.020892	0.805626	-1.831319
H	5.837893	0.280931	-0.801945
H	4.739245	-1.438319	0.612452
H	0.210330	2.217086	-2.141645
H	2.067771	1.097149	-2.690751
H	4.499008	1.536023	-2.473744
C	0.520860	-0.525322	-1.267973
O	-0.070728	-0.328270	-2.324371
P	-0.481495	0.493442	0.281081
C	0.359963	0.802041	1.936315
C	0.645049	-0.508867	2.688731
C	1.619778	1.677153	1.799329
H	-0.383748	1.372869	2.507121
C	1.318372	-0.240160	4.040363
H	1.297391	-1.142832	2.084510
H	-0.279396	-1.068054	2.845963
C	2.279670	1.915710	3.163638
H	2.336503	1.191856	1.132716
H	1.361851	2.638452	1.351096
C	2.589316	0.597626	3.877643
H	1.544163	-1.193236	4.527309
H	0.616249	0.290752	4.695562
H	3.192745	2.501769	3.024224
H	1.610203	2.517734	3.791009
H	3.043670	0.791749	4.853604
H	3.324025	0.032387	3.291276
C	-1.982269	-0.555877	0.697590
C	-2.879443	0.044083	1.794485
C	-2.802577	-1.015530	-0.522155
H	-1.501150	-1.451468	1.104914
C	-3.957740	-0.967625	2.210807
H	-3.360394	0.956098	1.426809
H	-2.291766	0.327797	2.670715
C	-3.859277	-2.035019	-0.079320
H	-3.305307	-0.162177	-0.980420
H	-2.151878	-1.447354	-1.278750
C	-4.771304	-1.461069	1.009847
H	-4.613780	-0.514584	2.960358
H	-3.470875	-1.823990	2.693417
H	-4.448188	-2.346463	-0.946696
H	-3.356631	-2.934118	0.296908
H	-5.499529	-2.210782	1.333390

H	-5.345627	-0.622994	0.594458
C	-0.949764	2.206990	-0.334804
C	0.157836	2.806599	-1.223976
C	-2.299808	2.291606	-1.066118
H	-1.020379	2.799411	0.586750
C	-0.156442	4.265468	-1.573522
H	2.318795	-1.854675	0.395337
H	1.134802	2.737237	-0.745223
C	-2.622149	3.746433	-1.435373
H	-2.252925	1.679816	-1.971476
H	-3.103171	1.893978	-0.446434
C	-1.512362	4.385136	-2.275235
H	0.639102	4.666879	-2.208310
H	-0.162483	4.867940	-0.656152
H	-3.575004	3.780595	-1.971801
H	-2.758927	4.326248	-0.513567
H	-1.746843	5.434301	-2.478293
H	-1.458607	3.878238	-3.246200

int-s13a.log

C	5.781418	-0.809626	0.006375
C	5.334550	0.511561	0.008081
C	3.969754	0.787830	0.003460
C	3.042806	-0.259516	-0.002673
C	3.495841	-1.582563	-0.004486
C	4.859542	-1.857302	-0.000067
H	6.844574	-1.022390	0.010094
H	6.049449	1.326386	0.013297
H	3.610786	1.808323	0.005000
H	2.765235	-2.381433	-0.009217
H	5.205495	-2.884634	-0.001557
C	1.566390	-0.001269	-0.005655
O	0.752323	-0.912933	-0.006237
O	1.268421	1.283667	-0.006868
B	-0.108336	1.830507	0.000860
F	0.004953	3.220777	0.000998
F	-0.812880	1.406386	1.158834
F	-0.826560	1.408716	-1.149696
O	-3.460623	-0.494664	0.003740
C	-3.749407	0.286702	-1.245114
H	-4.708956	0.757314	-1.040574
H	-2.957903	1.033148	-1.329124
C	-3.829568	-0.642772	-2.431360
H	-4.561653	-1.433180	-2.271861
H	-4.146843	-0.052124	-3.292596
H	-2.865234	-1.081523	-2.678157
C	-3.763530	0.268410	1.257046
H	-4.720627	0.730773	1.049354
H	-2.982645	1.014592	1.352435
C	-3.840309	-0.664066	2.432244
H	-2.885787	-1.124573	2.645208
H	-4.116912	-0.072529	3.306575
H	-4.588779	-1.433805	2.282983
C	-2.101843	-1.184244	-0.002556
H	-1.578190	-0.790305	-0.866542
H	-1.584845	-0.803057	0.866521
C	-2.282368	-2.673745	-0.015735
H	-2.807327	-3.027941	0.869211
H	-2.812479	-3.012320	-0.904958
H	-1.285909	-3.111124	-0.024058

int-s12b.log

C	7.149787	-1.526537	-1.039611
C	6.800794	-0.178972	-0.973208

C	5.460685	0.202463	-0.970952
C	4.458976	-0.768324	-1.053574
C	4.814919	-2.120168	-1.137223
C	6.152023	-2.499369	-1.117608
H	8.193698	-1.818976	-1.033469
H	7.572845	0.579835	-0.918488
H	5.195908	1.248242	-0.907088
H	4.029622	-2.860578	-1.221472
H	6.417194	-3.549164	-1.171052
C	3.001447	-0.429124	-1.141931
O	2.239485	-1.179654	-1.735783
O	2.537640	0.694373	-0.622065
B	2.969475	1.506108	0.553220
F	3.811487	2.554023	0.144513
F	3.596796	0.699989	1.512703
F	1.781014	2.054465	1.087529
C	-0.616391	-0.467109	-1.054933
H	-0.415834	-1.539200	-0.955126
H	0.264671	0.030823	-0.638799
C	-0.748690	-0.093190	-2.537743
H	-1.591704	-0.565267	-3.032061
H	-0.803029	0.974735	-2.692073
H	0.172805	-0.440300	-3.010748
P	-2.003353	-0.099248	0.040443
C	-2.517699	1.670809	-0.095399
C	-1.299391	2.607393	-0.249329
C	-3.566573	1.963447	-1.186198
H	-2.990363	1.871033	0.875520
C	-1.744256	4.072177	-0.185290
H	-0.829609	2.426591	-1.218682
H	-0.534737	2.412958	0.499505
C	-3.995550	3.435984	-1.128118
H	-3.135234	1.755944	-2.170881
H	-4.444414	1.323929	-1.073690
C	-2.797705	4.382120	-1.251909
H	-0.871195	4.716185	-0.305385
H	-2.156077	4.283060	0.808325
H	-4.725726	3.631283	-1.917354
H	-4.505237	3.620081	-0.175287
H	-3.128439	5.421326	-1.171299
H	-2.348340	4.270435	-2.245668
C	-1.467253	-0.412986	1.791244
C	-0.492072	-1.606927	1.876486
C	-0.870081	0.796190	2.536208
H	-2.404822	-0.678617	2.296853
C	-0.191821	-1.970792	3.336339
H	0.439908	-1.330374	1.378112
H	-0.884633	-2.480289	1.355737
C	-0.638719	0.427308	4.008846
H	0.088099	1.071407	2.086037
H	-1.525819	1.668017	2.473351
C	0.318246	-0.763160	4.131223
H	0.548756	-2.773120	3.354867
H	-1.098648	-2.366815	3.806098
H	-0.235849	1.297689	4.538694
H	-1.602952	0.183890	4.480911
H	0.454335	-1.034504	5.182863
H	1.302461	-0.465287	3.747461
C	-3.488112	-1.170979	-0.260825
C	-3.784763	-1.389588	-1.758449
C	-3.436566	-2.541657	0.444972
H	-4.315437	-0.598531	0.182310
C	-5.125441	-2.110130	-1.945823
H	-2.987894	-2.008809	-2.181523

H -3.786755 -0.451680 -2.311624
C -4.765764 -3.289087 0.268480
H -2.627886 -3.137602 0.010293
H -3.223463 -2.436340 1.508398
C -5.142120 -3.451555 -1.207177
H -5.303563 -2.264492 -3.014816
H -5.937765 -1.468651 -1.579721
H -4.688307 -4.269488 0.745407
H -5.556258 -2.743690 0.795727
H -6.123736 -3.924702 -1.297648
H -4.421792 -4.126274 -1.686162

ts-s9a.log

C 5.574290 -0.923047 -0.018542
C 5.189785 0.416218 0.039977
C 3.840094 0.755711 0.040013
C 2.866740 -0.247174 -0.018759
C 3.256617 -1.588829 -0.077462
C 4.605792 -1.925798 -0.077280
H 6.626091 -1.185293 -0.018377
H 5.941527 1.195374 0.085609
H 3.528589 1.790444 0.084946
H 2.491896 -2.353453 -0.122352
H 4.904017 -2.966703 -0.122785
C 1.410145 0.088645 -0.020142
O 0.558274 -0.803147 -0.068208
O 1.167806 1.371542 0.034095
B -0.176231 2.027012 0.067993
F 0.051944 3.393155 0.155172
F -0.906592 1.573627 1.191825
F -0.894249 1.719379 -1.112030
O -3.376608 -0.662898 0.017634
C -3.821683 0.178563 -1.073688
H -4.843474 0.471301 -0.827418
H -3.188567 1.067798 -1.100151
C -3.789905 -0.588963 -2.377969
H -4.387405 -1.500257 -2.310825
H -4.214246 0.044872 -3.159806
H -2.775388 -0.848600 -2.682407
C -3.712924 -0.135108 1.316115
H -4.754898 0.182774 1.250428
H -3.083896 0.737264 1.505986
C -3.554322 -1.201187 2.379932
H -2.514406 -1.499555 2.517876
H -3.907325 -0.793784 3.329936
H -4.150478 -2.084265 2.141437
C -1.630992 -1.024886 -0.110455
H -1.439174 -0.380765 -0.956490
H -1.338255 -0.594312 0.831166
C -1.616564 -2.505313 -0.292828
H -2.032506 -3.021274 0.572229
H -2.164356 -2.801788 -1.187106
H -0.574739 -2.807246 -0.408219

ts-s9b.log

C 6.545231 -1.966806 -0.733225
C 6.451558 -0.576446 -0.712655
C 5.224002 0.046999 -0.917218
C 4.082032 -0.723961 -1.155423
C 4.182972 -2.120277 -1.190394
C 5.407185 -2.739426 -0.970289
H 7.501938 -2.448448 -0.566128
H 7.334693 0.025036 -0.532612
H 5.150511 1.124789 -0.888521

H	3.293370	-2.703648	-1.389340
H	5.477905	-3.820789	-0.986285
C	2.755458	-0.102023	-1.416715
O	1.862508	-0.743317	-1.993270
O	2.517742	1.139818	-1.062976
B	2.804330	1.848277	0.236850
F	3.909469	2.690693	0.097276
F	3.009400	0.896093	1.247662
F	1.651965	2.605660	0.510115
C	-0.101244	-0.429174	-1.381336
H	0.105686	-1.424669	-1.037158
H	0.480423	0.364351	-0.950139
C	-0.672590	-0.239622	-2.739692
H	-1.430203	-0.983691	-2.981045
H	-1.110187	0.750951	-2.865620
H	0.133481	-0.335193	-3.469307
P	-1.835889	-0.126829	0.100227
C	-2.702549	1.541441	-0.108496
C	-1.683050	2.628460	-0.497304
C	-3.879417	1.524267	-1.096307
H	-3.101891	1.766200	0.888066
C	-2.356072	4.005084	-0.545380
H	-1.275290	2.398445	-1.486465
H	-0.834123	2.647751	0.183533
C	-4.549452	2.905014	-1.152008
H	-3.517954	1.259874	-2.095848
H	-4.618586	0.772715	-0.810797
C	-3.548899	4.009915	-1.506352
H	-1.620441	4.757629	-0.841530
H	-2.695684	4.275750	0.462191
H	-5.368983	2.882469	-1.876523
H	-4.998662	3.121663	-0.174828
H	-4.044908	4.984842	-1.495064
H	-3.186021	3.852825	-2.529843
C	-1.107621	-0.167370	1.842460
C	0.093720	-1.131251	1.894955
C	-0.716574	1.199484	2.427072
H	-1.918214	-0.573570	2.459442
C	0.591194	-1.299428	3.336094
H	0.905682	-0.717849	1.290588
H	-0.163346	-2.105462	1.472457
C	-0.225699	1.030185	3.872680
H	0.082126	1.645345	1.832371
H	-1.565083	1.887306	2.410896
C	0.951667	0.053588	3.957794
H	1.458997	-1.965198	3.344254
H	-0.190068	-1.786908	3.934113
H	0.063423	2.007816	4.268954
H	-1.052759	0.666321	4.496824
H	1.257809	-0.079037	4.999970
H	1.805779	0.474662	3.418694
C	-3.188506	-1.475694	0.080321
C	-3.556060	-1.889455	-1.354859
C	-2.827019	-2.724427	0.900828
H	-4.053856	-0.990960	0.550365
C	-4.734042	-2.872192	-1.353790
H	-2.687088	-2.376281	-1.810225
H	-3.792992	-1.022508	-1.971646
C	-3.997542	-3.718633	0.910480
H	-1.949038	-3.207756	0.457973
H	-2.565419	-2.463947	1.927152
C	-4.424010	-4.110588	-0.507489
H	-4.970865	-3.161138	-2.381843
H	-5.622961	-2.367710	-0.955228

H	-3.714970	-4.606424	1.483617
H	-4.847012	-3.263078	1.434249
H	-5.292307	-4.774757	-0.471761
H	-3.613794	-4.676664	-0.983602

ts-s14a.log

C	5.383615	-0.126688	-0.343360
C	4.764068	1.121383	-0.411249
C	3.384310	1.220807	-0.272650
C	2.615813	0.071250	-0.066851
C	3.239274	-1.180386	-0.000084
C	4.620393	-1.275805	-0.138016
H	6.459625	-0.202850	-0.448936
H	5.356358	2.014082	-0.572355
H	2.885043	2.179909	-0.323811
H	2.639614	-2.066525	0.158560
H	5.102308	-2.245072	-0.084505
C	1.140433	0.226648	0.072597
O	0.526066	-0.956265	0.237577
O	0.558528	1.289222	0.033874
O	-3.826160	0.242255	-0.062640
C	-4.240590	-0.584783	-1.139980
H	-5.132184	-0.154238	-1.630326
H	-3.437057	-0.627378	-1.895456
C	-4.539033	-1.972759	-0.608112
H	-5.396115	-1.958988	0.064420
H	-4.753820	-2.659494	-1.439992
H	-3.676864	-2.362136	-0.061574
C	-3.579418	1.589172	-0.460180
H	-4.516283	2.026313	-0.825060
H	-2.863584	1.605416	-1.292415
C	-3.036527	2.369588	0.720155
H	-2.138458	1.873282	1.110579
H	-2.756361	3.387451	0.403744
H	-3.795831	2.448382	1.516875
C	-0.930057	-0.986051	0.369567
H	-1.246435	-1.916257	-0.109549
H	-1.364903	-0.146162	-0.190781
C	-1.334703	-0.965601	1.837000
H	-0.956473	-0.061918	2.326933
H	-2.429532	-0.936776	1.891607
H	-0.971989	-1.839391	2.378524

ts-s14b.log

C	7.165925	-1.691101	-0.782233
C	6.879265	-0.335411	-0.630967
C	5.564635	0.111626	-0.679206
C	4.530730	-0.807214	-0.898654
C	4.821496	-2.170790	-1.065247
C	6.135934	-2.609030	-0.995270
H	8.192804	-2.034140	-0.733833
H	7.680213	0.375959	-0.470162
H	5.344319	1.162435	-0.560762
H	4.017249	-2.873739	-1.235249
H	6.360042	-3.662747	-1.107443
C	3.137108	-0.359116	-0.997020
O	2.370494	-1.155107	-1.687998
O	2.645598	0.689827	-0.510308
B	3.069637	1.641441	0.703767
F	3.901769	2.611222	0.174772
F	3.688233	0.839088	1.640519
F	1.866303	2.150126	1.132393
C	0.922993	-0.850714	-1.789852
H	0.482993	-1.840323	-1.926385

H	0.584006	-0.433150	-0.848020
C	0.661298	0.065289	-2.964096
H	-0.406578	0.243817	-3.046730
H	1.153656	1.014846	-2.849391
H	1.006972	-0.385640	-3.889935
P	-2.095661	-0.159864	-0.028706
C	-2.673019	1.636777	-0.131671
C	-1.444854	2.537099	-0.374133
C	-3.739641	1.918373	-1.202503
H	-3.114490	1.896415	0.838431
C	-1.825880	4.020959	-0.385519
H	-1.003961	2.269828	-1.342384
H	-0.669667	2.357952	0.369517
C	-4.114522	3.407641	-1.247808
H	-3.366220	1.614427	-2.188345
H	-4.641197	1.331044	-1.008057
C	-2.888281	4.302923	-1.451258
H	-0.935857	4.634775	-0.553402
H	-2.217872	4.298542	0.601516
H	-4.846026	3.575548	-2.045063
H	-4.607678	3.681178	-0.306481
H	-3.180191	5.357870	-1.442065
H	-2.458420	4.103107	-2.441807
C	-1.750789	-0.366721	1.822252
C	-0.839527	-1.593861	2.024768
C	-1.118076	0.857671	2.505890
H	-2.711813	-0.558639	2.316434
C	-0.586138	-1.872249	3.510212
H	0.118895	-1.399171	1.528532
H	-1.261453	-2.477475	1.543598
C	-0.823122	0.591918	3.990180
H	-0.183745	1.127398	2.003498
H	-1.784060	1.721843	2.430049
C	0.044729	-0.654112	4.190651
H	0.053293	-2.753727	3.625366
H	-1.541274	-2.109188	3.997740
H	-0.327307	1.468814	4.419462
H	-1.769495	0.465920	4.533089
H	0.198402	-0.843702	5.257664
H	1.035402	-0.475319	3.754350
C	-3.703872	-1.130511	-0.249895
C	-3.923153	-1.395378	-1.752691
C	-3.749333	-2.462328	0.518549
H	-4.526264	-0.507406	0.124236
C	-5.229393	-2.150460	-2.022271
H	-3.077459	-1.984309	-2.127570
H	-3.916797	-0.460530	-2.313406
C	-5.050439	-3.231541	0.248805
H	-2.897874	-3.087559	0.222714
H	-3.655536	-2.287214	1.592782
C	-5.274265	-3.469853	-1.247261
H	-5.340688	-2.332250	-3.095982
H	-6.077330	-1.522861	-1.720116
H	-5.029212	-4.184719	0.786533
H	-5.895240	-2.661234	0.654352
H	-6.225804	-3.983802	-1.414103
H	-4.486328	-4.133095	-1.626633

ts-s10.log

C	5.118596	-0.354753	0.315558
C	4.527165	0.890413	0.466935
C	3.172085	1.045917	0.244237
C	2.395932	-0.051353	-0.129267
C	2.996043	-1.304643	-0.278754

C	4.352575	-1.448995	-0.057716
H	6.177714	-0.473347	0.490307
H	5.125690	1.738969	0.761604
H	2.694600	2.005891	0.362571
H	2.394481	-2.152566	-0.564840
H	4.816361	-2.417205	-0.173638
C	0.936437	0.109530	-0.328723
O	0.314104	-1.020742	-0.516573
O	0.391518	1.220124	-0.301966
C	-1.853549	2.804159	-0.138067
H	-0.950538	3.281728	-0.512516
H	-2.061893	3.162230	0.865938
H	-2.670997	3.086357	-0.802176
C	-1.615875	1.349255	-0.181396
H	-1.659189	0.869051	-1.143609
H	-1.483458	0.801271	0.734693
F	-3.467427	0.693612	-0.009541
B	-3.389989	-0.780550	0.207359
F	-4.625013	-1.281666	0.116947
F	-2.780696	-0.945463	1.415560
F	-2.525969	-1.227092	-0.786684
H	-0.662321	-0.986375	-0.610195

Int-s13.log

C	5.013166	-0.568882	0.079970
C	4.500386	0.728853	0.139159
C	3.129538	0.935868	0.085311
C	2.267692	-0.166531	-0.025887
C	2.783291	-1.469365	-0.083357
C	4.156415	-1.664777	-0.031501
H	6.084587	-0.727546	0.118423
H	5.170540	1.574443	0.231662
H	2.721887	1.936655	0.136481
H	2.106519	-2.308114	-0.178701
H	4.558302	-2.669149	-0.087937
C	0.826030	0.035065	-0.090750
O	0.062705	-0.988752	-0.081434
O	0.424659	1.260061	-0.145260
C	-1.045117	3.151053	-0.021963
H	-0.514111	3.538649	0.848236
H	-2.086844	3.464011	0.037304
H	-0.605690	3.577725	-0.921153
C	-1.009172	1.646841	-0.052679
H	-1.529681	1.243709	-0.919544
H	-1.409778	1.194432	0.853564
F	-3.776807	0.614696	-0.406233
B	-3.508699	-0.666791	0.077870
F	-4.522941	-1.564438	-0.198351
F	-3.154729	-0.636733	1.429420
F	-2.281709	-1.137127	-0.650512
H	-0.958859	-0.917673	-0.252866

8 References

[1] a) D. J. Morgan, *Surf. Interf. Anal*. 2023, **55**, 331-335 b) D. J. Morgan. S. Uthayasekaren, *Surf. Interf. Anal*. 2023, **55**, 556-563

[2] N. Fairley, V. Fernandez, M. Richard-Plouet, C. Guillot-Deudon, J. Walton, E. Smith, D. Flahaut, M. Greiner, M. Biesinger, S. Tougaard, D. Morgan, J. Baltrusaitis, *Appl. Surf. Sci. Adv*. 2021, **5**, 100112-100121

[3] C. J. Tanuma, D. R. Powell, *Surf. Interf. Anal*., 1994, **21**, 165-176

[4] K. Li, R. Li, Y. Cui, C. Liu, *Org. Biomol. Chem.* 2024, **22**, 1693-1698

[5] I. A. Bashir, S. Lee, *J. Org. Chem.* 2023, **88**, 6159-6167

[6] D. E. Fagnani, D. Kim, S. I. Camarero, J. F. Alfaro, A. J. McNeil, *Nat. Chem*. 2023, **15**, 222-229

[7] D. Sheldon, J. M. Parr, M. R. Crimmin, *J. Am. Chem. Soc*. 2023, **145**, 10486-10490

[8] S. E. S. Farley, D. Mulryan, F. Rekhroukh, A. Phanopolous, M. R. Crimmin, *Angew. Chem. Int. E*d., 2024, **63**, e202317550

[9] G. H. Kim, D. H. Kim, Y. Lee, J. W. Yang, *Adv. Synth. Catal*. 2025, **367**, e202400103

[10] H. Hattori, K. Ishida, Y. Ogiwara, N. Sakai, *Eur. J. Org. Chem*. 2022, **2022**, e202201118

[11] Y. Liang, Z. Zhao, A. Taya, N. Shibata, *Org. Lett*. 2021, **23**, 847-852

[12] M. B. Röthel, A. Schöler, F. Buß, P. Löwe, F. Dielmann, *Chem. Eur. J*. 2024, **30**, e202404048

[13] Y. Zhang, L. Sun, Z. Xu, T. Tan, Z. Wang, *Org. Lett.* 2024, **26**, 6164-6168

[14] J. Sommer, Y. Yang, D. Rambow, J. Blümel, *Inorg. Chem*. 2004, **43**, 7561-7563

[15] D. Li, M. Liao, *Polym. Degred. Stab*. 2018, **152**, 116-125

[16] Gaussian 16, Revision C.01, M. J. Frisch, G. W. Trucks, H. B. Schlegel, G. E. Scuseria, M. A. Robb, J. R. Cheeseman, G. Scalmani, V. Barone, G. A. Petersson, H. Nakatsuji, X. Li, M. Caricato, A. V. Marenich, J. Bloino, B. G. Janesko, R. Gomperts, B. Mennucci, H. P. Hratchian, J. V. Ortiz, A. F. Izmaylov, J. L. Sonnenberg, D. Williams-Young, F. Ding, F. Lipparini, F. Egidi, J. Goings, B. Peng, A. Petrone, T. Henderson, D. Ranasinghe, V. G. Zakrzewski, J. Gao, N. Rega, G. Zheng, W. Liang, M. Hada, M. Ehara, K.

Toyota, R. Fukuda, J. Hasegawa, M. Ishida, T. Nakajima, Y. Honda, O. Kitao, H. Nakai, T. Vreven, K. Throssell, J. A. Montgomery, Jr., J. E. Peralta, F. Ogliaro, M. J. Bearpark, J. J. Heyd, E. N. Brothers, K. N. Kudin, V. N. Staroverov, T. A. Keith, R. Kobayashi, J. Normand, K. Raghavachari, A. P. Rendell, J. C. Burant, S. S. Iyengar, J. Tomasi, M. Cossi, J. M. Millam, M. Klene, C. Adamo, R. Cammi, J. W. Ochterski, R. L. Martin, K. Morokuma, O. Farkas, J. B. Foresman, and D. J. Fox, Gaussian, Inc., Wallingford CT, 2016.

[17] (a) A. D. Becke, *J. Chem. Phys*. 1993, **98**, 5648–5652. (b) C. Lee, W. Yang, R. G. Parr, *Phys. Rev. B,* 1988, **37**, 785–789. (c) S. H. Vosko, L. Wilk, M. Nusair, *Can. J. Phys*. 1980, **58**, 1200– 1211. (d) P. J. Stephens, F. J. Devlin, C. F. Chabalowski, M. J. Frisch, *J. Phys. Chem*. 1994, **98**, 11623–11627

[18] S. Grimme, J. Antony, S. Ehrlich, H. Krieg, *J. Chem. Phys*. 2010, **132**, 154104

[19] (a) A. D. Becke, E. R. Johnson, *J. Chem. Phys*. 2005, **122**, 154101. (b) E. R. Johnson, A. D. Becke, *J. Chem. Phys*. 2005, **123**, 024101. (c) E. R. Johnson, A. D. Becke, *J. Chem. Phys*. 2006, **124**, 174104. (d) S. Grimme, S. Ehrlich, L. Goerigk, *J. Comput. Chem*. 2011, **32**, 1456

[20] (a) R. Krishnan, J. S. Binkley, R. Seeger, J. A. Pople, *J. Chem. Phys*. 1980, **72**, 650–654. (b) T. Clark, J. Chandrasekhar, G. W. Spitznagel, P. V. R. Schleyer, *J. Comput. Chem*. 1983, **4**, 294– 301

[21] (a) F. Weigend, F. Furche, R. Ahlrichs, *J. Chem. Phys*. 2003, **119**, 12753–12762. (b) D. Rappoport, F. Furche, *J. Chem. Phys.* 2010, **133**, 134105

[22] G. Scalmani, M. J. Frisch, *J. Chem. Phys*. 2010, **132**, 114110

[23] A. V. Marenich, C. J. Cramer, D. G. Truhlar, *J. Phys. Chem. B,* 2009, **113**, 6378–6396.

[24] (a) D. Feller, *J. Comput. Chem*. 1996, **17**, 1571–1586. (b) K. L. Schuchardt, B. T. Didier, T. Elsethagen, L. Sun, V. Gurumoorthi, J. Chase, J. Li, T. L. Windus, *J. Chem. Inf. Model*. 2007, **47**, 1045–1052. (c) B. P. Pritchard, D. Altarawy, B. Didier, T. D. Gibson, T. L. Windus, *J. Chem. Inf. Model*. 2019, **59**, 4814–4820.

[25] G. Luchini, J. V. Alegre-Requena, I. Funes-Ardoiz, R. S. Paton, GoodVibes: Automated Thermochemistry for Heterogeneous Computational Chemistry Data. *F1000Research*, 2020, **9**, 291
